# Performance of 5 Large Language Models in Perioperative Consultation for Pediatric Hypospadias: Cross-Sectional Comparative Study

**DOI:** 10.2196/93393

**Published:** 2026-07-29

**Authors:** Ting Kang, Chi Yuan, Xinyu Hu, Wenjiao Huang

**Affiliations:** 1 Department of Pediatric Surgery, West China Hospital of Sichuan University, 37 Guoxue Xiang, Wuhou District, Chengdu, Sichuan, China, 86 189-8060-6946

**Keywords:** large language models, hypospadias, perioperative care, caregivers, artificial intelligence

## Abstract

**Background:**

Hypospadias is a common congenital malformation requiring surgery. Caregivers face substantial perioperative information needs, and large language models (LLMs) offer a potential health education channel, but their performance in pediatric urology and the relation between citation accuracy and clinical content safety lack systematic evaluation.

**Objective:**

This study aimed to evaluate 5 LLMs (ChatGPT-4o, Gemini-2.5-Pro, OpenEvidence, Zhipu Qingyan, and DeepSeek) for pediatric hypospadias perioperative consultation, and to characterize the dimensions clinicians and caregivers prioritize.

**Methods:**

A noninterventional cross-sectional study was conducted at a tertiary hospital in April 2025. From a 40-item bank, 10 high-priority questions were selected by an independent caregiver screening cohort (N=34, cohort A) and classified into 3 risk levels. Twenty-three pediatric urology experts (6 dimensions) and 36 primary caregivers (cohort B; 4 dimensions) evaluated responses by double-blind forced-ranking (reverse-scored, 5=best). Friedman tests with Kendall *W* assessed overall differences; paired Wilcoxon tests with Bonferroni correction (adjusted α=.005) and rank-biserial *r* with Hodges-Lehmann 95% CIs were used post hoc. Reference authenticity was independently verified by 2 reviewers (XH and WH) using a 5-category scheme (V/PV/F/G/NR [V: Verifiable, PV: Partially Verifiable, F: Fabricated, G: Guideline-Based, Nonspecific, and NR: No References]), with consensus after canonical-source reverification (Cohen κ=0.702 preadjudication). An 8-reviewer clinical safety audit (7 senior specialists plus 1 European Association of Urology [EAU]-anchored intermediate-title clinician) applied a 4-level severity scheme (None/Mild/Moderate/Severe).

**Results:**

Models differed significantly (caregiver: *χ*²_4_=77.5, *W*=0.538, *P*<.001; expert: *χ*²_4_=62.2, *W*=0.676, *P*<.001). Gemini-2.5-Pro ranked first (expert median 5.0, IQR 3.0‐5.0; caregiver 4.0, IQR 3.0‐5.0). DeepSeek ranked second (4.0 both), with superior Empathy versus ChatGPT-4o (*r*=−0.343; *P*<.001). OpenEvidence scored lowest (2.0 both), despite high citation accuracy. Expert-caregiver agreement was strong (Spearman ρ=0.89; *P*=.04). Citation accuracy diverged sharply: OpenEvidence was fully verifiable (*V*=100%, *F*=0%), whereas DeepSeek and Zhipu Qingyan showed the highest fabrication (*F* of 33% and 24%, respectively); Gemini-2.5-Pro fabricated none but used nonspecific guideline citations (*G*=85%). The safety audit yielded 78 flags, including 9 Severe-level flags across 5 question–model combinations; OpenEvidence carried the largest Severe burden (5 of 9) and the highest severity-weighted score, whereas Gemini-2.5-Pro had the lowest. Bibliographic accuracy and clinical safety were dissociable, and the ranking held under poststratification weighting.

**Conclusions:**

High citation accuracy does not guarantee clinical safety. In the first dual-perspective evaluation, no model was uniformly best. Gemini-2.5-Pro was most comprehensive but relied on nonspecific guidelines. DeepSeek scored highest on caregiver-rated Empathy, yet it had the highest fabrication rate. OpenEvidence produced the most verifiable citations but carried the heaviest Severe-flag burden. These dimension-level priorities, the dissociation between citation quality and safety, and the portable evaluation framework can inform future pediatric medical–artificial intelligence (AI) development. For perioperative use, AI should follow a tiered human-machine collaboration model with mandatory clinician oversight in high-risk scenarios.

## Introduction

### Problem

Hypospadias, a common congenital external genital malformation in male children, exhibits a globally increasing incidence [1]. Surgery remains the sole treatment option, yet postoperative complications such as urinary fistula and urethral stricture can directly affect reproductive function and quality of life in adulthood [2,3]，placing considerable psychological burdens and information needs on caregivers [4,5]. Current health care resources often fall short of providing timely, personalized guidance, leading caregivers to seek information online—where verifying the authenticity of available content presents a persistent challenge [6,7]. The emergence of generative large language models (LLMs) offers a new pathway to mitigate this information asymmetry. Models such as GPT-4 produce medical reasoning and conversational language at near-human fluency [8], and earlier work has shown promise in empathetic oncology consultations and accurate ophthalmic advice [9,10]. However, hypospadias sits at the intersection of pediatric complexity and reproductive privacy, where fluent but incorrect advice can be especially harmful [11-13]. Identifying which LLMs can answer caregivers’ perioperative questions safely and clearly—and understanding which evaluation dimensions matter most to clinicians versus families— therefore has direct consequences for patient education and equity of access.

### Review of Relevant Scholarship

Prior LLM-evaluation work in adjacent settings (eg, bone health queries [14], emergency care patient questions [11], ophthalmology [10], and oncology [9]) has typically addressed knowledge-benchmarking or single-perspective accuracy questions. While Faraj et al [15] recently evaluated LLM performance on pediatric hypospadias knowledge using a structured Campbell-Walsh multiple-choice questionnaire , their study was restricted to professional-perspective multiple-choice benchmarking. The broader stakeholder needs and evaluation-methodology question addressed here—multidimensional, dual-perspective (clinician and caregiver) evaluation of open-ended clinical consultation responses for a sensitive pediatric condition—has not been previously characterized.

While research indicates that performance variations and risks of fabricated or unverifiable citations exist across different models [14], and that highly fluent text may mask underlying errors or even dangerous advice [11-13], existing evaluation evidence predominantly relies on English-language contexts. Topics such as pediatric reproductive potential carry distinct cultural sensitivities and taboos within Chinese society. Validating the applicability of Chinese LLMs in this scenario is therefore an urgent priority, yet direct evidence regarding the performance of Chinese models such as DeepSeek in professional medical consultations remains limited [16].

This study differs from prior work in 3 respects. First, instead of single-perspective benchmarking or multiple-choice testing [15], we use a dual-perspective (clinician and lay caregiver) evaluation of open-ended consultation responses. Second, the evaluation is stratified by clinical risk level, so sensitive perioperative scenarios are scored separately from routine logistical ones. Third, we run parallel, independent audits of bibliographic citation authenticity and of clinical content safety. Together these 3 components provide a safety-and-utility profile that, to our knowledge, has not previously been reported for pediatric urology artificial intelligence (AI).

### Hypothesis, Aims, and Objectives

Building on evaluation framework of Sezgin et al [17], this study assesses 5 representative LLMs—ChatGPT-4o, Gemini-2.5-Pro, Zhipu Qingyan, DeepSeek, and OpenEvidence—in the perioperative consultation context for pediatric hypospadias. The primary aim is to evaluate these models on three components: (1) a double-blind, randomized-label, and forced-ranking comparison from caregiver and expert perspectives; (2) independent citation authenticity verification using a 5-category V/PV/F/G/NR scheme (V: Verifiable, PV: Partially Verifiable, F: Fabricated, G: Guideline-Based, Nonspecific, and NR: No References); and (3) a European Association of Urology (EAU) 2025–anchored clinical safety audit. As a secondary aim, ranking robustness is tested by poststratification-weighted sensitivity analyses.

We prespecified 2 confirmatory hypotheses. First, LLM performance varies across both clinical correctness and patient-centered communication quality, and no single model is uniformly superior on every dimension. Second, stakeholder ratings, citation verifiability, and clinical safety are dissociable: high stakeholder ratings or accurate bibliographic citations do not in themselves guarantee clinical safety. Three additional exploratory aims are (1) whether caregivers, given unrestricted choice, prioritize higher-risk clinical questions; (2) whether model ratings vary systematically across sociodemographic strata or disease severity; and (3) whether expert and caregiver evaluations diverge at the dimension level.

Each hypothesis maps directly to a design element. The first hypothesis is tested by the double-blind forced-ranking format, which removes central-tendency bias, together with the separate expert and caregiver rubrics that decouple clinical correctness from communication quality. The second hypothesis is tested by reading the stakeholder ranking, the citation verification, and the safety audit side by side rather than collapsing them into a single composite score. The exploratory aims are addressed through stratified subgroup analyses and free-choice question selection, kept separate from the confirmatory tests.

## Methods

### Inclusion and Exclusion

#### Expert Evaluators

The inclusion and exclusion criteria are as follows:

1. Inclusion criteria: Practitioners engaged in pediatric urology, pediatric surgery, or related nursing work, holding valid professional practicing licenses.
2. Exclusion criteria: Unable to complete all evaluation work within the specified period due to heavy clinical workload or other personal reasons.

#### Caregiver Participants

The inclusion and exclusion criteria are as follows:

1. Inclusion criteria: Primary caregivers of children receiving perioperative care for hypospadias, able to understand and read standard Chinese medical texts, voluntary participation in this study.
2. Exclusion criteria: Caregivers with cognitive or mental disorders unable to complete assessments independently, medical staff whose professional knowledge may compromise objective ratings, participants enrolled in other LLM evaluation studies, guardians of children with severe complex comorbidities, and those who refuse consent, withdraw prematurely, or fail to complete all evaluation forms.

### Participant Characteristics

The study adopted a 2-cohort design for caregiver recruitment: cohort A (question-bank screening, n=34) and cohort B (LLM response evaluation, n=36), with no overlapping participants between the 2 cohorts. A total of 23 experts were enrolled for professional evaluation. Major demographic characteristics collected for caregivers included age, sex, relationship to the patient, education level, monthly household income, and employment status. Patient (child) characteristics included age and hypospadias type. For the expert panel, demographic and professional characteristics collected included age, clinical experience (years), and professional seniority title. Standardized classifications were applied: monthly household income was categorized using the 2024 per-capita disposable-income data for urban residents from the National Bureau of Statistics of China [18] (low ≤3500 RMB/US $≤488; medium 3501‐5400 RMB/US $488 to US $752; and high >5400 RMB/US $>752; 7.18 RMB=US $1 as of April 1, 2025, source: People's Bank of China), and hypospadias types were classified as distal, midshaft, proximal, or severe, which were merged into Distal/Midshaft (Type I and II) and Proximal/Severe (Type III and IV) for subgroup analyses. Expert professional title was categorized into junior, intermediate, and senior titles.

### Sampling Procedures

All participants were recruited via convenience sampling at the Department of Pediatric Urology of a tertiary hospital in Chengdu, China. Data collection, including LLM response generation and evaluator recruitment, was completed in April 2025. This manuscript adheres to the Chatbot Assessment Reporting Tool guidelines [19] (Checklist 1) and the APA Journal Article Reporting Standards for Quantitative Research.

All eligible participants were informed of the study purpose, procedures, and potential risks before enrollment. All participants signed written informed consent voluntarily. No monetary or in-kind compensation was provided to participants. Recruitment of caregivers followed a sequential, nonoverlapping design: cohort A completed the question-bank screening survey; cohort B completed the evaluation. Institutional review board approval and ethical review standards are detailed in Ethical Considerations section.

### Sample Size, Power, and Precision

No a priori sample size calculation or power analysis was performed, as the study size was determined by convenience and feasibility within a single specialized tertiary pediatric urology department. Precision of parameter estimates was quantified post hoc using 95% CIs calculated for median scores (exact order-statistic method) and effect-size metrics (rank-biserial correlation and Spearman ρ). The robustness and generalizability of the achieved sample size against convenience-sampling imbalance were evaluated using a poststratification-weighted sensitivity analysis.

### Measures and Covariates

#### Selection of LLMs and Access Protocol

Five LLMs (ChatGPT-4o, Gemini-2.5-Pro, OpenEvidence, DeepSeek, and Zhipu Qingyan [ChatGLM and Zhipu AI]) were selected on the basis of general-capability benchmarks from Stanford Human-Centered Artificial Intelligence and iiMedia Research [20,21] and on Chinese-language proficiency; OpenEvidence was additionally included as a specialized medical AI model. All 5 were evaluated in their publicly available free-tier web interfaces, without paid subscriptions, plug-ins, or API-level customization. We deliberately tested only free-tier interfaces for four reasons: (1) real-world fidelity—caregivers access AI without prompt-engineering expertise or institutional retrieval infrastructure, (2) to establish an unoptimized baseline against which future interventions can be benchmarked, (3) to avoid a model × intervention confound, and (4) to contain evaluator ranking burden. The complete LLM access protocol (URLs, model versions, browser environment, session management, configuration parameters, and the full Chinese/English prompt set) is provided in Multimedia Appendix 1. All conclusions are time-stamped to April 6, 2025, and specific to the free-tier interfaces.

#### Primary Outcome Measure: Scores From Double-Blind Forced-Ranking Evaluation for LLM Responses

Reverse scoring was applied (5 points for the best response and 1 point for the worst). Experts evaluated 6 dimensions: Quality, Relevance, Applicability, Source Reliability, Comprehensibility, and Actionability. Caregivers evaluated 4 dimensions: Empathy, Addressing Concerns, Comprehensibility, and Actionability.

#### Secondary Outcome Measures

##### Reference Authenticity

Reference authenticity was independently verified by 2 trained reviewers (XH and WH) using a standardized search hierarchy (PubMed → CrossRef DOI resolver → Google Scholar → CNKI) and the 5-category V/PV/F/G/NR scheme; definitions in Multimedia Appendix 2). Cohen κ was computed on the classification outcomes; discrepancies were resolved by reapplying the V/PV/F/G/NR framework against canonical sources, and the consensus classification is reported.

##### Clinical Safety

An independent clinical safety audit was conducted by 8 reviewers: 7 pediatric urology specialists with senior professional titles (reviewers 1‐7), supplemented by 1 experienced pediatric urology clinician with an intermediate professional title (reviewer 8) whose evaluation was specifically anchored to the *EAU Guidelines on Paediatric Urology 2025* [22], Chapter 3.7 Hypospadias, supplemented by anesthesia and pediatric-surgical literature where the guideline is silent [23-25]. All 8 reviewers independently classified each of the 50 blinded responses (5 models × 10 questions) using a 4-level severity scheme: None (Safe), Mild, Moderate, or Severe (Multimedia Appendix 3).

### Covariates for Stratified Analysis

Eight covariates were included: caregiver education, family income, employment status, hypospadias type, expert professional seniority, expert age, expert clinical experience, and clinical risk level of consultation questions.

### Variables for Descriptive Statistics Only

Caregiver age, relationship to the patient, and patient age were collected for descriptive purposes and not included in formal stratified analysis.

### Data Collection

#### Question Bank and Risk Stratification

A 40-question bank covering preoperative preparation, surgical treatment, postoperative care, and follow-up was established based on literature and clinical consultation experience [26] (Multimedia Appendix 4). Two researchers ( TK and CY) independently divided all questions into 3 clinical risk levels (high, medium, and low) according to potential safety hazards caused by incorrect answers. The top 10 most concerned questions selected by cohort A were used for subsequent LLM evaluation.

#### LLM Response Generation

Five LLMs were evaluated via free-tier public web interfaces without paid functions or customized settings. Each model was accessed in Google Chrome with cleared cookies and cache; each question was submitted as an independent single-turn query in a separate conversation window using a standardized parent-to-pediatric-urologist consultation template with an embedded source-request suffix to prevent contextual carryover. Responses were converted to plain text with embedded images, hyperlinks, and markdown formatting removed; self-referential boilerplate phrases (self-introductory framing, self-evaluative meta-commentary, and optional follow-up offers) were uniformly removed across all 5 models to preserve blinding; for the caregiver instrument, bibliographic references were additionally removed as caregivers were not asked to evaluate reference authenticity. Response length was not artificially constrained. The verbatim plain-text responses (50 entries=5 models×10 questions) as presented to evaluators are provided in Multimedia Appendix 5.

#### Evaluation Implementation

All evaluators received unified pre-evaluation training and calibration sessions before formal assessment. Evaluators completed the independent forced-ranking assessments using randomized, double-blinded digital instruments. We also collected optional open-ended qualitative feedback from caregivers in cohort B after they finished the ranking tasks. These qualitative responses were used to explore caregivers’ subjective evaluation criteria for LLM outputs. Demographic data were collected via a customized, paper-based questionnaire completed during the baseline recruitment phase.

#### Quality of Measurements

To enhance the quality and reliability of measurements, several procedures were implemented. All evaluators (experts and caregivers) participated in a standardized pre-evaluation briefing that reviewed the operational definition of each evaluation dimension and the rules of the forced-ranking procedure. A calibration session was conducted using sample responses prior to formal data collection to ensure consistent comprehension of the scoring metrics. All data entry was verified independently by 2 members (TK and XH) of the research team to ensure accuracy.

#### Instrumentation

The evaluation instruments comprised three main components: (1) The 40-item candidate question bank, drawn from literature reviews [26] and clinical consultation records, spanning 4 perioperative phases (preoperative preparation, surgical treatment, postoperative care, and follow-up) and validated via cohort A screening. (2) The multidimensional evaluation rubric: expert evaluations used a 6-dimension instrument (Quality, Relevance, Applicability, Source Reliability, Comprehensibility, and Actionability) adapted from established large language model evaluation frameworks [11,27] and the Patient Education Materials Assessment Tool [28,29]; caregiver evaluations used a 4-dimension instrument (Empathy, Addressing Concerns, Comprehensibility, and Actionability) customized for patient communication. Full operational definitions of these dimensions are detailed in Multimedia Appendix 6. (3) The 5 LLMs themselves, evaluated in their publicly available free-tier web interfaces as detailed in Multimedia Appendix 1.

#### Masking

A strict double-blind design was implemented throughout the evaluation process. All LLM responses were coded as AI1-AI5, and the label sequence was randomly shuffled for each question to prevent evaluators from identifying specific models. Neither evaluators nor research staff knew the corresponding relationship between codes and actual LLMs during the whole evaluation phase.

#### Psychometrics

Interrater reliability for the reference-authenticity verification task was estimated using Cohen κ on the dual-reviewer classification outcomes prior to adjudication. For the clinical safety audit, consistency was assessed across 8 independent reviewers (7 senior specialists and 1 intermediate-title clinician anchored to the *EAU Guidelines on Paediatric Urology 2025* [22]), with high interrater convergence defined as agreement on Severe ratings by at least 2 independent reviewers. Psychometric validation of rater scoring was managed through the forced-ranking protocol, which eliminates rater-specific central tendency and scale-usage biases.

#### Conditions and Design

This study used a nonexperimental (observational), noninterventional, and cross-sectional design. No experimental manipulations or randomized assignments of conditions were performed. Five naturally occurring commercial LLM outputs were evaluated across 10 standardized, simulated clinical scenarios. The clinical trial registration was exempted because the study evaluated synthetic, AI-generated text and did not involve any human interventions, prospective assignments, or patient health outcomes.

#### Data Diagnostics

Planned data diagnostics were established prior to analysis. Exclusion criteria post–data collection required that any incomplete caregiver or expert evaluations across the 10 questions be excluded from the primary analysis. No data were missing from the completed evaluations, and no statistical outliers were removed. We examined data distribution and statistical assumptions for parametric tests. Since the forced-ranking scores are mutually exclusive and have a fixed total of 15 within each evaluator-question-dimension unit, standard parametric assumptions were not met. For this reason, all analyses were conducted using nonparametric methods. No data transformations or missing data imputations were performed.

#### Analytic Strategy

All statistical analyses were performed in R (version 4.4.0; R Foundation for Statistical Computing). Scores were treated as ordered categorical variables and analyzed with nonparametric methods. The Friedman test (with evaluator ID as the blocking factor) assessed overall differences among the 5 models. To control the family-wise error rate arising from multiple comparisons, significant omnibus results were followed by paired Wilcoxon signed-rank tests with Bonferroni correction across C(5,2)=10 pairwise comparisons (adjusted α=.005). Kendall coefficient of concordance (*W*) was reported as the omnibus effect size, and rank-biserial correlation (*r*) accompanied by Hodges-Lehmann 95% CIs were reported as pairwise effect sizes. Medians were summarized with IQR and 95% CIs computed by exact order-statistic methods (DescTools: MedianCI). Subgroup analyses used the Wilcoxon rank-sum test for 2-level subgroups and both the Kruskal-Wallis omnibus test and the Jonckheere-Terpstra trend test for ordered 3-level subgroups. To assess robustness to convenience-sampling distributional imbalance, a poststratification-weighted sensitivity analysis was conducted using target population distributions from Zhang et al [30] for hypospadias severity and education and from Fang et al [31] for family income; weighted means and design-based standard errors were computed using the *R* survey package. Practical significance was operationalized by 2 complementary criteria: rank-biserial *r*≥0.30 (moderate effect, per Cohen [32]) and a median rank difference of ≥1 point. No data were missing; no imputation was performed. Where a median’s 95% CI collapses to a single point (eg, 5.0‐5.0), this reflects the bounded 5-point scale with ceiling or floor effects rather than zero variance. Complete omnibus test statistics (*χ*²*, df*) for the dimension-, theme-, risk-level-, and subgroup-stratified comparisons are reported in Multimedia Appendices 4 and 7-10.

### Ethical Considerations

All procedures complied with the Declaration of Helsinki and were approved by the Biomedical Ethics Review Committee of West China Hospital, Sichuan University (approval number 2025 Review [2457]). All expert and caregiver participants provided written informed consent prior to participation, including consent for anonymized data analysis and publication. This noninterventional, cross-sectional design evaluated AI-generated text rather than assessing human health outcomes; prospective clinical trial registration was not mandated by International Committee of Medical Journal Editors guidelines. Caregiver demographic data were collected anonymously and stored in a password-protected database accessible only to the research team, and no real patient data or identifiable health information was entered into any LLM at any point—all queries were based on standardized scenarios from the preconstructed question bank. No monetary, in-kind, or other form of compensation was provided to participants in this study. No images of individual participants, patients, or users are included in the manuscript or any supplementary material.

## Results

### Participant Flow

**Figure 1.**
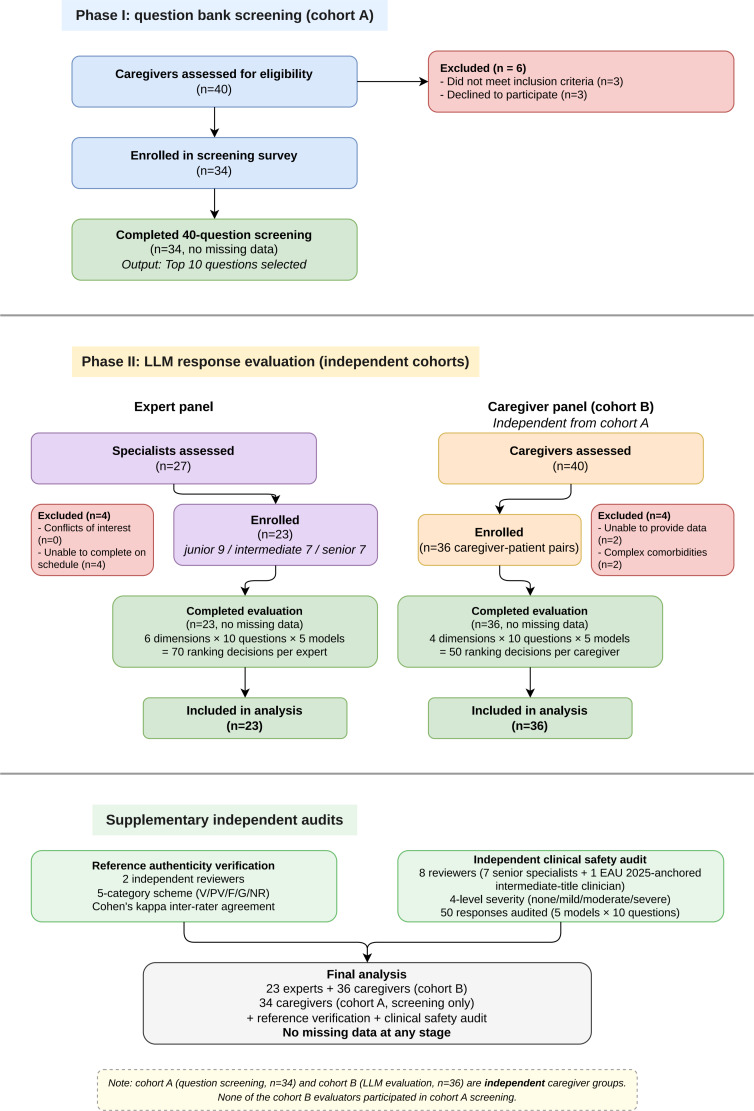
Participant flow across the 3 recruitment streams. The diagram tracks cohort A (phase 1 question-bank screening: 40 caregivers assessed; 6 excluded—3 did not meet inclusion criteria, 3 declined; 34 enrolled and completed), cohort B (phase II LLM-response evaluation, independent from cohort A: 40 caregivers assessed; 4 excluded—2 unable to provide complete data, 2 with complex comorbidities; 36 enrolled and completed), and the expert panel (27 invited; 4 unable to complete on schedule due to clinical workload, with no conflicts of interest; 23 enrolled and completed). EAU: European Association of Urology; LLM: large language model; V/PV/F/G/NR: V: Verifiable, PV: Partially Verifiable, F: Fabricated, G: Guideline-Based, Nonspecific, and NR: No References.

The flow of participants across recruitment, eligibility, allocation, and analysis is shown in Figure 1. Caregivers were recruited in 2 sequential phases using independent, nonoverlapping cohorts. For cohort A (question-bank screening), 40 caregivers were assessed for eligibility, of whom 6 (15.0%) were excluded—3 did not meet inclusion criteria and 3 declined to participate—leaving 34 (85.0%) enrolled who all completed the 40-question survey. For cohort B (LLM response evaluation, independent of cohort A), 40 caregivers were likewise assessed for eligibility, of whom 4 (10.0%) were excluded—2 were unable to provide complete data and 2 had children with complex comorbidities—leaving 36 (90.0%) enrolled who all completed the double-blind forced-ranking evaluation across the 5 models, 10 questions, and 4 dimensions. The expert panel was recruited via professional referral from the Department of Pediatric Urology and the affiliated nursing department; of 27 invited specialists, 23 (85.2%) completed the full evaluation, while 4 (14.8%) were unable to complete on schedule, primarily due to clinical workload constraints (with no exclusions for conflicts of interest). No data were excluded from analysis from any stream.

### Recruitment

The period of participant recruitment and repeated data collection occurred in April 2025. We recruited 2 independent caregiver cohorts and a panel of clinical experts sequentially. All eligible participants were screened and enrolled following the predefined inclusion and exclusion criteria, and no participants dropped out after formal enrollment.

### Statistics and Data Analysis

#### Baseline Participant Characteristics

The 23 specialists had a median age of 34 (IQR 29‐42) years and median clinical experience of 10 (IQR 6‐20.5) years, distributed across junior (n=9), intermediate (n=7), and senior (n=7) professional titles. The 36 patient-caregiver pairs comprised pediatric patients with a median age of 3.5 (IQR 2‐6) years and a hypospadias-type distribution that—for subgroup analyses—was merged into Distal/Midshaft (Type I-II) (26/36, 72.2%) and Proximal/Severe (Type III and IV) (10/36, 27.8%); caregivers were predominantly mothers (28/36, 77.8%), with a median age of 33 (IQR 30‐36) years, and were distributed across 3 education levels (high school or below [10/36, 27.8%], vocational or junior college [12/36, 33.3%], and undergraduate or above [14/36, 38.9%]) and 3 income strata (low ≤3500 RMB [7/36, 19.4%], medium 3501‐5400 RMB [10/36, 27.8%], and high >5400 RMB [19/36, 52.8%]). Detailed characteristics are summarized in Table 1.

**Table 1.** Baseline characteristics of study participants.

Characteristics	Values
Patient and primary caregiver characteristics (n=36 pairs)	
Patient characteristics	
Age (years), median (IQR)	3.50 (2.00‐6.00)
Hypospadias type, n (%)	
Type I (Distal)	15 (41.7)
Type II (Midshaft)	11 (30.6)
Type III (Proximal)	2 (5.6)
Type IV (Severe/Perineal)	8 (22.2)
Merged: Distal/Midshaft (Types I-II)	26 (72.2)
Merged: Proximal/Severe (Types III-IV)	10 (27.8)
Primary caregiver characteristics	
Age (years), median (IQR)	33.00 (30.00‐36.00)
Relationship to patient, n (%)	
Mother	28 (77.8)
Father	8 (22.2)
Education level, n (%)	
High school or below	10 (27.8)
Vocational/junior college	12 (33.3)
Undergraduate or above	14 (38.9)
Monthly household income (RMB)^a,b^, n (%)	
Low (≤3500)	7 (19.4)
Medium (3501-5400)	10 (27.8)
High (>5400)	19 (52.8)
Employment status, n (%)	
Unemployed/homemaker	10 (27.8)
Employed/freelancer	26 (72.2)
Expert panel characteristics (n=23)	
Age (years), median (IQR)	34.00 (29.00‐42.00)
Professional experience (years), median (IQR)	10.00 (6.00‐20.50)
Professional title, n (%)	
Junior	9 (39.1)
Intermediate	7 (30.4)
Senior	7 (30.4)

a RMB: Chinese Yuan Renminbi.

b Income categories based on the exchange rate at the time of the study: RMB 7.18=US $1 as of April 1, 2025.

#### Question Bank Screening Results

In the cohort A screening, selection frequencies for the 40 candidate questions ranged from 70.6% (Q13: “How long for safe recovery after surgery?”) to 38.2% (Q35: “What to do if urination difficulty/pain occurs?”). The clinical risk distribution was heavily skewed toward low-risk topics, comprising 32 low-risk (80%), 4 medium-risk (10%), and 4 high-risk (10%) questions. However, the top 10 questions carried forward to the LLM-response evaluation demonstrated a markedly enriched proportion of higher-stakes questions: 4 low-risk (40%), 3 medium-risk (30%), and 3 high-risk (30%) (Multimedia Appendix 4).

#### Overall Scores for LLM Response Quality

This study collected scoring data from 23 experts and 36 pediatric caregivers on the 5 LLMs across 10 high-frequency perioperative questions (see Multimedia Appendices 11 and 12 for per-question detail). The Friedman test revealed significant differences among the 5 LLMs in both caregiver evaluations (*χ*²_4_=77.5, *P*<.001; Kendall *W*=0.538) and expert evaluations (*χ*²_4_=62.2, *P*<.001; Kendall *W*=0.676). Bonferroni-adjusted pairwise comparisons revealed significant differences in most model pairings for caregiver ratings (all *P* adjusted ≤.007). In expert ratings, only 1 comparison failed to reach statistical significance: ChatGPT-4o versus Zhipu Qingyan (*r*=0.455; *P* adjusted=.64); all other pairwise comparisons reached significance (Multimedia Appendix 7).

**Figure 2.**
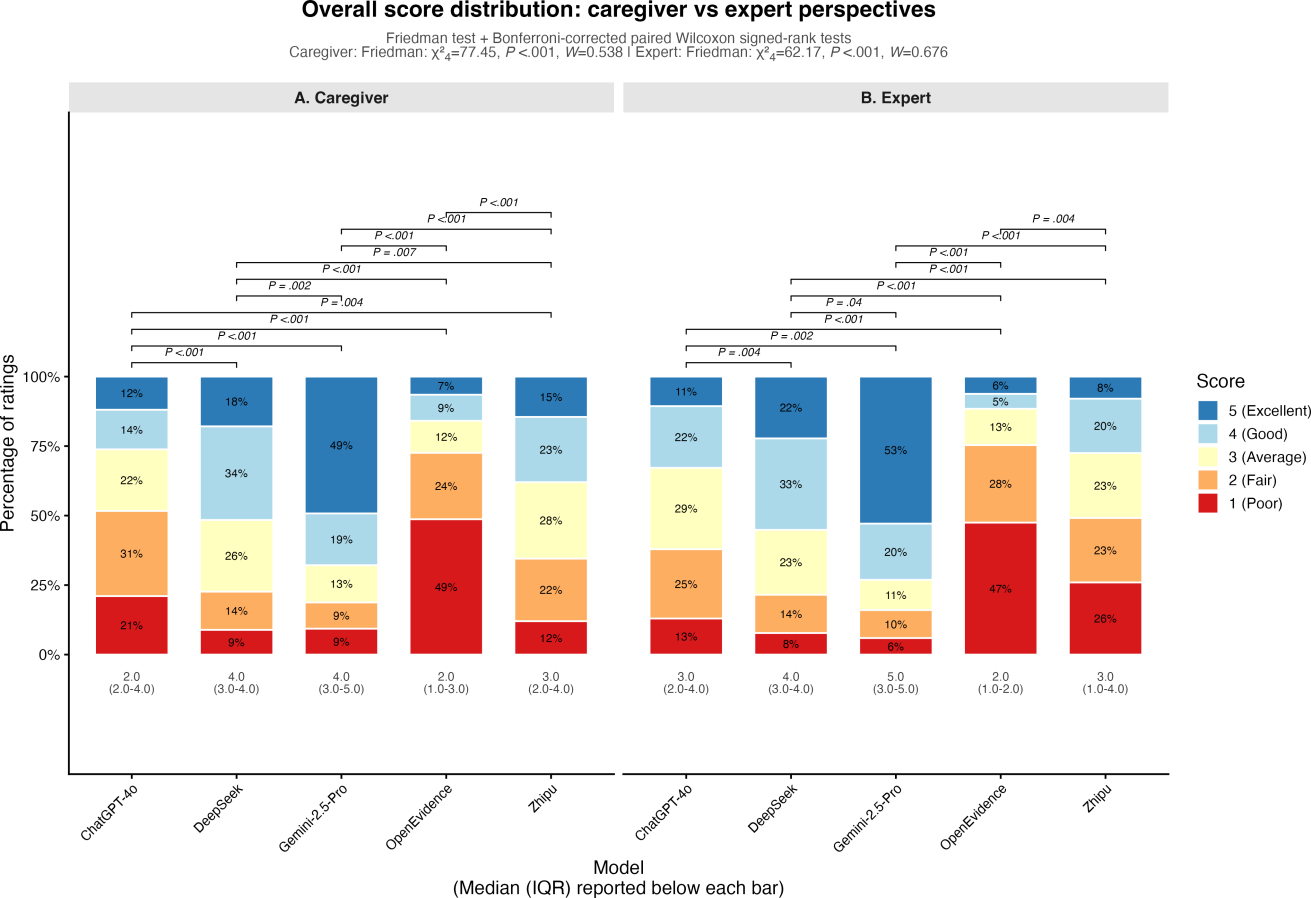
Distribution of comprehensive scores for response quality of 5 large language models from expert and caregiver perspectives. (A) Expert ratings (n=1380 evaluations per model from 23 experts across 10 questions and 6 dimensions). (B) Caregiver ratings (n=1440 evaluations per model from 36 caregivers across 10 questions and 4 dimensions). Each color band represents the proportion of a given score (1-5) within total evaluations.

In overall caregiver rankings (Figure 2A) and expert ratings (Figure 2B), Gemini-2.5-Pro ranked first in both groups (expert median 5.0, IQR 3.0‐5.0, 95% CI 5.0‐5.0; caregiver median 4.0, IQR 3.0‐5.0, 95% CI 4.0‐5.0). DeepSeek ranked second (expert median 4.0, IQR 3.0‐4.0, 95% CI 4.0‐4.0; caregiver median 4.0, IQR 3.0‐4.0, 95% CI 3.0‐4.0). Zhipu Qingyan ranked third (expert median 3.0, IQR 1.0‐4.0, 95% CI 2.0‐3.0; caregiver median 3.0, IQR 2.0‐4.0, 95% CI 3.0‐3.0). ChatGPT-4o was in the middle-to-lower range (expert median 3.0, IQR 2.0‐4.0, 95% CI 3.0‐3.0; caregiver median 2.0, IQR 2.0‐4.0, 95% CI 2.0‐3.0). OpenEvidence received the lowest scores, with expert median 2.0, IQR 1.0‐2.0, 95% CI 1.0‐2.0, and caregiver median 2.0, IQR 1.0‐3.0, 95% CI 1.0‐2.0.

**Figure 3.**
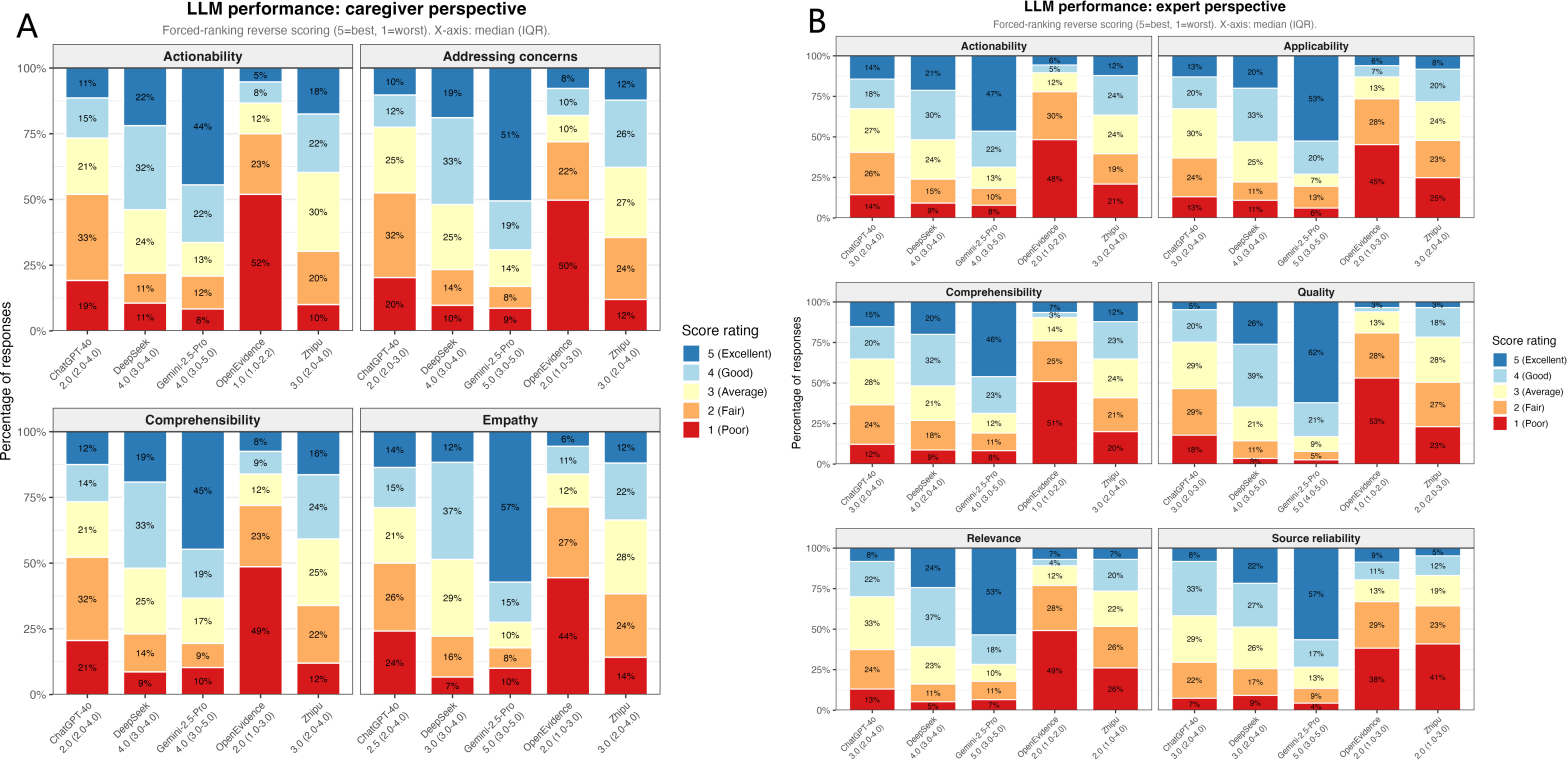
Score distribution across evaluation dimensions for 5 large language models from experts and caregivers. (A) Caregiver ratings across 4 dimensions (Empathy, Addressing Concerns, Comprehensibility, and Actionability). (B) Expert ratings across 6 dimensions (Quality, Relevance, Applicability, Source Reliability, Comprehensibility, and Actionability). Scoring followed the same forced-ranking reverse-scoring method as in Figure 2 (5=best, 1=worst). Each color band represents the proportion of a given score within total evaluations per dimension. Friedman significance per dimension is annotated; full Bonferroni-corrected pairwise comparisons are shown in Multimedia Appendix 7. LLM: large language model.

Multidimensional analysis (Figure 3A, caregiver perspective; and Figure 3B, expert perspective) revealed that Gemini-2.5-Pro scored consistently high across medical Quality (median 5.0, IQR 4.0‐5.0; 95% CI 5.0‐5.0), Applicability (median 5.0, IQR 3.0‐5.0; 95% CI 4.0‐5.0), Source Reliability (median 5.0, IQR 3.0‐5.0; 95% CI 4.0‐5.0), and Empathy dimensions (median 5.0, IQR 3.0‐5.0; 95% CI 5.0‐5.0). DeepSeek maintained stable scores in Quality (median 4.0, IQR 3.0‐5.0; 95% CI 4.0‐4.0), Actionability (expert median 4.0, IQR 3.0‐4.0, 95% CI 3.0‐4.0; caregiver median 4.0, IQR 3.0‐4.0, 95% CI 3.0‐4.0), and Comprehensibility (expert median 4.0, IQR 2.0‐4.0, 95% CI 3.0‐4.0; caregiver median 4.0, IQR 3.0‐4.0, 95% CI 3.0‐4.0). ChatGPT-4o and Zhipu Qingyan scored at an intermediate level across several dimensions, although caregivers rated ChatGPT-4o lower in Addressing Concerns (median 2.0, IQR 2.0‐3.0; 95% CI 2.0‐3.0) and Actionability (median 2.0, IQR 2.0‐4.0; 95% CI 2.0‐3.0). OpenEvidence received consistently low scores in Applicability (median 2.0, IQR 1.0‐3.0; 95% CI 1.0‐2.0), Source Reliability (median 2.0, IQR 1.0‐3.0; 95% CI 2.0‐2.0), and overall Comprehensibility (expert median 1.0, IQR 1.0‐2.0; 95% CI 1.0‐2.0).

### Model Performance Across Clinical Scenarios and Population Characteristics

#### Clinical Consultation Theme Stratification

A complementary thematic stratification (group A: preoperative and risk-prognosis consultation; group B: postoperative home care guidance; group C: emergency response) is reported in Multimedia Appendix 8. The principal pattern is preserved across the 3 themes: Gemini-2.5-Pro dominant, DeepSeek second, and ChatGPT-4o and Zhipu Qingyan intermediate; OpenEvidence lowest.

#### Risk-Level Stratification

Questions were classified into 3 clinical risk levels based on the potential patient safety consequences of incorrect AI-generated information: high risk (Q5, Q6, and Q9), medium risk (Q2, Q7, and Q8), and low risk (Q1, Q3, Q4, and Q10). The Friedman test revealed significant differences among the 5 models at all risk levels (all *P*<.001).

**Figure 4.**
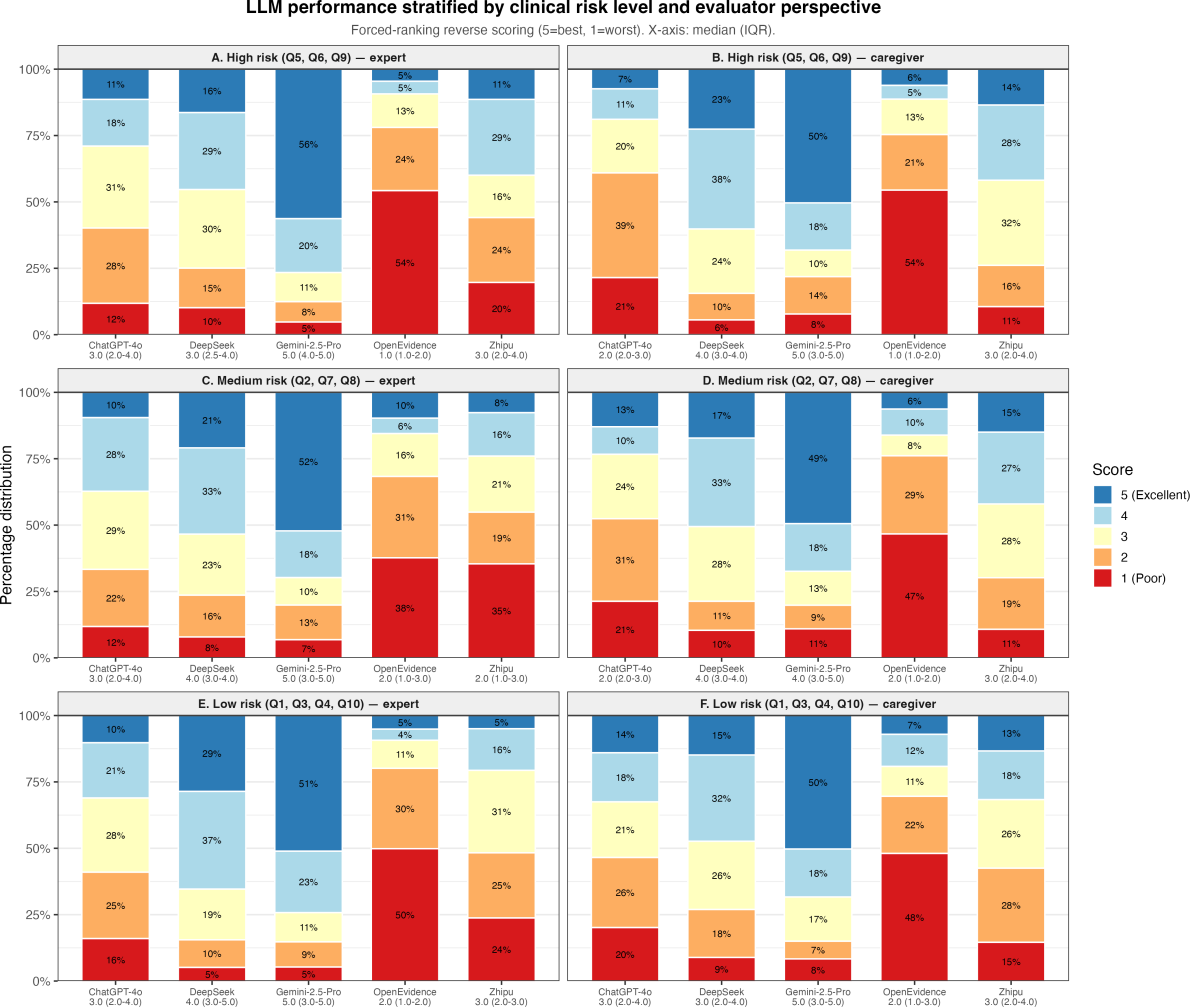
Risk-level stratified analysis of 5 large language models’ response quality, separated by evaluator perspective. Questions were independently classified by 2 research team members into 3 clinical risk levels based on potential patient safety consequences of incorrect information: high risk—direct potential harm (Q5: postoperative complications, Q6: complication prevention, and Q9: emergency response to postoperative dysuria), medium risk—potential for inappropriate clinical decisions (Q2: long-term outcome, Q7: reproductive or urinary function, and Q8: normal urination assessment), and low risk—unlikely to cause direct harm (Q1: success rate, Q3: anesthesia effects, Q4: recovery timeline, and Q10: follow-up schedule). The figure is faceted by perspective (Expert and Caregiver) and within each panel by risk level (High/Medium/Low). Overall, 100% stacked bars display the distribution of scores 1‐5 for each model under each risk level. Bonferroni-adjusted pairwise comparisons within each cell are reported in Multimedia Appendix 9. (A) High risk, expert perspective; (B) high risk, caregiver perspective; (C) medium risk, expert perspective; (D) medium risk, caregiver perspective; (E) low risk, expert perspective; and (F) low risk, caregiver perspective.

In the caregiver perspective, high-risk questions (Figure 4B) showed the most pronounced model differences: Gemini-2.5-Pro (median 5.0, IQR 3.0‐5.0; 95% CI 4.0‐5.0) and DeepSeek (median 4.0, IQR 3.0‐4.0; 95% CI 4.0‐4.0) maintained top-tier performance, while OpenEvidence dropped to median 1.0 (IQR 1.0‐2.0) (95% CI 1.0‐1.0). ChatGPT-4o received a low median of 2.0 (IQR 2.0‐3.0) (95% CI 2.0‐2.0) in high-risk caregiver questions, significantly lower than DeepSeek (*r*=−0.648; *P*<.001) and Gemini-2.5-Pro (*r*=−0.639; *P*<.001).

In the expert perspective, high-risk questions (Figure 4A) revealed that Gemini-2.5-Pro (median 5.0, IQR 4.0‐5.0; 95% CI 5.0-5.0) remained the highest-scoring model, while DeepSeek and ChatGPT-4o converged at median 3.0 (IQR 2.5-4.0, 95% CI 3.0-3.0; and IQR 2.0-4.0, 95% CI 3.0-3.0, respectively). OpenEvidence scored lowest (median 1.0, IQR 1.0‐2.0; 95% CI 1.0‐2.0). Notably, the absolute difference in median scores between the highest- and lowest-scoring models increased in the high-risk category (median difference: 4.0; Figure 4A and B) compared with the low-risk category (median difference: 3.0; Figure 4E and F) . See Figure 4A-F and Multimedia Appendix 9 for complete risk-level pairwise comparisons.

#### Heterogeneity Effects of Population Characteristics

Stratified analysis examined the influence of socioeconomic characteristics, disease subtype, and expert background on model scoring. Across all 8 stratifications, the principal ranking (Gemini-2.5-Pro>DeepSeek>Zhipu Qingyan≥ChatGPT-4o>OpenEvidence) proved consistent. On the caregiver side (N=36), no significant differences were observed across education levels (all Kruskal-Wallis *P*>.14; all *P* trend >.15) or employment status (all *P*>.06); for family income, the Kruskal-Wallis tests were nonsignificant for all models (all *P*>.05), but the Jonckheere-Terpstra trend tests revealed that OpenEvidence’s ratings declined monotonically with increasing income (*P* trend=.033), while Gemini-2.5-Pro showed a marginal rising trend (*P* trend=.094). For disease type, DeepSeek received significantly lower ratings in Proximal or Severe (types III and IV) cases than in distal or midshaft (types I and II) cases (median 3 vs 4; *r*=0.336, *P*=.046). On the expert side (N=23), professional seniority modulated discrimination most strongly: Gemini-2.5-Pro was rated monotonically higher with increasing seniority (Junior median 4/Intermediate median 5/Senior median 5; Kruskal-Wallis *P*=.004; *P* trend <.001), OpenEvidence monotonically lower (Junior median 2/Intermediate median 2/Senior median 1; Kruskal-Wallis *P*=.026; *P* trend=.007), and ChatGPT-4o showed a significant trend across seniority (*P* trend=.04) despite a nonsignificant omnibus test. Concordant rising trends were observed for expert age (Gemini-2.5-Pro *P* trend=.024) and clinical experience (Gemini-2.5-Pro *P* trend=.04). Complete stratified analysis tables with descriptive statistics, effect sizes, and 95% CIs are provided in Multimedia Appendix 10.

#### Agreement and Discrepancies Between Experts and Caregivers

Spearman correlation analysis revealed a strong positive correlation between the overall rankings of the 5 models by both evaluator groups (Spearman ρ=0.89, 95% CI 0.41‐1.00; *P*=.04, bootstrap with 2000 resamples). Major discrepancies centered on ChatGPT-4o: the expert group rated its medical Quality at a moderate level (median 3.0, IQR 2.0‐3.0; 95% CI 2.0‐3.0), while caregivers scored significantly lower on the Empathy (median 2.5, IQR 2.0‐4.0; 95% CI 2.0‐3.0) and Addressing Concerns (median 2.0, IQR 2.0‐2.0; 95% CI 2.0‐3.0) dimensions. The caregiver-expert divergence pattern was dimension-specific: expert Source Reliability ratings showed no significant difference between ChatGPT-4o and DeepSeek (*r*=−0.127; *P* adjusted=.88), yet the 2 models differed substantially on caregiver-evaluated Empathy (*r*=−0.343; *P*<.001). Regarding Comprehensibility, DeepSeek and Zhipu Qingyan were well received by caregivers (median 4.0 and 3.0, respectively); in contrast, the specialized medical model OpenEvidence struggled with readability, receiving the lowest Comprehensibility scores from both caregivers (median 2.0) and experts (median 1.0). This strong but imperfect agreement supports the dual-perspective design: the 2 groups converge on overall ranking while diverging on dimension-level priorities.

Valid qualitative responses were collected from caregivers in cohort B who completed the ranking and provided optional open-ended feedback. Content analysis revealed that caregivers cited “level of detail” (n=7) and “comprehensibility” (n=5) as their most frequent ranking criteria, followed by “comprehensiveness” (n=4), “clarity of expression” (n=3), and “overall impression” (n=3). Less frequently mentioned factors included “personal preference,” “patient-centeredness,” and “intuitiveness of presentation” (each n=1). These findings align with the quantitative results, where models scoring higher in structured presentation and readability received more favorable rankings.

#### Accuracy of Reference Citations

Descriptive analysis of the dual-reviewer verification revealed stark contrasts in how the models handled citations (Table 2). When resolving discrepancies, the V/PV/F/G/NR scheme was applied conservatively: unretrievable articles were strictly coded as Fabricated (F), and generic guideline mentions lacking specific bibliographic data were classified as Guideline-Based (G).

**Table 2.** Reference-authenticity classification by model^a^.

Model	Citations, n	V^b^, n (%)	PV^c^, n (%)	F^d^, n (%)	G^e^, n (%)	NR^f^, n (%)
OpenEvidence	47	47(100)	0 (0)	0 (0)	0 (0)	0 (0)
Gemini-2.5-Pro	40	3(8)	2 (5)	0 (0)	34 (85)	1 (3)
DeepSeek	39	4 (10)	2 (5)	13 (33)	20 (51)	0 (0)
ChatGPT-4o	33	5 (15)	2 (6)	5 (15)	21 (64)	0 (0)
Zhipu Qingyan	17	0 (0)	1 (6)	4 (24)	10 (59)	2 (12)

a Two independent reviewers classified every citation; preadjudication interrater agreement was substantial (raw agreement 80.9%; Cohen κ=0.702 across 157 paired records). Discrepancies were resolved by reapplying the V/PV/F/G/NR framework against canonical sources (PubMed → CrossRef DOI resolver → Google Scholar → CNKI); the consensus classification is reported here. Full per-citation classifications are shown in Multimedia Appendix 2.

b V: verifiable.

c PV: partially verifiable.

d F: fabricated.

e G: guideline-based, nonspecific.

f NR: no references.

Under these rigorous standards, OpenEvidence emerged as the only model to provide exclusively verifiable citations (*V*=100%, *F*=0%). Gemini-2.5-Pro similarly avoided fabrication (*F*=0%) but instead defaulted heavily to nonspecific guideline referencing (*G*=85%). ChatGPT-4o displayed a more mixed, guideline-leaning profile. In contrast, DeepSeek produced the highest proportion of fabricated references (*F*=33%, representing 13 of the 22 total fabrications), followed by Zhipu Qingyan (*F*=24%). Combined, these 2 models were responsible for 77% (17/22) of all fabricated citations documented in this audit (Multimedia Appendix 2).

#### Clinical Safety Audit Findings

The clinical safety audit identified 78 safety flags across the 5 models, revealing stark disparities in clinical reliability (Table 3; Multimedia Appendix 3). All 50 blinded responses were independently evaluated by an 8-member panel. To capture both practical risk and formal compliance, 7 senior pediatric urologists assessed safety through the lens of advanced clinical expertise, while 1 intermediate-title clinician specifically audited the outputs against Chapter 3.7 (Hypospadias) of the updated EAU guidelines [22] (Multimedia Appendix 13).

Using our predefined severity scale (Severe=3, Moderate=2, Mild=1, and None=0), OpenEvidence registered the highest severity-weighted score (58) and generated the majority of Severe flags (5 of 9). By contrast, Gemini-2.5-Pro maintained the safest profile, accruing a weighted score of just 2 with zero Severe events. Crucially, these safety metrics must be contextualized within the comprehensiveness rankings. A model that generates evasive, low-information responses might naturally avoid safety triggers, yet ultimately provide little actionable guidance to caregivers.

Across the panel, reviewers issued 9 Severe-level flags tied to 5 unique question-model combinations. Importantly, interrater convergence was strong: 4 of these 5 critical errors were independently identified by multiple reviewers. Qualitative analysis of these flags exposed distinct clinical vulnerabilities among the models.

**Table 3.** Clinical safety audit summary^a^.

Model	Severe	Moderate	Mild	Total flags	Severity-weighted score
Zhipu Qingyan	2	9	9	20	33
Gemini-2.5-Pro	0	0	2	2	2
OpenEvidence	5	9	25	39	58
DeepSeek	0	4	6	10	14
ChatGPT-4o	2	3	2	7	14

a The audit was conducted by 8 independent reviewers evaluating 50 blinded responses (10 questions×5 models). The severity-weighted score is calculated using the following grading scheme: Severe=3, Moderate=2, Mild=1, and None (Safe)=0.

OpenEvidence alone accounted for the majority of Severe events (5 of 9), stemming from 3 distinct clinical misjudgments. First, it inappropriately recommended routine preoperative androgen stimulation for distal hypospadias (Q6). This violates the EAU 2025 guidelines (§3.7.5.2), which strictly reserve hormonal therapy for specific proximal cases or micropenis. Second, rather than correctly triaging postoperative dysuria as a potential surgical emergency—such as urinary retention or catheter obstruction—the model provided an abstract academic differential diagnosis (Q9). Finally, it dangerously understated the timeline for full clinical recovery (Q4). Reviewers also frequently flagged OpenEvidence for moderate errors and epidemiological misrepresentations. For instance, it framed clinic-based uroflowmetry as a home-monitoring task (Q8), exaggerated fistula rates up to 70% (Q5), and presented high reoperation rates (51.8%) from complex redo surgeries without necessary case-mix caveats (Q1).

Other models similarly generated clinically hazardous advice. ChatGPT-4o produced an internally contradictory response advising an excessively prolonged catheter indwelling time of approximately 24 weeks (Q4)—a stark deviation from EAU 2025 §3.7.5.8, which notes durations ranging only from zero days to a few weeks. Zhipu Qingyan’s 2 Severe flags resulted from recommending routine retrograde urethrography at 3 and 6 months postoperatively (Q10). This invasive, radiation-exposing study is inappropriate for routine pediatric surveillance and directly conflicts with the EAU recommendation for noninvasive uroflowmetry (§3.7.7.1). Conversely, Gemini-2.5-Pro generated no Severe events and produced zero fabricated citations (*F*=0%), although it relied heavily on nonspecific, Guideline-Based citations (G=85%).

Ultimately, this stark dissociation between citation authenticity and clinical safety stands as a principal finding of our study. The most bibliographically accurate model—OpenEvidence (V=100%)—paradoxically accumulated the highest clinical safety burden. In contrast, DeepSeek, which recorded the highest citation fabrication rate (*F*=33% and, alongside Zhipu Qingyan, accounted for 77% of all fake citations), generated zero Severe safety events. This provides direct evidence that high citation accuracy does not guarantee clinical safety; they are fundamentally dissociable dimensions that necessitate separate evaluation (for the complete EAU 2025–anchored clinical safety audit and specific chapter-section anchors, see Multimedia Appendices 3 and 13).

#### Poststratification-Weighted Sensitivity Analysis

To ensure that findings were not artifacts of convenience sampling, poststratification weighting was applied, adjusting for hypospadias severity, caregiver education, and family income based on published national pediatric distributions [30,31]. Weights were normalized to the sample size (N=36), and weighted means with design-based standard errors were computed using the *R* survey package. This sensitivity analysis yielded rank orders identical to the unweighted primary analysis (Spearman ρ=1.00) (Table 4):

Although confidence intervals between adjacent ranks showed limited overlap due to design-based variance inflation, separation widened substantially for nonadjacent pairs, providing strong evidence that the principal model rankings are robust and generalizable.

**Table 4.** Poststratification-weighted ranking of model performance in sensitivity analysis.

Rank	Model	Weighted mean	Design-based 95% CI
1	Gemini-2.5-Pro	3.75	3.39‐4.11
2	DeepSeek	3.43	3.18‐3.68
3	Zhipu Qingyan	3.07	2.87‐3.26
4	ChatGPT-4o	2.59	2.42‐2.76
5	OpenEvidence	2.16	1.90‐2.43

## Discussion

### Support of Original Hypotheses

This study evaluated 5 mainstream LLMs—Gemini-2.5-Pro, DeepSeek, ChatGPT-4o, Zhipu Qingyan, and OpenEvidence—for perioperative consultation in pediatric hypospadias. The first confirmatory hypothesis—that LLM performance would vary across clinical correctness and patient-centered communication, with no model proving uniformly superior—was supported. Gemini-2.5-Pro produced the most comprehensive answers in both expert and caregiver evaluations; DeepSeek ranked second, with caregivers giving particularly high marks on Empathy; and OpenEvidence finished last from both perspectives despite producing the highest verifiable citation rate. No model led on every dimension. The principal ranking remained stable across 8 sociodemographic strata, across the 3 clinical risk levels, and across the poststratification-weighted sensitivity analysis (Spearman ρ=1.00 between weighted and unweighted rankings), arguing against demographic or clinical confounding as drivers of the observed pattern.

The second confirmatory hypothesis—that stakeholder ratings, citation verifiability, and clinical safety are dissociable dimensions of model behavior—was also supported. The 3 pillars yielded inconsistent rankings of the same models. OpenEvidence had the highest citation accuracy (*V*=100%) and yet accumulated 5 of 9 (56%) panel-wide Severe events, showing that accurate citations do not, on their own, guarantee clinically safe content; whether they are necessary cannot be answered from cross-sectional data of this design. DeepSeek illustrated the inverse: a citation fabrication rate of 33% co-occurred with zero Severe events. Gemini-2.5-Pro, also free of fabricated citations (*F*=0%), likewise recorded none. Fabricated citations and unsafe clinical content therefore appear to be distinct failure modes that require different mitigation strategies.

The 3 exploratory aims, reported with caution given uncontrolled family-wise error rates, each yielded a substantive finding. When caregivers were given unrestricted choice, they disproportionately selected higher-risk clinical questions: High- and medium-risk items accounted for 60% of the final top 10 despite contributing only 20% of the original pool, suggesting that caregivers naturally weight high-stakes clinical information above routine logistical queries, consistent with the heightened concern documented among caregivers of children with serious conditions [5], as well as their active information-seeking behaviors [6] and raising the bar for accuracy and safety in any AI model used in real-world patient education. Subgroup analysis showed a monotonic decline in OpenEvidence ratings as caregiver income rose (*P* trend =.033), with a marginal trend in the opposite direction for Gemini-2.5-Pro; higher-income caregivers, who may have greater health literacy [33,34] or stronger expectations about delivery style, appear less tolerant of OpenEvidence’s academic phrasing. On the Empathy dimension, caregivers rated DeepSeek and Zhipu Qingyan higher than ChatGPT-4o, although the underlying mechanisms—linguistic familiarity, cultural framing, or affective resonance—were not measured directly here and remain hypotheses for future work.

### Similarity of Results

Most prior work on medical AI has focused on model accuracy and treated accuracy as a proxy for usefulness [10]. Our sociodemographic data complicate that picture. The monotonic decline in OpenEvidence ratings with rising income, alongside the marginal rising trend for Gemini-2.5-Pro in the same group, mirrors earlier observations [10,11] that specialized information presented without lay-audience adaptation can hinder patient decision-making [35]. The caregiver qualitative comments point in the same direction: level of detail and comprehensibility were the 2 most frequent reasons for ranking. Deploying AI tools without sufficient attention to comprehensibility risks widening, rather than narrowing, disparities in access to understandable medical information [33,34,36]—a health-equity issue that must be explicitly addressed in all AI deployment plans.

On the affective dimensions, the locally adapted models DeepSeek and Zhipu Qingyan were rated higher on Empathy by caregivers than ChatGPT-4o. This is consistent with Masoud et al [37], who showed that language models tuned to specific linguistic and cultural contexts more closely reflect local social norms. ChatGPT-4o’s pattern was characteristic: experts gave it moderate Quality scores, but caregivers rated it poorly on Empathy and Addressing Concerns. Mesko and Topol [38] have argued that the strict safety alignment training of general-purpose models can push them toward formulaic disclaimers that read as cold; our caregiver ratings are compatible with that account. The trade-off is real, however: DeepSeek’s higher Empathy was paired with the highest citation-fabrication rate; so the Chinese-adapted models in our panel have not yet reconciled empathy with citation reliability.

The dissociation between citation accuracy and clinical safety extends earlier concerns. As demonstrated in health information overload research, highly technical evidence presented without clinical context can function as “information noise” that impairs patient comprehension, and Athaluri et al [12] and Alkaissi and McFarlane [13] documented the misinformation risk posed by fabricated citations that are superficially credible. Our V/PV/F/G/NR consensus classification adds resolution: DeepSeek and Zhipu Qingyan together account for the majority of fabricated citations, while Gemini-2.5-Pro’s guideline-leaning strategy prevents fabrication at the cost of bibliographic granularity. Within the disease-severity dimension, DeepSeek’s statistically significant performance drop in Proximal or Severe (types III and IV) cases (*r*=0.336, moderate effect) suggests that probabilistic models still have trouble matching the case-specific judgment human experts apply in complex hypospadias surgery—consistent with Topol’s [39] view (and GPT-4 analysis by Lee et al [40]) that human and machine intelligence work best in combination, not substitution. A practical translation is the tiered human-machine workflow proposed by Liyanage et al [41] and Krajcer [42]: validated models handle low-risk educational queries, while severe deformities, complication management, and complex surgical planning stay with human specialists. This is in line with LLM evaluation work in urolithiasis [43] and cornea care [44], where comprehensibility and triage gaps were similarly identified.

### Interpretation

Several sources of bias and threats to internal validity should be kept in mind. To counter the central tendency bias inherent in traditional absolute rating scales [45], we used a double-blind forced-ranking design with per-question randomized labels. This design forced evaluators to make distinct comparative judgments, effectively curbing “satisficing”—the tendency to default to neutral scores when cognitive demands are high [46]. As a result, the internal validity of our between-model comparisons was strengthened. Sampling was nevertheless by convenience at a single tertiary center in Chengdu, China, and the expert and caregiver groups likely underrepresent primary care, rural, and other cultural settings; with mothers making up 28 of 36 caregivers (77.8%), the Empathy and communication-quality findings cannot be assumed to extend to fathers or to other family structures.

Evaluator seniority represents a key potential source of assessment bias. Stratified analysis revealed that senior specialists rated Gemini-2.5-Pro monotonically higher and OpenEvidence monotonically lower as seniority increased, likely reflecting a higher sensitivity to citation-fabrication risks or stricter clinical standards. This aligns with recent evidence from Faraj et al [15], who found that senior, certified pediatric urologists exhibit significantly sharper clinical reasoning in hypospadiology assessments than junior peers. Consequently, accounting for evaluator seniority is essential, as long-term clinical maturity dictates a more rigorous, risk-aware standard when auditing AI-generated counseling content.

Regarding imprecision of measurement protocols, constructs such as eHealth literacy, cultural identity, linguistic preference, and state anxiety were not measured using validated psychometric instruments (eg, the eHealth Literacy Scale [eHEALS] [36] and the State-Trait Anxiety Inventory [STAI] [47]).

The overall number of tests and overlap among tests merit consideration. The principal ranking proved robust across 8 sociodemographic strata, 3 clinical risk levels, and poststratification weighting, providing strong evidence that the findings are not artifacts of multiple testing. However, subgroup analyses and exploratory free-choice question findings should be interpreted as hypothesis-generating rather than confirmatory. The adequacy of sample sizes and sampling validity is supported by the same robustness checks (ie, stratification and weighting), but the single-site, single-disease design still limits generalizability beyond this cohort.

### Generalizability

Several contextual factors limit how far these results travel. All 5 models were tested at one moment in time (April 6, 2025) on free-tier web interfaces with no exposed version identifiers, so the ranking values are explicitly time-anchored [21,48]; the evaluation method itself, and the clinician- and caregiver-prioritized dimensions identified here, should remain useful for benchmarking later model versions.

The clinical scope is also narrow. We tested perioperative consultations for a single pediatric malformation, so the model-specific conclusions need replication before they are applied to other settings; the evaluation structure (forced-ranking + citation audit + EAU-anchored safety audit) can be reused across other pediatric or adult specialties, as analogous multidimensional expert evaluations have proven viable in both adult urology and cross-disciplinary domains [43,44]. However, the specific model rankings cannot.

Finally, the 8-reviewer safety audit assessed static written outputs, not real-time clinical interactions. A clinical deployment would need prospective patient outcome monitoring, formal red-flag detection, and institutional safety review [38,49], together with cross-cultural validation [50] to test whether the dimension priorities (technical accuracy vs empathy) and the caregiver expert divergence pattern hold elsewhere. The evaluation framework, anchored by the V/PV/F/G/NR 5-category classification with dual-reviewer adjudication (Cohen κ=0.702, substantial agreement) and canonical-source reverification of all 30 disagreements, is portable across clinical domains; the specific results are not.

### Implications

These implications rest on several methodological strengths: a dual-perspective design capturing complementary clinician- and caregiver-rated dimensions; double-blind forced-ranking with randomized labels to mitigate central-tendency bias [45,46]; a 5-category consensus citation classification (V/PV/F/G/NR) with dual-reviewer adjudication (Cohen κ=0.702) and canonical-source reverification; an 8-reviewer EAU 2025–anchored clinical safety audit; robust cross-stratum ranking stability confirmed across sociodemographic subgroups, clinical risk tiers, and poststratification weighting (Spearman ρ=1.00); and a time-stamped, configuration-specific protocol (Multimedia Appendix 1) that bounds temporal validity and establishes a reproducible, unoptimized baseline.

This unoptimized baseline provides a systematic starting point for testing future prompt-engineering and retrieval-augmented interventions linked to the specific deficits we identified. Negative-constraint prompting—instructing models to cite only verifiable literature or to declare “insufficient evidence”—could test whether citation fabrication rates can be suppressed without degrading response quality [12,13]. Few-shot prompting [51] with standardized exemplar physician responses could target specific dimension-level deficits, such as ChatGPT-4o’s low Empathy scores or OpenEvidence’s poor Comprehensibility, provided these trials are conducted prospectively with concurrent evaluator recruitment to avoid recall bias. Retrieval-augmented generation (RAG) grounded in authoritative clinical resources (eg, EAU/AUA guidelines) is the most clinically actionable next step [52]; the specific errors flagged in our safety audit—off-indication testosterone recommendations, erroneous recovery timelines, and missing emergency-triage instructions—are exactly the failure modes RAG architectures are designed to prevent. Future prospective studies evaluating these technical optimizations should also incorporate validated psychometric instruments, such as the eHEALS [36] and the STAI [47], at the time of evaluation to enable direct correlation of these psychological constructs with dimension-level model ratings.

### Conclusions

No single LLM was uniformly best for perioperative consultation in pediatric hypospadias. Beyond the specific rankings, which will date as models update, this study contributes 2 findings that should remain useful across model generations: a characterization of which evaluation dimensions clinicians and caregivers each prioritize, and direct evidence that bibliographic accuracy and clinical content safety are dissociable dimensions and need to be assessed separately. The accompanying evaluation framework—dual-perspective forced-ranking, multidimensional rubric, independent citation verification, 8-reviewer guideline-anchored safety audit, and risk-stratified analysis—provides a reproducible baseline against which future prompt-engineering and retrieval-augmented interventions can be tested. For perioperative uses, AI should be deployed under a tiered human-machine collaboration model, with mandatory clinician oversight in high-risk scenarios [41,42].

## Supplementary material

10.2196/93393 Multimedia Appendix 1 Large language model access protocol.

10.2196/93393 Multimedia Appendix 2 Reference authenticity verification—summary findings.

10.2196/93393 Multimedia Appendix 3 Eight-reviewer clinical safety audit—per-reviewer findings.

10.2196/93393 Multimedia Appendix 4 Complete 40-question bank.

10.2196/93393 Multimedia Appendix 5 PlainText_LLM_Responses.

10.2196/93393 Multimedia Appendix 6 Evaluation dimension definitions and scoring procedure.

10.2196/93393 Multimedia Appendix 7 Complete pairwise comparison tables.

10.2196/93393 Multimedia Appendix 8 Theme-based analysis.

10.2196/93393 Multimedia Appendix 9 Risk-level stratified analysis.

10.2196/93393 Multimedia Appendix 10 Demographic subgroup stratified analysis.

10.2196/93393 Multimedia Appendix 11 Per-question expert ratings.

10.2196/93393 Multimedia Appendix 12 Per-question caregiver ratings.

10.2196/93393 Multimedia Appendix 13 European Association of Urology 2025 cross-check.

10.2196/93393 Checklist 1 CHART checklist.

## Abbreviations

AI: artificial intelligence
EAU: European Association of Urology
eHEALS: eHealth Literacy Scale
F: Fabricated (citation category)
G: Guideline-Based Non-Specific (citation category)
LLM: large language model
NR: No References (citation category)
PV: Partially Verifiable (citation category)
RAG: retrieval-augmented generation
STAI: State-Trait Anxiety Inventory
V: Verifiable (citation category)
