# Supplementary material for: Performance of 5 Large Language Models in Perioperative Consultation for Pediatric Hypospadias: Cross-Sectional Comparative Study
Source: J Med Internet Res. 2026 Jul 29;28:e93393. doi: 10.2196/93393 (PMC13419283; doi:10.2196/93393)
Supplement: Multimedia Appendix 1 [file jmir-v28-e93393-s001.docx]

**LLM access protocol**

Part A. Model access configuration.

| **Parameter** | **ChatGPT-4o** | **Gemini-2.5-Pro** | **OpenEvidence** | **DeepSeek** | **Zhipu Qingyan** |
| --- | --- | --- | --- | --- | --- |
| Access URL | chatgpt.com | gemini.google.com/app | openevidence.com | chat.deepseek.com | chatglm.cn |
| Model version (publicly disclosed) | GPT-4o (free tier; internal build not user-visible) | Gemini 2.5 Pro (free tier) | Not publicly disclosed | DeepSeek-V3 (free tier) | GLM-4 (free tier) |
| Open-source status | Closed-source | Closed-source | Closed-source | Open-source | Open-source |
| Web search / grounding | Disabled (free-tier default) | Disabled (Google Search grounding not activated) | Built-in medical-literature retrieval (default behaviour) | Disabled (default) | Disabled (default) |
| Plug-ins / tools | None enabled | None enabled | N/A | None enabled | None enabled |

Note: All models were accessed via their free tier. The input language was Simplified Chinese. Default temperature/sampling settings were used, and no response length constraints were applied.

Part B. Session management protocol.

| Step | Procedure |
| --- | --- |
| 1 | Open Google Chrome and clear cookies, cache, and browsing history. |
| 2 | Navigate to the model URL (Part A) and log in with a fresh or cleared session. |
| 3 | Open a new, independent conversation window for each of the 10 questions. |
| 4 | Paste the standardised prompt (Part C) verbatim. |
| 5 | Record the model’s complete response verbatim. The source request is embedded in each prompt; no separate follow-up is required. |
| 6 | Close the conversation window before submitting the next question, to reset session state and prevent contextual carry-over. |
| 7 | After all 10 questions are completed for a model, close the browser tab; clear cookies and cache; proceed to the next model from Step 1. |
| 8 | Convert all responses to plain text; remove embedded images, formatting elements, and hyperlinks. |

Part C. Complete prompt set. The parent-to-pediatric-urologist consultation template with an embedded source-request suffix was used for all 10 questions.

| Question | Prompt (Chinese, verbatim) | Prompt (English translation) |
| --- | --- | --- |
| Q1 | 如果你是一名小儿泌尿外科的医生，家长问到”尿道下裂手术成功率有多大？“问题，你应该怎么回答？并给出来源依据或参考出处。 | If you are a pediatric urologist, and a parent asks “What is the success rate of hypospadias surgery?”, how should you respond? Please provide sources/references. |
| Q2 | 如果你是一名小儿泌尿外科的医生，家长问到”尿道下裂手术后能保证长期的良好状态吗？“问题，你应该怎么回答？并给出来源依据或参考出处。 | If you are a pediatric urologist, and a parent asks “Can hypospadias surgery guarantee a long-term good outcome?”, how should you respond? Please provide sources/references. |
| Q3 | 如果你是一名小儿泌尿外科的医生，家长问到”全麻手术对患儿有哪些影响？“问题，你应该怎么回答？并给出来源依据或参考出处。 | If you are a pediatric urologist, and a parent asks “What are the effects of general anaesthesia on the child?”, how should you respond? Please provide sources/references. |
| Q4 | 如果你是一名小儿泌尿外科的医生，家长问到”尿道下裂患儿术后一般需要多长时间安全康复？“问题，你应该怎么回答？并给出来源依据或参考出处。 | If you are a pediatric urologist, and a parent asks “How long does it generally take for safe recovery after surgery?”, how should you respond? Please provide sources/references. |
| Q5 | 如果你是一名小儿泌尿外科的医生，家长问到”尿道下裂患儿手术有哪些潜在风险和并发症？“问题，你应该怎么回答？并给出来源依据或参考出处。 | If you are a pediatric urologist, and a parent asks “What are the potential risks and complications of hypospadias surgery?”, how should you respond? Please provide sources/references. |
| Q6 | 如果你是一名小儿泌尿外科的医生，家长问到”尿道下裂患儿如何预防术后并发症的发生？“问题，你应该怎么回答？并给出来源依据或参考出处。 | If you are a pediatric urologist, and a parent asks “How to prevent postoperative complications?”, how should you respond? Please provide sources/references. |
| Q7 | 如果你是一名小儿泌尿外科的医生，家长问到”尿道下裂手术对患儿未来的生殖功能和泌尿系统功能会有怎样的影响？“问题，你应该怎么回答？并给出来源依据或参考出处。 | If you are a pediatric urologist, and a parent asks “What is the long-term impact on reproductive and urinary function?”, how should you respond? Please provide sources/references. |
| Q8 | 如果你是一名小儿泌尿外科的医生，家长问到”尿道下裂患儿术后怎样判断排尿是否正常？“问题，你应该怎么回答？并给出来源依据或参考出处。 | If you are a pediatric urologist, and a parent asks “How to judge whether urination is normal after surgery?”, how should you respond? Please provide sources/references. |
| Q9 | 如果你是一名小儿泌尿外科的医生，家长问到”尿道下裂患儿术后若出现排尿困难或尿痛等情况，应该怎么办？“问题，你应该怎么回答？并给出来源依据或参考出处。 | If you are a pediatric urologist, and a parent asks “What should be done if urination difficulty or pain occurs after surgery?”, how should you respond? Please provide sources/references. |
| Q10 | 如果你是一名小儿泌尿外科的医生，家长问到”尿道下裂患儿术后需要多久进行一次复诊？“问题，你应该怎么回答？并给出来源依据或参考出处。 | If you are a pediatric urologist, and a parent asks “How often should follow-up visits be scheduled after surgery?”, how should you respond? Please provide sources/references. |

These results apply only to the free-tier web interfaces accessed on April 6, 2025. Model performance may change with future updates.
