# Supplementary material for: Performance of 5 Large Language Models in Perioperative Consultation for Pediatric Hypospadias: Cross-Sectional Comparative Study
Source: J Med Internet Res. 2026 Jul 29;28:e93393. doi: 10.2196/93393 (PMC13419283; doi:10.2196/93393)
Supplement: Multimedia Appendix 2 [file jmir-v28-e93393-s002.docx]

**Reference-authenticity verification—summary findings**

Two independent trained reviewers (Reviewer A and Reviewer B) classified every citation produced by the five LLMs using the V/PV/F/G/NR scheme (V = Verifiable; PV = Partially Verifiable; F = Fabricated; G = Guideline-Based, Non-Specific; NR = No References). Discrepancies were resolved by canonical-source re-verification (PubMed → CrossRef → Google Scholar → CNKI). This appendix summarises the verification outcomes in three views: (1) the question × model consensus distribution; (2) inter-rater agreement; and (3) the adjudication pattern for the 30 disagreement records.

**How to read the cells in Part A.** Each cell shows the per-category citation counts produced by that model in answering that question. For example, the Q1 × AI1 cell value “PV:1, F:1, G:2” indicates that AI1 produced four citations in answering Q1, of which the consensus classification placed 1 in the Partially Verifiable category, 1 in the Fabricated category, and 2 in the Guideline-Based Non-Specific category. Similarly, “G:3” in Q1 × AI2 means AI2 produced three citations all classified as G (named guidelines without specific bibliographic detail); “V:4” in Q1 × AI3 means AI3 produced four citations all of which were Verifiable in canonical databases. A dash indicates that no citation was produced for that question × model combination.

Part A. Consensus classification distribution by question and by AI label.

| Question | AI1 | AI2 | AI3 | AI4 | AI5 |
| --- | --- | --- | --- | --- | --- |
| Q1 | PV:1,F:1, G:2 | G:3 | V:4 | PV:1, F:1 | F:3, G:2 |
| Q2 | PV:1, G:3 | G:2 | V:1, F:1, G:1 | V:1, PV:1, F:1, G:1 | V:3 |
| Q3 | G:2 | V:2,PV:1, G:1 | V:4 | V:2, G:1 | V:3, G:2 |
| Q4 | G:3 | V:1, G:3 | F:1, G:2 | G:4 | V:3 |
| Q5 | V:1, F:1, G:2 | PV:1, F:3, G:1 | G:4, NR:1 | V:12 | G:2 |
| Q6 | PV:1, G:3 | G:3 | V:6 | F:2 | F:2, G:2 |
| Q7 | NR:1 | G:4 | V:1, F:1, G:1 | V:6 | F:2, G:2 |
| Q8 | V:3 | G:1 | G:3 | G:3 | G:3 |
| Q9 | V:3 | G:4 | NR:1 | F:1, G:3 | G:3 |
| Q10 | G:3 | G:4 | V:3 | F:1 | F:1, G:2 |

Part B. Inter-rater agreement (pre-adjudication, 157 paired records).

| Statistic | Value |
| --- | --- |
| Paired citation records | 157 |
| Records on which both reviewers agreed | 127 |
| Records on which reviewers disagreed | 30 |
| Raw agreement | 80.9% |
| Cohen’s κ | 0.702 (substantial) |

Part C. Disagreement-type distribution and adjudication outcomes.

| Disagreement type | n records | Adjudicated consensus (n records resolved to each category) |
| --- | --- | --- |
| G vs PV (boundary case) | 7 | G: 5; F: 1; PV: 1 |
| G vs V (boundary case) | 6 | G: 4; V: 2 |
| PV vs V | 6 | V: 4; G: 1; F: 1 |
| F vs PV | 5 | F: 5 |
| F vs G | 4 | F: 3; G: 1 |
| G vs NR | 2 | NR: 2 |

Aggregating across all 30 disagreements, the adjudicated consensus distribution was: F=12 (40%); G=9 (30%); V=6 (20%); NR = 2 (7%); PV = 1 (3%). The dominant adjudication pattern was conservative reclassification of guideline name-only citations to G and of unretrievable citations to F. Adjudication rationales were grounded in canonical-source re-verification against PubMed, CrossRef DOI resolver, Google Scholar, and CNKI.
