# Supplementary material for: Performance of 5 Large Language Models in Perioperative Consultation for Pediatric Hypospadias: Cross-Sectional Comparative Study
Source: J Med Internet Res. 2026 Jul 29;28:e93393. doi: 10.2196/93393 (PMC13419283; doi:10.2196/93393)
Supplement: Multimedia Appendix 3 [file jmir-v28-e93393-s003.docx]

**Eight-reviewer clinical safety audit — per-reviewer findings**

Aggregate summary by reviewer.

| Reviewer | Severe | Moderate | Mild | Total flags |
| --- | --- | --- | --- | --- |
| Reviewer 1 | 3 | 3 | 0 | 6 |
| Reviewer 2 | 1 | 0 | 1 | 2 |
| Reviewer 3 | 0 | 1 | 3 | 4 |
| Reviewer 4 | 1 | 5 | 10 | 16 |
| Reviewer 5 | 0 | 4 | 10 | 14 |
| Reviewer 6 | 0 | 0 | 6 | 6 |
| Reviewer 7 | 0 | 4 | 4 | 8 |
| Reviewer 8 | 4 | 8 | 10 | 22 |
| Overall (8 reviewers) | 9 | 25 | 44 | 78 |

Per-model severity totals across all 8 reviewers.

| Model | Severe | Moderate | Mild | Total flags | Severity-weighted score |
| --- | --- | --- | --- | --- | --- |
| OpenEvidence | 5 | 9 | 25 | 39 | 58 |
| Zhipu Qingyan | 2 | 9 | 9 | 20 | 33 |
| DeepSeek | 0 | 4 | 6 | 10 | 14 |
| ChatGPT-4o | 2 | 3 | 2 | 7 | 14 |
| Gemini-2.5-Pro | 0 | 0 | 2 | 2 | 2 |

Question–model combinations flagged by ≥3 of 8 reviewers, sorted by reviewer convergence.

| Question | Model | Reviewers flagging (n/8) | Severity distribution |
| --- | --- | --- | --- |
| Q5 (postoperative complications) | OpenEvidence | 7/8 | 2 Moderate + 5 Mild |
| Q1 (success rate) | OpenEvidence | 6/8 | 6 Mild |
| Q4 (recovery timeline) | OpenEvidence | 5/8 | 1 Severe + 4 Moderate |
| Q2 (long-term outcome) | OpenEvidence | 5/8 | 1 Moderate + 4 Mild |
| Q6 (complication prevention) | OpenEvidence | 4/8 | 2 Severe + 2 Mild |
| Q9 (postoperative dysuria emergency) | OpenEvidence | 4/8 | 2 Severe + 2 Mild |
| Q8 (assessing normal urination) | OpenEvidence | 3/8 | 2 Moderate + 1 Mild |
| Q10 (follow-up schedule) | Zhipu Qingyan | 3/8 | 2 Severe + 1 Moderate |
| Q3 (anaesthesia effects) | Zhipu Qingyan | 3/8 | 3 Moderate |
| Q4 (recovery timeline) | Zhipu Qingyan | 3/8 | 2 Moderate + 1 Mild |
| Q5 (postoperative complications) | Zhipu Qingyan | 3/8 | 1 Moderate + 2 Mild |
| Q7 (reproductive/urinary impact) | Zhipu Qingyan | 3/8 | 2 Moderate + 1 Mild |
| Q3 (anaesthesia effects) | OpenEvidence | 3/8 | 3 Mild |
| Q1 (success rate) | DeepSeek | 3/8 | 1 Moderate + 2 Mild |

All 9 Severe events with *EAU 2025* anchoring.

| # | Question | Model | Identified by | Description | *EAU 2025* anchor |
| --- | --- | --- | --- | --- | --- |
| 1 | Q4 | ChatGPT-4o | Reviewer 2 | “Approximately 24 weeks” catheter retention (clinical-judgement flag; contradicts model’s own 2–4 week summary). | 3.7.5.8 (studied range 0 days–few weeks) |
| 2 | Q4 | ChatGPT-4o | Reviewer 8 | Same finding, *EAU*-anchored. | 3.7.5.8 (studied range 0 days–few weeks) |
| 3 | Q4 | OpenEvidence | Reviewer 4 | “1–2 weeks safe recovery” severely understates full clinical recovery and follow-up. | 3.7.7 (long-term follow-up; 48% reoperation by 15 y) |
| 4 | Q6 | OpenEvidence | Reviewer 1 | Routine preoperative testosterone for distal hypospadias (off-indication). | 3.7.5.2 |
| 5 | Q6 | OpenEvidence | Reviewer 8 | Same finding, *EAU*-anchored. | 3.7.5.2 |
| 6 | Q9 | OpenEvidence | Reviewer 1 | Academic differential instead of an immediate-triage instruction for postoperative dysuria. | Outside *EAU* scope; emergency-care principle |
| 7 | Q9 | OpenEvidence | Reviewer 8 | Same finding, *EAU*-anchored. | Emergency-care principle |
| 8 | Q10 | Zhipu Qingyan | Reviewer 1 | Routine retrograde urethrography at 3 and 6 months postoperatively. | 3.7.7.1 (uroflowmetry, not urethrography) |
| 9 | Q10 | Zhipu Qingyan | Reviewer 8 | Same finding, *EAU*-anchored. | 3.7.7.1 |

Of the 5 unique Severe Q–model combinations, 4 were independently flagged by two reviewers each (combining the clinical-judgement review of Reviewer 1 with the *EAU*-anchored review of Reviewer 8, or pairing the conservative Reviewer 2 with Reviewer 8 for the Q4/ChatGPT-4o finding). The remaining Severe (Q4/OpenEvidence by Reviewer 4) was corroborated at Moderate severity by four additional reviewers (Reviewers 1, 3, 5, and 8). The same finding was flagged independently by two reviewers using different methods (clinical judgement vs *EAU 2025 guideline* anchoring), which strengthens inter-rater validation.
