# Supplementary material for: Performance of 5 Large Language Models in Perioperative Consultation for Pediatric Hypospadias: Cross-Sectional Comparative Study
Source: J Med Internet Res. 2026 Jul 29;28:e93393. doi: 10.2196/93393 (PMC13419283; doi:10.2196/93393)
Supplement: Multimedia Appendix 4 [file jmir-v28-e93393-s004.pdf]

Complete 40-question bank

Rank	Question ID	Question (English)	Question (Chinese)	Phase	Risk Level	n / 34	%	Top 10
1	Q13	How long for safe recovery after surgery?	尿道下裂患儿术后一般需要多长时间安全康复？	Postoperative Care	Low	24/34	70.6	✓
2	Q08	Can surgery guarantee long-term good outcomes?	尿道下裂手术后能保证长期的良好状态吗？	Postoperative Care	Medium	23/34	67.6	✓
3	Q18	Impact on future reproductive/ urinary function?	尿道下裂手术对患儿未来的生殖功能和泌尿系统功能会有怎样的影响？	Follow-up/ Prognosis	Medium	21/34	61.8	✓
4	Q16	What are potential risks and complications?	尿道下裂患儿手术有哪些潜在风险和并发症？	Other	High	17/34	50	✓
5	Q17	How to prevent postoperative complications?	尿道下裂患儿如何预防术后并发症的发生？	Postoperative Care	High	16/34	47.1	✓
6	Q38	How often for follow-up visits?	尿道下裂患儿术后需要多久进行一次复诊？	Postoperative Care	Low	16/34	47.1	✓
7	Q06	What is the surgical success rate?	尿道下裂手术成功率有多大？	Surgical/ Intraoperative	Low	13/34	38.2	✓

Rank	Question ID	Question (English)	Question (Chinese)	Phase	Risk Level	n / 34	%	Top 10
8	Q09	What are the effects of general anesthesia?	全麻手术对患儿有哪些影响？	Surgical/ Intraoperative	Low	13/34	38.2	✓
9	Q34	How to judge if urination is normal?	尿道下裂患儿术后怎样判断排尿是否正常？	Postoperative Care	Medium	13/34	38.2	✓
10	Q35	What to do if urination difficulty/pain occurs?	尿道下裂患儿术后若出现排尿困难或尿痛等情况，应该怎么办？	Postoperative Care	High	13/34	38.2	✓
11	Q14	Is physiotherapy needed for recovery?	尿道下裂术后是否需要理疗或康复治疗来帮助恢复？	Postoperative Care	Low	12/34	35.3	—
12	Q19	What to observe about the surgical wound?	尿道下裂患儿术后伤口需要观察哪些问题？	Postoperative Care	Low	12/34	35.3	—
13	Q21	How to avoid wound friction during home care?	尿道下裂患儿术后居家护理时，如何避免摩擦伤口？	Postoperative Care	Low	11/34	32.4	—
14	Q15	Will additional surgeries be needed?	尿道下裂患儿今后还需要进行其他手术吗？	Follow-up/ Prognosis	Medium	10/34	29.4	—
15	Q11	Does a pain pump affect the child?	镇痛泵对患儿是否	Surgical/ Intraoperative	Low	9/34	26.5	—

Rank	Question ID	Question (English)	Question (Chinese)	Phase	Risk Level	n / 34	%	Top 10
			有影响？					
16	Q26	What activity restrictions after surgery?	尿道下裂患儿手术后会有哪些活动限制？	Postoperative Care	Low	9/34	26.5	—
17	Q28	When can the child bathe after surgery?	尿道下裂患儿术后儿何时可以洗澡？	Postoperative Care	Low	9/34	26.5	—
18	Q23	Precautions for indwelling urinary catheter?	尿道下裂患儿术后留置尿管需要注意哪些问题？	Postoperative Care	Low	8/34	23.5	—
19	Q33	What to watch for during postoperative urination?	尿道下裂患儿术后排尿时需要注意什么？	Postoperative Care	Low	8/34	23.5	—
20	Q40	Impact of missing follow-up appointments?	尿道下裂患儿术后如果不能按时复诊，会对患儿的恢复产生什么影响？	Postoperative Care	Low	8/34	23.5	—
21	Q27	When can the child return to school?	尿道下裂患儿.手术后何时可以上学？	Postoperative Care	Low	7/34	20.6	—
22	Q05	What is the surgical procedure?	尿道下裂手术流程是什么？	Surgical/Intraoperative	Low	6/34	17.6	—
23	Q20	How to check for wound redness/bleeding/	尿道下裂患儿术后怎样观察	Postoperative Care	High	6/34	17.6	—

Rank	Question ID	Question (English)	Question (Chinese)	Phase	Risk Level	n / 34	%	Top 10
		discharge?	伤口是否有红肿、渗血、渗液等异常情况？					
24	Q01	What preoperative examinations are needed?	尿道下裂患儿术前需要做哪些检查？	Preoperative	Low	5/34	14.7	—
25	Q02	How should families prepare for surgery?	尿道下裂患儿术前家属需要如何为患儿的手术做准备？	Preoperative	Low	5/34	14.7	—
26	Q24	Dietary precautions after surgery?	尿道下裂患儿术后饮食方面需要注意哪些问题？	Postoperative Care	Low	5/34	14.7	—
27	Q31	Methods to relieve postoperative pain?	尿道下裂患儿术后有哪些方法可以缓解疼痛？	Postoperative Care	Low	5/34	14.7	—
28	Q39	What is checked during follow-up visits?	尿道下裂患儿术后复诊主要检查哪些项目？	Preoperative	Low	5/34	14.7	—
29	Q12	How long is the postoperative hospital stay?	尿道下裂患儿术后需要住院多长时间？	Postoperative Care	Low	4/34	11.8	—
30	Q29	Special clothing requirements after surgery?	尿道下裂患儿术后的衣物选择上有什	Postoperative Care	Low	4/34	11.8	—

Rank	Question ID	Question (English)	Question (Chinese)	Phase	Risk Level	n / 34	%	Top 10
			么特殊要求吗？					
31	Q03	What is the purpose of hypospadias surgery?	尿道下裂手术的目的是什么？	Other	Low	3/34	8.8	—
32	Q04	What is the surgical method?	尿道下裂手术方式是什么？	Surgical/ Intraoperative	Low	3/34	8.8	—
33	Q10	Is a pain pump necessary?	尿道下裂患儿是否必须安置镇痛泵？	Surgical/ Intraoperative	Low	3/34	8.8	—
34	Q22	What drainage tubes may be placed?	尿道下裂患儿术后可能有哪些管道？	Postoperative Care	Low	3/34	8.8	—
35	Q32	How to choose pain management strategies?	尿道下裂患儿术后疼痛时应该如何选择应对措施？	Postoperative Care	Low	3/34	8.8	—
36	Q07	How long does the surgery take?	尿道下裂手术时长有多久？	Surgical/ Intraoperative	Low	2/34	5.9	—
37	Q25	How to handle poor appetite after surgery?	尿道下裂患儿术后食欲不佳，如何应对？	Postoperative Care	Low	2/34	5.9	—
38	Q30	How long does postoperative pain last?	尿道下裂患儿术后疼痛一般会持续多久？	Postoperative Care	Low	2/34	5.9	—
39	Q36	How to handle child’s emotional distress?	尿道下裂患儿术后可能会因为疼痛、	Postoperative Care	Low	2/34	5.9	—

Rank	Question ID	Question (English)	Question (Chinese)	Phase	Risk Level	n / 34	%	Top 10
			不适等出现情绪问题，作为家属应如何应对？					
40	Q37	How can caregivers manage their own psychological stress?	家属自身在面对尿道下裂患儿手术和康复过程中，如何缓解自身的心理压力？	Postoperative Care	Low	2/34	5.9	—

Phase = perioperative phase classification (Preoperative; Surgical/Intraoperative; Postoperative Care; Follow-up/Prognosis; Other). Risk Level = clinical risk classification (High = potential for direct harm; Medium = potential for inappropriate clinical decisions; Low = unlikely to cause direct harm), independently assigned by two research team members based on the potential patient-safety consequences of incorrect AI-generated information. n / 34 = number of Cohort A caregivers who selected the question as a primary concern. The Top 10 (highest-frequency questions, denoted ✓) were carried forward to the LLM-response evaluation; they span all three clinical risk levels.

The selection frequencies colour-coded by risk level are shown as a horizontal bar chart below.

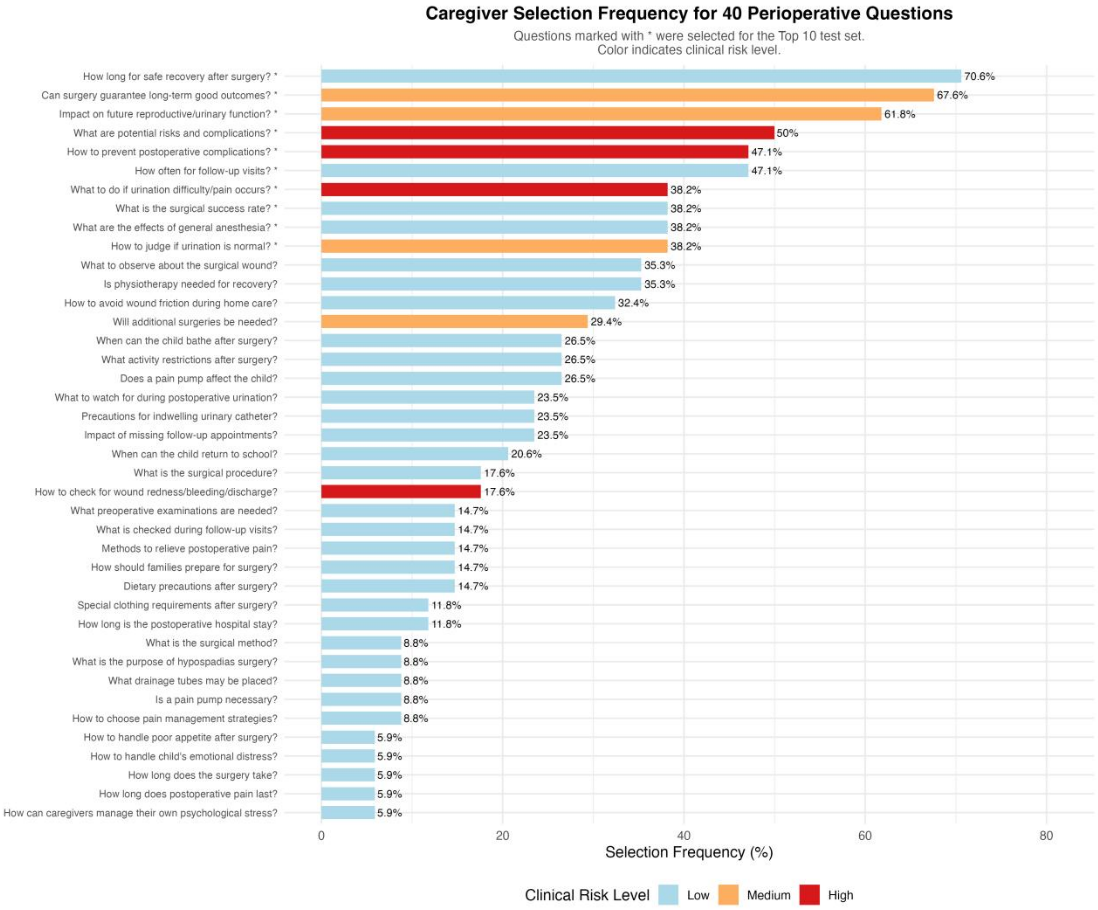
Caregiver Selection Frequency for 40 Perioperative Questions
Questions marked with * were selected for the Top 10 test set.
Color indicates clinical risk level.
How long for safe recovery after surgery? * 70.6%
Can surgery guarantee long-term good outcomes? * 67.6%
Impact on future reproductive/urinary function? * 61.8%
What are potential risks and complications? * 50%
How to prevent postoperative complications? * 47.1%
How often for follow-up visits? * 47.1%
What to do if urination difficulty/pain occurs? * 38.2%
What is the surgical success rate? * 38.2%
What are the effects of general anesthesia? * 38.2%
How to judge if urination is normal? * 38.2%
What to observe about the surgical wound? 35.3%
Is physiotherapy needed for recovery? 35.3%
How to avoid wound friction during home care? 32.4%
Will additional surgeries be needed? 29.4%
When can the child bathe after surgery? 26.5%
What activity restrictions after surgery? 26.5%
Does a pain pump affect the child? 26.5%
What to watch for during postoperative urination? 23.5%
Precautions for indwelling urinary catheter? 23.5%
Impact of missing follow-up appointments? 23.5%
When can the child return to school? 20.6%
What is the surgical procedure? 17.6%
How to check for wound redness/bleeding/discharge? 17.6%
What preoperative examinations are needed? 14.7%
What is checked during follow-up visits? 14.7%
Methods to relieve postoperative pain? 14.7%
How should families prepare for surgery? 14.7%
Dietary precautions after surgery? 14.7%
Special clothing requirements after surgery? 11.8%
How long is the postoperative hospital stay? 11.8%
What is the surgical method? 8.8%
What is the purpose of hypospadias surgery? 8.8%
What drainage tubes may be placed? 8.8%
Is a pain pump necessary? 8.8%
How to choose pain management strategies? 8.8%
How to handle poor appetite after surgery? 5.9%
How to handle child's emotional distress? 5.9%
How long does the surgery take? 5.9%
How long does postoperative pain last? 5.9%
How can caregivers manage their own psychological stress? 5.9%
0
20
40
60
80
Selection Frequency (%)
Clinical Risk Level
Low
Medium
High
