# Supplementary material for: Performance of 5 Large Language Models in Perioperative Consultation for Pediatric Hypospadias: Cross-Sectional Comparative Study
Source: J Med Internet Res. 2026 Jul 29;28:e93393. doi: 10.2196/93393 (PMC13419283; doi:10.2196/93393)
Supplement: Multimedia Appendix 5 [file jmir-v28-e93393-s005.pdf]

PlainText_LLM_Responses

Preamble

This appendix contains the verbatim plain-text responses produced by each of the five evaluated large language models — ChatGPT-4o, Gemini-2.5-Pro, OpenEvidence, Zhipu Qingyan, and DeepSeek — when queried with the 10 Top-10 perioperative-hypospadias questions identified by the Cohort A screening. Fifty responses are presented (5 models × 10 questions).

Setting and protocol. All responses were generated on April 6, 2025 in Google Chrome with cleared cookies/cache, in each model's publicly available free-tier web interface, without paid subscriptions, plug-ins, or API customisation. Each question was submitted as an independent single-turn query in a separate conversation window using a standardised parent-to-pediatric-urologist consultation template with an embedded source-request suffix. Responses were normalised to plain text — embedded images, hyperlinks, and markdown formatting were removed; self-referential boilerplate phrases (self-introductory framing, self-evaluative meta-commentary, and optional follow-up offers) were uniformly removed across all five models to preserve blinding; response length was not artificially constrained.

Blinded labelling. To prevent inferential leakage of model identity, model identities were coded AI1 through AI5 using a per-question randomised blinding scheme so that the AI label associated with a given model differs across questions. The mapping between AI label and model identity was held in the trial registry record and was not visible to evaluators while they completed the ranking task. The post-hoc unblinding key is presented at the head of each question section below.

Bilingual presentation. For each AI response, the original verbatim Chinese output is presented first (this is the authoritative version that evaluators saw), followed immediately by an English translation produced by automated machine translation (Google Translate, accessed via the deep-translator Python library). The English version is provided as a navigation aid for non-Chinese-reading readers; the Chinese original remains the authoritative scientific record.

Question 1 (Low risk; Surgical / Intraoperative)

Original prompt (Chinese):

如果你是一名小儿泌尿外科的医生，家长问到“尿道下裂手术成功率有多大？”问题，你应该怎么回答？并给出来源依据或参考出处。

Prompt translation (English):

If you are a pediatric urologist, and a parent asks, "What is the surgical success rate for hypospadias?", how should you answer? Please cite your sources or references.

Per-question unblinding key for Q1:

Blinded label	Model identity
AI1	ChatGPT-4o
AI2	Gemini-2.5-Pro
AI3	OpenEvidence
AI4	Zhipu Qingyan
AI5	DeepSeek

AI1 (post-hoc identity: ChatGPT-4o)

Chinese original (verbatim output):

临床沟通建议（作为小儿泌尿外科医生的回答示范）：

“尿道下裂的手术成功率，其实受很多因素影响，包括尿道下裂的类型（比如是远端型、近端型还是复杂型）、孩子的解剖条件、选择的手术方式，以及手术医生的经验。

对于大多数远端型尿道下裂，手术一次性成功、恢复良好的比例可以达到 85%-95%左右。近端型或者复杂尿道下裂，可能需要两次甚至多次手术，综合成功率相对低一些，可能在 70%-85%之间。

不同研究和不同中心的数据会略有差异，我们医院的情况大致在这个范围。总体来说，我们会根据孩子的具体情况，为家长制定最适合的手术方案，并尽可能提高手术的安全性和成功率。”

回答的证据来源或参考依据：

1. 国际指南与共识意见

- American Urological Association (AUA) Guidelines on Hypospadias (2014, update ongoing)

— 远端型尿道下裂一次性修复成功率可达 85-95%，近端型复杂尿道下裂可能需要 staged repair，复发率和并发症发生率较高。

- European Association of Urology (EAU) Guidelines on Paediatric Urology (最新版)

— 明确指出手术成功率依赖于尿道下裂的类型与复杂程度。远端型一次性成功率>85%，近端型低于此水平。

2. 经典文献数据

- Snodgrass W et al. (J Urol. 2020;203(4):746-752)

— 报告了 TIP（Snodgrass 术式）用于远端尿道下裂的长期随访结果，成功率约 90%。

- Long-term Outcomes of Hypospadias Surgery: A Systematic Review.

Springer A, J Pediatr Urol. 2014;10(1):S2-S9.

— 系统综述不同类型尿道下裂的手术成功率与并发症发生率。

小结

这种沟通方式的特点：

- 个体化 → 具体分析孩子的病情。
- 知情同意 → 提前说明成功率不是绝对的。
- 证据支持 → 背后有指南和文献数据支撑。
- 保持希望但不夸大 → 科学严谨，体现专业度和诚意。

English translation (machine translation; for navigation only):

Clinical Communication Suggestions (as a model answer for a pediatric urologist):

“The success rate of hypospadias surgery is actually affected by many factors, including the type of hypospadias (such as distal, proximal or complex), the child’s anatomical conditions, the selected surgical method, and the experience of the surgeon.

For most distal hypospadias, the rate of successful one-time surgery and good recovery can reach about 85%-95%. Proximal or complex hypospadias may require two or even multiple surgeries, and the overall success rate is relatively low, possibly between 70% and 85%.

The data of different studies and different centers will be slightly different, and the situation in our hospital is roughly within this range. Generally speaking, we will formulate the most suitable surgical plan for parents based on the child's specific situation, and try to improve the safety and success rate of the surgery as much as possible. "

Sources of evidence or references for your answer:

1. International guidelines and consensus opinions

- American Urological Association (AUA) Guidelines on Hypospadias (2014, update ongoing)

— The success rate of one-time repair of distal hypospadias can reach 85-95%, while complex proximal hypospadias may require staged repair, with higher recurrence and complication rates.

• European Association of Urology (EAU) Guidelines on Paediatric Urology (latest edition)

— It is clearly stated that the success rate of surgery depends on the type and complexity of hypospadias. The one-time success rate of the distal type is >85%, while the proximal type is lower than this level.

2. Classic literature data

- Snodgrass W et al. (J Urol. 2020;203(4):746-752)

— Reported the long-term follow-up results of TIP (Snodgrass procedure) for distal hypospadias, with a success rate of approximately 90%.

- Long-term Outcomes of Hypospadias Surgery: A Systematic Review.

Springer A, J Pediatr Urol. 2014;10(1):S2-S9.

— Systematic review of surgical success rates and complication rates for different types of hypospadias.

summary

Characteristics of this communication method:

• Individualization → Analyze the child's condition in detail.

• Informed consent → state upfront that success rates are not absolute.

• Supported by evidence → Backed by guidelines and literature data.

• Be hopeful without exaggerating → Be scientifically rigorous and reflect professionalism and sincerity.

AI2 (post-hoc identity: Gemini-2.5-Pro)

Chinese original (verbatim output):

好的，作为一名小儿泌尿外科医生，我会这样回答家长关于尿道下裂手术成功率的问题：

“您好！这是一个非常好的问题，也是很多家长都非常关心的。尿道下裂手术的目标是重建一个外观和功能都正常的阴茎，让孩子能够站立排尿，尿流向前，并且成年后有正常的勃起和性功能。

关于‘成功率’，这需要从几个方面来看：

1. 总体成功率： 总的来说，尿道下裂手术的成功率是比较高的，尤其对于轻中度的尿道下裂。根据国内外大量的临床研究和经验，一次手术的成功率通常可以达到 85% 到 95% 甚至更高。这意味着大多数孩子通过一次手术就能达到满意的效果。

2. 影响因素：但是，这个数字并不是绝对的，成功率会受到几个重要因素的影响：

  * 尿道下裂的严重程度： 这是最重要的因素。轻型（阴茎头型、冠状沟型）的尿道下裂，手术相对简单，成功率非常高。而重型（阴茎阴囊型、会阴型），手术难度大，需要更复杂的技术，并发症的风险相对会高一些，可能需要分期手术或再次修复的几率也更大。

  * 是否合并其他畸形： 比如严重的阴茎下弯，这会增加手术的复杂性。

  * 手术时机和孩子自身条件：孩子的年龄、阴茎发育情况、组织条件和愈合能力也会有一定影响。

  * 手术技术和医生经验： 选择合适的手术方式以及手术医生的经验和技术熟练程度至关重要。经验丰富的专科医生通常能获得更好的手术效果和更低的并发症率。

3. 成功的标准： 我们衡量‘成功’的标准通常包括：

  * 尿道开口位于阴茎头的正常位置或接近正常位置。

  * 阴茎外观良好，下弯得到完全矫正。

  * 排尿通畅，尿流呈直线向前。

  * 没有发生严重的并发症，如尿瘘（尿液从异常开口漏出）、尿道狭窄（排尿困难）、伤口裂开等。

4. 并发症的可能性： 需要了解的是，即使是经验丰富的医生，也存在一定比例的并发症风险，尤其对于复杂病例。常见的并发症包括尿瘘、尿道狭窄、伤口感染或裂开、阴茎下弯矫正不满意等。好消息是，大多数并发症是可以处理和修复的，尽管有时可能需要再次手术。

总结来说：对于大多数尿道下裂的孩子，手术效果是很好的，一次成功的几率很高。但对于复杂类型，确实挑战更大，可能需要更精心的手术设计和术后管理，甚至有再次手术的可能。

我们会在术前对您孩子的情况进行详细评估，根据尿道下裂的具体类型和严重程度，选择最适合的手术方案，并向您更具体地解释预期的成功率和可能的风险。请放心，我们会尽最大努力，争取一次手术就达到最佳效果。术后的精心护理也非常重要，我们会和您一起努力，确保孩子顺利康复。”

以上回答内容的来源依据或参考出处主要是：

1. 权威医学教科书： 如《坎贝尔-沃尔什泌尿外科学》（Campbell-Walsh Urology）、《小儿泌尿外科学》（Pediatric Urology）等国际权威教材中关于尿道下裂章节的论述。这些教科书总结了该领域的标准知识和公认的治疗原则。

2. 同行评审的医学文献：大量发表在国际和国内核心泌尿外科、小儿外科期刊（如 Journal of Urology, Journal of Pediatric Urology, European Urology, 中华泌尿外科杂志, 中华小儿外科杂志等）上的临床研究报告、回顾性分析、前瞻性研究以及 Meta 分析。这些研究提供了不同手术方式、不同严重程度尿道下裂手术成功率和并发症率的具体数据。

3. 临床指南和专家共识： 美国泌尿外科学会（AUA）、欧洲泌尿外科学会（EAU）等权威机构发布的相关疾病诊疗指南或专家共识，这些文件基于现有最佳证据，为临床实践提供指导。

4. 大型医学数据库和综述：如 PubMed, Cochrane Library 等数据库中的系统评价和综述文章，它们整合了多项研究的结果，提供了关于成功率和并发症风险的更全面的信息。

5. 临床经验和本中心数据： 作为医生，长期的临床实践、手术经验以及本医疗中心或团队内部对于过往病例的统计和总结也是回答该问题的实践基础。

综合以上来源的信息，可以为家长提供一个既符合医学事实，又相对容易理解的、关于尿道下裂手术成功率的全面回答。

English translation (machine translation; for navigation only):

OK, as a pediatric urologist, here’s how I would answer parents’ questions about the success rate of hypospadias surgery:

"Hello! This is a very good question and one that many parents are very concerned about. The goal of hypospadias surgery is to reconstruct a penis that looks and functions normally, so that the child can urinate standing up, with the urine flowing forward, and have normal erections and sexual function as an adult.

Regarding the ‘success rate’, this needs to be looked at from several aspects:

1. Overall success rate: Generally speaking, the success rate of hypospadias surgery is relatively high, especially for mild to moderate hypospadias. According to a large amount of clinical research and experience at home and abroad, the success rate of one operation can usually reach 85% to 95% or even higher. This means that most children can achieve satisfactory results with just one surgery.

2. Influencing factors: However, this number is not absolute. The success rate will be affected by several important factors:

* Severity of hypospadias: This is the most important factor. For mild hypospadias (glans type, coronal sulcus type), the operation is relatively simple and the success rate is very high. For severe cases (penoscrotal type, perineal type), the operation is difficult and requires more complex techniques. The risk of complications is relatively higher, and the chance of requiring staged surgery or re-repair is also greater.

* Whether it is combined with other deformities: such as severe penile curvature, which will increase the complexity of the surgery.

* Timing of surgery and the child's own conditions: The child's age, penis development, tissue conditions and healing ability will also have a certain impact.

* Surgical technique and surgeon experience: Choosing the appropriate surgical method and the experience and technical proficiency of the surgeon are crucial. Experienced specialists often achieve better surgical results and lower complication rates.

3. Criteria for success: The criteria by which we measure ‘success’ usually include:

* The urethral opening is at or near the normal position of the glans penis.

* The penis looks good and the downward curvature is completely corrected.

* Urination is smooth and the urine flow is straight forward.

* No serious complications occurred, such as urinary fistula (urine leaking from an abnormal opening), urethral stricture (difficulty urinating), wound dehiscence, etc.

4. Possibility of complications: It is important to understand that even experienced doctors have a certain risk of complications, especially in complex cases. Common complications include urinary fistula, urethral stricture, wound infection or dehiscence, and unsatisfactory penile curvature correction. The good news is that most complications can be treated and repaired, although sometimes additional surgery may be required.

In summary: For most children with hypospadias, the surgical effect is very good, and the chance of success is very high. However, for complex types, the challenges are indeed greater and may require more careful surgical design and postoperative management, and even the possibility of re-operation.

We will conduct a detailed preoperative assessment of your child's condition, select the most appropriate surgical option based on the specific type and severity of hypospadias, and explain to you in more detail the expected success rates and possible risks. Please rest assured that we will do our best to achieve the best results with just one operation. Careful post-operative care is also very important, and we will work with you to ensure your child recovers smoothly. "

The sources or references for the above answers are mainly:

1. Authoritative medical textbooks: Such as "Campbell-Walsh Urology" (Campbell-Walsh Urology), "Pediatric Urology" (Pediatric Urology) and other international authoritative textbooks discussing the chapters on hypospadias. These textbooks summarize standard knowledge and accepted treatment principles in the field.

2. Peer-reviewed medical literature: a large number of clinical research reports, retrospective analyses, prospective studies and meta-analyses published in core international and domestic urology and pediatric surgery journals (such as Journal of Urology, Journal of Pediatric Urology, European Urology, Chinese Journal of Urology, Chinese Journal of Pediatric Surgery, etc.). These studies provide specific data on the success and complication rates of hypospadias surgery based on different surgical approaches and severities of hypospadias.

3. Clinical guidelines and expert consensus: Related disease diagnosis and treatment guidelines or expert consensus issued by authoritative organizations such as the American Urological Association (AUA) and the European Association of Urology (EAU). These documents are based on the best available evidence and provide guidance for clinical practice.

4. Large medical databases and reviews: Systematic reviews and review articles in databases such as PubMed, Cochrane Library, etc., which integrate the results of multiple studies and provide more comprehensive information on success rates and risks of complications.

5. Clinical experience and data from this center: As a doctor, long-term clinical practice, surgical experience, and the statistics and summary of past cases within this medical center or team are also the practical basis for answering this question.

Combining the information from the above sources can provide parents with a comprehensive answer about the success rate of hypospadias surgery that is both consistent with medical facts and relatively easy to understand.

AI3 (post-hoc identity: OpenEvidence)

Chinese original (verbatim output):

Hypospadias 手术的成功率取决于多种因素，包括手术类型、患者的具体情况以及随访时间的长短。总体而言，手术的成功率较高，但并发症的发生率也不容忽视。

根据现有文献，远端型尿道下裂手术的并发症发生率约为 5%至 10%，而近端型尿道下裂手术的并发症发生率则较高，约为 12%至 23%。这些并发症包括尿瘘、尿道狭窄和伤口裂开等。[1]

长期随访研究表明，手术后的再手术率较高。例如，一项研究显示，手术后 5 年和 15 年的再手术率分别为 39.3%和 51.8%。另一项研究指出，24.1%的患者在手术后需要再次手术，其中 47.4%的再手术发生在手术后的第一年。[2-3]

此外，患者和家长对手术结果的满意度也较高。一项研究显示，83%的儿童和 87%的家长对手术后的外观结果感到满意，而功能性结果的满意度分别为 89%和 92%。[4]

综上所述，尿道下裂手术的成功率较高，但并发症和再手术的风险也需要考虑。长期随访对于评估手术的真实效果至关重要。

1.Discrepant Rates of Hypospadias Surgical Complications: A Comparison of U.S. News & World Report and Pediatric Health Information System® Data and Published Literature.Pohl HG, Rana S, Sprague BM, Beamer M, Rushton HG.The Journal of Urology. 2020;203(3):616-623. doi:10.1097/JU.0000000000000554.

2.Cumulative Re-Operation Rates During Follow-Up After Hypospadias Repair.Anttila A, Lahdes-Vasama T, Pakkasjärvi N, Taskinen S.BJU International. 2024;134(6):960-966. doi:10.1111/bju.16519. New Research

3.Hypospadias Repair at a Tertiary Care Center: Long-Term Followup Is Mandatory to Determine the Real Complication Rate.Spinoit AF, Poelaert F, Groen LA, Van Laecke E, Hoebeke P.The Journal of Urology. 2013;189(6):2276-81. doi:10.1016/j.juro.2012.12.100.

4.Hypospadias: Clinical Approach, Surgical Technique and Long-Term Outcome.Ceccarelli PL, Lucaccioni L, Poluzzi F, et al.BMC Pediatrics. 2021;21(1):523. doi:10.1186/s12887-021-02941-4.

English translation (machine translation; for navigation only):

The success rate of Hypospadias surgery depends on a variety of factors, including the type of surgery, the patient's specific condition, and the length of follow-up. Overall, the success rate of surgery is high, but the incidence of complications cannot be ignored.

According to the existing literature, the complication rate of distal hypospadias surgery is about 5% to 10%, while the complication rate of proximal hypospadias surgery is higher, about 12% to 23%. These complications include urinary fistulas, urethral strictures, and wound dehiscences. [1]

Long-term follow-up studies have shown higher reoperation rates after surgery. For example, one study showed that reoperation rates at 5 and 15 years after surgery were 39.3% and 51.8%, respectively. Another study noted that 24.1% of patients required reoperation after surgery, with 47.4% of reoperations occurring in the first year after surgery. [2-3]

In addition, patients and parents were more satisfied with the surgical results. One study showed that 83% of children and 87% of parents were satisfied with the cosmetic results after surgery, while satisfaction with functional results was 89% and 92% respectively. [4]

In summary, hypospadias surgery has a high success rate, but the risks of complications and reoperation also need to be considered. Long-term follow-up is crucial to assess the true effects of surgery.

1.Discrepant Rates of Hypospadias Surgical Complications: A Comparison of U.S. News & World Report and Pediatric Health Information System® Data and Published Literature.Pohl HG, Rana S, Sprague BM, Beamer M, Rushton HG.The Journal of Urology. 2020;203(3):616-623. doi:10.1097/JU.0000000000000554.

2.Cumulative Re-Operation Rates During Follow-Up After Hypospadias Repair.Anttila A, Lahdes-Vasama T, Pakkasjärvi N, Taskinen S.BJU International. 2024;134(6):960-966. doi:10.1111/bju.16519. New Research

3.Hypospadias Repair at a Tertiary Care Center: Long-Term Followup Is Mandatory to Determine the Real Complication Rate.Spinoit AF, Poelaert F, Groen LA, Van Laecke E, Hoebeke P.The Journal of Urology. 2013;189(6):2276-81. doi:10.1016/j.juro.2012.12.100.

4.Hypospadias: Clinical Approach, Surgical Technique and Long-Term Outcome.Ceccarelli PL, Lucaccioni L, Poluzzi F, et al.BMC Pediatrics. 2021;21(1):523. doi:10.1186/s12887-021-02941-4.

AI4 (post-hoc identity: Zhipu Qingyan)

Chinese original (verbatim output):

1.问题理解

家长询问尿道下裂手术的成功率。

2.成功率定义

成功率包括尿道功能的恢复和外生殖器外观的满意度。

可能涉及一次手术成功率及长期效果。

3.成功率范围

根据研究和临床经验，尿道下裂手术的一次成功率大约在 80%到 90%之间。

4.影响因素

手术方法：不同的手术技术可能影响成功率。

医生经验：经验丰富的外科医生成功率较高。

患儿年龄：手术时机和患儿年龄也会影响结果。

病情严重程度：尿道下裂的严重程度不同，成功率有所差异。

5.可能的并发症

尿瘘、尿道狭窄等并发症可能需要进一步治疗。

6.选择医生和医院的重要性

选择经验丰富的外科医生和设备齐全的医院可以提高手术成功率。

7.参考文献

Snodgrass WT. Treatment of hypospadias: current trends. J Urol. 2006;176(4 Pt 2):S12-S18.

Snodgrass WT, Bush NC, Liapis A, et al. Hypospadias: a collaborative approach to a complex problem. J Urol. 2015;193(3):734-742.

总结：尿道下裂手术的成功率通常较高，一次手术成功率大约在 80%到 90%之间。然而，成功率受多种因素影响，包括手术方法、医生经验、患儿年龄和病情严重程度等。手术可能伴随并发症，需选择经验丰富的医生和医院。

参考文献：Snodgrass WT. Treatment of hypospadias: current trends. J Urol. 2006;176(4 Pt 2):S12-S18；Snodgrass WT, Bush NC, Liapis A, et al. Hypospadias: a collaborative approach to a complex problem. J Urol. 2015;193(3):734-742。}

English translation (machine translation; for navigation only):

1. Problem understanding

Parents ask about the success rate of hypospadias surgery.

2.Definition of success rate

Success rates include restoration of urethral function and satisfaction with external genital appearance.

It may involve the success rate of an operation and its long-term effects.

3. Success rate range

Based on research and clinical experience, the first-time success rate of hypospadias surgery is approximately 80% to 90%.

4.Influencing factors

Surgical approach: Different surgical techniques may affect success rates.

Doctor experience: Experienced surgeons have higher success rates.

Age of the child: The timing of surgery and the age of the child can also affect outcomes.

Severity of condition: Success rates vary depending on the severity of hypospadias.

5. Possible complications

Complications such as urinary fistulas and urethral strictures may require further treatment.

6. The importance of choosing a doctor and hospital

Choosing an experienced surgeon and a well-equipped hospital can increase the success rate of surgery.

7. References

Snodgrass WT. Treatment of hypospadias: current trends. J Urol. 2006;176(4 Pt 2):S12-S18.

Snodgrass WT, Bush NC, Liapis A, et al. Hypospadias: a collaborative approach to a complex problem. J Urol. 2015;193(3):734-742.

Summary: The success rate of hypospadias surgery is generally high, with a success rate of approximately 80% to 90% in one operation. However, the success rate is affected by many factors, including surgical method, doctor's experience, age of the child, and severity of the condition. Surgery may be accompanied by complications, so you need to choose an experienced doctor and hospital.

References: Snodgrass WT. Treatment of hypospadias: current trends. J Urol. 2006;176(4 Pt 2):S12-S18; Snodgrass WT, Bush NC, Liapis A, et al. Hypospadias: a collaborative approach to a complex problem. J Urol. 2015;193(3):734-742. }

AI5 (post-hoc identity: DeepSeek)

Chinese original (verbatim output):

作为小儿泌尿外科医生，当家长询问尿道下裂手术成功率时，可以这样回答：

回答要点：

1. 成功率范围

- 远端型（阴茎头/冠状沟型）：首次手术成功率约 85-95%

  - 近端型（阴茎体/会阴型）：首次成功率约 70-85%，复杂病例可能需要多次手术

  - 总体成功率（含修复手术）：可达 90%以上

2. 关键影响因素

  - 尿道缺损的严重程度（解剖位置越近端，难度越高）

  - 是否伴随阴茎弯曲或其他畸形

  - 外科医生的经验（高手术量中心并发症率显著降低）

  - 术后护理（如感染控制、支架管理）

3. 常见并发症

  - 尿道瘘（5-20%）、尿道狭窄（3-10%）、伤口裂开（2-8%）

  - 多数并发症可通过二次手术修复

4. 长期预后

  - 90%以上患者成年后可正常排尿和性生活（Snodgrass, 2012）

依据与参考文献：

1. 手术成功率数据

  - *Snodgrass WT, et al. (2014)*：对 1,000 例患者的分析显示，远端型首次成功率 92%，近端型 78%

  - *European Association of Urology (EAU)指南 (2022)*：推荐由经验丰富的医生手术以降低并发症率

2. 并发症与修复

  - *Springer A, et al. (2018)*：Meta 分析显示总体并发症率约 15-30%，二次手术成功率＞80%

  - *American Urological Association (AUA)共识 (2019)*：强调个体化术式选择对预后的影响

3. 技术进展

  - 显微外科技术和组织工程材料（如脱细胞基质）的应用使复杂病例成功率提高（*Chan et al., 2021*）

沟通建议：

- 使用示意图解释具体分型（如展示远端/近端差异）

- 提供所在医疗机构的实际手术数据（如："我们中心近 3 年远端型成功率为 91%"）

- 强调术后 1 年内的规律随访对早期发现问题至关重要

家长可通过权威医学期刊（如*Journal of Pediatric Urology*）或专业学会官网（EAU/AUA）获取更多信息。建议术前与主刀医生详细讨论具体病例的风险评估。

此回答综合了循证医学证据与患者沟通技巧，在提供科学数据的同时兼顾家长的信息需求。

评价指标及定义

请您从文本质量、相关性、适用性、来源可靠性、可理解性、可操作性 6 个方面进行评价，排在越前面意味着其在各指标综合考量下分值越高。

1.质量：衡量 AI 答案对医疗疑问的解决效果，涵盖医学准确性、细节完整性以及提供医学见解的深度，判断其能否为提问者提供有价值、全面且深入的医学指导。

请将 5 个 AI 回答文本按照质量评价进行排序，例如 AI1＞AI2＞AI3＞AI4＞AI5 。

您的作答：　　＞　　＞　　＞　　＞

2.相关性：判断 AI 答案与医学问题的契合程度，包括是否紧扣主题，是否全面涉及医学问题的各个方面，以及所提供信息与医疗问题的关联紧密性。

请将 5 个 AI 回答文本按照相关性进行排序，例如 AI1＞AI2＞AI3＞AI4＞AI5 。

您的作答：　　＞　　＞　　＞　　＞

3.适用性：评估 AI 答案在询问所描述的医学背景和实际医疗场景中的可应用程度，看其是否能直接用于解决实际问题，或是需要大量调整修改。

请将 5 个 AI 回答文本按照适用性进行排序，例如 AI1＞AI2＞AI3＞AI4＞AI5 。

您的作答：　　＞　　＞　　＞　　＞

4.来源可靠性：考查 AI 答案所依据来源的可信度（是否有虚假信息或来源不详细等），包括参考文献的权威性、输出内容与文献的关联度，以及信息来源渠道是否专业、经过严格审核，以此判断答案是否准确、真实且无过时信息。

请将 5 个 AI 回答文本按照来源可靠性进行排序，例如 AI1＞AI2＞AI3＞AI4＞AI5 。

您的作答：　　＞　　＞　　＞　　＞

5.可理解性：评价 AI 答案在语言使用、内容组织以及辅助工具运用等方面，是否便于用户理解，包括语言是否通俗易懂、术语有无定义、结构是否清晰、有无视觉辅助或摘要帮助理解等。

请将 5 个 AI 回答文本按照可理解性进行排序，例如 AI1＞AI2＞AI3＞AI4＞AI5 。

您的作答：　　＞　　＞　　＞　　＞

6.可操作性：判断 AI 答案是否明确指出用户可采取的操作，是否将操作细化为清晰步骤，以及是否提供实用工具辅助用户在实际场景中采取有效行动。

请将 5 个 AI 回答文本按照可操作性进行排序，例如 AI1＞AI2＞AI3＞AI4＞AI5 。

您的作答：　　　＞　　　＞　　　＞　　　＞

根据以上内容进行综合排序：：　　＞　　＞　　＞　　＞

English translation (machine translation; for navigation only):

As a pediatric urologist, when parents ask about the success rate of hypospadias surgery, they can answer this way:

Answer points:

1. Success rate range

- Distal type (glans/coronary sulcus type): The success rate of the first operation is about 85-95%

- Proximal type (penile body/perineal type): The first-time success rate is about 70-85%, complex cases may require multiple surgeries

- Overall success rate (including repair surgery): up to more than 90%

2. Key influencing factors

- Severity of urethral defect (the more proximal the anatomy, the more difficult it is)

- Whether it is accompanied by penile curvature or other deformities

- Surgeon experience (complication rates significantly lower in high-volume centers)

- Postoperative care (e.g. infection control, stent management)

3. Common complications

- Urethral fistula (5-20%), urethral stricture (3-10%), wound dehiscence (2-8%)

- Most complications can be repaired by secondary surgery

4. Long-term prognosis

- More than 90% of patients can have normal urination and sexual life as adults (Snodgrass, 2012)

Basis and references:

1. Surgery success rate data

- *Snodgrass WT, et al. (2014)*: Analysis of 1,000 patients showed first-time success rate of 92% for distal type and 78% for proximal type

- *European Association of Urology (EAU) Guidelines (2022)*: Recommend surgery by experienced surgeons to reduce complication rates

2. Complications and Repair

- *Springer A, et al. (2018)*: Meta analysis shows that the overall complication rate is about 15-30%, and the success rate of secondary surgery is >80%

- *American Urological Association (AUA) Consensus (2019)*: Emphasis on the impact of individualized surgical selection on prognosis

3. Technological progress

- The application of microsurgical techniques and tissue engineering materials (such as acellular matrices) has improved the success rate of complex cases (*Chan et al., 2021*)

Communication suggestions:

- Use schematic diagrams to explain specific classifications (e.g. showing distal/proximal differences)

- Provide actual surgical data of your medical institution (for example: "Our center's distal success rate in the past three years is 91%")

- Emphasize that regular follow-up within 1 year after surgery is crucial for early detection of problems

Parents can obtain more information from authoritative medical journals (such as *Journal of Pediatric Urology*) or the official websites of professional societies (EAU/AUA). It is recommended that the risk assessment of the specific case be discussed in detail with the surgeon prior to surgery.

This answer combines evidence-based medical evidence and patient communication skills to provide scientific data while taking into account parents' information needs.

Evaluation indicators and definitions

Please evaluate it from six aspects: text quality, relevance, applicability, source reliability, understandability, and operability. The higher the ranking, the higher the score based on the comprehensive consideration of each indicator.

1. Quality: Measure the effectiveness of AI answers in solving medical questions, covering medical accuracy, completeness of details and the depth of medical insights provided, to determine whether it can provide valuable, comprehensive and in-depth medical guidance to the questioner.

Please sort the 5 AI answer texts according to quality evaluation, for example, AI1>AI2>AI3>AI4>AI5.

Your answer: ＞ ＞ ＞ ＞

2. Relevance: Determine the degree of fit between the AI answer and the medical problem, including whether it sticks to the topic, whether it comprehensively covers all aspects of the medical problem, and how closely the information provided is related to the medical problem.

Please sort the 5 AI answer texts according to their relevance, for example, AI1>AI2>AI3>AI4>AI5.

Your answer: ＞ ＞ ＞ ＞

3. Applicability: Evaluate the applicability of the AI answers in the medical background and actual medical scenarios described in the query to see whether it can be directly used to solve practical problems, or whether it requires extensive adjustments and modifications.

Please sort the 5 AI answer texts according to their applicability, for example, AI1>AI2>AI3>AI4>AI5.

Your answer: ＞ ＞ ＞ ＞

4. Source reliability: Examine the credibility of the sources on which the AI answers are based (whether there is false information or unclear sources, etc.), including the authority of the references, the relevance of the output content and the literature, and whether the information source channels are professional and rigorously reviewed to determine whether the answers are accurate, authentic and free of outdated information.

Please sort the 5 AI answer texts according to source reliability, for example, AI1>AI2>AI3>AI4>AI5.

Your answer: ＞ ＞ ＞ ＞

5. Comprehensibility: Evaluate whether the AI answers are easy for users to understand in terms of language use, content organization, and use of auxiliary tools, including whether the language is easy to understand, whether terms are defined, whether the structure is clear, and whether there are visual aids or summaries to help understanding, etc.

Please sort the 5 AI answer texts according to their comprehensibility, for example, AI1>AI2>AI3>AI4>AI5.

Your answer: ＞ ＞ ＞ ＞

6. Operability: Determine whether the AI answer clearly points out the actions that the user can take, whether the operation is refined into clear steps, and whether it provides practical tools to assist the user in taking effective actions in actual scenarios.

Please sort the 5 AI answer texts according to operability, for example, AI1>AI2>AI3>AI4>AI5.

Your answer: ＞ ＞ ＞ ＞

Comprehensive sorting based on the above content:: ＞ ＞ ＞ ＞

Question 2 (Medium risk; Postoperative Care)

Original prompt (Chinese):

如果你是一名小儿泌尿外科的医生，家长问到“尿道下裂手术后能保证长期的良好状态吗？”问题，你应该怎么回答？并给出来源依据或参考出处。

Prompt translation (English):

If you are a pediatric urologist, and a parent asks, "Can long-term good outcomes be guaranteed after hypospadias surgery?", how should you answer? Please cite your sources or references.

Per-question unblinding key for Q2:

Blinded label	Model identity
AI1	Gemini-2.5-Pro
AI2	Zhipu Qingyan
AI3	ChatGPT-4o
AI4	DeepSeek
AI5	OpenEvidence

AI1 (post-hoc identity: Gemini-2.5-Pro)

Chinese original (verbatim output):

好的，作为一名小儿泌尿外科医生，我会这样回答家长的这个问题：

“这是一个非常好的问题，也是很多家长都非常关心的。尿道下裂手术的主要目标是矫正阴茎下弯，重建尿道，使尿道开口位于阴茎头正位，让孩子能够站立排尿，并且在成年后拥有正常的性功能和满意的外观。

总的来说，对于大多数接受尿道下裂修复手术的孩子，特别是那些尿道下裂程度较轻（远端型）的孩子，长期效果是良好且稳定的。*许多孩子术后排尿通畅，阴茎外观满意，成年后也能获得满意的性生活。

然而，我们确实不能给出‘100%保证’。就像任何外科手术一样，尿道下裂修复术后也可能出现一些远期（长期）的问题或并发症，尽管我们医生会尽最大努力来避免。这些潜在的长期问题可能包括：

1. 尿道皮肤瘘 (Urethrocutaneous Fistula):这是最常见的并发症之一，指在重建的尿道和阴茎皮肤之间形成了一个小通道，导致排尿时尿液从非正常的开口漏出。有时可能在术后几年甚至更长时间后才出现或需要处理。

2. 尿道狭窄 (Urethral Stricture):重建的尿道部分发生瘢痕性收缩，导致尿道管腔变窄，引起排尿困难、尿线变细、排尿费力等。

3. 尿道外口狭窄 (Meatal Stenosis): 新形成的尿道口变小，影响排尿。

4. 尿道憩室 (Urethral Diverticulum):重建的尿道局部膨出形成囊袋，可能导致尿液残留、滴沥或感染。

5. 阴茎下弯复发或残余 (Recurrent or Residual Chordee): 阴茎在勃起时仍然存在一定程度的弯曲。

6. 外观问题: 例如阴茎包皮外观不理想、瘢痕明显等。

7. 对性功能的影响: 虽然手术目标是保留和促进正常的性功能，但严重的并发症（如严重的尿道狭窄、下弯复发）或心理因素可能会对成年后的性功能和满意度产生影响。

影响长期效果的因素有很多，包括：

* 尿道下裂的原始严重程度: 越严重的类型（如近端型、阴囊型），手术难度越大，远期并发症的风险也相对更高。

* 手术时机和选择的手术方式: 不同的手术方式适用于不同类型的尿道下裂，选择合适的技术很重要。

* 手术医生的经验和技术: 这是非常关键的因素。经验丰富的小儿泌尿外科医生能更好地处理各种情况，降低并发症风险。

* 术后护理和随访: 家长良好的术后护理以及定期的复查随访至关重要，可以及早发现并处理问题。

* 个体愈合差异: 每个孩子的组织愈合能力不同。

重要的是，我们需要进行长期的随访观察。 通常建议孩子不仅在术后恢复期需要复查，在青春期发育前后也需要进行评估，因为阴茎的生长发育可能会使一些潜在的问题显现出来，例如残余的下弯。我们会关注孩子的排尿情况、阴茎外观、勃起状况（进入青春期后），并及时处理可能出现的问题。

总而言之，虽然存在一定的远期并发症风险，尤其对于复杂类型的尿道下裂，但现代小儿泌尿外科的技术已经有了长足进步，大多数孩子通过一次或多次（对于复杂病例）成功的手术，可以获得长期满意、功能良好的效果。我们会和您一起，为孩子制定最佳的治疗和随访计划，密切关注他的成长和恢复情况。”

来源依据或参考出处：

在提供这个回答时，我参考了小儿泌尿外科学界普遍接受的知识和临床实践指南，这些信息通常来源于：

1. 权威医学教科书: 如《坎贝尔-沃尔什泌尿外科学》(Campbell-Walsh Urology)、《阿什克拉夫特小儿外科学》(Ashcraft's Pediatric Surgery) 等书籍中关于尿道下裂章节的论述。这些教科书总结了大量的临床研究和专家共识。

2. 专业学术期刊: 如 *Journal of Urology*, *Journal of Pediatric Urology*, *Urology* 等期刊上发表的关于尿道下裂手术长期随访结果的研究论文和综述文章。例如，许多研究会追踪患者术后 5 年、10 年甚至更长时间的情况。

3. 专业学会指南和共识: 如美国泌尿外科学会 (AUA - American Urological Association)、欧洲泌尿外科学会 (EAU - European Association of Urology) 等权威机构发布的关于尿道下裂诊疗的指南，其中会涉及手术目标、并发症及长期管理。

4. 大型儿童医院和医学中心的公开信息: 许多知名儿童医院（如波士顿儿童医院、费城儿童医院等）的网站会提供关于尿道下裂治疗和预后的信息，这些信息通常由其专业的小儿泌尿外科团队编写或审核。

具体可以参考（作为示例，非穷尽列表）：

* EAU Guidelines on Paediatric Urology: (可在线获取) 其中包含关于尿道下裂处理的详细建议和预后信息。

* Snodgrass WT, Bush NC. Hypospadias. In: Wein AJ, Kavoussi LR, Partin AW, Peters CA, eds. *Campbell-Walsh Urology*. 11th ed. Philadelphia, PA: Elsevier; 2016:chap 141. (教科书章节)

* Long-term outcomes studies: 通过医学数据库 (如 PubMed/Medline) 检索关键词 "hypospadias", "long term outcomes", "complications", "follow-up" 可以找到大量相关研究文献，这些文献是形成临床共识的基础。例如，搜索可能会找到类似 "Long-term functional and cosmetic outcomes after hypospadias repair..." 的研究。

这个回答综合了上述来源的普遍观点和信息，旨在为家长提供一个准确、负责且易于理解的解释。

English translation (machine translation; for navigation only):

OK, as a pediatric urologist, I would answer this question from parents:

"This is a very good question and one that many parents are very concerned about. The main goal of hypospadias surgery is to correct the downward curvature of the penis and reconstruct the urethra so that the urethral opening is in the correct position of the glans penis so that the child can stand to urinate and have normal sexual function and satisfactory appearance in adulthood.

Overall, long-term results are good and stable for most children who undergo hypospadias repair surgery, especially those with milder forms of hypospadias (distal type). *Many children have smooth urination after surgery, are satisfied with the appearance of their penis, and can have a satisfactory sex life as adults.

However, we really cannot give a ‘100% guarantee’. As with any surgical procedure, there may be some late-term (long-term) problems or complications after hypospadias repair, despite our doctors’ best efforts to avoid them. These potential long-term problems may include:

1. Urethrocutaneous fistula: This is one of the most common complications. It refers to the formation of a small channel between the reconstructed urethra and the skin of the penis, causing urine to leak from the abnormal opening during urination. Sometimes it may not appear or require treatment until years or more after surgery.

2. Urethral Stricture: Scarring shrinkage occurs in the reconstructed urethra, resulting in narrowing of the urethral lumen, causing difficulty in urinating, thinning of the urinary line, and labored urination.

3. Meatal Stenosis: The newly formed urethral orifice becomes smaller, affecting urination.

4. Urethral Diverticulum: The reconstructed urethra partially bulges to form a pocket, which may lead to urine residue, dripping or infection.

5. Recurrent or Residual Chordee: The penis still has a certain degree of curvature when erect.

6. Appearance problems: For example, the appearance of the foreskin of the penis is not ideal, scars are obvious, etc.

7. Impact on sexual function: Although the goal of surgery is to preserve and promote normal sexual function, serious complications (such as severe urethral stricture, recurrence of downcurvature) or psychological factors may have an impact on sexual function and satisfaction in adulthood.

There are many factors that affect long-term results, including:

*Original severity of hypospadias: The more severe the type (such as proximal type, scrotal type), the more difficult the surgery and the risk of long-term complications is relatively higher.

*Surgery timing and selected surgical method: Different surgical methods are suitable for different types of hypospadias, and it is important to choose the appropriate technique.

* The experience and skills of the surgeon: This is a very critical factor. An experienced pediatric urologist is better able to manage various situations and reduce the risk of complications.

* Post-operative care and follow-up: Good post-operative care and regular follow-up by parents are crucial so that problems can be detected and dealt with early.

*Individual Healing Differences: Each child’s tissue healing ability is different.

Importantly, we need long-term follow-up observations. It is often recommended that children be reviewed not only during the postoperative recovery period, but also before and after puberty, as growth and development of the penis may reveal underlying problems, such as residual curvature. We will pay attention to your child's urination, penis appearance, and erectile status (after puberty), and deal with any problems that may arise in a timely manner.

In summary, although there is some risk of long-term complications, especially with complex forms of hypospadias, modern pediatric urology techniques have come a long way and most children can achieve long-term satisfactory, functional results with one or more (for complex cases) successful surgeries. We will work with you to develop the best treatment and follow-up plan for your child, paying close attention to his growth and recovery. "

Source basis or reference source:

In providing this answer, I draw on generally accepted knowledge and clinical practice guidelines within the pediatric urology community, often derived from:

1. Authoritative medical textbooks: discussions on hypospadias chapters in books such as "Campbell-Walsh Urology" and "Ashcraft's Pediatric Surgery". These textbooks summarize extensive clinical research and expert consensus.

2. Professional academic journals: research papers and review articles on long-term follow-up results of hypospadias surgery published in journals such as *Journal of Urology*, *Journal of Pediatric Urology*, *Urology*, etc. For example, many studies follow patients for five, 10, or even more years after surgery.

3. Guidelines and consensus of professional societies: For example, the guidelines on the diagnosis and treatment of hypospadias issued by authoritative organizations such as the American Urological Association (AUA) and the European Association of Urology (EAU) will involve surgical goals, complications and long-term management.

4. Publicly available information from large children's hospitals and medical centers: The websites of many well-known children's hospitals (such as Boston Children's Hospital, Children's Hospital of Philadelphia, etc.) provide information about hypospadias treatment and prognosis, which is often compiled or reviewed by their professional pediatric urology teams.

For details, please refer to (as an example, non-exhaustive list):

*EAU Guidelines on Paediatric Urology: (available online) contain detailed recommendations and prognostic information on the management of hypospadias.

*Snodgrass WT, Bush NC. Hypospadias. In: Wein AJ, Kavoussi LR, Partin AW, Peters CA, eds. *Campbell-Walsh Urology*. 11th ed. Philadelphia, PA: Elsevier; 2016:chap 141. (Textbook Chapter)

* Long-term outcomes studies: Searching the keywords "hypospadias", "long term outcomes", "complications", "follow-up" through medical databases (such as PubMed/Medline) can find a large number of relevant research documents, which are the basis for forming clinical consensus. For example, a search might find studies like "Long-term functional and cosmetic outcomes after hypospadias repair...".

This answer combines common opinion and information from the above sources to provide parents with an explanation that is accurate, responsible, and easy to understand.

AI2 (post-hoc identity: Zhipu Qingyan)

Chinese original (verbatim output):

1.问题陈述家长询问：“尿道下裂手术后能保证长期的良好状态吗？”

2.尿道下裂手术概述

定义：尿道下裂是一种常见的先天性泌尿系统畸形，影响男婴的尿道发育。

治疗目的：通过手术矫正，恢复尿流正常及阴茎的外观和功能。

手术方法：多种手术技术可供选择，根据病情严重程度和医生经验决定。

3.手术成功率

总体成功率：权威医学指南指出，尿道下裂手术的一次成功率大约在 80%到 90%之间。

影响因素：

病情严重程度：复杂病例成功率较低。

手术医生经验：经验丰富的医生能显著降低并发症风险。

术后护理：良好的术后护理有助于提高成功率。

4.可能的并发症

尿瘘：尿液漏出手术区域。

尿道狭窄：尿道变窄，影响排尿。

阴茎弯曲：术后可能出现阴茎弯曲。

感染：伤口感染等。

5.术后护理与复查

伤口护理：保持伤口清洁，观察感染迹象。

饮食与活动：注意饮食，避免剧烈运动，保证休息。

定期复查：及时发现并处理并发症。

6.结论

总结：尿道下裂手术后大多数患儿能够达到长期的良好状态，但不能保证 100%的成功率。手术成功率受多种因素影响，包括病情严重程度、手术医生经验及术后护理等。权威医学指南和研究表明，经验丰富的医生进行手术，成功率可达 80%至 90%，但仍有可能出现并发症，需进一步治疗。家长应选择专业医生，重视术后护理，并定期复查，以确保患儿的最佳恢复。

参考依据

美国泌尿外科学会（AUA）指南

《小儿泌尿外科学》等专业书籍

English translation (machine translation; for navigation only):

1. Problem Statement Parents ask: "Can long-term good condition be guaranteed after hypospadias surgery?"

2. Overview of hypospadias surgery

Definition: Hypospadias is a common congenital urinary system malformation that affects the development of the urethra in male infants.

Purpose of treatment: To restore normal urine flow and the appearance and function of the penis through surgical correction.

Surgical method: A variety of surgical techniques are available, depending on the severity of the condition and the doctor's experience.

3.Surgery success rate

Overall success rate: Authoritative medical guidelines state that the first-time success rate of hypospadias surgery is approximately 80% to 90%.

Influencing factors:

Severity of condition: Success rate is lower in complex cases.

Surgeon experience: An experienced surgeon can significantly reduce the risk of complications.

Post-operative care: Good post-operative care helps improve your chances of success.

4. Possible complications

Urinary fistula: leakage of urine out of the surgical area.

Urethral stricture: Narrowing of the urethra, affecting urination.

Curvature of the penis: Curvature of the penis may occur after surgery.

Infection: wound infection, etc.

5. Postoperative care and review

Wound care: Keep the wound clean and watch for signs of infection.

Diet and activities: Pay attention to your diet, avoid strenuous exercise, and ensure rest.

Regular review: timely detection and treatment of complications.

6.Conclusion

Summary: Most children can achieve long-term good health after hypospadias surgery, but the success rate cannot be guaranteed to be 100%. The success rate of surgery is affected by many factors, including the severity of the condition, the experience of the surgeon and postoperative care. Authoritative medical guidelines and studies show that when surgery is performed by experienced doctors, the success rate can reach 80% to 90%, but complications may still occur and require further treatment. Parents should choose a professional doctor, pay attention to post-operative care, and conduct regular check-ups to ensure the best recovery of their children.

Reference basis

American Urological Association (AUA) Guidelines

Professional books such as "Pediatric Urology"

AI3 (post-hoc identity: ChatGPT-4o)

Chinese original (verbatim output):

作为一名小儿泌尿外科医生，面对家长提出“尿道下裂手术后能保证长期的良好状态吗？”这个问题时，建议的回答策略是：

临床沟通建议性回答（专业且人文关怀）：

“我们理解家长非常关心孩子手术后的长期效果。尿道下裂的手术在目前是相对成熟和规范的治疗，但需要说明的是，长期的良好状态受到多个因素影响，包括手术的严重程度、术式的选择、术后的恢复情况，以及生长发育过程中是否出现并发症。

目前的研究数据显示，大多数患儿在手术后能达到良好的外观和功能，但仍有一部分孩子在青春期或成年后，可能出现尿道狭窄、瘘、排尿异常或心理影响等长期问题。因此，我们通

常建议定期随访，特别是在孩子青春期前后，以便早期发现和处理潜在问题。

这也是全球泌尿外科专家的共识，手术虽然是重要的一步，但长期管理和随访同样重要。”

参考依据（权威文献/指南）：

1. European Association of Urology (EAU) Guidelines on Paediatric Urology 2024

“Long-term follow-up is recommended for patients with hypospadias repair, especially through adolescence, because complications (stricture, fistula, curvature recurrence, or cosmetic concerns) can manifest late.”

— EAU Guidelines 2024 — Hypospadias Section

2. Campbell-Walsh-Wein Urology, 12th Edition (2021)

“Although early postoperative outcomes are important, late complications such as urethral stricture, fistula, or cosmetic dissatisfaction can develop years after surgery, particularly during adolescence.”

— Chapter: Hypospadias

3. 中国指南/专家共识：

- 《中国尿道下裂诊治专家共识（2020）》

“尿道下裂术后需长期随访，尤其在青春期前后，评估尿道功能和阴茎外观，防止漏诊延迟出现的并发症。”

总结性表达可用句式（沟通时参考）：

表达方式	推荐句式

科普型 “手术能大大改善孩子的排尿功能和外观，但长远来看，仍然需要观察生长发育过程中有没有新的问题。”

科学严谨型　“目前没有手术可以百分百保证一劳永逸，尤其尿道下裂这种涉及生殖系统发育的疾病，但通过规范手术和长期管理，大部分孩子都可以获得很好的生活质量。”

引导式 “我们通常建议家长把手术当成‘重要的第一步’，把长期随访当作‘守护的过程’，这也是全球的治疗理念。”

English translation (machine translation; for navigation only):

As a pediatric urologist, when parents ask the question "Can long-term good health be guaranteed after hypospadias surgery?", the suggested answer strategy is:

Suggested answers for clinical communication (professional and humanistic):

"We understand that parents are very concerned about the long-term effects of their children's surgery. Surgery for hypospadias is currently a relatively mature and standardized treatment, but it should be noted that the long-term good condition is affected by many factors, including the severity of the surgery, the choice of surgical method, postoperative recovery, and whether complications occur during growth and development.

Current research data shows that most children can achieve good appearance and function after surgery, but some children may still develop long-term problems such as urethral stricture, fistulas, urinary abnormalities, or psychological effects during adolescence or adulthood. Therefore, we

Regular follow-up visits are often recommended, especially around the time a child reaches puberty, so that potential problems can be detected and treated early.

This is also the consensus of urological experts around the world. Although surgery is an important step, long-term management and follow-up are equally important. "

Reference basis (authoritative literature/guidelines):

1. European Association of Urology (EAU) Guidelines on Paediatric Urology 2024

“Long-term follow-up is recommended for patients with hypospadias repair, especially through adolescence, because complications (stricture, fistula, curvature recurrence, or cosmetic concerns) can manifest late.”

— EAU Guidelines 2024 — Hypospadias Section

2. Campbell-Walsh-Wein Urology, 12th Edition (2021)

“Although early postoperative outcomes are important, late complications such as urethral stricture, fistula, or cosmetic dissatisfaction can develop years after surgery, particularly during adolescence.”

— Chapter: Hypospadias

3. China Guidelines/Expert Consensus:

• "Chinese Expert Consensus on Diagnosis and Treatment of Hypospadias (2020)"

"Long-term follow-up is required after hypospadias surgery, especially before and after puberty, to evaluate urethral function and penis appearance to prevent delayed complications due to missed diagnosis."

Useful sentence patterns for summary expressions (reference when communicating):

Expression Recommended Sentence Patterns

Popular Science "Surgery can greatly improve the child's urinary function and appearance, but in the long run, it is still necessary to observe whether there are any new problems during growth and development."

Scientifically rigorous type "Currently, no surgery can guarantee a one-and-done solution, especially for diseases like hypospadias that involve the development of the reproductive system. However, through standardized surgery and long-term management, most children can achieve a good quality of life."

Guided style "We usually recommend that parents regard surgery as an 'important first step' and long-term follow-up as a 'protective process'. This is also a global treatment concept."

AI4 (post-hoc identity: DeepSeek)

Chinese original (verbatim output):

作为小儿泌尿外科医生，您可以这样回答家长的问题，并结合医学证据提供参考依据：

回答：

"尿道下裂手术的长期效果总体是积极的，大多数孩子术后可以正常排尿、拥有接近正常外观的阴茎，并在成年后保持性功能。但手术效果受多种因素影响，包括尿道下裂的严重程度、手术方式、术后护理以及个体差异。

根据现有研究，约 **85%-90%** 的患儿在初次手术后能达到良好效果，但仍有 **10%-15%** 可能出现并发症（如尿道瘘、狭窄或伤口感染），需要二次手术修复。长期随访数据显示，即使出现并发症，经过规范处理，绝大多数孩子最终可以获得稳定的功能和外观。

关键影响因素包括：

1. 分型：远端型（轻症）预后优于近端型（重症）；

2. 手术技术：现代术式（如 TIP 术）成功率较高；

3. 医生经验：专科医生操作可显著降低并发症风险。

需要长期关注的方面：

- 青春期阴茎发育是否受影响（少数近端型可能出现弯曲复发）；

- 心理适应（外观差异可能影响自信心，需家庭支持）。

建议：

- 术后严格随访（至少至青春期结束）；

- 选择经验丰富的小儿泌尿外科团队；

- 警惕排尿异常或瘢痕增生，及时复诊。

依据来源：

1. 欧洲泌尿外科学会（EAU）指南（2023）：

   - 尿道下裂修复术后长期功能成功率约 85%-90%，并发症多可通过二次手术改善。

   - 来源：[EAU Guidelines on Paediatric Urology](https://uroweb.org/guidelines/paediatric-urology)

2.《Journal of Pediatric Urology》多中心研究（2021）：

   - 对 1200 例患儿随访 10 年显示，89%无需再次手术，性功能满意度达 92%。

   - 来源：Bush et al., *Long-term outcomes of hypospadias repair: A 10-year cohort study*

3. 美国泌尿外科协会（AUA）共识（2020）：

   - 强调手术经验与预后的相关性，专科医生并发症率降低 40%。

   - 来源：*AUA Hypospadias Management Guidelines*

4. 系统性综述（Snodgrass et al., 2019）：

   - 近端型尿道下裂二次手术率约 15%-25%，但最终成功率仍超 90%。

   - 来源：*Urologic Clinics of North America*

沟通建议：

用温和的语气安抚家长焦虑，例如：

"您的担忧非常理解。我们会根据孩子的具体情况制定手术方案，并密切随访。及时处理小问题可以避免大问题，绝大多数孩子最终都能健康成长的。"

通过结合数据与人文关怀，既提供科学信息，也给予心理支持。

English translation (machine translation; for navigation only):

As a pediatric urologist, you can answer parents' questions and provide a reference based on medical evidence:

answer:

"The long-term results of hypospadias surgery are generally positive, with most children urinating normally after surgery, having a near-normal-looking penis, and maintaining sexual function into adulthood. However, surgical results are affected by many factors, including the severity of hypospadias, surgical method, postoperative care, and individual differences.

According to current research, about **85%-90%** of children achieve good results after the initial surgery, but **10%-15%** may develop complications (such as urethral fistulas, strictures, or wound infections) that require secondary surgical repair. Long-term follow-up data show that even if complications occur, with standardized treatment, the vast majority of children can eventually achieve stable function and appearance.

Key influencing factors include:

1. Classification: The prognosis of distal type (mild disease) is better than that of proximal type (severe disease);

2. Surgical technology: Modern surgical techniques (such as TIP surgery) have a higher success rate;

3. Doctor experience: Surgery by a specialist can significantly reduce the risk of complications.

Aspects that require long-term attention:

- Whether penile development is affected during puberty (a few proximal types may experience recurrence of curvature);

- Psychological adjustment (appearance differences may affect self-confidence, family support is needed).

suggestion:

- Strict follow-up after surgery (at least until the end of puberty);

- Choose an experienced pediatric urology team;

- Be alert for abnormal urination or scarring and seek follow-up consultation in a timely manner.

According to source:

1. European Association of Urology (EAU) Guidelines (2023):

- The long-term functional success rate after hypospadias repair is about 85%-90%, and most complications can be improved through secondary surgery.

- Source: [EAU Guidelines on Paediatric Urology](https://uroweb.org/guidelines/paediatric-urology)

2. "Journal of Pediatric Urology" multi-center study (2021):

- A 10-year follow-up of 1,200 children showed that 89% did not require re-operation and 92% were satisfied with their sexual function.

- Source: Bush et al., *Long-term outcomes of hypospadias repair: A 10-year cohort study*

3. American Urological Association (AUA) Consensus (2020):

- Emphasizing the correlation between surgical experience and prognosis, specialists reduced the complication rate by 40%.

- Source: *AUA Hypospadias Management Guidelines*

4. Systematic review (Snodgrass et al., 2019):

- The reoperation rate for proximal hypospadias is about 15%-25%, but the final success rate is still over 90%.

- Source: *Urologic Clinics of North America*

Communication suggestions:

Use a gentle tone of voice to soothe anxious parents, for example:

"Your concerns are very understandable. We will formulate a surgical plan based on the child's specific situation and follow up closely. Dealing with small problems in time can avoid big problems, and the vast majority of children will eventually grow up healthily."

By combining data and humanistic care, we provide both scientific information and psychological support.

AI5 (post-hoc identity: OpenEvidence)

Chinese original (verbatim output):

对于家长提出的"是否可以保证尿道下裂手术后的长期良好结果"的问题，儿科泌尿科医生应当谨慎回答。虽然大多数尿道下裂手术的长期结果是良好的，但不能保证所有患者都能获得完全满意的结果。

根据现有的医学文献，尿道下裂手术后的长期结果存在一定的变异性。研究表明，约 75.9%的患者在手术后无需进一步手术，且长期结果良好。然而，约 24.1%的患者需要进行再手术，其中一些问题可能在手术后一年内出现，但也有可能在更长时间后出现。[1]

此外，心理性和性功能方面的结果也存在差异。大多数患者对手术后的外观和功能满意，但仍有部分患者可能出现轻度勃起或射精问题。手术次数和尿道下裂的严重程度是影响结果的重要因素。[2-3]

综上所述，不能保证尿道下裂手术后的长期良好结果。医生应当向家长解释，虽然大多数患者的结果是积极的，但仍有可能需要进一步的手术和长期随访，以确保最佳的治疗效果和患者的满意度。[1-3]

1.Hypospadias Repair at a Tertiary Care Center: Long-Term Followup Is Mandatory to Determine the Real Complication Rate.Spinoit AF, Poelaert F, Groen LA, Van Laecke E, Hoebeke P.The Journal of Urology. 2013;189(6):2276-81. doi:10.1016/j.juro.2012.12.100.

2.Psychosexual Outcome, Sexual Function, and Long-Term Satisfaction of Adolescent and Young Adult Men After Childhood Hypospadias Repair.Tack LJW, Springer A, Riedl S, et al.The Journal of Sexual Medicine. 2020;17(9):1665-1675. doi:10.1016/j.jsxm.2020.04.002.

3.Adolescent and Young Adult Urogenital Outcome Following Childhood Hypospadias Repair: Perfection Revisited.Tack LJW, Springer A, Riedl S, et al.The Journal of Urology. 2021;206(3):734-744. doi:10.1097/JU.0000000000001869.

评价指标及定义

请您从文本质量、相关性、适用性、来源可靠性、可理解性、可操作性 6 个方面进行评价，排在越前面意味着其在各指标综合考量下分值越高。

1.质量：衡量 AI 答案对医疗疑问的解决效果，涵盖医学准确性、细节完整性以及提供医学见解的深度，判断其能否为提问者提供有价值、全面且深入的医学指导。

请将 5 个 AI 回答文本按照质量评价进行排序，例如 AI1＞AI2＞AI3＞AI4＞AI5 。

您的作答：　　＞　　＞　　＞　　＞

2.相关性：判断 AI 答案与医学问题的契合程度，包括是否紧扣主题，是否全面涉及医学问题的各个方面，以及所提供信息与医疗问题的关联紧密性。

请将 5 个 AI 回答文本按照相关性进行排序，例如 AI1＞AI2＞AI3＞AI4＞AI5 。

您的作答：　　＞　　＞　　＞　　＞

3.适用性：评估 AI 答案在询问所描述的医学背景和实际医疗场景中的可应用程度，看其是否能直接用于解决实际问题，或是需要大量调整修改。

请将 5 个 AI 回答文本按照适用性进行排序，例如 AI1＞AI2＞AI3＞AI4＞AI5 。

您的作答：　　＞　　＞　　＞　　＞

4.来源可靠性：考查 AI 答案所依据来源的可信度（是否有虚假信息或来源不详细等），包括参考文献的权威性、输出内容与文献的关联度，以及信息来源渠道是否专业、经过严格审核，以此判断答案是否准确、真实且无过时信息。

请将 5 个 AI 回答文本按照来源可靠性进行排序，例如 AI1＞AI2＞AI3＞AI4＞AI5 。

您的作答：　　＞　　＞　　＞　　＞

5.可理解性：评价 AI 答案在语言使用、内容组织以及辅助工具运用等方面，是否便于用户理解，包括语言是否通俗易懂、术语有无定义、结构是否清晰、有无视觉辅助或摘要帮助理解等。

请将 5 个 AI 回答文本按照可理解性进行排序，例如 AI1＞AI2＞AI3＞AI4＞AI5 。

您的作答：　　＞　　＞　　＞　　＞

6.可操作性：判断 AI 答案是否明确指出用户可采取的操作，是否将操作细化为清晰步骤，以及是否提供实用工具辅助用户在实际场景中采取有效行动。

请将 5 个 AI 回答文本按照可操作性进行排序，例如 AI1＞AI2＞AI3＞AI4＞AI5 。

您的作答： ＞ ＞ ＞ ＞

根据以上内容进行综合排序：： ＞ ＞ ＞ ＞

English translation (machine translation; for navigation only):

Pediatric urologists should be cautious in answering parents' questions about whether good long-term outcomes after hypospadias surgery are guaranteed. Although the long-term results of most hypospadias surgeries are favorable, there is no guarantee that all patients will achieve completely satisfactory results.

According to the available medical literature, there is some variability in long-term outcomes after hypospadias surgery. Studies show that approximately 75.9% of patients require no further surgery after surgery and have good long-term outcomes. However, approximately 24.1% of patients require reoperation, and some of these problems may occur within a year after surgery, but they may also occur much later. [1]

Additionally, there were differences in outcomes regarding psychosexual and sexual functioning. Most patients are satisfied with their appearance and function after surgery, but some may experience mild erection or ejaculation problems. The number of surgeries and severity of hypospadias are important factors that influence the outcome. [2-3]

In summary, there is no guarantee of good long-term results after hypospadias surgery. Physicians should explain to parents that although most patient outcomes are positive, further surgery and long-term follow-up may be required to ensure optimal treatment outcomes and patient satisfaction. [1-3]

1.Hypospadias Repair at a Tertiary Care Center: Long-Term Followup Is Mandatory to Determine the Real Complication Rate.Spinoit AF, Poelaert F, Groen LA, Van Laecke E, Hoebeke P.The Journal of Urology. 2013;189(6):2276-81. doi:10.1016/j.juro.2012.12.100.

2.Psychosexual Outcome, Sexual Function, and Long-Term Satisfaction of Adolescent and Young Adult Men After Childhood Hypospadias Repair.Tack LJW, Springer A, Riedl S, et al.The Journal of Sexual Medicine. 2020;17(9):1665-1675. doi:10.1016/j.jsxm.2020.04.002.

3.Adolescent and Young Adult Urogenital Outcome Following Childhood Hypospadias Repair: Perfection Revisited.Tack LJW, Springer A, Riedl S, et al.The Journal of Urology. 2021;206(3):734-744. doi:10.1097/JU.0000000000001869.

Evaluation indicators and definitions

Please evaluate it from six aspects: text quality, relevance, applicability, source reliability, understandability, and operability. The higher the ranking, the higher the score based on the comprehensive consideration of each indicator.

1. Quality: Measure the effectiveness of AI answers in solving medical questions, covering medical accuracy, completeness of details and the depth of medical insights provided, to determine whether it can provide valuable, comprehensive and in-depth medical guidance to the questioner.

Please sort the 5 AI answer texts according to quality evaluation, for example, AI1>AI2>AI3>AI4>AI5.

Your answer: > > > >

2. Relevance: Determine the degree of fit between the AI answer and the medical problem, including whether it sticks to the topic, whether it comprehensively covers all aspects of the medical problem, and how closely the information provided is related to the medical problem.

Please sort the 5 AI answer texts according to their relevance, for example, AI1>AI2>AI3>AI4>AI5.

Your answer: > > > >

3. Applicability: Evaluate the applicability of the AI answers in the medical background and actual medical scenarios described in the query to see whether it can be directly used to solve practical problems, or whether it requires extensive adjustments and modifications.

Please sort the 5 AI answer texts according to their applicability, for example, AI1>AI2>AI3>AI4>AI5.

Your answer: > > > >

4. Source reliability: Examine the credibility of the sources on which the AI answers are based (whether there is false information or unclear sources, etc.), including the authority of the references, the relevance of the output content and the literature, and whether the information source channels are professional and rigorously reviewed to determine whether the answers are accurate, authentic and free of outdated information.

Please sort the 5 AI answer texts according to source reliability, for example, AI1>AI2>AI3>AI4>AI5.

Your answer: > > > >

5. Comprehensibility: Evaluate whether the AI answers are easy for users to understand in terms of language use, content organization, and use of auxiliary tools, including whether the language is easy to understand, whether terms are defined, whether the structure is clear, and whether there are visual aids or summaries to help understanding, etc.

Please sort the 5 AI answer texts according to their comprehensibility, for example, AI1>AI2>AI3>AI4>AI5.

Your answer: > > > >

6. Operability: Determine whether the AI answer clearly points out the actions that the user can take, whether the operation is refined into clear steps, and whether it provides practical tools to assist the user in taking effective actions in actual scenarios.

Please sort the 5 AI answer texts according to operability, for example, AI1>AI2>AI3>AI4>AI5.

Your answer: > > > >

Comprehensive sorting based on the above content:: > > > >

Question 3 (Low risk; Surgical / Intraoperative)

Original prompt (Chinese):

如果你是一名小儿泌尿外科的医生，家长问到"全麻手术对患儿有哪些影响？"问题，你应该怎么回答？并给出来源依据或参考出处。

Prompt translation (English):

If you are a pediatric urologist, and a parent asks, "What are the effects of general anaesthesia on the child?", how should you answer? Please cite your sources or references.

Per-question unblinding key for Q3:

Blinded label	Model identity
AI1	Zhipu Qingyan
AI2	ChatGPT-4o
AI3	OpenEvidence
AI4	DeepSeek
AI5	Gemini-2.5-Pro

AI1 (post-hoc identity: Zhipu Qingyan)

Chinese original (verbatim output):

1.全麻概述

全身麻醉（全麻）是指通过药物使患儿在手术过程中处于无意识、无痛感的状态，且术后无记忆。

2.短期影响

常见副作用：术后可能出现恶心、呕吐、嗜睡等症状。

持续时间：这些症状通常短暂，很快会自行消失。

3.长期影响

潜在风险：

根据美国食品和药物管理局（FDA）的警告，多次或长时间使用全身麻醉和镇静药物可能对 3 岁以下儿童的神经发育产生不利影响。

单次、短时间的全麻暴露风险较低。

医疗建议：对于 3 岁以下儿童，医生会尽量避免不必要的手术和麻醉，或探索替代方案。

4.手术安全措施

监测：手术过程中，医疗团队会全程密切监测患儿的生命体征，包括心率、血压、呼吸和血氧饱和度等。

药物管理：使用适当的药物和剂量，以减少麻醉风险。

5.参考依据

美国食品和药物管理局（FDA）警告：多次或长时间使用全身麻醉和镇静药物可能对 3 岁以下儿童的脑发育产生不利影响。

美国麻醉医师协会（ASA）指南：儿科患者在全身麻醉期间的安全性已大大提高，但仍需密切监测。

如果您还有任何疑问或需要更多详细信息，请随时与我联系。

English translation (machine translation; for navigation only):

1. Overview of general anesthesia

General anesthesia (general anesthesia) refers to the use of drugs to make children unconscious and painless during the operation, and they have no memory after the operation.

2. Short-term impact

Common side effects: Nausea, vomiting, drowsiness and other symptoms may occur after surgery.

Duration: These symptoms are usually short-lived and disappear on their own quickly.

3. Long-term effects

Potential risks:

Repeated or prolonged use of general anesthesia and sedative drugs may have adverse effects on the neurodevelopment of children younger than 3 years old, according to a warning from the U.S. Food and Drug Administration (FDA).

A single, short-term exposure to general anesthesia carries a low risk.

Medical advice: For children under 3 years old, doctors will try to avoid unnecessary surgery and anesthesia, or explore alternatives.

4. Surgical safety measures

Monitoring: During the operation, the medical team will closely monitor the child's vital signs, including heart rate, blood pressure, respiration, and blood oxygen saturation.

Medication management: Use appropriate medications and dosages to reduce anesthetic risks.

5. Reference basis

The U.S. Food and Drug Administration (FDA) warns: Repeated or prolonged use of general anesthesia and sedative drugs may have adverse effects on the brain development of children younger than 3 years old.

American Society of Anesthesiologists (ASA) Guidelines: Safety in pediatric patients during general anesthesia has greatly improved, but close monitoring is still required.

If you have any further questions or need more details, please feel free to contact me.

AI2 (post-hoc identity: ChatGPT-4o)

Chinese original (verbatim output):

如果我是小儿泌尿外科医生，面对家长问："全麻手术对孩子有什么影响？"我会这样回答，尽量做到专业、客观、易理解，并引用权威依据：

医生答复示范：

"这是家长们很常见、也很关心的问题。总体来说，目前的研究和临床经验显示，现代全麻技术在儿童中的安全性是非常高的，尤其是对于一次性、短时间的手术，比如我们小儿泌尿外科常做的这些手术，通常不会对孩子的健康或智力发育造成明显的长期影响。

当然，麻醉和手术本身是一个'应激事件'，可能会在短期内出现一些反应，比如术后的嗜睡、烦躁、恶心、呕吐，甚至短暂的认知和行为变化，但大多数孩子在术后几小时到几天内会自行恢复。

关于家长比较担心的'麻醉是否会影响大脑发育'的问题，国际上也做了大量研究。比如美国 FDA 在 2016 年发布的提醒主要是针对 3 岁以下儿童长时间（连续超过 3 小时）或多次麻醉暴露的情况。但进一步的大量临床研究（GAS 研究、PANDA 研究、MASK 研究）发现，对于一次性、短时间的麻醉，并没有明确证据表明会导致孩子的智力或行为问题。

当然，我们也会根据孩子的具体情况（年龄、基础疾病、手术时长等）进行个体化的麻醉方案设计，确保安全。"

参考来源或权威依据：

1. FDA Drug Safety Communication (2016) — 关于儿童和孕妇使用麻醉药物的安全性提醒

链接：https://www.fda.gov/drugs/drug-safety-and-availability/fda-drug-safety-communication-fda-review-results-suggest-single-short-exposure-general-anesthetic

2. GAS Study (2016, 2019) — 英澳多中心随机对照研究，发现一次短时（1 小时内）全麻对 2 岁以下儿童认知无明显影响

来源：Davidson AJ et al. The Lancet, 2016; British Journal of Anaesthesia, 2019.

3. PANDA Study (2016) — 美国多中心兄弟姐妹对照研究，无明显长期神经认知损害

来源：Sun LS et al. JAMA, 2016.

4. MASK Study (2019) — 明尼苏达州大规模人群研究，显示单次麻醉不会影响 IQ，多次麻醉可能对细微神经行为有影响

来源：Warner DO et al. Anesthesiology, 2019.

结论总结：

“所以，针对一次性、时间不长的全麻手术，尤其是在我们规范的儿童麻醉团队管理下，家长大可不必过度担心。我们也会尽一切努力，保障孩子的麻醉安全和术后恢复。”

English translation (machine translation; for navigation only):

If I were a pediatric urologist and asked parents: "What impact does general anesthesia surgery have on the child?" I would answer this way, trying to be professional, objective, and easy to understand, and cite authoritative evidence:

Doctor’s sample reply:

"This is a very common and concerned question among parents. Generally speaking, current research and clinical experience show that modern general anesthesia technology is very safe in children, especially for one-time, short-term surgeries, such as those commonly performed by our pediatric urology department, which usually do not cause obvious long-term effects on the child's health or intellectual development.

Of course, anesthesia and surgery itself are a 'stressful event', and some reactions may occur in the short term, such as postoperative drowsiness, irritability, nausea, vomiting, and even short-term cognitive and behavioral changes, but most children will recover on their own within a few hours to a few days after surgery.

A lot of research has been done internationally on the issue of ‘whether anesthesia will affect brain development’ that parents are worried about. For example, the reminder issued by the US FDA in 2016 is mainly aimed at children under 3 years old who are exposed to anesthesia for a long time (more than 3 consecutive hours) or multiple times. However, a large number of further clinical studies (GAS study, PANDA study, MASK study) found that there is no clear evidence that one-time, short-term anesthesia will cause intellectual or behavioral problems in children.

Of course, we will also design an individualized anesthesia plan based on the child’s specific situation (age, underlying disease, length of surgery, etc.) to ensure safety. "

Reference source or authoritative basis:

1. FDA Drug Safety Communication (2016) — Safety reminder about the use of narcotic drugs in children and pregnant women

Link: https://www.fda.gov/drugs/drug-safety-and-availability/fda-drug-safety-communication-fda-review-results-suggest-single-short-exposure-general-anesthetic

2. GAS Study (2016, 2019) — a British-Australian multi-center randomized controlled study, which found that a short period of general anesthesia (within 1 hour) had no significant impact on the cognition of children under 2 years old.

Source: Davidson AJ et al. The Lancet, 2016; British Journal of Anaesthesia, 2019.

3. PANDA Study (2016) — A multi-center sibling comparison study in the United States, no obvious long-term neurocognitive impairment

Source: Sun LS et al. JAMA, 2016.

4. MASK Study (2019) — A large-scale population study in Minnesota, showing that a single anesthesia does not affect IQ, but multiple anesthesia may have an impact on subtle neurological behaviors

Source: Warner DO et al. Anesthesiology, 2019.

Conclusion summary:

"Therefore, parents do not have to worry too much about one-time, short-term general anesthesia surgeries, especially under the management of our standardized children's anesthesia team. We will also make every effort to ensure their children's anesthesia safety and postoperative recovery."

AI3 (post-hoc identity: OpenEvidence)

Chinese original (verbatim output):

全身麻醉对儿童的影响是一个备受关注的话题。根据美国儿科外科协会（APSA）的系统综述，单次短时间的全身麻醉对儿童的一般神经发育没有显著影响。然而，长时间或多次麻醉暴露可能会对神经发育产生一些不利影响。此外，麻醉暴露可能在不同年龄段对不同的发育领域产生影响。[1]

一些研究表明，重复或长时间使用全身麻醉和镇静药物可能会影响儿童的大脑发育。美国食品药品监督管理局（FDA）也发布了相关警告，指出在 3 岁以下儿童或孕妇的第三孕期内，重复或长时间使用全身麻醉和镇静药物可能会影响儿童的大脑发育。[2]

尽管动物研究显示麻醉药物可能导致神经元细胞死亡和行为改变，但人类研究的结果并不一致。大多数临床研究是回顾性的，且许多研究未能检测到小的神经认知缺陷。一些研究发现，单次短时间的麻醉暴露与神经发育障碍无关。[3-4]

总的来说，现有证据表明，单次短时间的全身麻醉对儿童的长期神经发育风险较低，但多次或长时间的麻醉暴露可能会增加风险。因此，在计划手术时，应尽量减少麻醉暴露的次数和时间，并在可能的情况下考虑使用区域麻醉技术。[1][4]

1.The Effects of Early Anesthesia on Neurodevelopment: A Systematic Review.Grabowski J, Goldin A, Arthur LG, et al.Journal of Pediatric Surgery. 2021;56(5):851-861. doi:10.1016/j.jpedsurg.2021.01.002.

2.Clinical Update Regarding General Anesthesia-Associated Neurotoxicity in Infants and Children.

Graham MR.Current Opinion in Anaesthesiology. 2017;30(6):682-687. doi:10.1097/ACO.0000000000000520.

3.Does General Anesthesia Affect Neurodevelopment in Infants and Children?.McCann ME, Soriano SG.BMJ (Clinical Research Ed.). 2019;367:l6459. doi:10.1136/bmj.l6459. Leading Journal

4.Neurotoxicity of General Anesthetics in Children: Evidence and Uncertainties.Bellinger DC, Calderon J.Current Opinion in Pediatrics. 2019;31(2):267-273. doi:10.1097/MOP.0000000000000737.

English translation (machine translation; for navigation only):

The effects of general anesthesia on children is a topic of great concern. According to a systematic review by the American Pediatric Surgical Association (APSA), a single short session of general anesthesia has no significant impact on general neurodevelopment in children. However, prolonged or multiple anesthesia exposures may have some adverse effects on neurodevelopment. Furthermore, anesthesia exposure may have effects on different developmental domains at different ages. [1]

Some research suggests that repeated or prolonged use of general anesthesia and sedative drugs may affect children's brain development. The U.S. Food and Drug Administration (FDA) has also issued a relevant warning stating that repeated or prolonged use of general anesthesia and sedation drugs in children under 3 years of age or in pregnant women during their third trimester may affect the child's brain development. [2]

Although animal studies show that anesthetic drugs may cause neuronal cell death and behavioral changes, results from human studies are inconsistent. Most clinical studies are retrospective, and many fail to detect small neurocognitive deficits. Some studies have found that a single, short exposure to anesthesia is not associated with neurodevelopmental disorders. [3-4]

Overall, available evidence suggests that long-term neurodevelopmental risks in children are low from a single short dose of general anesthesia, but that multiple or prolonged anesthesia exposures may increase the risk. Therefore, when planning surgery, the number and duration of anesthetic exposure should be minimized and the use of regional anesthesia techniques considered when possible. [1][4]

1.The Effects of Early Anesthesia on Neurodevelopment: A Systematic Review.Grabowski J, Goldin A, Arthur LG, et al.Journal of Pediatric Surgery. 2021;56(5):851-861. doi:10.1016/j.jpedsurg.2021.01.002.

2.Clinical Update Regarding General Anesthesia-Associated Neurotoxicity in Infants and Children.

Graham MR.Current Opinion in Anaesthesiology. 2017;30(6):682-687. doi:10.1097/ACO.0000000000000520.

3.Does General Anesthesia Affect Neurodevelopment in Infants and Children?.McCann ME, Soriano SG.BMJ (Clinical Research Ed.). 2019;367:l6459. doi:10.1136/bmj.l6459. Leading Journal

4.Neurotoxicity of General Anesthetics in Children: Evidence and Uncertainties.Bellinger DC, Calderon J.Current Opinion in Pediatrics. 2019;31(2):267-273. doi:10.1097/MOP.0000000000000737.

AI4 (post-hoc identity: DeepSeek)

Chinese original (verbatim output):

作为小儿泌尿外科医生，当家长询问全麻手术对患儿的影响时，建议基于现有循证医学证据进行分层次沟通，以下是结构化回答及依据：

核心观点

现代全身麻醉总体是安全的，单次短时麻醉对绝大多数儿童无明显长期影响。但需根据患儿年龄、手术时长和基础疾病综合评估个体风险（*证据等级IA*）。

二、具体影响分层说明

1. 短期影响（术后 24-48 小时）

常见反应（发生率 15-30%）：

- 恶心呕吐（PONV）、咽喉痛（气管插管相关）、定向力短暂混乱

- 来源：Apfel CC et al. Anesthesiology 2012*

罕见风险（<1%）：

- 过敏反应、呼吸抑制（需加强术后监测）

- 来源：ASA Practice Guidelines 2023*

2.神经发育影响（关键争议点）

3 岁以下儿童：

- 单次麻醉<3 小时：GAS 研究（2016/2019）追踪 722 例腹股沟疝患儿至 5 岁，未发现神经认知差异（*Lancet 2019;393:664-677*）

- 反复/长时间麻醉：动物实验显示神经凋亡现象，但临床证据不足（FDA 黑框警告仅针对动物研究）

3 岁以上儿童：PANDA 研究（2016）显示单次麻醉对记忆/智商无影响（*J Neurosurg Anesthesiol 2016*）

3. 特殊风险群体

早产儿：术后呼吸暂停风险增加，需延长监测（*Cote CJ et al. Anesthesiology 1995*）

先天代谢病：如线粒体病需慎用丙泊酚（*相关药物说明书*）

三、权威机构建议

1. 美国儿科学会（AAP）& 麻醉医师协会（ASA）联合声明：

  - 必要手术不应因麻醉顾虑而延迟

  - 建议采用最低有效剂量和最短持续时间

  （Pediatrics 2019;143(1):e20183400）

2. 术前优化措施：

  - 6 月龄以下建议术前血红蛋白筛查

  - 严格执行禁食指南降低误吸风险

  （中华医学会麻醉学分会儿科麻醉指南 2021）

四、沟通要点

量化表述："您孩子接受的是约 1 小时的短时手术，根据国际研究数据，这类手术的麻醉风险与乘坐长途飞机的健康风险相当"

心理安抚：说明麻醉团队会采用儿童专用药物（如七氟烷起效快/恢复快）

决策支持：提供书面版《儿童麻醉知情手册》（可参考上海儿童医学中心 2022 版）

五、延伸建议

术后 3 天内观察异常哭闹/睡眠障碍（多与手术应激相关，非麻醉特异性）

复杂病例推荐转诊至具有儿科麻醉亚专业的医疗中心

通过分层告知风险、引用高质量证据、提供风险对照参照系，可有效缓解家长焦虑并建立治疗联盟。

以下是基于用户提供的搜索结果中关于“全麻手术对患儿影响”的参考出处整理，按内容分类标注引用来源：

1. 全麻的短期影响

恶心、呕吐：与麻醉药物对胃肠道的刺激有关，是术后常见反应。

咽喉痛：气管插管可能导致术后咽喉不适。

嗜睡、头晕：麻醉药物代谢期间可能出现，通常在 24-48 小时内恢复。

呼吸抑制、过敏反应：罕见但需严密监测。

2. 神经发育与认知功能

单次短时麻醉的安全性：

- GAS 研究（2016/2019）表明，单次麻醉（<3 小时）对 3 岁以下儿童无长期神经认知影响。
- PANDA 研究（2016）显示，单次麻醉对 3 岁以上儿童的记忆和智商无显著影响。

潜在争议：

- 动物实验提示反复/长时间麻醉可能引发神经凋亡，但人类临床证据不足。
- 术后短期记忆障碍或注意力下降（数小时至数天），但可完全恢复。

3. 特殊风险群体

早产儿：术后呼吸暂停风险增加，需延长监测时间。

先天代谢疾病患儿：如线粒体病，需谨慎选择麻醉药物（如丙泊酚）。

肝肾功能未成熟：可能延长药物代谢时间，增加术后不适。

4. 权威建议与家长沟通

必要性原则：必要手术不应因麻醉顾虑而延迟。

优化措施：

- 术前禁食以减少误吸风险。
- 选择儿童专用麻醉药物（如七氟烷），确保快速代谢。

心理安抚：术后行为改变（如焦虑、夜惊）多与手术应激相关，非麻醉特异性。

5. 常见误区澄清

智力影响：现代麻醉药物代谢快，无证据表明单次麻醉损害智力。

成瘾风险：临床规范用药不会导致药物依赖。

麻醉深度调控：麻醉医生全程监测，确保用药安全。

主要参考文献来源

临床研究：GAS 研究、PANDA 研究。

指南与共识：美国儿科学会（AAP）与麻醉医师协会（ASA）联合声明、中华医学会儿科麻醉指南。

专家观点：北京积水潭医院万乃君、连云港妇幼保健院陈静、赵华堂医生。

如需查看具体内容，可访问对应来源链接。

English translation (machine translation; for navigation only):

As a pediatric urologist, when parents ask about the impact of general anesthesia surgery on their children, it is recommended to conduct hierarchical communication based on existing evidence-based medical evidence. The following are structured answers and basis:

core ideas

Modern general anesthesia is generally safe, and a single short-term anesthesia has no significant long-term effects on the vast majority of children. However, individual risks need to be comprehensively assessed based on the age of the child, length of surgery, and underlying diseases (*Level of Evidence IA*).

2. Specific impact layering description

1. Short-term effects (24-48 hours after surgery)

Common reactions (incidence 15-30%):

- Nausea and vomiting (PONV), sore throat (related to endotracheal intubation), temporary disorientation

- Source: Apfel CC et al. Anesthesiology 2012*

Rare risk (<1%):

- Allergic reactions, respiratory depression (need to strengthen postoperative monitoring)

- Source: ASA Practice Guidelines 2023*

2. Neurodevelopmental impact (key controversial point)

Children under 3 years old:

- Single anesthesia <3 hours: GAS study (2016/2019) followed 722 children with inguinal hernia to 5 years old and found no neurocognitive differences (*Lancet 2019;393:664-677*)

- Repeated/prolonged anesthesia: Animal experiments show neuroapoptosis, but clinical evidence is insufficient (FDA black box warning only refers to animal studies)

Children over 3 years old: PANDA study (2016) shows that a single anesthesia has no effect on memory/IQ (*J Neurosurg Anesthesiol 2016*)

3. Special risk groups

Premature infants: increased risk of postoperative apnea, requiring extended monitoring (*Cote CJ et al. Anesthesiology 1995*)

Inborn errors of metabolism: If you have mitochondrial disease, propofol should be used with caution (*Related drug instructions*)

3. Recommendations from authoritative organizations

1. Joint statement from the American Academy of Pediatrics (AAP) & Society of Anesthesiologists (ASA):

- Necessary surgery should not be delayed due to anesthesia concerns

- The lowest effective dose and shortest duration recommended

（Pediatrics 2019;143(1):e20183400）

2. Preoperative optimization measures:

- Preoperative hemoglobin screening is recommended for children under 6 months of age

- Strictly follow fasting guidelines to reduce the risk of aspiration

(Chinese Medical Association Anesthesiology Branch Pediatric Anesthesia Guidelines 2021)

4. Key points of communication

Quantitative statement: "Your child received a short surgery of about one hour. According to international research data, the anesthesia risks of this type of surgery are equivalent to the health risks of taking a long-distance flight."

Psychological comfort: Explain that the anesthesia team will use special drugs for children (such as sevoflurane for quick onset/fast recovery)

Decision support: Provide a written version of the "Children's Anesthesia Information Manual" (please refer to the 2022 version of Shanghai Children's Medical Center)

5. Extended suggestions

Observe abnormal crying/sleep disturbance within 3 days after surgery (mostly related to surgical stress and not specific to anesthesia)

Referral of complex cases to a medical center with subspecialty in pediatric anesthesia is recommended

By informing risks in layers, citing high-quality evidence, and providing a risk comparison frame of reference, parents' anxiety can be effectively alleviated and a therapeutic alliance established.

The following is a compilation of reference sources on "The impact of general anesthesia surgery on children" based on the search results provided by users. The sources of citations are marked by content classification:

1. Short-term effects of general anesthesia

Nausea and vomiting: related to the stimulation of the gastrointestinal tract by anesthetic drugs, are common postoperative reactions.

Sore throat: Endotracheal intubation may cause postoperative throat discomfort.

Drowsiness and dizziness: may occur during the metabolism of anesthetic drugs and usually recover within 24-48 hours.

Respiratory depression, allergic reactions: rare but require close monitoring.

2. Neurodevelopment and cognitive function

Safety of a single short-term anesthesia:

- The GAS study (2016/2019) showed that a single dose of anesthesia (<3 hours) had no long-term neurocognitive effects in children under 3 years of age.

- The PANDA study (2016) shows that a single dose of anesthesia has no significant impact on the memory and IQ of children over 3 years old.

Potential disputes:

- Animal experiments suggest that repeated/long-term anesthesia may induce neural apoptosis, but there is insufficient clinical evidence in humans.

- Postoperative short-term memory impairment or decreased concentration (hours to days), but full recovery possible.

3. Special risk groups

Premature infants: The risk of postoperative apnea is increased and the monitoring time needs to be extended.

Children with inborn metabolic diseases: such as mitochondrial disease, need to choose anesthetic drugs (such as propofol) carefully.

Immature liver and kidney functions: may prolong drug metabolism and increase postoperative discomfort.

4. Authoritative advice to communicate with parents

Principle of necessity: Necessary surgery should not be delayed due to anesthesia concerns.

Optimization measures:

- Fasting before surgery to reduce the risk of aspiration.

- Choose an anesthetic drug specifically designed for children (such as sevoflurane) to ensure rapid metabolism.

Psychological comfort: Postoperative behavioral changes (such as anxiety, night terrors) are mostly related to surgical stress and are not anesthesia-specific.

5. Clarification of common misunderstandings

Influence on intelligence: Modern anesthetic drugs are metabolized quickly, and there is no evidence that a single dose of anesthesia damages intelligence.

Addiction risk: Clinically standardized medication will not lead to drug dependence.

Anesthesia depth control: The anesthesiologist monitors the whole process to ensure the safety of medication.

Main reference sources

Clinical research: GAS research, PANDA research.

Guidelines and consensus: Joint statement by the American Academy of Pediatrics (AAP) and the Society of Anesthesiologists (ASA), Chinese Medical Association Pediatric Anesthesia Guidelines.

Expert opinions: Wan Naijun from Beijing Jishuitan Hospital, Chen Jing and Zhao Huatang from Lianyungang Maternal and Child Health Hospital.

If you want to view specific content, please visit the corresponding source link.

AI5 (post-hoc identity: Gemini-2.5-Pro)

Chinese original (verbatim output):

好的，家长您好。您担心全麻对孩子的影响，这是非常可以理解的，也是我们医生非常重视的问题。作为小儿泌尿外科医生，我会和我们经验丰富的儿科麻醉医生团队紧密合作，确保您孩子在手术过程中的安全和舒适。

关于全麻对患儿的影响，我们可以从短期和长期两个方面来看：

1. 短期影响（常见且通常是暂时的）：

* 术后苏醒期反应：孩子刚从麻醉中醒来时，可能会有些迷糊、嗜睡，或者相反地，有些烦躁、哭闹、不安。这与麻醉药物的残留效应以及陌生环境有关，通常在麻醉药物完全代谢后会逐渐缓解。

* 恶心与呕吐：这是麻醉后比较常见的反应之一，现代麻醉药物和预防措施已经可以显著降低其发生率和严重程度。如果发生，我们有药物可以处理。

* 喉咙痛或声音嘶哑：如果手术中需要放置气管插管来辅助呼吸，孩子醒来后可能会感觉喉咙有些不适或声音嘶哑，一般几天内会自行恢复。

* 头晕、寒战： 部分孩子在苏醒过程中可能会感到头晕或身体发冷颤抖，我们会进行保暖和对症处理。

* 疼痛：手术切口本身会引起疼痛，麻醉医生和我们外科医生会使用多种镇痛方法（如切口局部麻醉、术后镇痛泵、口服或静脉镇痛药）来最大程度地减轻孩子的痛苦。

这些短期影响大多是暂时的，并且医护人员会在术后密切观察和处理，确保孩子平稳度过恢复期。

2. 长期影响（广受关注，但目前证据指向特定情况）：

* 对神经发育和认知功能的影响： 这是家长们最关心的问题。过去十几年中，医学界对此进行了大量的研究。

  * 目前的共识是： 对于单次、短时间（通常指少于 3 小时）的全身麻醉，在健康的婴幼儿和儿童中，现有的大规模临床研究证据并未明确显示会对长期的智力、学习能力、记忆力或行为发育造成显著的、持续性的负面影响。

  * 需要考虑的因素：

    * 麻醉次数和时长：多次或长时间（例如，远超 3 小时）的麻醉暴露，其潜在影响仍在研究中，部分研究提示可能需要更谨慎的评估，但仍缺乏确凿的因果关系证据。

* 患儿年龄：尤其关注 3 岁以下婴幼儿的麻醉暴露，因为这是大脑快速发育的关键时期。但即使在这个年龄段，单次短时麻醉的长期风险也未被证实。

* 患儿自身健康状况： 存在严重基础疾病（如先天性心脏病、神经系统疾病等）的儿童，麻醉风险评估会更复杂，需要综合考虑。

* 手术本身： 手术的类型、复杂程度以及手术要解决的疾病本身，都可能对孩子的恢复和长期健康产生影响，有时这些影响比麻醉本身更大。

权衡利弊至关重要：

我们需要认识到，进行手术是为了治疗或纠正一个影响孩子健康和生活质量的泌尿系统问题。如果不进行手术，疾病本身可能对孩子的肾脏功能、身体发育甚至心理健康造成更确切、更严重的长期损害。因此，在决定手术时，我们是在权衡手术治疗带来的明确益处与麻醉/手术相关的（目前看来对于单次短时麻醉而言）尚不明确或风险极低的潜在长期影响。

我们的做法：

* 专业的儿科麻醉团队：我们医院拥有经验丰富的儿科麻醉医生，他们接受过专门针对儿童生理特点和麻醉需求的培训。

* 个体化评估： 麻醉医生会在术前详细评估您孩子的具体情况（年龄、体重、健康状况、手术类型等），选择最合适的麻醉药物和剂量。

* 严密监护： 手术全程，麻醉医生会使用先进的监护设备，持续监测孩子的生命体征（心率、血压、呼吸、血氧饱和度等），确保麻醉深度适宜且生命体征平稳。

* 以安全为首要原则：整个医疗团队的目标都是在确保最大安全的前提下，顺利完成手术，解决孩子的病痛。

总结来说，现代小儿麻醉技术已经非常成熟和安全。对于您孩子即将接受的单次泌尿外科手术，全麻的短期影响是可控、可恢复的，而长期神经发育风险根据现有权威研究证据来看，是非常低的。我们会采取一切必要的预防措施，将风险降至最低。

请您放心，手术前麻醉医生会再次与您详细沟通麻醉计划和可能的风险，您可以将所有疑虑直接向他们提出。我们整个团队都会全力以赴，确保您孩子的安全。

来源依据与参考出处（供专业人士参考或家长深入了解）：

1. SmartTots Initiative (由国际麻醉研究学会 IARS 和美国食品药品监督管理局 FDA 合作)：这是一个专注于儿童麻醉安全性和对大脑发育影响的研究项目。其网站 (www.smarttots.org) 提供了大量面向家长和专业人士的信息和研究更新。他们目前的结论倾向于单次短时麻醉对健康儿童的长期认知功能影响风险很小。

2. 美国麻醉医师协会 (ASA) 和儿科麻醉学会 (SPA) 的声明和指南： 这些专业组织会定期发布关于小儿麻醉安全的声明，通常会反映最新的研究进展和临床共识。

3. 相关大型临床研究：

 * PANDA Study (Pediatric Anesthesia Neurodevelopment Assessment): 发表在 *JAMA* 等期刊，比较了幼儿期接受单次全麻的兄弟姐妹与其未接受麻醉的兄弟姐妹，未发现 IQ 或神经认知功能的显著差异。

 * GAS Study (General Anaesthesia compared to Spinal anaesthesia): 发表在 *The Lancet*，随机比较了婴儿期接受全麻与区域麻醉（脊麻）进行腹股沟疝修补术的儿童，在 5 岁时评估神经发育结局，未发现两组间有显著差异。

 * MASK Study (Mayo Anesthesia Safety in Kids): 发表在 *Anesthesiology*，回顾性研究，发现在 3 岁前接受单次或多次麻醉暴露与学习障碍风险增加可能相关，但强调需要更多前瞻性研究来确定因果关系，且单次暴露的风险尚不明确。

4. 权威医学期刊的综述文章： 如 *Anesthesiology*, *Pediatric Anesthesia*, *The New England Journal of Medicine*, *The Lancet* 等期刊上常有关于此主题的最新综述和评论。

希望这些信息能解答您的疑问，减轻您的顾虑。如果您还有其他问题，请随时提出。

评价指标及定义

请您从文本质量、相关性、适用性、来源可靠性、可理解性、可操作性 6 个方面进行评价，排在越前面意味着其在各指标综合考量下分值越高。

1.质量：衡量 AI 答案对医疗疑问的解决效果，涵盖医学准确性、细节完整性以及提供医学见解的深度，判断其能否为提问者提供有价值、全面且深入的医学指导。

请将 5 个 AI 回答文本按照质量评价进行排序，例如 AI1＞AI2＞AI3＞AI4＞AI5 。

您的作答： ＞ ＞ ＞ ＞

2.相关性：判断 AI 答案与医学问题的契合程度，包括是否紧扣主题，是否全面涉及医学问题的各个方面，以及所提供信息与医疗问题的关联紧密性。

请将 5 个 AI 回答文本按照相关性进行排序，例如 AI1＞AI2＞AI3＞AI4＞AI5 。

您的作答： ＞ ＞ ＞ ＞

3.适用性：评估 AI 答案在询问所描述的医学背景和实际医疗场景中的可应用程度，看其是否能直接用于解决实际问题，或是需要大量调整修改。

请将 5 个 AI 回答文本按照适用性进行排序，例如 AI1＞AI2＞AI3＞AI4＞AI5 。

您的作答： ＞ ＞ ＞ ＞

4.来源可靠性：考查 AI 答案所依据来源的可信度（是否有虚假信息或来源不详细等），包括参考文献的权威性、输出内容与文献的关联度，以及信息来源渠道是否专业、经过严格审核，以此判断答案是否准确、真实且无过时信息。

请将 5 个 AI 回答文本按照来源可靠性进行排序，例如 AI1＞AI2＞AI3＞AI4＞AI5 。

您的作答：　　＞　　＞　　＞　　＞

5.可理解性：评价 AI 答案在语言使用、内容组织以及辅助工具运用等方面，是否便于用户理解，包括语言是否通俗易懂、术语有无定义、结构是否清晰、有无视觉辅助或摘要帮助理解等。

请将 5 个 AI 回答文本按照可理解性进行排序，例如 AI1＞AI2＞AI3＞AI4＞AI5 。

您的作答：　　＞　　＞　　＞　　＞

6.可操作性：判断 AI 答案是否明确指出用户可采取的操作，是否将操作细化为清晰步骤，以及是否提供实用工具辅助用户在实际场景中采取有效行动。

请将 5 个 AI 回答文本按照可操作性进行排序，例如 AI1＞AI2＞AI3＞AI4＞AI5 。

您的作答：　　＞　　＞　　＞　　＞

根据以上内容进行综合排序：：　　＞　　＞　　＞　　＞

English translation (machine translation; for navigation only):

Okay, hello parents. It is very understandable that you are worried about the impact of general anesthesia on your child, and it is also an issue that our doctors take very seriously. As a pediatric urologist, I work closely with our team of experienced pediatric anesthesiologists to ensure your child's safety and comfort during the procedure.

Regarding the impact of general anesthesia on children, we can look at it from two aspects: short-term and long-term:

1. Short-term effects (common and usually temporary):

* Postoperative recovery period reaction: When the child just wakes up from anesthesia, he may be a little confused and sleepy, or conversely, he may be a little irritable, crying, and restless. This is related to the residual effects of the anesthetic drugs and the unfamiliar environment, which usually will gradually ease after the anesthetic drugs are completely metabolized.

* Nausea and vomiting: This is one of the more common reactions after anesthesia. Modern anesthetic drugs and preventive measures can significantly reduce its incidence and severity. If it happens, we have medicine to deal with it.

* Sore throat or hoarseness: If an endotracheal intubation is required to assist breathing during surgery, the child may feel some discomfort in the throat or hoarseness after waking up, which will usually resolve on its own within a few days.

* Dizziness and chills: Some children may feel dizzy or have chills and tremors during the recovery process. We will keep them warm and treat them symptomatically.

* Pain: The surgical incision itself can cause pain, and the anesthesiologist and our surgeons will use a variety of analgesic methods (such as local anesthesia for the incision, postoperative pain pumps, oral or intravenous analgesics) to minimize your child's pain.

Most of these short-term effects are temporary, and medical staff will observe and handle them closely after surgery to ensure that the child goes through the recovery period smoothly.

2. Long-term effects (widely concerned, but current evidence points to specific circumstances):

* Effects on neurodevelopment and cognitive function: This is the biggest concern for parents. In the past decade or so, the medical community has conducted a lot of research on this.

* The current consensus is that: For single, short-term (usually less than 3 hours) general anesthesia, the existing large-scale clinical research evidence in healthy infants and children does not clearly show that it will cause significant and sustained negative effects on long-term intelligence, learning ability, memory or behavioral development.

* Factors to consider:

*Number and duration of anesthesia: The potential effects of multiple or prolonged anesthesia exposures (e.g., well over 3 hours) are still being studied, and some studies suggest that more careful evaluation may be needed, but conclusive evidence of causality is still lacking.

*Age of child: Particular attention is paid to anesthesia exposure in infants and young children under 3 years of age, as this is a critical period for rapid brain development. But even in this age group, the long-term risks of a single short dose of anesthesia have not been proven.

* Children's own health status: For children with serious underlying diseases (such as congenital heart disease, neurological diseases, etc.), anesthesia risk assessment will be more complex and needs to be comprehensively considered.

* The surgery itself: The type, complexity, and condition of the surgery itself can all have an impact on your child's recovery and long-term health, sometimes more than the anesthesia itself.

It’s important to weigh the pros and cons:

We need to realize that surgery is performed to treat or correct a urinary system problem that affects the child's health and quality of life. Without surgery, the disease itself may cause more precise and severe long-term damage to the child's kidney function, physical development, and even mental health. Therefore, when deciding to operate, we are weighing the clear benefits of surgical treatment against the potential long-term effects associated with anesthesia/surgery that are (at this time, seemingly for a single short-duration anesthesia) unclear or of very low risk.

Our approach:

* Professional pediatric anesthesia team: Our hospital has experienced pediatric anesthesiologists who have received special training based on children's physiological characteristics and anesthesia needs.

* Individualized assessment: The anesthesiologist will evaluate your child's specific situation (age, weight, health status, type of surgery, etc.) in detail before surgery and select the most appropriate anesthetic drug and dosage.

* Close monitoring: During the entire operation, the anesthesiologist will use advanced monitoring equipment to continuously monitor the child's vital signs (heart rate, blood pressure, respiration, blood oxygen saturation, etc.) to ensure that the depth of anesthesia is appropriate and the vital signs are stable.

* Safety as the first principle: The goal of the entire medical team is to successfully complete the operation and solve the child's pain while ensuring maximum safety.

In summary, modern pediatric anesthesia technology is very mature and safe. For the single urological surgery your child is about to undergo, the short-term effects of general anesthesia are controllable and recoverable, while the long-term neurodevelopmental risks are very low based on available authoritative research evidence. We will take all necessary precautions to minimize risk.

Please rest assured that the anesthesiologist will communicate with you in detail about the anesthesia plan and possible risks again before the operation, and you can raise all concerns directly with them. Our entire team is dedicated to ensuring the safety of your child.

Source basis and reference source (for professional reference or parents’ in-depth understanding):

1. SmartTots Initiative (a collaboration between the International Anesthesia Research Society IARS and the U.S. Food and Drug Administration FDA): This is a research project focused on the safety of anesthesia in children and its impact on brain development. Its website (www.smarttots.org) provides a wealth of information and research updates for parents and professionals. Their current conclusion favors that a single short dose of anesthesia poses little risk to long-term cognitive function in healthy children.

2. Statements and Guidelines from the American Society of Anesthesiologists (ASA) and the Society of Pediatric Anesthesia (SPA): These professional organizations regularly issue statements on pediatric anesthesia safety, often reflecting the latest research advances and clinical consensus.

3. Relevant large-scale clinical studies:

* PANDA Study (Pediatric Anesthesia Neurodevelopment Assessment): Published in *JAMA* and other journals, compared siblings who received a single dose of general anesthesia in early childhood with siblings who did not receive anesthesia and found no significant differences in IQ or neurocognitive function.

* GAS Study (General Anaesthesia compared to Spinal anaesthesia): Published in *The Lancet*, randomized comparison of children who received general anesthesia versus regional anesthesia (spinal anesthesia) for inguinal hernia repair in infancy. Neurodevelopmental outcomes were assessed at 5 years of age and no significant differences were found between the two groups.

* MASK Study (Mayo Anesthesia Safety in Kids): Published in *Anesthesiology*, a retrospective study found that single or multiple anesthesia exposures before age 3 may be associated with an increased risk of learning disabilities, but emphasized that more prospective studies are needed to determine cause and effect, and that the risk of a single exposure is unclear.

4. Review articles in authoritative medical journals: Journals such as *Anesthesiology*, *Pediatric Anesthesia*, *The New England Journal of Medicine*, *The Lancet* and other journals often contain the latest reviews and comments on this topic.

Hopefully this information will answer your questions and alleviate your concerns. If you have further questions, please feel free to ask.

Evaluation indicators and definitions

Please evaluate it from six aspects: text quality, relevance, applicability, source reliability, understandability, and operability. The higher the ranking, the higher the score based on the comprehensive consideration of each indicator.

1. Quality: Measure the effectiveness of AI answers in solving medical questions, covering medical accuracy, completeness of details and the depth of medical insights provided, to determine whether it can provide valuable, comprehensive and in-depth medical guidance to the questioner.

Please sort the 5 AI answer texts according to quality evaluation, for example, AI1>AI2>AI3>AI4>AI5.

Your answer: ＞ ＞ ＞ ＞

2. Relevance: Determine the degree of fit between the AI answer and the medical problem, including whether it sticks to the topic, whether it comprehensively covers all aspects of the medical problem, and how closely the information provided is related to the medical problem.

Please sort the 5 AI answer texts according to their relevance, for example, AI1>AI2>AI3>AI4>AI5.

Your answer: ＞ ＞ ＞ ＞

3. Applicability: Evaluate the applicability of the AI answers in the medical background and actual medical scenarios described in the query to see whether it can be directly used to solve practical problems, or whether it requires extensive adjustments and modifications.

Please sort the 5 AI answer texts according to their applicability, for example, AI1>AI2>AI3>AI4>AI5.

Your answer: ＞ ＞ ＞ ＞

4. Source reliability: Examine the credibility of the sources on which the AI answers are based (whether there is false information or unclear sources, etc.), including the authority of the references, the relevance of the output content and the literature, and whether the information source channels are professional and rigorously reviewed to determine whether the answers are accurate, authentic and free of outdated information.

Please sort the 5 AI answer texts according to source reliability, for example, AI1>AI2>AI3>AI4>AI5.

Your answer: ＞ ＞ ＞ ＞

5. Comprehensibility: Evaluate whether the AI answers are easy for users to understand in terms of language use, content organization, and use of auxiliary tools, including whether the language is easy to understand, whether terms are defined, whether the structure is clear, and whether there are visual aids or summaries to help understanding, etc.

Please sort the 5 AI answer texts according to their comprehensibility, for example, AI1>AI2>AI3>AI4>AI5.

Your answer: ＞ ＞ ＞ ＞

6. Operability: Determine whether the AI answer clearly points out the actions that the user can take, whether the operation is refined into clear steps, and whether it provides practical tools to assist the user in taking effective actions in actual scenarios.

Please sort the 5 AI answer texts according to operability, for example, AI1>AI2>AI3>AI4>AI5.

Your answer: ＞ ＞ ＞ ＞

Comprehensive sorting based on the above content:: ＞ ＞ ＞ ＞

Question 4 (Low risk; Postoperative Care)

Original prompt (Chinese):

如果你是一名小儿泌尿外科的医生，家长问到"尿道下裂患儿术后一般需要多长时间安全康复？"问题，你应该怎么回答？并给出来源依据或参考出处。

Prompt translation (English):

If you are a pediatric urologist, and a parent asks, "How long does a child generally need for safe recovery after hypospadias surgery?", how should you answer? Please cite your sources or references.

Per-question unblinding key for Q4:

Blinded label	Model identity
AI1	Zhipu Qingyan
AI2	DeepSeek
AI3	ChatGPT-4o
AI4	Gemini-2.5-Pro
AI5	OpenEvidence

AI1 (post-hoc identity: Zhipu Qingyan)

Chinese original (verbatim output):

1.问题背景

尿道下裂是一种常见的先天性泌尿系统畸形，需要通过手术进行矫正。

家长关心患儿术后安全康复所需的时间。

2.术后康复时间

住院观察期：通常为 3 到 7 天，以便医生监测患儿的术后状况。

家中康复期：可能需要几周到几个月的时间，具体取决于个体差异、手术方式及手术复杂程度。

3.术后护理措施

疼痛管理：医生会开具止痛药物以缓解术后疼痛和不适。

伤口护理：保持伤口清洁和干燥，避免感染和出血。

活动限制：避免剧烈活动，减少对伤口的刺激。

复查：术后几周内需回医院复查，确保伤口愈合和尿道功能恢复。

4.多次手术的可能性

尿道下裂的手术矫正可能需要多次进行，以达到理想效果。

家长需有耐心，并与医生保持密切沟通，及时了解患儿恢复情况。

5.结论

尿道下裂患儿的术后康复是一个渐进过程，需要家长和医生的共同努力。

通过合理的治疗和护理，大多数患儿能够安全康复。

参考出处：

《小儿外科学》（人民卫生出版社）

UpToDate 临床顾问：尿道下裂的治疗和管理

National Institutes of Health: Hypospadias

English translation (machine translation; for navigation only):

1. Problem background

Hypospadias is a common congenital urinary system malformation that requires surgical correction.

Parents are concerned about the time it takes for their children to recover safely after surgery.

2. Postoperative recovery time

Hospitalization observation period: usually 3 to 7 days to allow doctors to monitor the child's postoperative condition.

Recovery at home: This may take anywhere from a few weeks to a few months, depending on individual differences, the type of surgery, and the complexity of the surgery.

3. Postoperative care measures

Pain management: Your doctor will prescribe pain medications to relieve pain and discomfort after surgery.

Wound care: Keep wounds clean and dry to avoid infection and bleeding.

Activity restrictions: Avoid strenuous activities to reduce irritation to the wound.

Review: You need to return to the hospital for review within a few weeks after the operation to ensure wound healing and recovery of urethral function.

4. Possibility of multiple surgeries

Surgical correction of hypospadias may require multiple procedures to achieve optimal results.

Parents need to be patient and maintain close communication with doctors to keep abreast of their children's recovery status.

5.Conclusion

The postoperative rehabilitation of children with hypospadias is a gradual process that requires the joint efforts of parents and doctors.

With appropriate treatment and care, most children can recover safely.

Reference source:

"Pediatric Surgery" (People's Medical Publishing House)

UpToDate Clinical Advisor: Treatment and Management of Hypospadias

National Institutes of Health: Hypospadias

AI2 (post-hoc identity: DeepSeek)

Chinese original (verbatim output):

作为小儿泌尿外科医生，针对“尿道下裂术后康复时间”的问题，以下是根据专业指南和研究的回答：

回答要点

1. 初步恢复（1-2 周）

伤口愈合：术后 1-2 周内切口初步愈合，需避免剧烈活动，防止感染。

导尿管留置：通常需留置导尿管 5-14 天（根据手术类型，如 Snodgrass 术式可能需 7-14 天），以支撑尿道成形并减少尿瘘风险。

2. 功能恢复（2-4 周）

拔管后观察：拔除导尿管后需密切观察排尿情况，确保无尿线细、尿瘘或狭窄。

活动限制：术后 3-4 周内避免骑跨运动（如骑车）以防伤口裂开。

3. 完全康复（3-6 个月）

瘢痕稳定：术后 3-6 个月瘢痕逐渐软化，阴茎外观趋于自然。

并发症监测：尿瘘、尿道狭窄等并发症多发生于术后 6 个月内，需定期随访。

4. 个体差异与并发症

复杂病例（如阴囊型尿道下裂）或出现并发症（如感染、尿瘘）时，恢复时间可能延长，需二次手术干预。

依据与参考文献

1. 美国泌尿外科协会（AUA）指南

建议术后导尿管留置时间为5-14天，具体取决于术式（如Snodgrass术式推荐7-10天）。

强调术后3-6个月内密切随访以监测并发症。

来源：*AUA Guidelines on Hypospadias Management (2020)*。

2. 欧洲小儿泌尿外科学会（ESPU）共识

指出大多数患儿术后2-4周可恢复正常日常活动，但完全愈合需3个月以上。

来源：*ESPU Consensus Statement on Hypospadias Repair (2018)*。

3. 经典文献支持

Snodgrass等人的研究显示，TIP（尿道板纵切卷管）术后导尿管平均留置时间为7天，尿瘘发生率低于10%。

来源：*Snodgrass W, et al. J Urol. 1996;156(2 Pt 2):858-860.*

4. 教科书参考

《Campbell-Walsh Urology》指出，尿道下裂术后需至少6个月评估最终效果，因瘢痕重塑期较长。

来源：*Wein AJ, et al. Campbell-Walsh Urology, 12th Edition. 2020.*

家长沟通建议

个性化说明：根据患儿的手术类型（如远端或近端尿道下裂）和术中情况调整康复预期。

术后护理重点：强调保持伤口清洁、避免压迫、按时复诊。

心理支持：鼓励家长耐心等待瘢痕自然软化，避免过早焦虑外观问题。

若家长需要更具体的时间规划，建议结合患儿的个体情况（如年龄、手术复杂性）进行详细解释。

English translation (machine translation; for navigation only):

As a pediatric urologist, in response to the question "recovery time after hypospadias surgery", here are the answers based on professional guidelines and research:

Answer points

1. Initial recovery (1-2 weeks)

Wound healing: The incision will initially heal within 1-2 weeks after the operation. Strenuous activities need to be avoided to prevent infection.

Urethral catheter indwelling: A urinary catheter is usually left in place for 5-14 days (depending on the type of surgery, such as Snodgrass surgery, it may take 7-14 days) to support the urethroplasty and reduce the risk of urinary fistula.

2. Functional recovery (2-4 weeks)

Observation after removal of the catheter: After removal of the urinary catheter, the urination situation needs to be closely observed to ensure that there is no thin urinary line, urinary fistula or stricture.

Activity restrictions: Avoid straddle sports (such as cycling) for 3-4 weeks after surgery to prevent wound dehiscence.

3. Full recovery (3-6 months)

Scar stabilization: Scars gradually soften 3-6 months after surgery, and the appearance of the penis becomes more natural.

Complication monitoring: Complications such as urinary fistula and urethral stricture mostly occur within 6 months after surgery and require regular follow-up.

4. Individual differences and complications

In complex cases (such as scrotal hypospadias) or complications (such as infection, urinary fistula), the recovery time may be prolonged and a second surgical intervention is required.

Basis and references

1. American Urological Association (AUA) Guidelines

The recommended post-operative catheter indwelling time is 5-14 days, depending on the surgical procedure (for example, Snodgrass surgery recommends 7-10 days).

Emphasize close follow-up within 3-6 months after surgery to monitor complications.

Source: *AUA Guidelines on Hypospadias Management (2020)*.

2. European Society of Pediatric Urology (ESPU) consensus

It is pointed out that most children can return to normal daily activities 2-4 weeks after surgery, but complete healing takes more than 3 months.

Source: *ESPU Consensus Statement on Hypospadias Repair (2018)*.

3. Classic literature support

The study by Snodgrass et al showed that the average indwelling time of urinary catheter after TIP (urethral plate incision and rolling tube) was 7 days, and the incidence of urinary fistula was less than 10%.

Source: *Snodgrass W, et al. J Urol. 1996;156(2 Pt 2):858-860.*

4. Textbook Reference

"Campbell-Walsh Urology" points out that it takes at least 6 months to evaluate the final effect after hypospadias surgery because the scar remodeling period is longer.

Source: *Wein AJ, et al. Campbell-Walsh Urology, 12th Edition. 2020.*

Parent communication suggestions

Personalized instructions: Adjust recovery expectations based on the child's type of surgery (eg, distal or proximal hypospadias) and intraoperative circumstances.

Key points of postoperative care: Emphasis on keeping the wound clean, avoiding compression, and returning to the hospital on time.

Psychological support: Parents are encouraged to wait patiently for scars to soften naturally to avoid premature anxiety about appearance problems.

If parents need more specific time planning, it is recommended to provide a detailed explanation based on the child's individual situation (such as age, complexity of surgery).

AI3 (post-hoc identity: ChatGPT-4o)

Chinese original (verbatim output):

作为小儿泌尿外科医生，针对家长提出的"尿道下裂患儿术后一般需要多长时间安全康复？"这个问题，可以这样专业而温和地回答：

医生回答建议（临床沟通版）：

"尿道下裂手术后的康复时间会根据手术类型、下裂的严重程度、是否合并其他问题，以及孩子的个体恢复情况而有所不同。

一般来说，术后短期恢复（指尿管拔除、尿路功能恢复、无明显感染或并发症）大约需要 24 周。

而完全恢复到比较安全、稳定的状态，包括尿道愈合良好、排尿功能正常、无明显瘢痕或狭窄风险，通常需要 36 个月。

当然，在这期间需要定期门诊复查，特别是术后 3 个月和 6 个月是关键的评估节点。

对于部分复杂或再次手术的患儿，恢复期可能会更长一些。我们会根据孩子的具体情况为您制定个体化的康复和随访方案。"

推荐参考文献或指南依据：

1. 《Campbell-Walsh-Wein Urology》第 12 版（小儿泌尿外科章节）

提到尿道下裂术后恢复通常需要 24 周拔除尿管，36 个月作为术后并发症监测窗口期。

2. ESPU (European Society for Paediatric Urology) Guidelines 2023

建议术后随访至少持续至 6 个月，评估排尿、尿道愈合及远期并发症。

3. 中国《小儿尿道下裂诊治专家共识（2022）》

强调术后早期（2~4 周）注意导尿及抗感染管理，中期（3 个月内）评估排尿功能，远期（6 个月及以上）监测尿道瘢痕、狭窄等。

小结

阶段	时间	康复目标	临床处理
短期恢复	2~4 周	拔除导尿管，无感染，排尿顺畅	门诊复查，抗感染，护理指导
中期恢复	3 个月	排尿功能良好，尿道愈合良好	重点评估排尿及尿道情况
远期康复	6 个月及以上	无瘢痕狭窄，无再手术需求	长期随访，根据需要调整

需要我帮你整理成宣传册、宣教单页、家长易懂的版本吗？还是你更需要标准化的医患沟通模板？

English translation (machine translation; for navigation only):

As a pediatric urologist, I can answer the question raised by parents in a professional and gentle way: "How long does it usually take for children with hypospadias to recover safely after surgery?"

Suggestions from doctors (clinical communication version):

“Recovery time after hypospadias surgery will vary based on the type of surgery, the severity of the hypospadias, whether there are other problems involved, and the child’s individual recovery.

Generally speaking, short-term recovery after surgery (referring to removal of the urinary catheter, recovery of urinary tract function, and no obvious infection or complications) takes about 24 weeks.

It usually takes 36 months to completely return to a relatively safe and stable state, including good urethral healing, normal urinary function, and no risk of obvious scarring or stricture.

Of course, regular outpatient review is required during this period, especially 3 months and 6 months after surgery are key evaluation points.

For some children with complex or re-operations, the recovery period may be longer. We will develop an individualized rehabilitation and follow-up plan for you based on your child's specific situation. "

Recommended references or guides based on:

1. "Campbell-Walsh-Wein Urology" 12th Edition (Pediatric Urology Chapter)

It is mentioned that recovery after hypospadias surgery usually requires removal of the urinary catheter at 24 weeks, and 36 months is used as the window period for monitoring postoperative complications.

2. ESPU (European Society for Paediatric Urology) Guidelines 2023

It is recommended that postoperative follow-up be continued for at least 6 months to evaluate urination, urethral healing, and long-term complications.

3. China's "Expert Consensus on the Diagnosis and Treatment of Hypospadias in Children (2022)"

It is emphasized to pay attention to urinary catheterization and anti-infection management in the early postoperative period (2 to 4 weeks), evaluate urinary function in the mid-term period (within 3 months), and monitor urethral scars and strictures in the long-term period (6 months and above).

summary

Stage Time Rehabilitation Goals Clinical Management

Short-term recovery 2 to 4 weeks. Remove the urinary catheter, no infection, and urinate smoothly. Outpatient review, anti-infection, and nursing guidance.

Mid-term recovery: 3 months. Urinary function is good and urethra is healing well. Focus on evaluation of urination and urethra conditions.

Long-term recovery 6 months and above No scar stenosis, no need for re-operation Long-term follow-up, adjustments as needed

Do you need me to help you organize it into a brochure, a leaflet, or a parent-friendly version? Or do you need more standardized doctor-patient communication templates?

AI4 (post-hoc identity: Gemini-2.5-Pro)

Chinese original (verbatim output):

好的，家长您好。您问的这个问题非常好，也是很多家长都非常关心的。“尿道下裂宝宝术后多久能安全康复？”这个问题没有一个绝对统一的答案，因为每个孩子的具体情况不同，恢复时间也会有差异。但我可以给您一个大概的框架和影响恢复时间的因素。

一般来说，尿道下裂术后的恢复可以分为几个阶段：

1. 早期恢复期（带管期和拔管初期，大约 2-3 周）：

   * 住院时间：根据手术复杂程度和孩子恢复情况，通常需要住院几天到一周左右，有些简单的病例甚至可以日间手术完成。

   * 带尿管/支架管时间：这是为了让新修复的尿道能够良好愈合并定型，同时引流尿液，避免尿液直接冲击伤口。这个时间通常是**7 到 14 天**，具体时长会根据手术方式、尿道重建的复杂程度以及医生的习惯来决定。有些复杂情况可能需要更长时间。

   * 伤口初步愈合：拔除尿管后，伤口基本完成表皮愈合，但内部组织仍在修复中。这个阶段需要非常小心护理，避免感染和伤口裂开。

2. 中期恢复期（拔管后至伤口稳定，大约 1-3 个月）：

   * 伤口稳定和疤痕软化： 伤口外观会逐渐改善，红肿消退，疤痕开始软化。但此时组织仍然比较脆弱。

   * 功能初步恢复：孩子开始自主排尿，需要观察尿线形态、有无漏尿（尿瘘）、排尿是否费力（尿道狭窄）等。

   * 活动限制： 在最初的 4-6 周内，需要限制剧烈活动，特别是骑跨动作（如骑自行车、摇马）或可能导致会阴部撞击的活动，以免影响伤口愈合。

3. 长期恢复与随访期（术后 3 个月至数年）：

   * “安全康复”的完整概念： 真正的“安全康复”不仅仅是伤口长好，更重要的是达到满意的外观和功能，并且没有出现远期并发症（如尿瘘、尿道狭窄、尿道憩室、阴茎弯曲复发等）。

   * 定期复查： 术后需要按照医嘱定期复诊（通常是术后 1 个月、3 个月、6 个月、1 年，之后根据情况每年或隔年复查直至青春期发育后），医生会评估排尿情况、阴茎外观、有无弯曲等。有些并发症可能在术后几个月甚至几年后才显现。

   * 最终稳定： 通常认为，术后半年到一年，如果没有出现并发症，可以认为达到了相对稳定的状态。但对外观和功能的最终评价，有时需要等到青春期发育完成后。

影响恢复时间的主要因素：

* 尿道下裂的严重程度：尿道开口越靠近阴囊或会阴部（重型），手术越复杂，恢复期可能越长，并发症风险也相对较高。

* 手术方式： 不同的手术方式对组织创伤不同，愈合过程和所需时间也会有差异。

* 孩子年龄和个体差异： 不同年龄段的孩子配合度、愈合能力不同。

* 术后护理： 家长是否能严格遵照医嘱进行伤口护理、导尿管护理、用药、限制活动等，对恢复至关重要。

* 是否出现并发症：如感染、伤口裂开、尿瘘、尿道狭窄等，一旦出现并发症，恢复时间会显著延长，甚至可能需要再次手术。

总结来说：

孩子尿道下裂术后，带尿管的时间一般是 1-2 周，伤口初步愈合和拔管大约需要 2-3 周，较为稳定的恢复期（可以逐渐恢复正常活动）大约需要 1-3 个月。但要达到真正意义上的“安全康复”，即功能和外观满意且无远期并发症，则需要数月甚至数年的观察和定期随访。

请您放心， 手术后我们会根据您孩子的具体情况，给您详细的术后护理指导和复查计划。恢复过程中有任何疑问或担忧，请随时与我们医疗团队联系。

来源依据与参考（示例性）：

*临床实践共识/指南： 中华医学会小儿外科学分会可能发布过相关诊疗指南或专家共识，其中会包含术后管理和随访的内容。（具体文献需根据最新发布查找）

* 权威医学教科书： 如《坎贝尔-沃尔什泌尿外科学》（Campbell-Walsh Urology）或《实用小儿外科学》等权威教材中关于尿道下裂章节，会详细描述手术方法、术后管理及恢复过程。

* 医学数据库文献： PubMed, CNKI 等数据库中关于尿道下裂术后恢复、并发症管理的研究论文。例如，可以检索 "hypospadias repair recovery time", "postoperative care hypospadias", "尿道下裂 术后恢复" 等关键词。

  * ‘示例性引用格式（非特定文献）’： Baskin LS. Hypospadias: A Critical Review of Surgical Techniques and Outcomes. *Rev Urol*. 2017;19(2):85-91. (此类文献会讨论不同术式的恢复特点和并发症发生率，间接反映恢复时间)

* 知名儿童医院或泌尿外科中心网站：如波士顿儿童医院、费城儿童医院、北京儿童医院、上海儿童医学中心等，其官方网站的健康信息板块常有关于尿道下裂手术及术后护理的介绍，会提及大致的恢复时间框架。

请注意：上述回答是基于普遍的医学知识和临床经验。具体到您孩子的情况，主刀医生会给出最个体化的建议。

English translation (machine translation; for navigation only):

Okay, hello parents. This is a very good question that many parents are very concerned about. "How long does it take for a baby with hypospadias to recover safely after surgery?" There is no absolutely unified answer to this question, because each child's specific situation is different and the recovery time will also vary. But I can give you a general framework and factors that affect recovery time.

Generally speaking, recovery after hypospadias surgery can be divided into several stages:

1. Early recovery period (tubation period and early stage of extubation, about 2-3 weeks):

* Length of hospitalization: Depending on the complexity of the surgery and the child's recovery, it usually takes a few days to about a week to be hospitalized. Some simple cases can even be completed as day surgeries.

* Time to wear urinary catheter/stent: This is to allow the newly repaired urethra to heal well and set its shape, and to drain urine to avoid direct impact of urine on the wound. This time is usually **7 to 14 days**, and the specific time will be determined based on the surgical method, the complexity of the urethral reconstruction, and the doctor's habits. Some complications may take longer.

* Initial wound healing: After the urinary catheter is removed, the wound has basically completed epidermal healing, but the internal tissue is still being repaired. This stage requires very careful care to avoid infection and wound dehiscence.

2. Mid-term recovery period (after extubation until the wound is stable, about 1-3 months):

* Wound stabilization and scar softening: The appearance of the wound will gradually improve, redness and swelling subside, and scars will begin to soften. But the organization is still relatively fragile at this time.

* Initial recovery of function: The child begins to urinate on his own. It is necessary to observe the shape of the urine line, whether there is urine leakage (urinary fistula), whether it is laborious to urinate (urethral stricture), etc.

* Activity restrictions: During the first 4-6 weeks, strenuous activities need to be limited, especially straddling movements (such as riding a bicycle, rocking horses) or activities that may cause perineal impact to avoid affecting wound healing.

3. Long-term recovery and follow-up period (3 months to several years after surgery):

* The complete concept of "safe recovery": The true "safe recovery" is not only about the wound growing well, but more importantly, achieving satisfactory appearance and function without long-term complications (such as urinary fistula, urethral stricture, urethral diverticulum, recurrence of penile curvature, etc.).

*Regular review: Regular follow-up check-ups are required after surgery (usually 1 month, 3 months, 6 months, and 1 year after surgery, and then annually or every other year until puberty develops). The doctor will evaluate the urination, appearance of the penis, and whether there is curvature, etc. Some complications may not show up until months or even years after surgery.

* Final stability: It is generally believed that half a year to one year after surgery, if no complications occur, it can be considered to have reached a relatively stable state. But final evaluation of appearance and function sometimes has to wait until pubertal development is complete.

Main factors affecting recovery time:

* Severity of hypospadias: The closer the urethral opening is to the scrotum or perineum (severe), the more complicated the surgery, the recovery period may be longer, and the risk of complications is relatively higher.

* Surgical methods: Different surgical methods cause different tissue trauma, and the healing process and time required will also be different.

*Children's age and individual differences: Children of different ages have different cooperation and healing abilities.

* Postoperative care: Whether parents can strictly follow the doctor's instructions for wound care, catheter care, medication, restriction of activities, etc. is crucial to recovery.

*Whether complications occur: such as infection, wound dehiscence, urinary fistula, urethral stricture, etc. Once complications occur, the recovery time will be significantly prolonged, and reoperation may even be required.

To sum up:

After hypospadias surgery in children, the duration of wearing a urinary catheter is generally 1-2 weeks, initial wound healing and extubation takes about 2-3 weeks, and a relatively stable recovery period (where normal activities can be gradually resumed) takes about 1-3 months. However, to achieve a true "safe recovery", that is, satisfactory function and appearance without long-term complications, months or even years of observation and regular follow-up are required.

Please rest assured that after the operation, we will give you detailed postoperative care guidance and review plan based on your child's specific situation. If you have any questions or concerns during the recovery process, please feel free to contact our medical team.

Source basis and reference (example):

*Clinical practice consensus/guidelines: The Pediatric Surgery Branch of the Chinese Medical Association may have issued relevant diagnosis and treatment guidelines or expert consensus, which will include postoperative management and follow-up. (Specific documents need to be found according to the latest release)

* Authoritative medical textbooks: The chapters on hypospadias in authoritative textbooks such as "Campbell-Walsh Urology" or "Practical Pediatric Surgery" will describe the surgical method, postoperative management and recovery process in detail.

*Medical database literature: Research papers on post-hypospadias recovery and complication management in databases such as PubMed and CNKI. For example, you can search for keywords such as "hypospadias repair recovery time", "postoperative care hypospadias", "hypospadias postoperative recovery".

* 'Example citation format (non-specific literature)': Baskin LS. Hypospadias: A Critical Review of Surgical Techniques and Outcomes. *Rev Urol*. 2017;19(2):85-91. (Such literature will discuss the recovery characteristics and complication rates of different surgical procedures, indirectly reflecting recovery time)

* Websites of well-known children's hospitals or urological surgery centers: such as Boston Children's Hospital, Children's Hospital of Philadelphia, Beijing Children's Hospital, Shanghai Children's Medical Center, etc. The health information section of their official websites often has introductions about hypospadias surgery and postoperative care, and will mention a rough recovery time frame.

Please note: The above answers are based on common medical knowledge and clinical experience. Depending on your child's situation, the surgeon will give you the most personalized advice.

AI5 (post-hoc identity: OpenEvidence)

Chinese original (verbatim output):

儿童在接受尿道下裂手术后，通常需要约 1 至 2 周的时间来安全恢复。手术后，患儿通常会留置导尿管以确保尿道的愈合。根据 Daher 等人的研究，导尿管的留置时间可以影响术后并发症的发生率，三周导尿管留置时间比一周导尿管留置时间的并发症率更低。此外，Kim 等人的研究表明，尿道下裂的严重程度和术后并发症的发生时间密切相关，严重的尿道下裂患者在术后两年内发生并发症的概率较高。[1-2]

术后早期并发症包括导尿管脱落、尿潴留和疼痛等，这些并发症可能会延长住院时间。因此，术后护理和随访非常重要，以确保手术的成功和减少并发症的发生。总体而言，大多数儿童在术后 1 至 2 周内可以安全恢复，但需要根据具体情况进行个体化的护理和随访。[3]

1.Three-Week or One-Week Bladder Catheterization for Hypospadias Repair? A Retrospective-Prospective Observational Study of 189 Patients.Daher P, Khoury A, Riachy E, Atallah B.Journal of Pediatric Surgery. 2015;50(6):1063-6. doi:10.1016/j.jpedsurg.2015.03.050.

2.Time to Event Analysis for Post-Hypospadias Repair Complications: A Single-Surgeon Experience.Kim JK, Shiff M, Chua ME, et al.World Journal of Urology. 2021;39(10):3913-3919. doi:10.1007/s00345-021-03689-3.

3.Age-Dependent Early Complications of Hypospadias Repair: A Single Institutional Experience.

Dale J, Woodward B, Elagami H.Pediatric Surgery International. 2023;39(1):115. doi:10.1007/s00383-023-05388-z.

评价指标及定义

请您从文本质量、相关性、适用性、来源可靠性、可理解性、可操作性 6 个方面进行评价，排在越前面意味着其在各指标综合考量下分值越高。

1.质量：衡量 AI 答案对医疗疑问的解决效果，涵盖医学准确性、细节完整性以及提供医学见解的深度，判断其能否为提问者提供有价值、全面且深入的医学指导。

请将 5 个 AI 回答文本按照质量评价进行排序，例如 AI1＞AI2＞AI3＞AI4＞AI5 。

您的作答：　　＞　　＞　　＞　　＞

2.相关性：判断 AI 答案与医学问题的契合程度，包括是否紧扣主题，是否全面涉及医学问题的各个方面，以及所提供信息与医疗问题的关联紧密性。

请将 5 个 AI 回答文本按照相关性进行排序，例如 AI1＞AI2＞AI3＞AI4＞AI5 。

您的作答：　　＞　　＞　　＞　　＞

3.适用性：评估 AI 答案在询问所描述的医学背景和实际医疗场景中的可应用程度，看其是否能直接用于解决实际问题，或是需要大量调整修改。

请将 5 个 AI 回答文本按照适用性进行排序，例如 AI1＞AI2＞AI3＞AI4＞AI5 。

您的作答：　　＞　　＞　　＞　　＞

4.来源可靠性：考查 AI 答案所依据来源的可信度（是否有虚假信息或来源不详细等），包括参考文献的权威性、输出内容与文献的关联度，以及信息来源渠道是否专业、经过严格审核，以此判断答案是否准确、真实且无过时信息。

请将 5 个 AI 回答文本按照来源可靠性进行排序，例如 AI1＞AI2＞AI3＞AI4＞AI5 。

您的作答：　　＞　　＞　　＞　　＞

5.可理解性：评价 AI 答案在语言使用、内容组织以及辅助工具运用等方面，是否便于用户理解，包括语言是否通俗易懂、术语有无定义、结构是否清晰、有无视觉辅助或摘要帮助理解等。

请将 5 个 AI 回答文本按照可理解性进行排序，例如 AI1＞AI2＞AI3＞AI4＞AI5 。

您的作答：　　＞　　＞　　＞　　＞

6.可操作性：判断 AI 答案是否明确指出用户可采取的操作，是否将操作细化为清晰步骤，以及是否提供实用工具辅助用户在实际场景中采取有效行动。

请将 5 个 AI 回答文本按照可操作性进行排序，例如 AI1＞AI2＞AI3＞AI4＞AI5 。

您的作答：　　＞　　＞　　＞　　＞

根据以上内容进行综合排序：：　　＞　　＞　　＞　　＞

English translation (machine translation; for navigation only):

It usually takes about 1 to 2 weeks for children to recover safely after hypospadias surgery. After surgery, children usually have an indwelling urinary catheter to ensure healing of the urethra. According to the study by Daher et al., the indwelling time of the urinary catheter can affect the incidence of postoperative complications. A three-week catheter indwelling time has a lower complication rate than a one-week catheter indwelling time. In addition, the study by Kim et al. showed that the severity of hypospadias is closely related to the occurrence time of postoperative complications. Patients with severe hypospadias have a higher probability of complications within two years after surgery. [1-2]

Early postoperative complications include catheter detachment, urinary retention, and pain, which may prolong hospital stay. Therefore, postoperative care and follow-up are very important to ensure the success of the surgery and reduce the occurrence of complications. Overall, most children recover safely within 1 to 2 weeks after surgery, but individualized care and follow-up are required on a case-by-case basis. [3]

1.Three-Week or One-Week Bladder Catheterization for Hypospadias Repair? A Retrospective-Prospective Observational Study of 189 Patients.Daher P, Khoury A, Riachy E, Atallah B.Journal of Pediatric Surgery. 2015;50(6):1063-6. doi:10.1016/j.jpedsurg.2015.03.050.

2.Time to Event Analysis for Post-Hypospadias Repair Complications: A Single-Surgeon Experience.Kim JK, Shiff M, Chua ME, et al.World Journal of Urology. 2021;39(10):3913-3919. doi:10.1007/s00345-021-03689-3.

3.Age-Dependent Early Complications of Hypospadias Repair: A Single Institutional Experience.

Dale J, Woodward B, Elagami H.Pediatric Surgery International. 2023;39(1):115. doi:10.1007/s00383-023-05388-z.

Evaluation indicators and definitions

Please evaluate it from six aspects: text quality, relevance, applicability, source reliability, understandability, and operability. The higher the ranking, the higher the score based on the comprehensive consideration of each indicator.

1. Quality: Measure the effectiveness of AI answers in solving medical questions, covering medical accuracy, completeness of details and the depth of medical insights provided, to determine whether it can provide valuable, comprehensive and in-depth medical guidance to the questioner.

Please sort the 5 AI answer texts according to quality evaluation, for example, AI1>AI2>AI3>AI4>AI5.

Your answer: ＞＞＞＞

2. Relevance: Determine the degree of fit between the AI answer and the medical problem, including whether it sticks to the topic, whether it comprehensively covers all aspects of the medical problem, and how closely the information provided is related to the medical problem.

Please sort the 5 AI answer texts according to their relevance, for example, AI1>AI2>AI3>AI4>AI5.

Your answer: ＞＞＞＞

3. Applicability: Evaluate the applicability of the AI answers in the medical background and actual medical scenarios described in the query to see whether it can be directly used to solve practical problems, or whether it requires extensive adjustments and modifications.

Please sort the 5 AI answer texts according to their applicability, for example, AI1>AI2>AI3>AI4>AI5.

Your answer: ＞＞＞＞

4. Source reliability: Examine the credibility of the sources on which the AI answers are based (whether there is false information or unclear sources, etc.), including the authority of the references, the relevance of the output content and the literature, and whether the information source channels are professional and rigorously reviewed to determine whether the answers are accurate, authentic and free of outdated information.

Please sort the 5 AI answer texts according to source reliability, for example, AI1>AI2>AI3>AI4>AI5.

Your answer: ＞＞＞＞

5. Comprehensibility: Evaluate whether the AI answers are easy for users to understand in terms of language use, content organization, and use of auxiliary tools, including whether the language is easy to understand, whether terms are defined, whether the structure is clear, and whether there are visual aids or summaries to help understanding, etc.

Please sort the 5 AI answer texts according to their comprehensibility, for example, AI1>AI2>AI3>AI4>AI5.

Your answer: ＞＞＞＞

6. Operability: Determine whether the AI answer clearly points out the actions that the user can take, whether the operation is refined into clear steps, and whether it provides practical tools to assist the user in taking effective actions in actual scenarios.

Please sort the 5 AI answer texts according to operability, for example, AI1>AI2>AI3>AI4>AI5.

Your answer: > > > >

Comprehensive sorting based on the above content:: > > > >

Question 5 (High risk; Other)

Original prompt (Chinese):

如果你是一名小儿泌尿外科的医生，家长问到"尿道下裂患儿手术有哪些潜在风险和并发症？"问题，你应该怎么回答？并给出来源依据或参考出处。

Prompt translation (English):

If you are a pediatric urologist, and a parent asks, "What potential risks and complications can occur with hypospadias surgery?", how should you answer? Please cite your sources or references.

Per-question unblinding key for Q5:

Blinded label	Model identity
AI1	ChatGPT-4o
AI2	DeepSeek
AI3	Gemini-2.5-Pro
AI4	OpenEvidence
AI5	Zhipu Qingyan

AI1 (post-hoc identity: ChatGPT-4o)

Chinese original (verbatim output):

作为一名小儿泌尿外科医生，如果家长问到"尿道下裂患儿手术有哪些潜在风险和并发症？"，可以这样规范且人性化地回答（符合临床沟通习惯，同时具有循证依据）：

医生答复示范：

"您好，尿道下裂手术总体上是比较成熟和安全的，但和所有外科手术一样，也存在一定的风险和潜在并发症。根据目前的医学研究和指南，尿道下裂手术的主要并发症包括：

1. 尿瘘（最常见）：大约 5%~30%的患儿可能发生尿瘘，就是术后尿液从新建尿道以外的地方漏出，严重的需要再次手术修复。
2. 尿道狭窄或憩室：新建尿道在愈合过程中可能出现狭窄，影响排尿，或者形成憩室（局部膨出）。
3. 外观问题：部分患儿术后阴茎外观或尿道口位置不够理想，可能需要进一步修整。
4. 阴茎弯曲残留或复发：尤其是术前弯曲较重的患儿，术后可能存在弯曲未完全纠正或复发的可能。
5. 愈合不良或感染：伤口愈合不好或术区感染也可能发生，但发生率较低。

我们在手术前会综合评估孩子的具体情况，选择最适合他的手术方式，并在术后密切随访，尽可能降低这些风险。”

参考依据或出处：

1. American Urological Association (AUA) Guidelines — Hypospadias Diagnosis and Management (2022)https://www.auanet.org/guidelines/hypospadias-guideline

2. Springer Reference Book: Pediatric Urology — Hypospadias Repair Complications

Editors: Hadley Wood, John Gearhart, Ricardo Gonzalez (2021)

3. 文献综述：中国泌尿外科专业委员会推荐《中国尿道下裂诊治指南（2020）》— 中华医学会泌尿外科学分会

4. 临床研究参考：

Snodgrass W, Bush N. “Primary Hypospadias Repair Techniques: A Review of the Evidence” J Pediatr Urol. 2016.

如果家长特别关注具体数据或风险比例，可以进一步展开，例如：

并发症类型	发生率（大致范围）	说明
尿瘘	5%~30%	与尿道位置、手术方式和术者经验相关
尿道狭窄	2%~10%	多见于复杂或长段尿道重建
阴茎弯曲复发	<5%	重度病例风险稍高
感染或伤口问题	<5%	良好护理可降低风险

English translation (machine translation; for navigation only):

As a pediatric urologist, if a parent asks "What are the potential risks and complications of surgery for children with hypospadias?", they can answer in a standardized and humane way (in line with clinical communication habits and evidence-based):

Doctor’s sample reply:

"Hello, hypospadias surgery is generally relatively mature and safe, but like all surgeries, there are certain risks and potential complications. According to current medical research and guidelines, the main complications of hypospadias surgery include:

1. Urinary fistula (the most common): About 5% to 30% of children may develop urinary fistula, which means urine leaks from places other than the newly created urethra after surgery. In severe cases, another surgery is needed to repair it.

2. Urethral stricture or diverticulum: The newly constructed urethra may become narrow during the healing process, affecting urination, or forming diverticula (local bulging).

3. Appearance problems: In some children, the appearance of the penis or the position of the urethral opening after surgery is not ideal and may require further modification.

4. Retention or recurrence of penile curvature: Especially in children with severe curvature before surgery, there may be a possibility that the curvature is not completely corrected or recurs after surgery.

5. Poor healing or infection: Poor wound healing or infection in the surgical area may also occur, but the incidence is lower.

We will comprehensively evaluate the child's specific situation before surgery, choose the most suitable surgical method for him, and follow up closely after surgery to reduce these risks as much as possible. "

Reference basis or source:

1. American Urological Association (AUA) Guidelines — Hypospadias Diagnosis and Management (2022)https://www.auanet.org/guidelines/hypospadias-guideline

2. Springer Reference Book: Pediatric Urology — Hypospadias Repair Complications

Editors: Hadley Wood, John Gearhart, Ricardo Gonzalez (2021)

3. Literature review: The Chinese Professional Committee of Urology recommends the "Chinese Guidelines for the Diagnosis and Treatment of Hypospadias (2020)" - Urology Branch of the Chinese Medical Association

4. Clinical research reference:

Snodgrass W, Bush N. “Primary Hypospadias Repair Techniques: A Review of the Evidence” J Pediatr Urol. 2016.

If parents are particularly concerned about specific data or risk ratios, they can expand further, for example:

Complication Type Incidence (Approximate Range) Description

Urinary fistula 5%~30% is related to the urethra location, surgical method and operator experience

Urethral stricture 2%~10% is more common in complex or long-segment urethral reconstruction

Recurrence of penile curvature <5%, slightly higher risk in severe cases

Infection or wound problems <5% Good care reduces risk

AI2 (post-hoc identity: DeepSeek)

Chinese original (verbatim output):

作为小儿泌尿外科医生，我会用以下方式向家长解释尿道下裂手术的风险和并发症，并提供科学依据：

尿道下裂手术的潜在风险与并发症

1. 尿道皮肤瘘（尿瘘）

- 表现：尿液从新尿道以外的位置（如缝合处）漏出。

- 发生率：约 5%-30%（根据手术复杂程度和术者经验不同）[^1]。

- 处理：多数需二次手术修补。

2. 尿道狭窄

- 表现：术后尿道变窄导致排尿困难、尿流变细。

- 原因：瘢痕形成或局部缺血[^2]。

- 处理：可通过尿道扩张或再次手术矫正。

3. 伤口感染或裂开

- 风险因素：儿童活动量大、局部卫生不佳。

- 预防：术后使用抗生素敷料、限制剧烈活动[^3]。

4. 尿道憩室

- 表现：尿道局部膨出，可能导致尿液淤积或感染。

- 处理：轻者观察，严重者需手术修复[^4]。

5. 外观不满意

- 原因：阴茎弯曲矫正不足、瘢痕明显或尿道口位置不自然。

- 对策：术前充分沟通期望值，必要时二次整形。

6. 需再次手术

- 概率：约 10%-20%的患儿因并发症或初次效果不佳需二次手术[^5]。

7. 长期排尿或性功能问题

- 罕见风险：成年后可能出现尿流异常或勃起疼痛（多见于严重病例）[^6]。

降低风险的医疗措施

- 术前：通过超声评估阴茎弯曲程度，选择个性化术式（如 Snodgrass 术、Duckett 术等）。

- 术中：使用放大镜或显微镜精细操作，减少组织损伤。

- 术后：规范使用硅胶尿管、预防性抗生素，并定期随访。

权威依据

[^1]: Snodgrass W. *Urethral strictures following hypospadias repair*. World J Urol. 2018.

[^2]: European Association of Urology (EAU) Guidelines on Paediatric Urology, 2022.

[^3]: Springer A. *Complications in hypospadias surgery*. J Pediatr Urol. 2019.

[^4]: American Academy of Pediatrics (AAP) Surgical Standards for Hypospadias, 2021.

[^5]: Long CJ. *Reoperation risk after hypospadias repair: A meta-analysis*. J Urol. 2020.

[^6]: Rynja SP. *Long-term functional outcomes in hypospadias*. BJU Int. 2021.

家长沟通建议

- 共情：理解家长对手术安全的担忧，明确告知并发症可控且团队有丰富处理经验。

- 透明：提供本院具体并发症发生率数据（如有）。

- 随访：强调术后 1 年内的定期复查对早期发现问题至关重要。

如有其他疑问，可进一步讨论具体术式选择或预防措施。

English translation (machine translation; for navigation only):

As a pediatric urologist, I explain the risks and complications of hypospadias surgery to parents in the following ways and provide scientific evidence:

Potential risks and complications of hypospadias surgery

1. Urethrocutaneous fistula (urinary fistula)

- Manifestation: Urine leaks from locations other than the neourethra (such as sutures).

- Incidence rate: about 5%-30% (depending on the complexity of the surgery and the experience of the surgeon) [^1].

- Treatment: Most require secondary surgical repair.

2. Urethral stricture

- Manifestation: Postoperative urethral narrowing leads to difficulty in urinating and thinning of urine stream.

- Cause: Scarring or ischemia[^2].

- Treatment: Can be corrected by urethral dilation or reoperation.

3. Wound infection or dehiscence

- Risk factors: Children are active and have poor local hygiene.

- Prevention: Use antibiotic dressings after surgery and limit strenuous activities[^3].

4. Urethral diverticulum

- Manifestation: Local bulging of the urethra, which may lead to urine accumulation or infection.

- Treatment: Observation in mild cases, surgical repair in severe cases [^4].

5. Unsatisfactory appearance

- Cause: Insufficient correction of penile curvature, obvious scars, or unnatural position of the urethral opening.

- Countermeasures: Fully communicate expectations before surgery, and conduct secondary plastic surgery if necessary.

6. Need for another surgery

- Probability: About 10%-20% of children require a second surgery due to complications or poor initial results[^5].

7. Long-term urination or sexual function problems

- Rare risks: Abnormal urinary flow or painful erections may occur in adulthood (more common in severe cases) [^6].

Medical measures to reduce risk

- Preoperative: Use ultrasound to evaluate the degree of penile curvature and select a personalized surgical procedure (such as Snodgrass surgery, Duckett surgery, etc.).

- Intraoperative: use a magnifying glass or microscope to perform precise operations to reduce tissue damage.

- Postoperative: standard use of silicone urinary catheter, preventive antibiotics, and regular follow-up.

authoritative basis

[^1]: Snodgrass W. *Urethral strictures following hypospadias repair*. World J Urol. 2018.

[^2]: European Association of Urology (EAU) Guidelines on Paediatric Urology, 2022.

[^3]: Springer A. *Complications in hypospadias surgery*. J Pediatr Urol. 2019.

[^4]: American Academy of Pediatrics (AAP) Surgical Standards for Hypospadias, 2021.

[^5]: Long CJ. *Reoperation risk after hypospadias repair: A meta-analysis*. J Urol. 2020.

[^6]: Rynja SP. *Long-term functional outcomes in hypospadias*. BJU Int. 2021.

Parent communication suggestions

- Empathy: Understand parents' concerns about surgical safety, clearly inform them that complications are controllable and the team has rich experience in handling them.

- Transparency: Provide hospital-specific complication rate data (if available).

- Follow-up: It is emphasized that regular review within 1 year after surgery is crucial for early detection of problems.

If you have any other questions, we can further discuss specific surgical options or preventive measures.

AI3 (post-hoc identity: Gemini-2.5-Pro)

Chinese original (verbatim output):

好的，家长您好。您关心孩子手术的风险和并发症，这是非常重要和可以理解的。任何手术都有一定的风险，尿道下裂修复手术虽然技术已经很成熟，成功率也很高，但也确实存在一些潜在的风险和可能出现的并发症。和您详细沟通这些，是为了让您有充分的了解，我们共同努力，争取最好的结果。

尿道下裂手术的主要目标是矫直阴茎（如果存在弯曲）、将尿道开口重建在龟头顶端，并塑造一个外观尽可能正常的阴茎。手术后可能出现的潜在风险和并发症主要包括以下几个方面：

1. 尿道皮肤瘘 (Urethrocutaneous Fistula)：这是最常见的并发症之一。指的是在重建的尿道和阴茎皮肤之间形成了一个异常的小通道，导致排尿时除了从正常的尿道口排尿外，还有尿液从这个瘘口漏出。瘘口的大小不一，小的可能自行愈合，大的或持续存在的通常需要再次手术修复。

2. 尿道狭窄 (Urethral Stricture)：指重建后的尿道部分或全部管腔变窄。可能发生在新的尿道口（称为尿道口狭窄，Meatal Stenosis）或者重建尿道的其他部位。这会导致排尿困难、尿线变细、排尿费力等。轻度的可能通过扩张治疗，严重的可能需要再次手术。

3. 伤口感染与裂开 (Wound Infection and Dehiscence)：任何手术都有感染的风险。术后伤口感染可能导致红肿、疼痛、流脓，严重时可能导致伤口裂开，影响愈合和最终效果。我们会使用抗生素来预防，并指导您做好术后护理。

4. 出血或血肿 (Bleeding/Hematoma)：手术中或手术后可能发生出血。少量渗血是正常的，但如果出血较多形成血肿（血液在组织下积聚），可能会影响伤口愈合，有时需要处理。

5. 阴茎下弯复发或残留 (Recurrent or Residual Chordee)：有些尿道下裂伴有阴茎下弯（Chordee）。手术的一个重要目标是矫直阴茎。但有时术后可能仍有轻微弯曲，或者随着孩子的生长发育，弯曲再次出现。这可能需要再次手术矫正。

6. 尿道憩室 (Urethral Diverticulum)：指重建的尿道壁局部膨出，形成一个囊袋。排尿时尿液可能积存在憩室里，导致排尿后滴沥或增加感染风险。可能需要手术修复。

7. 外观不满意 (Cosmetic Concerns)：虽然手术目标是尽可能恢复正常外观，但有时家长或孩子可能对术后的阴茎外观（如疤痕、形状）不完全满意。

8. 需要再次手术 (Need for Reoperation)：上述提到的并发症，如尿道瘘、尿道狭窄、弯曲复发等，如果出现且无法通过保守方法解决，就可能需要进行再次手术修复。再次手术通常比初次手术更复杂。

9. 麻醉风险 (Anesthesia Risks)：就像所有需要全身麻醉的手术一样，存在与麻醉相关的风险，尽管在现代医学条件下，小儿麻醉总体上是非常安全的。麻醉医生会在术前对孩子进行评估。

需要强调的是：

* 这些并发症的发生率与尿道下裂的严重程度、选择的手术方式、手术医生的经验以及孩子自身的愈合能力等多种因素有关。一般来说，轻度的尿道下裂，并发症发生率相对较低；复杂、严重的尿道下裂，手术难度大，风险相对会高一些。

* 我们医疗团队会采取各种措施来最大程度地降低这些风险，包括精心的术前规划、精细的手术操作、术中预防感染、术后仔细的护理指导等。

* 即使发生了并发症，绝大多数也是可以处理和修复的。我们会密切关注孩子术后的恢复情况，一旦发现问题会及时处理。

请您放心，我们会根据您孩子的具体情况，选择最合适的手术方案，并尽最大努力确保手术成功、减少并发症的发生。如果您还有任何其他疑问或担忧，请随时提出来，我们非常乐意为您解答。

来源依据与参考出处：

这些信息是基于标准的小儿泌尿外科学知识和临床实践。您可以参考以下类型的资源获取更详细或权威的信息：

1. 权威医学教科书：

   * 《坎贝尔-沃尔什泌尿外科学》（Campbell-Walsh Urology）：这是泌尿外科领域的权威参考书，有专门章节讨论尿道下裂及其手术。

   * 《小儿泌尿外科学》（Pediatric Urology）：多种以此命名的专业书籍。

2. 专业医学协会指南与患者信息：

   * 美国泌尿外科学会 (American Urological Association, AUA) - 提供相关的患者教育材料。

   * 欧洲小儿泌尿外科学会 (European Society for Paediatric Urology, ESPU) - 提供专业指南。

3. 知名儿童医院的官方网站：

* 例如，波士顿儿童医院 (Boston Children's Hospital)、费城儿童医院 (Children's Hospital of Philadelphia - CHOP)、梅奥诊所 (Mayo Clinic) 等网站通常有关于尿道下裂及其治疗的详细介绍，包括风险和并发症。

4. 同行评议的医学文献数据库：

* 如 PubMed (pubmed.ncbi.nlm.nih.gov)，可以搜索关于 "hypospadias surgery complications" 的研究论文，了解具体的发生率和风险因素分析（这部分内容可能对非专业人士来说比较难懂）。

请注意，网络信息繁杂，建议优先参考上述权威机构或专业书籍发布的信息。与您的主治医生进行充分沟通是获取针对您孩子具体情况信息的最佳途径。

English translation (machine translation; for navigation only):

Okay, hello parents. It is important and understandable that you are concerned about the risks and complications of your child's surgery. Any surgery has certain risks. Although the technology of hypospadias repair surgery is very mature and the success rate is high, there are indeed some potential risks and possible complications. Communicating these with you in detail is to give you a full understanding, and we will work together to strive for the best results.

The main goals of hypospadias surgery are to straighten the penis (if curvature is present), reestablish the urethral opening at the tip of the glans, and create a penis that is as normal looking as possible. Potential risks and complications that may occur after surgery mainly include the following aspects:

1. Urethrocutaneous Fistula: This is one of the most common complications. It refers to the formation of an abnormal small channel between the reconstructed urethra and the skin of the penis, causing urine to leak from this fistula in addition to urinating from the normal urethral opening during urination. Fistulas vary in size; small ones may heal on their own, but large or persistent ones often require additional surgery to repair.

2. Urethral Stricture: refers to the narrowing of part or all of the lumen of the urethra after reconstruction. It may occur with a new urethral opening (called meatal stenosis) or with reconstruction of other parts of the urethra. This can lead to difficulty urinating, thinning of the urine line, and difficulty in urinating. Mild cases may be treated with dilation, while severe cases may require additional surgery.

3. Wound Infection and Dehiscence: Any surgery carries the risk of infection. Postoperative wound infection may cause redness, swelling, pain, and pus. In severe cases, it may cause wound dehiscence, affecting healing and final results. We will give you antibiotics for prophylaxis and guide you through post-operative care.

4. Bleeding/Hematoma: Bleeding may occur during or after surgery. A small amount of bleeding is normal, but if there is more bleeding and a hematoma (blood accumulation under the tissue) occurs, it may affect wound healing and sometimes requires treatment.

5. Recurrent or Residual Chordee: Some hypospadias are accompanied by Chordee. An important goal of surgery is to straighten the penis. But sometimes there may still be a slight curvature after surgery, or the curvature may reappear as the child grows and develops. This may require another surgical correction.

6. Urethral Diverticulum: refers to the partial bulging of the reconstructed urethral wall, forming a pocket. Urine may accumulate in diverticula during urination, causing post-urination dripping or increasing the risk of infection. Surgical repair may be required.

7. Cosmetic Concerns: Although the goal of surgery is to restore the normal appearance as much as possible, sometimes parents or children may not be completely satisfied with the postoperative appearance of the penis (such as scars, shape).

8. Need for Reoperation: If the complications mentioned above, such as urethral fistula, urethral stricture, recurrence of curvature, etc., occur and cannot be solved by conservative methods, reoperation may be required. Reoperation is usually more complex than the initial surgery.

9. Anesthesia Risks: As with all surgeries requiring general anesthesia, there are risks associated with anesthesia, although pediatric anesthesia is generally very safe under modern medical conditions. The anesthesiologist will evaluate your child before surgery.

What needs to be emphasized is:

*The incidence of these complications is related to various factors such as the severity of hypospadias, the surgical method chosen, the experience of the surgeon, and the child's own healing ability. Generally speaking, mild hypospadias has a relatively low complication rate; complex and severe hypospadias are more difficult to operate and the risks are relatively higher.

* Our medical team will take various measures to minimize these risks, including careful preoperative planning, meticulous surgical operations, intraoperative infection prevention, careful postoperative care guidance, etc.

* Even if complications occur, most can be treated and repaired. We will pay close attention to the child's recovery after surgery and deal with problems in a timely manner if any problems are discovered.

Please rest assured that we will choose the most appropriate surgical plan based on your child's specific situation and do our best to ensure the success of the surgery and reduce the occurrence of complications. If you have any other questions or concerns, please feel free to ask and we'll be happy to answer them.

Source basis and reference:

This information is based on standard pediatric urology knowledge and clinical practice. You can refer to the following types of resources for more detailed or authoritative information:

1. Authoritative medical textbook:

*Campbell-Walsh Urology: This is the authoritative reference text in the field of urology, with a dedicated chapter discussing hypospadias and its surgery.

* "Pediatric Urology": A variety of professional books with this name.

2. Professional Medical Association Guidelines and Patient Information:

* American Urological Association (AUA) - Provides relevant patient education materials.

* European Society for Pediatric Urology (ESPU) - Provides professional guidance.

3. Official website of a well-known children’s hospital:

* For example, websites such as Boston Children's Hospital, Children's Hospital of Philadelphia (CHOP), and the Mayo Clinic often have detailed information about hypospadias and its treatment, including risks and complications.

4. Peer-reviewed medical literature database:

* For example, PubMed (pubmed.ncbi.nlm.nih.gov), you can search for research papers on "hypospadias surgery complications" to understand the specific incidence and risk factor analysis (this part may be difficult for non-professionals to understand).

Please note that there is a lot of information on the Internet, and it is recommended that you give priority to the information published by the above-mentioned authoritative organizations or professional books. Full communication with your primary care physician is the best way to obtain information specific to your child's situation.

AI4 (post-hoc identity: OpenEvidence)

Chinese original (verbatim output):

手术治疗小儿尿道下裂的潜在风险和并发症包括以下几个方面：

1. 尿瘘：这是最常见的并发症之一，发生率在 5%到 70%之间，具体取决于手术类型和尿道下裂的严重程度。[1-12]

2. 尿道狭窄：手术后尿道狭窄的发生率较高，尤其是在近端尿道下裂的病例中。[1-12]

3. 尿道憩室：这是由于尿道壁的薄弱或手术技术问题导致的尿道扩张。[1-12]

4. 龟头裂开：手术后龟头裂开的风险也存在，尤其是在手术技术不当或术后护理不佳的情况下。[1-12]

5. 阴茎弯曲复发：手术后阴茎弯曲可能会复发，尤其是在近端尿道下裂的病例中。[1-12]

6. 感染和愈合问题：手术部位感染和愈合不良也是常见的并发症，可能需要进一步的医疗干预。[1-12]

7. 心理影响：手术和并发症可能对患儿及其家庭造成心理压力，需要长期的心理支持和随访。[1-12]

这些并发症的发生率和严重程度与手术类型、尿道下裂的严重程度、手术技术以及术后护理密切相关。长期随访对于及时发现和处理这些并发症至关重要。

1.Discrepant Rates of Hypospadias Surgical Complications: A Comparison of U.S. News & World Report and Pediatric Health Information System® Data and Published Literature.Pohl HG, Rana S, Sprague BM, Beamer M, Rushton HG.The Journal of Urology. 2020;203(3):616-623. doi:10.1097/JU.0000000000000554.

2.Risk Factors for Postoperative Complications Following One-Stage Proximal Hypospadias RepairInvolving the Disconnection of the Urethral Plate: A Retrospective Study.

Hu J, Zhao Y, He T, et al.BMC Pediatrics. 2023;23(1):504. doi:10.1186/s12887-023-04339-w.

3.Complications Following Staged Hypospadias Repair Using Transposed Preputial Skin Flaps.Stanasel I, Le HK, Bilgutay A, et al.The Journal of Urology. 2015;194(2):512-6. doi:10.1016/j.juro.2015.02.044.

4.Prevalence, Repairs and Complications of Hypospadias: An Australian Population-Based Study.

Schneuer FJ, Holland AJ, Pereira G, Bower C, Nassar N.Archives of Disease in Childhood. 2015;100(11):1038-43. doi:10.1136/archdischild-2015-308809.

5.Time to Complication Detection After Primary Pediatric Hypospadias Repair: A Large, Single Center, Retrospective Cohort Analysis.Lucas J, Hightower T, Weiss DA, et al.The Journal of Urology. 2020;204(2):338-344. doi:10.1097/JU.0000000000000762.

6.Effect of Caudal vs. Penile Block on the Incidence of Hypospadias Complications Following Primary Repairs: A Retrospective Cohort Study.Adler AC, Chandrakantan A, Lee AD, et al.

The Journal of Urology. 2021;205(5):1454-1459. doi:10.1097/JU.0000000000001448.

7.Late Surgical Correction of Hypospadias Increases the Risk of Complications: A Series of 501 Consecutive Patients.Garnier S, Maillet O, Cereda B, et al.BJU International. 2017;119(6):942-947. doi:10.1111/bju.13771.

8.Quality Assessment of Hypospadias Repair With Emphasis on Techniques Used and Experience of Pediatric Urologic Surgeons.Chrzan R, Dik P, Klijn AJ, de Jong TP.Urology. 2007;70(1):148-52. doi:10.1016/j.urology.2007.01.103.

9.Failed Hypospadias in Paediatric Patients.Cimador M, Vallasciani S, Manzoni G, et al.Nature Reviews. Urology. 2013;10(11):657-66. doi:10.1038/nrurol.2013.164.

10.Management of Complications After Hypospadias Repair.Snyder CL, Evangelidis A, Hansen G, et al.Urology. 2005;65(4):782-5. doi:10.1016/j.urology.2004.11.037.

11.Intermediate-Term Followup of Proximal Hypospadias Repair Reveals High Complication Rate.

Long CJ, Chu DI, Tenney RW, et al.The Journal of Urology. 2017;197(3 Pt 2):852-858. doi:10.1016/j.juro.2016.11.054.

12.Primary Hypospadias Repair Outcomes: Results From a Retrospective Cohort of 292 Children.

Hild O, Fotso Kamdem A, Boulard N, Auber F, Chaussy Y.World Journal of Urology. 2024;42(1):137. doi:10.1007/s00345-024-04811-x.

English translation (machine translation; for navigation only):

Potential risks and complications of surgical treatment of hypospadias in children include the following:

1. Urinary fistula: This is one of the most common complications, with an incidence ranging from 5% to 70%, depending on the type of surgery and the severity of hypospadias. [1-12]

2. Urethral stricture: The incidence of urethral stricture is higher after surgery, especially in cases of proximal hypospadias. [1-12]

3. Urethral diverticulum: This is a dilation of the urethra due to weakness in the urethral wall or problems with surgical technique. [1-12]

4. Glans dehiscence: There is also a risk of glans dehiscence after surgery, especially if surgical technique is improper or post-operative care is poor. [1-12]

5. Recurrence of penile curvature: Recurrence of penile curvature may occur after surgery, especially in cases of proximal hypospadias. [1-12]

6. Infections and Healing Issues: Surgical site infections and poor healing are also common complications and may require further medical intervention. [1-12]

7. Psychological impact: Surgery and complications may cause psychological pressure on children and their families, requiring long-term psychological support and follow-up. [1-12]

The incidence and severity of these complications are closely related to the type of surgery, severity of hypospadias, surgical technique, and postoperative care. Long-term follow-up is crucial for timely detection and management of these complications.

1.Discrepant Rates of Hypospadias Surgical Complications: A Comparison of U.S. News & World Report and Pediatric Health Information System® Data and Published Literature.Pohl HG, Rana S, Sprague BM, Beamer M, Rushton HG.The Journal of Urology. 2020;203(3):616-623. doi:10.1097/JU.0000000000000554.

2.Risk Factors for Postoperative Complications Following One-Stage Proximal Hypospadias RepairInvolving the Disconnection of the Urethral Plate: A Retrospective Study.

Hu J, Zhao Y, He T, et al.BMC Pediatrics. 2023;23(1):504. doi:10.1186/s12887-023-04339-w.

3.Complications Following Staged Hypospadias Repair Using Transposed Preputial Skin Flaps.Stanasel I, Le HK, Bilgutay A, et al.The Journal of Urology. 2015;194(2):512-6. doi:10.1016/j.juro.2015.02.044.

4.Prevalence, Repairs and Complications of Hypospadias: An Australian Population-Based Study.

Schneuer FJ, Holland AJ, Pereira G, Bower C, Nassar N.Archives of Disease in Childhood. 2015;100(11):1038-43. doi:10.1136/archdischild-2015-308809.

5.Time to Complication Detection After Primary Pediatric Hypospadias Repair: A Large, Single Center, Retrospective Cohort Analysis.Lucas J, Hightower T, Weiss DA, et al.The Journal of Urology. 2020;204(2):338-344. doi:10.1097/JU.0000000000000762.

6.Effect of Caudal vs. Penile Block on the Incidence of Hypospadias Complications Following Primary Repairs: A Retrospective Cohort Study.Adler AC, Chandrakantan A, Lee AD, et al.

The Journal of Urology. 2021;205(5):1454-1459. doi:10.1097/JU.0000000000001448.

7.Late Surgical Correction of Hypospadias Increases the Risk of Complications: A Series of 501 Consecutive Patients.Garnier S, Maillet O, Cereda B, et al.BJU International. 2017;119(6):942-947. doi:10.1111/bju.13771.

8.Quality Assessment of Hypospadias Repair With Emphasis on Techniques Used and Experience of Pediatric Urologic Surgeons.Chrzan R, Dik P, Klijn AJ, de Jong TP.Urology. 2007;70(1):148-52. doi:10.1016/j.urology.2007.01.103.

9.Failed Hypospadias in Paediatric Patients.Cimador M, Vallasciani S, Manzoni G, et al.Nature Reviews. Urology. 2013;10(11):657-66. doi:10.1038/nrurol.2013.164.

10.Management of Complications After Hypospadias Repair.Snyder CL, Evangelidis A, Hansen G, et al.Urology. 2005;65(4):782-5. doi:10.1016/j.urology.2004.11.037.

11.Intermediate-Term Followup of Proximal Hypospadias Repair Reveals High Complication Rate.

Long CJ, Chu DI, Tenney RW, et al.The Journal of Urology. 2017;197(3 Pt 2):852-858. doi:10.1016/j.juro.2016.11.054.

12.Primary Hypospadias Repair Outcomes: Results From a Retrospective Cohort of 292 Children.

Hild O, Fotso Kamdem A, Boulard N, Auber F, Chaussy Y.World Journal of Urology. 2024;42(1):137. doi:10.1007/s00345-024-04811-x.

AI5 (post-hoc identity: Zhipu Qingyan)

Chinese original (verbatim output):

1.引言

尿道下裂是一种常见的先天性泌尿系统畸形，手术矫正是主要的治疗方式。

2.麻醉风险

解释：手术过程中需要全身麻醉，可能存在过敏反应、呼吸困难等风险。

参考：现代麻醉技术成熟，风险相对较低。

3.感染

解释：术后可能发生手术部位感染。

预防措施：使用抗生素预防感染。

处理：如出现红肿、发热等症状，需及时就医。

4.瘢痕形成

解释：手术部位可能形成瘢痕，影响阴茎的外观和功能。

参考：瘢痕严重程度个体差异较大，部分患儿可能需要进一步治疗。

5.尿瘘

解释：尿液可能从手术部位以外的地方漏出。

处理：可能需要再次手术修复。

6.尿道狭窄

解释：尿道可能变窄，导致排尿困难。

处理：可能需要尿道扩张或再次手术治疗。

7.阴茎弯曲

解释：部分患儿可能出现阴茎弯曲，需进一步矫正手术。

8.不治疗的后果

解释：不进行手术可能導致排尿困难、尿路感染、心理问题等更严重后果。

9.术后护理与沟通

解释：医疗团队会尽力减少并发症风险，并提供密切的术后护理。

建议：家长如有任何疑问或担忧，应与医生充分沟通。

参考依据：

《坎贝尔-沃尔什泌尿外科学》（Campbell-Walsh Urology）

美国泌尿外科学会（American Urological Association, AUA）相关指南

尿道下裂手术的潜在风险和并发症包括麻醉风险、感染、瘢痕形成、尿瘘、尿道狭窄和阴茎弯曲等。虽然手术有一定的风险，但不治疗可能会导致更严重的后果。医疗团队会尽力减少风险，并提供术后护理。家长如有任何疑问，应与医生充分沟通。

评价指标及定义

请您从文本质量、相关性、适用性、来源可靠性、可理解性、可操作性 6 个方面进行评价，排在越前面意味着其在各指标综合考量下分值越高。

1.质量：衡量 AI 答案对医疗疑问的解决效果，涵盖医学准确性、细节完整性以及提供医学见解的深度，判断其能否为提问者提供有价值、全面且深入的医学指导。

请将 5 个 AI 回答文本按照质量评价进行排序，例如 AI1＞AI2＞AI3＞AI4＞AI5 。

您的作答：　　　＞　　　＞　　　＞　　　＞

2.相关性：判断 AI 答案与医学问题的契合程度，包括是否紧扣主题，是否全面涉及医学问题的各个方面，以及所提供信息与医疗问题的关联紧密性。

请将 5 个 AI 回答文本按照相关性进行排序，例如 AI1＞AI2＞AI3＞AI4＞AI5 。

您的作答：　　　＞　　　＞　　　＞　　　＞

3.适用性：评估 AI 答案在询问所描述的医学背景和实际医疗场景中的可应用程度，看其是否能直接用于解决实际问题，或是需要大量调整修改。

请将 5 个 AI 回答文本按照适用性进行排序，例如 AI1＞AI2＞AI3＞AI4＞AI5 。

您的作答：　　　＞　　　＞　　　＞　　　＞

4.来源可靠性：考查 AI 答案所依据来源的可信度（是否有虚假信息或来源不详细等），包括参考文献的权威性、输出内容与文献的关联度，以及信息来源渠道是否专业、经过严格审核，以此判断答案是否准确、真实且无过时信息。

请将 5 个 AI 回答文本按照来源可靠性进行排序，例如 AI1＞AI2＞AI3＞AI4＞AI5 。

您的作答：　　　＞　　　＞　　　＞　　　＞

5.可理解性：评价 AI 答案在语言使用、内容组织以及辅助工具运用等方面，是否便于用户理解，包括语言是否通俗易懂、术语有无定义、结构是否清晰、有无视觉辅助或摘要帮助理解等。

请将 5 个 AI 回答文本按照可理解性进行排序，例如 AI1＞AI2＞AI3＞AI4＞AI5 。

您的作答：　　　＞　　　＞　　　＞　　　＞

6.可操作性：判断 AI 答案是否明确指出用户可采取的操作，是否将操作细化为清晰步骤，以及是否提供实用工具辅助用户在实际场景中采取有效行动。

请将 5 个 AI 回答文本按照可操作性进行排序，例如 AI1＞AI2＞AI3＞AI4＞AI5 。

您的作答：　　　＞　　　＞　　　＞　　　＞

根据以上内容进行综合排序：：　　＞　　＞　　＞　　＞

English translation (machine translation; for navigation only):

1. Introduction

Hypospadias is a common congenital urinary system malformation, and surgical correction is the main treatment method.

2. Anesthesia risks

Explanation: General anesthesia is required during the operation, and there may be risks such as allergic reactions and difficulty breathing.

Reference: Modern anesthesia technology is mature and the risks are relatively low.

3. Infection

Explanation: Surgical site infection may occur postoperatively.

Precautions: Use antibiotics to prevent infection.

Treatment: If symptoms such as redness, swelling, and fever occur, seek medical attention promptly.

4.Scarring

Explanation: Scarring may form at the surgical site, affecting the appearance and function of the penis.

Reference: The severity of scars varies greatly among individuals, and some children may require further treatment.

5. Urinary fistula

Explanation: Urine may leak from locations other than the surgical site.

Treatment: Additional surgical repair may be required.

6. Urethral stricture

Explanation: The urethra may become narrowed, making urination difficult.

Treatment: Urethral dilation or reoperation may be required.

7. Penis curvature

Explanation: Some children may have penile curvature and require further corrective surgery.

8. Consequences of no treatment

Explanation: Failure to undergo surgery may lead to more serious consequences such as difficulty urinating, urinary tract infections, and psychological problems.

9. Postoperative care and communication

Explanation: The medical team will try to reduce the risk of complications and provide close postoperative care.

Recommendation: Parents should fully communicate with their doctors if they have any questions or concerns.

Reference basis:

Campbell-Walsh Urology

Guidelines from the American Urological Association (AUA)

Potential risks and complications of hypospadias surgery include anesthetic risks, infection, scarring, urinary fistulas, urethral stricture, and penile curvature. While surgery carries certain risks, leaving it untreated can lead to more serious consequences. The medical team will do their best to reduce risks and provide post-operative care. If parents have any questions, they should fully communicate with the doctor.

Evaluation indicators and definitions

Please evaluate it from six aspects: text quality, relevance, applicability, source reliability, understandability, and operability. The higher the ranking, the higher the score based on the comprehensive consideration of each indicator.

1. Quality: Measure the effectiveness of AI answers in solving medical questions, covering medical accuracy, completeness of details and the depth of medical insights provided, to determine whether it can provide valuable, comprehensive and in-depth medical guidance to the questioner.

Please sort the 5 AI answer texts according to quality evaluation, for example, AI1>AI2>AI3>AI4>AI5.

Your answer: > > > >

2. Relevance: Determine the degree of fit between the AI answer and the medical problem, including whether it sticks to the topic, whether it comprehensively covers all aspects of the medical problem, and how closely the information provided is related to the medical problem.

Please sort the 5 AI answer texts according to their relevance, for example, AI1>AI2>AI3>AI4>AI5.

Your answer: > > > >

3. Applicability: Evaluate the applicability of the AI answers in the medical background and actual medical scenarios described in the query to see whether it can be directly used to solve practical problems, or whether it requires extensive adjustments and modifications.

Please sort the 5 AI answer texts according to their applicability, for example, AI1>AI2>AI3>AI4>AI5.

Your answer: > > > >

4. Source reliability: Examine the credibility of the sources on which the AI answers are based (whether there is false information or unclear sources, etc.), including the authority of the references, the relevance of the output content and the literature, and whether the information source channels are professional and rigorously reviewed to determine whether the answers are accurate, authentic and free of outdated information.

Please sort the 5 AI answer texts according to source reliability, for example, AI1>AI2>AI3>AI4>AI5.

Your answer: > > > >

5. Comprehensibility: Evaluate whether the AI answers are easy for users to understand in terms of language use, content organization, and use of auxiliary tools, including whether the language is easy to understand, whether terms are defined, whether the structure is clear, and whether there are visual aids or summaries to help understanding, etc.

Please sort the 5 AI answer texts according to their comprehensibility, for example, AI1>AI2>AI3>AI4>AI5.

Your answer: > > > >

6. Operability: Determine whether the AI answer clearly points out the actions that the user can take, whether the operation is refined into clear steps, and whether it provides practical tools to assist the user in taking effective actions in actual scenarios.

Please sort the 5 AI answer texts according to operability, for example, AI1>AI2>AI3>AI4>AI5.

Your answer: > > > >

Comprehensive sorting based on the above content:: > > > >

Question 6 (High risk; Postoperative Care)

Original prompt (Chinese):

如果你是一名小儿泌尿外科的医生，家长问到“尿道下裂患儿如何预防术后并发症的发生？”问题，你应该怎么回答？并给出来源依据或参考出处。

Prompt translation (English):

If you are a pediatric urologist, and a parent asks, "How can we prevent postoperative complications in a child with hypospadias?", how should you answer? Please cite your sources or references.

Per-question unblinding key for Q6:

Blinded label	Model identity
AI1	Gemini-2.5-Pro
AI2	ChatGPT-4o
AI3	OpenEvidence
AI4	Zhipu Qingyan
AI5	DeepSeek

AI1 (post-hoc identity: Gemini-2.5-Pro)

Chinese original (verbatim output):

好的，家长您好。您这个问题问得非常好，也是我们医生和家长都非常关心的问题。尿道下裂手术虽然技术已经很成熟，但术后护理确实对预防并发症、保证手术效果至关重要。作为小儿泌尿外科医生，我会告诉您，预防术后并发症需要我们医患双方，特别是家长的细心配合。主要注意以下几个方面：

1. 严格遵医嘱进行伤口和敷料护理：

 * 保持清洁干燥： 手术后，我们会用无菌敷料覆盖伤口。请务必按照我们的指示保护好敷料，避免被尿液或粪便污染。我们会告知您何时可以移除敷料，以及移除后的护理方法（如是否需要药浴、涂抹药膏等）。

 * 防止敷料脱落： 要注意固定好敷料，防止孩子活动时过早脱落或移位。

2. 妥善管理尿管/支架管：

 * 保持通畅： 大多数尿道下裂术后会留置尿管或内部支架管，目的是支撑新尿道、引流尿液，让伤口更好地愈合。家长需要确保尿管通畅，引流袋（如果有的话）低于膀胱水平，避免尿管扭曲、受压或堵塞。

 * 防止脱出： 要特别注意防止孩子意外拔出尿管。我们可能会建议使用双尿布法（将尿管从内层尿布的小洞中引出到外层尿布，内层接大便，外层接小便和引流尿液）来帮助固定和保持清洁。

* 观察引流情况： 注意观察尿液的颜色、量和有无沉淀物。如有异常（如长时间无尿液流出、尿色深红等），需及时联系医生。

3. 限制活动，避免伤口受压或损伤：

* 安静休养： 术后早期（通常是几周内，具体时间遵医嘱），需要限制孩子的剧烈活动，如跑、跳、骑跨玩具（如小自行车、木马）等，避免对会阴部造成压力或撞击，影响伤口愈合或导致尿管移位。

* 正确抱姿： 抱孩子时也要注意姿势，避免挤压到手术区域。

4. 合理使用药物：

* 止痛： 术后疼痛是正常的，我们会根据情况给予止痛药。按时按量给药，有助于孩子舒适，减少因疼痛哭闹挣扎对伤口造成的影响。

* 抗生素： 通常会使用抗生素预防感染，请务必按时按量完成整个疗程，即使孩子看起来已经好转。

* 解痉药物（可能）： 有时会使用药物（如奥昔布宁）来减少膀胱痉挛，这有助于减轻疼痛和防止孩子用力排尿。

5. 注意饮食和排便：

* 鼓励饮水： 充足的水分可以稀释尿液，保持尿流畅通，减少感染风险。

* 保持大便通畅：避免便秘，因为排便困难时用力可能影响伤口。可以适当调整饮食，多吃富含纤维的食物，必要时可在医生指导下使用温和的通便药物。

6. 密切观察，及时发现异常：

* 感染迹象： 注意观察伤口有无红肿加剧、流脓、异常分泌物、异味；孩子有无发热等。

* 出血： 少量渗血可能正常，但如果出血量大或持续不止，需立即联系医生。

* 排尿问题： 拔除尿管后，要观察孩子排尿是否顺畅，尿线形态如何，有无尿瘘（从非尿道口漏尿）、尿道狭窄（排尿费力、尿线细）等情况。

* 支架管/尿管问题： 如发生堵塞、过早脱落等。

7. 按时复诊：

* 严格按照医生安排的时间进行复诊非常重要。复诊时医生会评估伤口愈合情况、排尿情况，及时发现并处理潜在问题。

总结来说，家长最重要的角色就是细心观察、严格执行医嘱、及时沟通。 虽然我们尽力做好手术，但术后的精心护理是手术成功和减少并发症的关键一环。请不要担心，我们会和您一起努力，确保孩子顺利康复。有任何疑问或担忧，随时和我们医疗团队联系。

来源依据或参考出处：

这些建议是基于小儿泌尿外科临床实践的普遍共识和指南。您可以参考以下类型的资源获取更详细或权威的信息（请注意，具体操作需遵照您的主治医师的个性化指导）：

1. 权威医学教科书：*如《Campbell-Walsh Urology》（坎贝尔-沃尔什泌尿外科学）或《Ashcraft's Pediatric Surgery》（阿什克拉夫特小儿外科学）中关于尿道下裂章节的术后管理部分。

2. 专业学会指南/共识： 美国泌尿外科学会（AUA）、欧洲泌尿外科学会（EAU）、中华医学会泌尿外科学分会儿科学组等发布的关于尿道下裂诊疗的指南或专家共识，其中会包含术后护理的原则。

3. 大型儿童医院或医学中心的官方网站/患者教育资料：许多知名的儿童医院（如波士顿儿童医院、费城儿童医院、国内大型三甲医院的小儿外科/泌尿外科）会提供关于尿道下裂手术及术后护理的患者和家属教育信息。 *例如，可以搜索特定医院名称 + "hypospadias postoperative care" 或 "尿道下裂术后护理"。*

4. 同行评议的医学文献： 发表在专业医学期刊上的关于尿道下裂术后并发症预防和管理的研究论文和综述。 *例如，可以在 PubMed 等数据库搜索相关关键词。*

请注意： 网络信息仅供参考，不能替代主治医师的专业判断和具体指导。每个孩子的情况不同，手术方式也可能不同，务必以您的主管医生和护士的交代为准。

English translation (machine translation; for navigation only):

Okay, hello parents. You asked this very good question, and it is also a question that our doctors and parents are very concerned about. Although the technology of hypospadias surgery is very mature, postoperative care is indeed crucial to prevent complications and ensure surgical results. As a pediatric urologist, I will tell you that preventing postoperative complications requires careful cooperation from both our doctors and patients, especially parents. Mainly pay attention to the following aspects:

1. Strictly follow the doctor’s instructions for wound and dressing care:

* Keep it clean and dry: After surgery, we will cover the wound with a sterile dressing. Be sure to follow our instructions to protect the dressing from contamination with urine or feces. We will tell you when the dressing can be removed and how to care for it after removal (such as whether medicated baths, ointments, etc. are needed).

* Prevent the dressing from falling off: Pay attention to fixing the dressing to prevent premature falling off or shifting when the child moves.

2. Properly manage urinary catheters/stents:

* Maintain patency: Most hypospadias surgeries will leave a urinary catheter or internal stent in place to support the new urethra, drain urine, and allow the wound to heal better. Parents need to ensure that the urinary tube is clear and that the drainage bag (if applicable) is below the level of the bladder to avoid twisting, pressure, or blockage of the urinary tube.

* Prevent prolapse: Pay special attention to prevent children from accidentally pulling out the urinary catheter. We may recommend using the double diaper method (routing the urinary tube from the hole in the inner diaper to the outer diaper, the inner layer catches stool, and the outer layer catches and drains urine) to help secure and keep it clean.

* Observe the drainage situation: Pay attention to the color, volume and presence of sediment in the urine. If there are any abnormalities (such as no urine flowing out for a long time, dark red urine, etc.), you need to contact your doctor in time.

3. Limit activities to avoid pressure or damage to the wound:

* Quiet recuperation: In the early postoperative period (usually within a few weeks, the specific time is subject to the doctor's advice), it is necessary to limit the child's strenuous activities, such as running, jumping, riding on toys (such as small bicycles, wooden horses), etc., to avoid causing pressure or impact on the perineum, affecting wound healing or causing urinary catheter displacement.

* Correct holding posture: Pay attention to your posture when holding your child to avoid squeezing the surgical area.

4. Use drugs rationally:

* Pain relief: Postoperative pain is normal, and we will give painkillers according to the situation. Administering the medicine on time and in the right amount will help the child feel comfortable and reduce the impact on the wound caused by crying and struggling in pain.

* Antibiotics: Antibiotics are usually given to prevent infection, but be sure to complete the entire course of treatment on time and in the right amount, even if your child seems to be getting better.

* Antispasmodic medications (possibly): Medications (such as oxybutynin) are sometimes used to reduce bladder spasms, which can help reduce pain and prevent your child from straining to urinate.

5. Pay attention to diet and defecation:

* Encourage drinking water: Adequate water can dilute urine, keep urine flowing smoothly, and reduce the risk of infection.

* Keep stools clear: Avoid constipation, as straining during difficult defecation may affect the wound. You can adjust your diet appropriately and eat more fiber-rich foods. If necessary, you can use mild laxatives under the guidance of a doctor.

6. Observe closely and detect abnormalities in time:

* Signs of infection: Pay attention to the wound to see if there is increased redness, swelling, pus, abnormal secretions, odor; whether the child has fever, etc.

* Bleeding: A small amount of bleeding may be normal, but if the bleeding is heavy or persistent, contact your doctor immediately.

* Urination problems: After removing the urinary catheter, observe whether the child urinates smoothly, the shape of the urine line, whether there are urinary fistulas (urine leakage from non-urethral openings), urethral stricture (difficulty urinating, thin urine lines), etc.

*Stent/urinary catheter problems: such as blockage, premature detachment, etc.

7. Follow-up visits on time:

* It is very important to strictly follow the time scheduled by the doctor for follow-up consultations. During the follow-up visit, the doctor will evaluate the wound healing and urination conditions to detect and deal with potential problems in a timely manner.

In summary, the most important role of parents is to observe carefully, strictly implement medical instructions, and communicate in a timely manner. Although we do our best to perform the surgery well, careful post-operative care is a key part of the success of the surgery and the reduction of complications. Please don't worry, we will work with you to ensure a smooth recovery for your child. If you have any questions or concerns, please feel free to contact our medical team.

Source basis or reference source:

These recommendations are based on general consensus and guidelines for clinical practice in pediatric urology. You can refer to the following types of resources for more detailed or authoritative information (please note that specific operations need to follow the personalized guidance of your attending physician):

1. Authoritative medical textbooks: *such as the postoperative management section of the hypospadias chapter in "Campbell-Walsh Urology" or "Ashcraft's Pediatric Surgery".

2. Professional society guidelines/consensus: Guidelines or expert consensus on the diagnosis and treatment of hypospadias issued by the American Urological Association (AUA), the European Association of Urology (EAU), the Pediatric Group of the Urology Branch of the Chinese Medical Association, etc., which will include the principles of postoperative care.

3. Official websites/patient education materials of large children’s hospitals or medical centers: Many well-known children’s hospitals (such as Boston Children’s Hospital, Children’s Hospital of Philadelphia, pediatric surgery/urology departments of large domestic tertiary hospitals) will provide patient and family education information about hypospadias surgery and postoperative care. *For example, you can search for a specific hospital name + "hypospadias postoperative care" or "hypospadias postoperative care." *

4. Peer-reviewed medical literature: Research papers and reviews published in professional medical journals on the prevention and management of complications after hypospadias surgery. *For example, you can search for related keywords in databases such as PubMed. *

Please note: Online information is for reference only and cannot replace the professional judgment and specific guidance of the attending physician. Each child’s situation is different, and the surgical method may also be different. Be sure to follow the instructions of your doctor and nurse in charge.

AI2 (post-hoc identity: ChatGPT-4o)

Chinese original (verbatim output):

如果我是小儿泌尿外科医生，面对家长关于“尿道下裂患儿如何预防术后并发症的发生？”这个问题，我会这样回答（结合循证医学和权威指南）：

医生回答示范（专业+易懂版）

“术后并发症的预防，主要依靠科学的手术方案、良好的护理和规范的随访。我们通常重点关注以下几个方面：

1. 手术技术和个体化方案

- 选择适合孩子的手术方式（如 TIP、Duplay 或带蒂皮瓣等）非常重要，这可以最大限度减少术后瘘、狭窄或畸形的发生。

来源：2022 年欧洲儿科泌尿外科学会（ESPU）尿道下裂指南建议，手术方式应根据尿道下裂类型和解剖特点个体化制定。

2. 术后正确护理

- 遵医嘱使用抗生素预防感染。
- 保持会阴部清洁干燥，避免尿布刺激。
- 妥善固定尿管，防止牵拉或堵塞。

来源：Campbell-Walsh Urology（12th edition）章节指出，术后感染和局部刺激是尿道下裂术后常见并发症诱因，良好的护理至关重要。

3. 控制排尿压力

- 指导家长注意观察排尿情况，避免便秘或排尿困难引起膀胱高压，减少尿道瘘和狭窄风险。

来源：中国医师协会泌尿外科分会小儿泌尿学组专家共识（2021）建议，术后排尿功能评估和干预是减少远期并发症的关键。

4. 定期复诊和长期随访

- 建议术后定期复诊，包括评估外观、尿道通畅度、排尿功能和心理健康。
- 一些并发症如瘘口或狭窄，可能需要数月甚至 1-2 年后才表现，需要密切观察。

来源：ESPU 和 AUA 指南均强调尿道下裂患者需要长期随访至青春期，特别关注功能和心理支持。

总结提醒家长

“简单来说，尿道下裂手术后的并发症是可以通过规范的手术、良好的术后护理和长期随访来最大程度预防的。家长在家中主要注意卫生、观察排尿情况、遵医嘱护理，并定期复诊。如果出现异常情况，比如尿流变细、排尿困难或局部红肿渗液，应及时就诊。”

参考文献出处

1. Springer A, et al. ESPU Guidelines on Hypospadias Management. European Society for Paediatric Urology (ESPU), 2022.

2. Campbell-Walsh Urology, 12th Edition. Chapter: Hypospadias.

3. 中国医师协会泌尿外科分会小儿泌尿学组. 小儿尿道下裂诊治中国专家共识（2021 年版）. 中华泌尿外科杂志, 2021, 42(8): 561-568.

English translation (machine translation; for navigation only):

If I were a pediatric urologist and faced the question from parents about "How to prevent postoperative complications in children with hypospadias?", I would answer this way (combining evidence-based medicine and authoritative guidelines):

Doctor's answer demonstration (professional + easy-to-understand version)

"The prevention of postoperative complications mainly relies on scientific surgical plans, good care and standardized follow-up. We usually focus on the following aspects:

1. Surgical techniques and individualized plans

• It is important to choose a surgical approach that is appropriate for your child (such as TIP, Duplay, or pedicled flap, etc.) to minimize the occurrence of postoperative fistulas, stenosis, or deformities.

Source: The 2022 European Society of Pediatric Urology (ESPU) hypospadias guidelines recommend that surgical methods should be individualized based on the type of hypospadias and anatomical characteristics.

2. Proper postoperative care

• Use antibiotics as directed by your doctor to prevent infection.

• Keep the perineum clean and dry to avoid diaper irritation.

• Secure the urinary catheter properly to prevent traction or blockage.

Source: Campbell-Walsh Urology (12th edition) chapter points out that postoperative infection and local irritation are common complications after hypospadias surgery, and good care is crucial.

3. Control urinary pressure

• Instruct parents to pay attention to urination to avoid bladder hypertension caused by constipation or difficulty urinating, and to reduce the risk of urethral fistula and stricture.

Source: Expert consensus of the Pediatric Urology Group of the Urology Branch of the Chinese Medical Doctor Association (2021) recommends that postoperative urinary function assessment and intervention are the key to reducing long-term complications.

4. Regular follow-up and long-term follow-up

• Regular postoperative follow-up visits are recommended, including assessment of appearance, urethral patency, voiding function, and mental health.

• Some complications, such as fistulas or strictures, may take months or even 1-2 years to manifest and require close observation.

Source: Both the ESPU and AUA guidelines emphasize the need for long-term follow-up of patients with hypospadias into adolescence, with special attention to functional and psychological support.

Summary to remind parents

"Simply speaking, complications after hypospadias surgery can be prevented to the greatest extent through standardized surgery, good postoperative care and long-term follow-up. Parents should pay attention to hygiene at home, observe urination, follow doctor's advice, and follow up with doctors for regular follow-up visits. If there are any abnormalities, such as thinning of the urine stream, difficulty in urinating, or local redness, swelling and oozing, they should see a doctor in time."

Reference source

1. Springer A, et al. ESPU Guidelines on Hypospadias Management. European Society for Paediatric Urology (ESPU), 2022.

2. Campbell-Walsh Urology, 12th Edition. Chapter: Hypospadias.

3. Pediatric Urology Group of the Urology Branch of the Chinese Medical Doctor Association. Chinese expert consensus on the diagnosis and treatment of hypospadias in children (2021 edition). Chinese Journal of Urology, 2021, 42(8): 561-568.

AI3 (post-hoc identity: OpenEvidence)

Chinese original (verbatim output):

预防儿童尿道下裂手术后并发症的措施包括以下几个方面：

1. 手术时机：尽早进行手术可以减少并发症的发生率。研究表明，2 岁以下进行手术的儿童早期并发症较少。[1]

2. 术前准备：术前使用睾酮可能有助于减少远端尿道下裂修复术后的并发症。此外，术前使用广谱抗生素可以减少术后复杂性尿路感染的风险。[2-3]

3. 术后护理：严格的术后护理对于预防并发症至关重要。包括家长教育如何正确护理敷料和导管，确保药物按时服用。术后使用抗生素预防感染也是推荐的措施。[1][3]

4. 手术技术：选择合适的手术技术和术中使用屏障层可以减少并发症的发生。手术时间的安排也很重要，研究发现上午进行的手术并发症风险较高。[4-5]

5. 随访：术后定期随访可以早期发现和处理并发症。对于近端尿道下裂和伴有阴茎弯曲的患者，术后两年内并发症的发生率较高，因此需要更频繁的随访。[6]

通过以上措施，可以有效减少儿童尿道下裂手术后的并发症，提高手术成功率和患者满意度。

1.Age-Dependent Early Complications of Hypospadias Repair: A Single Institutional Experience.

Dale J, Woodward B, Elagami H.Pediatric Surgery International. 2023;39(1):115. doi:10.1007/s00383-023-05388-z.

2.Does Preoperative Testosterone Administration Decrease Complications in Distal Hypospadias Repair With Urethroplasty?.Godlewski KF, Mittal S, Hyacinthe N, et al.The Journal of Urology. 2023;210(2):352-359. doi:10.1097/JU.0000000000003548.

3.Is Prophylactic Antimicrobial Treatment Necessary After Hypospadias Repair?.Meir DB, Livne PM.The Journal of Urology. 2004;171(6 Pt 2):2621-2. doi:10.1097/01.ju.0000124007.55430.d3.

4.Failed Hypospadias in Paediatric Patients.Cimador M, Vallasciani S, Manzoni G, et al.Nature Reviews. Urology. 2013;10(11):657-66. doi:10.1038/nrurol.2013.164.

5.The Impact of Perioperative and Operative Variables on Early Postoperative Complications Following Primary Hypospadias Repair.Storm DW, Lockwood GM, Bonnett MA, et al.Urology. 2022;170:184-188. doi:10.1016/j.urology.2022.08.002.

6.Time to Event Analysis for Post-Hypospadias Repair Complications: A Single-Surgeon Experience.Kim JK, Shiff M, Chua ME, et al.World Journal of Urology. 2021;39(10):3913-3919. doi:10.1007/s00345-021-03689-3.

English translation (machine translation; for navigation only):

Measures to prevent complications after hypospadias surgery in children include the following aspects:

1. Timing of surgery: Surgery as early as possible can reduce the incidence of complications. Research shows that children under 2 years of age who have surgery have fewer early complications. [1]

2. Preoperative preparation: Preoperative use of testosterone may help reduce complications after distal hypospadias repair. In addition, preoperative use of broad-spectrum antibiotics may reduce the risk of postoperative complicated urinary tract infections. [2-3]

3. Post-operative care: Strict post-operative care is essential to prevent complications. This includes parent education on how to properly care for dressings and catheters and ensuring medications are taken on time. The use of antibiotics to prevent infection after surgery is also recommended. [1][3]

4. Surgical technique: Choosing appropriate surgical technique and using a barrier layer intraoperatively can reduce the occurrence of complications. Timing of surgery is also important, with studies finding that surgeries performed in the morning have a higher risk of complications. [4-5]

5. Follow-up: Regular follow-up after surgery can detect and deal with complications early. Patients with proximal hypospadias and concomitant penile curvature have a higher incidence of complications within two years of surgery and require more frequent follow-up. [6]

Through the above measures, complications after hypospadias surgery in children can be effectively reduced, and the success rate of surgery and patient satisfaction can be improved.

1.Age-Dependent Early Complications of Hypospadias Repair: A Single Institutional Experience.

Dale J, Woodward B, Elagami H.Pediatric Surgery International. 2023;39(1):115. doi:10.1007/s00383-023-05388-z.

2.Does Preoperative Testosterone Administration Decrease Complications in Distal Hypospadias Repair With Urethroplasty?.Godlewski KF, Mittal S, Hyacinthe N, et al.The Journal of Urology. 2023;210(2):352-359. doi:10.1097/JU.0000000000003548.

3.Is Prophylactic Antimicrobial Treatment Necessary After Hypospadias Repair?.Meir DB, Livne PM.The Journal of Urology. 2004;171(6 Pt 2):2621-2. doi:10.1097/01.ju.0000124007.55430.d3.

4.Failed Hypospadias in Paediatric Patients.Cimador M, Vallasciani S, Manzoni G, et al.Nature Reviews. Urology. 2013;10(11):657-66. doi:10.1038/nrurol.2013.164.

5.The Impact of Perioperative and Operative Variables on Early Postoperative Complications Following Primary Hypospadias Repair.Storm DW, Lockwood GM, Bonnett MA, et al.Urology. 2022;170:184-188. doi:10.1016/j.urology.2022.08.002.

6.Time to Event Analysis for Post-Hypospadias Repair Complications: A Single-Surgeon Experience.Kim JK, Shiff M, Chua ME, et al.World Journal of Urology. 2021;39(10):3913-3919. doi:10.1007/s00345-021-03689-3.

AI4 (post-hoc identity: Zhipu Qingyan)

Chinese original (verbatim output):

尿道下裂是一种常见的先天性泌尿系统畸形，患儿术后护理对于预防并发症至关重要。以下是一些关键的预防措施：

1.伤口护理

保持干燥清洁：术后伤口应保持干燥和清洁，避免尿液污染。

定期更换敷料：按照医生指导定期更换敷料，不要自行揭掉伤口上的纱布或绷带。

观察感染迹象：密切关注伤口是否有红肿、渗液、发热等感染迹象，如有异常及时就医。

2.抗生素的使用

按时按量服用：根据医生处方，按时按量给孩子服用抗生素，不要随意停药或更改剂量。

注意药物反应：观察孩子是否有药物过敏反应，如皮疹、呼吸困难等，如有应及时告知医生。

3.饮食管理

增加水分摄入：鼓励孩子多喝水，保持充足的水分摄入，有助于预防尿路感染。

清淡易消化：饮食应以清淡、易消化为主，避免辛辣、油腻、刺激性食物。

均衡营养：增加蔬菜和水果的摄入，保证维生素和矿物质的充足供应。

4.活动限制

避免剧烈运动：术后一段时间内，应避免让孩子进行剧烈运动、长时间站立或久坐。

逐渐增加活动量：根据医生的建议，逐渐增加活动量，避免过度劳累。

5.排尿观察

注意排尿情况：观察孩子的排尿情况，注意是否有尿痛、尿频、尿急等症状。

及时就医：如有异常，应及时就医检查。

6.定期复查

遵循医嘱复查：按照医生的安排定期复查，包括伤口愈合情况、尿道功能等。

反馈恢复情况：复查时如实向医生反映孩子的恢复情况，以便及时调整治疗方案。

参考文献：

李强, 张华. 尿道下裂术后并发症的预防与处理[J]. 中华小儿外科杂志, 2010, 31(5): 321-323.

王丽, 李娜. 小儿尿道下裂手术护理要点[J]. 护理实践与研究, 2012, 9(10): 76-77.

通过上述措施，可以有效降低尿道下裂患儿术后并发症的发生风险，促进患儿的顺利康复

English translation (machine translation; for navigation only):

Hypospadias is a common congenital urinary system malformation, and postoperative care of children is crucial to prevent complications. Here are some key precautions:

1. Wound care

Keep Dry and Clean: Postoperative wounds should be kept dry and clean to avoid urine contamination.

Change the dressing regularly: Change the dressing regularly according to the doctor's instructions. Do not remove the gauze or bandage on the wound by yourself.

Observe signs of infection: Pay close attention to the wound for signs of infection such as redness, swelling, exudation, fever, etc. If there are any abnormalities, seek medical attention promptly.

2. Use of antibiotics

Take antibiotics on time and in the right amount: According to the doctor's prescription, give your child antibiotics on time and in the right amount. Do not stop the medication or change the dose at will.

Pay attention to drug reactions: Observe whether the child has drug allergic reactions, such as rash, difficulty breathing, etc. If so, inform the doctor in time.

3. Diet management

Increase fluid intake: Encourage children to drink more water and maintain adequate fluid intake to help prevent urinary tract infections.

Light and easy to digest: The diet should be light and easy to digest, and avoid spicy, greasy, and irritating foods.

Balanced nutrition: Increase the intake of vegetables and fruits to ensure an adequate supply of vitamins and minerals.

4. Activity restrictions

Avoid strenuous exercise: For some time after surgery, your child should avoid strenuous exercise, standing or sitting for long periods of time.

Gradually increase the amount of activity: According to the doctor's advice, gradually increase the amount of activity to avoid overexertion.

5. Observation of urination

Pay attention to urination: Observe your child's urination and pay attention to whether there are symptoms such as painful urination, frequent urination, and urgency.

Seek medical attention promptly: If there are any abnormalities, seek medical attention promptly.

6.Regular review

Follow the doctor's orders for review: Follow the doctor's arrangements for regular reviews, including wound healing, urethral function, etc.

Feedback on the recovery: truthfully report the child's recovery to the doctor during the review so that the treatment plan can be adjusted in a timely manner.

References:

Li Qiang, Zhang Hua. Prevention and treatment of complications after hypospadias surgery[J]. Chinese Journal of Pediatric Surgery, 2010, 31(5): 321-323.

Wang Li, Li Na. Key points of nursing care for hypospadias surgery in children [J]. Nursing Practice and Research, 2012, 9(10): 76-77.

Through the above measures, the risk of postoperative complications in children with hypospadias can be effectively reduced and the smooth recovery of children can be promoted.

AI5 (post-hoc identity: DeepSeek)

Chinese original (verbatim output):

作为小儿泌尿外科医生，关于尿道下裂术后并发症的预防，我会从以下方面向家长解释，并提供科学依据：

一、术前准备

1. 激素治疗（针对严重病例）

对于阴茎发育不良或重度尿道下裂患儿，术前局部或全身激素（如睾酮、HCG）可改善阴茎尺寸和血管化，降低手术难度和术后并发症风险。

依据：欧洲泌尿外科协会（EAU）指南建议术前激素治疗可能减少术后尿道瘘发生（EAU Paediatric Urology Guidelines, 2023）。

二、术中技术要点

1. 精细手术操作

采用保留血供的皮瓣技术（如 Snodgrass 术式）和分层缝合，减少组织缺血和张力，可显著降低尿道瘘和狭窄风险。

依据：多项研究证实 Snodgrass 术式尿道瘘发生率低于 5%（BJU International, 2019）。

2. 导尿管选择

使用硅胶导尿管（而非乳胶）减少尿道刺激，留置时间根据术式调整（通常 5-14 天）。

依据：美国儿科学会（AAP）推荐硅胶管降低感染风险（Pediatrics, 2014）。

三、术后护理关键

1. 导尿管管理

- 确保导尿管固定牢固，避免牵拉或移位。

- 每日冲洗生理盐水（按医嘱）防止血块堵塞。

依据：临床研究显示导尿管堵塞是术后出血和感染的主要诱因（Journal of Pediatric Urology, 2020）。

2. 伤口护理

- 使用抗生素软膏（如莫匹罗星）涂抹伤口，每日 2 次。

- 保持敷料干燥，排尿后生理盐水清洁会阴部。

依据：随机对照试验证实局部抗生素可降低感染率（The Lancet Child & Adolescent Health, 2021）。

3. 活动限制

术后 2 周内避免剧烈运动（如骑车、跑跳），防止伤口裂开。

4. 饮食调整

多饮水（每日 1.5-2L）稀释尿液，减少刺激；避免辛辣食物。

四、并发症监测与随访

1. 早期识别症状

- 尿道瘘：尿液从阴茎非尿道口位置渗出。

- 狭窄：排尿费力、尿线变细。

- 感染：发热、伤口红肿渗液。

建议：出现上述症状需立即复诊。

2. 定期复查

- 术后 1 个月、3 个月、6 个月随访，评估排尿功能和伤口愈合。

- 必要时行尿流率检测或尿道造影。

依据：Meta 分析显示规律随访可减少二次手术率（Cochrane Database, 2018）。

家长常见误区纠正

- 误区：导尿管拔除后即可正常活动。

纠正：需逐步恢复活动，1 个月内避免挤压伤口。

-误区：尿频、尿急是正常现象。

纠正：可能提示感染或狭窄，需及时检查。

参考文献

1. EAU Guidelines on Paediatric Urology (2023)

2. Snodgrass W, et al. "Urethroplasty for hypospadias: A systematic review." *BJU International* (2019).

3. American Academy of Pediatrics. "Hypospadias management guidelines." *Pediatrics* (2014).

4. Springer A, et al. "Risk factors for complications in hypospadias surgery." *Journal of Pediatric Urology* (2020).

通过以上措施，可将术后并发症风险控制在 5%-15%（依术式和病情不同）。家长需与主刀医生保持沟通，严格遵循个性化护理方案。

评价指标及定义

请您从文本质量、相关性、适用性、来源可靠性、可理解性、可操作性 6 个方面进行评价，排在越前面意味着其在各指标综合考量下分值越高。

1.质量：衡量 AI 答案对医疗疑问的解决效果，涵盖医学准确性、细节完整性以及提供医学见解的深度，判断其能否为提问者提供有价值、全面且深入的医学指导。

请将 5 个 AI 回答文本按照质量评价进行排序，例如 AI1＞AI2＞AI3＞AI4＞AI5 。

您的作答：　　＞　　＞　　＞　　＞

2.相关性：判断 AI 答案与医学问题的契合程度，包括是否紧扣主题，是否全面涉及医学问题的各个方面，以及所提供信息与医疗问题的关联紧密性。

请将 5 个 AI 回答文本按照相关性进行排序，例如 AI1＞AI2＞AI3＞AI4＞AI5 。

您的作答：　　＞　　＞　　＞　　＞

3.适用性：评估 AI 答案在询问所描述的医学背景和实际医疗场景中的可应用程度，看其是否能直接用于解决实际问题，或是需要大量调整修改。

请将 5 个 AI 回答文本按照适用性进行排序，例如 AI1＞AI2＞AI3＞AI4＞AI5 。

您的作答：　　＞　　＞　　＞　　＞

4.来源可靠性：考查 AI 答案所依据来源的可信度（是否有虚假信息或来源不详细等），包括参考文献的权威性、输出内容与文献的关联度，以及信息来源渠道是否专业、经过严格审核，以此判断答案是否准确、真实且无过时信息。

请将 5 个 AI 回答文本按照来源可靠性进行排序，例如 AI1＞AI2＞AI3＞AI4＞AI5 。

您的作答：　　＞　　＞　　＞　　＞

5.可理解性：评价 AI 答案在语言使用、内容组织以及辅助工具运用等方面，是否便于用户理解，包括语言是否通俗易懂、术语有无定义、结构是否清晰、有无视觉辅助或摘要帮助理解等。

请将 5 个 AI 回答文本按照可理解性进行排序，例如 AI1＞AI2＞AI3＞AI4＞AI5 。

您的作答：　　＞　　＞　　＞　　＞

6.可操作性：判断 AI 答案是否明确指出用户可采取的操作，是否将操作细化为清晰步骤，以及是否提供实用工具辅助用户在实际场景中采取有效行动。

请将 5 个 AI 回答文本按照可操作性进行排序，例如 AI1＞AI2＞AI3＞AI4＞AI5 。

您的作答：　　＞　　＞　　＞　　＞

根据以上内容进行综合排序：：　　＞　　＞　　＞　　＞

English translation (machine translation; for navigation only):

As a pediatric urologist, I will explain to parents the following aspects about the prevention of complications after hypospadias surgery and provide scientific basis:

1. Preoperative preparation

1. Hormone treatment (for severe cases)

For children with penile dysplasia or severe hypospadias, preoperative local or systemic hormones (such as testosterone, HCG) can improve penile size and vascularization, reduce surgical difficulty and the risk of postoperative complications.

Basis: European Association of Urology (EAU) guidelines recommend that preoperative hormonal therapy may reduce the occurrence of postoperative urethral fistulas (EAU Paediatric Urology Guidelines, 2023).

2. Key points of intraoperative techniques

1. Delicate surgical operations

Using blood supply-preserving flap technology (such as the Snodgrass procedure) and layered suturing to reduce tissue ischemia and tension can significantly reduce the risk of urethral fistulas and strictures.

Basis: Multiple studies have confirmed that the incidence of urethral fistula after Snodgrass surgery is less than 5% (BJU International, 2019).

2. Catheter selection

Use a silicone catheter (rather than latex) to reduce urethral irritation, and the indwelling time is adjusted according to the surgical procedure (usually 5-14 days).

Basis: The American Academy of Pediatrics (AAP) recommends silicone tubes to reduce the risk of infection (Pediatrics, 2014).

3. Key Postoperative Care

1. Catheter management

- Make sure the catheter is securely fixed to avoid pulling or shifting.

- Daily flushing with normal saline (as directed by your doctor) to prevent blood clots.

Basis: Clinical studies show that urinary catheter obstruction is the main cause of postoperative bleeding and infection (Journal of Pediatric Urology, 2020).

2. Wound care

- Apply antibiotic ointment (such as mupirocin) to the wound twice daily.

- Keep the dressing dry and clean the perineum with saline after urination.

Rationale: Randomized controlled trials confirm that topical antibiotics reduce infection rates (The Lancet Child & Adolescent Health, 2021).

3. Activity restrictions

Avoid strenuous exercise (such as cycling, running and jumping) within 2 weeks after surgery to prevent wound dehiscence.

4. Diet modification

Drink more water (1.5-2L per day) to dilute urine and reduce irritation; avoid spicy food.

4. Complication monitoring and follow-up

1. Early identification of symptoms

- Urethral fistula: leakage of urine from the penis at a location other than the urethral opening.

- Stricture: difficulty urinating and thinning of the urine line.

- Infection: fever, redness, swelling and drainage from the wound.

Recommendation: If you experience the above symptoms, you need to seek medical attention immediately.

2. Regular review

- Follow-up at 1 month, 3 months, and 6 months after surgery to evaluate urinary function and wound healing.

- Perform uroflowmetry or urethrography if necessary.

Basis: Meta analysis shows that regular follow-up can reduce the rate of secondary surgery (Cochrane Database, 2018).

Common misunderstandings corrected by parents

- Myth: Normal activities can be resumed after the urinary catheter is removed.

Correction: It is necessary to gradually resume activities and avoid squeezing the wound within 1 month.

-Myth: Frequent urination and urgency are normal phenomena.

Correction: It may indicate infection or stenosis and requires timely examination.

References

1. EAU Guidelines on Paediatric Urology (2023)

2. Snodgrass W, et al. "Urethroplasty for hypospadias: A systematic review." *BJU International* (2019).

3. American Academy of Pediatrics. "Hypospadias management guidelines." *Pediatrics* (2014).

4. Springer A, et al. "Risk factors for complications in hypospadias surgery." *Journal of Pediatric Urology* (2020).

Through the above measures, the risk of postoperative complications can be controlled to 5%-15% (depending on the type of surgery and the condition). Parents need to maintain communication with the surgeon and strictly follow the personalized care plan.

Evaluation indicators and definitions

Please evaluate it from six aspects: text quality, relevance, applicability, source reliability, understandability, and operability. The higher the ranking, the higher the score based on the comprehensive consideration of each indicator.

1. Quality: Measure the effectiveness of AI answers in solving medical questions, covering medical accuracy, completeness of details and the depth of medical insights provided, to determine whether it can provide valuable, comprehensive and in-depth medical guidance to the questioner.

Please sort the 5 AI answer texts according to quality evaluation, for example, AI1>AI2>AI3>AI4>AI5.

Your answer: ＞＞＞＞

2. Relevance: Determine the degree of fit between the AI answer and the medical problem, including whether it sticks to the topic, whether it comprehensively covers all aspects of the medical problem, and how closely the information provided is related to the medical problem.

Please sort the 5 AI answer texts according to their relevance, for example, AI1>AI2>AI3>AI4>AI5.

Your answer: ＞＞＞＞

3. Applicability: Evaluate the applicability of the AI answers in the medical background and actual medical scenarios described in the query to see whether it can be directly used to solve practical problems, or whether it requires extensive adjustments and modifications.

Please sort the 5 AI answer texts according to their applicability, for example, AI1>AI2>AI3>AI4>AI5.

Your answer: ＞＞＞＞

4. Source reliability: Examine the credibility of the sources on which the AI answers are based (whether there is false information or unclear sources, etc.), including the authority of the references, the relevance of the output content and the literature, and whether the information source channels are professional and rigorously reviewed to determine whether the answers are accurate, authentic and free of outdated information.

Please sort the 5 AI answer texts according to source reliability, for example, AI1>AI2>AI3>AI4>AI5.

Your answer: ＞ ＞ ＞ ＞

5. Comprehensibility: Evaluate whether the AI answers are easy for users to understand in terms of language use, content organization, and use of auxiliary tools, including whether the language is easy to understand, whether terms are defined, whether the structure is clear, and whether there are visual aids or summaries to help understanding, etc.

Please sort the 5 AI answer texts according to their comprehensibility, for example, AI1>AI2>AI3>AI4>AI5.

Your answer: ＞ ＞ ＞ ＞

6. Operability: Determine whether the AI answer clearly points out the actions that the user can take, whether the operation is refined into clear steps, and whether it provides practical tools to assist the user in taking effective actions in actual scenarios.

Please sort the 5 AI answer texts according to operability, for example, AI1>AI2>AI3>AI4>AI5.

Your answer: ＞ ＞ ＞ ＞

Comprehensive sorting based on the above content:: ＞ ＞ ＞ ＞

Question 7 (Medium risk; Follow-up / Prognosis)

Original prompt (Chinese):

如果你是一名小儿泌尿外科的医生，家长问到“尿道下裂手术对患儿未来的生殖功能和泌尿系统功能会有怎样的影响？”问题，你应该怎么回答？并给出来源依据或参考出处。

Prompt translation (English):

If you are a pediatric urologist, and a parent asks, "How will hypospadias surgery affect the child's future reproductive and urinary function?", how should you answer? Please cite your sources or references.

Per-question unblinding key for Q7:

Blinded label	Model identity
AI1	Zhipu Qingyan
AI2	Gemini-2.5-Pro
AI3	ChatGPT-4o
AI4	OpenEvidence
AI5	DeepSeek

AI1 (post-hoc identity: Zhipu Qingyan)

Chinese original (verbatim output):

1.问题理解

家长询问尿道下裂手术对患儿未来生殖功能和泌尿系统功能的潜在影响。

2.尿道下裂概述

尿道下裂是一种常见的先天性泌尿系统畸形，影响排尿功能和生殖器外观。

3.手术目的

修复尿道下裂，恢复正常的排尿功能。

改善阴茎的外观，促进患儿的心理健康发展。

4.手术效果

积极影响：

大多数患儿术后能够恢复正常排尿功能和生殖器外观。

提高生育能力，尤其是伴随的阴茎弯曲和睾丸下降不全等问题得到纠正后。

潜在并发症：

尿瘘：发生率约为 5%至 10%，可能需要进一步手术治疗。

尿道狭窄：发生率类似，也可通过后续治疗纠正。

5.长期影响

根据现有研究，尿道下裂手术后患儿的生殖功能和泌尿系统功能通常正常。

每个患儿的情况独特，手术效果可能有所不同。

6.家长建议

与孩子的医生充分沟通，了解手术的风险和益处。

密切跟踪患儿的术后恢复情况，必要时进行进一步治疗。

尿道下裂手术通常能够有效修复尿道下裂，恢复正常的排尿功能和生殖器外观。大多数患儿术后生殖功能和泌尿系统功能正常，但像所有手术一样，可能存在并发症，如尿瘘或尿道狭窄，这些并发症的发生率相对较低，且可通过进一步治疗纠正。家长应与医生充分沟通，了解手术的风险和益处，以做出最适合孩子的决定。

English translation (machine translation; for navigation only):

1. Problem understanding

Parents inquire about the potential impact of hypospadias surgery on the child's future reproductive and urinary function.

2. Overview of hypospadias

Hypospadias is a common congenital urinary system malformation that affects urinary function and genital appearance.

3. Purpose of surgery

Repair hypospadias and restore normal urinary function.

Improve the appearance of the penis and promote the mental health development of children.

4. Surgical effect

Positive impact:

Most children recover normal urinary function and genital appearance after surgery.

Improves fertility, especially after the accompanying problems of penile curvature and incomplete testicular descent are corrected.

Potential complications:

Urinary fistula: occurs in approximately 5% to 10% of cases and may require further surgical treatment.

Urethral stricture: occurs at a similar rate and can also be corrected with subsequent treatment.

5. Long-term effects

According to available research, reproductive and urinary function in children after hypospadias surgery is usually normal.

Each child's situation is unique and the results of surgery may vary.

6. Parental suggestions

Communicate fully with your child's doctor about the risks and benefits of surgery.

The patient's postoperative recovery will be closely followed and further treatment will be provided if necessary.

Hypospadias surgery is often effective in repairing hypospadias and restoring normal urinary function and genital appearance. Most children have normal reproductive and urinary function after surgery, but like all surgeries, there may be complications, such as urinary fistulas or urethral strictures. The incidence of these complications is relatively low and can be corrected with further treatment. Parents should fully communicate with their doctor to understand the risks and benefits of surgery to make the best decision for their child.

AI2 (post-hoc identity: Gemini-2.5-Pro)

Chinese original (verbatim output):

您好，非常理解您对孩子未来健康的关心，这是一个非常重要的问题。尿道下裂手术的目标不仅仅是修复外观，更重要的是确保孩子未来能够拥有正常的泌尿功能和生殖功能。让我为您详细解释一下：

手术的主要目标：

尿道下裂手术的核心目标是：

1. 矫正阴茎下弯 (Chordee Correction):确保阴茎在勃起时能够伸直，这是未来正常性功能的基础。

2. 重建尿道 (Urethroplasty):将尿道延伸至阴茎头顶端（或尽可能接近顶端），形成一个功能良好的新尿道。

3. 重建阴茎头 (Glanuloplasty): 使阴茎头外观更接近正常。

4. 确保外观满意: 尽可能达到美观的阴茎外形。

对未来生殖功能的影响：

1. 勃起功能: 成功矫正阴茎下弯是关键。如果手术成功，阴茎能够正常伸直，那么大多数患儿成年后的勃起功能不会受到显著影响。如果术后仍有残余弯曲，可能会影响性生活。

2. 射精功能: 手术旨在重建一条通畅的尿道。如果新尿道通畅，没有发生严重的狭窄，那么精液通常可以正常射出。严重的尿道狭窄可能会影响射精。

3. 生育能力: 尿道下裂手术本身通常不直接影响睾丸产生精子的能力。生育能力主要取决于睾丸功能是否正常。需要注意的是，一小部分尿道下裂患儿（尤其是严重类型）可能同时合并有其他泌尿生殖系统异常，例如隐睾，这可能会影响生育能力，但这通常与尿道下裂本身相关，而非手术直接导致。总体而言，大多数 接受尿道下裂修复术的男性具有正常的生育潜力。

对未来泌尿系统功能的影响：

1. 排尿姿势与尿流: 手术成功的主要标志之一就是孩子能够站立排尿，并且尿流能够向前喷射，而不是向下或分叉。绝大多数患儿通过一次或多次手术可以达到这个目标。

2. 控尿能力: 尿道下裂手术通常不直接影响控制排尿的膀胱颈部和括约肌。因此，手术本身一般不会导致尿失禁。

3. 潜在的远期并发症: 虽然手术目标是实现正常功能，但任何手术都有可能出现并发症，有些可能在术后多年才显现：

  * 尿道狭窄 (Urethral Stricture): 新建的尿道部分发生缩窄，导致排尿困难、尿流变细、排尿费力等。这是最需要关注的远期并发症之一。

  * 尿瘘 (Urethrocutaneous Fistula): 尿液从新建尿道以外的其他开口漏出。虽然多在术后早期发现，但少数也可能晚期出现。

  * 尿道憩室 (Urethral Diverticulum): 新建尿道局部膨出，可能导致尿液残留、滴沥和感染。

  * 尿道口狭窄 (Meatal Stenosis): 阴茎头的新尿道口变窄。

总结与建议：

总的来说，现代小儿泌尿外科的尿道下裂修复技术已经非常成熟，绝大多数 接受手术的孩子在成年后可以获得满意的泌尿功能（站立排尿、尿流通畅）和生殖功能（正常勃起、射精和生育能力）。

但是，手术效果确实受到多种因素影响，包括：

* 尿道下裂的严重程度: 轻型尿道下裂的成功率更高，远期并发症风险更低。重型或复杂型尿道下裂可能需要分期手术，并发症风险相对较高。

* 手术时机和手术技术: 合适的手术时机和经验丰富的外科医生采用的精细技术至关重要。

* 术后护理和随访: 良好的术后护理和长期的定期随访（包括青春期和成年后）对于及时发现并处理潜在问题非常重要。

作为医生，我们会根据您孩子的具体情况，选择最合适的手术方案，并尽最大努力达到最佳的功能和外观效果。术后，我们会安排定期的复查，不仅是观察近期的愈合情况，更重要的是长期跟踪排尿和未来的功能状况。请您放心，我们会和您一起努力，为孩子的健康未来保驾护航。

来源依据与参考:

1. 《坎贝尔-沃尔什泌尿外科学》（Campbell-Walsh Urology）： 这是泌尿外科领域的权威教科书，其中有专门章节详细论述尿道下裂的诊断、手术治疗及长期预后。（具体可查阅最新版关于 Hypospadias 的章节）

2. UpToDate 临床顾问: 这是一个循证医学临床决策支持资源，其中关于 "Hypospadias" 的专题综述了手术目标、技术、并发症和长期结局。（需要订阅访问，但其结论基于大量文献回顾）

3. 美国泌尿外科学会 (AUA) / 欧洲泌尿外科学会 (EAU) 指南 这些专业组织的临床指南会根据最新的研究证据，提供关于尿道下裂处理的建议，包括对功能预后的评估。

4. 相关医学文献: 大量的临床研究论文发表在《Journal of Urology》、《Journal of Pediatric Urology》、《Urology》等专业期刊上，探讨了不同手术方式的长期功能结果和并发症发生率。例如，搜索 "long-term outcomes hypospadias repair", "fertility after hypospadias repair", "urinary function after hypospadias repair" 可以找到相关研究。

请注意，以上信息是一般性的解释。您孩子的具体情况和预后，还需要结合他的尿道下裂类型、身体状况以及手术的具体细节来评估。我们会在后续的诊疗过程中与您进行更详细的沟通。

English translation (machine translation; for navigation only):

Hello, I understand your concerns about your child's future health. This is a very important issue. The goal of hypospadias surgery is not only to repair the appearance, but more importantly, to ensure that the child can have normal urinary and reproductive functions in the future. Let me explain it to you in detail:

Main goals of surgery:

The core goals of hypospadias surgery are:

1. Correction of penile curvature (Chordee Correction): ensuring that the penis can straighten when erect, which is the basis for normal sexual function in the future.

2. Reconstruction of the urethra (Urethroplasty): Extend the urethra to the top of the glans penis (or as close to the top as possible) to form a new urethra with good function.

3. Reconstruction of the glans penis (Glanuloplasty): Make the appearance of the glans penis closer to normal.

4. Ensure satisfactory appearance: Achieve a beautiful penis shape as much as possible.

Effects on future reproductive function:

1. Erectile function: Successfully correcting penile downward curvature is key. If the surgery is successful and the penis can straighten normally, most children's erectile function will not be significantly affected as adults. If there is still residual curvature after surgery, it may affect your sex life.

2. Ejaculatory function: The surgery aims to reconstruct a smooth urethra. If the new urethra is clear and no severe stricture has occurred, semen can usually be ejaculated normally. Severe urethral stricture may interfere with ejaculation.

3. Fertility: Hypospadias surgery itself usually does not directly affect the testicles’ ability to produce sperm. Fertility depends primarily on normal testicular function. It should be noted that a small proportion of children with hypospadias (especially severe types) may also have other urogenital abnormalities, such as cryptorchidism, which may affect fertility, but this is usually related to hypospadias itself and is not directly caused by surgery. Overall, most men who undergo hypospadias repair have normal reproductive potential.

Effects on future urinary system function:

1. Urinating posture and urine flow: One of the main signs of successful surgery is that the child is able to stand up to urinate and that the urine flow jets forward instead of downward or branching. Most children can achieve this goal with one or more surgeries.

2. Urinary continence: Hypospadias surgery usually does not directly affect the bladder neck and sphincter muscles that control urination. Therefore, the surgery itself generally does not cause urinary incontinence.

3. Potential long-term complications: Although the goal of surgery is to achieve normal function, complications may occur with any surgery, some of which may not appear until many years after surgery:

* Urethral Stricture: The newly constructed part of the urethra is narrowed, causing difficulty in urinating, thinning of the urine stream, and labored urination. This is one of the most concerning long-term complications.

* Urethrocutaneous Fistula: Urine leaks from an opening other than the newly created urethra. Although it is often detected early after surgery, a few may also appear late.

* Urethral Diverticulum (Urethral Diverticulum): A local bulge in the new urethra, which may lead to urine retention, dripping and infection.

* Meatal Stenosis: Narrowing of the new urethral opening at the glans penis.

Summary and suggestions:

In general, hypospadias repair technology in modern pediatric urology is very mature, and the vast majority of children who undergo surgery can achieve satisfactory urinary function (standing urination, smooth urine flow) and reproductive function (normal erection, ejaculation, and fertility) in adulthood.

However, the effectiveness of surgery does depend on a variety of factors, including:

* Severity of hypospadias: Mild cases of hypospadias have a higher success rate and lower risk of long-term complications. Severe or complex hypospadias may require staged surgery, with a relatively high risk of complications.

* Timing of surgery and technique: Proper timing of surgery and meticulous technique by an experienced surgeon are crucial.

* Post-operative care and follow-up: Good post-operative care and regular follow-up over the long term (including adolescence and adulthood) are important to detect and deal with potential problems in a timely manner.

As doctors, we will choose the most appropriate surgical option based on your child's specific situation and do our best to achieve the best functional and cosmetic results. After surgery, we will arrange regular follow-up

visits, not only to observe the recent healing, but more importantly, to track urination and future functional status in the long term. Please rest assured that we will work with you to protect your child's healthy future.

Source basis and reference:

1. "Campbell-Walsh Urology": This is the authoritative textbook in the field of urology. It has special chapters that discuss the diagnosis, surgical treatment and long-term prognosis of hypospadias in detail. (For details, please refer to the latest version of the chapter on Hypospadias)

2. UpToDate Clinical Advisor: This is an evidence-based clinical decision support resource with a special topic on "Hypospadias" that reviews surgical goals, techniques, complications, and long-term outcomes. (Subscription required to access, but conclusions based on extensive literature review)

3. American Urological Association (AUA)/European Association of Urology (EAU) Guidelines Clinical guidelines from these professional organizations provide recommendations on the management of hypospadias, including assessment of functional prognosis, based on the latest research evidence.

4. Relevant medical literature: A large number of clinical research papers have been published in professional journals such as "Journal of Urology", "Journal of Pediatric Urology", and "Urology", discussing the long-term functional results and complication rates of different surgical methods. For example, search for "long-term outcomes hypospadias repair", "fertility after hypospadias repair", "urinary function after hypospadias repair" to find relevant studies.

Please note that the above information is a general interpretation. Your child's specific situation and prognosis also need to be evaluated in light of his type of hypospadias, physical condition, and the specific details of the surgery. We will communicate with you in more detail during the subsequent diagnosis and treatment process.

AI3 (post-hoc identity: ChatGPT-4o)

Chinese original (verbatim output):

如果我是小儿泌尿外科医生，面对家长这个问题，我会这样回答，尽量做到专业、诚恳且易于理解：

医生回答示范：

“这是家长非常关心、也是我们非常重视的问题。总体来说，大多数尿道下裂的孩子在接受规范的手术治疗后，未来的排尿功能和生殖功能是可以达到正常或接近正常的状态的。

手术的主要目的是重建尿道、恢复正常的尿道开口位置、改善阴茎外观，并保证良好的排尿功能。大部分轻中度尿道下裂的患者，在儿童期或青春期复查时，排尿射流、方向、速度等都接近正常。重度尿道下裂的患者，虽然手术难度大一些，但如果接受了个体化和多阶段的良好治疗，排尿功能通常也能获得较好的恢复。

至于生殖功能——主要涉及勃起、性交、射精及生育——现有研究和长期随访数据显示，绝大多数患者成年后勃起功能不受明显影响，可以正常性生活。部分重度尿道下裂患者可能因阴茎长度、弯曲矫正程

度或尿道并发症等因素影响性生活质量，但这并不是普遍现象，而且可以通过后续的干预改善。生育能力总体上良好，但若存在精液排出异常或射精困难，需要进一步评估和治疗。

需要说明的是，术后可能存在的并发症（如尿道瘘、狭窄、阴茎弯曲复发）可能会影响排尿或性生活质量，但这些大多数是可以通过进一步的修复手术解决的。

参考证据与文献依据：

1. Springer, A. (2014). Assessment of outcome in hypospadias surgery – A review. Frontiers in Pediatrics, 2, 2.

总结了尿道下裂术后排尿和生殖功能的长期评估，认为大多数患者排尿和性功能良好，但重度患者可能存在一定的功能障碍，需要个体化评估。

2. Long-term outcomes study:

Rynja SP, et al. (2012). Long-term followup of male patients with hypospadias: functional and cosmetic results. The Journal of Urology, 188(4 Suppl), 1487-1492.

显示大多数患者成年后对排尿和性功能满意，但重度尿道下裂的患者存在一定比例的性生活问题或生育问题。

3. Guidelines Reference:

European Association of Urology (EAU) Guidelines on Paediatric Urology (最新版)

指出手术目标包括恢复正常排尿和生殖功能，强调随访和功能评估的重要性。

结论性的安慰：

“当然，每个孩子的情况都不完全一样，我们会根据他具体的情况进行个体化的治疗和长期随访。家长不需要过度担心，只要规范手术和定期复查，大多数孩子未来的生活质量、排尿和生殖功能都能达到良好的状态。”

English translation (machine translation; for navigation only):

If I were a pediatric urologist and faced with this question from parents, I would answer this question, trying to be professional, sincere and easy to understand:

Doctor’s sample answer:

"This is an issue that parents are very concerned about and that we attach great importance to. Generally speaking, after most children with hypospadias receive standard surgical treatment, their future urinary and reproductive functions can reach normal or near-normal status.

The main purpose of surgery is to reconstruct the urethra, restore the normal urethral opening position, improve the appearance of the penis, and ensure good urinary function. For most patients with mild to moderate hypospadias, the urinary jet, direction, speed, etc. are close to normal when they are reviewed in childhood or adolescence. Although patients with severe hypospadias have more difficult surgery, their urinary function can usually be restored well if they receive good individualized and multi-stage treatment.

As for reproductive function - which mainly involves erection, sexual intercourse, ejaculation and fertility - existing research and long-term follow-up data show that the vast majority of patients have no significant impact on their erectile function as adults and can have normal sex lives. Some patients with severe hypospadias may have quality of sexual life affected by factors such as penis length, degree of curvature correction, or urethral complications, but this is not a common phenomenon and can be improved through subsequent intervention. Fertility is generally good, but any abnormal semen discharge or difficulty ejaculating requires further evaluation and treatment.

It should be noted that possible complications after surgery (such as urethral fistula, stricture, recurrence of penile curvature) may affect urination or sexual life quality, but most of these can be solved through further repair surgery.

Reference evidence and literature basis:

1. Springer, A. (2014). Assessment of outcome in hypospadias surgery – A review. Frontiers in Pediatrics, 2, 2.

This paper summarizes the long-term evaluation of urinary and reproductive functions after hypospadias surgery and concludes that most patients have good urinary and sexual functions, but severe patients may have certain functional impairments and require individualized evaluation.

2. Long-term outcomes study:

Rynja SP, et al. (2012). Long-term followup of male patients with hypospadias: functional and cosmetic results. The Journal of Urology, 188(4 Suppl), 1487-1492.

It has been shown that most patients are satisfied with urination and sexual function in adulthood, but a certain proportion of patients with severe hypospadias have sexual life problems or fertility problems.

3. Guidelines Reference:

European Association of Urology (EAU) Guidelines on Paediatric Urology (latest version)

It was pointed out that the goals of surgery include restoration of normal urinary and reproductive function, emphasizing the importance of follow-up and functional assessment.

Concluding words of comfort:

"Of course, every child's situation is different, and we will carry out individualized treatment and long-term follow-up based on his specific situation. Parents do not need to worry too much. As long as the surgery is standardized and regular reviews are performed, most children can achieve good quality of life, urinary and reproductive functions in the future."

AI4 (post-hoc identity: OpenEvidence)

Chinese original (verbatim output):

儿童尿道下裂手术对未来生殖功能和泌尿系统功能的影响是家长们常常关心的问题。根据现有的医学文献，手术后的长期结果在功能和美观方面存在一定的变异。

首先，尿道下裂修复手术通常能够恢复正常的尿道功能，但部分患者可能会出现排尿功能障碍。研究显示，约 22.1%的患者在手术后存在排尿功能不佳的情况，尽管很少有患者有显著的残余尿量。此外，手术后尿道狭窄和尿道瘘管的发生率也较高，尤其是在初次手术年龄较小的患者中。[1-2]

在生殖功能方面，手术后的性功能和生殖能力通常是良好的。大多数患者在青春期和成年期的性功能与正常人群相当，勃起和射精功能问题较少见，约 10%的患者可能会出现轻度的勃起或射精问题。然而，严重的尿道下裂患者可能会有较高的生殖功能障碍风险，尤其是伴有隐睾的患者。[3-4]

总体而言，尿道下裂手术后的患者在美观和功能方面的满意度较高，但需要长期随访以监测和处理可能出现的并发症。手术后的长期随访对于确保患者的泌尿和生殖健康至关重要。[5-6]

1.Adolescent and Young Adult Urogenital Outcome Following Childhood Hypospadias Repair: Perfection Revisited.Tack LJW, Springer A, Riedl S, et al.The Journal of Urology. 2021;206(3):734-744. doi:10.1097/JU.0000000000001869.

2.Better Defining the Spectrum of Adult Hypospadias: Examining the Effect of Childhood Surgery on Adult Presentation.Hoy NY, Rourke KF.Urology. 2017;99:281-286. doi:10.1016/j.urology.2016.07.057.

3.Psychosexual Outcome, Sexual Function, and Long-Term Satisfaction of Adolescent and Young Adult Men After Childhood Hypospadias Repair.Tack LJW, Springer A, Riedl S, et al.The Journal of Sexual Medicine. 2020;17(9):1665-1675. doi:10.1016/j.jsxm.2020.04.002.

4.Long-Term Outcome of Pituitary-Gonadal Axis and Gonadal Growth in Patients With Hypospadias at Puberty.Moriya K, Mitsui T, Tanaka H, Nakamura M, Nonomura K.The Journal of Urology. 2010;184(4 Suppl):1610-4. doi:10.1016/j.juro.2010.04.022.

5.Long-Term Followup of Men Born With Hypospadias: Urological and Cosmetic Results.

Örtqvist L, Fossum M, Andersson M, et al.The Journal of Urology. 2015;193(3):975-81. doi:10.1016/j.juro.2014.09.103.

6.Do New Complications Develop During Puberty After Childhood Hypospadias Repair?.Snodgrass W, Bush N.The Journal of Urology. 2022;208(3):696-701. doi:10.1097/JU.0000000000002738.

English translation (machine translation; for navigation only):

Parents are often concerned about the impact of children's hypospadias surgery on future reproductive and urinary system functions. According to the available medical literature, there is some variability in long-term outcomes after surgery in terms of function and aesthetics.

First, hypospadias repair surgery usually restores normal urethral function, but some patients may experience voiding dysfunction. Studies have shown that about 22.1% of patients have poor urinary function after surgery, although few patients have significant residual urine volume. In addition, the incidence of urethral strictures and urethral fistulas is higher after surgery, especially in patients who are younger at initial surgery. [1-2]

In terms of reproductive function, sexual function and reproductive ability after surgery are usually good. The sexual function of most patients during adolescence and adulthood is comparable to that of the normal population. Problems with erectile and ejaculation functions are less common, and about 10% of patients may experience mild erection or ejaculation problems. However, patients with severe hypospadias may be at higher risk for reproductive dysfunction, especially those with cryptorchidism. [3-4]

Overall, patient satisfaction after hypospadias surgery is high in terms of aesthetics and function, but long-term follow-up is needed to monitor and manage possible complications. Long-term follow-up after surgery is critical to ensure the patient's urinary and reproductive health. [5-6]

1.Adolescent and Young Adult Urogenital Outcome Following Childhood Hypospadias Repair: Perfection Revisited.Tack LJW, Springer A, Riedl S, et al.The Journal of Urology. 2021;206(3):734-744. doi:10.1097/JU.0000000000001869.

2.Better Defining the Spectrum of Adult Hypospadias: Examining the Effect of Childhood Surgery on Adult Presentation.Hoy NY, Rourke KF.Urology. 2017;99:281-286. doi:10.1016/j.urology.2016.07.057.

3.Psychosexual Outcome, Sexual Function, and Long-Term Satisfaction of Adolescent and Young Adult Men After Childhood Hypospadias Repair.Tack LJW, Springer A, Riedl S, et al.The Journal of Sexual Medicine. 2020;17(9):1665-1675. doi:10.1016/j.jsxm.2020.04.002.

4.Long-Term Outcome of Pituitary-Gonadal Axis and Gonadal Growth in Patients With Hypospadias at Puberty.Moriya K, Mitsui T, Tanaka H, Nakamura M, Nonomura K.The Journal of Urology. 2010;184(4 Suppl):1610-4. doi:10.1016/j.juro.2010.04.022.

5.Long-Term Followup of Men Born With Hypospadias: Urological and Cosmetic Results.

Örtqvist L, Fossum M, Andersson M, et al.The Journal of Urology. 2015;193(3):975-81. doi:10.1016/j.juro.2014.09.103.

6.Do New Complications Develop During Puberty After Childhood Hypospadias Repair?.Snodgrass W, Bush N.The Journal of Urology. 2022;208(3):696-701. doi:10.1097/JU.0000000000002738.

AI5 (post-hoc identity: DeepSeek)

Chinese original (verbatim output):

作为小儿泌尿外科医生，当家长询问尿道下裂手术对未来生殖功能和泌尿系统功能的影响时，可以基于现有医学证据进行以下分点解释：

一、对生殖功能的影响

1. 生育能力

多数术后患儿成年后生育能力正常。研究表明：

- 轻中度尿道下裂（如阴茎头型、冠状沟型）术后生育率与普通人群无显著差异。

- 严重类型（如会阴型）可能因合并隐睾或睾丸发育不良导致生育力下降，需结合内分泌评估。

依据：欧洲小儿泌尿外科协会（ESPU）指南指出，单纯尿道下裂不直接导致不育，但合并其他生殖系统异常需额外关注（Springer et al., 2020）。

2. 勃起功能与性生活质量

- 术后阴茎弯曲矫正不良可能导致勃起疼痛或性交困难，但现代手术技术（如保留尿道板的 Snodgrass 术式）显著降低了此类风险。

- 长期随访显示，80%-90%患者成年后性功能正常（Bush et al., 2017）。

二、对泌尿系统功能的影响

1. 排尿功能

- 成功修复后，90%以上患儿可站立排尿且尿流正常。

- 并发症（如尿道狭窄、尿瘘）发生率约为 5%-15%，多数可通过二次手术矫正（Snodgrass et al., 2016）。

2. 远期泌尿健康

- 术后尿道感染风险与普通人群相近，但需注意严重病例可能合并膀胱功能异常（如神经性膀胱），需长期随访。

依据：美国泌尿外科协会（AUA）指南强调术后定期评估排尿情况（Kraft et al., 2018）。

三、心理与生活质量

- 外观与自信心：早期手术（推荐 1-2 岁）可减少心理创伤，术后阴茎外观接近正常。

- 社会适应：研究显示，及时手术干预的患儿在青春期社交焦虑水平与同龄人无差异（Weber et al., 2019）。

关键建议

1. 手术时机：推荐 1-2 岁完成修复，以减少记忆创伤并利用组织弹性。

2. 术后随访：需持续至青春期，监测尿道功能及睾丸发育。

3. 多学科支持：严重病例需联合内分泌科、心理科干预。

主要参考文献

1. Snodgrass, W., & Bush, N. (2017). *Urethral strictures after hypospadias repair: A systematic review*. Journal of Pediatric Urology.

2. Springer, A., et al. (2020). *ESPU/SPU guidelines on hypospadias: Long-term follow-up*. European Urology.

3. Kraft, K. H., et al. (2018). *AUA guideline on complications of hypospadias repair*. Journal of Urology.

4. Weber, D. M., et al. (2019). *Psychosocial outcomes in adolescents after hypospadias surgery*. Pediatrics.

通过以上结构化回答，家长可以清晰了解手术的预期效果、潜在风险及应对策略，同时权威文献引用增强了可信度。需注意根据患儿具体分型（如前型/后型）调整解释侧重点。

评价指标及定义

请您从文本质量、相关性、适用性、来源可靠性、可理解性、可操作性 6 个方面进行评价，排在越前面意味着其在各指标综合考量下分值越高。

1.质量：衡量 AI 答案对医疗疑问的解决效果，涵盖医学准确性、细节完整性以及提供医学见解的深度，判断其能否为提问者提供有价值、全面且深入的医学指导。

请将 5 个 AI 回答文本按照质量评价进行排序，例如 AI1＞AI2＞AI3＞AI4＞AI5 。

您的作答：　　＞　　＞　　＞　　＞

2.相关性：判断 AI 答案与医学问题的契合程度，包括是否紧扣主题，是否全面涉及医学问题的各个方面，以及所提供信息与医疗问题的关联紧密性。

请将 5 个 AI 回答文本按照相关性进行排序，例如 AI1＞AI2＞AI3＞AI4＞AI5 。

您的作答：　　＞　　＞　　＞　　＞

3.适用性：评估 AI 答案在询问所描述的医学背景和实际医疗场景中的可应用程度，看其是否能直接用于解决实际问题，或是需要大量调整修改。

请将 5 个 AI 回答文本按照适用性进行排序，例如 AI1＞AI2＞AI3＞AI4＞AI5 。

您的作答：　　＞　　＞　　＞　　＞

4.来源可靠性：考查 AI 答案所依据来源的可信度（是否有虚假信息或来源不详细等），包括参考文献的权威性、输出内容与文献的关联度，以及信息来源渠道是否专业、经过严格审核，以此判断答案是否准确、真实且无过时信息。

请将 5 个 AI 回答文本按照来源可靠性进行排序，例如 AI1＞AI2＞AI3＞AI4＞AI5 。

您的作答：　　＞　　＞　　＞　　＞

5.可理解性：评价 AI 答案在语言使用、内容组织以及辅助工具运用等方面，是否便于用户理解，包括语言是否通俗易懂、术语有无定义、结构是否清晰、有无视觉辅助或摘要帮助理解等。

请将 5 个 AI 回答文本按照可理解性进行排序，例如 AI1＞AI2＞AI3＞AI4＞AI5 。

您的作答：　　　＞　　　＞　　　＞　　　＞

6.可操作性：判断 AI 答案是否明确指出用户可采取的操作，是否将操作细化为清晰步骤，以及是否提供实用工具辅助用户在实际场景中采取有效行动。

请将 5 个 AI 回答文本按照可操作性进行排序，例如 AI1＞AI2＞AI3＞AI4＞AI5 。

您的作答：　　　＞　　　＞　　　＞　　　＞

根据以上内容进行综合排序：：　　　＞　　＞　　＞　　＞

English translation (machine translation; for navigation only):

As a pediatric urologist, when parents ask about the impact of hypospadias surgery on future reproductive and urinary system functions, the following points can be explained based on the existing medical evidence:

1. Impact on reproductive function

1. Fertility

Most children after surgery have normal fertility as adults. Research shows:

- The postoperative fertility rate of mild to moderate hypospadias (such as glans penis type and coronal sulcus type) is not significantly different from that of the general population.

- Severe types (such as perineal type) may lead to decreased fertility due to cryptorchidism or testicular dysplasia, and need to be combined with endocrine evaluation.

Basis: The European Society of Pediatric Urology (ESPU) guidelines state that hypospadias alone does not directly cause infertility, but combined with other reproductive system abnormalities requires additional attention (Springer et al., 2020).

2. Erectile function and sexual life quality

- Poor correction of penile curvature after surgery may lead to erection pain or dyspareunia, but modern surgical techniques (such as the urethral plate-preserving Snodgrass procedure) have significantly reduced such risks.

- Long-term follow-up shows that 80%-90% of patients have normal sexual function in adulthood (Bush et al., 2017).

2. Impact on urinary system function

1. Urination function

- After successful repair, more than 90% of children can urinate standing up and have normal urine flow.

- The incidence rate of complications (such as urethral stricture, urinary fistula) is about 5%-15%, and most of them can be corrected by secondary surgery (Snodgrass et al., 2016).

2. Long-term urinary health

- The risk of postoperative urinary tract infection is similar to that of the general population, but it should be noted that severe cases may be complicated by bladder dysfunction (such as neurogenic bladder) and require long-term follow-up.

Rationale: The American Urological Association (AUA) guidelines emphasize regular evaluation of voiding status after surgery (Kraft et al., 2018).

3. Psychology and quality of life

- Appearance and self-confidence: Early surgery (recommended 1-2 years old) can reduce psychological trauma, and the appearance of the penis after surgery is close to normal.

- Social adaptation: Research shows that children with timely surgical intervention have no difference in social anxiety levels from their peers in adolescence (Weber et al., 2019).

Key recommendations

1. Timing of surgery: It is recommended to complete the repair at 1-2 years of age to reduce memory trauma and utilize tissue elasticity.

2. Postoperative follow-up: It needs to continue until puberty to monitor urethral function and testicular development.

3. Multidisciplinary support: Severe cases require combined endocrinology and psychology intervention.

Main references

1. Snodgrass, W., & Bush, N. (2017). *Urethral strictures after hypospadias repair: A systematic review*. Journal of Pediatric Urology.

2. Springer, A., et al. (2020). *ESPU/SPU guidelines on hypospadias: Long-term follow-up*. European Urology.

3. Kraft, K. H., et al. (2018). *AUA guideline on complications of hypospadias repair*. Journal of Urology.

4. Weber, D. M., et al. (2019). *Psychosocial outcomes in adolescents after hypospadias surgery*. Pediatrics.

Through the above structured answers, parents can clearly understand the expected effects, potential risks and coping strategies of the surgery, while citing authoritative literature enhances credibility. It is necessary to pay attention to adjusting the focus of explanation according to the specific classification of the child (such as anterior type/posterior type).

Evaluation indicators and definitions

Please evaluate it from six aspects: text quality, relevance, applicability, source reliability, understandability, and operability. The higher the ranking, the higher the score based on the comprehensive consideration of each indicator.

1. Quality: Measure the effectiveness of AI answers in solving medical questions, covering medical accuracy, completeness of details and the depth of medical insights provided, to determine whether it can provide valuable, comprehensive and in-depth medical guidance to the questioner.

Please sort the 5 AI answer texts according to quality evaluation, for example, AI1>AI2>AI3>AI4>AI5.

Your answer: > > > >

2. Relevance: Determine the degree of fit between the AI answer and the medical problem, including whether it sticks to the topic, whether it comprehensively covers all aspects of the medical problem, and how closely the information provided is related to the medical problem.

Please sort the 5 AI answer texts according to their relevance, for example, AI1>AI2>AI3>AI4>AI5.

Your answer: > > > >

3. Applicability: Evaluate the applicability of the AI answers in the medical background and actual medical scenarios described in the query to see whether it can be directly used to solve practical problems, or whether it requires extensive adjustments and modifications.

Please sort the 5 AI answer texts according to their applicability, for example, AI1>AI2>AI3>AI4>AI5.

Your answer: > > > >

4. Source reliability: Examine the credibility of the sources on which the AI answers are based (whether there is false information or unclear sources, etc.), including the authority of the references, the relevance of the output content and the literature, and whether the information source channels are professional and rigorously reviewed to determine whether the answers are accurate, authentic and free of outdated information.

Please sort the 5 AI answer texts according to source reliability, for example, AI1>AI2>AI3>AI4>AI5.

Your answer: > > > >

5. Comprehensibility: Evaluate whether the AI answers are easy for users to understand in terms of language use, content organization, and use of auxiliary tools, including whether the language is easy to understand, whether terms are defined, whether the structure is clear, and whether there are visual aids or summaries to help understanding, etc.

Please sort the 5 AI answer texts according to their comprehensibility, for example, AI1>AI2>AI3>AI4>AI5.

Your answer: > > > >

6. Operability: Determine whether the AI answer clearly points out the actions that the user can take, whether the operation is refined into clear steps, and whether it provides practical tools to assist the user in taking effective actions in actual scenarios.

Please sort the 5 AI answer texts according to operability, for example, AI1>AI2>AI3>AI4>AI5.

Your answer: > > > >

Comprehensive sorting based on the above content:: > > > >

Question 8 (Medium risk; Postoperative Care)

Original prompt (Chinese):

如果你是一名小儿泌尿外科的医生，家长问到“尿道下裂患儿术后怎样判断排尿是否正常？”问题，你应该怎么回答？并给出来源依据或参考出处。

Prompt translation (English):

If you are a pediatric urologist, and a parent asks, "How can we tell whether the child's urination is normal after surgery?", how should you answer? Please cite your sources or references.

Per-question unblinding key for Q8:

Blinded label	Model identity
AI1	OpenEvidence
AI2	Zhipu Qingyan
AI3	ChatGPT-4o
AI4	DeepSeek
AI5	Gemini-2.5-Pro

AI1 (post-hoc identity: OpenEvidence)

Chinese original (verbatim output):

在儿童尿道下裂手术后，评估排尿是否正常可以通过以下几种方法：

1. 尿流动力学检查（Uroflowmetry）：这是评估尿流速率和形状的标准方法。根据 Perera 等人的研究，尿流动力学检查可以用于长期随访，评估手术后的尿道功能。研究发现，尽管尿道下裂手术后的尿流速率显著低于同龄对照组，但仍在正常范围内。[1]

2. 残余尿量测定（Post-void Residual Volume Measurement）：通过超声检查测量排尿后的残余尿量。Perera 等人的研究指出，尿流速率低与残余尿量增加显著相关。[1]

3. 症状问卷调查：评估患者的下尿路症状，如排尿后滴漏、排尿困难和尿失禁等。Moriya 等人的研究表明，尽管大多数患者在手术后有良好的尿控，但严重尿道下裂患者可能会出现终末滴漏等症状。[2]

4. 临床检查和随访：定期的临床检查和随访对于评估手术后的尿道功能和发现潜在的并发症至关重要。González 和 Ludwikowski 的系统综述建议，在无症状的儿童中，手术后应进行一次尿流检查，如果结果异常，应重复检查。[3]

综上所述，评估儿童尿道下裂手术后的正常排尿功能主要依赖于尿流动力学检查、残余尿量测定、症状问卷调查和定期临床随访。这些方法可以帮助医生全面了解患者的尿道功能，及时发现和处理潜在问题。

1.Long-Term Urethral Function Measured by Uroflowmetry After Hypospadias Surgery: Comparison With an Age Matched Control.Perera M, Jones B, O'Brien M, Hutson JM.The Journal of Urology. 2012;188(4 Suppl):1457-62. doi:10.1016/j.juro.2012.04.105.

2.Long-Term Patient Reported Outcome of Urinary Symptoms After Hypospadias Surgery: Norm Related Study in Adolescents.Moriya K, Kakizaki H, Tanaka H, et al.The Journal of Urology. 2007;178(4 Pt 2):1659-62; discussion 1662. doi:10.1016/j.juro.2007.03.176.

3.Importance of Urinary Flow Studies After Hypospadias Repair: A Systematic Review.González R, Ludwikowski BM.International Journal of Urology : Official Journal of the Japanese Urological Association. 2011;18(11):757-61. doi:10.1111/j.1442-2042.2011.02839.x.

English translation (machine translation; for navigation only):

After hypospadias surgery in children, there are several ways to assess whether urination is normal:

1. Uroflowmetry: This is a standard method to evaluate the rate and shape of urine flow. According to Perera et al., urodynamic testing can be used for long-term follow-up to evaluate urethral function after surgery. The study found that although urinary flow rate after hypospadias surgery was significantly lower than that of age-matched controls, it was still within the normal range. [1]

2. Post-void Residual Volume Measurement: Measures the residual urine volume after urination through ultrasonic examination. The study by Perera et al pointed out that low urinary flow rate was significantly associated with increased residual urine volume. [1]

3. Symptom questionnaire: Assess the patient's lower urinary tract symptoms, such as post-voiding dripping, dysuria, and urinary incontinence. The study by Moriya et al showed that although most patients have good urinary continence after surgery, patients with severe hypospadias may experience symptoms such as terminal drip. [2]

4. Clinical examination and follow-up: Regular clinical examination and follow-up are essential to evaluate urethral function after surgery and detect potential complications. The systematic review by González and Ludwikowski recommended that in asymptomatic children, a urinary flow test should be performed once after surgery and repeated if the results are abnormal. [3]

In summary, the evaluation of normal urinary function after hypospadias surgery in children mainly relies on urodynamic examination, residual urine volume measurement, symptom questionnaires and regular clinical follow-up. These methods can help doctors comprehensively understand the patient's urethral function and detect and deal with potential problems in a timely manner.

1.Long-Term Urethral Function Measured by Uroflowmetry After Hypospadias Surgery: Comparison With an Age Matched Control.Perera M, Jones B, O'Brien M, Hutson JM.The Journal of Urology. 2012;188(4 Suppl):1457-62. doi:10.1016/j.juro.2012.04.105.

2.Long-Term Patient Reported Outcome of Urinary Symptoms After Hypospadias Surgery: Norm Related Study in Adolescents.Moriya K, Kakizaki H, Tanaka H, et al.The Journal of Urology. 2007;178(4 Pt 2):1659-62; discussion 1662. doi:10.1016/j.juro.2007.03.176.

3.Importance of Urinary Flow Studies After Hypospadias Repair: A Systematic Review.González R, Ludwikowski BM.International Journal of Urology : Official Journal of the Japanese Urological Association. 2011;18(11):757-61. doi:10.1111/j.1442-2042.2011.02839.x.

AI2 (post-hoc identity: Zhipu Qingyan)

Chinese original (verbatim output):

1.观察尿流方向

正常情况：尿流应笔直向前，无分散或喷洒现象。

异常情况：尿流分散或喷洒可能表明手术修复不理想或愈合过程中存在问题。

2.注意尿流强度和稳定性

正常情况：尿流应稳定而有力，无中断或滴漏。

异常情况：尿流弱或有滴漏可能是尿道狭窄或瘘管等问题的迹象。

3.观察排尿频率和量

正常情况：排尿频率应逐渐恢复正常，尿量适中。

异常情况：排尿次数过多或过少，以及尿量异常，需引起注意。

4.注意排尿时的舒适度和其他症状

正常情况：排尿过程应无疼痛或不适。

异常情况：排尿时疼痛、血尿或发热等症状可能是感染或其他并发症的迹象。

5.记录和复查

家长应定期观察并记录患儿的排尿情况。

如发现任何异常，应及时联系医生进行复查。医生可能建议进行尿流率检查、超声波检查等进一步评估。

参考来源：

小儿泌尿外科常见疾病的诊断与治疗指南。

English translation (machine translation; for navigation only):

1. Observe the direction of urine flow

Normal situation: The urine flow should be straight forward without scattering or spraying.

Abnormalities: A scattered or spraying urine stream may indicate a suboptimal surgical repair or a problem with the healing process.

2. Pay attention to the strength and stability of urine flow

Normal conditions: The urine flow should be steady and strong, with no interruptions or dripping.

Abnormalities: A weak urine stream or dripping may be a sign of a problem such as a urethral stricture or fistula.

3. Observe the frequency and amount of urination

Normal situation: Urination frequency should gradually return to normal and urine output should be moderate.

Abnormalities: Too much or too little urination, and abnormal urine output require attention.

4. Pay attention to comfort and other symptoms while urinating

Normal conditions: Urination should occur without pain or discomfort.

Abnormalities: Symptoms such as pain when urinating, blood in the urine, or fever may be signs of infection or other complications.

5. Record and review

Parents should regularly observe and record their children's urination.

If any abnormalities are found, you should contact your doctor promptly for review. Your doctor may recommend further evaluation such as uroflowmetry or ultrasound.

Reference sources:

Guidelines for the diagnosis and treatment of common conditions in pediatric urology.

AI3 (post-hoc identity: ChatGPT-4o)

Chinese original (verbatim output):

如果我是小儿泌尿外科医生，针对家长提出的“尿道下裂患儿术后怎样判断排尿是否正常？”这个问题，我会这样回答：

临床医生的标准化回答（面向家长）：

尿道下裂手术后的排尿情况，主要从以下几个方面来观察是否正常：

1. 排尿的射流方向是否居中，或者是否明显偏斜。

2. 排尿的尿流形态是否连续、有力，而不是滴流或分叉。

3. 排尿的尿流速度是否与年龄相符，排尿时间是否延长。

4. 排尿过程中是否存在疼痛、憋尿、鼓肚子用力等异常表现。

5. 是否存在尿潴留或反复尿路感染。

如果有条件，也可以通过术后3-6个月进行客观的尿流动力学检查（如尿流率检查）来判断尿流的速度和形态是否达标。

总体而言，如果孩子术后能顺利排尿，尿流单股居中，无明显喷溅或滴流，无疼痛、憋尿现象，且没有尿路感染，一般认为排尿是正常的。但如果家长发现异常，建议及时复诊，必要时完善相关检查。

参考依据与出处：

1. Campbell-Walsh-Wein Urology 第12版（2021年）第155章：

"Postoperative evaluation of hypospadias repair includes observation of the urinary stream for straightness, forcefulness, and absence of spraying or dribbling. Voiding cystourethrography or uroflowmetry may be used if abnormalities are suspected."

2. 中国医学会泌尿外科学分会小儿泌尿外科学组专家共识（尿道下裂的诊治建议）2020版：

"术后定期随访应关注排尿功能，包括尿线方向、尿流速度、尿流连续性以及排尿不适等主观或客观指标。"

3. 欧洲泌尿外科学会（EAU）小儿泌尿外科指南（2023）：

"Assessment of voiding after hypospadias repair should include parental report, observation of urinary stream and, when indicated, uroflowmetry to detect functional obstruction or poor urinary flow."

English translation (machine translation; for navigation only):

If I were a pediatric urologist, and asked by parents, "How to determine whether urination is normal in children with hypospadias after surgery?" I would answer this way:

Clinician's standardized responses (for parents):

The urination situation after hypospadias surgery is mainly observed from the following aspects to see whether it is normal:

1. Is the direction of the urination jet centered or significantly deviated?

2. Whether the urinary flow pattern is continuous and powerful, rather than dripping or bifurcated.

3. Whether the urinary flow speed is consistent with the age and whether the urination time is prolonged.

4. Are there any abnormal symptoms during urination, such as pain, holding back urine, straining to bulge the abdomen, etc.

5. Whether there is urinary retention or recurrent urinary tract infections.

If possible, objective urodynamic tests (such as uroflowmetry) can be performed 3-6 months after surgery to determine whether the speed and shape of the urine flow are up to standard.

Generally speaking, if the child can urinate smoothly after surgery, with a single stream of urine in the center, no obvious splashing or dripping, no pain, no urinary retention, and no urinary tract infection, urination is generally considered normal. However, if parents find abnormalities, it is recommended that they be followed up in time and complete relevant examinations if necessary.

Reference basis and source:

1. Campbell-Walsh-Wein Urology 12th Edition (2021) Chapter 155:

“Postoperative evaluation of hypospadias repair includes observation of the urinary stream for straightness, forcefulness, and absence of spraying or dribbling. Voiding cystourethrography or uroflowmetry may be used if abnormalities are suspected.”

2. Expert consensus of the Pediatric Urology Group of the Urology Branch of the Chinese Medical Association (Recommendations for the Diagnosis and Treatment of Hypospadias) 2020 Edition:

"Regular follow-up after surgery should focus on urinary function, including subjective or objective indicators such as urine line direction, urine flow speed, urine flow continuity, and urinary discomfort."

3. European Association of Urology (EAU) Pediatric Urology Guidelines (2023):

“Assessment of voiding after hypospadias repair should include parental report, observation of urinary stream and, when indicated, uroflowmetry to detect functional obstruction or poor urinary flow.”

AI4 (post-hoc identity: DeepSeek)

Chinese original (verbatim output):

作为小儿泌尿外科医生，您可以这样回答家长的问题，并附上专业依据：

尿道下裂术后排尿正常的判断标准

1. 尿线粗细与方向

- 正常表现：尿流呈连续直线，无明显分叉或散射，方向与尿道开口位置一致（如正位尿道口则向前喷射）。

- 异常信号：尿线细弱、滴沥状、分叉明显或向后（如阴囊方向）排尿，可能提示尿道狭窄、瘘管或残余弯曲。

2. 排尿顺畅度

- 正常表现：患儿可自主控制排尿，无费力、哭闹或中断。

- 异常信号：排尿时需用力、哭闹或尿流中断，需警惕尿道狭窄或膀胱出口梗阻。

3. 尿量及频率

- 正常表现：尿量与术前一致，无尿频、尿急或夜尿增多。

- 异常信号：尿量减少、频繁排尿或尿不尽感，可能与感染或膀胱功能异常相关。

4. 局部症状

- 正常表现：阴茎外观对称，尿道口无红肿、渗液，排尿后无尿液从异常部位漏出（如尿道瘘）。

- 异常信号：尿道口红肿、渗液，或排尿后阴囊/阴茎根部湿润（提示尿瘘），需及时复查。

5. 实验室及影像学评估

- 术后随访：医生可能通过尿流率测定（评估尿流速度）、尿道造影或超声检查确认尿道通畅性。

家长需注意的观察要点

- 短期（术后 1-2 周）：因局部水肿可能导致暂时性排尿困难，需遵医嘱使用支架管或压迫包扎。

- 长期（术后 3-6 个月）：定期复查尿道口径及功能，避免瘢痕挛缩导致的远期狭窄。

专业依据与参考文献

1. 欧洲泌尿外科学会（EAU）指南

- 指出尿道下裂术后需评估尿道口径、尿流动力学及外观，尿瘘和狭窄是最常见并发症（EAU Paediatric Urology Guidelines, 2023）。

2. 美国泌尿外科学会（AUA）共识

- 强调术后需通过尿流率测定和临床检查判断排尿功能，建议术后 1、3、6 个月随访（AUA Hypospadias Guidelines, 2014）。

3. 《Campbell-Walsh 泌尿外科学》

- 提出术后排尿异常的评估应包括尿流形态、尿道造影及排除膀胱功能障碍（第 12 版，第 146 章）。

家长沟通建议

用通俗语言解释："术后孩子排尿应该是一条连续的水柱，方向朝前，没有滴漏或疼痛。如果发现尿流变细、分叉，或者尿液从其他位置漏出，请及时联系我们复查。"

通过以上标准，家长可初步观察孩子的排尿情况，但需结合医生的专业评估以确保远期效果。

English translation (machine translation; for navigation only):

As a pediatric urologist, you can answer parents' questions with professional support:

Criteria for judging normal urination after hypospadias surgery

1. Urine line thickness and direction

- Normal performance: The urine flow is a continuous straight line, without obvious bifurcation or scattering, and the direction is consistent with the position of the urethral opening (if the urethral opening is in the upright position, it will spray forward).

- Abnormal signals: The urine line is thin, dripping, obviously bifurcated, or urinating backward (such as in the direction of the scrotum), which may indicate urethral stricture, fistula, or residual curvature.

2. Smoothness of urination

- Normal performance: The child can control urination independently without effort, crying or interruption.

- Abnormal signs: Struggling to urinate, crying, or interruption of urine flow. Be wary of urethral stricture or bladder outlet obstruction.

3. Urine volume and frequency

- Normal performance: The urine output is consistent with that before surgery, and there is no urinary frequency, urgency or nocturia.

- Abnormal signs: decreased urine output, frequent urination or a feeling of incomplete urination, which may be related to infection or abnormal bladder function.

4. Local symptoms

- Normal manifestations: The appearance of the penis is symmetrical, there is no redness, swelling or leakage at the urethral opening, and there is no leakage of urine from abnormal parts after urination (such as urethral fistula).

- Abnormal signals: redness and swelling of the urethral orifice, exudation, or moisture at the base of the scrotum/penis after urination (suggesting urinary fistula), which requires timely review.

5. Laboratory and imaging evaluation

- Postoperative follow-up: Your doctor may confirm urethral patency with uroflowmetry (assessing the speed of urine flow), urethrogram, or ultrasound.

Observation points for parents to pay attention to

- Short-term (1-2 weeks after surgery): Local edema may cause temporary difficulty in urinating, and you need to use a stent or compression bandage as directed by your doctor.

- Long-term (3-6 months after surgery): Regularly review the caliber and function of the urethra to avoid long-term stenosis caused by scar contracture.

Professional basis and references

1. European Association of Urology (EAU) Guidelines

- It is pointed out that urethral caliber, urodynamics and appearance need to be evaluated after hypospadias surgery, and urinary fistula and stricture are the most common complications (EAU Paediatric Urology Guidelines, 2023).

2. American Urological Association (AUA) consensus

- It is emphasized that urinary function needs to be judged through uroflowmetry and clinical examination after surgery, and follow-up is recommended for 1, 3, and 6 months after surgery (AUA Hypospadias Guidelines, 2014).

3. "Campbell-Walsh Urology"

- It is proposed that the evaluation of postoperative voiding abnormalities should include urinary flow pattern, urethrography, and exclusion of bladder dysfunction (12th edition, Chapter 146).

Parent communication suggestions

Explained in plain language: "After surgery, your child should urinate in a continuous column of water, facing forward, without dripping or pain. If you find that the urine stream becomes thinner, bifurcated, or urine leaks from other locations, please contact us in time for review."

Through the above standards, parents can initially observe their children's urination, but it needs to be combined with a doctor's professional evaluation to ensure long-term results.

AI5 (post-hoc identity: Gemini-2.5-Pro)

Chinese original (verbatim output):

好的，家长您好。很高兴能解答您的疑问。孩子做完尿道下裂手术后，家长确实会非常关心排尿的情况，这是完全可以理解的，也是术后观察恢复情况的重要指标。判断排尿是否“正常”需要一个过程，并且要结合孩子术后的不同阶段来看。

以下是一些关键的观察点，帮助您判断孩子的排尿情况：

1. 尿线的位置和方向：

* 核心目标： 手术的主要目的之一就是将尿道开口重建在阴茎头的顶端。因此，最理想的情况是，孩子排尿时，尿液是从阴茎头最前端的新尿道口排出。

* 观察重点：注意观察尿液是否准确地从龟头顶端的新开口流出。

* 是否正常： 如果尿液从此位置排出，通常表明手术在重建尿道开口位置方面是成功的。

2. 尿线的形态和力量：

* 核心目标： 重建的尿道应该通畅，能够形成一股相对集中的尿线，而不是呈喷洒状或非常细弱。

* 观察重点： 观察孩子排尿时尿线的粗细、是否有力、是否呈直线（允许有轻微的自然弧度）。刚拔除尿管后，尿线可能会有些分叉或不够完美，这需要一定的恢复时间。但如果长时间尿线非常细、排尿费力、或者呈喷洒状（像花洒一样），需要警惕。

* 是否正常： 一条比较直、有一定力量、不费力的尿线通常是正常的。需要注意，尿线的完美程度可能需要几个月甚至更长时间来稳定。

排尿过程是否顺畅，有无疼痛或费力：

* 核心目标： 排尿应该是自然、不费力的过程。

* 观察重点： 观察孩子排尿时是否显得很用力、哭闹、面色涨红，或者表示疼痛。术后早期可能因伤口有些不适，但持续的、明显的疼痛或排尿困难则不正常。

* 是否正常： 孩子能够比较轻松、自然地完成排尿过程是正常的。

4. 有无异常漏尿：

* 核心目标： 尿液应该只从新建的尿道口排出。

* 观察重点： 仔细检查除了龟头顶端的新尿道口外，阴茎体、根部或阴囊皮肤上是否有其他地方在排尿时漏尿。这可能是出现了“尿道皮肤瘘”。

* 是否正常： 只有单一尿道口排尿是正常的。任何其他部位的漏尿都需要及时告知医生。

排尿时有无局部皮肤鼓包：

* 核心目标： 尿道应该均匀，没有狭窄或憩室。

* 观察重点： 观察排尿时尿道附近的皮肤（特别是阴茎腹侧）是否有像气球一样鼓起来的情况。这可能提示尿道存在狭窄或憩室。

* 是否正常： 排尿时局部皮肤不应有异常鼓包。

6. 伤口愈合情况及有无感染迹象：

* 核心目标： 伤口良好愈合，没有感染。

* 观察重点： 伤口周围是否红肿、发热、有脓性分泌物？孩子是否有发烧？尿液是否浑浊、有异味？这些都可能是感染的迹象，感染会影响排尿和愈合。

* 是否正常： 伤口清洁、干燥、无红肿、无脓液，孩子体温正常，尿液清亮，则愈合情况较好。

总结和建议：

* 初期观察（刚拔管后）： 可能会有些肿胀、尿线分叉、轻微不适，甚至少量血丝，这在允许范围内。关键是尿液能从新尿道口排出，并且没有持续的排尿困难。

* 恢复期观察（术后几周至几个月）： 重点关注尿线是否逐渐变直、有力，有无漏尿，排尿是否费力。

* 定期复查至关重要： 即使您觉得孩子排尿看起来正常，也一定要按照医嘱按时复查。医生会通过专业检查（有时包括尿流率测定、B 超等）来更准确地评估恢复情况，及时发现潜在问题，如远期的尿道狭窄或瘘。

* 及时沟通： 如果您观察到任何上述提到的“不正常”情况，或者您有任何疑虑，请不要犹豫，务必及时联系您的主治医生或手术团队进行咨询。

来源依据与参考出处：

判断尿道下裂术后排尿情况的标准和观察要点是小儿泌尿外科领域公认的临床实践原则。这些信息通常基于：

1. 权威医学教科书：

* 《坎贝尔-沃尔什泌尿外科学》（Campbell-Walsh Urology）：这是国际泌尿外科领域的权威参考书，其中有章节详细讨论尿道下裂的手术技术、术后管理和并发症处理。

* 《实用小儿泌尿外科学》或国内类似的小儿外科/泌尿外科教材：通常会包含尿道下裂术后护理和评估的内容。

2. 临床指南和专家共识：

* 例如，美国泌尿外科学会（AUA）或欧洲泌尿外科学会（EAU）可能会发布相关指南或最佳实践建议，虽然可能更侧重于手术技术，但术后评估是其中重要环节。

3. 医学研究论文和文献：

* 大量发表在专业期刊（如 *Journal of Urology*, *Journal of Pediatric Urology*, *Urology* 等）上的研究论文会探讨尿道下裂术后效果的评估方法、并发症的识别和处理。例如，搜索 "hypospadias post-operative assessment", "uroflowmetry after hypospadias repair", "hypospadias complications fistula stenosis" 等关键词可以找到相关研究。

4. 大型儿童医院或医学中心的患者教育材料：

* 许多知名儿童医院（如波士顿儿童医院、费城儿童医院、国内的复旦大学附属儿科医院、北京儿童医院等）的官方网站或宣传册，会为家长提供关于尿道下裂术后护理的指导信息，其中就包括如何观察排尿。这些材料通常由专业医生编写或审核。

* *示例（非直接引用，仅说明来源类型）*：波士顿儿童医院关于尿道下裂的页面可能会有 "After Surgery Care" 的部分，提到观察尿流、伤口等。 (您可以通过搜索 "Boston Children's Hospital hypospadias patient information" 等找到类似资源)。

请记住，每个孩子的具体情况和恢复过程可能略有不同，最了解您孩子情况的是您的主治医生。保持沟通，按时复查是确保孩子顺利康复的关键。希望这些信息对您有帮助！

评价指标及定义

请您从文本质量、相关性、适用性、来源可靠性、可理解性、可操作性 6 个方面进行评价，排在越前面意味着其在各指标综合考量下分值越高。

1.质量：衡量 AI 答案对医疗疑问的解决效果，涵盖医学准确性、细节完整性以及提供医学见解的深度，判断其能否为提问者提供有价值、全面且深入的医学指导。

请将 5 个 AI 回答文本按照质量评价进行排序，例如 AI1＞AI2＞AI3＞AI4＞AI5 。

您的作答：　　＞　　＞　　＞　　＞

2.相关性：判断 AI 答案与医学问题的契合程度，包括是否紧扣主题，是否全面涉及医学问题的各个方面，以及所提供信息与医疗问题的关联紧密性。

请将 5 个 AI 回答文本按照相关性进行排序，例如 AI1＞AI2＞AI3＞AI4＞AI5 。

您的作答：　　＞　　＞　　＞　　＞

3.适用性：评估 AI 答案在询问所描述的医学背景和实际医疗场景中的可应用程度，看其是否能直接用于解决实际问题，或是需要大量调整修改。

请将 5 个 AI 回答文本按照适用性进行排序，例如 AI1＞AI2＞AI3＞AI4＞AI5 。

您的作答：　　＞　　＞　　＞　　＞

4.来源可靠性：考查 AI 答案所依据来源的可信度（是否有虚假信息或来源不详细等），包括参考文献的权威性、输出内容与文献的关联度，以及信息来源渠道是否专业、经过严格审核，以此判断答案是否准确、真实且无过时信息。

请将 5 个 AI 回答文本按照来源可靠性进行排序，例如 AI1＞AI2＞AI3＞AI4＞AI5 。

您的作答：　　＞　　＞　　＞　　＞

5.可理解性：评价 AI 答案在语言使用、内容组织以及辅助工具运用等方面，是否便于用户理解，包括语言是否通俗易懂、术语有无定义、结构是否清晰、有无视觉辅助或摘要帮助理解等。

请将 5 个 AI 回答文本按照可理解性进行排序，例如 AI1＞AI2＞AI3＞AI4＞AI5 。

您的作答：　　＞　　＞　　＞　　＞

6.可操作性：判断 AI 答案是否明确指出用户可采取的操作，是否将操作细化为清晰步骤，以及是否提供实用工具辅助用户在实际场景中采取有效行动。

请将 5 个 AI 回答文本按照可操作性进行排序，例如 AI1＞AI2＞AI3＞AI4＞AI5 。

您的作答：　　＞　　＞　　＞　　＞

根据以上内容进行综合排序：：　　＞　　＞　　＞　　＞

English translation (machine translation; for navigation only):

Okay, hello parents. I'm happy to answer your questions. After a child undergoes hypospadias surgery, parents will indeed be very concerned about the urination situation. This is completely understandable and is also an important indicator of postoperative recovery. Determining whether urination is "normal" requires a process, and must be considered in conjunction with the child's different stages after surgery.

Here are some key observations to help you determine how your child is urinating:

1. Location and direction of urine line:

* Core Goal: One of the main goals of surgery is to reconstruct the urethral opening at the top of the glans penis. Therefore, the most ideal situation is that when the child urinates, the urine is discharged from the new urethral opening at the front of the penis head.

* Observation focus: Pay attention to whether urine flows out accurately from the new opening on the top of the glans.

* Is it normal: If urine is discharged from this location, it usually indicates that the surgery was successful in reestablishing the location of the urethral opening.

2. The shape and strength of the urine line:

* Core goal: The reconstructed urethra should be unobstructed and should be able to form a relatively concentrated urine line, rather than being spray-like or very thin.

* Observation focus: Observe the thickness of the urine line when the child urinates, whether it is strong, and whether it is straight (a slight natural curvature is allowed). Immediately after the urinary catheter is removed, the urinary line may be somewhat bifurcated or imperfect, which requires some recovery time. But if the urine line is very thin for a long time, it is difficult to urinate, or it sprays like a shower head, you need to be vigilant.

* Is it normal: A relatively straight, strong, and effortless urine line is usually normal. It is important to note that it may take several months or even longer for a perfect urine line to stabilize.

Is the urination process smooth, painful or laborious:

* Core Goal: Urination should be a natural, effortless process.

* Observation points: Observe whether the child exerts force when urinating, cries, turns red, or expresses pain. There may be some discomfort from the wound in the early postoperative period, but persistent, significant pain or difficulty urinating is not normal.

* Is it normal: It is normal for children to be able to complete the urination process relatively easily and naturally.

4. Is there any abnormal urine leakage:

* Core Goal: Urine should exit only through the newly created urethral opening.

* Key points to observe: In addition to the new urethral opening at the top of the glans penis, carefully check whether there are any other places on the penis body, root or scrotal skin that leak urine during urination. This may be a "urethrocutaneous fistula".

* Is it normal: It is normal to urinate from only one urethral opening. Any leakage of urine from other areas requires prompt notification to your doctor.

Is there any local skin bulging when urinating:

* Core Goal: The urethra should be even and free of strictures or diverticula.

* Observation focus: Observe whether the skin near the urethra (especially the ventral side of the penis) bulges like a balloon during urination. This may indicate a stricture or diverticulum in the urethra.

* Is it normal: There should be no abnormal bulging of the local skin during urination.

6. Wound healing status and signs of infection:

* Core Goal: Good wound healing without infection.

* Key points to observe: Is there redness, swelling, heat, or purulent secretions around the wound? Does your child have a fever? Is the urine cloudy and smelly? These may be signs of infection, which can affect urination and healing.

* Is it normal: If the wound is clean, dry, no redness, swelling, or pus, the child's body temperature is normal, and the urine is clear, the healing is good.

Summary and recommendations:

*Initial observation (just after extubation): There may be some swelling, bifurcated urine lines, slight discomfort, and even a small amount of bloodshot, which is within the allowable range. The key is that urine can pass through the new urethral opening without ongoing difficulty urinating.

* Observation during the recovery period (weeks to months after surgery): Focus on whether the urine line gradually becomes straighter and stronger, whether there is urine leakage, and whether it is difficult to urinate.

* Regular check-ups are crucial: Even if you think your child's urination seems normal, be sure to follow your doctor's instructions for follow-up check-ups. Doctors will conduct professional examinations (sometimes including uroflowmetry, B-ultrasound, etc.) to more accurately assess recovery and promptly detect potential problems, such as long-term urethral stricture or fistula.

* Timely communication: If you observe any of the above-mentioned "abnormal" conditions, or you have any concerns, please do not hesitate to contact your attending doctor or surgical team for consultation.

Source basis and reference:

The criteria and observation points for judging urination after hypospadias surgery are recognized clinical practice principles in the field of pediatric urology. This information is usually based on:

1. Authoritative medical textbook:

* "Campbell-Walsh Urology": This is the authoritative reference book in the field of international urology. It has chapters that discuss the surgical technique, postoperative management and complications of hypospadias in detail.

* "Practical Pediatric Urology" or similar domestic pediatric surgery/urology textbooks: usually include content on postoperative care and evaluation of hypospadias.

2. Clinical guidelines and expert consensus:

* For example, the American Urological Association (AUA) or the European Association of Urology (EAU) may issue relevant guidelines or best practice recommendations, which may focus more on surgical technique, but postoperative evaluation is an important part of it.

3. Medical research papers and documents:

* A large number of research papers published in professional journals (such as *Journal of Urology*, *Journal of Pediatric Urology*, *Urology*, etc.) will discuss the evaluation methods of postoperative effects of hypospadias, and the identification and management of complications. For example, searching for keywords such as "hypospadias post-operative assessment", "uroflowmetry after hypospadias repair", "hypospadias complications fistula stenosis" and other keywords can find relevant studies.

4. Patient education materials from a large children's hospital or medical center:

* The official websites or brochures of many well-known children's hospitals (such as Boston Children's Hospital, Children's Hospital of Philadelphia, the Children's Hospital of Fudan University in China, Beijing Children's Hospital, etc.) will provide parents with guidance information on post-operative care for hypospadias, including how to observe urination. These materials are usually written or reviewed by professional doctors.

* *Example (not a direct quote, just stating the type of source)*: Boston Children's Hospital's page about hypospadias may have an "After Surgery Care" section that mentions observing urine flow, wounds, etc. (You can find similar resources by searching for "Boston Children's Hospital hypospadias patient information" and more).

Keep in mind that each child's specific situation and recovery process may be slightly different, and the person who knows your child's situation best is your primary care physician. Maintaining communication and regular check-ups are key to ensuring a smooth recovery for your child. Hope this information is helpful!

Evaluation indicators and definitions

Please evaluate it from six aspects: text quality, relevance, applicability, source reliability, understandability, and operability. The higher the ranking, the higher the score based on the comprehensive consideration of each indicator.

1. Quality: Measure the effectiveness of AI answers in solving medical questions, covering medical accuracy, completeness of details and the depth of medical insights provided, to determine whether it can provide valuable, comprehensive and in-depth medical guidance to the questioner.

Please sort the 5 AI answer texts according to quality evaluation, for example, AI1>AI2>AI3>AI4>AI5.

Your answer: ＞ ＞ ＞ ＞

2. Relevance: Determine the degree of fit between the AI answer and the medical problem, including whether it sticks to the topic, whether it comprehensively covers all aspects of the medical problem, and how closely the information provided is related to the medical problem.

Please sort the 5 AI answer texts according to their relevance, for example, AI1>AI2>AI3>AI4>AI5.

Your answer: ＞ ＞ ＞ ＞

3. Applicability: Evaluate the applicability of the AI answers in the medical background and actual medical scenarios described in the query to see whether it can be directly used to solve practical problems, or whether it requires extensive adjustments and modifications.

Please sort the 5 AI answer texts according to their applicability, for example, AI1>AI2>AI3>AI4>AI5.

Your answer: ＞ ＞ ＞ ＞

4. Source reliability: Examine the credibility of the sources on which the AI answers are based (whether there is false information or unclear sources, etc.), including the authority of the references, the relevance of the output content and the literature, and whether the information source channels are professional and rigorously reviewed to determine whether the answers are accurate, authentic and free of outdated information.

Please sort the 5 AI answer texts according to source reliability, for example, AI1>AI2>AI3>AI4>AI5.

Your answer: ＞ ＞ ＞ ＞

5. Comprehensibility: Evaluate whether the AI answers are easy for users to understand in terms of language use, content organization, and use of auxiliary tools, including whether the language is easy to understand, whether terms are defined, whether the structure is clear, and whether there are visual aids or summaries to help understanding, etc.

Please sort the 5 AI answer texts according to their comprehensibility, for example, AI1>AI2>AI3>AI4>AI5.

Your answer: ＞ ＞ ＞ ＞

6. Operability: Determine whether the AI answer clearly points out the actions that the user can take, whether the operation is refined into clear steps, and whether it provides practical tools to assist the user in taking effective actions in actual scenarios.

Please sort the 5 AI answer texts according to operability, for example, AI1>AI2>AI3>AI4>AI5.

Your answer: > > > >

Comprehensive sorting based on the above content:: > > > >

Question 9 (High risk; Postoperative Care)

Original prompt (Chinese):

如果你是一名小儿泌尿外科的医生，家长问到“尿道下裂患儿术后若出现排尿困难或尿痛等情况，应该怎么办？”问题，你应该怎么回答？并给出来源依据或参考出处。

Prompt translation (English):

If you are a pediatric urologist, and a parent asks, "What should we do if the child experiences urinary difficulty or dysuria after surgery?", how should you answer? Please cite your sources or references.

Per-question unblinding key for Q9:

Blinded label	Model identity
AI1	OpenEvidence
AI2	Gemini-2.5-Pro
AI3	Zhipu Qingyan
AI4	DeepSeek
AI5	ChatGPT-4o

AI1 (post-hoc identity: OpenEvidence)

Chinese original (verbatim output):

如果一名家长询问关于孩子在尿道下裂手术后出现排尿困难或排尿时疼痛的处理方法，作为儿科泌尿科医生，您可以这样回答：

首先，排尿困难或疼痛可能是术后常见的并发症之一。根据《Pediatric Surgery International》的一项研究，年龄较大的儿童在尿道下裂手术后更容易出现早期并发症，包括尿潴留和显著的痉挛性疼痛。因此，术后监测和及时处理这些症状非常重要。[1]

如果孩子出现排尿困难或疼痛，建议首先进行详细的临床评估，以确定是否存在尿道狭窄、尿道瘘或其他术后并发症。根据《The Journal of Urology》的一项研究，术后尿流量可能会随着时间的推移而改善，但在某些情况下可能需要进行尿道扩张或肉膜切开术。[2]

此外，术后护理和家长教育也至关重要。根据《Pediatric Surgery International》的建议，家长应严格遵守术后护理指导，包括正确处理敷料和导管，并确保药物的按时服用。[1]

如果症状持续或加重，可能需要进一步的影像学检查，如超声检查或尿道膀胱造影，以评估是否存在结构性异常。在某些情况下，可能需要进行紧急处理，如插入耻骨上导尿管以缓解尿潴留。[3]

综上所述，术后出现排尿困难或疼痛时，应进行详细的临床评估和适当的影像学检查，并根据具体情况采取相应的治疗措施。家长教育和术后护理也非常重要，以减少并发症的发生。

1.Age-Dependent Early Complications of Hypospadias Repair: A Single Institutional Experience.

Dale J, Woodward B, Elagami H.Pediatric Surgery International. 2023;39(1):115. doi:10.1007/s00383-023-05388-z.

2.Normalized Urinary Flow at Puberty After Tubularized Incised Plate Urethroplasty for Hypospadias in Childhood.Andersson M, Doroszkiewicz M, Arfwidsson C, et al.The Journal of Urology. 2015;194(5):1407-13. doi:10.1016/j.juro.2015.06.072.

3.Urinary Tract Abnormalities in Boys With Recurrent Urinary Tract Infections After Hypospadias Repair.Wehbi E, Patel P, Kanaroglou N, et al.BJU International. 2014;113(2):304-8. doi:10.1111/bju.12387.

English translation (machine translation; for navigation only):

If a parent asks about managing their child's difficulty urinating or pain while urinating after hypospadias surgery, as a pediatric urologist, you might respond by:

First, difficulty or pain during urination may be one of the common complications after surgery. Older children are more likely to experience early complications after hypospadias surgery, including urinary retention and significant cramping pain, according to a study in Pediatric Surgery International. Therefore, it is important to monitor and promptly manage these symptoms after surgery. [1]

If a child presents with difficulty or pain during urination, a detailed clinical evaluation is recommended first to determine whether urethral stricture, urethral fistula, or other postoperative complications are present. According to a study in The Journal of Urology, postoperative urinary flow may improve over time, but in some cases a urethral dilation or carnosiotomy may be required. [2]

Additionally, post-operative care and parent education are critical. According to recommendations from Pediatric Surgery International, parents should strictly follow post-operative care instructions, including proper handling of dressings and catheters, and ensuring medications are taken on time. [1]

If symptoms persist or worsen, further imaging, such as ultrasonography or urethrocystography, may be needed to evaluate for structural abnormalities. In some cases, emergency treatment may be needed, such as inserting a suprapubic catheter to relieve urinary retention. [3]

In summary, when dysuria or pain occurs after surgery, detailed clinical evaluation and appropriate imaging examinations should be performed, and corresponding treatment measures should be taken according to the specific situation. Parental education and postoperative care are also very important to reduce the occurrence of complications.

1.Age-Dependent Early Complications of Hypospadias Repair: A Single Institutional Experience.

Dale J, Woodward B, Elagami H.Pediatric Surgery International. 2023;39(1):115. doi:10.1007/s00383-023-05388-z.

2.Normalized Urinary Flow at Puberty After Tubularized Incised Plate Urethroplasty for Hypospadias in Childhood.Andersson M, Doroszkiewicz M, Arfwidsson C, et al.The Journal of Urology. 2015;194(5):1407-13. doi:10.1016/j.juro.2015.06.072.

3.Urinary Tract Abnormalities in Boys With Recurrent Urinary Tract Infections After Hypospadias Repair.Wehbi E, Patel P, Kanaroglou N, et al.BJU International. 2014;113(2):304-8. doi:10.1111/bju.12387.

AI2 (post-hoc identity: Gemini-2.5-Pro)

Chinese original (verbatim output):

您好，非常理解您作为家长的担心。孩子在尿道下裂手术后出现排尿困难或尿痛的情况，确实需要我们密切关注和及时处理。请不要慌张，按照以下步骤来做：

1. 立即联系您的主刀医生或手术团队： 这是最重要的一步。请尽快通过您有的联系方式（电话、微信等）联系负责孩子手术的医生或医院的相关科室。

  * 详细说明情况： 告诉医生孩子具体是什么时候开始出现排尿困难或尿痛的，排尿时是什么表现（比如：尿线很细、断断续续、滴尿、需要很用力才能尿出来、排尿时哭闹严重等）。

  * 提供其他信息： 同时告知医生孩子是否有发烧、尿液是否浑浊或有异味、伤口敷料是否有异常渗出、精神状态如何等。

2. 可能的原因及医生的评估方向： 医生会根据您描述的情况，初步判断可能的原因。术后早期出现这些症状，常见的原因可能包括：

  * 术后正常水肿： 手术区域会有一定程度的肿胀，这可能暂时性地影响排尿的顺畅度。

  * 尿道痉挛： 手术刺激或疼痛可能引起尿道或膀胱肌肉的痉挛，导致排尿费力或疼痛。

  * 尿管问题（如果还留置尿管）： 尿管可能发生堵塞、打折或轻微移位。

  * 伤口敷料影响： 包扎过紧或敷料位置不当有时也会影响排尿。

  * 暂时性堵塞： 小血块或分泌物可能暂时堵塞新形成的尿道。

  * 尿路感染： 虽然相对少见，但也是可能的原因之一，通常会伴随发烧、尿液异常等。

3. 遵循医生的具体指导：

  * 医生可能会先通过电话指导您一些观察或处理方法（比如调整敷料、确认尿管是否通畅等）。

  * 很多情况下，医生会要求您尽快带孩子回医院进行评估。医生需要亲自检查孩子的伤口愈合情况、评估尿道是否通畅，可能需要进行尿常规检查排除感染，或者做 B 超检查了解膀胱和尿道的情况。

  * 切勿自行处理： 请不要自行拔除尿管（如果还在留置）、挑开敷料或尝试用任何工具插入尿道，这可能会导致更严重的问题或感染。

4. 在家等待就医期间可以做的：

  * 安抚孩子： 尽量让孩子保持放松，情绪紧张可能会加重排尿困难。

  * 遵医嘱用药： 如果医生之前有开具止痛药或解痉药物（如缓解膀胱痉挛的药物），请按时按量给孩子服用。

  * 观察体温： 定时测量孩子的体温。

* 鼓励饮水（如医生允许）： 保持充足的水分有助于稀释尿液，减轻刺激，并可能促进排尿，但请先确认医生是否允许。

总结来说，遇到这种情况，最关键的是及时与您的手术医生或团队取得联系，并遵循他们的专业指导。大多数术后早期的排尿问题，通过及时的评估和处理都能够得到解决。请对医疗团队保持信任，密切配合。

来源依据与参考：

虽然无法直接引用特定的某一篇文献作为唯一“答案”，但以下类型的资源和公认的临床实践支持上述回答：

1. 权威医学教科书： 如《坎贝尔-沃尔什泌尿外科学》（Campbell-Walsh Urology）等权威小儿泌尿外科教科书中关于尿道下裂术后并发症处理的章节，会详细描述术后可能出现的排尿困难、尿瘘、尿道狭窄等并发症及其处理原则，强调早期识别和及时就医的重要性。

2. 临床诊疗指南： 各国小儿泌尿外科学会或协会发布的关于尿道下裂诊疗的指南或专家共识，通常会包含术后管理和并发症处理的部分。这些指南强调了术后密切随访和出现问题时需联系手术医生的原则。

3. 大型医疗中心/儿童医院网站： 许多知名的儿童医院（如波士顿儿童医院、费城儿童医院、国内各大儿童医学中心）的官方网站上，会有关于尿道下裂手术的介绍，其中通常包含术后护理和注意事项，会提及排尿异常是需要联系医生的指征。 *(例如，搜索 "hypospadias surgery post-op care urination problems" 或中文 "尿道下裂术后护理 排尿困难" 可以找到类似信息)*

4. 医学期刊文献： 相关医学期刊（如 Journal of Pediatric Urology, Urology 等）发表的研究论文和病例报告，会讨论尿道下裂术后并发症的发生率、原因分析和处理经验，这些文献共同构成了临床实践的基础。

核心原则：上述回答的核心原则——“术后出现异常症状（如排尿困难/疼痛）需立即联系手术医生进行评估和处理”——是小儿外科围手术期管理的基本准则，适用于几乎所有类型的手术，在尿道下裂这种精细重建手术后尤为重要。

English translation (machine translation; for navigation only):

Hello, I understand your concerns as a parent. Children who have difficulty urinating or painful urination after hypospadias surgery do need our close attention and prompt treatment. Please don’t panic and follow these steps:

1. Contact your surgeon or surgical team immediately: This is the most important step. Please contact the doctor responsible for your child's surgery or the relevant department of the hospital through the contact information you have (telephone, WeChat, etc.) as soon as possible.

* Describe the situation in detail: Tell the doctor exactly when the child began to have difficulty urinating or painful urination, and what the symptoms were when urinating (for example: very thin urine lines, intermittent urination, dripping urine, requiring a lot of force to urinate, severe crying during urination, etc.).

* Provide other information: Also inform the doctor whether the child has a fever, whether the urine is turbid or has a peculiar smell, whether there is abnormal leakage from the wound dressing, and what the child's mental state is.

2. Possible causes and the doctor’s assessment direction: The doctor will make a preliminary judgment on the possible causes based on the situation you describe. Common causes of these symptoms early after surgery may include:

* Normal postoperative edema: There will be a certain degree of swelling in the surgical area, which may temporarily affect the smoothness of urination.

* Urethral spasm: Surgical irritation or pain may cause spasms in the urethra or bladder muscles, making urination difficult or painful.

* Catheter problems (if you still have a urinary catheter): The urinary catheter may be blocked, kinked, or slightly displaced.

* Impact of wound dressings: Over-tight dressing or improper placement of dressings can sometimes affect urination.

* Temporary blockage: A small blood clot or secretion may temporarily block the newly formed urethra.

* Urinary tract infection: Although relatively rare, it is also a possible cause and is usually accompanied by fever, abnormal urine, etc.

3. Follow your doctor’s specific instructions:

* The doctor may first guide you over the phone with some observation or treatment methods (such as adjusting the dressing, confirming whether the urinary catheter is unobstructed, etc.).

* In many cases, the doctor will ask you to take your child back to the hospital for evaluation as soon as possible. The doctor needs to personally check the child's wound healing and assess whether the urethra is unobstructed. He may need to conduct a routine urine test to rule out infection, or do an ultrasound to understand the condition of the bladder and urethra.

* Never do it yourself: Please do not remove the urinary catheter (if it is still in place), pick at the dressing, or try to insert any tool into the urethra as this may cause more serious problems or infection.

4. What you can do while waiting for medical treatment at home:

* Soothe your child: Try to keep your child as relaxed as possible. Emotional stress may worsen urinary difficulties.

* Take medications as directed by your doctor: If the doctor has previously prescribed painkillers or antispasmodics (such as drugs to relieve bladder spasms), please give them to your child on time and in the right amount.

* Observe body temperature: Measure your child's temperature regularly.

* Encourage drinking water (if your doctor allows it): Staying adequately hydrated can help dilute urine, reduce irritation, and may promote urination, but check with your doctor first.

In summary, when encountering this situation, the most critical thing is to contact your surgeon or team promptly and follow their professional guidance. Most early postoperative voiding problems can be resolved with prompt evaluation and management. Please maintain trust in the medical team and cooperate closely.

Source basis and reference:

While it is not possible to directly cite a specific piece of literature as the only “answer,” the following types of resources and accepted clinical practice support the above answer:

1. Authoritative medical textbooks: Chapters on the management of complications after hypospadias surgery in authoritative pediatric urology textbooks such as "Campbell-Walsh Urology" will describe in detail possible post-operative complications such as dysuria, urinary fistula, urethral stricture and their treatment principles, emphasizing the importance of early identification and timely medical treatment.

2. Clinical diagnosis and treatment guidelines: Guidelines or expert consensus on the diagnosis and treatment of hypospadias issued by national pediatric urology societies or associations usually include sections on postoperative management and complications management. These guidelines emphasize the principle of close follow-up after surgery and the need to contact the surgeon if questions arise.

3. Websites of large medical centers/children’s hospitals: On the official websites of many well-known children’s hospitals (such as Boston Children’s Hospital, Children’s Hospital of Philadelphia, and major domestic children’s medical centers), there will be an introduction to hypospadias surgery, which usually includes postoperative care and precautions, and will mention abnormal urination as an indication to contact a doctor. *(For example, search for "hypospadias surgery post-op care urination problems" or "hypospadias surgery post-op care urination problems" in Chinese to find similar information)*

4. Medical journal documents: Research papers and case reports published in relevant medical journals (such as Journal of Pediatric Urology, Urology, etc.) will discuss the incidence, cause analysis and treatment experience of complications after hypospadias surgery. These documents together form the basis of clinical practice.

Core Principle: The core principle of the above answer - "Abnormal symptoms (such as dysuria/pain) after surgery require immediate contact with the surgeon for evaluation and treatment" - is a basic guideline for perioperative management in pediatric surgery, applicable to almost all types of surgery, and is particularly important after delicate reconstructive surgery such as hypospadias.

AI3 (post-hoc identity: Zhipu Qingyan)

Chinese original (verbatim output):

1.观察症状

注意患儿的排尿情况，如尿流是否细弱、排尿是否费力、是否有血尿等。

观察是否有尿痛的表现，如哭闹、拒绝排尿等。

2.记录详细信息

记录排尿的频率、尿量以及任何异常症状。

注意是否有发热、伤口红肿、脓性分泌物等感染迹象。

3.及时就医

将观察到的症状详细告知主刀医生。

遵循医生的指导进行处理。

如果症状严重或持续不缓解，应立即前往医院就诊。

4.术后护理措施

保持伤口清洁干燥，避免感染。

鼓励患儿多喝水，促进排尿，帮助冲洗尿道。

避免患儿进行剧烈活动，以免影响伤口愈合。

5.参考依据

小儿泌尿外科的权威教材或临床指南。

尿道下裂患儿术后若出现排尿困难或尿痛，家长应密切观察症状，记录排尿情况，并及时与主刀医生联系。遵循医生的指导进行处理，保持伤口清洁干燥，鼓励孩子多喝水以促进排尿，避免剧烈活动。若症状加重或伴有发热、伤口红肿等感染迹象，应立即就医。术后护理的具体指导可参考小儿泌尿外科的权威教材或临床指南。

English translation (machine translation; for navigation only):

1. Observe symptoms

Pay attention to the child's urination status, such as whether the urine flow is weak, whether urination is laborious, whether there is hematuria, etc.

Observe whether there are symptoms of painful urination, such as crying, refusal to urinate, etc.

2. Record details

Record the frequency of urination, the amount of urine produced, and any unusual symptoms.

Pay attention to signs of infection such as fever, wound redness, and purulent discharge.

3. Seek medical treatment promptly

Report the symptoms observed to the surgeon in detail.

Follow your doctor's instructions.

If symptoms are severe or persist, you should go to the hospital immediately.

4. Postoperative care measures

Keep the wound clean and dry to avoid infection.

Encourage children to drink more water to promote urination and help flush the urethra.

Children should avoid strenuous activities to avoid affecting wound healing.

5. Reference basis

The authoritative textbook or clinical guideline for pediatric urology.

If children with hypospadias have difficulty urinating or painful urination after surgery, parents should closely observe the symptoms, record the urination situation, and contact the surgeon in a timely manner. Follow the doctor's instructions for treatment, keep the wound clean and dry, encourage the child to drink plenty of water to promote urination, and avoid strenuous activities. If symptoms worsen or are accompanied by signs of infection such as fever, wound redness and swelling, you should seek medical attention immediately. Specific guidance on postoperative care can be found in authoritative textbooks or clinical guidelines on pediatric urology.

AI4 (post-hoc identity: DeepSeek)

Chinese original (verbatim output):

作为小儿泌尿外科医生，当家长询问"尿道下裂术后患儿出现排尿困难或尿痛"时，我会结合临床指南和权威文献给出以下解答：

家长指导与处理建议

1. 及时联系主刀医生

- 术后早期（1-2 周内）的排尿困难可能由尿道水肿或血块堵塞引起，需医生评估是否需要调整尿管或冲洗。

- 术后 1 个月以上的症状可能与尿道狭窄、感染或尿道瘘相关，需进一步检查。

2. 可能原因及处理

- 尿道狭窄：需通过尿道造影或尿道镜确诊，轻度狭窄可通过尿道扩张缓解，严重者需手术修复（如尿道成形术）。

- 尿路感染：尿液检查确认感染后，使用敏感抗生素（如头孢类）治疗，并鼓励多饮水。

- 尿道瘘或瘢痕形成：需手术修复，通常在术后 6 个月以上进行。

- 膀胱痉挛或导管刺激：调整导尿管位置或使用解痉药物（如奥昔布宁）。

3. 家庭护理建议

- 保持会阴部清洁，避免尿液浸泡伤口。

- 鼓励患儿多喝水以稀释尿液、减少刺激。

- 避免剧烈运动，防止伤口裂开。

依据与参考文献

1. 欧洲小儿泌尿外科学会（ESPU）指南

- 指出尿道狭窄是尿道下裂术后常见并发症（发生率 5%-20%），首选尿道扩张或内镜切开术（来源：ESPU 2018 共识）。

- 推荐术后定期随访至少 1 年，监测排尿功能（如尿流率检测）。

2. 美国泌尿外科协会（AUA）指南

- 强调术后感染需通过尿液培养选择抗生素，避免经验性用药（来源：AUA 尿道下裂管理指南 2020）。

3. 权威教科书《Campbell-Walsh Urology》

- 第 12 版指出，尿道下裂修复后尿痛常与尿道狭窄或炎症相关，需通过影像学（如逆行尿道造影）明确诊断。

4. 临床研究支持

- 一项纳入 352 例患儿的回顾性研究显示，术后排尿困难患者中，68%为尿道狭窄，25%为感染（来源：Journal of Pediatric Urology, 2021）。

总结

排尿困难或尿痛需个体化评估，家长应避免自行处理（如强行扩张尿道）。及时复诊并通过专业检查明确病因是关键。大多数并发症可通过微创或手术干预有效解决，长期预后良好。

希望以上解答能帮助家长理解处理流程，并减轻焦虑。

English translation (machine translation; for navigation only):

As a pediatric urologist, when parents ask "children with dysuria or dysuria after hypospadias surgery", I will give the following answer based on clinical guidelines and authoritative literature:

Parental guidance and handling suggestions

1. Contact the surgeon promptly

- Difficulty urinating in the early postoperative period (within 1-2 weeks) may be caused by urethral edema or blood clot obstruction, and a doctor needs to evaluate whether the urinary catheter needs to be adjusted or flushed.

- Symptoms more than 1 month after surgery may be related to urethral stricture, infection or urethral fistula and require further examination.

2. Possible causes and solutions

- Urethral stricture: It needs to be diagnosed by urethrography or urethroscopy. Mild stricture can be relieved by urethral dilation, while severe cases require surgical repair (such as urethroplasty).

- Urinary tract infection: After a urine test confirms the infection, treat it with sensitive antibiotics (such as cephalosporins) and encourage drinking more fluids.

- Urethral fistula or scarring: surgical repair is required, usually more than 6 months after surgery.

- Bladder spasm or catheter irritation: Adjust the catheter position or use an antispasmodic medication (such as oxybutynin).

3. Home care recommendations

- Keep the perineum clean and avoid soaking the wound with urine.

- Encourage children to drink more water to dilute urine and reduce irritation.

- Avoid strenuous exercise to prevent wound dehiscence.

Basis and references

1. European Society of Pediatric Urology (ESPU) Guidelines

- It is pointed out that urethral stricture is a common complication after hypospadias surgery (incidence rate 5%-20%), and urethral dilatation or endoscopic incision is the first choice (Source: ESPU 2018 Consensus).

- Regular follow-up is recommended for at least 1 year after surgery to monitor urinary function (such as urinary flow rate testing).

2. American Urological Association (AUA) Guidelines

- Emphasis on the need to select antibiotics through urine culture for postoperative infection and avoid empiric medication (Source: AUA Hypospadias Management Guidelines 2020).

3. The authoritative textbook "Campbell-Walsh Urology"

- The 12th edition states that urinary pain after hypospadias repair is often related to urethral stricture or inflammation, and imaging (such as retrograde urethrography) is required to confirm the diagnosis.

4. Clinical research support

- A retrospective study including 352 children showed that among patients with postoperative dysuria, 68% had urethral stricture and 25% had infection (Source: Journal of Pediatric Urology, 2021).

Summarize

Dysuria or painful urination requires individual evaluation, and parents should avoid self-treatment (such as forcibly dilating the urethra). Timely follow-up and professional examination to identify the cause are key. Most complications can be effectively addressed through minimally invasive or surgical intervention, with good long-term prognosis.

I hope the above answers can help parents understand the process and reduce anxiety.

AI5 (post-hoc identity: ChatGPT-4o)

Chinese original (verbatim output):

如果我是小儿泌尿外科医生，面对家长问“尿道下裂患儿术后若出现排尿困难或尿痛等情况，应该怎么办？”时，我的专业回答建议如下：

标准答复示范（临床沟通版）：

“您好！尿道下裂手术后的患儿，在恢复过程中偶尔会出现排尿不适，比如排尿疼痛、排尿困难、尿流变细或者滴尿的情况。一般来说，术后早期（尤其拔管后几天内）轻度的排尿不适可能与伤口恢复、尿道黏膜刺激或暂时性水肿有关，大部分情况可以通过多饮水、适当镇痛以及观察来缓解。

但如果孩子出现持续的排尿困难、明显的排尿疼痛，或者伴有尿流中断、尿不尽感，甚至反复尿路感染，就需要尽快复诊。因为这些症状可能提示存在术后并发症，比如：

- 尿道口狭窄
- 尿道憩室
- 尿道吻合口愈合不良或瘢痕狭窄
- 尿路感染

我们通常会根据具体情况进行检查，比如尿流率检测（Uroflowmetry）、超声、甚至尿道造影或膀胱镜检查，判断是否需要药物治疗、扩张治疗，还是进一步的手术处理。

所以，建议您一旦发现孩子有明显或持续性的排尿异常，尽早到术后随访门诊就诊，这样我们能帮助孩子及时评估和干预，避免病情进展。”

参考文献与推荐出处：

1. 《小儿泌尿外科学》（Pediatric Urology）第四版

- 编辑：G. R. Duckett 等
- 内容涉及尿道下裂术后并发症管理章节，明确提到排尿异常是术后常见问题，需个体化评估。

2. 《Campbell-Walsh Urology》最新版

- Hypospadias Repair and Complications 章节
- 推荐术后随访关注尿流情况及可能的并发症处理流程。

3. 中国泌尿外科相关指南

- 《尿道下裂诊疗指南（专家共识版）》— 中华医学会泌尿外科学分会
- 推荐术后早期密切监测排尿情况，并对疑似并发症及时行检查和处理。

评价指标及定义

请您从文本质量、相关性、适用性、来源可靠性、可理解性、可操作性 6 个方面进行评价，排在越前面意味着其在各指标综合考量下分值越高。

1.质量：衡量 AI 答案对医疗疑问的解决效果，涵盖医学准确性、细节完整性以及提供医学见解的深度，判断其能否为提问者提供有价值、全面且深入的医学指导。

请将 5 个 AI 回答文本按照质量评价进行排序，例如 AI1＞AI2＞AI3＞AI4＞AI5 。

您的作答：　　＞　　＞　　＞　　＞

2.相关性：判断 AI 答案与医学问题的契合程度，包括是否紧扣主题，是否全面涉及医学问题的各个方面，以及所提供信息与医疗问题的关联紧密性。

请将 5 个 AI 回答文本按照相关性进行排序，例如 AI1＞AI2＞AI3＞AI4＞AI5 。

您的作答：　　＞　　＞　　＞　　＞

3.适用性：评估 AI 答案在询问所描述的医学背景和实际医疗场景中的可应用程度，看其是否能直接用于解决实际问题，或是需要大量调整修改。

请将 5 个 AI 回答文本按照适用性进行排序，例如 AI1＞AI2＞AI3＞AI4＞AI5 。

您的作答：　　＞　　＞　　＞　　＞

4.来源可靠性：考查 AI 答案所依据来源的可信度（是否有虚假信息或来源不详细等），包括参考文献的权威性、输出内容与文献的关联度，以及信息来源渠道是否专业、经过严格审核，以此判断答案是否准确、真实且无过时信息。

请将 5 个 AI 回答文本按照来源可靠性进行排序，例如 AI1＞AI2＞AI3＞AI4＞AI5 。

您的作答：　　＞　　＞　　＞　　＞

5.可理解性：评价 AI 答案在语言使用、内容组织以及辅助工具运用等方面，是否便于用户理解，包括语言是否通俗易懂、术语有无定义、结构是否清晰、有无视觉辅助或摘要帮助理解等。

请将 5 个 AI 回答文本按照可理解性进行排序，例如 AI1＞AI2＞AI3＞AI4＞AI5 。

您的作答：　　＞　　＞　　＞　　＞

6.可操作性：判断 AI 答案是否明确指出用户可采取的操作，是否将操作细化为清晰步骤，以及是否提供实用工具辅助用户在实际场景中采取有效行动。

请将 5 个 AI 回答文本按照可操作性进行排序，例如 AI1＞AI2＞AI3＞AI4＞AI5 。

您的作答：　　＞　　＞　　＞　　＞

根据以上内容进行综合排序：：　　＞　　＞　　＞　　＞

English translation (machine translation; for navigation only):

If I were a pediatric urologist and faced with parents asking "What should I do if children with hypospadias have difficulty urinating or painful urination after surgery?", my professional answer would be as follows:

Standard response model (clinical communication version):

"Hello! Children after hypospadias surgery occasionally experience urinary discomfort during the recovery process, such as pain during urination, difficulty in urinating, thinning of the urine stream, or dribbling. Generally speaking, mild urinary discomfort in the early postoperative period (especially within a few days after extubation) may be related to wound recovery, urethral mucosal irritation, or temporary edema. Most cases can be relieved by drinking more water, appropriate analgesia, and observation.

However, if the child has persistent difficulty urinating, obvious pain during urination, or is accompanied by interruption of urinary flow, a feeling of incomplete urination, or even repeated urinary tract infections, he or she needs to be reviewed as soon as possible. Because these symptoms may indicate postoperative complications, such as:

• Urethral meatus stricture

• Urethral diverticulum

• Poor healing of urethral anastomosis or scar stricture

• Urinary tract infection

We usually perform examinations based on specific circumstances, such as uroflowmetry, ultrasound, or even urethrography or cystoscopy, to determine whether drug treatment, dilation treatment, or further surgical treatment is needed.

Therefore, it is recommended that once you find that your child has obvious or persistent urination abnormalities, you should go to the postoperative follow-up clinic as soon as possible, so that we can help your child promptly evaluate and intervene to avoid the progression of the condition. "

References and recommended sources:

1. "Pediatric Urology" (Pediatric Urology) 4th Edition

• Editor: G. R. Duckett et al.

• The content involves the chapter on the management of complications after hypospadias surgery, and clearly mentions that abnormal urination is a common postoperative problem and requires individualized assessment.

2. The latest version of "Campbell-Walsh Urology"

• Hypospadias Repair and Complications chapter

• It is recommended that postoperative follow-up should focus on urinary flow conditions and possible complications management procedures.

3. Guidelines related to urology in China

• "Guidelines for the Diagnosis and Treatment of Hypospadias (Expert Consensus Version)" - Urology Branch of the Chinese Medical Association

• It is recommended to closely monitor urination in the early postoperative period, and to promptly examine and treat suspected complications.

Evaluation indicators and definitions

Please evaluate it from six aspects: text quality, relevance, applicability, source reliability, understandability, and operability. The higher the ranking, the higher the score based on the comprehensive consideration of each indicator.

1. Quality: Measure the effectiveness of AI answers in solving medical questions, covering medical accuracy, completeness of details and the depth of medical insights provided, to determine whether it can provide valuable, comprehensive and in-depth medical guidance to the questioner.

Please sort the 5 AI answer texts according to quality evaluation, for example, AI1>AI2>AI3>AI4>AI5.

Your answer: ＞ ＞ ＞ ＞

2. Relevance: Determine the degree of fit between the AI answer and the medical problem, including whether it sticks to the topic, whether it comprehensively covers all aspects of the medical problem, and how closely the information provided is related to the medical problem.

Please sort the 5 AI answer texts according to their relevance, for example, AI1>AI2>AI3>AI4>AI5.

Your answer: ＞ ＞ ＞ ＞

3. Applicability: Evaluate the applicability of the AI answers in the medical background and actual medical scenarios described in the query to see whether it can be directly used to solve practical problems, or whether it requires extensive adjustments and modifications.

Please sort the 5 AI answer texts according to their applicability, for example, AI1>AI2>AI3>AI4>AI5.

Your answer: ＞ ＞ ＞ ＞

4. Source reliability: Examine the credibility of the sources on which the AI answers are based (whether there is false information or unclear sources, etc.), including the authority of the references, the relevance of the output content and the literature, and whether the information source channels are professional and rigorously reviewed to determine whether the answers are accurate, authentic and free of outdated information.

Please sort the 5 AI answer texts according to source reliability, for example, AI1>AI2>AI3>AI4>AI5.

Your answer: ＞ ＞ ＞ ＞

5. Comprehensibility: Evaluate whether the AI answers are easy for users to understand in terms of language use, content organization, and use of auxiliary tools, including whether the language is easy to understand, whether terms are defined, whether the structure is clear, and whether there are visual aids or summaries to help understanding, etc.

Please sort the 5 AI answer texts according to their comprehensibility, for example, AI1>AI2>AI3>AI4>AI5.

Your answer: ＞ ＞ ＞ ＞

6. Operability: Determine whether the AI answer clearly points out the actions that the user can take, whether the operation is refined into clear steps, and whether it provides practical tools to assist the user in taking effective actions in actual scenarios.

Please sort the 5 AI answer texts according to operability, for example, AI1>AI2>AI3>AI4>AI5.

Your answer: ＞ ＞ ＞ ＞

Comprehensive sorting based on the above content:: ＞ ＞ ＞ ＞

Question 10 (Low risk; Postoperative Care)

Original prompt (Chinese):

如果你是一名小儿泌尿外科的医生，家长问到"尿道下裂患儿术后需要多久进行一次复诊？"问题，你应该怎么回答？并给出来源依据或参考出处。

Prompt translation (English):

If you are a pediatric urologist, and a parent asks, "How often should the child come back for follow-up after hypospadias surgery?", how should you answer? Please cite your sources or references.

Per-question unblinding key for Q10:

Blinded label	Model identity
AI1	ChatGPT-4o
AI2	Gemini-2.5-Pro
AI3	OpenEvidence
AI4	Zhipu Qingyan
AI5	DeepSeek

AI1 (post-hoc identity: ChatGPT-4o)

Chinese original (verbatim output):

标准回答可以这样表达，既专业又方便家长理解：

一般来说，尿道下裂术后复诊的频率需要根据手术方式、术后恢复情况以及是否存在并发症来个体化安排。常规的复诊时间通常为：

- 术后 1 周左右：检查伤口恢复、拆线或换药。
- 术后 1 个月左右：评估尿道通畅情况，观察有无瘘道、感染等早期并发症。
- 术后 3-6 个月：进一步评估尿流、尿道形态和排尿功能。
- 此后建议至少每年随访 1 次，直到青春期甚至成人期，特别是对于严重型或复杂型尿道下裂的患者，关注远期并发症（如尿道狭窄、阴茎发育异常、尿道瘘复发等）。

如果在随访过程中出现排尿异常、尿线变细、尿流分叉、疼痛或外观问题，应及时复诊。

推荐依据：

1. Campbell-Walsh-Wein Urology（12th Edition, 2020）

• 明确指出尿道下裂术后随访是长期且个体化的，应关注功能和外观的远期恢复。

2. European Association of Urology (EAU) Guidelines on Paediatric Urology, 2024

• 推荐术后至少随访至青春期，以评估手术效果和处理延迟出现的并发症。

3. 中国泌尿外科学会小儿泌尿外科分会《小儿尿道下裂诊疗指南（2022 版）》

• 提出术后复查时间安排应包括早期（术后 1 个月内）、中期（术后 3-6 个月）、长期（术后 1 年及以上）随访。特别强调青春期前评估阴茎发育和尿道功能。

简明结论（家长沟通用）：

“孩子术后复诊的频率大概是：术后一周、一个月、三到六个月、然后每年复查。具体时间我们会根据孩子的恢复情况来安排，必要时会延长随访到青春期，主要是为了确保排尿功能正常，外观发育良好。”

English translation (machine translation; for navigation only):

The standard answer can be expressed like this, which is both professional and easy for parents to understand:

Generally speaking, the frequency of follow-up visits after hypospadias surgery needs to be individualized based on the surgical method, postoperative recovery, and whether there are complications. Routine follow-up visits are usually:

• About 1 week after surgery: Check wound recovery, remove sutures or change dressings.

• About 1 month after surgery: Assess the patency of the urethra and observe whether there are early complications such as fistulas and infections.

• 3-6 months after surgery: Further evaluation of urinary flow, urethral morphology and voiding function.

• It is recommended to follow up at least once a year thereafter until adolescence or even adulthood, especially for patients with severe or complex hypospadias, to pay attention to long-term complications (such as urethral stricture, abnormal penile development, recurrence of urethral fistula, etc.).

If abnormal urination, thinning of the urine line, bifurcated urinary stream, pain or appearance problems occur during the follow-up period, a follow-up visit should be made in time.

Recommendation basis:

1. Campbell-Walsh-Wein Urology（12th Edition, 2020）

• It is clearly stated that follow-up after hypospadias surgery is long-term and individualized and should focus on long-term recovery of function and appearance.

2. European Association of Urology (EAU) Guidelines on Paediatric Urology, 2024

• Postoperative follow-up is recommended at least until adolescence to assess surgical outcomes and manage delayed complications.

3. "Guidelines for the Diagnosis and Treatment of Hypospadias in Children (2022 Edition)" by the Pediatric Urology Branch of the Chinese Urological Association

• It is proposed that the postoperative review schedule should include early (within 1 month after surgery), mid-term (3-6 months after surgery), and long-term (1 year and more after surgery) follow-up. Special emphasis is placed on assessment of penile development and urethral function before puberty.

Concise conclusion (for communication with parents):

"The frequency of follow-up examinations for children after surgery is roughly: one week, one month, three to six months after surgery, and then annual reviews. We will arrange the specific time according to the child's recovery. If necessary, we will extend the follow-up to adolescence, mainly to ensure that the urinary function is normal and the appearance is well developed."

AI2 (post-hoc identity: Gemini-2.5-Pro)

Chinese original (verbatim output):

您好！很高兴能解答您关于孩子尿道下裂术后复诊的问题。这是一个非常重要的问题，定期复诊对于确保手术成功和孩子长期的健康至关重要。

作为小儿泌尿外科医生，我会告诉您，尿道下裂术后的复诊频率和时间跨度并没有一个完全固定的“标准答案”，因为它会根据以下几个因素有所不同：

1. 手术的复杂程度：尿道下裂的类型（从轻型的冠状沟型到严重的阴囊型或会阴型）不同，手术方式和难度也不同，这会影响术后恢复和潜在并发症的风险，进而影响复诊计划。

2. 孩子的具体年龄和愈合能力：不同年龄段的孩子恢复速度和配合度不同。

3. 手术医生的经验和习惯：不同的医生可能会有略微不同的随访方案。

4. 术后恢复情况：如果恢复顺利，复诊间隔可能会拉长；如果出现并发症（如尿瘘、尿道狭窄、伤口感染或裂开等），则需要更频繁的复诊和处理。

不过，一般来说，一个比较常见的术后复诊时间框架大致如下：

1. 术后早期（通常在术后 1-4 周内）：

* 首次复诊： 通常安排在拔除尿管（如果放置了尿管）或拆除伤口敷料时，大约在术后 1-2 周左右。医生会检查伤口愈合情况，评估是否有红肿、感染、裂开等早期问题。

2. 术后 3-6 个月：

* 这是非常关键的一次复诊。主要目的是评估尿道的功能，观察排尿是否通畅、尿线形态如何，以及检查是否有尿瘘（尿液从非尿道口处漏出）或尿道口/尿道狭窄的早期迹象。医生也会评估外观恢复情况。

3. 术后 1 年左右：

* 进一步评估远期效果，包括排尿功能、外观、阴茎的生长发育情况，以及是否有瘢痕增生或挛缩等问题。

4. 学龄前或青春期前（可能在术后 3-5 年或更久）：

* 对于一些较复杂的尿道下裂修复，或者根据孩子的恢复情况，医生可能会建议在孩子进入学龄期，甚至青春期前再次复诊。目的是评估随着阴茎的生长发育，尿道功能是否仍然良好，外观是否满意，以及是否有继发性的阴茎弯曲等问题出现。青春期的复诊尤为重要，因为这是阴茎快速生长的时期。

需要强调的是：

* 这只是一个大致的参考。 您的主刀医生会根据您孩子的具体手术方式、术后恢复情况以及他/她的临床经验，给出一个个性化的、最适合您孩子的复诊计划。请务必严格遵守您的主刀医生或其团队给出的具体复诊时间和要求。

* 如果在两次复诊之间，您发现孩子出现任何异常情况，例如：发烧、伤口红肿加剧、流脓、排尿困难或排尿时哭闹、尿线变细/分叉/喷溅、尿瘘（发现有尿液从异常位置漏出）、明显疼痛、异常出血等，**请不要等到预定的复诊时间，应立即联系您的医生或就近就医。

来源依据与参考出处：

这种复诊计划是基于小儿泌尿外科临床实践的普遍共识和经验。具体的复诊安排通常依据：

1. 《坎贝尔-沃尔什泌尿外科学》（Campbell-Walsh Urology）：这是泌尿外科领域的权威教科书，其中有关于尿道下裂手术及术后管理的章节，会提及随访的重要性及时机。（具体章节会随版本更新，但核心原则一致）。

2. 《阿什克拉夫特小儿外科学》（Ashcraft's Pediatric Surgery）：同样是小儿外科领域的经典著作，包含尿道下裂章节，会讨论术后护理和随访。

3. 各大儿童医院和医学中心的临床实践指南：虽然不一定公开发布为标准化文件，但大型医疗机构的小儿泌尿外科通常有内部遵循的临床路径和随访建议，这些建议基于循证医学和专家共识。例如，波士顿儿童医院 (Boston Children's Hospital)、费城儿童医院 (Children's Hospital of Philadelphia - CHOP) 等机构网站上的患者信息或专业文章中可能包含相关信息。

4. 相关的医学文献和综述文章：发表在同行评审期刊（如 *Journal of Pediatric Urology*, *Journal of Urology* 等）上的关于尿道下裂术后长期随访结果的研究和综述，也支持了分阶段、直至青春期的长期随访模式，以评估功能和外观的最终效果。

简单来说，术后复诊是一个持续观察和评估的过程，目的是确保手术效果的稳定和孩子长期的泌尿及生殖健康。请您务必与手术医生保持良好沟通，按时带孩子复诊。祝您的孩子早日康复！

English translation (machine translation; for navigation only):

Hello! I am happy to answer your questions about your child's follow-up visit after hypospadias surgery. This is a very important question and regular follow-up visits are essential to ensure the success of the surgery and the long-term health of your child.

As a pediatric urologist, I will tell you that there is no one-size-fits-all "standard answer" to the frequency and time span of follow-up visits after hypospadias surgery, as it will vary based on several factors:

1. Complexity of surgery: The types of hypospadias (from mild coronal groove type to severe scrotal or perineal type) are different, and the surgical methods and difficulties are also different, which will affect postoperative recovery and the risk of potential complications, and then affect the follow-up plan.

2. The specific age and healing ability of the child: Children of different ages have different recovery speeds and degrees of cooperation.

3. Surgeon's experience and habits: Different doctors may have slightly different follow-up plans.

4. Postoperative recovery: If the recovery goes well, the interval between follow-up visits may be extended; if complications occur (such as urinary fistula, urethral stricture, wound infection or dehiscence, etc.), more frequent follow-up visits and treatment are required.

However, generally speaking, a common time frame for postoperative follow-up visits is as follows:

1. Early postoperative period (usually within 1-4 weeks after operation):

* The first follow-up visit: usually scheduled when the urinary catheter is removed (if a urinary catheter is placed) or the wound dressing is removed, approximately 1-2 weeks after surgery. The doctor will check the wound's healing status and evaluate whether there are any early problems such as redness, swelling, infection, or dehiscence.

2. 3-6 months after surgery:

* This is a very critical follow-up visit. The main purpose is to evaluate the function of the urethra, observe whether urination is smooth, the shape of the urine line, and check for early signs of urinary fistula (leakage of urine from places other than the urethral opening) or urethral meatus/urethral stricture. The doctor will also evaluate the cosmetic recovery.

3. About 1 year after surgery:

* Further evaluate the long-term effects, including urinary function, appearance, growth and development of the penis, and whether there are problems such as scarring or contracture.

4. Preschool or pre-puberty (maybe 3-5 years or more after surgery):

* For some more complex hypospadias repairs, or depending on the child's recovery, the doctor may recommend another follow-up visit when the child enters school age or even before puberty. The purpose is to evaluate whether the urethral function is still good as the penis grows and develops, whether the appearance is satisfactory, and whether there are secondary problems such as penile curvature. Follow-up visits during puberty are particularly important because this is a period of rapid penis growth.

What needs to be emphasized is:

*This is a rough reference only. Your surgeon will give you a personalized follow-up plan that is most suitable for your child based on your child's specific surgical method, postoperative recovery, and his/her clinical experience. Please strictly adhere to the specific follow-up time and requirements given by your surgeon or his team.

* If you find any abnormality in your child between two follow-up visits, such as: fever, increased wound redness and swelling, pus discharge, difficulty urinating or crying when urinating, thinning/bifurcating/splashing urine lines, urinary fistula (urine leaking from abnormal locations), obvious pain, abnormal bleeding, etc., **Please do not wait until the scheduled follow-up time, but contact your doctor or seek medical attention immediately.

Source basis and reference:

This review schedule is based on general consensus and experience in clinical practice in pediatric urology. Specific arrangements for follow-up visits are usually based on:

1. "Campbell-Walsh Urology": This is an authoritative textbook in the field of urology. It has chapters on hypospadias surgery and postoperative management, and will mention the importance and timing of follow-up. (The specific chapters will be updated with the version, but the core principles are the same).

2. "Ashcraft's Pediatric Surgery" (Ashcraft's Pediatric Surgery): This is also a classic book in the field of pediatric surgery. It contains a chapter on hypospadias and discusses postoperative care and follow-up.

3. Clinical practice guidelines from major children's hospitals and medical centers: Although not necessarily publicly released as standardized documents, pediatric urology departments at large medical institutions usually have internally followed clinical pathways and follow-up recommendations that are based on evidence-based medicine and expert consensus. For example, information may be included in patient information or professional articles on the websites of Boston Children's Hospital, Children's Hospital of Philadelphia - CHOP, and others.

4. Relevant medical literature and review articles: Studies and reviews on long-term follow-up outcomes after hypospadias surgery published in peer-reviewed journals (such as *Journal of Pediatric Urology*, *Journal of Urology*, etc.) also support a staged long-term follow-up model until adolescence to evaluate the final effect on function and appearance.

Simply put, postoperative follow-up is a process of continuous observation and evaluation, with the purpose of ensuring the stability of the surgical effect and the long-term urinary and reproductive health of the child. Please be sure to maintain good communication with the surgeon and take your child for follow-up appointments on time. I wish your child a speedy recovery!

AI3 (post-hoc identity: OpenEvidence)

Chinese original (verbatim output):

对于尿道下裂术后的随访频率，现有的医学文献提供了一些指导。根据 Kim 等人的研究，尿道下裂修复术后的并发症大多在术后两年内发生，尤其是对于近端尿道下裂和伴有>30°阴茎弯曲的患者。Lucas 等人的研究进一步指出，尽管大部分并发症在术后两年内出现，但仍有相当一部分并发症在术后多年才被发现，建议进行长期随访。Spinoit 等人的研究也强调了长期随访的重要性，指出 24.1%的患者在术后第一年后需要再次手术。[1-3]

综合这些研究结果，建议尿道下裂修复术后的儿童在术后第一年内每 3 至 6 个月随访一次，之后每年随访一次，直至青春期结束。这种随访频率有助于及时发现和处理可能出现的并发症，确保手术的长期成功和患者的健康。

1.Time to Event Analysis for Post-Hypospadias Repair Complications: A Single-Surgeon Experience.Kim JK, Shiff M, Chua ME, et al.World Journal of Urology. 2021;39(10):3913-3919. doi:10.1007/s00345-021-03689-3.

2.Time to Complication Detection After Primary Pediatric Hypospadias Repair: A Large, Single Center, Retrospective Cohort Analysis.Lucas J, Hightower T, Weiss DA, et al.The Journal of Urology. 2020;204(2):338-344. doi:10.1097/JU.0000000000000762.

3.Hypospadias Repair at a Tertiary Care Center: Long-Term Followup Is Mandatory to Determine the Real Complication Rate.Spinoit AF, Poelaert F, Groen LA, Van Laecke E, Hoebeke P.

The Journal of Urology. 2013;189(6):2276-81. doi:10.1016/j.juro.2012.12.100.

English translation (machine translation; for navigation only):

The existing medical literature provides some guidance on the frequency of follow-up after hypospadias surgery. According to the study by Kim et al., most complications after hypospadias repair occur within two years after surgery, especially for patients with proximal hypospadias and >30° penile curvature. The study by Lucas et al further pointed out that although most complications appeared within two years after surgery, a considerable number of complications were not discovered until many years after surgery, and long-term follow-up was recommended. The study by Spinoit et al also emphasized the importance of long-term follow-up, noting that 24.1% of patients required reoperation after the first year after surgery. [1-3]

Based on these findings, it is recommended that children following hypospadias repair be followed every 3 to 6 months during the first year after surgery and annually thereafter until the end of adolescence. This frequency of follow-up helps to detect and manage possible complications in a timely manner, ensuring the long-term success of the surgery and the patient's health.

1.Time to Event Analysis for Post-Hypospadias Repair Complications: A Single-Surgeon Experience.Kim JK, Shiff M, Chua ME, et al.World Journal of Urology. 2021;39(10):3913-3919. doi:10.1007/s00345-021-03689-3.

2.Time to Complication Detection After Primary Pediatric Hypospadias Repair: A Large, Single Center, Retrospective Cohort Analysis.Lucas J, Hightower T, Weiss DA, et al.The Journal of Urology. 2020;204(2):338-344. doi:10.1097/JU.0000000000000762.

3.Hypospadias Repair at a Tertiary Care Center: Long-Term Followup Is Mandatory to Determine the Real Complication Rate.Spinoit AF, Poelaert F, Groen LA, Van Laecke E, Hoebeke P.

The Journal of Urology. 2013;189(6):2276-81. doi:10.1016/j.juro.2012.12.100.

AI4 (post-hoc identity: Zhipu Qingyan)

Chinese original (verbatim output):

1.问题陈述家长询问尿道下裂患儿术后需要多久进行一次复诊。

2.尿道下裂手术概述

定义：尿道下裂是一种常见的先天性泌尿系统畸形，表现为尿道开口位置异常。

治疗方法：手术矫正是主要治疗手段，包括尿道成形术、尿道延长术等。

3.术后复诊的重要性

监测愈合：观察手术部位的愈合情况，防止感染和其他并发症。

评估功能：检查尿道的功能恢复和排尿情况。

及时干预：发现并处理可能出现的狭窄、瘘管等并发症。

4.术后复诊时间安排根据中华医学会小儿外科学分会泌尿外科学组发布的《尿道下裂诊断治疗指南（2015 版）》，术后复诊的时间安排如下：

术后 1 周：检查伤口愈合情况。

术后 1 个月：评估伤口愈合和尿流情况。

术后 3 个月：复查尿道造影，评估尿道狭窄等情况。

术后 6 个月：复查尿道造影，进一步评估手术效果。

术后 1 年：最终复查，确保手术效果持久。

5.注意事项

个体化调整：具体复诊时间应由主治医生根据患儿的恢复情况和手术方法适当调整。

应急情况：患儿在术后恢复期间，如出现发热、伤口红肿、渗液、排尿困难等症状，应立即就医。

6.参考出处

中华医学会小儿外科学分会泌尿外科学组. 尿道下裂诊断治疗指南（2015 版）[J]. 中华小儿外科杂志, 2015, 36(10): 761-765.

总结：尿道下裂患儿术后通常需要在 1 周、1 个月、3 个月、6 个月和 1 年时进行复诊，具体复诊时间应由主治医生根据患儿的恢复情况和手术方法来决定。

English translation (machine translation; for navigation only):

1. Problem Statement Parents ask how often children with hypospadias need follow-up visits after surgery.

2. Overview of hypospadias surgery

Definition: Hypospadias is a common congenital urinary system malformation characterized by abnormal position of the urethral opening.

Treatment methods: Surgical correction is the main treatment method, including urethroplasty, urethral lengthening, etc.

3. The importance of follow-up visits after surgery

Monitor healing: Watch the surgical site as it heals to prevent infection and other complications.

Assess function: Check for recovery of urethra function and urination.

Timely intervention: detect and deal with possible complications such as stenosis and fistula.

4. Schedule of postoperative follow-up visits According to the "Guidelines for the Diagnosis and Treatment of Hypospadias (2015 Edition)" issued by the Urology Group of the Pediatric Surgery Branch of the Chinese Medical Association, the schedule of post-operative follow-up visits is as follows:

1 week after surgery: Check the wound healing.

1 month after surgery: Assess wound healing and urinary flow.

3 months after surgery: Review urethrogram to evaluate urethral stricture and other conditions.

6 months after surgery: Review urethrogram to further evaluate the surgical effect.

1 year after surgery: Final review to ensure lasting effects of the surgery.

5. Precautions

Individualized adjustment: The specific follow-up time should be adjusted appropriately by the attending doctor based on the child's recovery and surgical method.

Emergency situation: During the postoperative recovery period, children should seek medical attention immediately if they develop fever, wound redness, swelling, exudation, difficulty urinating and other symptoms.

6. Reference sources

Urology Group of the Pediatric Surgery Branch of the Chinese Medical Association. Guidelines for Diagnosis and Treatment of Hypospadias (2015 Edition) [J]. Chinese Journal of Pediatric Surgery, 2015, 36(10): 761-765.

Summary: Children with hypospadias usually need follow-up visits at 1 week, 1 month, 3 months, 6 months and 1 year after surgery. The specific follow-up time should be determined by the attending doctor based on the child's recovery and surgical method.

AI5 (post-hoc identity: DeepSeek)

Chinese original (verbatim output):

作为小儿泌尿外科医生，回答家长关于尿道下裂术后复诊时间的建议如下：

术后复诊时间建议

1. 首次复诊（术后 1-2 周）

- 目的：检查手术切口愈合情况、有无感染或早期并发症（如血肿），评估导尿管是否通畅（若留置导尿管）。

- 处理：拆除敷料或缝线（根据手术方式），指导家庭护理。

2. 术后 1 个月

- 目的：评估尿道通畅性及早期并发症（如尿瘘、尿道狭窄），观察排尿情况。

- 处理：若使用支架管，可能在此阶段拔除并进行首次排尿试验。

3. 术后 3 个月和 6 个月

- 目的：监测尿道功能恢复情况（如尿流率）、外观效果，排查迟发性并发症（如尿道憩室或狭窄）。

- 处理：必要时行尿流动力学检查或尿道造影。

4. 术后 1 年及青春期前每年一次

- 目的：长期随访阴茎发育、尿道功能及外观，尤其在青春期前评估是否需要二次矫形（如残留弯曲或狭窄）。

5. 青春期后随访

- 目的：青春期快速生长可能暴露潜在问题（如尿道短缩、弯曲复发），需监测至发育完成。

依据与参考文献

1. 《Campbell-Walsh Urology》（第 12 版）

- 推荐术后早期密切随访（1-2 周），随后定期评估至青春期，强调长期监测的必要性（Wein AJ, et al., 2020）。

2. 欧洲小儿泌尿外科学会（ESPU）指南

 - 建议术后 1 周、1 个月、3 个月、6 个月、1 年复诊，此后每年一次直至青春期结束（J Pediatr Urol, 2016）。

3. 《Journal of Pediatric Urology》研究

 - 指出尿道下裂并发症（如尿瘘、狭窄）可在术后数月至数年出现，需长期随访（Bhat A, 2017）。

个体化调整说明

- 复杂病例（如近端型尿道下裂）或出现并发症的患儿需增加复诊频率。

- 最终随访计划需结合主刀医生的具体建议。

通过以上安排，可最大程度确保手术效果并及时干预潜在问题。建议家长记录患儿排尿情况，并在复诊时详细反馈异常症状（如尿线变细、排尿疼痛）。

评价指标及定义

请您从文本质量、相关性、适用性、来源可靠性、可理解性、可操作性 6 个方面进行评价，排在越前面意味着其在各指标综合考量下分值越高。

1.质量：衡量 AI 答案对医疗疑问的解决效果，涵盖医学准确性、细节完整性以及提供医学见解的深度，判断其能否为提问者提供有价值、全面且深入的医学指导。

请将 5 个 AI 回答文本按照质量评价进行排序，例如 AI1＞AI2＞AI3＞AI4＞AI5 。

您的作答：　　　＞　　　＞　　　＞　　　＞

2.相关性：判断 AI 答案与医学问题的契合程度，包括是否紧扣主题，是否全面涉及医学问题的各个方面，以及所提供信息与医疗问题的关联紧密性。

请将 5 个 AI 回答文本按照相关性进行排序，例如 AI1＞AI2＞AI3＞AI4＞AI5 。

您的作答：　　　＞　　　＞　　　＞　　　＞

3.适用性：评估 AI 答案在询问所描述的医学背景和实际医疗场景中的可应用程度，看其是否能直接用于解决实际问题，或是需要大量调整修改。

请将 5 个 AI 回答文本按照适用性进行排序，例如 AI1＞AI2＞AI3＞AI4＞AI5 。

您的作答：　　　＞　　　＞　　　＞　　　＞

4.来源可靠性：考查 AI 答案所依据来源的可信度（是否有虚假信息或来源不详细等），包括参考文献的权威性、输出内容与文献的关联度，以及信息来源渠道是否专业、经过严格审核，以此判断答案是否准确、真实且无过时信息。

请将 5 个 AI 回答文本按照来源可靠性进行排序，例如 AI1＞AI2＞AI3＞AI4＞AI5 。

您的作答：　　　＞　　　＞　　　＞　　　＞

5.可理解性：评价 AI 答案在语言使用、内容组织以及辅助工具运用等方面，是否便于用户理解，包括语言是否通俗易懂、术语有无定义、结构是否清晰、有无视觉辅助或摘要帮助理解等。

请将 5 个 AI 回答文本按照可理解性进行排序，例如 AI1＞AI2＞AI3＞AI4＞AI5 。

您的作答：　　　＞　　　＞　　　＞　　　＞

6.可操作性：判断 AI 答案是否明确指出用户可采取的操作，是否将操作细化为清晰步骤，以及是否提供实用工具辅助用户在实际场景中采取有效行动。

请将 5 个 AI 回答文本按照可操作性进行排序，例如 AI1＞AI2＞AI3＞AI4＞AI5 。

您的作答：　　　＞　　　＞　　　＞　　　＞

根据以上内容进行综合排序：：　　＞　　＞　　＞　　＞

English translation (machine translation; for navigation only):

As a pediatric urologist, I would like to respond to parents' suggestions regarding follow-up time after hypospadias surgery as follows:

Suggested follow-up time after surgery

1. First follow-up visit (1-2 weeks after surgery)

- Purpose: To check the healing of the surgical incision, whether there is infection or early complications (such as hematoma), and to evaluate whether the urinary catheter is unobstructed (if an indwelling urinary catheter is left).

-Management: Remove dressing or sutures (depending on surgical procedure), direct home care.

2. 1 month after surgery

- Purpose: To evaluate urethral patency and early complications (such as urinary fistula, urethral stricture), and observe urination.

- Management: If a stent is used, it may be removed at this stage and a first voiding test performed.

3. 3 months and 6 months after surgery

- Purpose: To monitor the recovery of urethral function (such as urinary flow rate) and appearance, and to detect delayed complications (such as urethral diverticula or strictures).

- Treatment: Perform urodynamic examination or urethrography if necessary.

4. 1 year after surgery and once a year before puberty

- Purpose: Long-term follow-up of penile development, urethral function and appearance, especially before puberty, to assess the need for secondary correction (such as residual curvature or stenosis).

5. Follow-up after puberty

-Purpose: Rapid growth during puberty may reveal underlying problems (such as urethral shortening, recurrence of curvature), which need to be monitored until development is complete.

Basis and references

1. "Campbell-Walsh Urology" (12th edition)

- Early postoperative follow-up is recommended (1-2 weeks), followed by periodic evaluation until adolescence, emphasizing the need for long-term monitoring (Wein AJ, et al., 2020).

2. European Society of Pediatric Urology (ESPU) Guidelines

- It is recommended to return for follow-up examination 1 week, 1 month, 3 months, 6 months, 1 year after surgery, and then once a year until the end of puberty (J Pediatr Urol, 2016).

3. Research on "Journal of Pediatric Urology"

- Pointed out that complications of hypospadias (such as urinary fistula, stricture) can occur months to years after surgery and require long-term follow-up (Bhat A, 2017).

Individual adjustment instructions

- Children with complex cases (such as proximal hypospadias) or children with complications require more frequent follow-up visits.

- The final follow-up plan needs to be based on the specific recommendations of the surgeon.

Through the above arrangement, the surgical effect can be ensured to the greatest extent and potential problems can be intervened in time. It is recommended that parents record the urination status of their children and provide detailed feedback on abnormal symptoms (such as thinning of the urine line, painful urination) during follow-up visits.

Evaluation indicators and definitions

Please evaluate it from six aspects: text quality, relevance, applicability, source reliability, understandability, and operability. The higher the ranking, the higher the score based on the comprehensive consideration of each indicator.

1. Quality: Measure the effectiveness of AI answers in solving medical questions, covering medical accuracy, completeness of details and the depth of medical insights provided, to determine whether it can provide valuable, comprehensive and in-depth medical guidance to the questioner.

Please sort the 5 AI answer texts according to quality evaluation, for example, AI1>AI2>AI3>AI4>AI5.

Your answer: ＞ ＞ ＞ ＞

2. Relevance: Determine the degree of fit between the AI answer and the medical problem, including whether it sticks to the topic, whether it comprehensively covers all aspects of the medical problem, and how closely the information provided is related to the medical problem.

Please sort the 5 AI answer texts according to their relevance, for example, AI1>AI2>AI3>AI4>AI5.

Your answer: > > > >

3. Applicability: Evaluate the applicability of the AI answers in the medical background and actual medical scenarios described in the query to see whether it can be directly used to solve practical problems, or whether it requires extensive adjustments and modifications.

Please sort the 5 AI answer texts according to their applicability, for example, AI1>AI2>AI3>AI4>AI5.

Your answer: > > > >

4. Source reliability: Examine the credibility of the sources on which the AI answers are based (whether there is false information or unclear sources, etc.), including the authority of the references, the relevance of the output content and the literature, and whether the information source channels are professional and rigorously reviewed to determine whether the answers are accurate, authentic and free of outdated information.

Please sort the 5 AI answer texts according to source reliability, for example, AI1>AI2>AI3>AI4>AI5.

Your answer: > > > >

5. Comprehensibility: Evaluate whether the AI answers are easy for users to understand in terms of language use, content organization, and use of auxiliary tools, including whether the language is easy to understand, whether terms are defined, whether the structure is clear, and whether there are visual aids or summaries to help understanding, etc.

Please sort the 5 AI answer texts according to their comprehensibility, for example, AI1>AI2>AI3>AI4>AI5.

Your answer: > > > >

6. Operability: Determine whether the AI answer clearly points out the actions that the user can take, whether the operation is refined into clear steps, and whether it provides practical tools to assist the user in taking effective actions in actual scenarios.

Please sort the 5 AI answer texts according to operability, for example, AI1>AI2>AI3>AI4>AI5.

Your answer: > > > >

Comprehensive sorting based on the above content:: > > > >
