# Supplementary material for: Performance of 5 Large Language Models in Perioperative Consultation for Pediatric Hypospadias: Cross-Sectional Comparative Study
Source: J Med Internet Res. 2026 Jul 29;28:e93393. doi: 10.2196/93393 (PMC13419283; doi:10.2196/93393)
Supplement: Multimedia Appendix 6 [file jmir-v28-e93393-s006.docx]

**Evaluation dimension definitions and scoring procedure**

Expert instrument (six dimensions):

| Dimension | Operational definition |
| --- | --- |
| Quality | Effectiveness of the AI response in resolving the medical query, including medical accuracy, completeness of detail, and depth of clinical insight. |
| Relevance | How well the response matches the question, stays on topic, and addresses all aspects of the clinical query. |
| Applicability | How directly the response can be applied in the clinical scenario, or whether it requires substantial modification before use. |
| Source Reliability | Measures how trustworthy the referenced materials are. Scoring takes into account undisclosed or fabricated sources, the authority of cited works, and alignment between text and reference literature. |
| Comprehensibility | Judges the rationality of wording and content layout. Evaluation standards include plain phrasing, glossary explanations, clear structural logic, and supporting summaries or visual auxiliary materials. |
| Actionability | Whether the response gives specific, actionable steps the user can follow. |

Caregiver instrument (four dimensions):

| Dimension | Operational definition |
| --- | --- |
| Empathy | Recognize and understand users’ feelings, and interact with emotional resonance; assesses the system’s capacity to detect user sentiment and offer thoughtful, empathetic replies. |
| Addressing Concerns | Resolve user troubles and anxieties effectively; evaluates whether solutions are straightforward, usable, and sufficient to settle user questions. |
| Comprehensibility | Clarity and accessibility of information delivery — logical structure, term explanation, and visual/structural aids. |
| Actionability | Make guidance suitable for practical use in daily scenarios; gauges if instructions are specific enough to guide users through concrete steps. |

Scoring procedure: a double-blind forced-ranking method with reverse scoring (1st place = 5 points; 5th place = 1 point). Each evaluator distributed the values 5, 4, 3, 2, 1 exactly once across the five models per question × dimension cell. All raters received a standardised briefing and calibration session prior to formal evaluation.
