# Supplementary material for: Performance of 5 Large Language Models in Perioperative Consultation for Pediatric Hypospadias: Cross-Sectional Comparative Study
Source: J Med Internet Res. 2026 Jul 29;28:e93393. doi: 10.2196/93393 (PMC13419283; doi:10.2196/93393)
Supplement: Multimedia Appendix 7 [file jmir-v28-e93393-s007.pdf]

Complete pairwise comparison tables

Part A. Overall pairwise comparisons (Bonferroni-adjusted α’=.005; 20 rows = 10 pairs × 2 perspectives).

Perspective	Model A	Model B	Score Model A	Score Model B	*P*-adj	Effect Size *r*	Diff 95% CI
Caregiver	ChatGPT-4o	DeepSeek	2.6 [2.4 to 2.9] (95% CI 2.5 to 2.8)	3.5 [3.2 to 3.6] (95% CI 3.3 to 3.5)	**<.001**	−0.773	−1.04 to −0.50
Caregiver	ChatGPT-4o	Gemini-2.5-Pro	2.6 [2.4 to 2.9] (95% CI 2.5 to 2.8)	4.0 [3.3 to 4.4] (95% CI 3.5 to 4.2)	**<.001**	−0.893	−1.61 to −0.90
Caregiver	ChatGPT-4o	Open-Evidence	2.6 [2.4 to 2.9] (95% CI 2.5 to 2.8)	1.9 [1.6 to 2.4] (95% CI 1.6 to 2.2)	**<.001**	0.904	0.46 to 0.81
Caregiver	ChatGPT-4o	Zhipu Qingyan	2.6 [2.4 to 2.9] (95% CI 2.5 to 2.8)	3.1 [2.8 to 3.2] (95% CI 2.9 to 3.2)	0.004	−0.674	−0.62 to −0.21
Caregiver	DeepSeek	Gemini-2.5-Pro	3.5 [3.2 to 3.6] (95% CI 3.3 to 3.5)	4.0 [3.3 to 4.4] (95% CI 3.5 to 4.2)	0.002	−0.716	−0.75 to −0.24
Caregiver	DeepSeek	Open-Evidence	3.5 [3.2 to 3.6] (95% CI 3.3 to 3.5)	1.9 [1.6 to 2.4] (95% CI 1.6 to 2.2)	**<.001**	0.934	1.14 to 1.76
Caregiver	DeepSeek	Zhipu Qingyan	3.5 [3.2 to 3.6] (95% CI 3.3 to 3.5)	3.1 [2.8 to 3.2] (95% CI 2.9 to 3.2)	0.007	0.657	0.14 to 0.49
Caregiver	Gemini-2.5-Pro	OpenEvidence	4.0 [3.3 to 4.4] (95% CI 3.5 to 4.2)	1.9 [1.6 to 2.4] (95% CI 1.6 to 2.2)	**<.001**	0.952	1.46 to 2.41
Caregiver	Gemini-2.5-Pro	Zhipu Qingyan	4.0 [3.3 to 4.4] (95% CI 3.5 to 4.2)	3.1 [2.8 to 3.2] (95% CI 2.9 to 3.2)	**<.001**	0.839	0.55 to 1.15
Caregiver	Open-Evidence	Zhipu Qingyan	1.9 [1.6 to 2.4] (95% CI 1.6 to 2.2)	3.1 [2.8 to 3.2] (95% CI 2.9 to 3.2)	**<.001**	−0.943	−1.34 to −0.80
Expert	ChatGPT-4o	DeepSeek	2.8 [2.7 to 3.2] (95% CI 2.7 to 3.1)	3.5 [3.3 to 3.7] (95% CI 3.3 to 3.7)	0.004	−0.851	−0.83 to −0.28

Perspective	Model A	Model B	Score Model A	Score Model B	*P*-adj	Effect Size *r*	Diff 95% CI
Expert	ChatGPT-4o	Gemini-2.5-Pro	2.8 [2.7 to 3.2] (95% CI 2.7 to 3.1)	4.0 [3.7 to 4.6] (95% CI 3.8 to 4.6)	0.002	−0.921	−1.62 to −0.78
Expert	ChatGPT-4o	Open-Evidence	2.8 [2.7 to 3.2] (95% CI 2.7 to 3.1)	1.8 [1.6 to 2.2] (95% CI 1.6 to 2.1)	**<.001**	1.000	0.73 to 1.18
Expert	ChatGPT-4o	Zhipu Qingyan	2.8 [2.7 to 3.2] (95% CI 2.7 to 3.1)	2.6 [2.5 to 2.8] (95% CI 2.5 to 2.8)	0.642	0.455	−0.02 to 0.63
Expert	DeepSeek	Gemini-2.5-Pro	3.5 [3.3 to 3.7] (95% CI 3.3 to 3.7)	4.0 [3.7 to 4.6] (95% CI 3.8 to 4.6)	0.042	−0.685	−0.95 to −0.22
Expert	DeepSeek	Open-Evidence	3.5 [3.3 to 3.7] (95% CI 3.3 to 3.7)	1.8 [1.6 to 2.2] (95% CI 1.6 to 2.1)	**<.001**	1.000	1.27 to 1.83
Expert	DeepSeek	Zhipu Qingyan	3.5 [3.3 to 3.7] (95% CI 3.3 to 3.7)	2.6 [2.5 to 2.8] (95% CI 2.5 to 2.8)	**<.001**	0.964	0.63 to 1.13
Expert	Gemini-2.5-Pro	Open-Evidence	4.0 [3.7 to 4.6] (95% CI 3.8 to 4.6)	1.8 [1.6 to 2.2] (95% CI 1.6 to 2.1)	**<.001**	0.992	1.83 to 2.63
Expert	Gemini-2.5-Pro	Zhipu Qingyan	4.0 [3.7 to 4.6] (95% CI 3.8 to 4.6)	2.6 [2.5 to 2.8] (95% CI 2.5 to 2.8)	**<.001**	0.986	1.03 to 1.78
Expert	Open-Evidence	Zhipu Qingyan	1.8 [1.6 to 2.2] (95% CI 1.6 to 2.1)	2.6 [2.5 to 2.8] (95% CI 2.5 to 2.8)	0.004	−0.855	−0.95 to −0.40

Part B. Dimension-level descriptive statistics (median [IQR] (95% CI); 100 rows = 10 dimensions × 5 models × 2 perspectives).

Perspective	Dimension_En	Model	n	Median	Q1	Q3	CI_lower	CI_upper
Caregiver	Actionability	ChatGPT-4o	360	2	2	4	2	3
Caregiver	Actionability	DeepSeek	360	4	3	4	3	4
Caregiver	Actionability	Gemini-2.5-Pro	360	4	3	5	4	4
Caregiver	Actionability	OpenEvidence	360	1	1	2.25	1	2
Caregiver	Actionability	Zhipu Qingyan	360	3	2	4	3	3
Caregiver	Addressing Concerns	ChatGPT-4o	360	2	2	3	2	3

Perspective	Dimension_En	Model	n	Median	Q1	Q3	CI_lower	CI_upper
Caregiver	Addressing Concerns	DeepSeek	360	4	3	4	3	4
Caregiver	Addressing Concerns	Gemini-2.5-Pro	360	5	3	5	4	5
Caregiver	Addressing Concerns	OpenEvidence	360	2	1	3	1	2
Caregiver	Addressing Concerns	Zhipu Qingyan	360	3	2	4	3	3
Caregiver	Comprehensibility	ChatGPT-4o	360	2	2	4	2	3
Caregiver	Comprehensibility	DeepSeek	360	4	3	4	3	4
Caregiver	Comprehensibility	Gemini-2.5-Pro	360	4	3	5	4	5
Caregiver	Comprehensibility	OpenEvidence	360	2	1	3	1	2
Caregiver	Comprehensibility	Zhipu Qingyan	360	3	2	4	3	3
Caregiver	Empathy	ChatGPT-4o	360	2.5	2	4	2	3
Caregiver	Empathy	DeepSeek	360	3	3	4	3	4
Caregiver	Empathy	Gemini-2.5-Pro	360	5	3	5	5	5
Caregiver	Empathy	OpenEvidence	360	2	1	3	2	2
Caregiver	Empathy	Zhipu Qingyan	360	3	2	4	3	3
Expert	Actionability	ChatGPT-4o	230	3	2	4	3	3
Expert	Actionability	DeepSeek	230	4	3	4	3	4
Expert	Actionability	Gemini-2.5-Pro	230	4	3	5	4	5
Expert	Actionability	OpenEvidence	230	2	1	2	1	2
Expert	Actionability	Zhipu Qingyan	230	3	2	4	3	3
Expert	Applicability	ChatGPT-4o	230	3	2	4	3	3
Expert	Applicability	DeepSeek	230	4	3	4	3	4
Expert	Applicability	Gemini-2.5-Pro	230	5	3	5	4	5
Expert	Applicability	OpenEvidence	230	2	1	3	1	2
Expert	Applicability	Zhipu Qingyan	230	3	2	4	2	3
Expert	Comprehensibility	ChatGPT-4o	230	3	2	4	3	3
Expert	Comprehensibility	DeepSeek	230	4	2	4	3	4
Expert	Comprehensibility	Gemini-2.5-Pro	230	4	3	5	4	5
Expert	Comprehensibility	OpenEvidence	230	1	1	2	1	2
Expert	Comprehensibility	Zhipu Qingyan	230	3	2	4	3	3
Expert	Quality	ChatGPT-4o	230	3	2	3	2	3
Expert	Quality	DeepSeek	230	4	3	5	4	4
Expert	Quality	Gemini-2.5-Pro	230	5	4	5	5	5
Expert	Quality	OpenEvidence	230	1	1	2	1	2
Expert	Quality	Zhipu Qingyan	230	2	2	3	2	3

Perspective	Dimension_En	Model	n	Median	Q1	Q3	CI_lower	CI_upper
Expert	Relevance	ChatGPT-4o	230	3	2	4	3	3
Expert	Relevance	DeepSeek	230	4	3	4	4	4
Expert	Relevance	Gemini-2.5-Pro	230	5	3	5	4	5
Expert	Relevance	OpenEvidence	230	2	1	2	1	2
Expert	Relevance	Zhipu Qingyan	230	2	1	4	2	3
Expert	Source Reliability	ChatGPT-4o	230	3	2	4	3	3
Expert	Source Reliability	DeepSeek	230	3	2	4	3	4
Expert	Source Reliability	Gemini-2.5-Pro	230	5	3	5	4	5
Expert	Source Reliability	OpenEvidence	230	2	1	3	2	2
Expert	Source Reliability	Zhipu Qingyan	230	2	1	3	2	2

Part C. Dimension-level pairwise comparisons (Bonferroni-adjusted; 100 rows = 10 dimensions × 10 pairs).

Perspective	Dimension	Model A	Model B	Score Model A	Score Model B	*P*-adj	Effect Size *r*	Diff 95% CI
Caregiver	Empathy	ChatGPT-4o	DeepSeek	2.5 [2.0 to 4.0] (95% CI 2.0 to 3.0)	3.0 [3.0 to 4.0] (95% CI 3.0 to 4.0)	**<.001**	−0.343	−1.00 to −0.50
Caregiver	Empathy	ChatGPT-4o	Gemini-2.5-Pro	2.5 [2.0 to 4.0] (95% CI 2.0 to 3.0)	5.0 [3.0 to 5.0] (95% CI 5.0 to 5.0)	**<.001**	−0.601	−2.00 to −1.00
Caregiver	Empathy	ChatGPT-4o	Open-Evidence	2.5 [2.0 to 4.0] (95% CI 2.0 to 3.0)	2.0 [1.0 to 3.0] (95% CI 2.0 to 2.0)	**<.001**	0.341	0.50 to 1.00
Caregiver	Empathy	ChatGPT-4o	Zhipu Qingyan	2.5 [2.0 to 4.0] (95% CI 2.0 to 3.0)	3.0 [2.0 to 4.0] (95% CI 3.0 to 3.0)	0.515	−0.117	−0.50 to 0.00
Caregiver	Empathy	DeepSeek	Gemini-2.5-Pro	3.0 [3.0 to 4.0] (95% CI 3.0 to 4.0)	5.0 [3.0 to 5.0] (95% CI 5.0 to 5.0)	**<.001**	−0.415	−1.00 to −0.50
Caregiver	Empathy	DeepSeek	Open-Evidence	3.0 [3.0 to 4.0] (95% CI 3.0 to	2.0 [1.0 to 3.0] (95% CI 2.0 to	**<.001**	0.661	1.00 to 1.50

Perspective	Dimension	Model A	Model B	Score Model A	Score Model B	*P*-adj	Effect Size *r*	Diff 95% CI
				4.0)	2.0)			
Caregiver	Empathy	DeepSeek	Zhipu Qingyan	3.0 [3.0 to 4.0] (95% CI 3.0 to 4.0)	3.0 [2.0 to 4.0] (95% CI 3.0 to 3.0)	**<.001**	0.242	0.00 to 0.50
Caregiver	Empathy	Gemini-2.5-Pro	Open-Evidence	5.0 [3.0 to 5.0] (95% CI 5.0 to 5.0)	2.0 [1.0 to 3.0] (95% CI 2.0 to 2.0)	**<.001**	0.733	2.00 to 2.50
Caregiver	Empathy	Gemini-2.5-Pro	Zhipu Qingyan	5.0 [3.0 to 5.0] (95% CI 5.0 to 5.0)	3.0 [2.0 to 4.0] (95% CI 3.0 to 3.0)	**<.001**	0.531	1.00 to 1.50
Caregiver	Empathy	Open-Evidence	Zhipu Qingyan	2.0 [1.0 to 3.0] (95% CI 2.0 to 2.0)	3.0 [2.0 to 4.0] (95% CI 3.0 to 3.0)	**<.001**	−0.496	−1.00 to −1.00
Caregiver	Addressing Concerns	ChatGPT-4o	DeepSeek	2.0 [2.0 to 3.0] (95% CI 2.0 to 3.0)	4.0 [3.0 to 4.0] (95% CI 3.0 to 4.0)	**<.001**	−0.425	−1.00 to −0.50
Caregiver	Addressing Concerns	ChatGPT-4o	Gemini-2.5-Pro	2.0 [2.0 to 3.0] (95% CI 2.0 to 3.0)	5.0 [3.0 to 5.0] (95% CI 4.0 to 5.0)	**<.001**	−0.631	−2.00 to −1.50
Caregiver	Addressing Concerns	ChatGPT-4o	Open-Evidence	2.0 [2.0 to 3.0] (95% CI 2.0 to 3.0)	2.0 [1.0 to 3.0] (95% CI 1.0 to 2.0)	**<.001**	0.330	0.50 to 1.00
Caregiver	Addressing Concerns	ChatGPT-4o	Zhipu Qingyan	2.0 [2.0 to 3.0] (95% CI 2.0 to 3.0)	3.0 [2.0 to 4.0] (95% CI 3.0 to 3.0)	0.001	−0.228	−0.50 to −0.00
Caregiver	Addressing Concerns	DeepSeek	Gemini-2.5-Pro	4.0 [3.0 to 4.0] (95% CI 3.0 to 4.0)	5.0 [3.0 to 5.0] (95% CI 4.0 to 5.0)	**<.001**	−0.316	−1.00 to −0.50

Perspective	Dimension	Model A	Model B	Score Model A	Score Model B	*P*-adj	Effect Size *r*	Diff 95% CI
Caregiver	Addressing Concerns	DeepSeek	Open-Evidence	4.0 [3.0 to 4.0] (95% CI 3.0 to 4.0)	2.0 [1.0 to 3.0] (95% CI 1.0 to 2.0)	**<.001**	0.628	1.00 to 2.00
Caregiver	Addressing Concerns	DeepSeek	Zhipu Qingyan	4.0 [3.0 to 4.0] (95% CI 3.0 to 4.0)	3.0 [2.0 to 4.0] (95% CI 3.0 to 3.0)	0.002	0.225	0.00 to 0.50
Caregiver	Addressing Concerns	Gemini-2.5-Pro	Open-Evidence	5.0 [3.0 to 5.0] (95% CI 4.0 to 5.0)	2.0 [1.0 to 3.0] (95% CI 1.0 to 2.0)	**<.001**	0.730	2.00 to 2.50
Caregiver	Addressing Concerns	Gemini-2.5-Pro	Zhipu Qingyan	5.0 [3.0 to 5.0] (95% CI 4.0 to 5.0)	3.0 [2.0 to 4.0] (95% CI 3.0 to 3.0)	**<.001**	0.479	1.00 to 1.50
Caregiver	Addressing Concerns	Open-Evidence	Zhipu Qingyan	2.0 [1.0 to 3.0] (95% CI 1.0 to 2.0)	3.0 [2.0 to 4.0] (95% CI 3.0 to 3.0)	**<.001**	−0.511	−1.50 to −1.00
Caregiver	Comprehensibility	ChatGPT-4o	DeepSeek	2.0 [2.0 to 4.0] (95% CI 2.0 to 3.0)	4.0 [3.0 to 4.0] (95% CI 3.0 to 4.0)	**<.001**	−0.400	−1.00 to −0.50
Caregiver	Comprehensibility	ChatGPT-4o	Gemini-2.5-Pro	2.0 [2.0 to 4.0] (95% CI 2.0 to 3.0)	4.0 [3.0 to 5.0] (95% CI 4.0 to 5.0)	**<.001**	−0.535	−1.50 to −1.00
Caregiver	Comprehensibility	ChatGPT-4o	OpenEvidence	2.0 [2.0 to 4.0] (95% CI 2.0 to 3.0)	2.0 [1.0 to 3.0] (95% CI 1.0 to 2.0)	**<.001**	0.359	0.50 to 1.00
Caregiver	Comprehensibility	ChatGPT-4o	Zhipu Qingyan	2.0 [2.0 to 4.0] (95% CI 2.0 to 3.0)	3.0 [2.0 to 4.0] (95% CI 3.0 to 3.0)	0.002	−0.223	−1.00 to −0.00
Caregiver	Comprehensibility	DeepSeek	Gemini-2.5-Pro	4.0 [3.0 to 4.0] (95% CI 3.0 to 4.0)	4.0 [3.0 to 5.0] (95% CI 4.0 to 5.0)	0.001	−0.228	−0.50 to −0.00

Perspective	Dimension	Model A	Model B	Score Model A	Score Model B	*P*-adj	Effect Size *r*	Diff 95% CI
Caregiver	Comprehensibility	DeepSeek	Open-Evidence	4.0 [3.0 to 4.0] (95% CI 3.0 to 4.0)	2.0 [1.0 to 3.0] (95% CI 1.0 to 2.0)	**<.001**	0.644	1.50 to 2.00
Caregiver	Comprehensibility	DeepSeek	Zhipu Qingyan	4.0 [3.0 to 4.0] (95% CI 3.0 to 4.0)	3.0 [2.0 to 4.0] (95% CI 3.0 to 3.0)	0.033	0.175	0.00 to 0.50
Caregiver	Comprehensibility	Gemini-2.5-Pro	Open-Evidence	4.0 [3.0 to 5.0] (95% CI 4.0 to 5.0)	2.0 [1.0 to 3.0] (95% CI 1.0 to 2.0)	**<.001**	0.713	2.00 to 2.50
Caregiver	Comprehensibility	Gemini-2.5-Pro	Zhipu Qingyan	4.0 [3.0 to 5.0] (95% CI 4.0 to 5.0)	3.0 [2.0 to 4.0] (95% CI 3.0 to 3.0)	**<.001**	0.340	0.50 to 1.00
Caregiver	Comprehensibility	Open-Evidence	Zhipu Qingyan	2.0 [1.0 to 3.0] (95% CI 1.0 to 2.0)	3.0 [2.0 to 4.0] (95% CI 3.0 to 3.0)	**<.001**	−0.542	−1.50 to −1.00
Caregiver	Actionability	ChatGPT-4o	DeepSeek	2.0 [2.0 to 4.0] (95% CI 2.0 to 3.0)	4.0 [3.0 to 4.0] (95% CI 3.0 to 4.0)	**<.001**	−0.414	−1.00 to −0.50
Caregiver	Actionability	ChatGPT-4o	Gemini-2.5-Pro	2.0 [2.0 to 4.0] (95% CI 2.0 to 3.0)	4.0 [3.0 to 5.0] (95% CI 4.0 to 4.0)	**<.001**	−0.530	−1.50 to −1.00
Caregiver	Actionability	ChatGPT-4o	Open-Evidence	2.0 [2.0 to 4.0] (95% CI 2.0 to 3.0)	1.0 [1.0 to 2.2] (95% CI 1.0 to 2.0)	**<.001**	0.434	1.00 to 1.00
Caregiver	Actionability	ChatGPT-4o	Zhipu Qingyan	2.0 [2.0 to 4.0] (95% CI 2.0 to 3.0)	3.0 [2.0 to 4.0] (95% CI 3.0 to 3.0)	**<.001**	−0.262	−1.00 to −0.00
Caregiver	Actionability	DeepSeek	Gemini-2.5-Pro	4.0 [3.0 to 4.0] (95% CI 3.0 to 4.0)	4.0 [3.0 to 5.0] (95% CI 4.0 to 4.0)	0.002	−0.220	−0.50 to −0.00

Perspective	Dimension	Model A	Model B	Score Model A	Score Model B	*P*-adj	Effect Size *r*	Diff 95% CI
Caregiver	Actionability	DeepSeek	Open-Evidence	4.0 [3.0 to 4.0] (95% CI 3.0 to 4.0)	1.0 [1.0 to 2.2] (95% CI 1.0 to 2.0)	**<.001**	0.696	1.50 to 2.00
Caregiver	Actionability	DeepSeek	Zhipu Qingyan	4.0 [3.0 to 4.0] (95% CI 3.0 to 4.0)	3.0 [2.0 to 4.0] (95% CI 3.0 to 3.0)	0.046	0.169	0.00 to 0.50
Caregiver	Actionability	Gemini-2.5-Pro	Open-Evidence	4.0 [3.0 to 5.0] (95% CI 4.0 to 4.0)	1.0 [1.0 to 2.2] (95% CI 1.0 to 2.0)	**<.001**	0.754	2.00 to 2.50
Caregiver	Actionability	Gemini-2.5-Pro	Zhipu Qingyan	4.0 [3.0 to 5.0] (95% CI 4.0 to 4.0)	3.0 [2.0 to 4.0] (95% CI 3.0 to 3.0)	**<.001**	0.352	0.50 to 1.00
Caregiver	Actionability	Open-Evidence	Zhipu Qingyan	1.0 [1.0 to 2.2] (95% CI 1.0 to 2.0)	3.0 [2.0 to 4.0] (95% CI 3.0 to 3.0)	**<.001**	−0.647	−1.50 to −1.50
Expert	Quality	ChatGPT-4o	DeepSeek	3.0 [2.0 to 3.0] (95% CI 2.0 to 3.0)	4.0 [3.0 to 5.0] (95% CI 4.0 to 4.0)	**<.001**	−0.587	−1.50 to −1.00
Expert	Quality	ChatGPT-4o	Gemini-2.5-Pro	3.0 [2.0 to 3.0] (95% CI 2.0 to 3.0)	5.0 [4.0 to 5.0] (95% CI 5.0 to 5.0)	**<.001**	−0.793	−2.00 to −1.50
Expert	Quality	ChatGPT-4o	Open-Evidence	3.0 [2.0 to 3.0] (95% CI 2.0 to 3.0)	1.0 [1.0 to 2.0] (95% CI 1.0 to 2.0)	**<.001**	0.552	1.00 to 1.00
Expert	Quality	ChatGPT-4o	Zhipu Qingyan	3.0 [2.0 to 3.0] (95% CI 2.0 to 3.0)	2.0 [2.0 to 3.0] (95% CI 2.0 to 3.0)	>.99	0.074	−0.00 to 0.50
Expert	Quality	DeepSeek	Gemini-2.5-Pro	4.0 [3.0 to 5.0] (95% CI 4.0 to 4.0)	5.0 [4.0 to 5.0] (95% CI 5.0 to 5.0)	**<.001**	−0.398	−1.00 to −0.50

Perspective	Dimension	Model A	Model B	Score Model A	Score Model B	*P*-adj	Effect Size *r*	Diff 95% CI
Expert	Quality	DeepSeek	Open-Evidence	4.0 [3.0 to 5.0] (95% CI 4.0 to 4.0)	1.0 [1.0 to 2.0] (95% CI 1.0 to 2.0)	**<.001**	0.875	2.00 to 2.50
Expert	Quality	DeepSeek	Zhipu Qingyan	4.0 [3.0 to 5.0] (95% CI 4.0 to 4.0)	2.0 [2.0 to 3.0] (95% CI 2.0 to 3.0)	**<.001**	0.649	1.00 to 1.50
Expert	Quality	Gemini-2.5-Pro	Open-Evidence	5.0 [4.0 to 5.0] (95% CI 5.0 to 5.0)	1.0 [1.0 to 2.0] (95% CI 1.0 to 2.0)	**<.001**	0.914	2.50 to 3.00
Expert	Quality	Gemini-2.5-Pro	Zhipu Qingyan	5.0 [4.0 to 5.0] (95% CI 5.0 to 5.0)	2.0 [2.0 to 3.0] (95% CI 2.0 to 3.0)	**<.001**	0.847	2.00 to 2.00
Expert	Quality	Open-Evidence	Zhipu Qingyan	1.0 [1.0 to 2.0] (95% CI 1.0 to 2.0)	2.0 [2.0 to 3.0] (95% CI 2.0 to 3.0)	**<.001**	−0.473	−1.00 to −0.50
Expert	Relevance	ChatGPT-4o	DeepSeek	3.0 [2.0 to 4.0] (95% CI 3.0 to 3.0)	4.0 [3.0 to 4.0] (95% CI 4.0 to 4.0)	**<.001**	−0.447	−1.00 to −0.50
Expert	Relevance	ChatGPT-4o	Gemini-2.5-Pro	3.0 [2.0 to 4.0] (95% CI 3.0 to 3.0)	5.0 [3.0 to 5.0] (95% CI 4.0 to 5.0)	**<.001**	−0.563	−1.50 to −1.00
Expert	Relevance	ChatGPT-4o	Open-Evidence	3.0 [2.0 to 4.0] (95% CI 3.0 to 3.0)	2.0 [1.0 to 2.0] (95% CI 1.0 to 2.0)	**<.001**	0.582	1.00 to 1.50
Expert	Relevance	ChatGPT-4o	Zhipu Qingyan	3.0 [2.0 to 4.0] (95% CI 3.0 to 3.0)	2.0 [1.0 to 4.0] (95% CI 2.0 to 3.0)	0.161	0.180	0.00 to 0.50
Expert	Relevance	DeepSeek	Gemini-2.5-Pro	4.0 [3.0 to 4.0] (95% CI 4.0 to 4.0)	5.0 [3.0 to 5.0] (95% CI 4.0 to 5.0)	0.022	−0.228	−1.00 to −0.00

Perspective	Dimension	Model A	Model B	Score Model A	Score Model B	*P*-adj	Effect Size *r*	Diff 95% CI
Expert	Relevance	DeepSeek	Open-Evidence	4.0 [3.0 to 4.0] (95% CI 4.0 to 4.0)	2.0 [1.0 to 2.0] (95% CI 1.0 to 2.0)	**<.001**	0.798	1.50 to 2.00
Expert	Relevance	DeepSeek	Zhipu Qingyan	4.0 [3.0 to 4.0] (95% CI 4.0 to 4.0)	2.0 [1.0 to 4.0] (95% CI 2.0 to 3.0)	**<.001**	0.557	1.00 to 1.50
Expert	Relevance	Gemini-2.5-Pro	Open-Evidence	5.0 [3.0 to 5.0] (95% CI 4.0 to 5.0)	2.0 [1.0 to 2.0] (95% CI 1.0 to 2.0)	**<.001**	0.771	2.50 to 2.50
Expert	Relevance	Gemini-2.5-Pro	Zhipu Qingyan	5.0 [3.0 to 5.0] (95% CI 4.0 to 5.0)	2.0 [1.0 to 4.0] (95% CI 2.0 to 3.0)	**<.001**	0.681	1.50 to 2.00
Expert	Relevance	Open-Evidence	Zhipu Qingyan	2.0 [1.0 to 2.0] (95% CI 1.0 to 2.0)	2.0 [1.0 to 4.0] (95% CI 2.0 to 3.0)	**<.001**	−0.355	−1.00 to −0.50
Expert	Applicability	ChatGPT-4o	DeepSeek	3.0 [2.0 to 4.0] (95% CI 3.0 to 3.0)	4.0 [3.0 to 4.0] (95% CI 3.0 to 4.0)	0.003	−0.272	−1.00 to −0.00
Expert	Applicability	ChatGPT-4o	Gemini-2.5-Pro	3.0 [2.0 to 4.0] (95% CI 3.0 to 3.0)	5.0 [3.0 to 5.0] (95% CI 4.0 to 5.0)	**<.001**	−0.498	−1.50 to −1.00
Expert	Applicability	ChatGPT-4o	Open-Evidence	3.0 [2.0 to 4.0] (95% CI 3.0 to 3.0)	2.0 [1.0 to 3.0] (95% CI 1.0 to 2.0)	**<.001**	0.582	1.00 to 1.50
Expert	Applicability	ChatGPT-4o	Zhipu Qingyan	3.0 [2.0 to 4.0] (95% CI 3.0 to 3.0)	3.0 [2.0 to 4.0] (95% CI 2.0 to 3.0)	0.203	0.174	0.00 to 0.50
Expert	Applicability	DeepSeek	Gemini-2.5-Pro	4.0 [3.0 to 4.0] (95% CI 3.0 to 4.0)	5.0 [3.0 to 5.0] (95% CI 4.0 to 5.0)	**<.001**	−0.351	−1.00 to −0.50

Perspective	Dimension	Model A	Model B	Score Model A	Score Model B	*P*-adj	Effect Size *r*	Diff 95% CI
Expert	Applicability	DeepSeek	Open-Evidence	4.0 [3.0 to 4.0] (95% CI 3.0 to 4.0)	2.0 [1.0 to 3.0] (95% CI 1.0 to 2.0)	**<.001**	0.642	1.50 to 2.00
Expert	Applicability	DeepSeek	Zhipu Qingyan	4.0 [3.0 to 4.0] (95% CI 3.0 to 4.0)	3.0 [2.0 to 4.0] (95% CI 2.0 to 3.0)	**<.001**	0.418	0.50 to 1.00
Expert	Applicability	Gemini-2.5-Pro	Open-Evidence	5.0 [3.0 to 5.0] (95% CI 4.0 to 5.0)	2.0 [1.0 to 3.0] (95% CI 1.0 to 2.0)	**<.001**	0.793	2.00 to 2.50
Expert	Applicability	Gemini-2.5-Pro	Zhipu Qingyan	5.0 [3.0 to 5.0] (95% CI 4.0 to 5.0)	3.0 [2.0 to 4.0] (95% CI 2.0 to 3.0)	**<.001**	0.642	1.50 to 2.00
Expert	Applicability	Open-Evidence	Zhipu Qingyan	2.0 [1.0 to 3.0] (95% CI 1.0 to 2.0)	3.0 [2.0 to 4.0] (95% CI 2.0 to 3.0)	**<.001**	−0.360	−1.00 to −0.50
Expert	Source Reliability	ChatGPT-4o	DeepSeek	3.0 [2.0 to 4.0] (95% CI 3.0 to 3.0)	3.0 [2.0 to 4.0] (95% CI 3.0 to 4.0)	0.875	−0.127	−0.50 to 0.00
Expert	Source Reliability	ChatGPT-4o	Gemini-2.5-Pro	3.0 [2.0 to 4.0] (95% CI 3.0 to 3.0)	5.0 [3.0 to 5.0] (95% CI 4.0 to 5.0)	**<.001**	−0.529	−1.50 to −1.00
Expert	Source Reliability	ChatGPT-4o	OpenEvidence	3.0 [2.0 to 4.0] (95% CI 3.0 to 3.0)	2.0 [1.0 to 3.0] (95% CI 2.0 to 2.0)	**<.001**	0.515	1.00 to 1.00
Expert	Source Reliability	ChatGPT-4o	Zhipu Qingyan	3.0 [2.0 to 4.0] (95% CI 3.0 to 3.0)	2.0 [1.0 to 3.0] (95% CI 2.0 to 2.0)	**<.001**	0.526	1.00 to 1.50
Expert	Source Reliability	DeepSeek	Gemini-2.5-Pro	3.0 [2.0 to 4.0] (95% CI 3.0 to 4.0)	5.0 [3.0 to 5.0] (95% CI 4.0 to 5.0)	**<.001**	−0.416	−1.00 to −0.50

Perspective	Dimension	Model A	Model B	Score Model A	Score Model B	*P*-adj	Effect Size *r*	Diff 95% CI
Expert	Source Reliability	DeepSeek	Open-Evidence	3.0 [2.0 to 4.0] (95% CI 3.0 to 4.0)	2.0 [1.0 to 3.0] (95% CI 2.0 to 2.0)	**<.001**	0.550	1.00 to 1.50
Expert	Source Reliability	DeepSeek	Zhipu Qingyan	3.0 [2.0 to 4.0] (95% CI 3.0 to 4.0)	2.0 [1.0 to 3.0] (95% CI 2.0 to 2.0)	**<.001**	0.599	1.00 to 1.50
Expert	Source Reliability	Gemini-2.5-Pro	Open-Evidence	5.0 [3.0 to 5.0] (95% CI 4.0 to 5.0)	2.0 [1.0 to 3.0] (95% CI 2.0 to 2.0)	**<.001**	0.767	2.00 to 2.50
Expert	Source Reliability	Gemini-2.5-Pro	Zhipu Qingyan	5.0 [3.0 to 5.0] (95% CI 4.0 to 5.0)	2.0 [1.0 to 3.0] (95% CI 2.0 to 2.0)	**<.001**	0.835	2.00 to 2.50
Expert	Source Reliability	Open-Evidence	Zhipu Qingyan	2.0 [1.0 to 3.0] (95% CI 2.0 to 2.0)	2.0 [1.0 to 3.0] (95% CI 2.0 to 2.0)	>.99	0.018	−0.00 to 0.50
Expert	Comprehensibility	ChatGPT-4o	DeepSeek	3.0 [2.0 to 4.0] (95% CI 3.0 to 3.0)	4.0 [2.0 to 4.0] (95% CI 3.0 to 4.0)	0.100	−0.193	−0.50 to −0.00
Expert	Comprehensibility	ChatGPT-4o	Gemini-2.5-Pro	3.0 [2.0 to 4.0] (95% CI 3.0 to 3.0)	4.0 [3.0 to 5.0] (95% CI 4.0 to 5.0)	**<.001**	−0.411	−1.50 to −0.50
Expert	Comprehensibility	ChatGPT-4o	Open-Evidence	3.0 [2.0 to 4.0] (95% CI 3.0 to 3.0)	1.0 [1.0 to 2.0] (95% CI 1.0 to 2.0)	**<.001**	0.650	1.00 to 1.50
Expert	Comprehensibility	ChatGPT-4o	Zhipu Qingyan	3.0 [2.0 to 4.0] (95% CI 3.0 to 3.0)	3.0 [2.0 to 4.0] (95% CI 3.0 to 3.0)	>.99	0.077	−0.00 to 0.50
Expert	Comprehensibility	DeepSeek	Gemini-2.5-Pro	4.0 [2.0 to 4.0] (95% CI 3.0 to 4.0)	4.0 [3.0 to 5.0] (95% CI 4.0 to 5.0)	0.001	−0.286	−1.00 to −0.00

Perspective	Dimension	Model A	Model B	Score Model A	Score Model B	*P*-adj	Effect Size *r*	Diff 95% CI
Expert	Comprehensibility	DeepSeek	Open-Evidence	4.0 [2.0 to 4.0] (95% CI 3.0 to 4.0)	1.0 [1.0 to 2.0] (95% CI 1.0 to 2.0)	**<.001**	0.693	1.50 to 2.00
Expert	Comprehensibility	DeepSeek	Zhipu Qingyan	4.0 [2.0 to 4.0] (95% CI 3.0 to 4.0)	3.0 [2.0 to 4.0] (95% CI 3.0 to 3.0)	0.003	0.269	0.00 to 1.00
Expert	Comprehensibility	Gemini-2.5-Pro	Open-Evidence	4.0 [3.0 to 5.0] (95% CI 4.0 to 5.0)	1.0 [1.0 to 2.0] (95% CI 1.0 to 2.0)	**<.001**	0.782	2.00 to 2.50
Expert	Comprehensibility	Gemini-2.5-Pro	Zhipu Qingyan	4.0 [3.0 to 5.0] (95% CI 4.0 to 5.0)	3.0 [2.0 to 4.0] (95% CI 3.0 to 3.0)	**<.001**	0.518	1.00 to 1.50
Expert	Comprehensibility	Open-Evidence	Zhipu Qingyan	1.0 [1.0 to 2.0] (95% CI 1.0 to 2.0)	3.0 [2.0 to 4.0] (95% CI 3.0 to 3.0)	**<.001**	−0.500	−1.50 to −1.00
Expert	Actionability	ChatGPT-4o	DeepSeek	3.0 [2.0 to 4.0] (95% CI 3.0 to 3.0)	4.0 [3.0 to 4.0] (95% CI 3.0 to 4.0)	0.008	−0.251	−1.00 to −0.00
Expert	Actionability	ChatGPT-4o	Gemini-2.5-Pro	3.0 [2.0 to 4.0] (95% CI 3.0 to 3.0)	4.0 [3.0 to 5.0] (95% CI 4.0 to 5.0)	**<.001**	−0.468	−1.50 to −0.50
Expert	Actionability	ChatGPT-4o	Open-Evidence	3.0 [2.0 to 4.0] (95% CI 3.0 to 3.0)	2.0 [1.0 to 2.0] (95% CI 1.0 to 2.0)	**<.001**	0.609	1.00 to 1.50
Expert	Actionability	ChatGPT-4o	Zhipu Qingyan	3.0 [2.0 to 4.0] (95% CI 3.0 to 3.0)	3.0 [2.0 to 4.0] (95% CI 3.0 to 3.0)	>.99	0.017	−0.00 to 0.50
Expert	Actionability	DeepSeek	Gemini-2.5-Pro	4.0 [3.0 to 4.0] (95% CI 3.0 to 4.0)	4.0 [3.0 to 5.0] (95% CI 4.0 to 5.0)	**<.001**	−0.307	−1.00 to −0.00

Perspective	Dimension	Model A	Model B	Score Model A	Score Model B	*P*-adj	Effect Size *r*	Diff 95% CI
Expert	Actionability	DeepSeek	Open-Evidence	4.0 [3.0 to 4.0] (95% CI 3.0 to 4.0)	2.0 [1.0 to 2.0] (95% CI 1.0 to 2.0)	**<.001**	0.695	1.50 to 2.00
Expert	Actionability	DeepSeek	Zhipu Qingyan	4.0 [3.0 to 4.0] (95% CI 3.0 to 4.0)	3.0 [2.0 to 4.0] (95% CI 3.0 to 3.0)	**<.001**	0.300	0.00 to 1.00
Expert	Actionability	Gemini-2.5-Pro	Open-Evidence	4.0 [3.0 to 5.0] (95% CI 4.0 to 5.0)	2.0 [1.0 to 2.0] (95% CI 1.0 to 2.0)	**<.001**	0.785	2.00 to 2.50
Expert	Actionability	Gemini-2.5-Pro	Zhipu Qingyan	4.0 [3.0 to 5.0] (95% CI 4.0 to 5.0)	3.0 [2.0 to 4.0] (95% CI 3.0 to 3.0)	**<.001**	0.492	1.00 to 1.50
Expert	Actionability	Open-Evidence	Zhipu Qingyan	2.0 [1.0 to 2.0] (95% CI 1.0 to 2.0)	3.0 [2.0 to 4.0] (95% CI 3.0 to 3.0)	**<.001**	−0.523	−1.50 to −1.00

Significance shown in bold (*P*<.001). *r* = paired matched-pairs rank-biserial correlation. Diff 95% CI = Hodges–Lehmann estimate of the median location shift with 95% confidence interval.
