# Supplementary material for: Performance of 5 Large Language Models in Perioperative Consultation for Pediatric Hypospadias: Cross-Sectional Comparative Study
Source: J Med Internet Res. 2026 Jul 29;28:e93393. doi: 10.2196/93393 (PMC13419283; doi:10.2196/93393)
Supplement: Multimedia Appendix 8 [file jmir-v28-e93393-s008.pdf]

Theme-based analysis

Group A — Expert evaluation of Risk and Prognosis questions (Q1, Q2, Q3, Q5, Q7); Group B — Caregiver evaluation of Home-Care Guidance questions (Q4, Q6, Q8, Q10); Group C — Expert evaluation of the Emergency Response question (Q9).

Part A. Theme-level descriptive statistics (15 rows = 3 themes × 5 models).

Theme	Model	n	Median	Q1	Q3	CI_lower	CI_upper	Stat_Label
A. Expert: Risk & Prognosis (Q1,2,3,5,7)	ChatGPT-4o	805	3	2	4	3	3	3.0 [2.0-4.0] (95% CI 3.0-3.0)
A. Expert: Risk & Prognosis (Q1,2,3,5,7)	DeepSeek	805	4	3	4	4	4	4.0 [3.0-4.0] (95% CI 4.0-4.0)
A. Expert: Risk & Prognosis (Q1,2,3,5,7)	Gemini-2.5-Pro	805	5	3	5	4	5	5.0 [3.0-5.0] (95% CI 4.0-5.0)
A. Expert: Risk & Prognosis (Q1,2,3,5,7)	OpenEvidence	805	2	1	3	2	2	2.0 [1.0-3.0] (95% CI 2.0-2.0)
A. Expert: Risk & Prognosis (Q1,2,3,5,7)	Zhipu Qingyan	805	2	1	3	2	2	2.0 [1.0-3.0] (95% CI 2.0-2.0)
B. Caregiver: Home Care Guidance (Q4,6,8,10)	ChatGPT-4o	720	3	2	4	2	3	3.0 [2.0-4.0] (95% CI 2.0-3.0)
B. Caregiver: Home Care Guidance (Q4,6,8,10)	DeepSeek	720	4	3	4	3	4	4.0 [3.0-4.0] (95% CI 3.0-4.0)
B. Caregiver: Home Care Guidance (Q4,6,8,10)	Gemini-2.5-Pro	720	5	3	5	4	5	5.0 [3.0-5.0] (95% CI 4.0-5.0)
B. Caregiver: Home Care Guidance (Q4,6,8,10)	OpenEvidence	720	1	1	2	1	1	1.0 [1.0-2.0] (95% CI 1.0-1.0)
B. Caregiver:	Zhipu Qingyan	720	3	2	4	3	3	3.0 [2.0-4.0]

Theme	Model	n	Median	Q1	Q3	CI_lower	CI_upper	Stat_Label
Home Care Guidance (Q4,6,8,10)								(95% CI 3.0-3.0)
C. Expert: Emergency Response (Q9)	ChatGPT-4o	161	3	2	4	3	3	3.0 [2.0-4.0] (95% CI 3.0-3.0)
C. Expert: Emergency Response (Q9)	DeepSeek	161	4	2	4	3	4	4.0 [2.0-4.0] (95% CI 3.0-4.0)
C. Expert: Emergency Response (Q9)	Gemini-2.5-Pro	161	4	3	5	4	5	4.0 [3.0-5.0] (95% CI 4.0-5.0)
C. Expert: Emergency Response (Q9)	OpenEvidence	161	1	1	2	1	1	1.0 [1.0-2.0] (95% CI 1.0-1.0)
C. Expert: Emergency Response (Q9)	Zhipu Qingyan	161	3	2	4	3	4	3.0 [2.0-4.0] (95% CI 3.0-4.0)

Part B. Theme-level pairwise comparisons (Bonferroni-adjusted; 30 rows = 3 themes × 10 pairs).

Theme	Model A	Model B	Score Model A	Score Model B	*P*-adj	Effect Size *r*	Diff 95% CI
A. Expert: Risk & Prognosis (Q1,2,3,5,7)	ChatGPT-4o	DeepSeek	3.0 [2.0 to 4.0] (95% CI 3.0 to 3.0)	4.0 [3.0 to 4.0] (95% CI 4.0 to 4.0)	**<.001**	−0.288	−1.00 to −0.50
A. Expert: Risk & Prognosis (Q1,2,3,5,7)	ChatGPT-4o	Gemini-2.5-Pro	3.0 [2.0 to 4.0] (95% CI 3.0 to 3.0)	5.0 [3.0 to 5.0] (95% CI 4.0 to 5.0)	**<.001**	−0.519	−1.50 to −1.00
A. Expert: Risk & Prognosis (Q1,2,3,5,7)	ChatGPT-4o	OpenEvidence	3.0 [2.0 to 4.0] (95% CI 3.0 to 3.0)	2.0 [1.0 to 3.0] (95% CI 2.0 to 2.0)	**<.001**	0.399	0.50 to 1.00
A. Expert: Risk & Prognosis (Q1,2,3,5,7)	ChatGPT-4o	Zhipu Qingyan	3.0 [2.0 to 4.0] (95% CI 3.0 to 3.0)	2.0 [1.0 to 3.0] (95% CI 2.0 to 2.0)	**<.001**	0.337	0.50 to 1.00
A. Expert: Risk	DeepSeek	Gemini-2.5-Pro	4.0 [3.0 to	5.0 [3.0 to	**<.001**	−0.332	−1.00 to

Theme	Model A	Model B	Score Model A	Score Model B	*P*-adj	Effect Size *r*	Diff 95% CI
& Prognosis (Q1,2,3,5,7)			4.0] (95% CI 4.0 to 4.0)	5.0] (95% CI 4.0 to 5.0)			−0.50
A. Expert: Risk & Prognosis (Q1,2,3,5,7)	DeepSeek	OpenEvidence	4.0 [3.0 to 4.0] (95% CI 4.0 to 4.0)	2.0 [1.0 to 3.0] (95% CI 2.0 to 2.0)	**<.001**	0.581	1.00 to 1.50
A. Expert: Risk & Prognosis (Q1,2,3,5,7)	DeepSeek	Zhipu Qingyan	4.0 [3.0 to 4.0] (95% CI 4.0 to 4.0)	2.0 [1.0 to 3.0] (95% CI 2.0 to 2.0)	**<.001**	0.599	1.00 to 1.50
A. Expert: Risk & Prognosis (Q1,2,3,5,7)	Gemini-2.5-Pro	OpenEvidence	5.0 [3.0 to 5.0] (95% CI 4.0 to 5.0)	2.0 [1.0 to 3.0] (95% CI 2.0 to 2.0)	**<.001**	0.685	2.00 to 2.50
A. Expert: Risk & Prognosis (Q1,2,3,5,7)	Gemini-2.5-Pro	Zhipu Qingyan	5.0 [3.0 to 5.0] (95% CI 4.0 to 5.0)	2.0 [1.0 to 3.0] (95% CI 2.0 to 2.0)	**<.001**	0.775	1.50 to 2.00
A. Expert: Risk & Prognosis (Q1,2,3,5,7)	OpenEvidence	Zhipu Qingyan	2.0 [1.0 to 3.0] (95% CI 2.0 to 2.0)	2.0 [1.0 to 3.0] (95% CI 2.0 to 2.0)	>.99	0.008	−0.00 to 0.00
B. Caregiver: Home Care Guidance (Q4,6,8,10)	ChatGPT-4o	DeepSeek	3.0 [2.0 to 4.0] (95% CI 2.0 to 3.0)	4.0 [3.0 to 4.0] (95% CI 3.0 to 4.0)	**<.001**	−0.327	−1.00 to −0.50
B. Caregiver: Home Care Guidance (Q4,6,8,10)	ChatGPT-4o	Gemini-2.5-Pro	3.0 [2.0 to 4.0] (95% CI 2.0 to 3.0)	5.0 [3.0 to 5.0] (95% CI 4.0 to 5.0)	**<.001**	−0.571	−1.50 to −1.00
B. Caregiver: Home Care Guidance (Q4,6,8,10)	ChatGPT-4o	OpenEvidence	3.0 [2.0 to 4.0] (95% CI 2.0 to 3.0)	1.0 [1.0 to 2.0] (95% CI 1.0 to 1.0)	**<.001**	0.498	1.00 to 1.50
B. Caregiver: Home Care Guidance (Q4,6,8,10)	ChatGPT-4o	Zhipu Qingyan	3.0 [2.0 to 4.0] (95% CI 2.0 to 3.0)	3.0 [2.0 to 4.0] (95% CI 3.0 to 3.0)	0.072	−0.113	−0.50 to −0.00
B. Caregiver:	DeepSeek	Gemini-2.5-Pro	4.0 [3.0 to	5.0 [3.0 to	**<.001**	−0.333	−1.00 to

Theme	Model A	Model B	Score Model A	Score Model B	*P*-adj	Effect Size *r*	Diff 95% CI
Home Care Guidance (Q4,6,8,10)			4.0] (95% CI 3.0 to 4.0)	5.0] (95% CI 4.0 to 5.0)			−0.50
B. Caregiver: Home Care Guidance (Q4,6,8,10)	DeepSeek	OpenEvidence	4.0 [3.0 to 4.0] (95% CI 3.0 to 4.0)	1.0 [1.0 to 2.0] (95% CI 1.0 to 1.0)	**<.001**	0.670	1.50 to 2.00
B. Caregiver: Home Care Guidance (Q4,6,8,10)	DeepSeek	Zhipu Qingyan	4.0 [3.0 to 4.0] (95% CI 3.0 to 4.0)	3.0 [2.0 to 4.0] (95% CI 3.0 to 3.0)	**<.001**	0.231	0.00 to 0.50
B. Caregiver: Home Care Guidance (Q4,6,8,10)	Gemini-2.5-Pro	OpenEvidence	5.0 [3.0 to 5.0] (95% CI 4.0 to 5.0)	1.0 [1.0 to 2.0] (95% CI 1.0 to 1.0)	**<.001**	0.747	2.50 to 2.50
B. Caregiver: Home Care Guidance (Q4,6,8,10)	Gemini-2.5-Pro	Zhipu Qingyan	5.0 [3.0 to 5.0] (95% CI 4.0 to 5.0)	3.0 [2.0 to 4.0] (95% CI 3.0 to 3.0)	**<.001**	0.479	1.00 to 1.00
B. Caregiver: Home Care Guidance (Q4,6,8,10)	OpenEvidence	Zhipu Qingyan	1.0 [1.0 to 2.0] (95% CI 1.0 to 1.0)	3.0 [2.0 to 4.0] (95% CI 3.0 to 3.0)	**<.001**	−0.616	−1.50 to −1.00
C. Expert: Emergency Response (Q9)	ChatGPT-4o	DeepSeek	3.0 [2.0 to 4.0] (95% CI 3.0 to 3.0)	4.0 [2.0 to 4.0] (95% CI 3.0 to 4.0)	0.029	−0.263	−1.00 to −0.00
C. Expert: Emergency Response (Q9)	ChatGPT-4o	Gemini-2.5-Pro	3.0 [2.0 to 4.0] (95% CI 3.0 to 3.0)	4.0 [3.0 to 5.0] (95% CI 4.0 to 5.0)	**<.001**	−0.561	−1.50 to −1.00
C. Expert: Emergency Response (Q9)	ChatGPT-4o	OpenEvidence	3.0 [2.0 to 4.0] (95% CI 3.0 to 3.0)	1.0 [1.0 to 2.0] (95% CI 1.0 to 1.0)	**<.001**	0.641	1.00 to 1.50
C. Expert: Emergency Response (Q9)	ChatGPT-4o	Zhipu Qingyan	3.0 [2.0 to 4.0] (95% CI 3.0 to 3.0)	3.0 [2.0 to 4.0] (95% CI 3.0 to 4.0)	0.041	−0.258	−0.50 to −0.00
C. Expert:	DeepSeek	Gemini-2.5-Pro	4.0 [2.0 to	4.0 [3.0 to	**<.001**	−0.361	−1.00 to

Theme	Model A	Model B	Score Model A	Score Model B	*P*-adj	Effect Size *r*	Diff 95% CI
Emergency Response (Q9)			4.0] (95% CI 3.0 to 4.0)	5.0] (95% CI 4.0 to 5.0)			−0.50
C. Expert: Emergency Response (Q9)	DeepSeek	OpenEvidence	4.0 [2.0 to 4.0] (95% CI 3.0 to 4.0)	1.0 [1.0 to 2.0] (95% CI 1.0 to 1.0)	**<.001**	0.756	1.50 to 2.00
C. Expert: Emergency Response (Q9)	DeepSeek	Zhipu Qingyan	4.0 [2.0 to 4.0] (95% CI 3.0 to 4.0)	3.0 [2.0 to 4.0] (95% CI 3.0 to 4.0)	>.99	0.023	−0.50 to 0.50
C. Expert: Emergency Response (Q9)	Gemini-2.5-Pro	OpenEvidence	4.0 [3.0 to 5.0] (95% CI 4.0 to 5.0)	1.0 [1.0 to 2.0] (95% CI 1.0 to 1.0)	**<.001**	0.858	2.00 to 3.00
C. Expert: Emergency Response (Q9)	Gemini-2.5-Pro	Zhipu Qingyan	4.0 [3.0 to 5.0] (95% CI 4.0 to 5.0)	3.0 [2.0 to 4.0] (95% CI 3.0 to 4.0)	0.002	0.339	0.50 to 1.00
C. Expert: Emergency Response (Q9)	OpenEvidence	Zhipu Qingyan	1.0 [1.0 to 2.0] (95% CI 1.0 to 1.0)	3.0 [2.0 to 4.0] (95% CI 3.0 to 4.0)	**<.001**	−0.757	−2.00 to −1.50

Part C. Theme-level stacked bar chart. A three-panel 100% stacked bar chart of score distributions for the five LLMs, faceted by theme. Figure caption: 100% stacked bar chart of score distributions for the five LLMs, faceted by theme (A: expert-evaluated risk and prognosis; B: caregiver-evaluated home-care guidance; C: expert-evaluated emergency response). Each colour band represents the proportion of a given score (1–5) within total evaluations per panel.

Across all three themes, the relative ranking pattern remained consistent: Gemini-2.5-Pro achieved the highest proportion of top scores, followed by DeepSeek. ChatGPT-4o and Zhipu Qingyan demonstrated intermediate performance, while OpenEvidence consistently recorded the lowest scores under this specific evaluation metric.

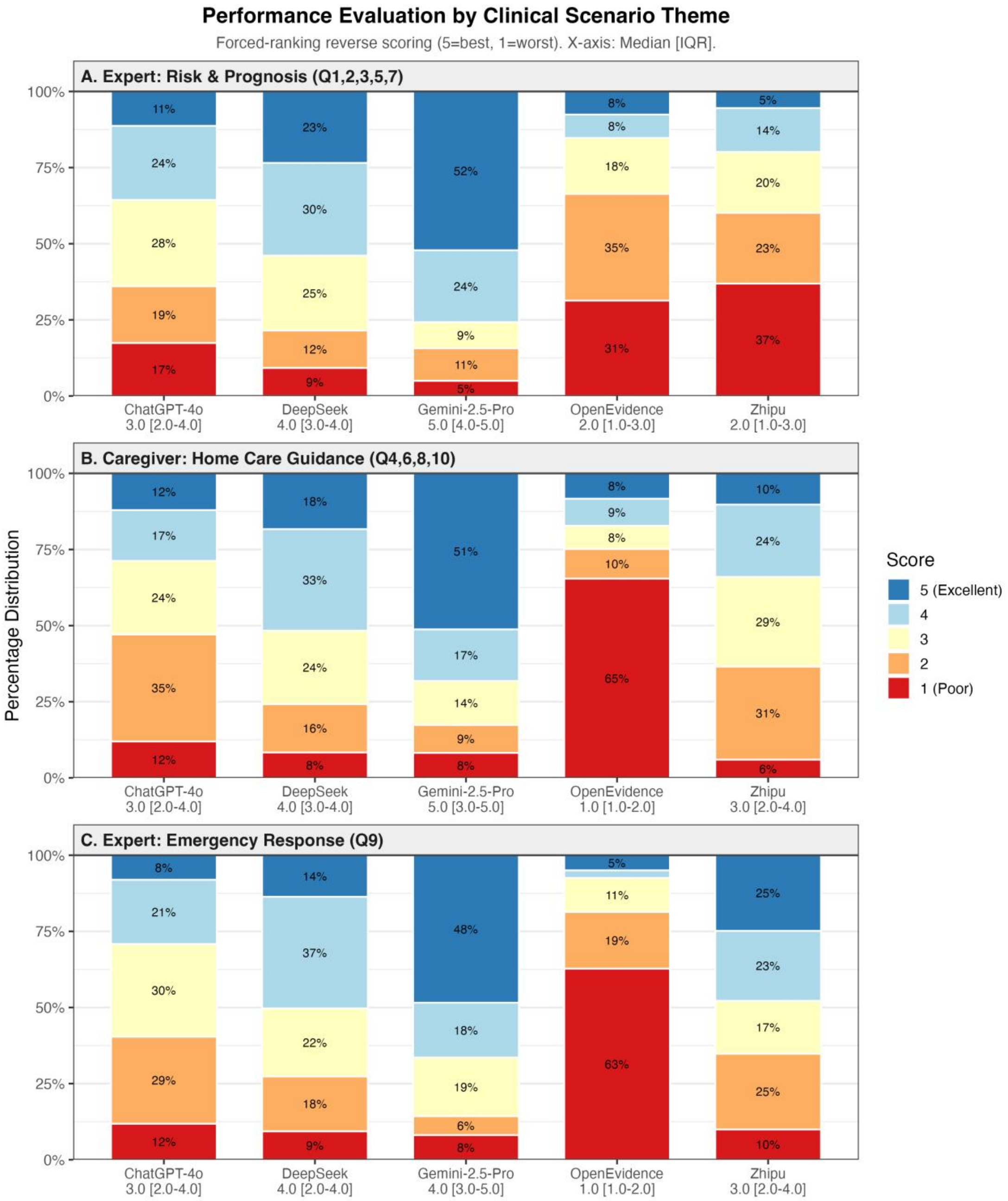

Performance Evaluation by Clinical Scenario Theme
Forced-ranking reverse scoring (5=best, 1=worst). X-axis: Median [IQR].
Percentage Distribution
100%
75%
50%
25%
0%
A. Expert: Risk & Prognosis (Q1,2,3,5,7)
11%
24%
28%
19%
17%
23%
30%
25%
12%
9%
52%
24%
9%
11%
5%
8%
8%
18%
35%
31%
5%
14%
20%
23%
37%
ChatGPT-4o 3.0 [2.0-4.0]
DeepSeek 4.0 [3.0-4.0]
Gemini-2.5-Pro 5.0 [4.0-5.0]
OpenEvidence 2.0 [1.0-3.0]
Zhipu 2.0 [1.0-3.0]
B. Caregiver: Home Care Guidance (Q4,6,8,10)
12%
17%
24%
35%
12%
18%
33%
24%
16%
8%
51%
17%
14%
9%
8%
8%
9%
8%
10%
65%
10%
24%
29%
31%
6%
ChatGPT-4o 3.0 [2.0-4.0]
DeepSeek 4.0 [3.0-4.0]
Gemini-2.5-Pro 5.0 [3.0-5.0]
OpenEvidence 1.0 [1.0-2.0]
Zhipu 3.0 [2.0-4.0]
C. Expert: Emergency Response (Q9)
8%
21%
30%
29%
12%
14%
37%
22%
18%
9%
48%
18%
19%
6%
8%
5%
11%
19%
63%
25%
23%
17%
25%
10%
ChatGPT-4o 3.0 [2.0-4.0]
DeepSeek 4.0 [2.0-4.0]
Gemini-2.5-Pro 4.0 [3.0-5.0]
OpenEvidence 1.0 [1.0-2.0]
Zhipu 3.0 [2.0-4.0]
Score
5 (Excellent)
4
3
2
1 (Poor)
