# Supplementary material for: Performance of 5 Large Language Models in Perioperative Consultation for Pediatric Hypospadias: Cross-Sectional Comparative Study
Source: J Med Internet Res. 2026 Jul 29;28:e93393. doi: 10.2196/93393 (PMC13419283; doi:10.2196/93393)

Risk-level stratified analysis

Part A. Descriptive statistics (30 rows = 3 risk levels × 5 models × 2 perspectives).

Perspective	Risk_Group	Model	n	Median	Q1	Q3	CI_lower	CI_upper
Caregiver	High Risk (Q5, Q6, Q9)	ChatGPT-4o	540	2	2	3	2	2
Caregiver	High Risk (Q5, Q6, Q9)	DeepSeek	540	4	3	4	4	4
Caregiver	High Risk (Q5, Q6, Q9)	Gemini-2.5-Pro	540	5	3	5	4	5
Caregiver	High Risk (Q5, Q6, Q9)	OpenEvidence	540	1	1	2	1	1
Caregiver	High Risk (Q5, Q6, Q9)	Zhipu Qingyan	540	3	2	4	3	3
Caregiver	Medium Risk (Q2, Q7, Q8)	ChatGPT-4o	540	2	2	3	2	3
Caregiver	Medium Risk (Q2, Q7, Q8)	DeepSeek	540	4	3	4	3	4
Caregiver	Medium Risk (Q2, Q7, Q8)	Gemini-2.5-Pro	540	4	3	5	4	5
Caregiver	Medium Risk (Q2, Q7, Q8)	OpenEvidence	540	2	1	2	1	2
Caregiver	Medium Risk (Q2, Q7, Q8)	Zhipu Qingyan	540	3	2	4	3	3
Caregiver	Low Risk (Q1, Q3, Q4, Q10)	ChatGPT-4o	720	3	2	4	2	3
Caregiver	Low Risk (Q1, Q3, Q4, Q10)	DeepSeek	720	3	2	4	3	4
Caregiver	Low Risk (Q1, Q3, Q4, Q10)	Gemini-2.5-Pro	720	5	3	5	4	5
Caregiver	Low Risk (Q1, Q3, Q4, Q10)	OpenEvidence	720	2	1	3	1	2
Caregiver	Low Risk (Q1, Q3, Q4, Q10)	Zhipu Qingyan	720	3	2	4	3	3
Expert	High Risk (Q5, Q6, Q9)	ChatGPT-4o	483	3	2	4	3	3
Expert	High Risk	DeepSeek	483	3	2.5	4	3	3

Perspective	Risk_Group	Model	n	Median	Q1	Q3	CI_lower	CI_upper
	(Q5, Q6, Q9)							
Expert	High Risk (Q5, Q6, Q9)	Gemini-2.5-Pro	483	5	4	5	5	5
Expert	High Risk (Q5, Q6, Q9)	OpenEvidence	483	1	1	2	1	2
Expert	High Risk (Q5, Q6, Q9)	Zhipu Qingyan	483	3	2	4	3	3
Expert	Medium Risk (Q2, Q7, Q8)	ChatGPT-4o	483	3	2	4	3	3
Expert	Medium Risk (Q2, Q7, Q8)	DeepSeek	483	4	3	4	3	4
Expert	Medium Risk (Q2, Q7, Q8)	Gemini-2.5-Pro	483	5	3	5	4	5
Expert	Medium Risk (Q2, Q7, Q8)	OpenEvidence	483	2	1	3	2	2
Expert	Medium Risk (Q2, Q7, Q8)	Zhipu Qingyan	483	2	1	3	2	2
Expert	Low Risk (Q1, Q3, Q4, Q10)	ChatGPT-4o	644	3	2	4	3	3
Expert	Low Risk (Q1, Q3, Q4, Q10)	DeepSeek	644	4	3	5	4	4
Expert	Low Risk (Q1, Q3, Q4, Q10)	Gemini-2.5-Pro	644	5	3	5	4	5
Expert	Low Risk (Q1, Q3, Q4, Q10)	OpenEvidence	644	2	1	2	1	2
Expert	Low Risk (Q1, Q3, Q4, Q10)	Zhipu Qingyan	644	3	2	3	2	3

Part B. Pairwise comparisons (Bonferroni-adjusted; 60 rows = 3 risk levels × 10 pairs × 2 perspectives).

Perspective	Risk_Group	Model A	Model B	Score Model A	Score Model B	*P*-adj	Effect Size *r*	Diff 95% CI
Expert	High Risk (Q5, Q6, Q9)	ChatGPT-4o	DeepSeek	3.0 [2.0 to 4.0] (95% CI 3.0 to	3.0 [2.5 to 4.0] (95% CI 3.0 to	<.001	−0.236 (small)	−1.00 to −0.00

Perspective	Risk_Group	Model A	Model B	Score Model A	Score Model B	*P*-adj	Effect Size *r*	Diff 95% CI
				3.0)	3.0)			
Expert	High Risk (Q5, Q6, Q9)	ChatGPT-4o	Gemini-2.5-Pro	3.0 [2.0 to 4.0] (95% CI 3.0 to 3.0)	5.0 [4.0 to 5.0] (95% CI 5.0 to 5.0)	<.001	−0.626 (large)	−1.50 to −1.00
Expert	High Risk (Q5, Q6, Q9)	ChatGPT-4o	OpenEvidence	3.0 [2.0 to 4.0] (95% CI 3.0 to 3.0)	1.0 [1.0 to 2.0] (95% CI 1.0 to 2.0)	<.001	0.633 (large)	1.00 to 1.50
Expert	High Risk (Q5, Q6, Q9)	ChatGPT-4o	Zhipu Qingyan	3.0 [2.0 to 4.0] (95% CI 3.0 to 3.0)	3.0 [2.0 to 4.0] (95% CI 3.0 to 3.0)	>.99	−0.009 (negligible)	−0.00 to 0.00
Expert	High Risk (Q5, Q6, Q9)	DeepSeek	Gemini-2.5-Pro	3.0 [2.5 to 4.0] (95% CI 3.0 to 3.0)	5.0 [4.0 to 5.0] (95% CI 5.0 to 5.0)	<.001	−0.491 (moderate)	−1.50 to −1.00
Expert	High Risk (Q5, Q6, Q9)	DeepSeek	OpenEvidence	3.0 [2.5 to 4.0] (95% CI 3.0 to 3.0)	1.0 [1.0 to 2.0] (95% CI 1.0 to 2.0)	<.001	0.702 (large)	1.50 to 2.00
Expert	High Risk (Q5, Q6, Q9)	DeepSeek	Zhipu Qingyan	3.0 [2.5 to 4.0] (95% CI 3.0 to 3.0)	3.0 [2.0 to 4.0] (95% CI 3.0 to 3.0)	<.001	0.222 (small)	0.00 to 0.50
Expert	High Risk (Q5, Q6, Q9)	Gemini-2.5-Pro	OpenEvidence	5.0 [4.0 to 5.0] (95% CI 5.0 to 5.0)	1.0 [1.0 to 2.0] (95% CI 1.0 to 2.0)	<.001	0.875 (large)	2.50 to 3.00
Expert	High Risk (Q5, Q6, Q9)	Gemini-2.5-Pro	Zhipu Qingyan	5.0 [4.0 to 5.0] (95% CI 5.0 to 5.0)	3.0 [2.0 to 4.0] (95% CI 3.0 to 3.0)	<.001	0.659 (large)	1.50 to 1.50

Perspective	Risk_Group	Model A	Model B	Score Model A	Score Model B	*P*-adj	Effect Size *r*	Diff 95% CI
Expert	High Risk (Q5, Q6, Q9)	OpenEvidence	Zhipu Qingyan	1.0 [1.0 to 2.0] (95% CI 1.0 to 2.0)	3.0 [2.0 to 4.0] (95% CI 3.0 to 3.0)	<.001	−0.548 (large)	−1.50 to −1.00
Expert	Medium Risk (Q2, Q7, Q8)	ChatGPT-4o	DeepSeek	3.0 [2.0 to 4.0] (95% CI 3.0 to 3.0)	4.0 [3.0 to 4.0] (95% CI 3.0 to 4.0)	<.001	−0.240 (small)	−0.50 to −0.00
Expert	Medium Risk (Q2, Q7, Q8)	ChatGPT-4o	Gemini-2.5-Pro	3.0 [2.0 to 4.0] (95% CI 3.0 to 3.0)	5.0 [3.0 to 5.0] (95% CI 4.0 to 5.0)	<.001	−0.451 (moderate)	−1.50 to −1.00
Expert	Medium Risk (Q2, Q7, Q8)	ChatGPT-4o	OpenEvidence	3.0 [2.0 to 4.0] (95% CI 3.0 to 3.0)	2.0 [1.0 to 3.0] (95% CI 2.0 to 2.0)	<.001	0.515 (large)	1.00 to 1.00
Expert	Medium Risk (Q2, Q7, Q8)	ChatGPT-4o	Zhipu Qingyan	3.0 [2.0 to 4.0] (95% CI 3.0 to 3.0)	2.0 [1.0 to 3.0] (95% CI 2.0 to 2.0)	<.001	0.318 (moderate)	0.50 to 1.00
Expert	Medium Risk (Q2, Q7, Q8)	DeepSeek	Gemini-2.5-Pro	4.0 [3.0 to 4.0] (95% CI 3.0 to 4.0)	5.0 [3.0 to 5.0] (95% CI 4.0 to 5.0)	<.001	−0.312 (moderate)	−1.00 to −0.50
Expert	Medium Risk (Q2, Q7, Q8)	DeepSeek	OpenEvidence	4.0 [3.0 to 4.0] (95% CI 3.0 to 4.0)	2.0 [1.0 to 3.0] (95% CI 2.0 to 2.0)	<.001	0.617 (large)	1.00 to 1.50
Expert	Medium Risk (Q2, Q7, Q8)	DeepSeek	Zhipu Qingyan	4.0 [3.0 to 4.0] (95% CI 3.0 to 4.0)	2.0 [1.0 to 3.0] (95% CI 2.0 to 2.0)	<.001	0.526 (large)	1.00 to 1.50
Expert	Medium Risk	Gemini-2.5-Pro	OpenEvidence	5.0 [3.0	2.0 [1.0	<.001	0.702	2.00 to

Perspective	Risk_Group	Model A	Model B	Score Model A	Score Model B	P-adj	Effect Size r	Diff 95% CI
	(Q2, Q7, Q8)			to 5.0] (95% CI 4.0 to 5.0)	to 3.0] (95% CI 2.0 to 2.0)		(large)	2.50
Expert	Medium Risk (Q2, Q7, Q8)	Gemini-2.5-Pro	Zhipu Qingyan	5.0 [3.0 to 5.0] (95% CI 4.0 to 5.0)	2.0 [1.0 to 3.0] (95% CI 2.0 to 2.0)	<.001	0.694 (large)	1.50 to 2.00
Expert	Medium Risk (Q2, Q7, Q8)	OpenEvidence	Zhipu Qingyan	2.0 [1.0 to 3.0] (95% CI 2.0 to 2.0)	2.0 [1.0 to 3.0] (95% CI 2.0 to 2.0)	.09	−0.136 (small)	−0.50 to −0.00
Expert	Low Risk (Q1, Q3, Q4, Q10)	ChatGPT-4o	DeepSeek	3.0 [2.0 to 4.0] (95% CI 3.0 to 3.0)	4.0 [3.0 to 5.0] (95% CI 4.0 to 4.0)	<.001	−0.479 (moderate)	−1.00 to −1.00
Expert	Low Risk (Q1, Q3, Q4, Q10)	ChatGPT-4o	Gemini-2.5-Pro	3.0 [2.0 to 4.0] (95% CI 3.0 to 3.0)	5.0 [3.0 to 5.0] (95% CI 4.0 to 5.0)	<.001	−0.588 (large)	−1.50 to −1.00
Expert	Low Risk (Q1, Q3, Q4, Q10)	ChatGPT-4o	OpenEvidence	3.0 [2.0 to 4.0] (95% CI 3.0 to 3.0)	2.0 [1.0 to 2.0] (95% CI 1.0 to 2.0)	<.001	0.580 (large)	1.00 to 1.00
Expert	Low Risk (Q1, Q3, Q4, Q10)	ChatGPT-4o	Zhipu Qingyan	3.0 [2.0 to 4.0] (95% CI 3.0 to 3.0)	3.0 [2.0 to 3.0] (95% CI 2.0 to 3.0)	<.001	0.195 (small)	0.00 to 0.50
Expert	Low Risk (Q1, Q3, Q4, Q10)	DeepSeek	Gemini-2.5-Pro	4.0 [3.0 to 5.0] (95% CI 4.0 to 4.0)	5.0 [3.0 to 5.0] (95% CI 4.0 to 5.0)	<.001	−0.201 (small)	−0.50 to −0.00
Expert	Low Risk (Q1, Q3, Q4,	DeepSeek	OpenEvidence	4.0 [3.0 to 5.0]	2.0 [1.0 to 2.0]	<.001	0.802 (large)	2.00 to 2.50

Perspective	Risk_Group	Model A	Model B	Score Model A	Score Model B	*P*-adj	Effect Size *r*	Diff 95% CI
	Q10)			(95% CI 4.0 to 4.0)	(95% CI 1.0 to 2.0)			
Expert	Low Risk (Q1, Q3, Q4, Q10)	DeepSeek	Zhipu Qingyan	4.0 [3.0 to 5.0] (95% CI 4.0 to 4.0)	3.0 [2.0 to 3.0] (95% CI 2.0 to 3.0)	<.001	0.637 (large)	1.00 to 1.50
Expert	Low Risk (Q1, Q3, Q4, Q10)	Gemini-2.5-Pro	OpenEvidence	5.0 [3.0 to 5.0] (95% CI 4.0 to 5.0)	2.0 [1.0 to 2.0] (95% CI 1.0 to 2.0)	<.001	0.844 (large)	2.50 to 2.50
Expert	Low Risk (Q1, Q3, Q4, Q10)	Gemini-2.5-Pro	Zhipu Qingyan	5.0 [3.0 to 5.0] (95% CI 4.0 to 5.0)	3.0 [2.0 to 3.0] (95% CI 2.0 to 3.0)	<.001	0.719 (large)	1.50 to 2.00
Expert	Low Risk (Q1, Q3, Q4, Q10)	OpenEvidence	Zhipu Qingyan	2.0 [1.0 to 2.0] (95% CI 1.0 to 2.0)	3.0 [2.0 to 3.0] (95% CI 2.0 to 3.0)	<.001	−0.412 (moderate)	−1.00 to −0.50
Caregiver	High Risk (Q5, Q6, Q9)	ChatGPT-4o	DeepSeek	2.0 [2.0 to 3.0] (95% CI 2.0 to 2.0)	4.0 [3.0 to 4.0] (95% CI 4.0 to 4.0)	<.001	−0.648 (large)	−1.50 to −1.50
Caregiver	High Risk (Q5, Q6, Q9)	ChatGPT-4o	Gemini-2.5-Pro	2.0 [2.0 to 3.0] (95% CI 2.0 to 2.0)	5.0 [3.0 to 5.0] (95% CI 4.0 to 5.0)	<.001	−0.639 (large)	−2.00 to −1.50
Caregiver	High Risk (Q5, Q6, Q9)	ChatGPT-4o	OpenEvidence	2.0 [2.0 to 3.0] (95% CI 2.0 to 2.0)	1.0 [1.0 to 2.0] (95% CI 1.0 to 1.0)	<.001	0.372 (moderate)	0.50 to 1.00
Caregiver	High Risk (Q5, Q6, Q9)	ChatGPT-4o	Zhipu Qingyan	2.0 [2.0 to 3.0] (95% CI	3.0 [2.0 to 4.0] (95% CI	<.001	−0.387 (moderate)	−1.00 to −0.50

Perspective	Risk_Group	Model A	Model B	Score Model A	Score Model B	*P*-adj	Effect Size *r*	Diff 95% CI
				2.0 to 2.0)	3.0 to 3.0)			
Caregiver	High Risk (Q5, Q6, Q9)	DeepSeek	Gemini-2.5-Pro	4.0 [3.0 to 4.0] (95% CI 4.0 to 4.0)	5.0 [3.0 to 5.0] (95% CI 4.0 to 5.0)	.04	−0.140 (small)	−0.50 to −0.00
Caregiver	High Risk (Q5, Q6, Q9)	DeepSeek	OpenEvidence	4.0 [3.0 to 4.0] (95% CI 4.0 to 4.0)	1.0 [1.0 to 2.0] (95% CI 1.0 to 1.0)	<.001	0.778 (large)	2.00 to 2.00
Caregiver	High Risk (Q5, Q6, Q9)	DeepSeek	Zhipu Qingyan	4.0 [3.0 to 4.0] (95% CI 4.0 to 4.0)	3.0 [2.0 to 4.0] (95% CI 3.0 to 3.0)	<.001	0.275 (small)	0.00 to 0.50
Caregiver	High Risk (Q5, Q6, Q9)	Gemini-2.5-Pro	OpenEvidence	5.0 [3.0 to 5.0] (95% CI 4.0 to 5.0)	1.0 [1.0 to 2.0] (95% CI 1.0 to 1.0)	<.001	0.774 (large)	2.00 to 2.50
Caregiver	High Risk (Q5, Q6, Q9)	Gemini-2.5-Pro	Zhipu Qingyan	5.0 [3.0 to 5.0] (95% CI 4.0 to 5.0)	3.0 [2.0 to 4.0] (95% CI 3.0 to 3.0)	<.001	0.409 (moderate)	0.50 to 1.00
Caregiver	High Risk (Q5, Q6, Q9)	OpenEvidence	Zhipu Qingyan	1.0 [1.0 to 2.0] (95% CI 1.0 to 1.0)	3.0 [2.0 to 4.0] (95% CI 3.0 to 3.0)	<.001	−0.630 (large)	−2.00 to −1.50
Caregiver	Medium Risk (Q2, Q7, Q8)	ChatGPT-4o	DeepSeek	2.0 [2.0 to 3.0] (95% CI 2.0 to 3.0)	4.0 [3.0 to 4.0] (95% CI 3.0 to 4.0)	<.001	−0.387 (moderate)	−1.00 to −0.50
Caregiver	Medium Risk (Q2, Q7, Q8)	ChatGPT-4o	Gemini-2.5-Pro	2.0 [2.0 to 3.0] (95% CI 2.0 to	4.0 [3.0 to 5.0] (95% CI 4.0 to	<.001	−0.558 (large)	−1.50 to −1.00

Perspective	Risk_Group	Model A	Model B	Score Model A	Score Model B	*P*-adj	Effect Size *r*	Diff 95% CI
				3.0)	5.0)			
Caregiver	Medium Risk (Q2, Q7, Q8)	ChatGPT-4o	OpenEvidence	2.0 [2.0 to 3.0] (95% CI 2.0 to 3.0)	2.0 [1.0 to 2.0] (95% CI 1.0 to 2.0)	<.001	0.369 (moderate)	0.50 to 1.00
Caregiver	Medium Risk (Q2, Q7, Q8)	ChatGPT-4o	Zhipu Qingyan	2.0 [2.0 to 3.0] (95% CI 2.0 to 3.0)	3.0 [2.0 to 4.0] (95% CI 3.0 to 3.0)	<.001	−0.294 (small)	−1.00 to −0.50
Caregiver	Medium Risk (Q2, Q7, Q8)	DeepSeek	Gemini-2.5-Pro	4.0 [3.0 to 4.0] (95% CI 3.0 to 4.0)	4.0 [3.0 to 5.0] (95% CI 4.0 to 5.0)	<.001	−0.290 (small)	−1.00 to −0.50
Caregiver	Medium Risk (Q2, Q7, Q8)	DeepSeek	OpenEvidence	4.0 [3.0 to 4.0] (95% CI 3.0 to 4.0)	2.0 [1.0 to 2.0] (95% CI 1.0 to 2.0)	<.001	0.658 (large)	1.50 to 2.00
Caregiver	Medium Risk (Q2, Q7, Q8)	DeepSeek	Zhipu Qingyan	4.0 [3.0 to 4.0] (95% CI 3.0 to 4.0)	3.0 [2.0 to 4.0] (95% CI 3.0 to 3.0)	.04	0.139 (small)	0.00 to 0.50
Caregiver	Medium Risk (Q2, Q7, Q8)	Gemini-2.5-Pro	OpenEvidence	4.0 [3.0 to 5.0] (95% CI 4.0 to 5.0)	2.0 [1.0 to 2.0] (95% CI 1.0 to 2.0)	<.001	0.734 (large)	2.00 to 2.50
Caregiver	Medium Risk (Q2, Q7, Q8)	Gemini-2.5-Pro	Zhipu Qingyan	4.0 [3.0 to 5.0] (95% CI 4.0 to 5.0)	3.0 [2.0 to 4.0] (95% CI 3.0 to 3.0)	<.001	0.363 (moderate)	0.50 to 1.00
Caregiver	Medium Risk (Q2, Q7, Q8)	OpenEvidence	Zhipu Qingyan	2.0 [1.0 to 2.0] (95% CI 1.0 to 2.0)	3.0 [2.0 to 4.0] (95% CI 3.0 to 3.0)	<.001	−0.585 (large)	−1.50 to −1.00

Perspective	Risk_Group	Model A	Model B	Score Model A	Score Model B	*P*-adj	Effect Size *r*	Diff 95% CI
Caregiver	Low Risk (Q1, Q3, Q4, Q10)	ChatGPT-4o	DeepSeek	3.0 [2.0 to 4.0] (95% CI 2.0 to 3.0)	3.0 [2.0 to 4.0] (95% CI 3.0 to 4.0)	<.001	−0.259 (small)	−0.50 to −0.50
Caregiver	Low Risk (Q1, Q3, Q4, Q10)	ChatGPT-4o	Gemini-2.5-Pro	3.0 [2.0 to 4.0] (95% CI 2.0 to 3.0)	5.0 [3.0 to 5.0] (95% CI 4.0 to 5.0)	<.001	−0.564 (large)	−1.50 to −1.00
Caregiver	Low Risk (Q1, Q3, Q4, Q10)	ChatGPT-4o	OpenEvidence	3.0 [2.0 to 4.0] (95% CI 2.0 to 3.0)	2.0 [1.0 to 3.0] (95% CI 1.0 to 2.0)	<.001	0.363 (moderate)	0.50 to 1.00
Caregiver	Low Risk (Q1, Q3, Q4, Q10)	ChatGPT-4o	Zhipu Qingyan	3.0 [2.0 to 4.0] (95% CI 2.0 to 3.0)	3.0 [2.0 to 4.0] (95% CI 3.0 to 3.0)	>.99	−0.030 (negligible)	−0.00 to 0.00
Caregiver	Low Risk (Q1, Q3, Q4, Q10)	DeepSeek	Gemini-2.5-Pro	3.0 [2.0 to 4.0] (95% CI 3.0 to 4.0)	5.0 [3.0 to 5.0] (95% CI 4.0 to 5.0)	<.001	−0.423 (moderate)	−1.00 to −0.50
Caregiver	Low Risk (Q1, Q3, Q4, Q10)	DeepSeek	OpenEvidence	3.0 [2.0 to 4.0] (95% CI 3.0 to 4.0)	2.0 [1.0 to 3.0] (95% CI 1.0 to 2.0)	<.001	0.584 (large)	1.00 to 1.50
Caregiver	Low Risk (Q1, Q3, Q4, Q10)	DeepSeek	Zhipu Qingyan	3.0 [2.0 to 4.0] (95% CI 3.0 to 4.0)	3.0 [2.0 to 4.0] (95% CI 3.0 to 3.0)	<.001	0.228 (small)	0.00 to 0.50
Caregiver	Low Risk (Q1, Q3, Q4, Q10)	Gemini-2.5-Pro	OpenEvidence	5.0 [3.0 to 5.0] (95% CI 4.0 to 5.0)	2.0 [1.0 to 3.0] (95% CI 1.0 to 2.0)	<.001	0.722 (large)	2.00 to 2.50
Caregiver	Low Risk	Gemini-2.5-Pro	Zhipu Qingyan	5.0 [3.0	3.0 [2.0	<.001	0.519	1.00 to

Perspective	Risk_Group	Model A	Model B	Score Model A	Score Model B	*P*-adj	Effect Size *r*	Diff 95% CI
	(Q1, Q3, Q4, Q10)			to 5.0] (95% CI 4.0 to 5.0)	to 4.0] (95% CI 3.0 to 3.0)		(large)	1.50
Caregiver	Low Risk (Q1, Q3, Q4, Q10)	OpenEvidence	Zhipu Qingyan	2.0 [1.0 to 3.0] (95% CI 1.0 to 2.0)	3.0 [2.0 to 4.0] (95% CI 3.0 to 3.0)	<.001	−0.447 (moderate)	−1.00 to −0.50

The risk-stratified analysis shows that model differences increase with clinical risk: the gap between the best and worst models is larger for high-risk questions than for low-risk questions. These results are shown in Figure 4 in the main text and are also presented in the figure below.

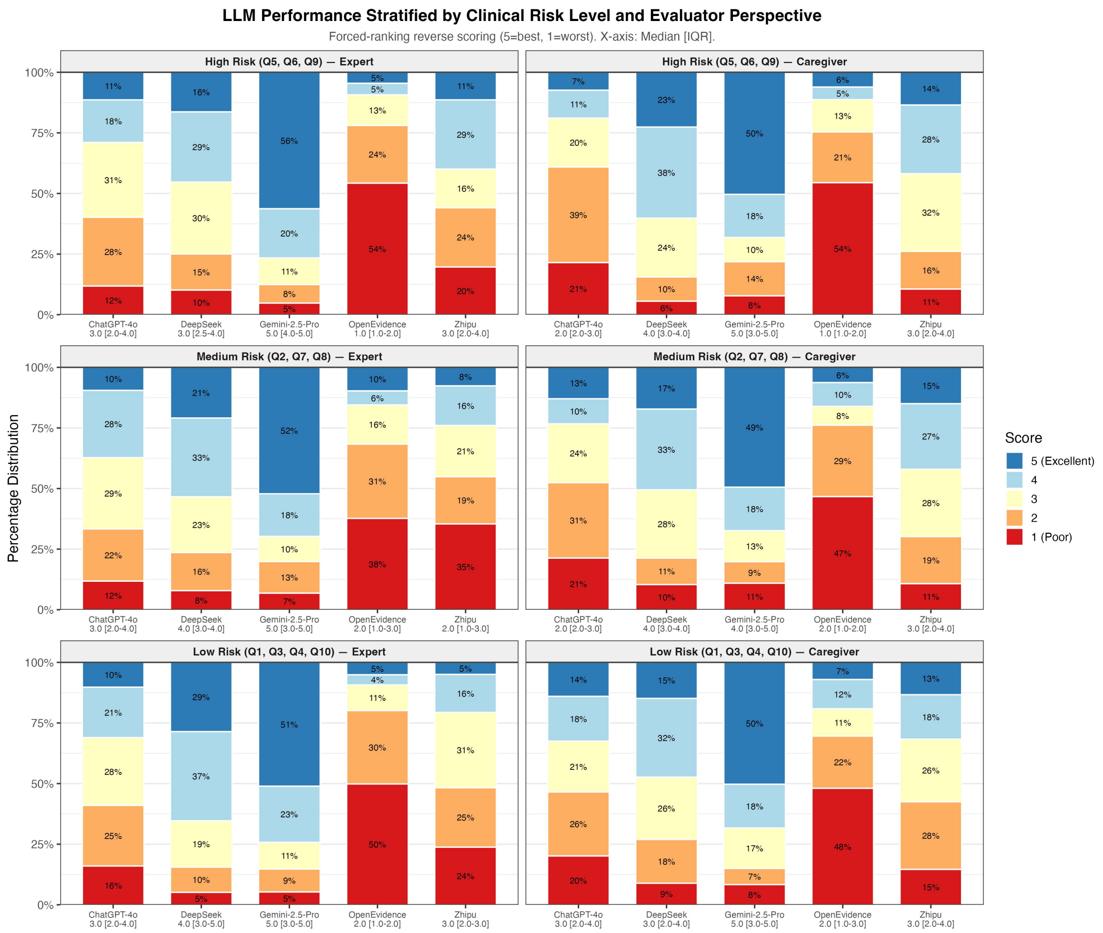

Supplement: Multimedia Appendix 9 [file jmir-v28-e93393-s009.pdf]
