# Supplementary material for: Performance of 5 Large Language Models in Perioperative Consultation for Pediatric Hypospadias: Cross-Sectional Comparative Study
Source: J Med Internet Res. 2026 Jul 29;28:e93393. doi: 10.2196/93393 (PMC13419283; doi:10.2196/93393)
Supplement: Multimedia Appendix 10 [file jmir-v28-e93393-s010.pdf]

Demographic subgroup stratified analysis

Subgroup 1. Hypospadias type (Wilcoxon rank-sum).

Model	Distal/Midshaft (Type I-II)	Proximal/Severe (Type III-IV)	Method	*P* value	Effect Size
ChatGPT-4o	2 [2-4] (95% CI 2.0-3.0)	2 [2-4] (95% CI 2.0-3.0)	Wilcoxon	>.99	0.000 (small)
DeepSeek	4 [3-4] (95% CI 4.0-4.0)	3 [3-4] (95% CI 3.0-3.0)	Wilcoxon	.046	0.336 (moderate)
Gemini-2.5-Pro	4 [3-5] (95% CI 4.0-4.0)	5 [3-5] (95% CI 5.0-5.0)	Wilcoxon	.61	0.088 (small)
OpenEvidence	2 [1-3] (95% CI 1.0-2.0)	2 [1-3] (95% CI 1.0-2.0)	Wilcoxon	.57	0.097 (small)
Zhipu Qingyan	3 [2-4] (95% CI 3.0-3.0)	3 [2-4] (95% CI 3.0-3.0)	Wilcoxon	.42	0.139 (small)

Subgroup 2. Caregiver education (Kruskal–Wallis omnibus and Jonckheere–Terpstra trend tests).

Model	High School or Below	Vocational/Junior College	Undergraduate or Above	Method	*P* value	*P* trend
ChatGPT-4o	2 [1-3] (95% CI 2.0-3.0)	2 [2-4] (95% CI 2.0-3.0)	2 [2-3] (95% CI 2.0-3.0)	Kruskal-Wallis + Jonckheere-Terpstra	.57	.77
DeepSeek	4 [2-4] (95% CI 4.0-4.0)	3 [3-4] (95% CI 3.0-4.0)	4 [3-4] (95% CI 3.0-4.0)	Kruskal-Wallis + Jonckheere-Terpstra	.67	.41
Gemini-2.5-Pro	4 [3-5] (95% CI 4.0-4.0)	4 [3-5] (95% CI 4.0-5.0)	5 [3-5] (95% CI 5.0-5.0)	Kruskal-Wallis + Jonckheere-Terpstra	.39	.15
Open-Evidence	2 [1-3] (95% CI 2.0-2.0)	1 [1-3] (95% CI 1.0-2.0)	2 [1-3] (95% CI 1.0-2.0)	Kruskal-Wallis + Jonckheere-Terpstra	.21	.31
Zhipu Qingyan	3 [2-4] (95% CI 3.0-3.0)	3 [2-4] (95% CI 3.0-3.0)	3 [2-4] (95% CI 3.0-3.0)	Kruskal-Wallis +Jonckheere-Terpstra	.14	.17

Subgroup 3. Family income (Kruskal–Wallis omnibus and Jonckheere–Terpstra trend tests).

Model	Low (≤3500)	Medium (3501-5400)	High (>5400)	Method	*P* value	*P* trend
ChatGPT-4o	2 [2-3] (95% CI 2.0-3.0)	2 [2-4] (95% CI 2.0-3.0)	2 [2-4] (95% CI 2.0-3.0)	Kruskal-Wallis + Jonckheere-Terpstra	.89	.71
DeepSeek	4 [3-5] (95% CI	3 [3-4] (95% CI	4 [3-4] (95% CI	Kruskal-Wallis +	.65	.64

Model	Low (≤3500)	Medium (3501-5400)	High (>5400)	Method	*P* value	*P* trend
	4.0-4.0)	3.0-4.0)	3.0-4.0)	Jonckheere-Terpstra		
Gemini-2.5-Pro	4 [2-5] (95% CI 3.0-4.0)	5 [3-5] (95% CI 4.0-5.0)	5 [3-5] (95% CI 4.0-5.0)	Kruskal-Wallis + Jonckheere-Terpstra	.12	.09
Open-Evidence	2 [1-4] (95% CI 2.0-2.0)	2 [1-3] (95% CI 1.0-2.0)	1 [1-2] (95% CI 1.0-2.0)	Kruskal-Wallis + Jonckheere-Terpstra	.06	.03
Zhipu Qingyan	3 [2-4] (95% CI 3.0-3.0)	3 [2-4] (95% CI 3.0-3.0)	3 [2-4] (95% CI 3.0-3.0)	Kruskal-Wallis + Jonckheere-Terpstra	.55	.49

Subgroup 4. Employment status (Wilcoxon rank-sum).

Model	Employed/ Freelancer	Unemployed/ Homemaker	Method	*P* value	Effect Size
ChatGPT-4o	2 [2-4] (95% CI 2.0-3.0)	2 [2-4] (95% CI 2.0-3.0)	Wilcoxon	.96	0.012 (small)
DeepSeek	3 [3-4] (95% CI 3.0-4.0)	4 [3-4] (95% CI 4.0-4.0)	Wilcoxon	.27	0.188 (small)
Gemini-2.5-Pro	5 [3-5] (95% CI 5.0-5.0)	4 [3-5] (95% CI 4.0-4.0)	Wilcoxon	.06	0.315 (moderate)
OpenEvidence	1 [1-2] (95% CI 1.0-2.0)	2 [1-3] (95% CI 2.0-2.0)	Wilcoxon	.11	0.271 (small)
Zhipu Qingyan	3 [2-4] (95% CI 3.0-3.0)	3 [2-4] (95% CI 3.0-3.0)	Wilcoxon	.52	0.109 (small)

Subgroup 5. Expert professional title (Kruskal–Wallis omnibus and Jonckheere–Terpstra trend tests).

Model	Junior (Staff Nurse)	Intermediate (Attending/ Nurse-in-Charge)	Senior (Associate Chief/ Chief)	Method	*P* value	*P* trend
ChatGPT-4o	3 [2-4] (95% CI 3.0-3.0)	3 [2-4] (95% CI 3.0-3.0)	3 [2-4] (95% CI 3.0-3.0)	Kruskal-Wallis + Jonckheere-Terpstra	.15	.04
DeepSeek	4 [2-5] (95% CI 4.0-4.0)	4 [3-4] (95% CI 3.0-4.0)	4 [3-4] (95% CI 4.0-4.0)	Kruskal-Wallis + Jonckheere-Terpstra	.68	.59
Gemini-2.5-Pro	4 [2-5] (95% CI 4.0-4.0)	5 [4-5] (95% CI 5.0-5.0)	5 [4-5] (95% CI 5.0-5.0)	Kruskal-Wallis + Jonckheere-Terpstra	.004	<.001

Model	Junior (Staff Nurse)	Intermediate (Attending/ Nurse-in-Charge)	Senior (Associate Chief/ Chief)	Method	*P* value	*P* trend
Open-Evidence	2 [1-3] (95% CI 2.0-2.0)	2 [1-2] (95% CI 1.0-2.0)	1 [1-2] (95% CI 1.0-1.0)	Kruskal-Wallis + Jonckheere-Terpstra	.03	.007
Zhipu Qingyan	3 [2-4] (95% CI 2.0-3.0)	2 [1-4] (95% CI 2.0-3.0)	3 [2-3] (95% CI 2.0-3.0)	Kruskal-Wallis + Jonckheere-Terpstra	.91	.65

Subgroup 6. Expert age tertiles (Kruskal–Wallis omnibus and Jonckheere–Terpstra trend tests).

Model	Young (≤32 yrs)	Middle (33-39 yrs)	Senior (>39 yrs)	Method	*P* value	*P* trend
ChatGPT-4o	3 [2-4] (95% CI 3.0-3.0)	3 [2-4] (95% CI 3.0-3.0)	3 [2-4] (95% CI 3.0-3.0)	Kruskal-Wallis + Jonckheere-Terpstra	.61	.27
DeepSeek	4 [3-5] (95% CI 4.0-4.0)	4 [3-4] (95% CI 4.0-4.0)	3 [3-4] (95% CI 3.0-4.0)	Kruskal-Wallis + Jonckheere-Terpstra	.65	.37
Gemini-2.5-Pro	4 [3-5] (95% CI 4.0-4.0)	5 [3-5] (95% CI 5.0-5.0)	5 [4-5] (95% CI 5.0-5.0)	Kruskal-Wallis + Jonckheere-Terpstra	.07	.02
OpenEvidence	2 [1-3] (95% CI 2.0-2.0)	2 [1-3] (95% CI 1.0-2.0)	1 [1-2] (95% CI 1.0-2.0)	Kruskal-Wallis + Jonckheere-Terpstra	.27	.17
Zhipu Qingyan	3 [1-4] (95% CI 2.0-3.0)	2 [1-3] (95% CI 2.0-3.0)	3 [2-4] (95% CI 3.0-3.0)	Kruskal-Wallis + Jonckheere-Terpstra	.69	.57

Subgroup 7. Expert clinical experience tertiles (Kruskal–Wallis omnibus and Jonckheere–Terpstra trend tests).

Model	Short (≤7 yrs)	Medium (8-18 yrs)	Long (>18 yrs)	Method	*P* value	*P* trend
ChatGPT-4o	3 [2-4] (95% CI 3.0-3.0)	3 [2-4] (95% CI 3.0-3.0)	3 [2-4] (95% CI 3.0-3.0)	Kruskal-Wallis + Jonckheere-Terpstra	.45	.25
DeepSeek	4 [3-5] (95% CI 4.0-4.0)	4 [3-4] (95% CI 3.0-4.0)	4 [3-4] (95% CI 4.0-4.0)	Kruskal-Wallis + Jonckheere-Terpstra	.77	.50
Gemini-2.5-Pro	4 [2-5] (95% CI 4.0-4.0)	5 [4-5] (95% CI 5.0-5.0)	5 [4-5] (95% CI 5.0-5.0)	Kruskal-Wallis + Jonckheere-Terpstra	.06	.04
OpenEvidence	2 [1-3] (95% CI 1.0-2.0)	2 [1-3] (95% CI 2.0-2.0)	1 [1-2] (95% CI 1.0-2.0)	Kruskal-Wallis + Jonckheere-Terpstra	.36	.25
Zhipu Qingyan	3 [2-4] (95% CI 2.0-3.0)	2 [1-4] (95% CI 2.0-3.0)	3 [2-4] (95% CI 2.0-3.0)	Kruskal-Wallis + Jonckheere-Terpstra	.84	.84

Subgroup 8 (clinical risk level) is reported in Multimedia Appendix 12. P<.05 indicates statistical significance. Corresponding visualisations for each subgroup are presented in the sections that follow.

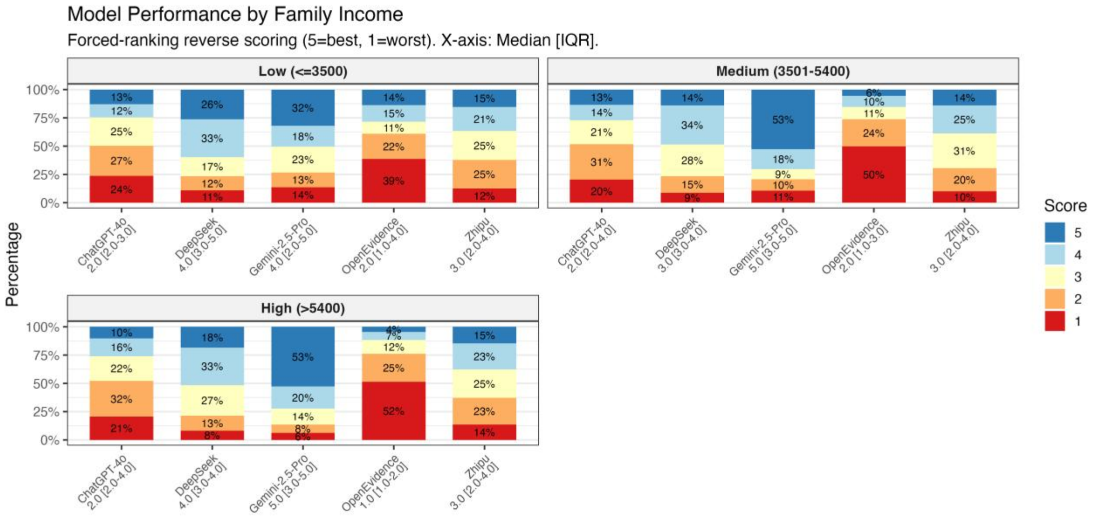

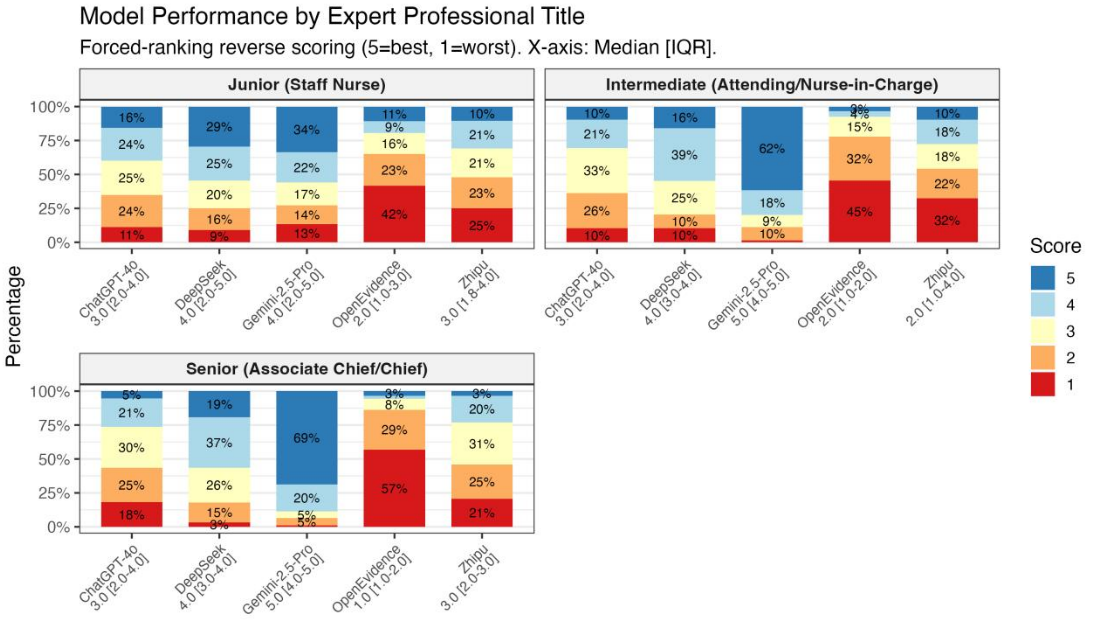

Model Performance by Clinical Experience (Tertiles)

Forced-ranking reverse scoring (5=best, 1=worst). X-axis: Median [IQR].

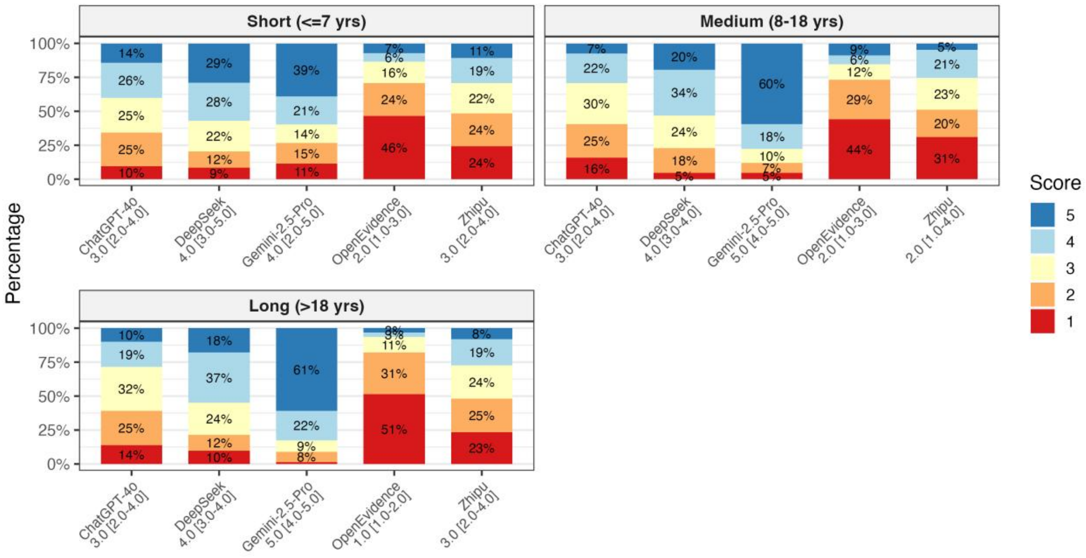

Model Performance by Expert Age (Tertiles)

Forced-ranking reverse scoring (5=best, 1=worst). X-axis: Median [IQR].

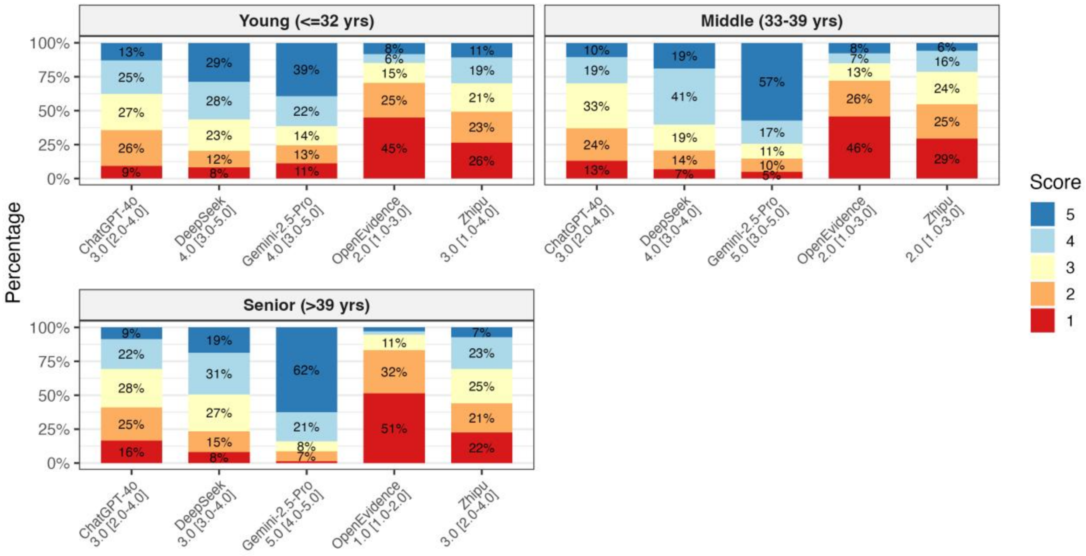

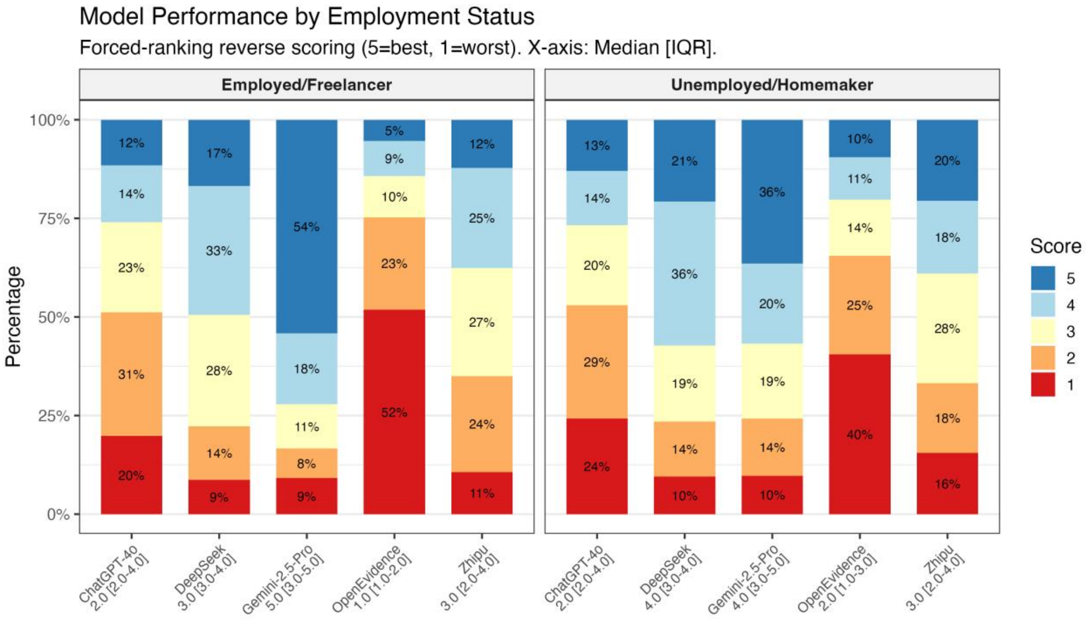
Model Performance by Employment Status
Forced-ranking reverse scoring (5=best, 1=worst). X-axis: Median [IQR].
Employed/Freelancer
Unemployed/Homemaker
Percentage
100%
75%
50%
25%
0%
Score
5
4
3
2
1
ChatGPT-4o 2.0 [2.0-4.0]
DeepSeek 3.0 [3.0-4.0]
Gemini-2.5-Pro 5.0 [3.0-5.0]
OpenEvidence 1.0 [1.0-2.0]
Zhipu 3.0 [2.0-4.0]
ChatGPT-4o 2.0 [2.0-4.0]
DeepSeek 4.0 [3.0-4.0]
Gemini-2.5-Pro 4.0 [3.0-5.0]
OpenEvidence 2.0 [1.0-3.0]
Zhipu 3.0 [2.0-4.0]

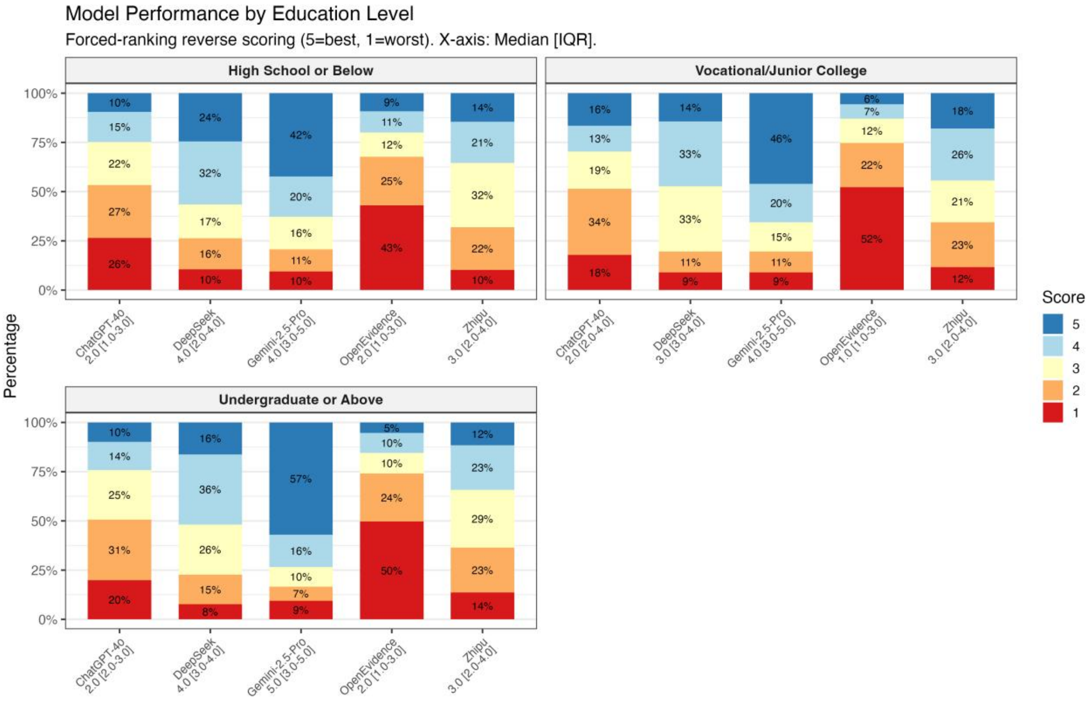
Model Performance by Education Level
Forced-ranking reverse scoring (5=best, 1=worst). X-axis: Median [IQR].
High School or Below
Vocational/Junior College
Undergraduate or Above
Percentage
Score
5
4
3
2
1

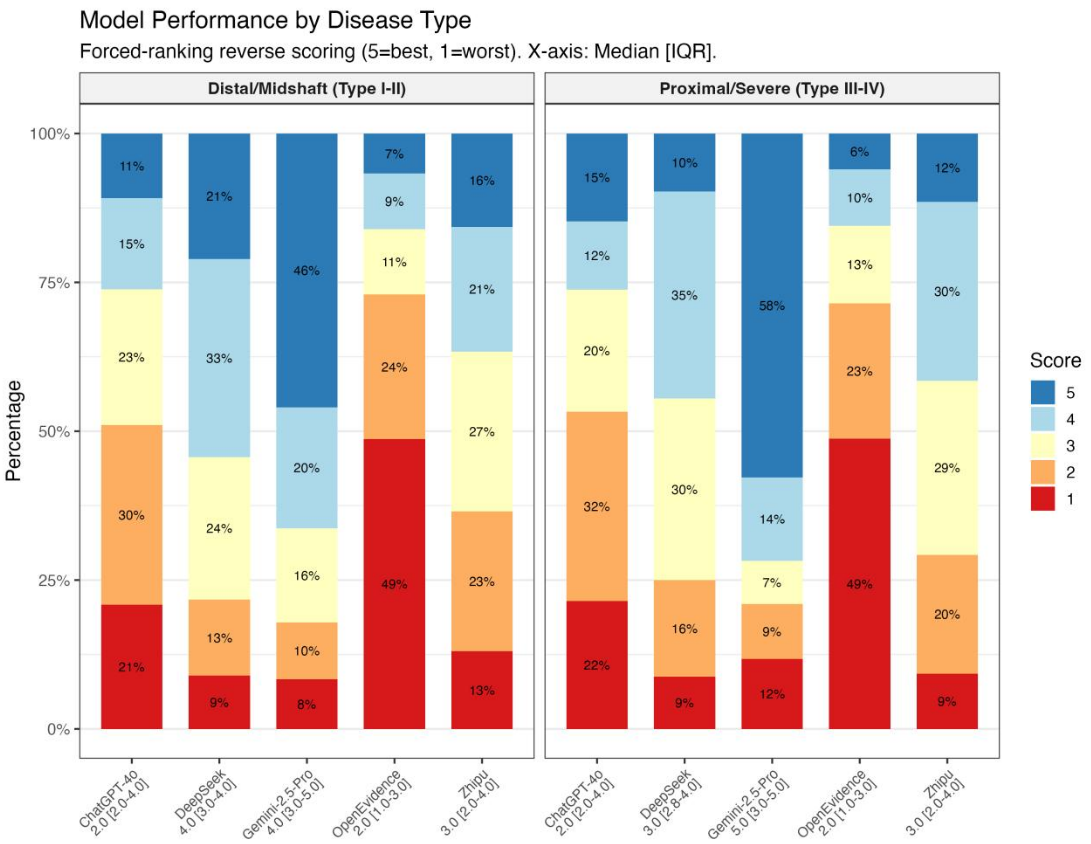

Model Performance by Disease Type
Forced-ranking reverse scoring (5=best, 1=worst). X-axis: Median [IQR].
Distal/Midshaft (Type I-II)
Proximal/Severe (Type III-IV)
Percentage
100%
75%
50%
25%
0%
Score
5
4
3
2
1
ChatGPT-4o 2.0 [2.0-4.0]
DeepSeek 4.0 [3.0-4.0]
Gemini-2.5-Pro 4.0 [3.0-5.0]
OpenEvidence 2.0 [1.0-3.0]
Zhipu 3.0 [2.0-4.0]
ChatGPT-4o 2.0 [2.0-4.0]
DeepSeek 3.0 [2.8-4.0]
Gemini-2.5-Pro 5.0 [3.0-5.0]
OpenEvidence 2.0 [1.0-3.0]
Zhipu 3.0 [2.0-4.0]
