# Supplementary material for: Performance of 5 Large Language Models in Perioperative Consultation for Pediatric Hypospadias: Cross-Sectional Comparative Study
Source: J Med Internet Res. 2026 Jul 29;28:e93393. doi: 10.2196/93393 (PMC13419283; doi:10.2196/93393)
Supplement: Multimedia Appendix 11 [file jmir-v28-e93393-s011.pdf]

Per-question expert ratings

This appendix presents granular per-question ratings from 23 pediatric urology specialists across 10 perioperative questions, comprising 10 stacked bar charts and 10 violin (rain-cloud) plots. Together with Multimedia Appendix 9 (caregiver ratings), these figures provide detailed question-level data that complement the overall and dimension-level results shown in Figure 2 and Multimedia Appendix 10.

How to read the figures.

Visual element	Meaning
Stacked bar chart (each question)	Each bar represents one of the five models; the bar is segmented by score (1–5) using a diverging Likert colour scheme (blue–yellow–red); segment lengths show the proportion of evaluations falling at each score within that model. Total evaluations per model per question = 138 (23 experts × 6 dimensions).
Violin (rain-cloud) plot (each question)	The "cloud" (left side) shows the kernel density of the score distribution by model; the "rain" (right side) shows the individual data points jittered for visibility; the inner box shows the median and interquartile range. Score values 1–5 reflect the forced-ranking reverse-scoring scheme (5 = best, 1 = worst).
Score colour scheme (1–5)	5 = dark blue (Excellent); 4 = light blue (Good); 3 = pale yellow (Average); 2 = orange (Fair); 1 = red (Poor).
Friedman annotation	The Friedman omnibus result is annotated as "Friedman: χ^2(df) = X, P, W = Y" at the top of each panel. P values are presented as "P < .001" or to three decimal places. W = Kendall's coefficient of concordance.
Pairwise significance markers	Bonferroni-corrected pairwise Wilcoxon signed-rank comparisons are annotated with exact *P* values are annotated above pairwise comparison bars. *P* values omit leading zeros and are reported to two decimal places for *P* > .01, three decimal places for .001 ≤ *P* ≤ .01, and marked "<.001" for *P* < .001. All significance thresholds are kept to three decimal places to prevent rounding flip errors. Asterisk markers (//) and "ns" labels are removed; non-significant pairs are omitted to reduce visual clutter (corrected *α*' = .005).
Model labels on the x-axis	The five LLM identities are shown unblinded (ChatGPT-4o, DeepSeek, Gemini-2.5-Pro, OpenEvidence, Zhipu Qingyan); during the actual evaluation, evaluators saw only the blinded AI1 – AI5 labels with a per-question randomised mapping (the unblinding key is in Multimedia Appendix 4).

Stacked Bar Charts: Per-Question Expert Rating Distributions

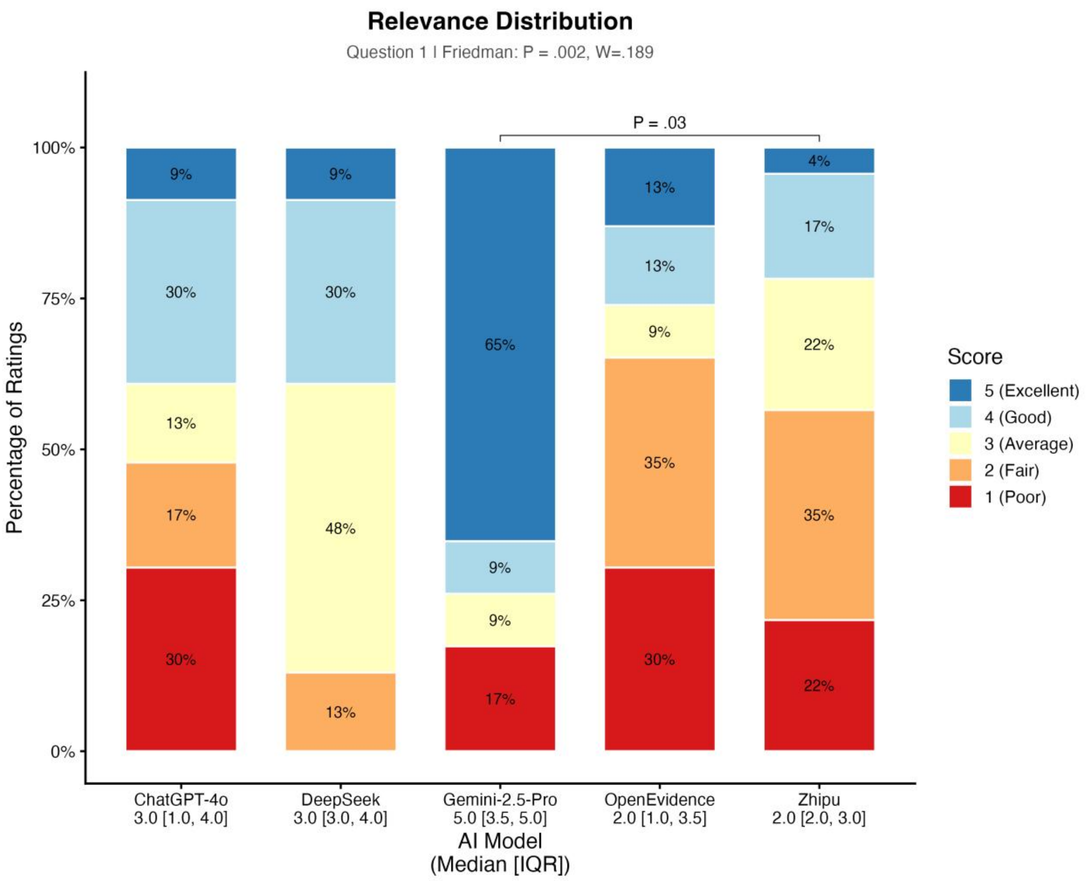

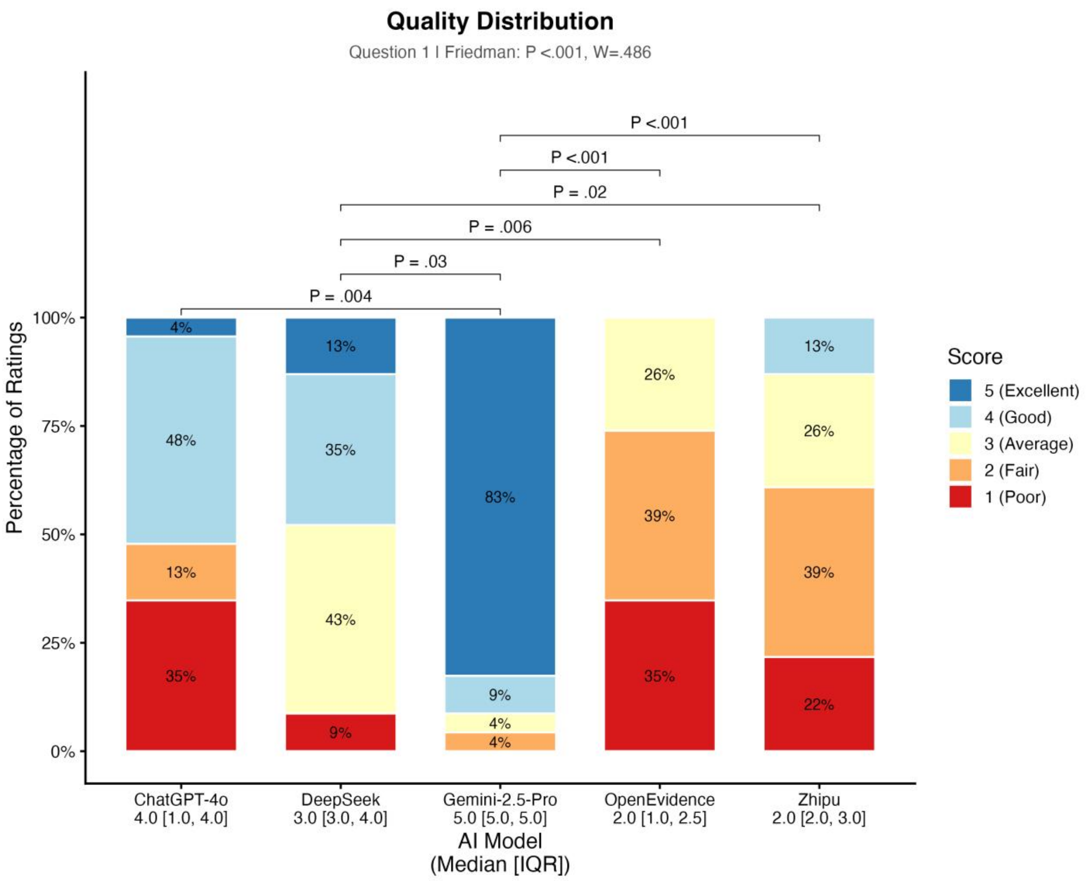
Quality Distribution
Question 1 | Friedman: P <.001, W=.486
P <.001
P <.001
P = .02
P = .006
P = .03
P = .004
Percentage of Ratings
100%
75%
50%
25%
0%
4%
48%
13%
35%
13%
35%
43%
9%
83%
9%
4%
4%
26%
39%
35%
13%
26%
39%
22%
ChatGPT-4o
4.0 [1.0, 4.0]
DeepSeek
3.0 [3.0, 4.0]
Gemini-2.5-Pro
5.0 [5.0, 5.0]
OpenEvidence
2.0 [1.0, 2.5]
Zhipu
2.0 [2.0, 3.0]
AI Model
(Median [IQR])
Score
5 (Excellent)
4 (Good)
3 (Average)
2 (Fair)
1 (Poor)

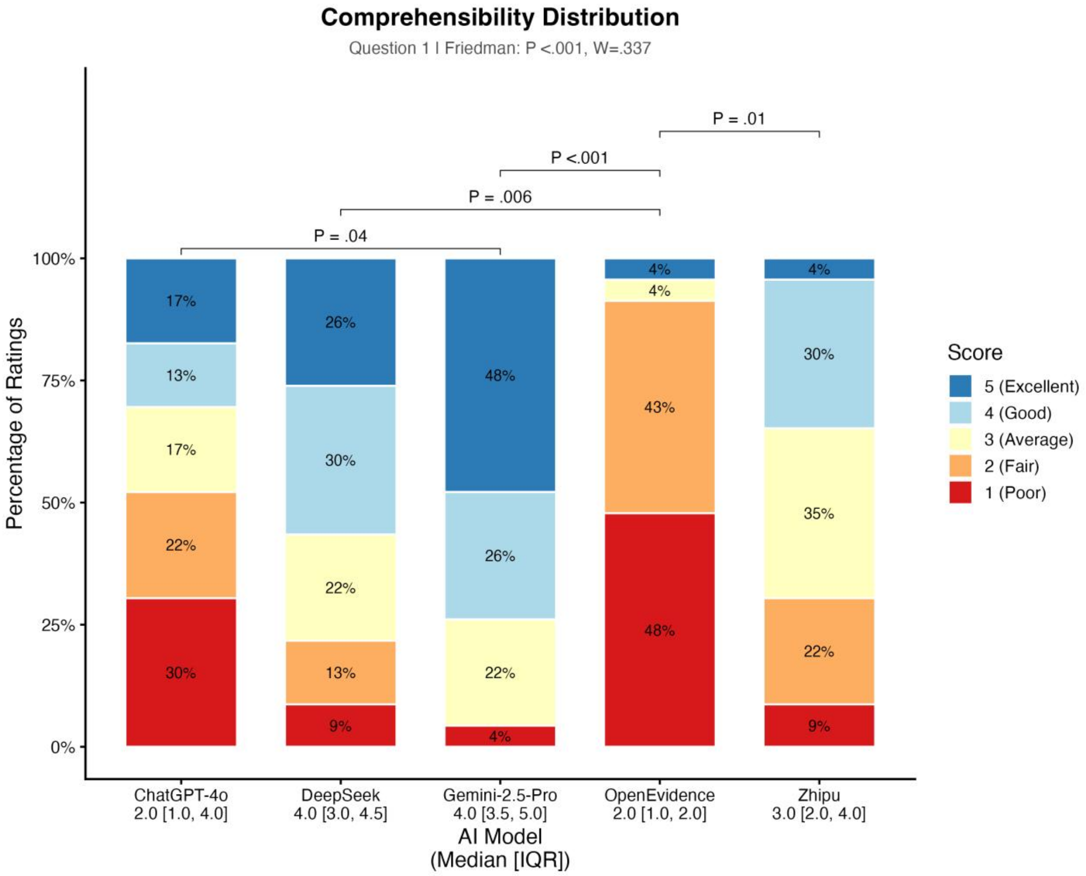
Comprehensibility Distribution
Question 1 | Friedman: P <.001, W=.337
P = .01
P <.001
P = .006
P = .04
Percentage of Ratings
100%
75%
50%
25%
0%
17%
13%
17%
22%
30%
26%
30%
22%
13%
9%
48%
26%
22%
4%
4%
4%
43%
48%
4%
30%
35%
22%
9%
ChatGPT-4o
2.0 [1.0, 4.0]
DeepSeek
4.0 [3.0, 4.5]
Gemini-2.5-Pro
4.0 [3.5, 5.0]
OpenEvidence
2.0 [1.0, 2.0]
Zhipu
3.0 [2.0, 4.0]
AI Model
(Median [IQR])
Score
5 (Excellent)
4 (Good)
3 (Average)
2 (Fair)
1 (Poor)

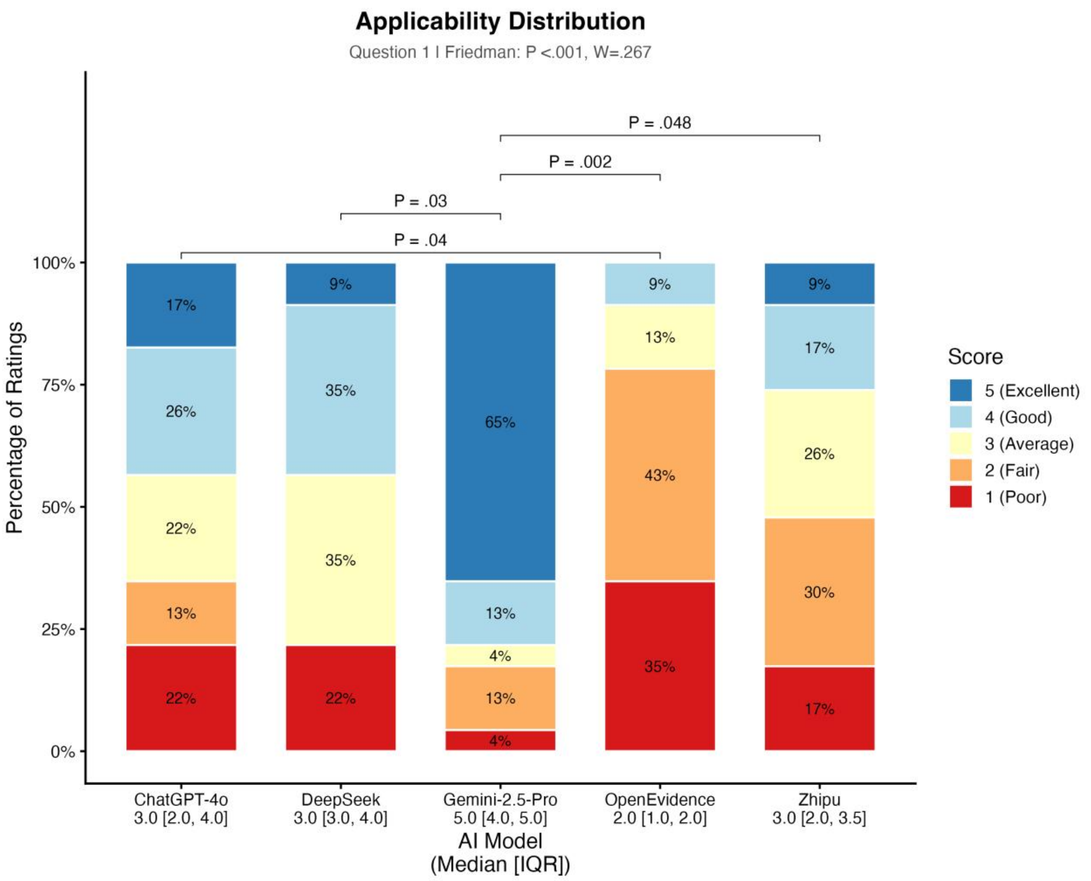
Applicability Distribution
Question 1 | Friedman: P <.001, W=.267
P = .048
P = .002
P = .03
P = .04
Percentage of Ratings
100%
75%
50%
25%
0%
17%
26%
22%
13%
22%
9%
35%
35%
22%
65%
13%
4%
13%
4%
9%
13%
43%
35%
9%
17%
26%
30%
17%
ChatGPT-4o
3.0 [2.0, 4.0]
DeepSeek
3.0 [3.0, 4.0]
Gemini-2.5-Pro
5.0 [4.0, 5.0]
OpenEvidence
2.0 [1.0, 2.0]
Zhipu
3.0 [2.0, 3.5]
AI Model
(Median [IQR])
Score
5 (Excellent)
4 (Good)
3 (Average)
2 (Fair)
1 (Poor)

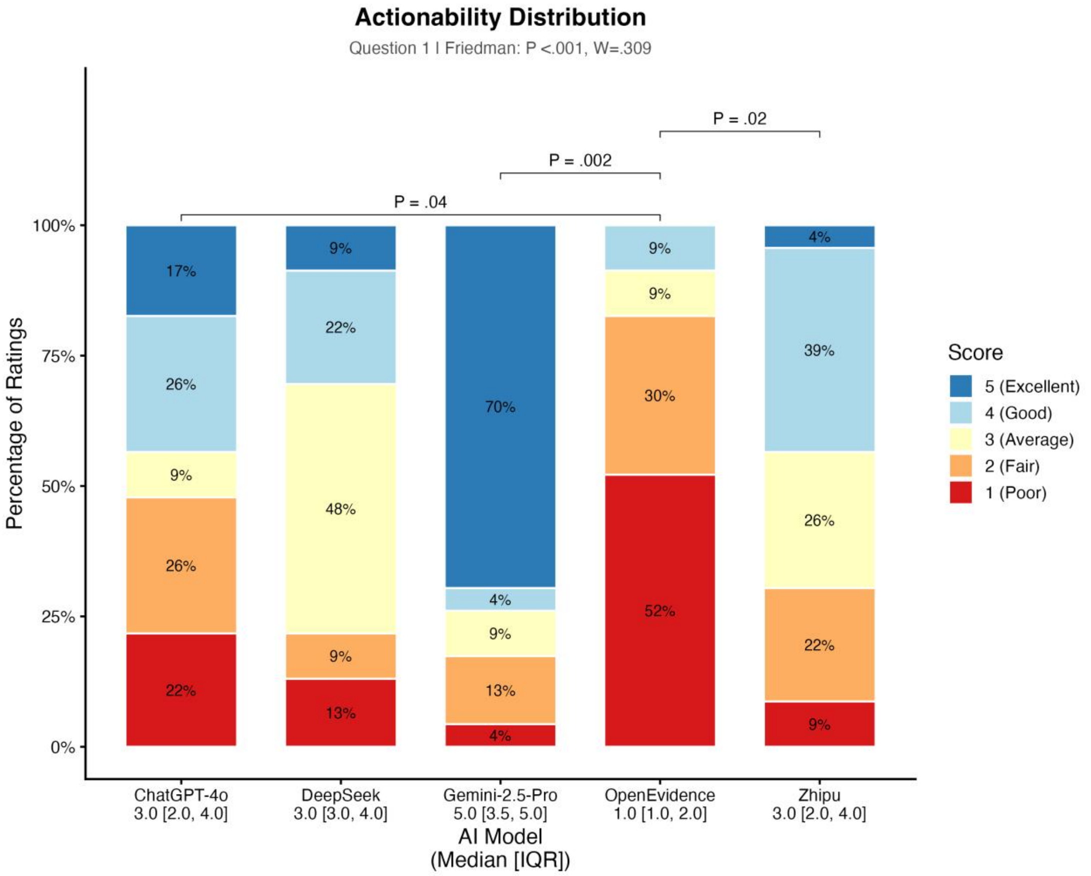
Actionability Distribution
Question 1 | Friedman: P <.001, W=.309
P = .02
P = .002
P = .04
Percentage of Ratings
100%
75%
50%
25%
0%
17%
26%
9%
26%
22%
9%
22%
48%
9%
13%
70%
4%
9%
13%
4%
9%
9%
30%
52%
4%
39%
26%
22%
9%
ChatGPT-4o
3.0 [2.0, 4.0]
DeepSeek
3.0 [3.0, 4.0]
Gemini-2.5-Pro
5.0 [3.5, 5.0]
OpenEvidence
1.0 [1.0, 2.0]
Zhipu
3.0 [2.0, 4.0]
AI Model
(Median [IQR])
Score
5 (Excellent)
4 (Good)
3 (Average)
2 (Fair)
1 (Poor)

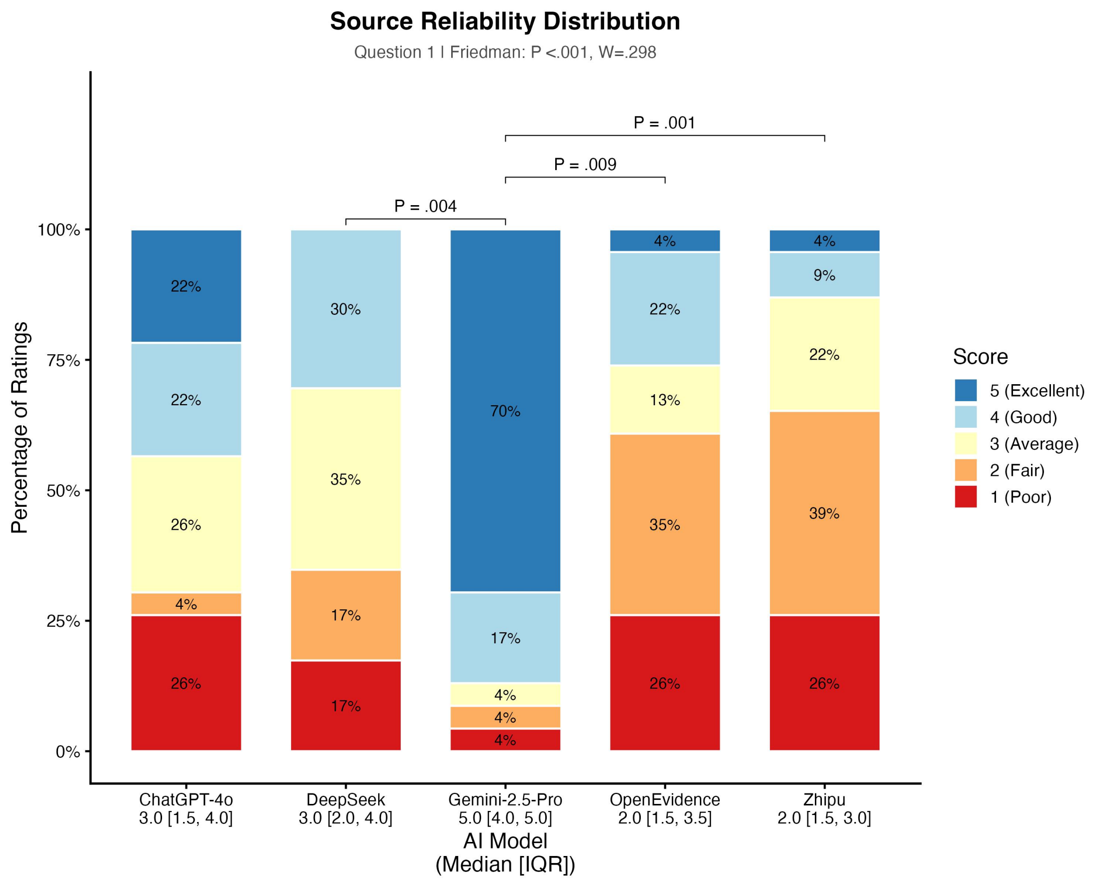

Source Reliability Distribution
Question 1 | Friedman: P <.001, W=.298
Percentage of Ratings
100%
75%
50%
25%
0%
P = .001
P = .009
P = .004
22%
22%
26%
4%
26%
30%
35%
17%
17%
70%
17%
4%
4%
4%
4%
22%
13%
35%
26%
4%
9%
22%
39%
26%
ChatGPT-4o
3.0 [1.5, 4.0]
DeepSeek
3.0 [2.0, 4.0]
Gemini-2.5-Pro
5.0 [4.0, 5.0]
OpenEvidence
2.0 [1.5, 3.5]
Zhipu
2.0 [1.5, 3.0]
AI Model
(Median [IQR])
Score
5 (Excellent)
4 (Good)
3 (Average)
2 (Fair)
1 (Poor)

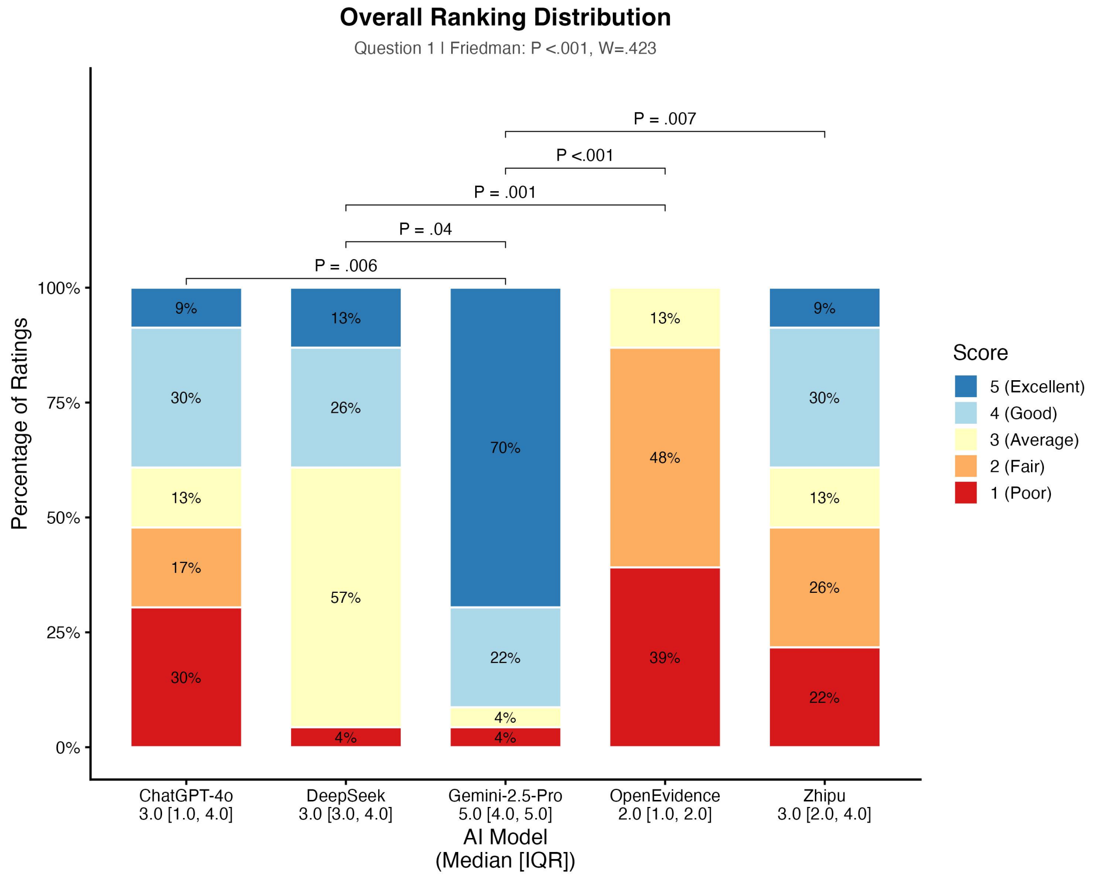

Overall Ranking Distribution
Question 1 | Friedman: P <.001, W=.423
Percentage of Ratings
100%
75%
50%
25%
0%
P = .007
P <.001
P = .001
P = .04
P = .006
9%
30%
13%
17%
30%
13%
26%
57%
4%
70%
22%
4%
4%
13%
48%
39%
9%
30%
13%
26%
22%
ChatGPT-4o
3.0 [1.0, 4.0]
DeepSeek
3.0 [3.0, 4.0]
Gemini-2.5-Pro
5.0 [4.0, 5.0]
OpenEvidence
2.0 [1.0, 2.0]
Zhipu
3.0 [2.0, 4.0]
AI Model
(Median [IQR])
Score
5 (Excellent)
4 (Good)
3 (Average)
2 (Fair)
1 (Poor)

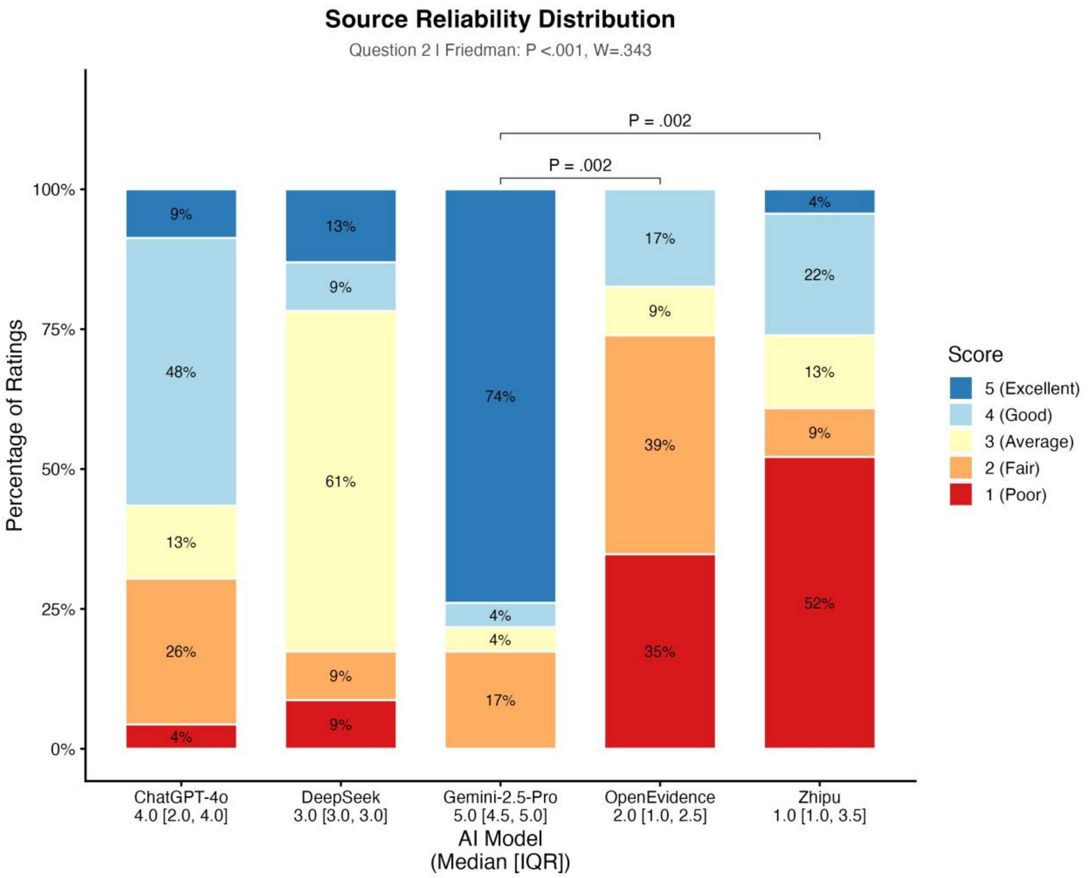
Source Reliability Distribution
Question 2 | Friedman: P <.001, W=.343
P = .002
P = .002
Percentage of Ratings
100%
75%
50%
25%
0%
9%
48%
13%
26%
4%
13%
9%
61%
9%
9%
74%
4%
4%
17%
17%
9%
39%
35%
4%
22%
13%
9%
52%
ChatGPT-4o
4.0 [2.0, 4.0]
DeepSeek
3.0 [3.0, 3.0]
Gemini-2.5-Pro
5.0 [4.5, 5.0]
OpenEvidence
2.0 [1.0, 2.5]
Zhipu
1.0 [1.0, 3.5]
AI Model
(Median [IQR])
Score
5 (Excellent)
4 (Good)
3 (Average)
2 (Fair)
1 (Poor)

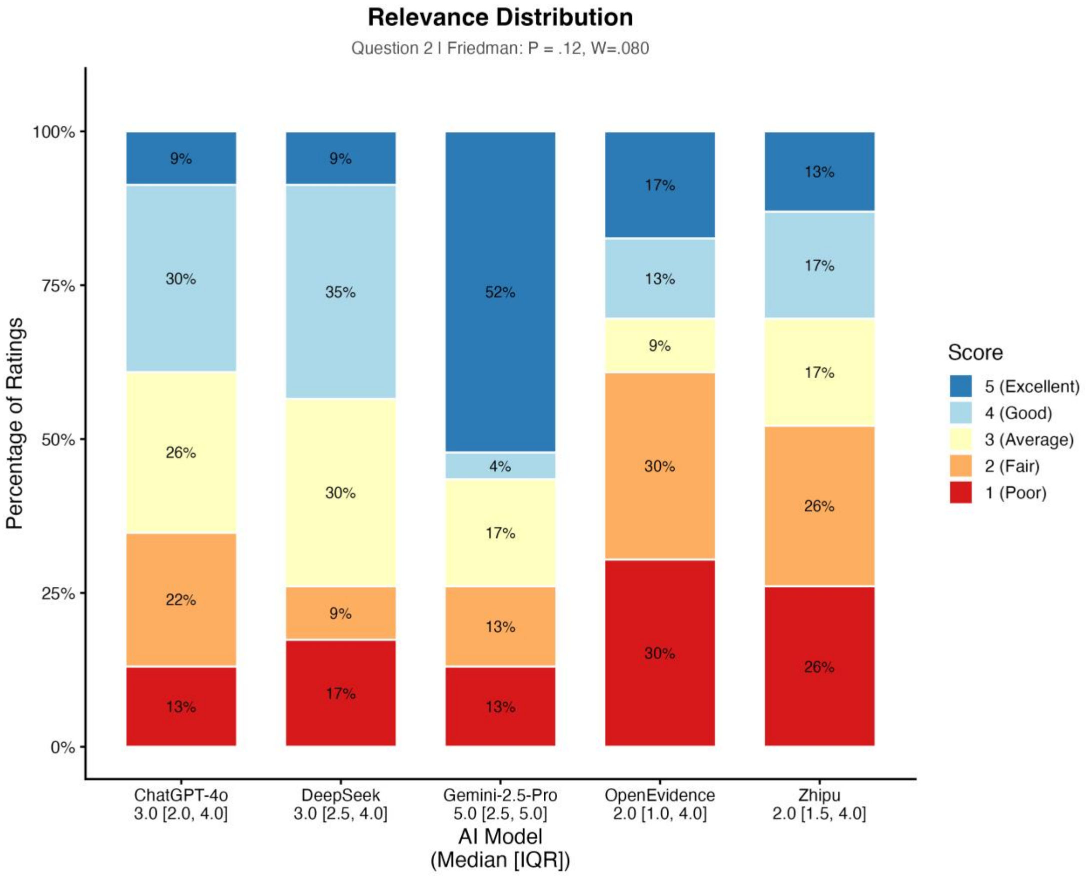
Relevance Distribution
Question 2 | Friedman: P = .12, W=.080
Percentage of Ratings
100%
75%
50%
25%
0%
9%
30%
26%
22%
13%
9%
35%
30%
9%
17%
52%
4%
17%
13%
13%
17%
13%
9%
30%
30%
13%
17%
17%
26%
26%
ChatGPT-4o
3.0 [2.0, 4.0]
DeepSeek
3.0 [2.5, 4.0]
Gemini-2.5-Pro
5.0 [2.5, 5.0]
OpenEvidence
2.0 [1.0, 4.0]
Zhipu
2.0 [1.5, 4.0]
AI Model
(Median [IQR])
Score
5 (Excellent)
4 (Good)
3 (Average)
2 (Fair)
1 (Poor)

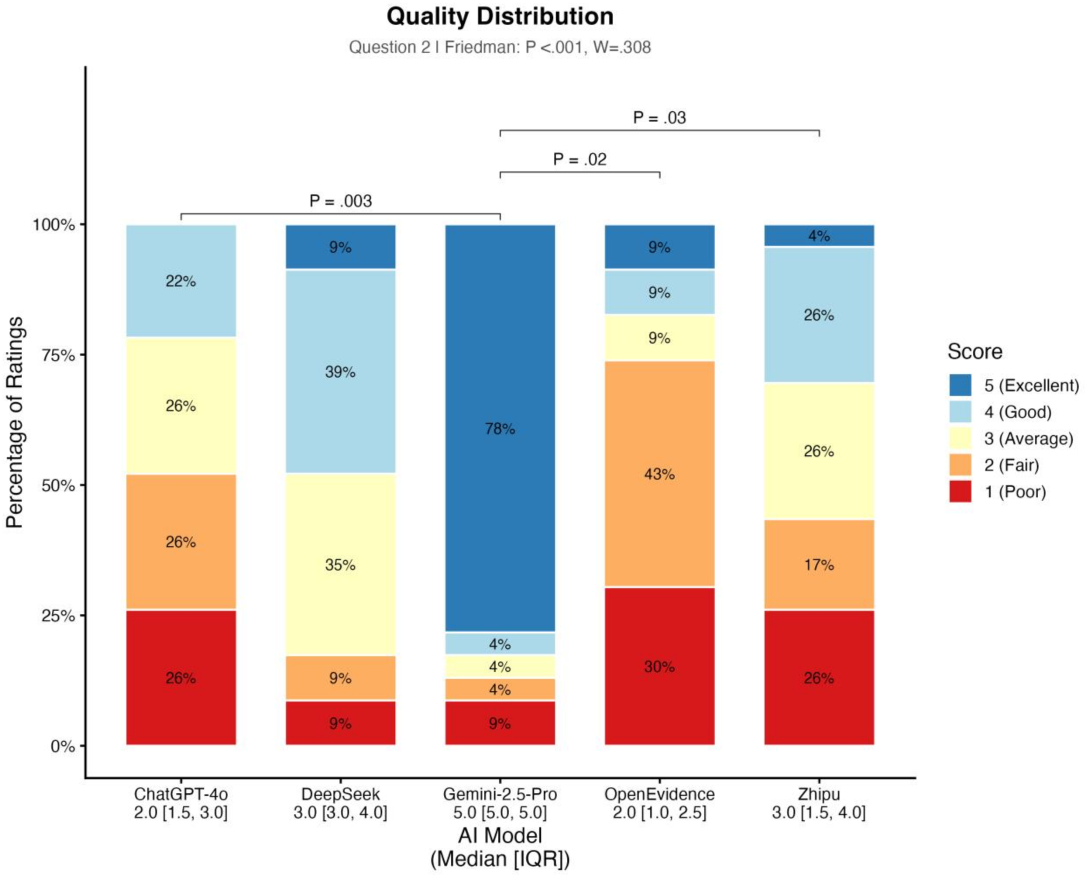
Quality Distribution
Question 2 I Friedman: P <.001, W=.308
P = .03
P = .02
P = .003
100%
75%
50%
25%
0%
Percentage of Ratings
22%
26%
26%
26%
9%
39%
35%
9%
9%
78%
4%
4%
4%
9%
9%
9%
9%
43%
30%
4%
26%
26%
17%
26%
Score
5 (Excellent)
4 (Good)
3 (Average)
2 (Fair)
1 (Poor)
ChatGPT-4o
2.0 [1.5, 3.0]
DeepSeek
3.0 [3.0, 4.0]
Gemini-2.5-Pro
5.0 [5.0, 5.0]
OpenEvidence
2.0 [1.0, 2.5]
Zhipu
3.0 [1.5, 4.0]
AI Model
(Median [IQR])

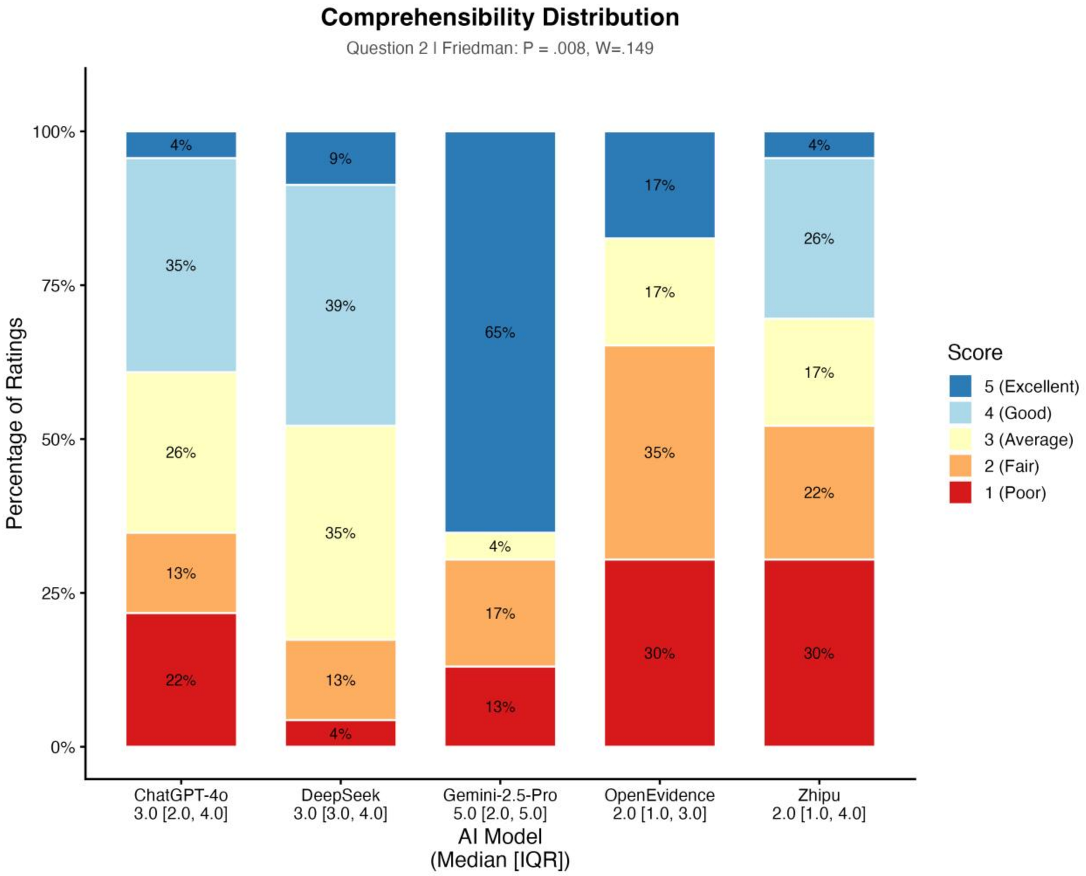
Comprehensibility Distribution
Question 2 I Friedman: P = .008, W=.149
100%
75%
50%
25%
0%
Percentage of Ratings
4%
35%
26%
13%
22%
9%
39%
35%
13%
4%
65%
4%
17%
13%
17%
17%
35%
30%
4%
26%
17%
22%
30%
Score
5 (Excellent)
4 (Good)
3 (Average)
2 (Fair)
1 (Poor)
ChatGPT-4o
3.0 [2.0, 4.0]
DeepSeek
3.0 [3.0, 4.0]
Gemini-2.5-Pro
5.0 [2.0, 5.0]
OpenEvidence
2.0 [1.0, 3.0]
Zhipu
2.0 [1.0, 4.0]
AI Model
(Median [IQR])

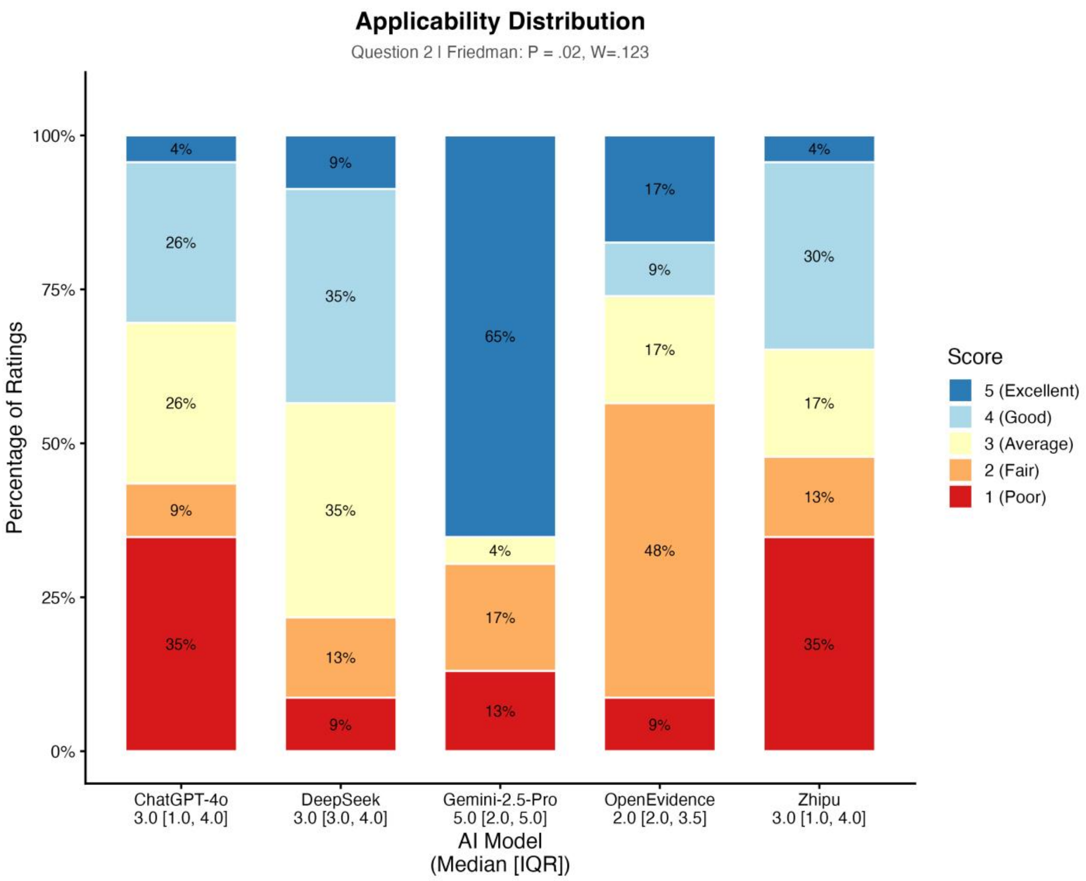
Applicability Distribution
Question 2 | Friedman: P = .02, W=.123
Percentage of Ratings
100%
75%
50%
25%
0%
ChatGPT-4o
3.0 [1.0, 4.0]
DeepSeek
3.0 [3.0, 4.0]
Gemini-2.5-Pro
5.0 [2.0, 5.0]
OpenEvidence
2.0 [2.0, 3.5]
Zhipu
3.0 [1.0, 4.0]
AI Model
(Median [IQR])
Score
5 (Excellent)
4 (Good)
3 (Average)
2 (Fair)
1 (Poor)
ChatGPT-4o: 4%, 26%, 26%, 9%, 35%
DeepSeek: 9%, 35%, 35%, 13%, 9%
Gemini-2.5-Pro: 65%, 4%, 17%, 13%
OpenEvidence: 17%, 9%, 17%, 48%, 9%
Zhipu: 4%, 30%, 17%, 13%, 35%

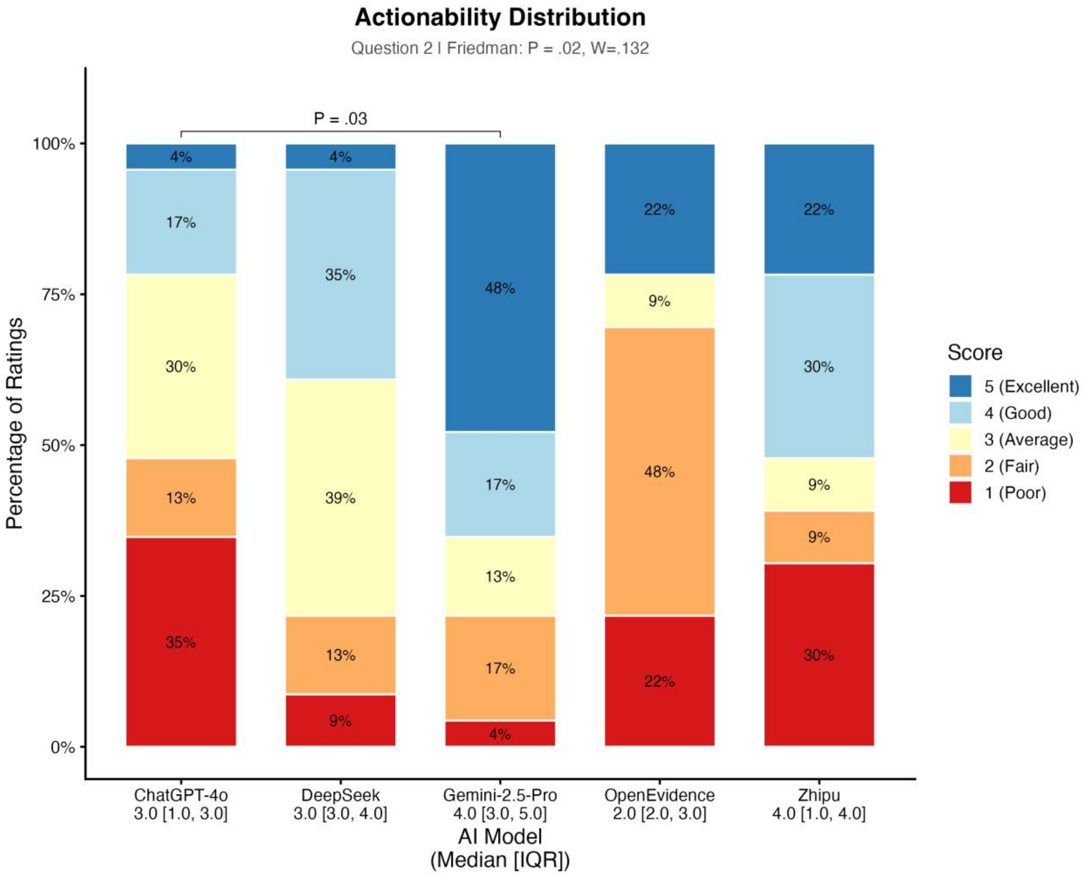
Actionability Distribution
Question 2 | Friedman: P = .02, W=.132
P = .03
Percentage of Ratings
100%
75%
50%
25%
0%
ChatGPT-4o
3.0 [1.0, 3.0]
DeepSeek
3.0 [3.0, 4.0]
Gemini-2.5-Pro
4.0 [3.0, 5.0]
OpenEvidence
2.0 [2.0, 3.0]
Zhipu
4.0 [1.0, 4.0]
AI Model
(Median [IQR])
Score
5 (Excellent)
4 (Good)
3 (Average)
2 (Fair)
1 (Poor)
ChatGPT-4o: 4%, 17%, 30%, 13%, 35%
DeepSeek: 4%, 35%, 39%, 13%, 9%
Gemini-2.5-Pro: 48%, 17%, 13%, 17%, 4%
OpenEvidence: 22%, 9%, 48%, 22%
Zhipu: 22%, 30%, 9%, 9%, 30%

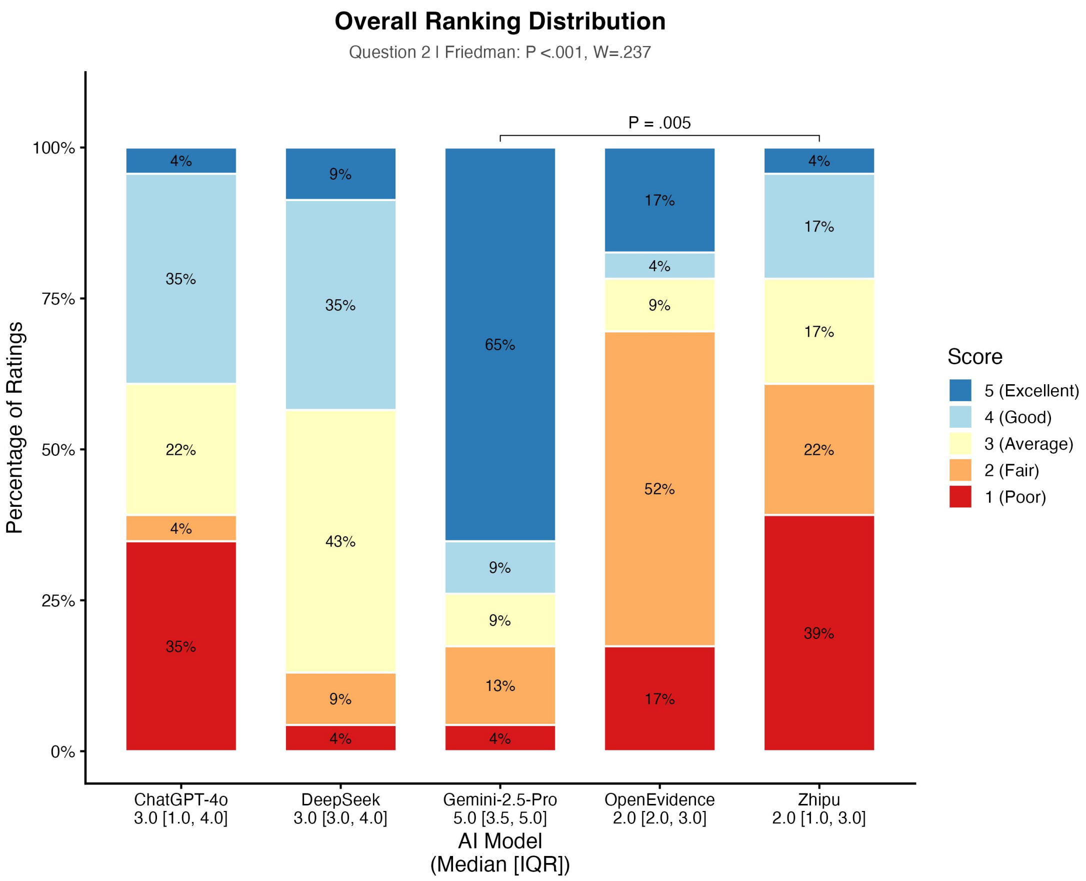
Overall Ranking Distribution
Question 2 I Friedman: P <.001, W=.237
P = .005
Percentage of Ratings
100%
75%
50%
25%
0%
4%
35%
22%
4%
35%
9%
35%
43%
9%
4%
65%
9%
9%
13%
4%
17%
4%
9%
52%
17%
4%
17%
17%
22%
39%
Score
5 (Excellent)
4 (Good)
3 (Average)
2 (Fair)
1 (Poor)
ChatGPT-4o
3.0 [1.0, 4.0]
DeepSeek
3.0 [3.0, 4.0]
Gemini-2.5-Pro
5.0 [3.5, 5.0]
OpenEvidence
2.0 [2.0, 3.0]
Zhipu
2.0 [1.0, 3.0]
AI Model
(Median [IQR])

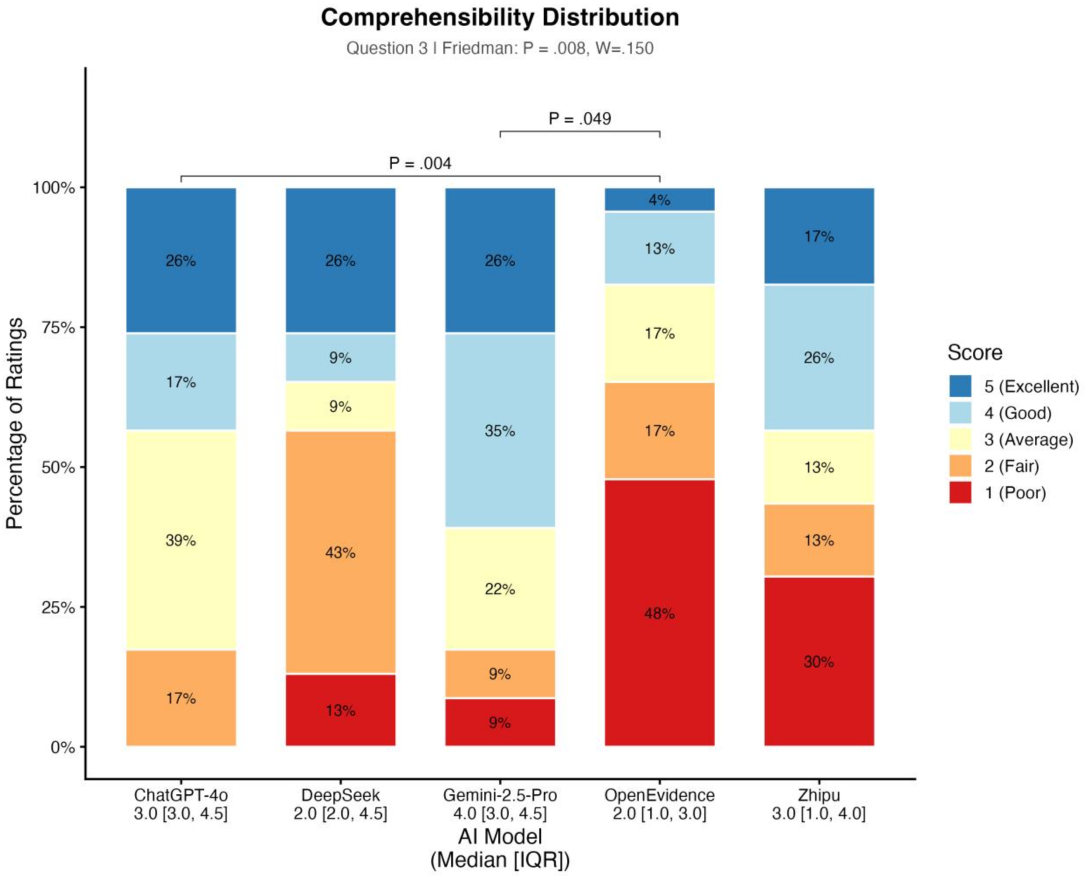
Comprehensibility Distribution
Question 3 I Friedman: P = .008, W=.150
P = .049
P = .004
Percentage of Ratings
100%
75%
50%
25%
0%
26%
17%
39%
17%
26%
9%
9%
43%
13%
26%
35%
22%
9%
9%
4%
13%
17%
17%
48%
17%
26%
13%
13%
30%
Score
5 (Excellent)
4 (Good)
3 (Average)
2 (Fair)
1 (Poor)
ChatGPT-4o
3.0 [3.0, 4.5]
DeepSeek
2.0 [2.0, 4.5]
Gemini-2.5-Pro
4.0 [3.0, 4.5]
OpenEvidence
2.0 [1.0, 3.0]
Zhipu
3.0 [1.0, 4.0]
AI Model
(Median [IQR])

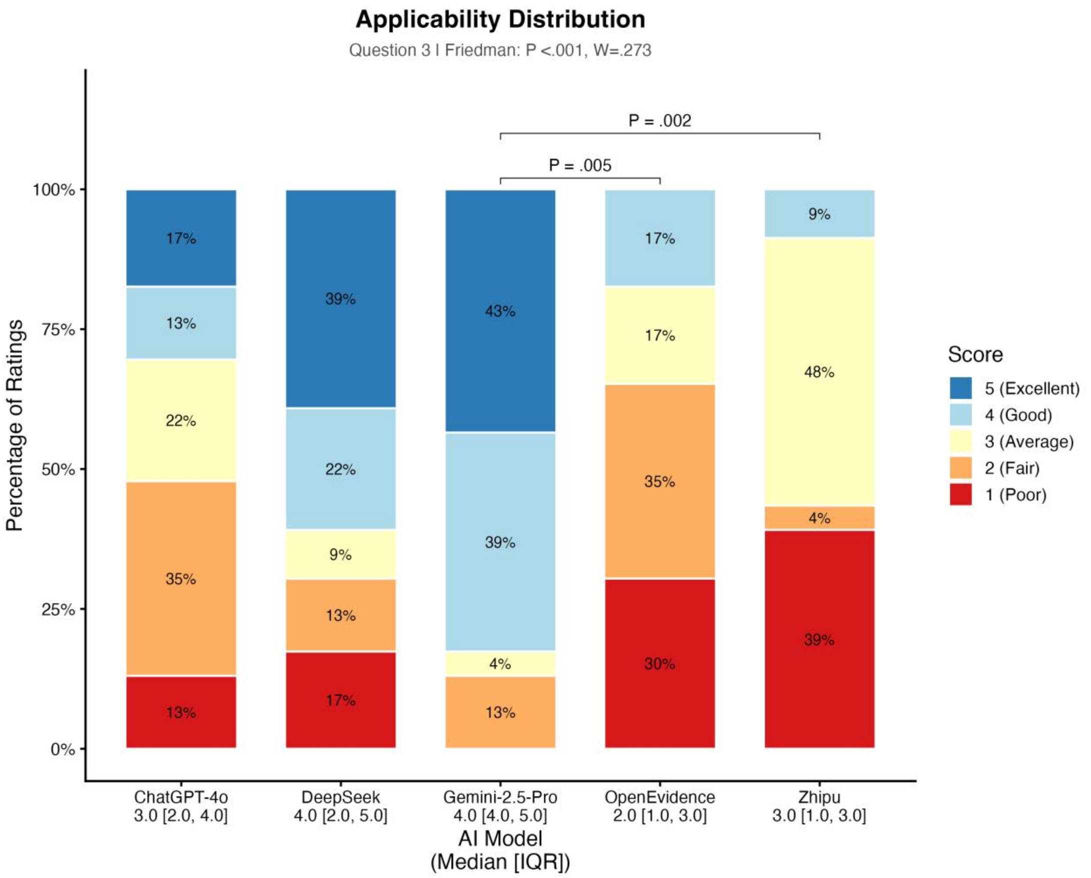
Applicability Distribution
Question 3 I Friedman: P <.001, W=.273
Percentage of Ratings
100%
75%
50%
25%
0%
P = .002
P = .005
ChatGPT-4o 3.0 [2.0, 4.0]: 17%, 13%, 22%, 35%, 13%
DeepSeek 4.0 [2.0, 5.0]: 39%, 22%, 9%, 13%, 17%
Gemini-2.5-Pro 4.0 [4.0, 5.0]: 43%, 39%, 4%, 13%
OpenEvidence 2.0 [1.0, 3.0]: 17%, 17%, 35%, 30%
Zhipu 3.0 [1.0, 3.0]: 9%, 48%, 4%, 39%
AI Model
(Median [IQR])
Score
5 (Excellent)
4 (Good)
3 (Average)
2 (Fair)
1 (Poor)

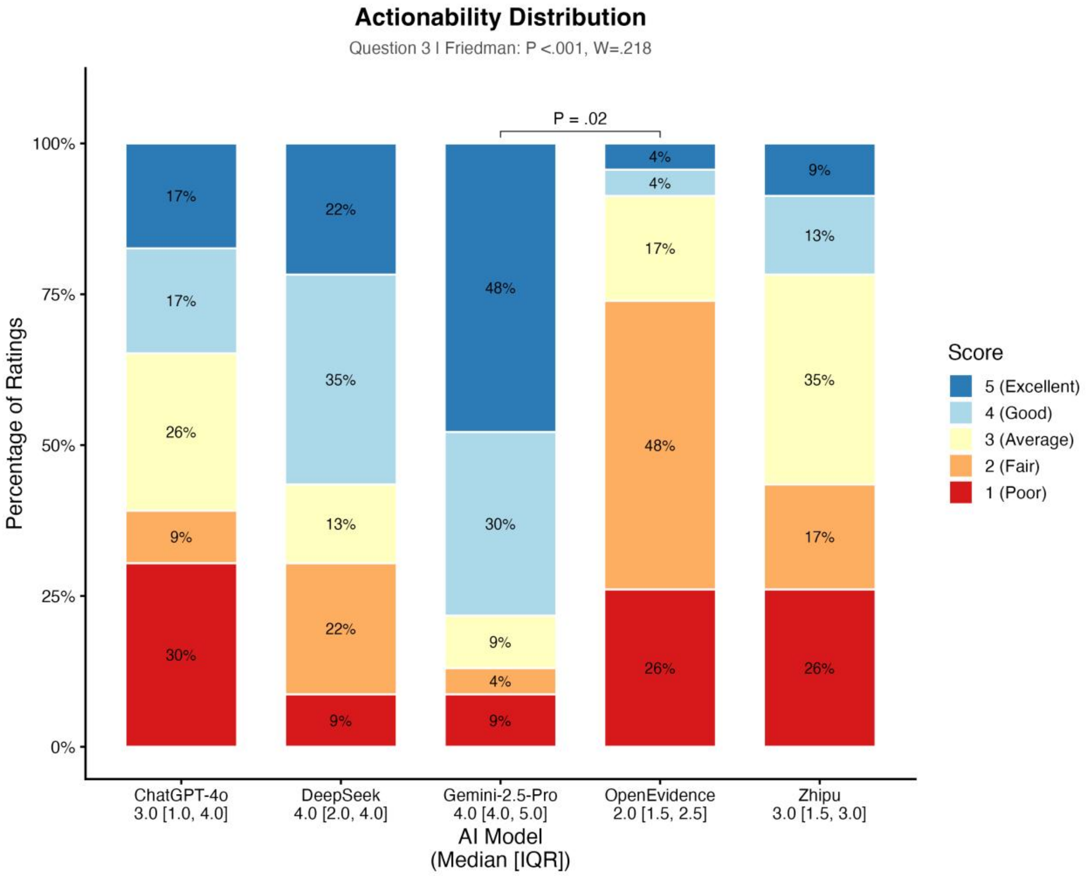
Actionability Distribution
Question 3 I Friedman: P <.001, W=.218
Percentage of Ratings
100%
75%
50%
25%
0%
P = .02
ChatGPT-4o 3.0 [1.0, 4.0]: 17%, 17%, 26%, 9%, 30%
DeepSeek 4.0 [2.0, 4.0]: 22%, 35%, 13%, 22%, 9%
Gemini-2.5-Pro 4.0 [4.0, 5.0]: 48%, 30%, 9%, 4%, 9%
OpenEvidence 2.0 [1.5, 2.5]: 4%, 4%, 17%, 48%, 26%
Zhipu 3.0 [1.5, 3.0]: 9%, 13%, 35%, 17%, 26%
AI Model
(Median [IQR])
Score
5 (Excellent)
4 (Good)
3 (Average)
2 (Fair)
1 (Poor)

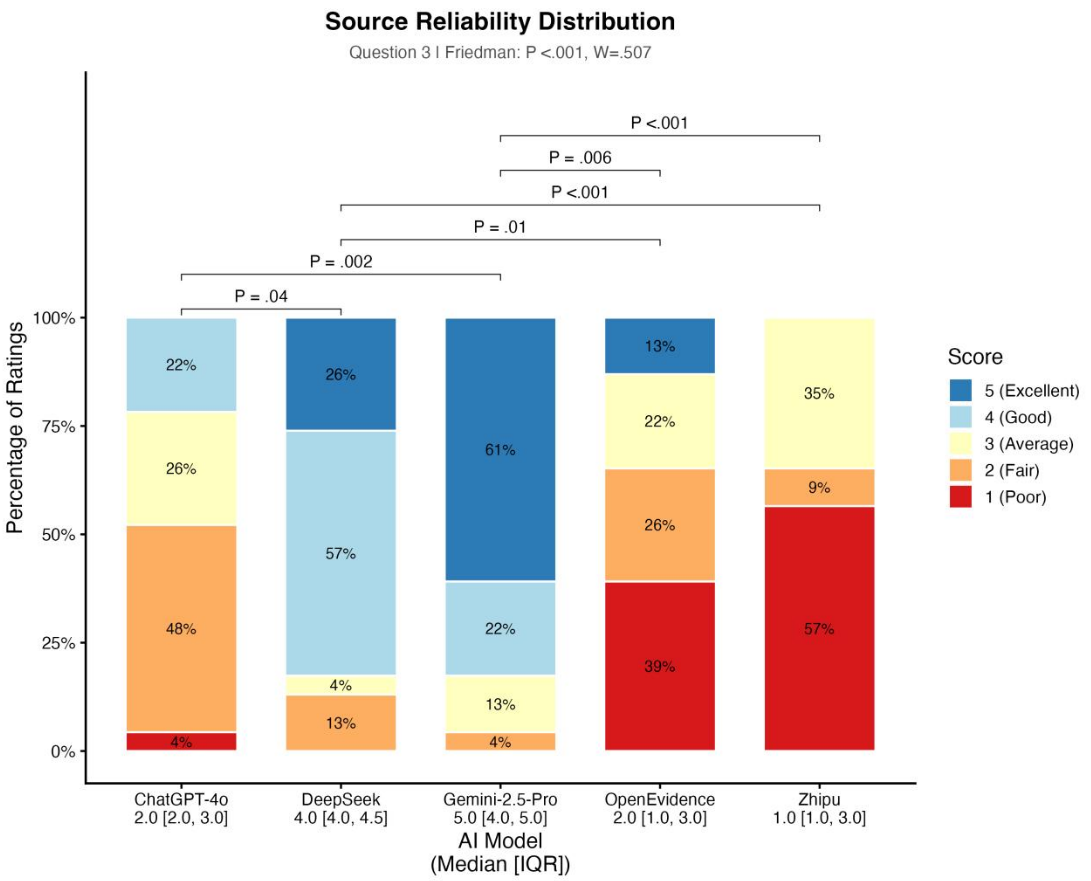
Source Reliability Distribution
Question 3 | Friedman: P <.001, W=.507
P <.001
P = .006
P <.001
P = .01
P = .002
P = .04
Percentage of Ratings
100%
75%
50%
25%
0%
22%
26%
48%
4%
26%
57%
4%
13%
61%
22%
13%
4%
13%
22%
26%
39%
35%
9%
57%
ChatGPT-4o
2.0 [2.0, 3.0]
DeepSeek
4.0 [4.0, 4.5]
Gemini-2.5-Pro
5.0 [4.0, 5.0]
OpenEvidence
2.0 [1.0, 3.0]
Zhipu
1.0 [1.0, 3.0]
AI Model
(Median [IQR])
Score
5 (Excellent)
4 (Good)
3 (Average)
2 (Fair)
1 (Poor)

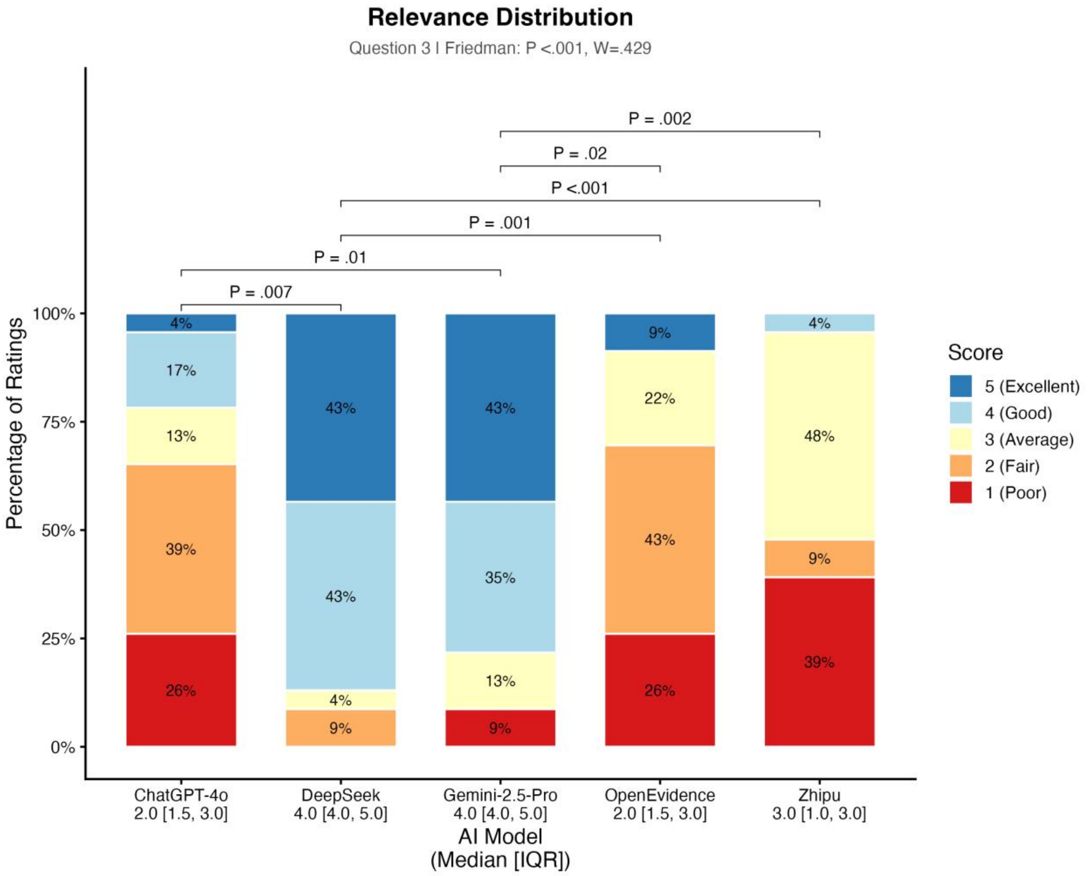
Relevance Distribution
Question 3 | Friedman: P <.001, W=.429
P = .002
P = .02
P <.001
P = .001
P = .01
P = .007
Percentage of Ratings
100%
75%
50%
25%
0%
4%
17%
13%
39%
26%
43%
43%
4%
9%
43%
35%
13%
9%
9%
22%
43%
26%
4%
48%
9%
39%
ChatGPT-4o
2.0 [1.5, 3.0]
DeepSeek
4.0 [4.0, 5.0]
Gemini-2.5-Pro
4.0 [4.0, 5.0]
OpenEvidence
2.0 [1.5, 3.0]
Zhipu
3.0 [1.0, 3.0]
AI Model
(Median [IQR])
Score
5 (Excellent)
4 (Good)
3 (Average)
2 (Fair)
1 (Poor)

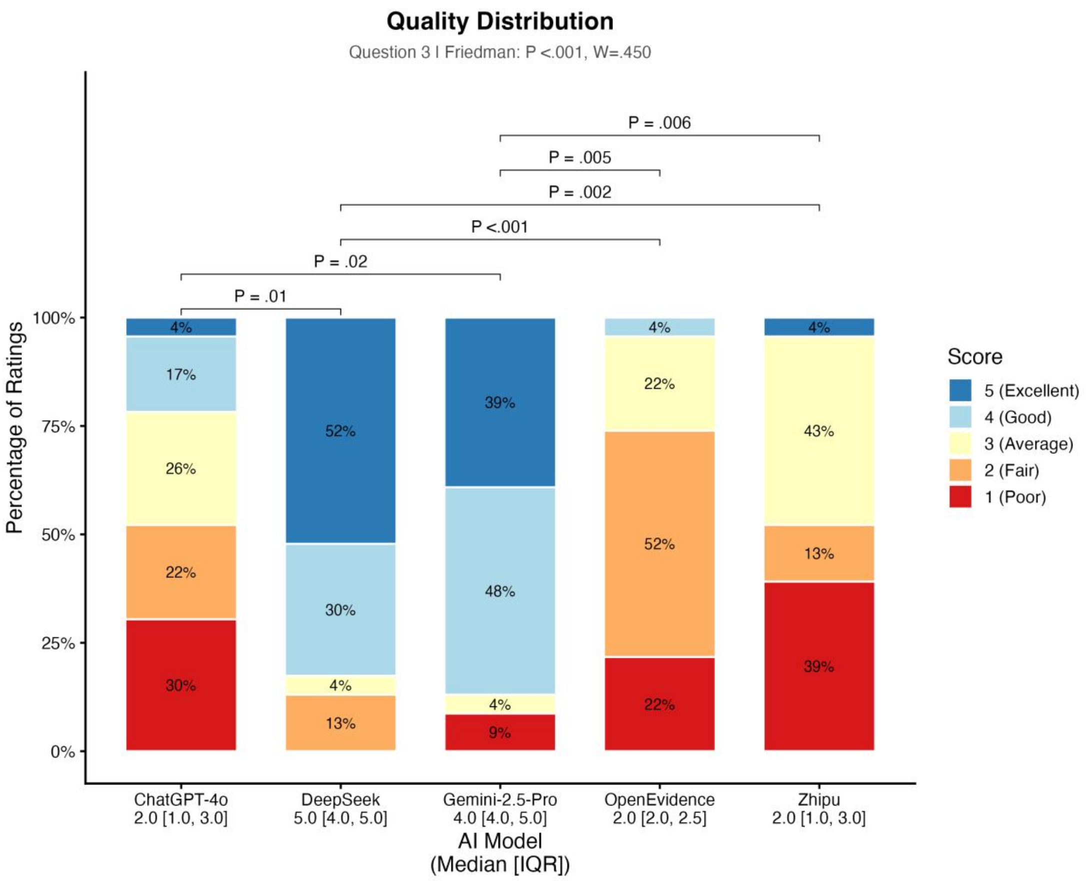
Quality Distribution
Question 3 | Friedman: P <.001, W=.450
P = .006
P = .005
P = .002
P <.001
P = .02
P = .01
100%
75%
50%
25%
0%
Percentage of Ratings
4%
17%
26%
22%
30%
52%
30%
4%
13%
39%
48%
4%
9%
4%
22%
52%
22%
4%
43%
13%
39%
ChatGPT-4o
2.0 [1.0, 3.0]
DeepSeek
5.0 [4.0, 5.0]
Gemini-2.5-Pro
4.0 [4.0, 5.0]
OpenEvidence
2.0 [2.0, 2.5]
Zhipu
2.0 [1.0, 3.0]
AI Model
(Median [IQR])
Score
5 (Excellent)
4 (Good)
3 (Average)
2 (Fair)
1 (Poor)

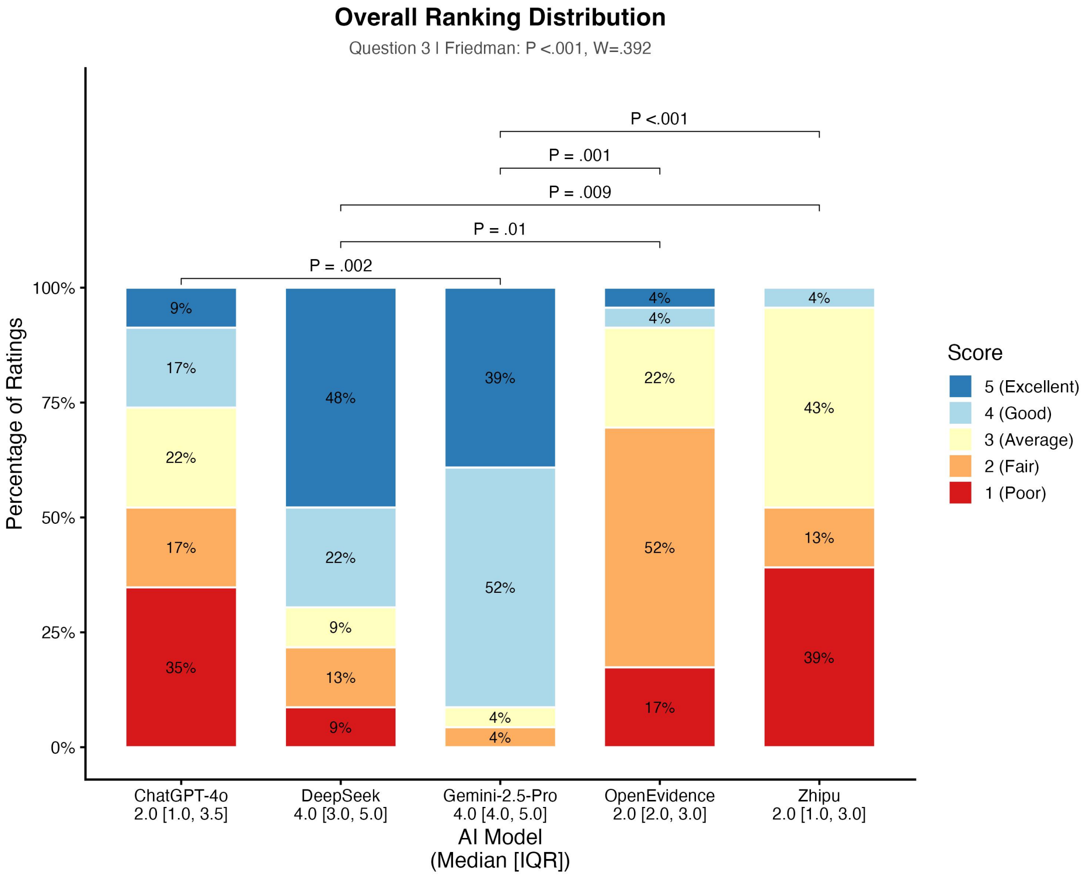
Overall Ranking Distribution
Question 3 | Friedman: P <.001, W=.392
P <.001
P = .001
P = .009
P = .01
P = .002
100%
75%
50%
25%
0%
Percentage of Ratings
9%
17%
22%
17%
35%
48%
22%
9%
13%
9%
39%
52%
4%
4%
4%
4%
22%
52%
17%
4%
43%
13%
39%
ChatGPT-4o
2.0 [1.0, 3.5]
DeepSeek
4.0 [3.0, 5.0]
Gemini-2.5-Pro
4.0 [4.0, 5.0]
OpenEvidence
2.0 [2.0, 3.0]
Zhipu
2.0 [1.0, 3.0]
AI Model
(Median [IQR])
Score
5 (Excellent)
4 (Good)
3 (Average)
2 (Fair)
1 (Poor)

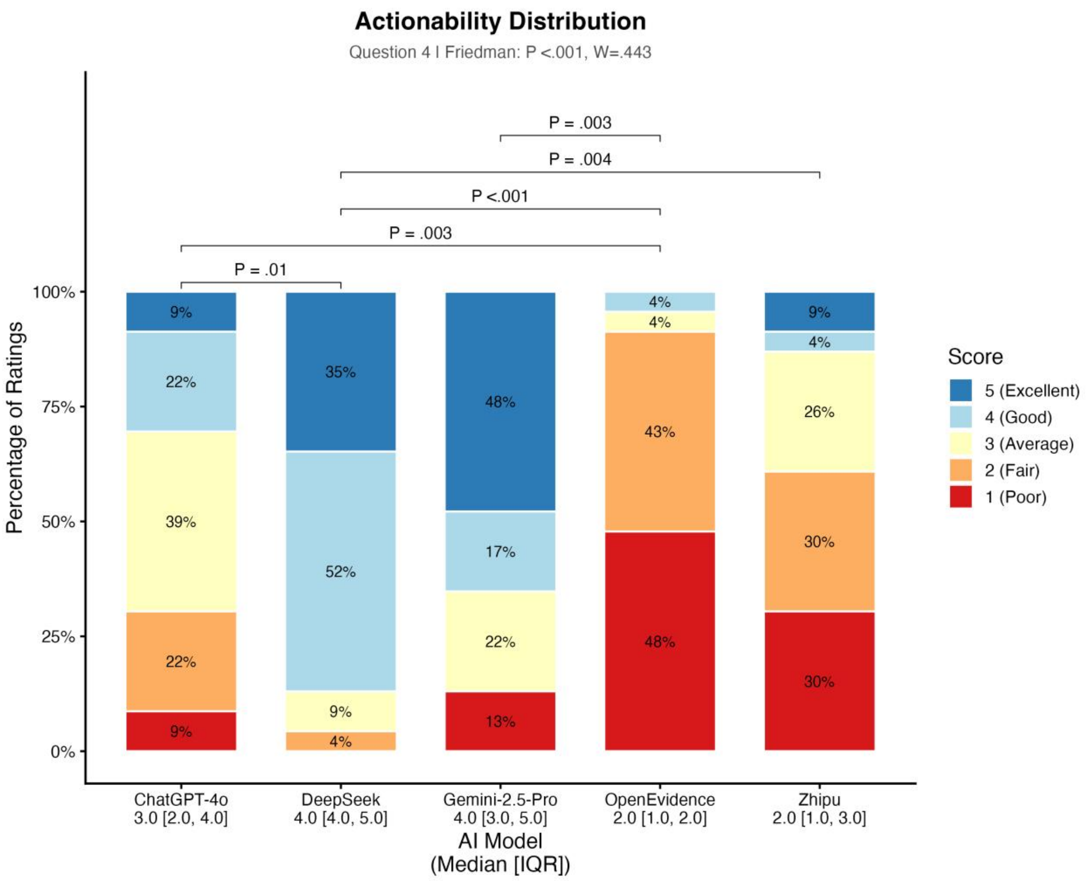
Actionability Distribution
Question 4 | Friedman: P <.001, W=.443
P = .003
P = .004
P <.001
P = .003
P = .01
Percentage of Ratings
100%
75%
50%
25%
0%
9%
22%
39%
22%
9%
35%
52%
9%
4%
48%
17%
22%
13%
4%
4%
43%
48%
9%
4%
26%
30%
30%
Score
5 (Excellent)
4 (Good)
3 (Average)
2 (Fair)
1 (Poor)
ChatGPT-4o
3.0 [2.0, 4.0]
DeepSeek
4.0 [4.0, 5.0]
Gemini-2.5-Pro
4.0 [3.0, 5.0]
OpenEvidence
2.0 [1.0, 2.0]
Zhipu
2.0 [1.0, 3.0]
AI Model
(Median [IQR])

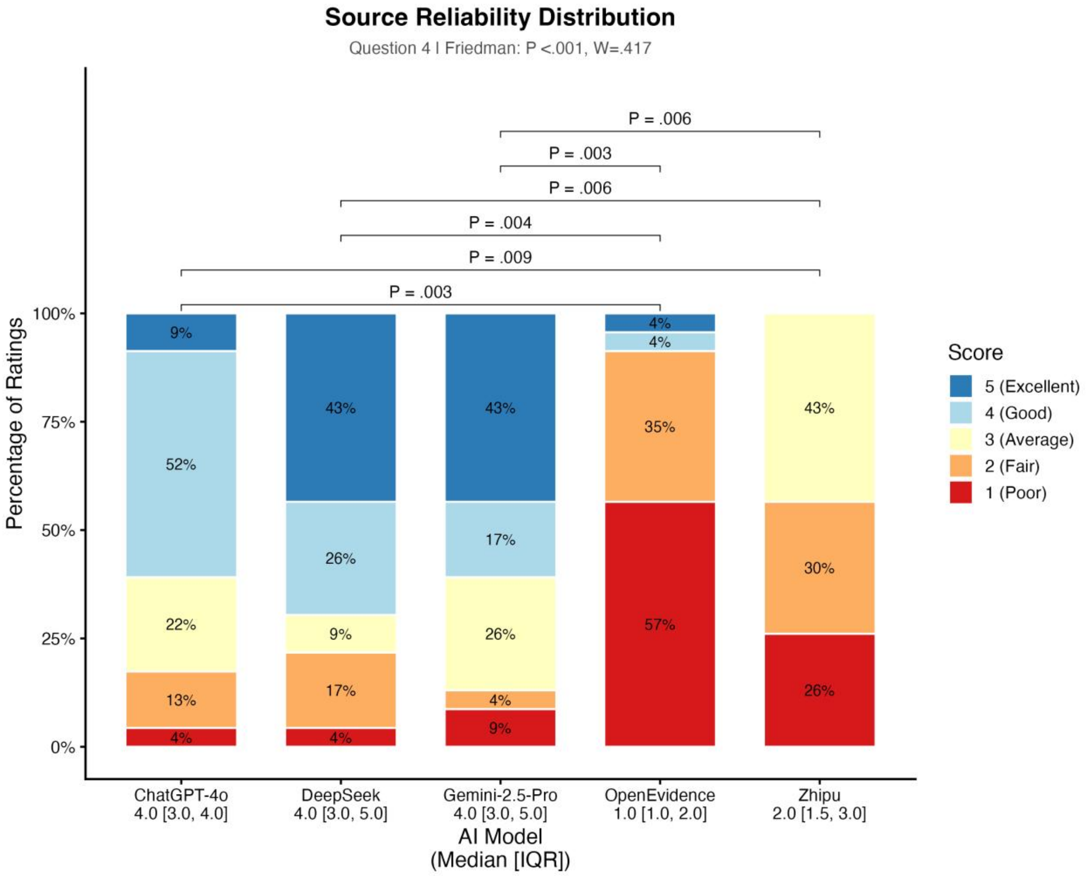
Source Reliability Distribution
Question 4 | Friedman: P <.001, W=.417
P = .006
P = .003
P = .006
P = .004
P = .009
P = .003
Percentage of Ratings
100%
75%
50%
25%
0%
9%
52%
22%
13%
4%
43%
26%
9%
17%
4%
43%
17%
26%
4%
9%
4%
4%
35%
57%
43%
30%
26%
Score
5 (Excellent)
4 (Good)
3 (Average)
2 (Fair)
1 (Poor)
ChatGPT-4o
4.0 [3.0, 4.0]
DeepSeek
4.0 [3.0, 5.0]
Gemini-2.5-Pro
4.0 [3.0, 5.0]
OpenEvidence
1.0 [1.0, 2.0]
Zhipu
2.0 [1.5, 3.0]
AI Model
(Median [IQR])

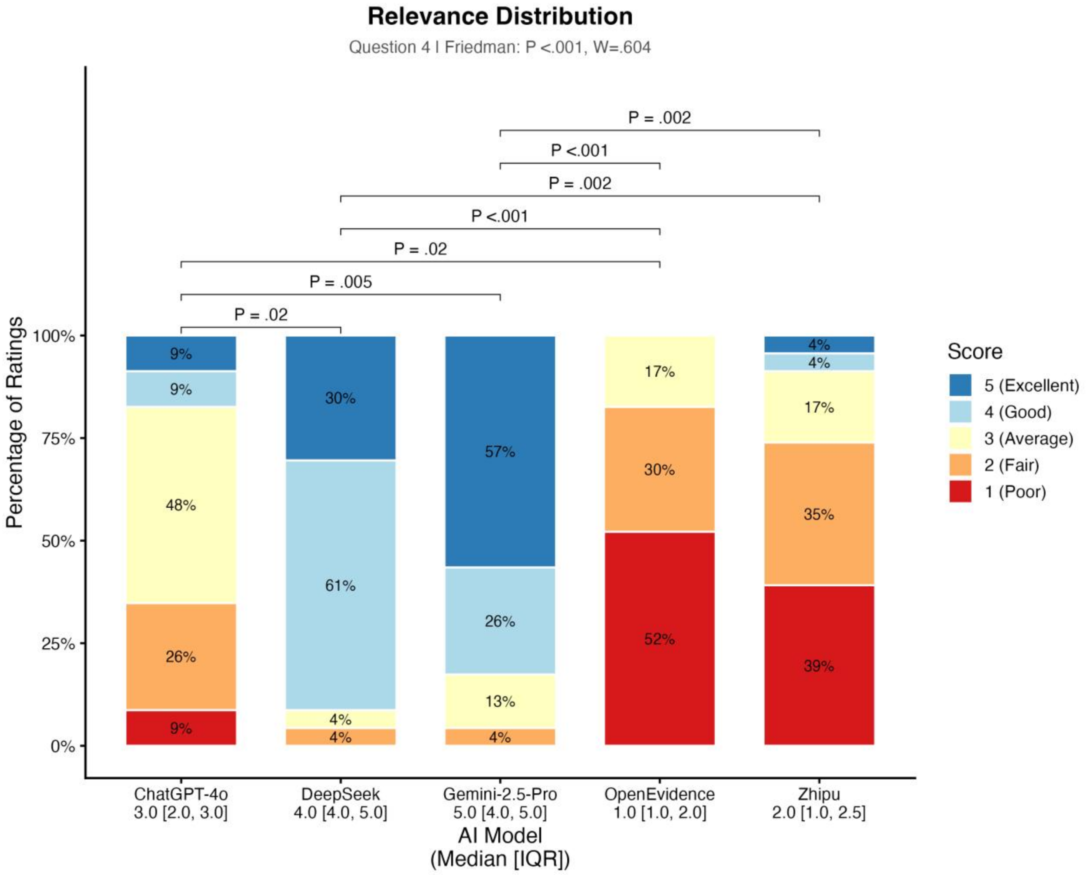
Relevance Distribution
Question 4 | Friedman: P <.001, W=.604
P = .002
P <.001
P = .002
P <.001
P = .02
P = .005
P = .02
Percentage of Ratings
100%
75%
50%
25%
0%
9%
9%
48%
26%
9%
30%
61%
4%
4%
57%
26%
13%
4%
17%
30%
52%
4%
4%
17%
35%
39%
ChatGPT-4o
3.0 [2.0, 3.0]
DeepSeek
4.0 [4.0, 5.0]
Gemini-2.5-Pro
5.0 [4.0, 5.0]
OpenEvidence
1.0 [1.0, 2.0]
Zhipu
2.0 [1.0, 2.5]
AI Model
(Median [IQR])
Score
5 (Excellent)
4 (Good)
3 (Average)
2 (Fair)
1 (Poor)

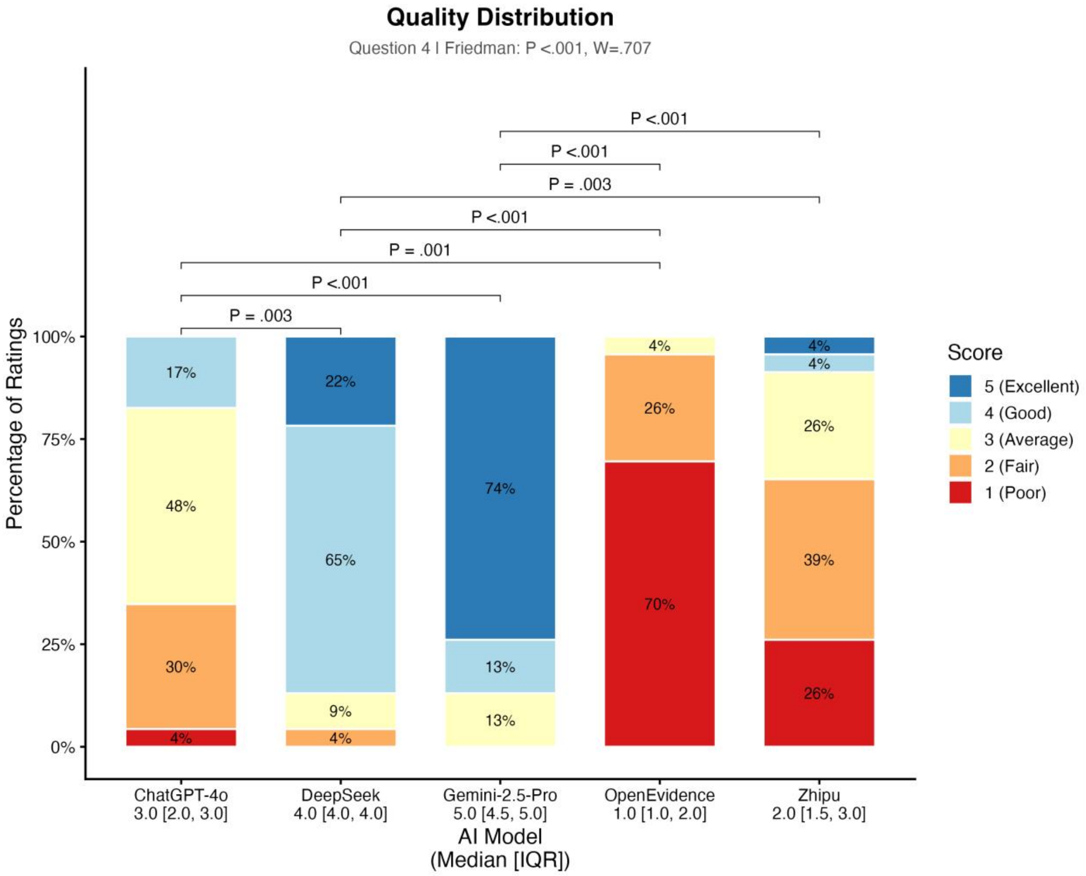
Quality Distribution
Question 4 | Friedman: P <.001, W=.707
P <.001
P <.001
P = .003
P <.001
P = .001
P <.001
P = .003
Percentage of Ratings
100%
75%
50%
25%
0%
17%
48%
30%
4%
22%
65%
9%
4%
74%
13%
13%
4%
26%
70%
4%
4%
26%
39%
26%
ChatGPT-4o
3.0 [2.0, 3.0]
DeepSeek
4.0 [4.0, 4.0]
Gemini-2.5-Pro
5.0 [4.5, 5.0]
OpenEvidence
1.0 [1.0, 2.0]
Zhipu
2.0 [1.5, 3.0]
AI Model
(Median [IQR])
Score
5 (Excellent)
4 (Good)
3 (Average)
2 (Fair)
1 (Poor)

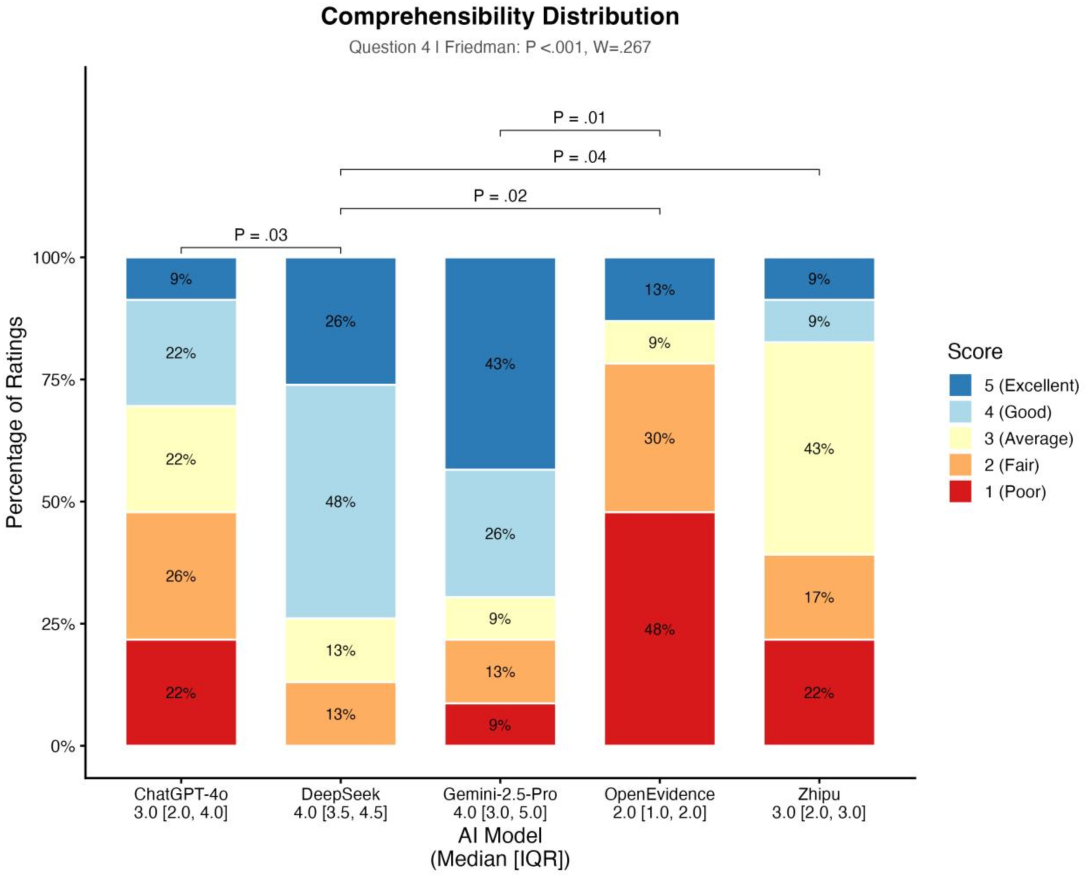
Comprehensibility Distribution
Question 4 | Friedman: P <.001, W=.267
P = .01
P = .04
P = .02
P = .03
Percentage of Ratings
100%
75%
50%
25%
0%
9%
22%
22%
26%
22%
26%
48%
13%
13%
43%
26%
9%
13%
9%
13%
9%
30%
48%
9%
9%
43%
17%
22%
ChatGPT-4o
3.0 [2.0, 4.0]
DeepSeek
4.0 [3.5, 4.5]
Gemini-2.5-Pro
4.0 [3.0, 5.0]
OpenEvidence
2.0 [1.0, 2.0]
Zhipu
3.0 [2.0, 3.0]
AI Model
(Median [IQR])
Score
5 (Excellent)
4 (Good)
3 (Average)
2 (Fair)
1 (Poor)

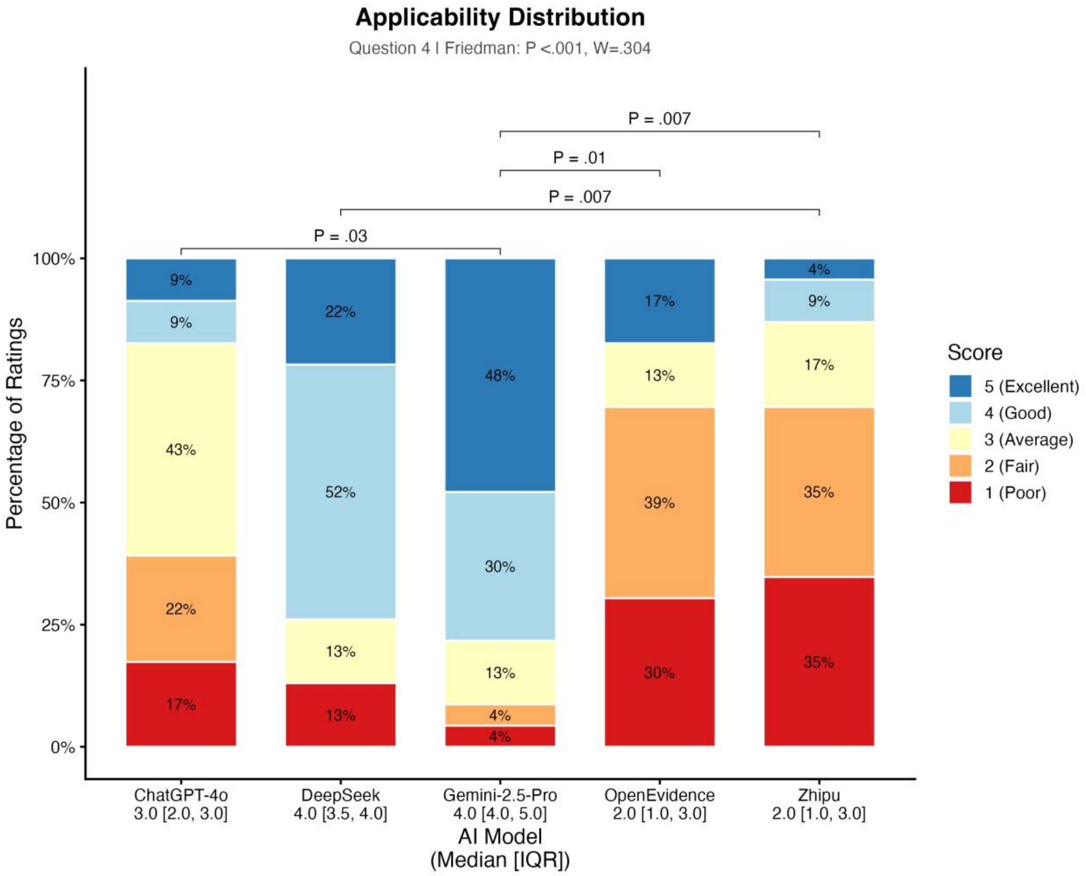
Applicability Distribution
Question 4 | Friedman: P <.001, W=.304
P = .007
P = .01
P = .007
P = .03
Percentage of Ratings
100%
75%
50%
25%
0%
9%
9%
43%
22%
17%
22%
52%
13%
13%
48%
30%
13%
4%
4%
17%
13%
39%
30%
4%
9%
17%
35%
35%
ChatGPT-4o
3.0 [2.0, 3.0]
DeepSeek
4.0 [3.5, 4.0]
Gemini-2.5-Pro
4.0 [4.0, 5.0]
OpenEvidence
2.0 [1.0, 3.0]
Zhipu
2.0 [1.0, 3.0]
AI Model
(Median [IQR])
Score
5 (Excellent)
4 (Good)
3 (Average)
2 (Fair)
1 (Poor)

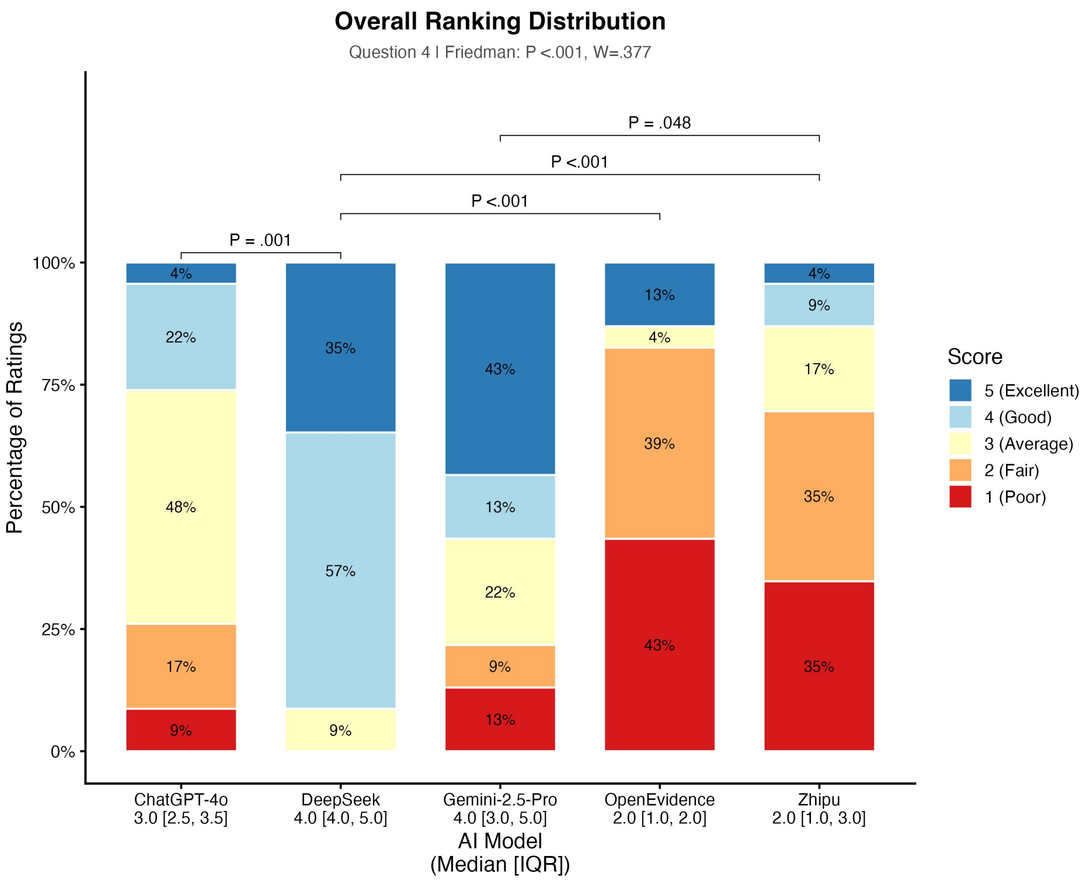
Overall Ranking Distribution
Question 4 I Friedman: P <.001, W=.377
P = .048
P <.001
P <.001
P = .001
100%
75%
50%
25%
0%
Percentage of Ratings
4%
22%
48%
17%
9%
35%
57%
9%
43%
13%
22%
9%
13%
13%
4%
39%
43%
4%
9%
17%
35%
35%
ChatGPT-4o
3.0 [2.5, 3.5]
DeepSeek
4.0 [4.0, 5.0]
Gemini-2.5-Pro
4.0 [3.0, 5.0]
OpenEvidence
2.0 [1.0, 2.0]
Zhipu
2.0 [1.0, 3.0]
AI Model
(Median [IQR])
Score
5 (Excellent)
4 (Good)
3 (Average)
2 (Fair)
1 (Poor)

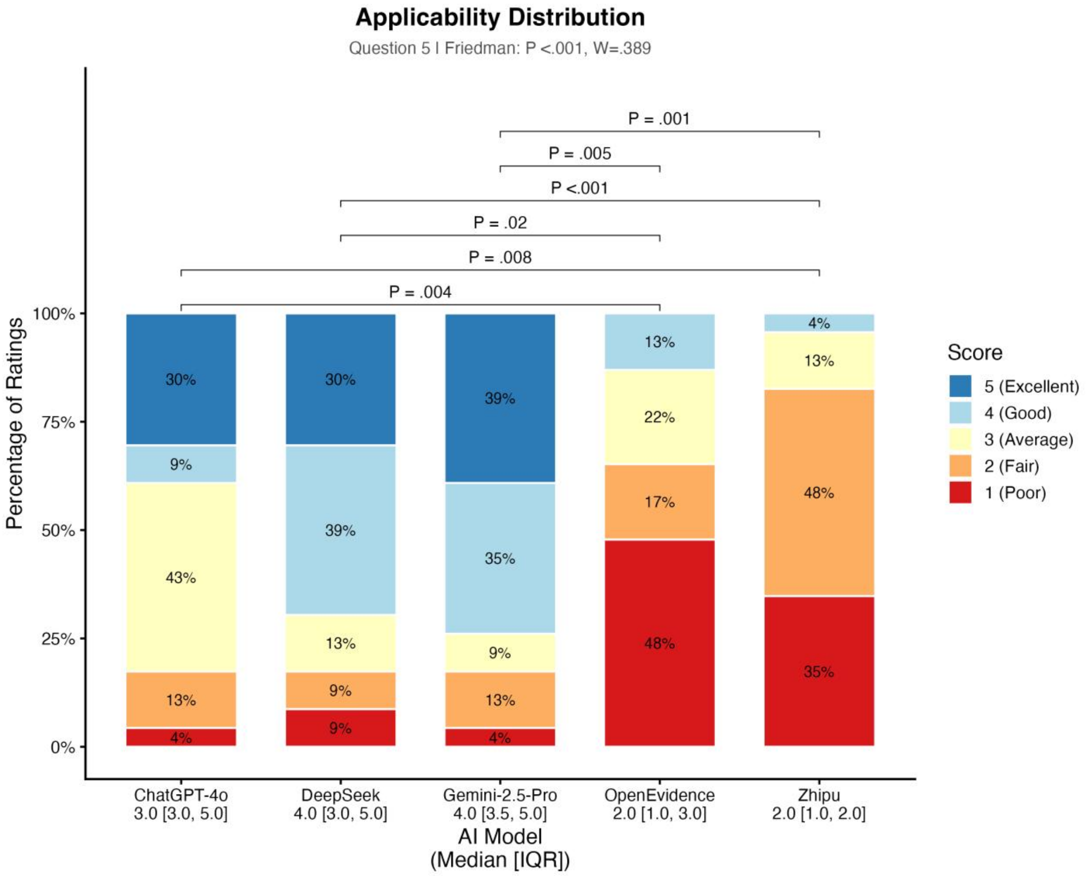
Applicability Distribution
Question 5 I Friedman: P <.001, W=.389
P = .001
P = .005
P <.001
P = .02
P = .008
P = .004
100%
75%
50%
25%
0%
Percentage of Ratings
30%
9%
43%
13%
4%
30%
39%
13%
9%
9%
39%
35%
9%
13%
4%
13%
22%
17%
48%
4%
13%
48%
35%
ChatGPT-4o
3.0 [3.0, 5.0]
DeepSeek
4.0 [3.0, 5.0]
Gemini-2.5-Pro
4.0 [3.5, 5.0]
OpenEvidence
2.0 [1.0, 3.0]
Zhipu
2.0 [1.0, 2.0]
AI Model
(Median [IQR])
Score
5 (Excellent)
4 (Good)
3 (Average)
2 (Fair)
1 (Poor)

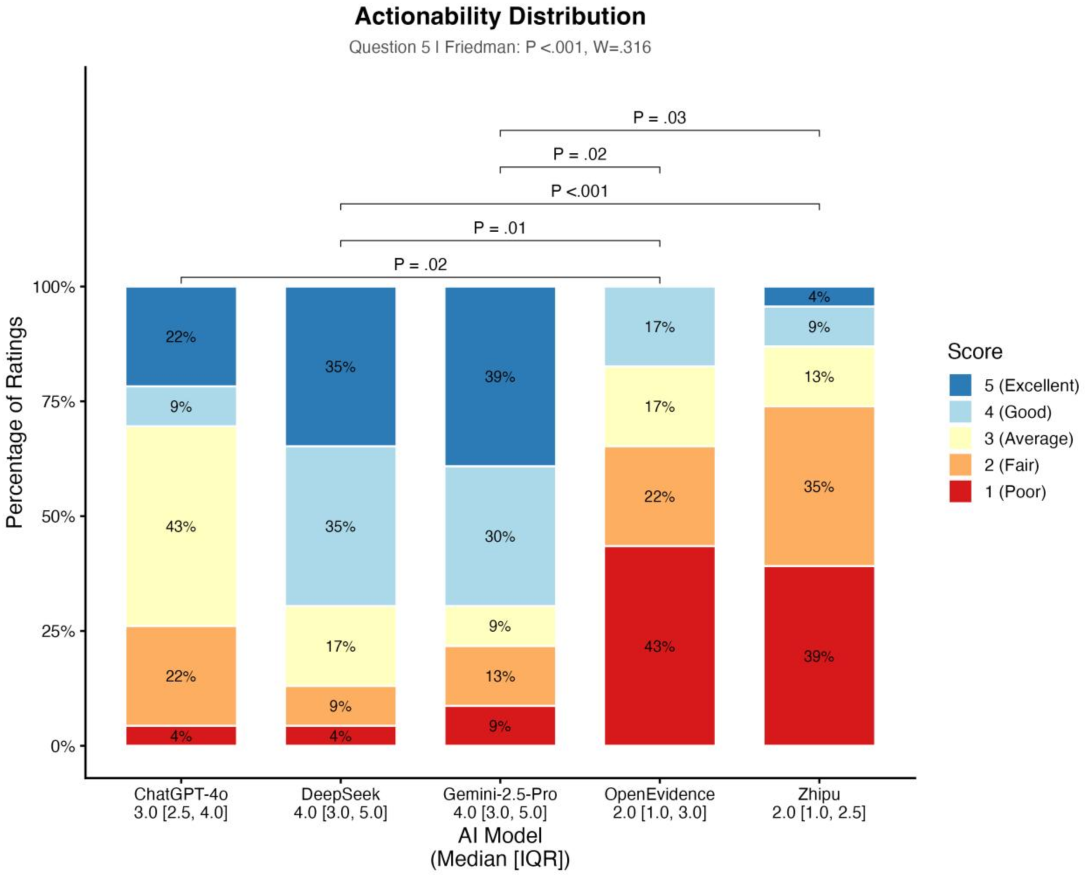

Actionability Distribution
Question 5 | Friedman: P <.001, W=.316
P = .03
P = .02
P <.001
P = .01
P = .02
Percentage of Ratings
100%
75%
50%
25%
0%
22%
9%
43%
22%
4%
35%
35%
17%
9%
4%
39%
30%
9%
13%
9%
17%
17%
22%
43%
4%
9%
13%
35%
39%
Score
5 (Excellent)
4 (Good)
3 (Average)
2 (Fair)
1 (Poor)
ChatGPT-4o
3.0 [2.5, 4.0]
DeepSeek
4.0 [3.0, 5.0]
Gemini-2.5-Pro
4.0 [3.0, 5.0]
OpenEvidence
2.0 [1.0, 3.0]
Zhipu
2.0 [1.0, 2.5]
AI Model
(Median [IQR])

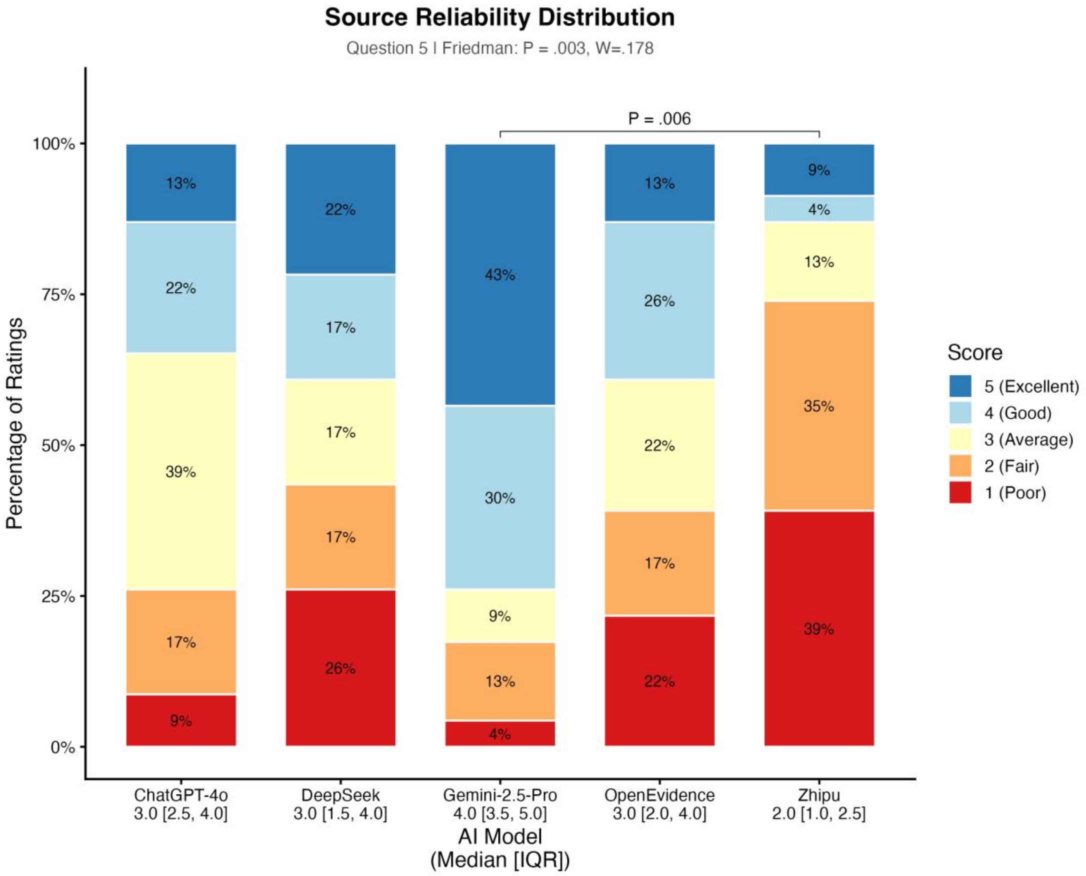

Source Reliability Distribution
Question 5 | Friedman: P = .003, W=.178
P = .006
Percentage of Ratings
100%
75%
50%
25%
0%
13%
22%
39%
17%
9%
22%
17%
17%
17%
26%
43%
30%
9%
13%
4%
13%
26%
22%
17%
22%
9%
4%
13%
35%
39%
Score
5 (Excellent)
4 (Good)
3 (Average)
2 (Fair)
1 (Poor)
ChatGPT-4o
3.0 [2.5, 4.0]
DeepSeek
3.0 [1.5, 4.0]
Gemini-2.5-Pro
4.0 [3.5, 5.0]
OpenEvidence
3.0 [2.0, 4.0]
Zhipu
2.0 [1.0, 2.5]
AI Model
(Median [IQR])

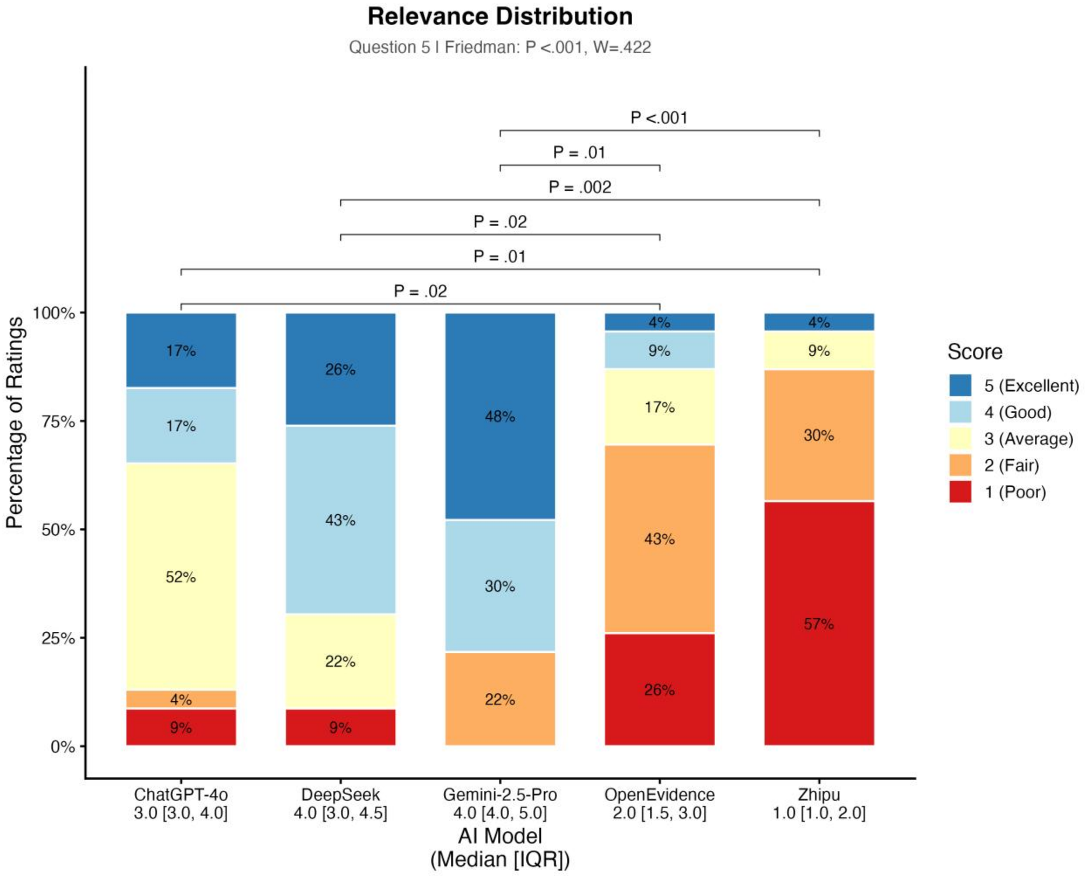
Relevance Distribution
Question 5 | Friedman: P <.001, W=.422
P <.001
P = .01
P = .002
P = .02
P = .01
P = .02
Percentage of Ratings
100%
75%
50%
25%
0%
17%
17%
52%
4%
9%
26%
43%
22%
9%
48%
30%
22%
4%
9%
17%
43%
26%
4%
9%
30%
57%
ChatGPT-4o
3.0 [3.0, 4.0]
DeepSeek
4.0 [3.0, 4.5]
Gemini-2.5-Pro
4.0 [4.0, 5.0]
OpenEvidence
2.0 [1.5, 3.0]
Zhipu
1.0 [1.0, 2.0]
AI Model
(Median [IQR])
Score
5 (Excellent)
4 (Good)
3 (Average)
2 (Fair)
1 (Poor)

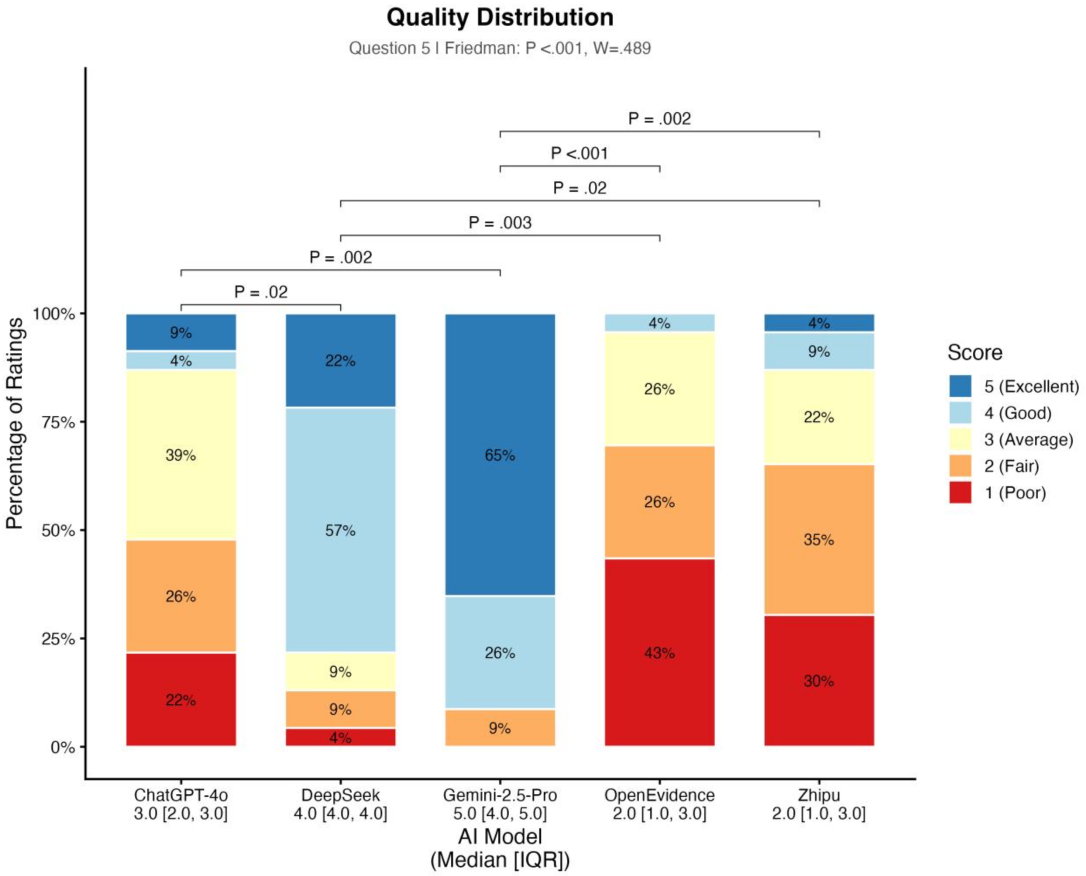
Quality Distribution
Question 5 | Friedman: P <.001, W=.489
P = .002
P <.001
P = .02
P = .003
P = .002
P = .02
Percentage of Ratings
100%
75%
50%
25%
0%
9%
4%
39%
26%
22%
22%
57%
9%
9%
4%
65%
26%
9%
4%
26%
26%
43%
4%
9%
22%
35%
30%
ChatGPT-4o
3.0 [2.0, 3.0]
DeepSeek
4.0 [4.0, 4.0]
Gemini-2.5-Pro
5.0 [4.0, 5.0]
OpenEvidence
2.0 [1.0, 3.0]
Zhipu
2.0 [1.0, 3.0]
AI Model
(Median [IQR])
Score
5 (Excellent)
4 (Good)
3 (Average)
2 (Fair)
1 (Poor)

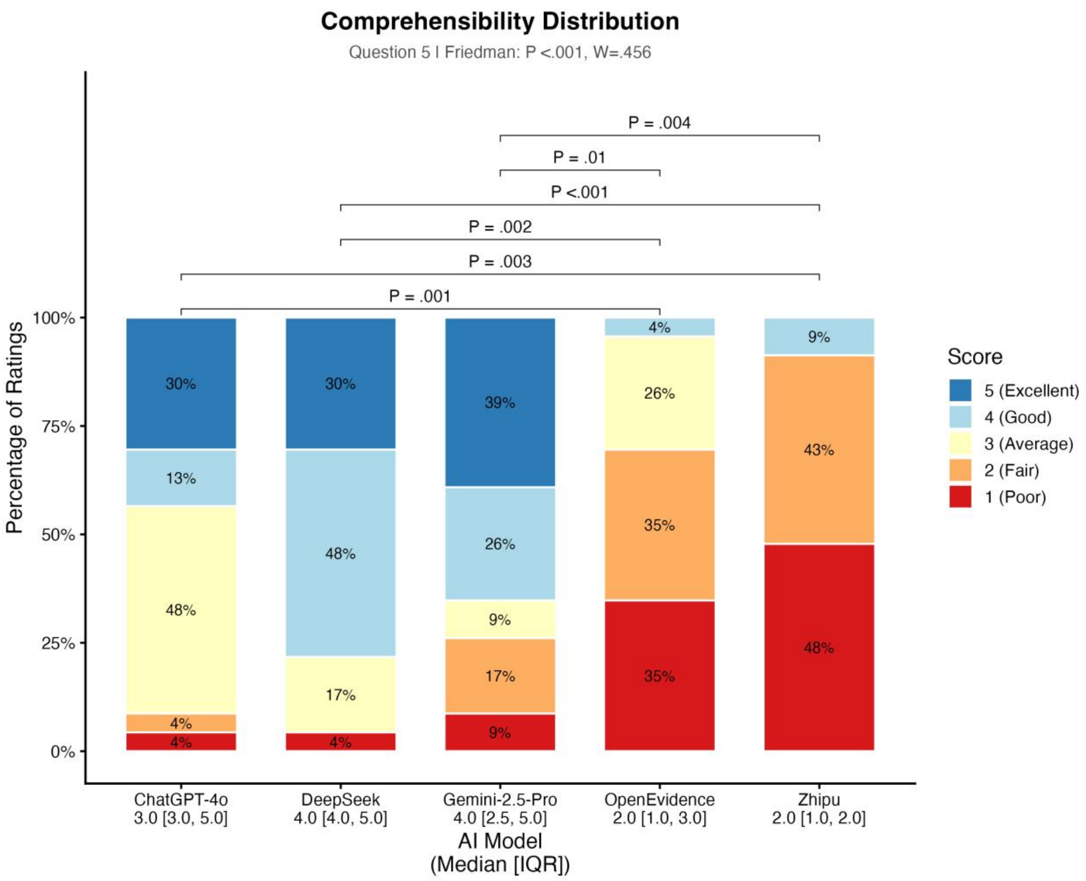
Comprehensibility Distribution
Question 5 | Friedman: P <.001, W=.456
P = .004
P = .01
P <.001
P = .002
P = .003
P = .001
Percentage of Ratings
100%
75%
50%
25%
0%
30%
13%
48%
4%
4%
30%
48%
17%
4%
39%
26%
9%
17%
9%
4%
26%
35%
35%
9%
43%
48%
ChatGPT-4o
3.0 [3.0, 5.0]
DeepSeek
4.0 [4.0, 5.0]
Gemini-2.5-Pro
4.0 [2.5, 5.0]
OpenEvidence
2.0 [1.0, 3.0]
Zhipu
2.0 [1.0, 2.0]
AI Model
(Median [IQR])
Score
5 (Excellent)
4 (Good)
3 (Average)
2 (Fair)
1 (Poor)

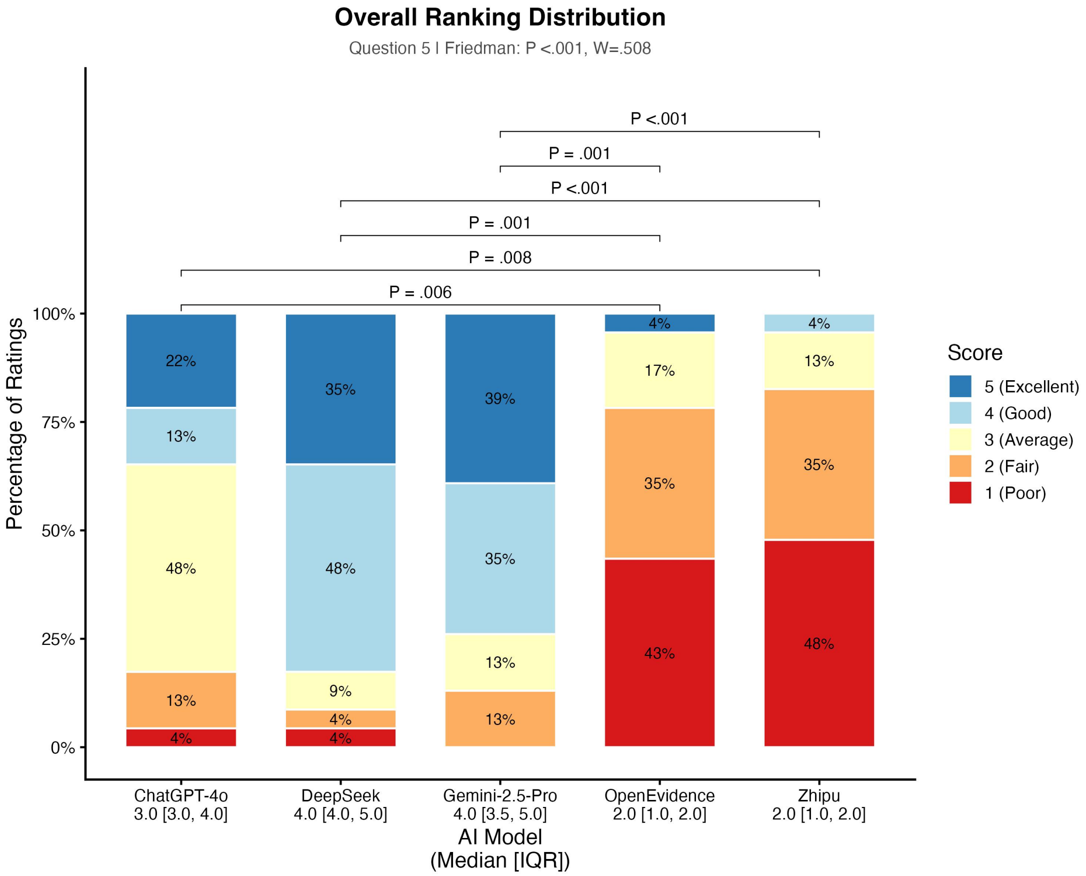
Overall Ranking Distribution
Question 5 | Friedman: P <.001, W=.508
P <.001
P = .001
P <.001
P = .001
P = .008
P = .006
Percentage of Ratings
100%
75%
50%
25%
0%
22%
13%
48%
13%
4%
35%
48%
9%
4%
4%
39%
35%
13%
13%
4%
17%
35%
43%
4%
13%
35%
48%
ChatGPT-4o
3.0 [3.0, 4.0]
DeepSeek
4.0 [4.0, 5.0]
Gemini-2.5-Pro
4.0 [3.5, 5.0]
OpenEvidence
2.0 [1.0, 2.0]
Zhipu
2.0 [1.0, 2.0]
AI Model
(Median [IQR])
Score
5 (Excellent)
4 (Good)
3 (Average)
2 (Fair)
1 (Poor)

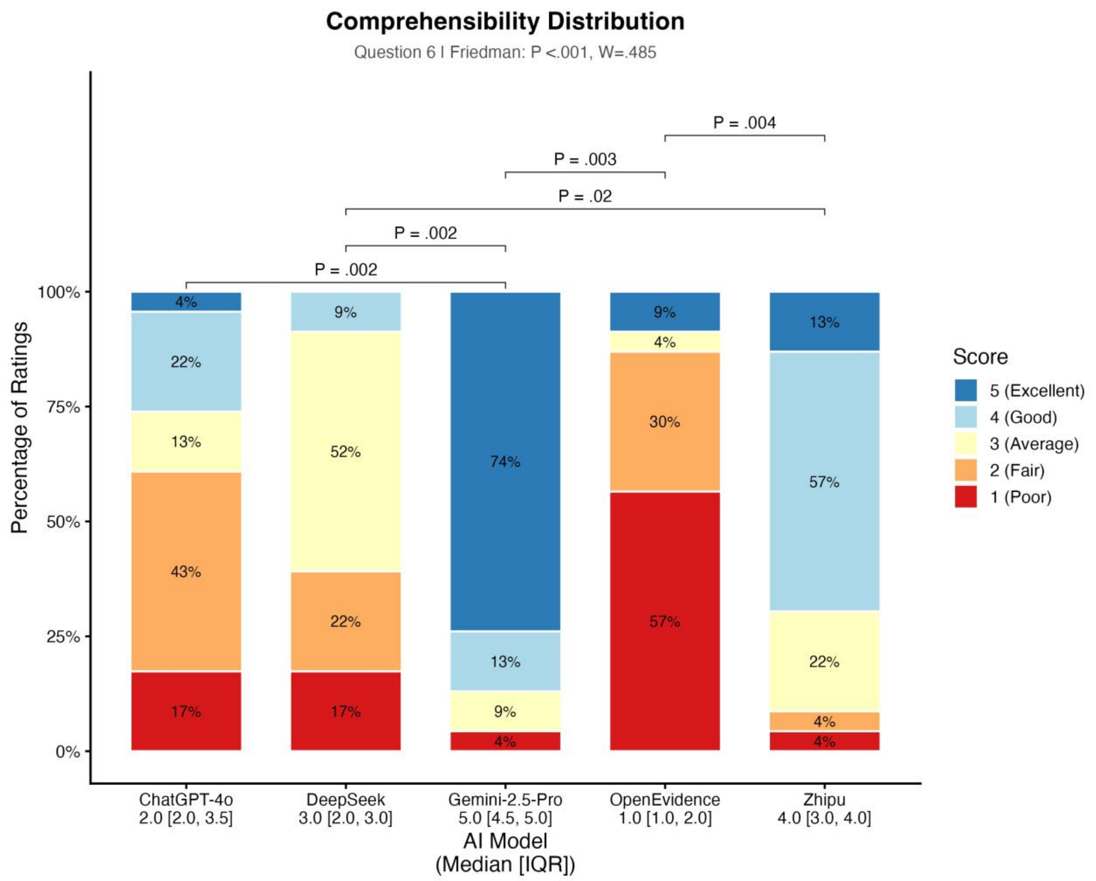

Comprehensibility Distribution
Question 6 | Friedman: P <.001, W=.485
P = .004
P = .003
P = .02
P = .002
P = .002
Percentage of Ratings
100%
75%
50%
25%
0%
4%
22%
13%
43%
17%
9%
52%
22%
17%
74%
13%
9%
4%
9%
4%
30%
57%
13%
57%
22%
4%
4%
ChatGPT-4o
2.0 [2.0, 3.5]
DeepSeek
3.0 [2.0, 3.0]
Gemini-2.5-Pro
5.0 [4.5, 5.0]
OpenEvidence
1.0 [1.0, 2.0]
Zhipu
4.0 [3.0, 4.0]
AI Model
(Median [IQR])
Score
5 (Excellent)
4 (Good)
3 (Average)
2 (Fair)
1 (Poor)

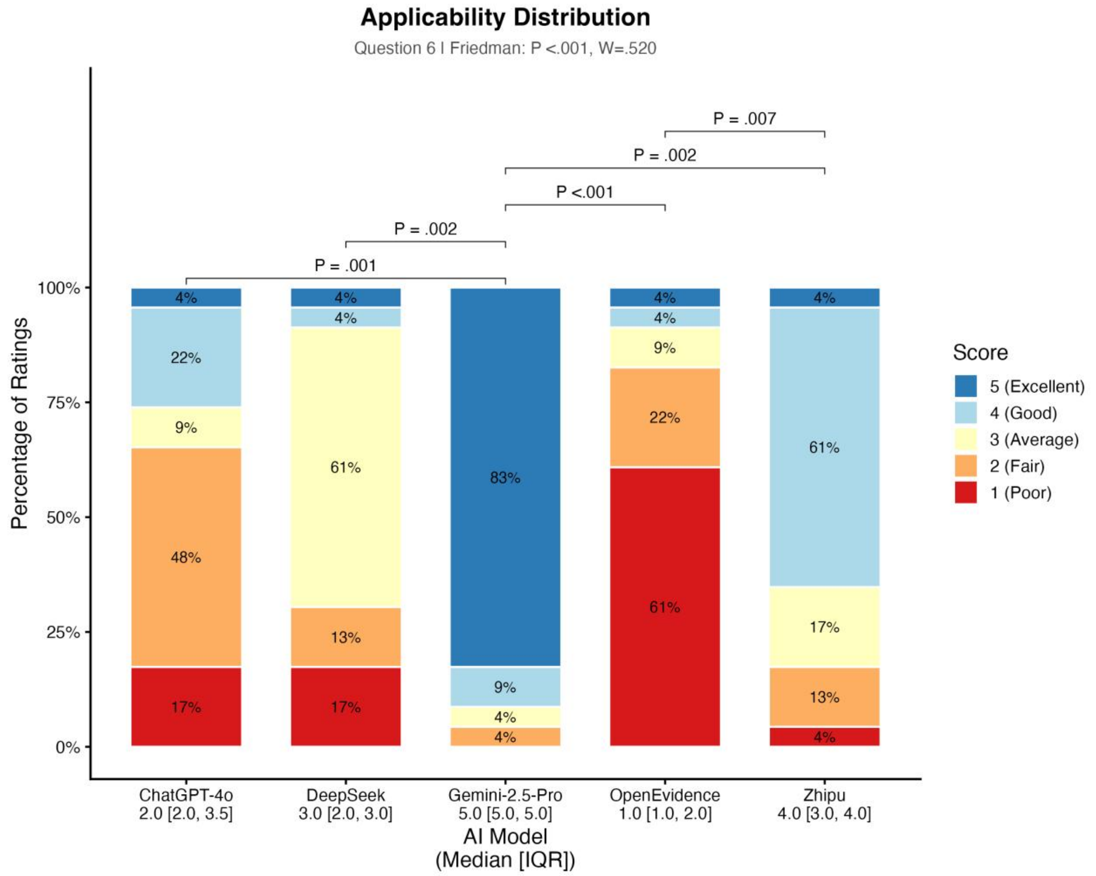

Applicability Distribution
Question 6 | Friedman: P <.001, W=.520
P = .007
P = .002
P <.001
P = .002
P = .001
Percentage of Ratings
100%
75%
50%
25%
0%
4%
22%
9%
48%
17%
4%
4%
61%
13%
17%
83%
9%
4%
4%
4%
4%
9%
22%
61%
4%
61%
17%
13%
4%
ChatGPT-4o
2.0 [2.0, 3.5]
DeepSeek
3.0 [2.0, 3.0]
Gemini-2.5-Pro
5.0 [5.0, 5.0]
OpenEvidence
1.0 [1.0, 2.0]
Zhipu
4.0 [3.0, 4.0]
AI Model
(Median [IQR])
Score
5 (Excellent)
4 (Good)
3 (Average)
2 (Fair)
1 (Poor)

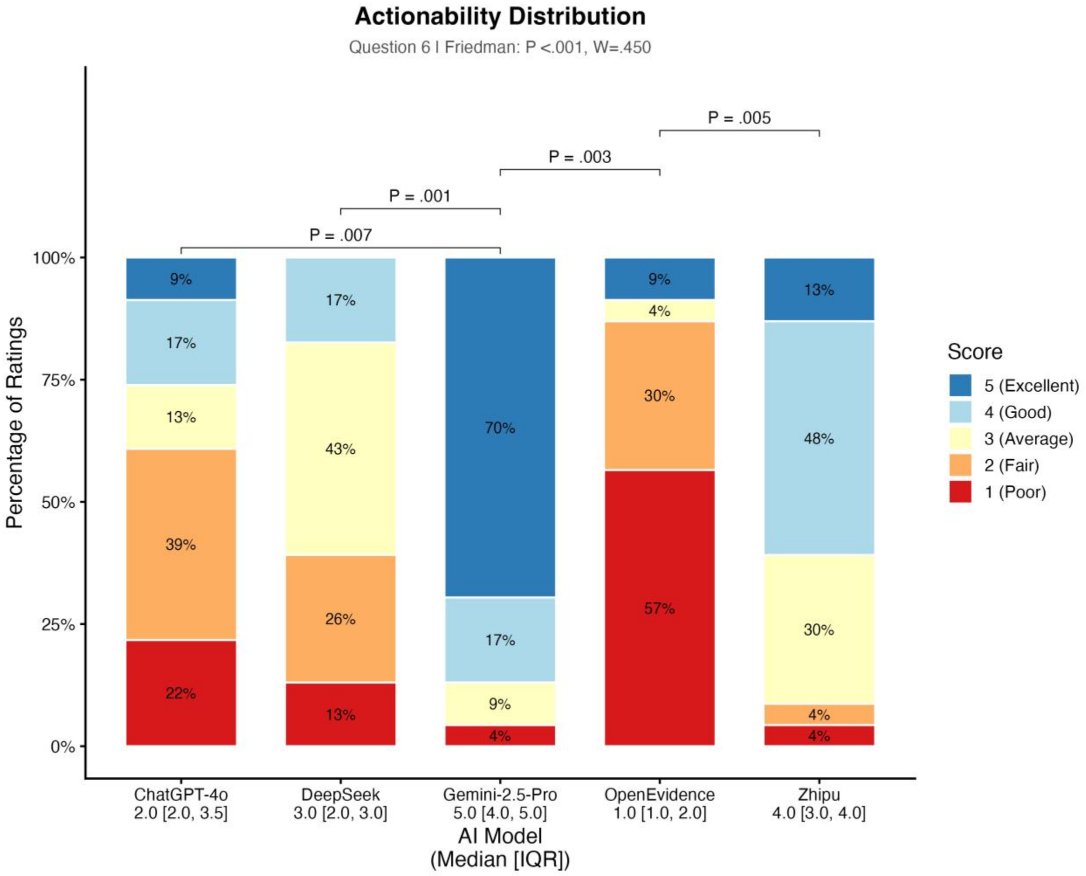
Actionability Distribution
Question 6 | Friedman: P <.001, W=.450
P = .005
P = .003
P = .001
P = .007
100%
75%
50%
25%
0%
Percentage of Ratings
9%
17%
13%
39%
22%
17%
43%
26%
13%
70%
17%
9%
4%
9%
4%
30%
57%
13%
48%
30%
4%
4%
Score
5 (Excellent)
4 (Good)
3 (Average)
2 (Fair)
1 (Poor)
ChatGPT-4o
2.0 [2.0, 3.5]
DeepSeek
3.0 [2.0, 3.0]
Gemini-2.5-Pro
5.0 [4.0, 5.0]
OpenEvidence
1.0 [1.0, 2.0]
Zhipu
4.0 [3.0, 4.0]
AI Model
(Median [IQR])

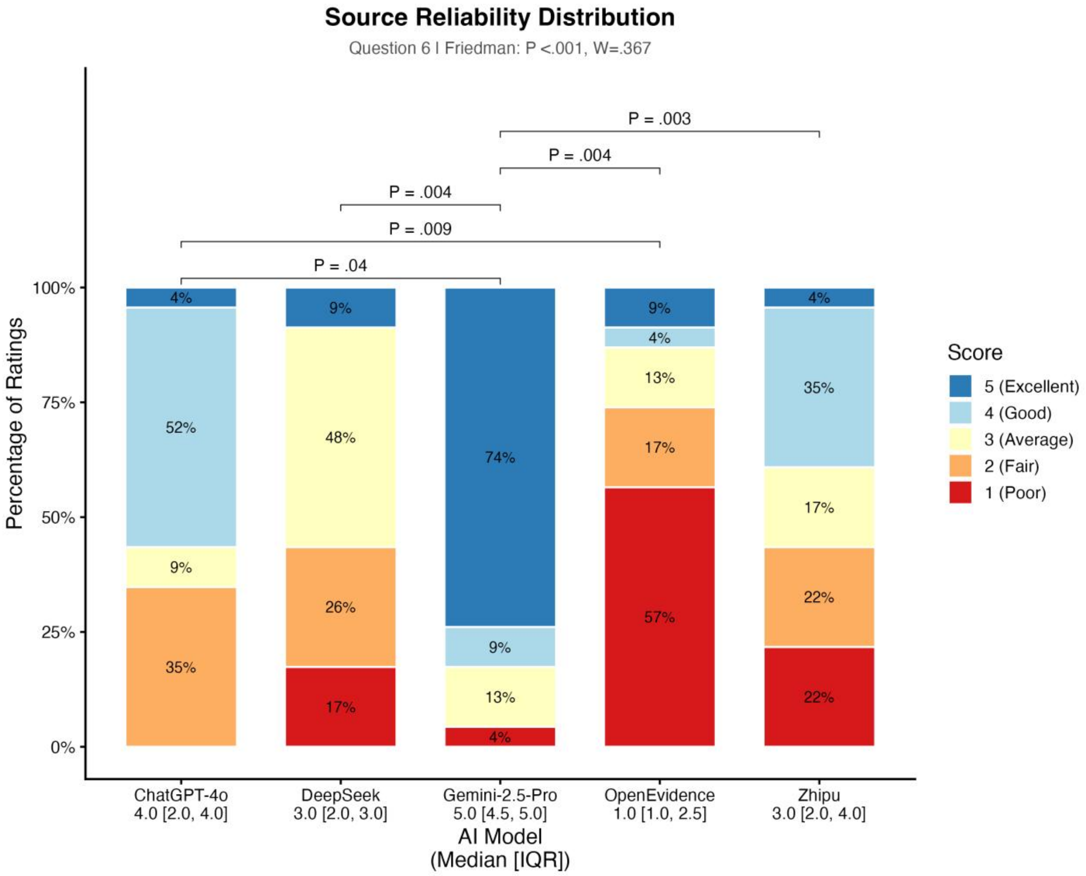
Source Reliability Distribution
Question 6 | Friedman: P <.001, W=.367
P = .003
P = .004
P = .004
P = .009
P = .04
100%
75%
50%
25%
0%
Percentage of Ratings
4%
52%
9%
35%
9%
48%
26%
17%
74%
9%
13%
4%
9%
4%
13%
17%
57%
4%
35%
17%
22%
22%
Score
5 (Excellent)
4 (Good)
3 (Average)
2 (Fair)
1 (Poor)
ChatGPT-4o
4.0 [2.0, 4.0]
DeepSeek
3.0 [2.0, 3.0]
Gemini-2.5-Pro
5.0 [4.5, 5.0]
OpenEvidence
1.0 [1.0, 2.5]
Zhipu
3.0 [2.0, 4.0]
AI Model
(Median [IQR])

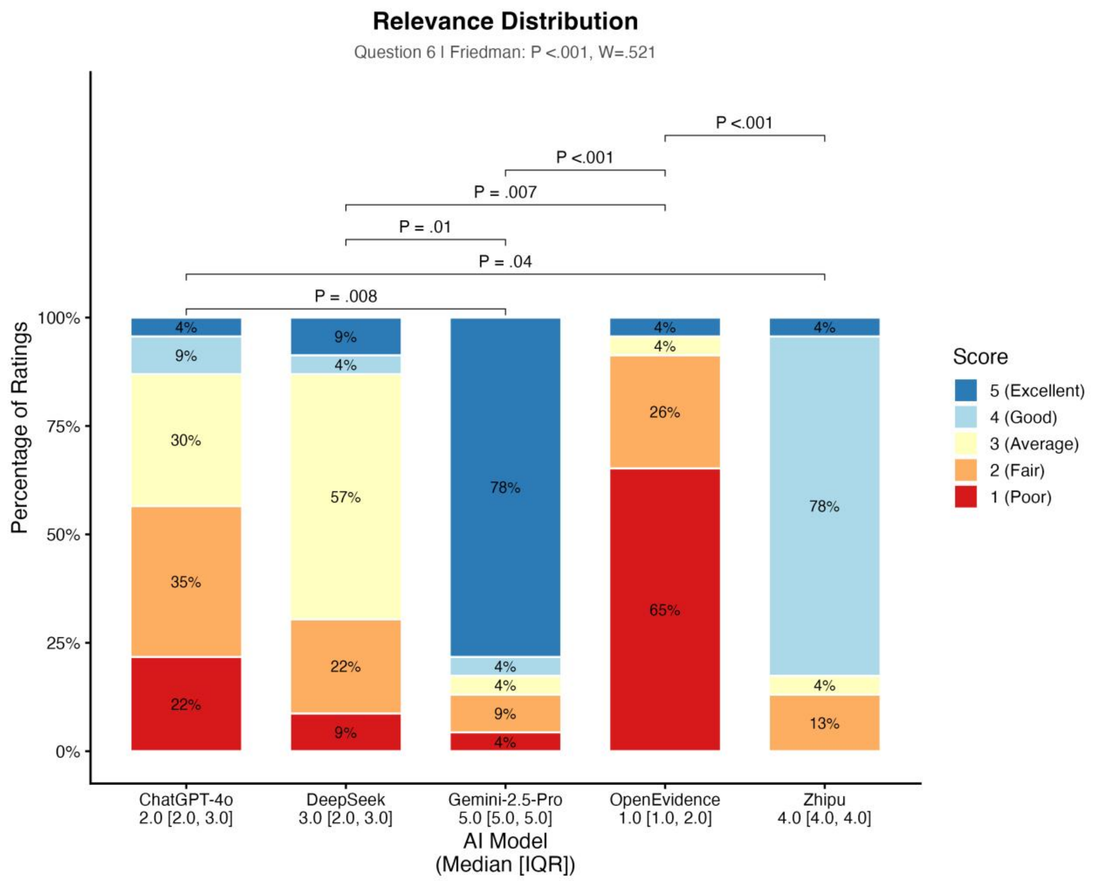
Relevance Distribution
Question 6 | Friedman: P <.001, W=.521
P <.001
P <.001
P = .007
P = .01
P = .04
P = .008
Percentage of Ratings
100%
75%
50%
25%
0%
4%
9%
30%
35%
22%
9%
4%
57%
22%
9%
78%
4%
4%
9%
4%
4%
4%
26%
65%
4%
78%
4%
13%
Score
5 (Excellent)
4 (Good)
3 (Average)
2 (Fair)
1 (Poor)
ChatGPT-4o
2.0 [2.0, 3.0]
DeepSeek
3.0 [2.0, 3.0]
Gemini-2.5-Pro
5.0 [5.0, 5.0]
OpenEvidence
1.0 [1.0, 2.0]
Zhipu
4.0 [4.0, 4.0]
AI Model
(Median [IQR])

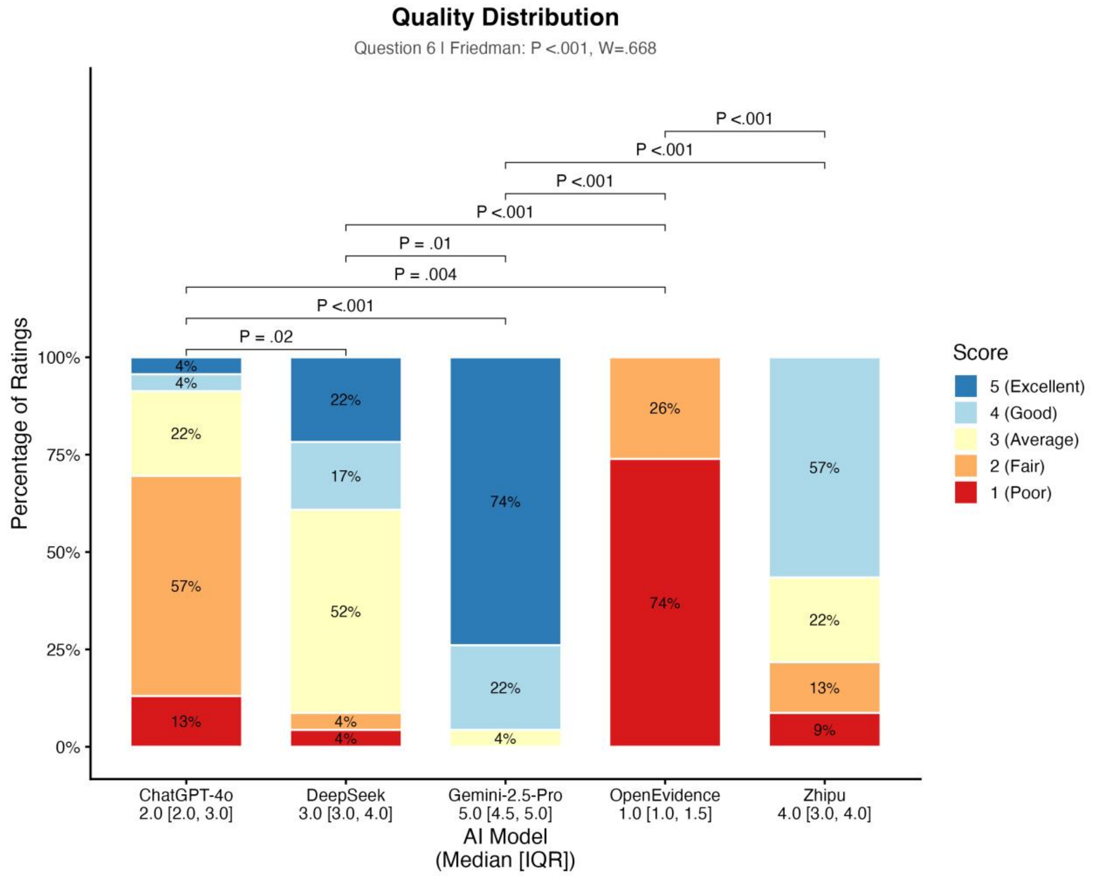
Quality Distribution
Question 6 | Friedman: P <.001, W=.668
P <.001
P <.001
P <.001
P <.001
P = .01
P = .004
P <.001
P = .02
Percentage of Ratings
100%
75%
50%
25%
0%
4%
4%
22%
57%
13%
22%
17%
52%
4%
4%
74%
22%
4%
26%
74%
57%
22%
13%
9%
Score
5 (Excellent)
4 (Good)
3 (Average)
2 (Fair)
1 (Poor)
ChatGPT-4o
2.0 [2.0, 3.0]
DeepSeek
3.0 [3.0, 4.0]
Gemini-2.5-Pro
5.0 [4.5, 5.0]
OpenEvidence
1.0 [1.0, 1.5]
Zhipu
4.0 [3.0, 4.0]
AI Model
(Median [IQR])

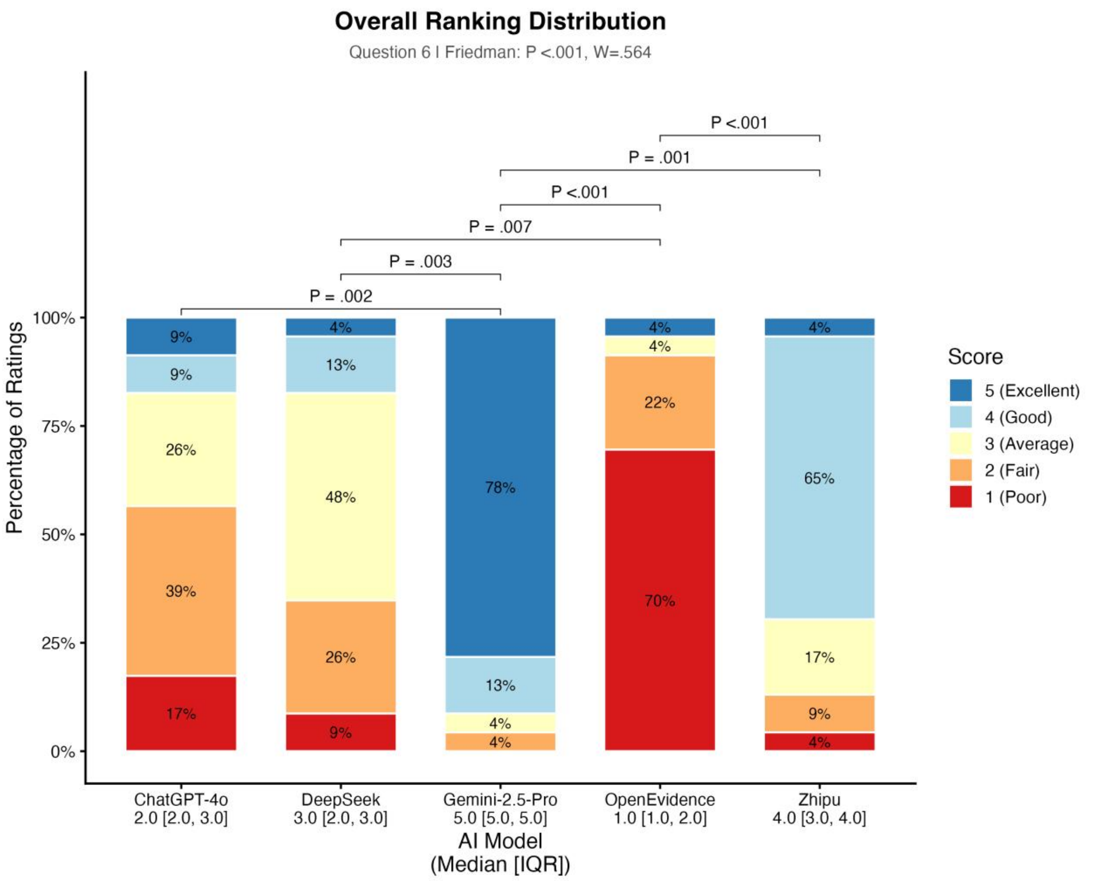
Overall Ranking Distribution
Question 6 | Friedman: P <.001, W=.564
Percentage of Ratings
P <.001
P = .001
P <.001
P = .007
P = .003
P = .002
100%
75%
50%
25%
0%
ChatGPT-4o
2.0 [2.0, 3.0]
DeepSeek
3.0 [2.0, 3.0]
Gemini-2.5-Pro
5.0 [5.0, 5.0]
OpenEvidence
1.0 [1.0, 2.0]
Zhipu
4.0 [3.0, 4.0]
AI Model
(Median [IQR])
Score
5 (Excellent)
4 (Good)
3 (Average)
2 (Fair)
1 (Poor)

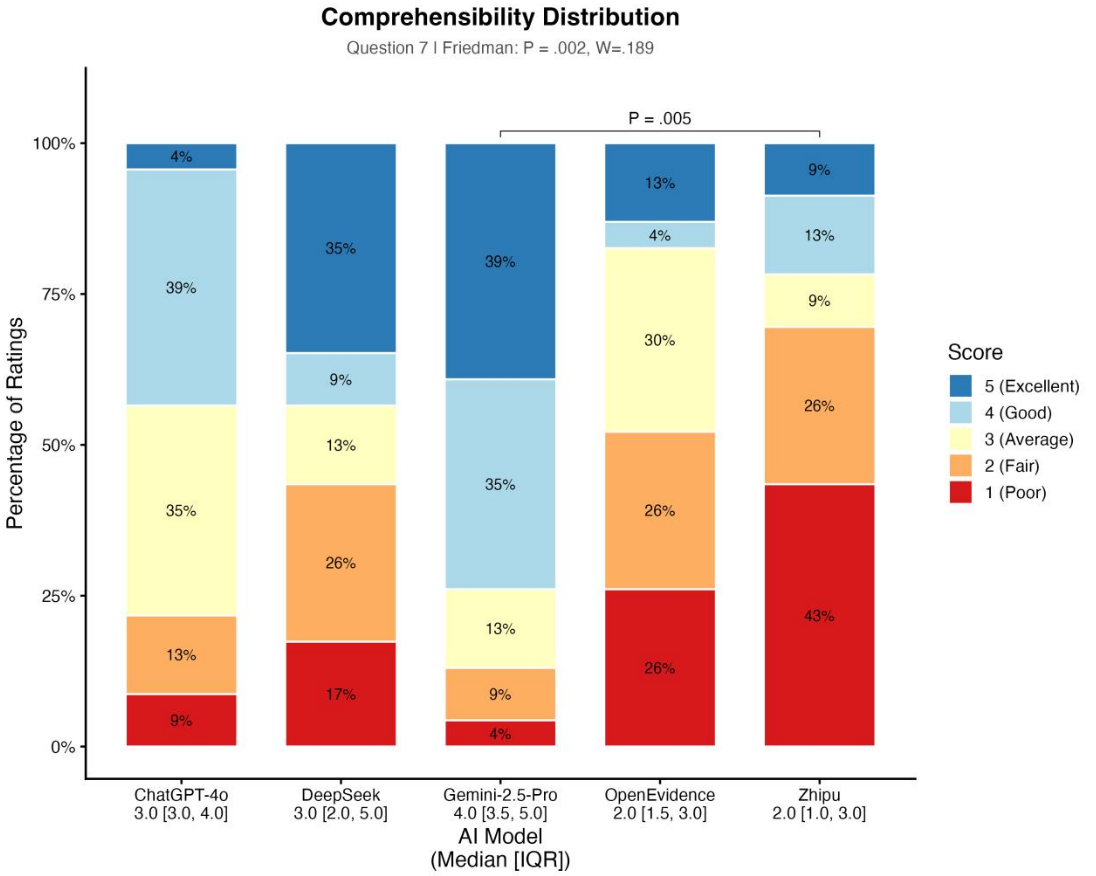
Comprehensibility Distribution
Question 7 | Friedman: P = .002, W=.189
Percentage of Ratings
P = .005
100%
75%
50%
25%
0%
ChatGPT-4o
3.0 [3.0, 4.0]
DeepSeek
3.0 [2.0, 5.0]
Gemini-2.5-Pro
4.0 [3.5, 5.0]
OpenEvidence
2.0 [1.5, 3.0]
Zhipu
2.0 [1.0, 3.0]
AI Model
(Median [IQR])
Score
5 (Excellent)
4 (Good)
3 (Average)
2 (Fair)
1 (Poor)

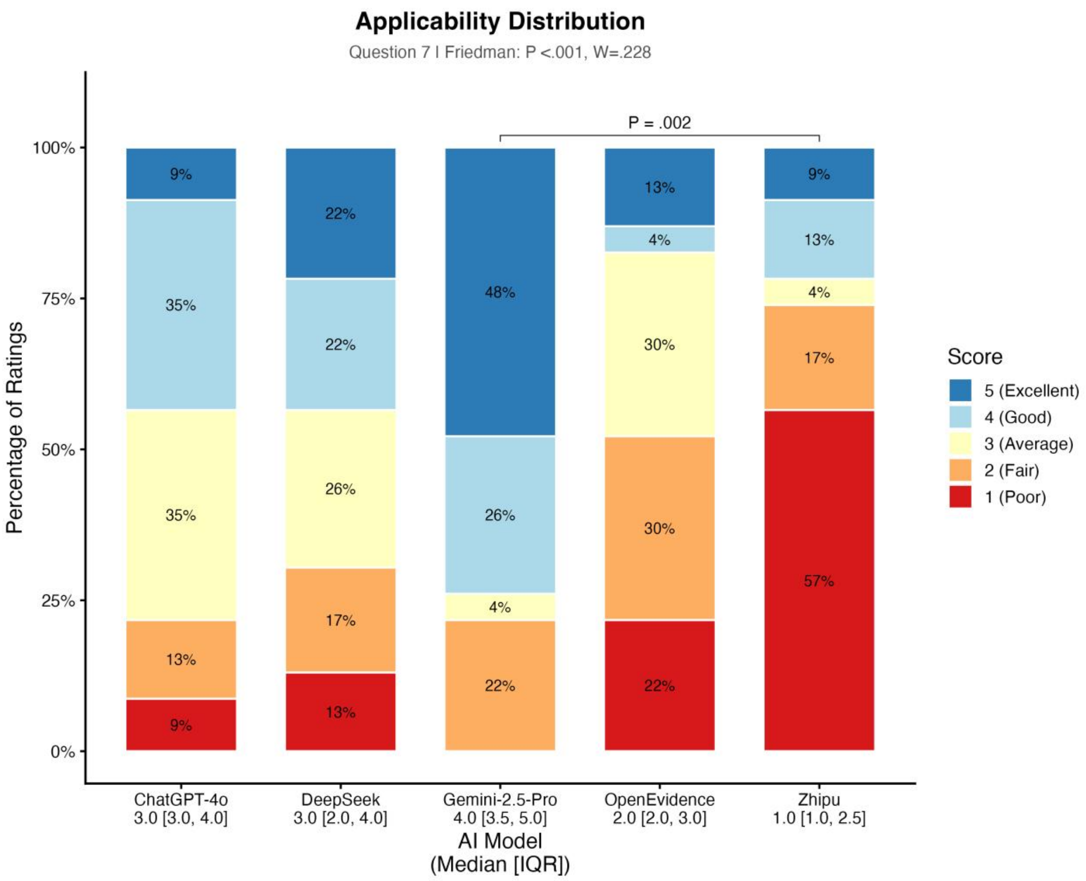
Applicability Distribution
Question 7 | Friedman: P <.001, W=.228
P = .002
Percentage of Ratings
100%
75%
50%
25%
0%
9%
35%
35%
13%
9%
22%
22%
26%
17%
13%
48%
26%
4%
22%
13%
4%
30%
30%
22%
9%
13%
4%
17%
57%
Score
5 (Excellent)
4 (Good)
3 (Average)
2 (Fair)
1 (Poor)
ChatGPT-4o
3.0 [3.0, 4.0]
DeepSeek
3.0 [2.0, 4.0]
Gemini-2.5-Pro
4.0 [3.5, 5.0]
OpenEvidence
2.0 [2.0, 3.0]
Zhipu
1.0 [1.0, 2.5]
AI Model
(Median [IQR])

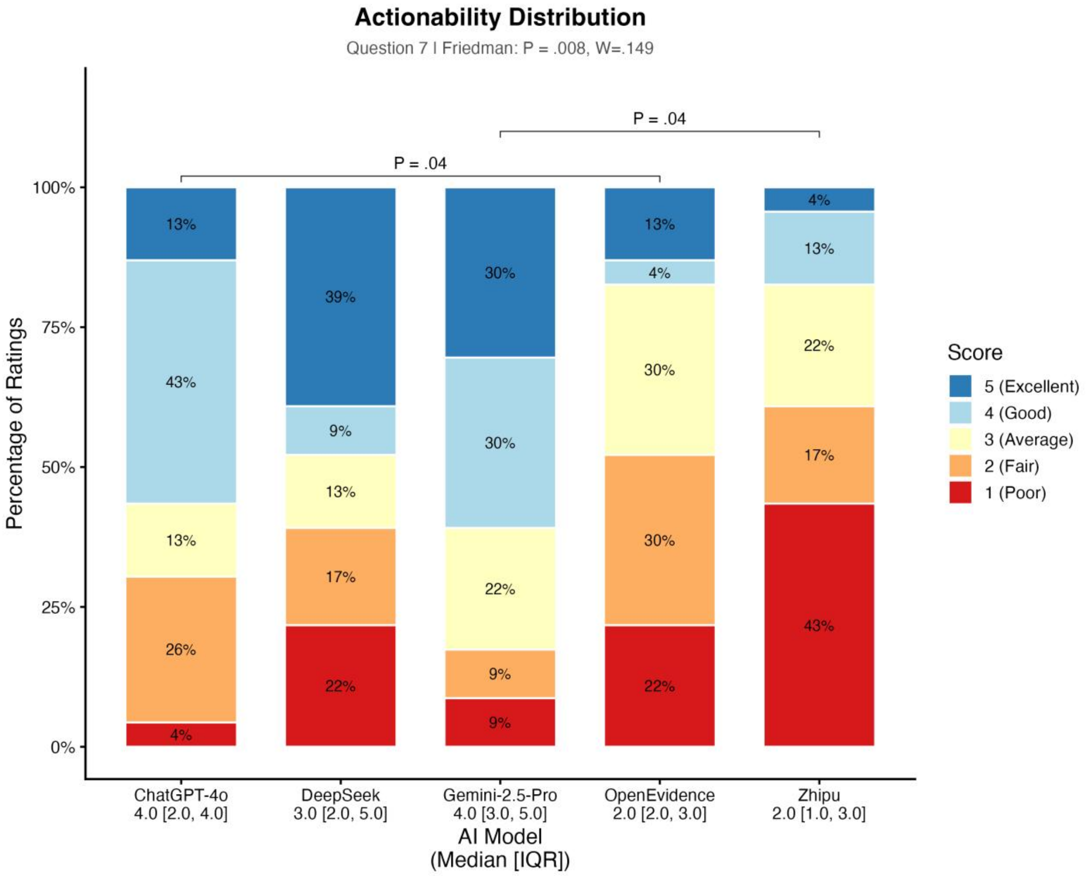
Actionability Distribution
Question 7 | Friedman: P = .008, W=.149
P = .04
P = .04
Percentage of Ratings
100%
75%
50%
25%
0%
13%
43%
13%
26%
4%
39%
9%
13%
17%
22%
30%
30%
22%
9%
9%
13%
4%
30%
30%
22%
4%
13%
22%
17%
43%
Score
5 (Excellent)
4 (Good)
3 (Average)
2 (Fair)
1 (Poor)
ChatGPT-4o
4.0 [2.0, 4.0]
DeepSeek
3.0 [2.0, 5.0]
Gemini-2.5-Pro
4.0 [3.0, 5.0]
OpenEvidence
2.0 [2.0, 3.0]
Zhipu
2.0 [1.0, 3.0]
AI Model
(Median [IQR])

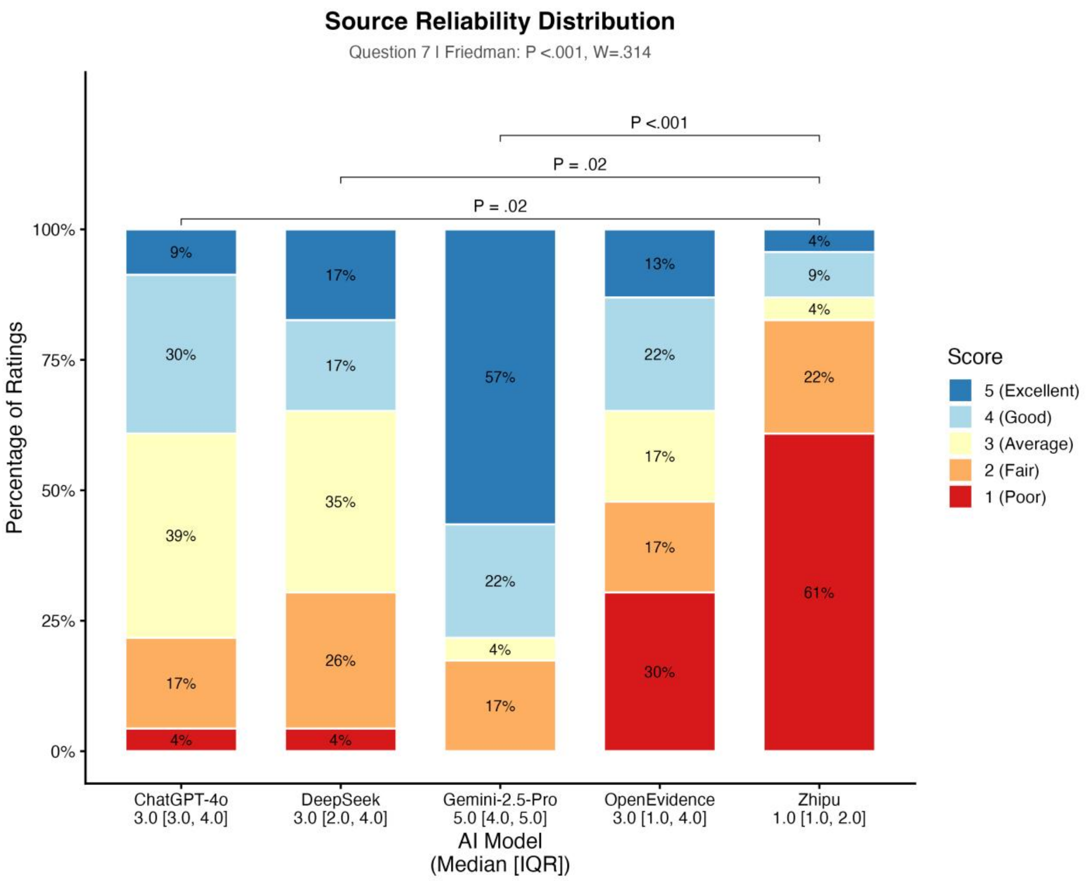
Source Reliability Distribution
Question 7 | Friedman: P <.001, W=.314
P <.001
P = .02
P = .02
Percentage of Ratings
100%
75%
50%
25%
0%
9%
30%
39%
17%
4%
17%
17%
35%
26%
4%
57%
22%
4%
17%
13%
22%
17%
17%
30%
4%
9%
4%
22%
61%
ChatGPT-4o
3.0 [3.0, 4.0]
DeepSeek
3.0 [2.0, 4.0]
Gemini-2.5-Pro
5.0 [4.0, 5.0]
OpenEvidence
3.0 [1.0, 4.0]
Zhipu
1.0 [1.0, 2.0]
AI Model
(Median [IQR])
Score
5 (Excellent)
4 (Good)
3 (Average)
2 (Fair)
1 (Poor)

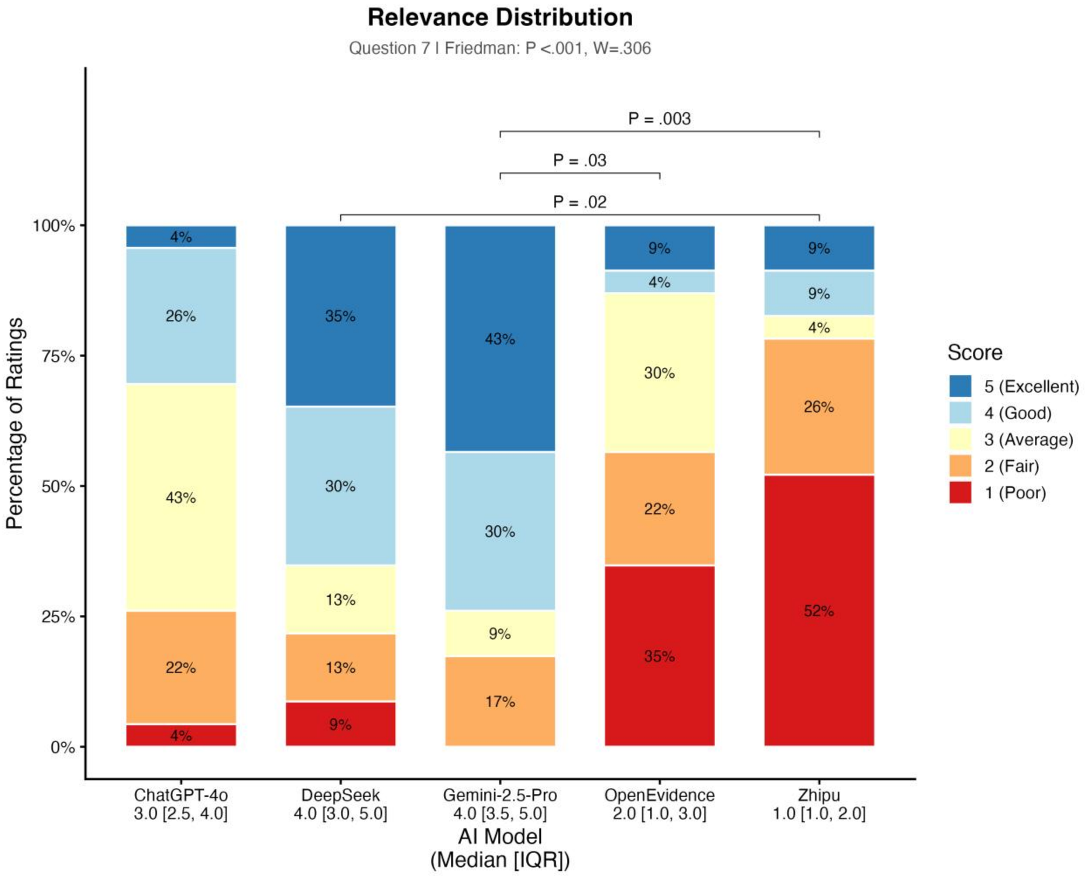
Relevance Distribution
Question 7 | Friedman: P <.001, W=.306
P = .003
P = .03
P = .02
Percentage of Ratings
100%
75%
50%
25%
0%
4%
26%
43%
22%
4%
35%
30%
13%
13%
9%
43%
30%
9%
17%
9%
4%
30%
22%
35%
9%
9%
4%
26%
52%
ChatGPT-4o
3.0 [2.5, 4.0]
DeepSeek
4.0 [3.0, 5.0]
Gemini-2.5-Pro
4.0 [3.5, 5.0]
OpenEvidence
2.0 [1.0, 3.0]
Zhipu
1.0 [1.0, 2.0]
AI Model
(Median [IQR])
Score
5 (Excellent)
4 (Good)
3 (Average)
2 (Fair)
1 (Poor)

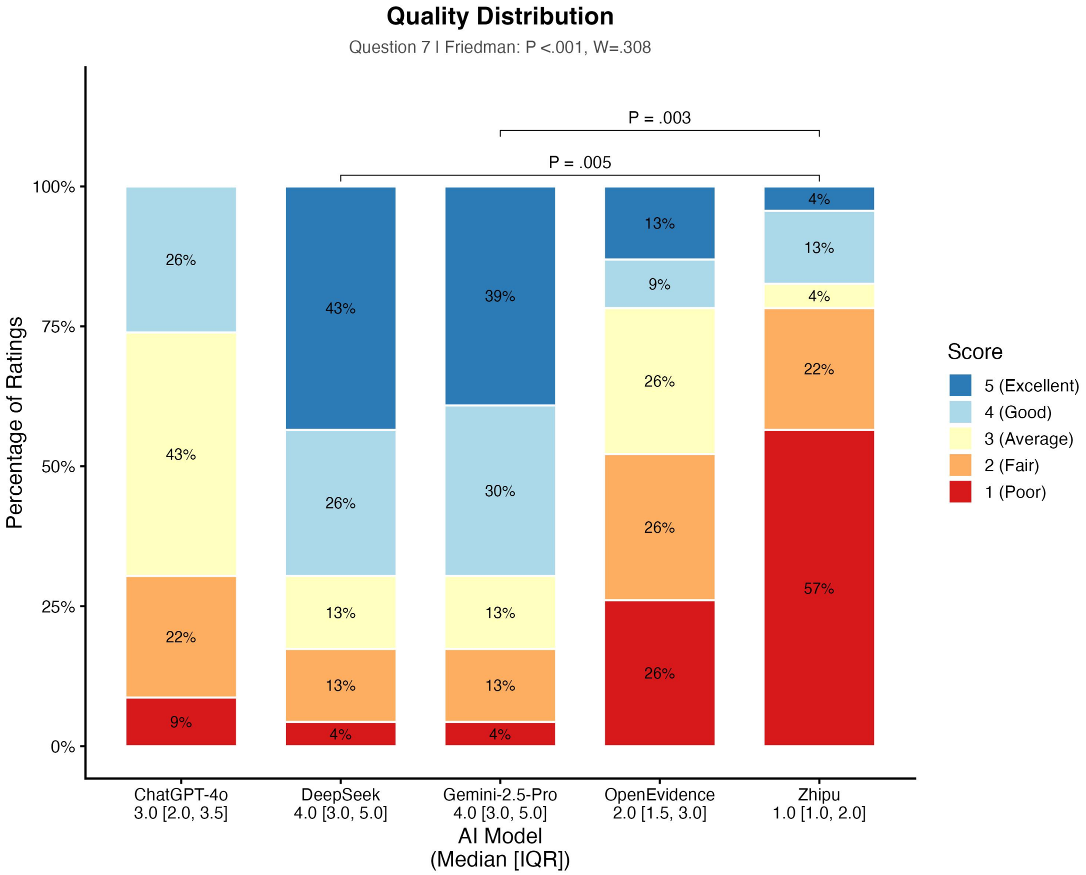
Quality Distribution
Question 7 | Friedman: P <.001, W=.308
P = .003
P = .005
Percentage of Ratings
100%
75%
50%
25%
0%
26%
43%
22%
9%
43%
26%
13%
13%
4%
39%
30%
13%
13%
4%
13%
9%
26%
26%
26%
4%
13%
4%
22%
57%
ChatGPT-4o
3.0 [2.0, 3.5]
DeepSeek
4.0 [3.0, 5.0]
Gemini-2.5-Pro
4.0 [3.0, 5.0]
OpenEvidence
2.0 [1.5, 3.0]
Zhipu
1.0 [1.0, 2.0]
AI Model
(Median [IQR])
Score
5 (Excellent)
4 (Good)
3 (Average)
2 (Fair)
1 (Poor)

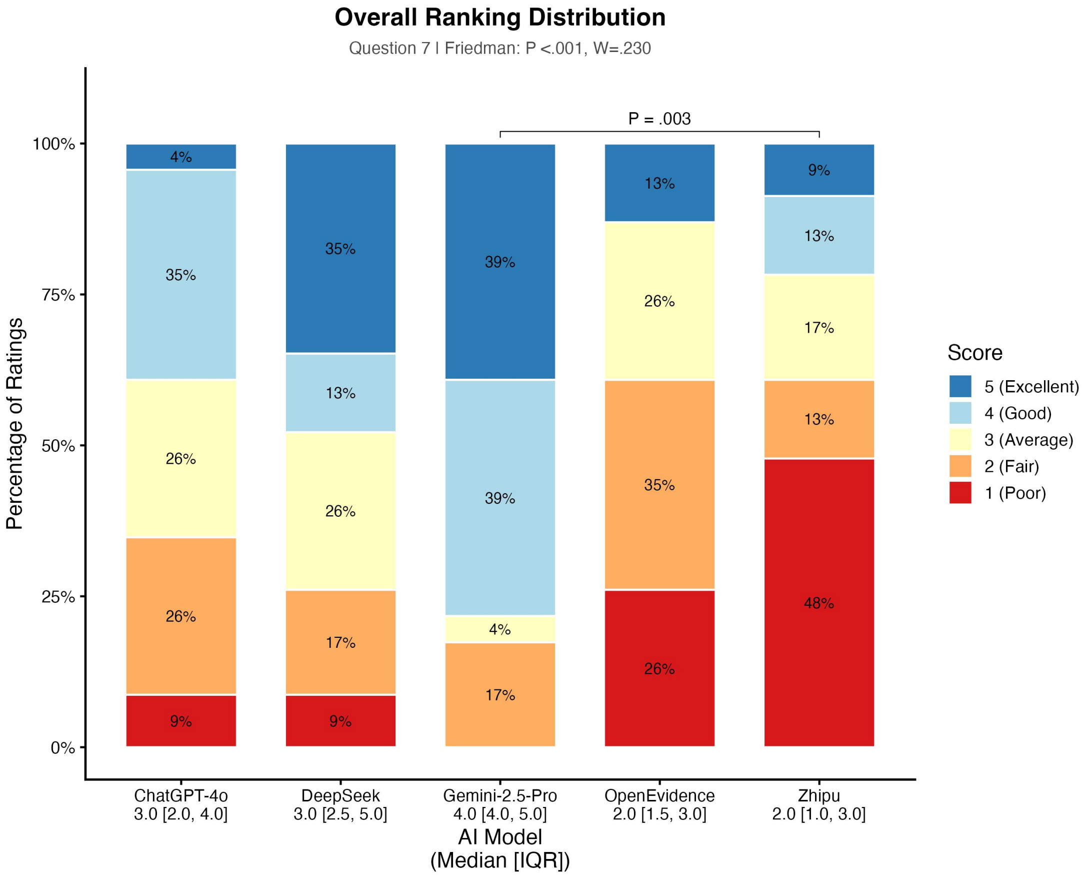
Overall Ranking Distribution
Question 7 | Friedman: P <.001, W=.230
P = .003
Percentage of Ratings
100%
75%
50%
25%
0%
4%
35%
26%
26%
9%
35%
13%
26%
17%
9%
39%
39%
4%
17%
13%
26%
35%
26%
9%
13%
17%
13%
48%
ChatGPT-4o
3.0 [2.0, 4.0]
DeepSeek
3.0 [2.5, 5.0]
Gemini-2.5-Pro
4.0 [4.0, 5.0]
OpenEvidence
2.0 [1.5, 3.0]
Zhipu
2.0 [1.0, 3.0]
AI Model
(Median [IQR])
Score
5 (Excellent)
4 (Good)
3 (Average)
2 (Fair)
1 (Poor)

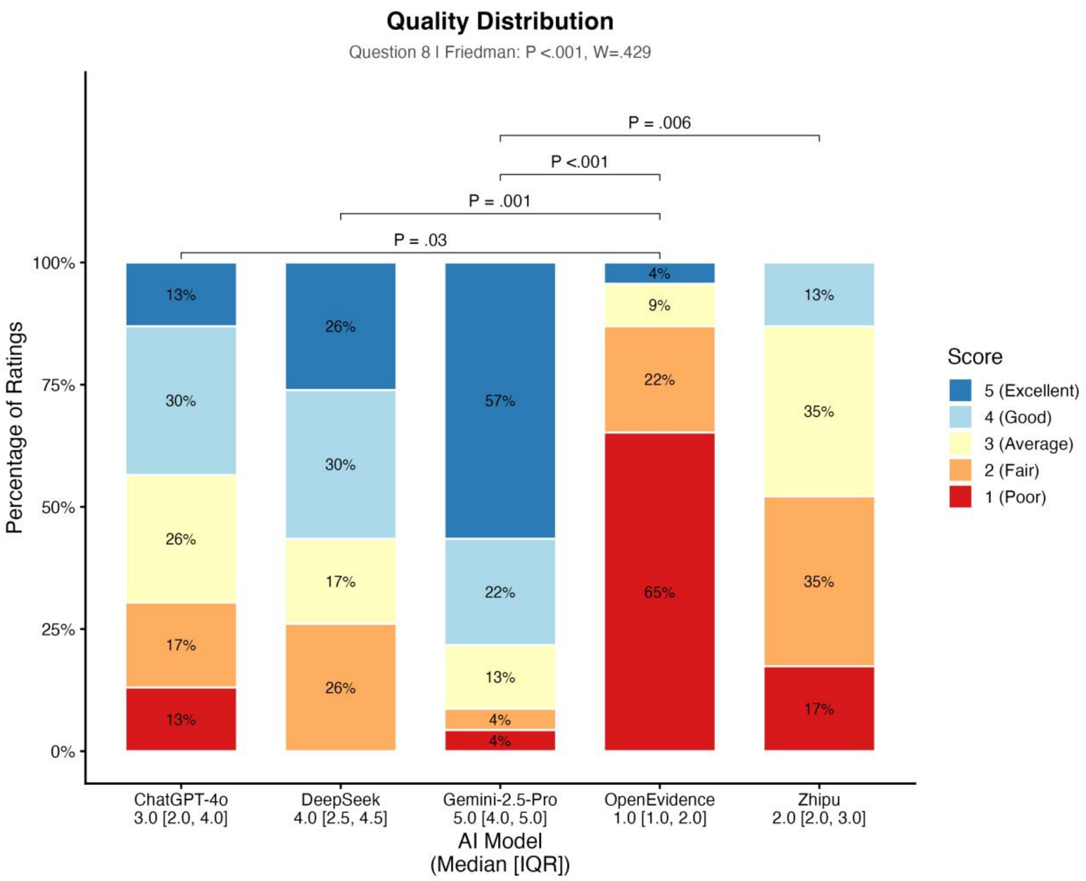
Quality Distribution
Question 8 | Friedman: P <.001, W=.429
P = .006
P <.001
P = .001
P = .03
Percentage of Ratings
100%
75%
50%
25%
0%
13%
30%
26%
17%
13%
26%
30%
17%
26%
57%
22%
13%
4%
4%
4%
9%
22%
65%
13%
35%
35%
17%
Score
5 (Excellent)
4 (Good)
3 (Average)
2 (Fair)
1 (Poor)
ChatGPT-4o
3.0 [2.0, 4.0]
DeepSeek
4.0 [2.5, 4.5]
Gemini-2.5-Pro
5.0 [4.0, 5.0]
OpenEvidence
1.0 [1.0, 2.0]
Zhipu
2.0 [2.0, 3.0]
AI Model
(Median [IQR])

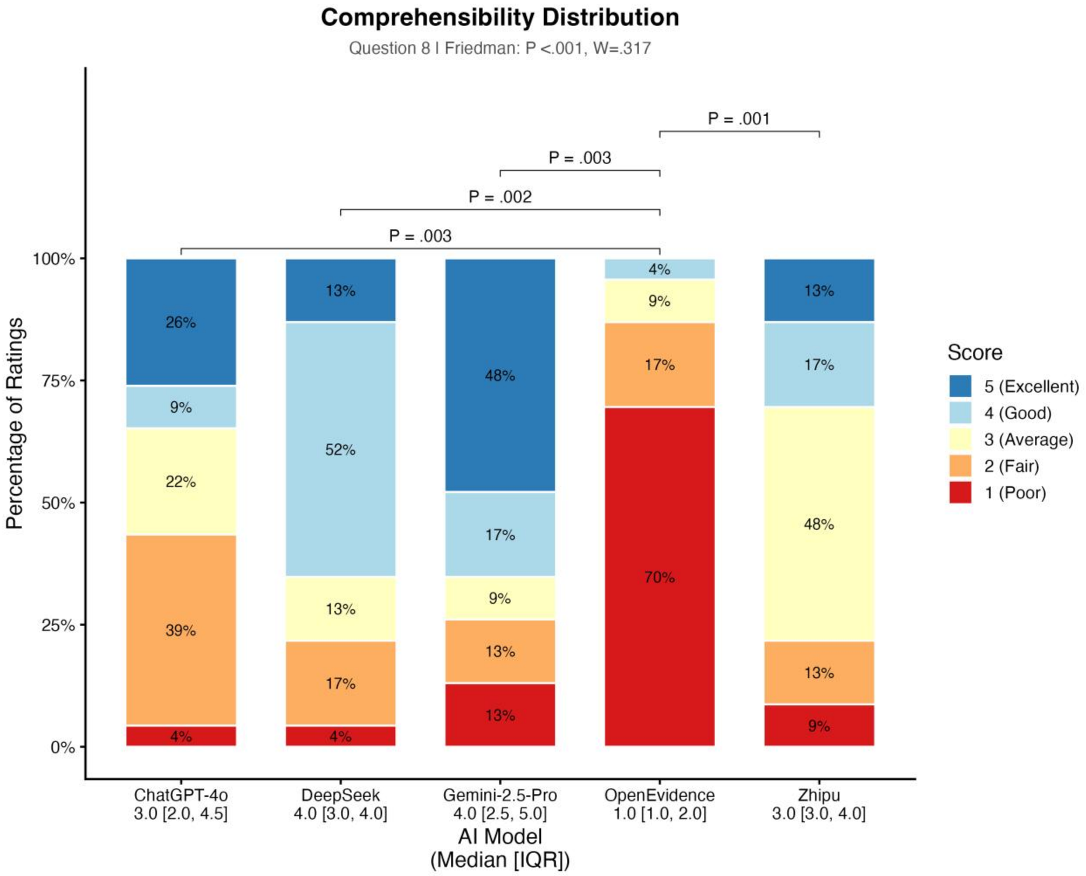
Comprehensibility Distribution
Question 8 | Friedman: P <.001, W=.317
P = .001
P = .003
P = .002
P = .003
Percentage of Ratings
100%
75%
50%
25%
0%
26%
9%
22%
39%
4%
13%
52%
13%
17%
4%
48%
17%
9%
13%
13%
4%
9%
17%
70%
13%
17%
48%
13%
9%
Score
5 (Excellent)
4 (Good)
3 (Average)
2 (Fair)
1 (Poor)
ChatGPT-4o
3.0 [2.0, 4.5]
DeepSeek
4.0 [3.0, 4.0]
Gemini-2.5-Pro
4.0 [2.5, 5.0]
OpenEvidence
1.0 [1.0, 2.0]
Zhipu
3.0 [3.0, 4.0]
AI Model
(Median [IQR])

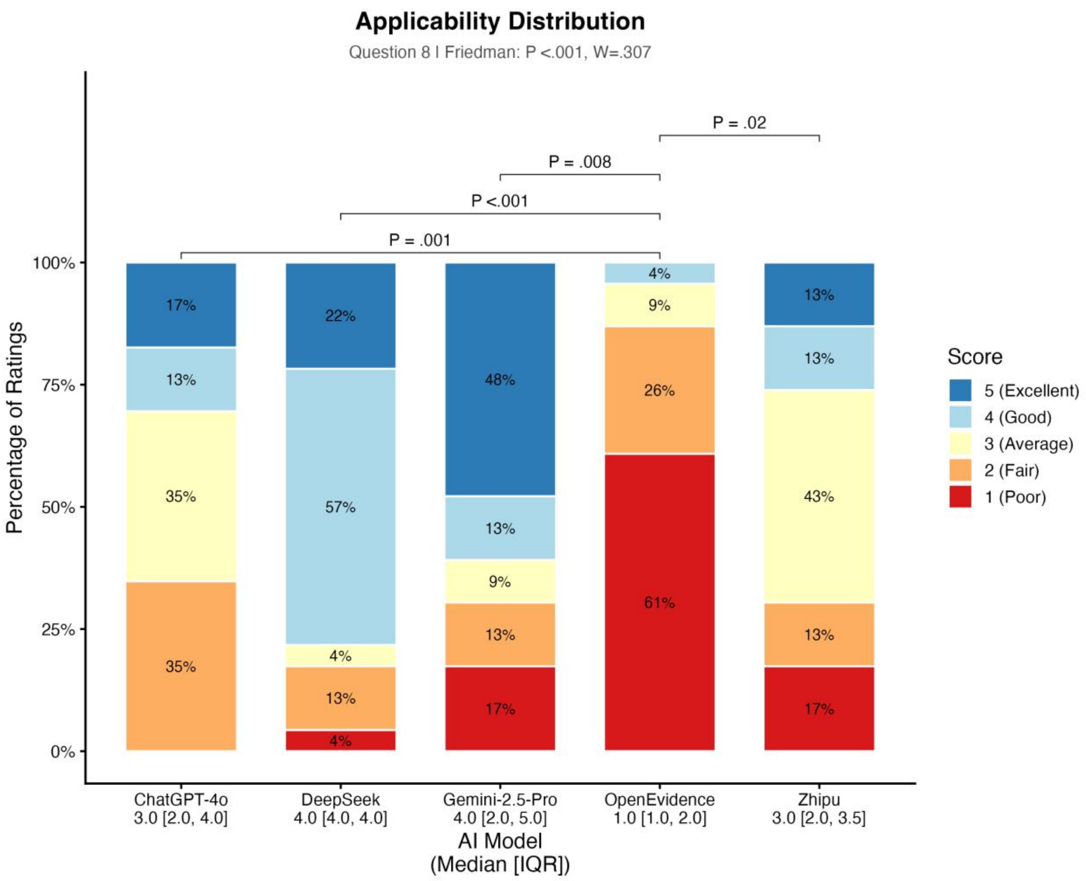
Applicability Distribution
Question 8 | Friedman: P <.001, W=.307
P = .02
P = .008
P <.001
P = .001
Percentage of Ratings
100%
75%
50%
25%
0%
17%
13%
35%
35%
22%
57%
4%
13%
4%
48%
13%
9%
13%
17%
4%
9%
26%
61%
13%
13%
43%
13%
17%
ChatGPT-4o
3.0 [2.0, 4.0]
DeepSeek
4.0 [4.0, 4.0]
Gemini-2.5-Pro
4.0 [2.0, 5.0]
OpenEvidence
1.0 [1.0, 2.0]
Zhipu
3.0 [2.0, 3.5]
AI Model
(Median [IQR])
Score
5 (Excellent)
4 (Good)
3 (Average)
2 (Fair)
1 (Poor)

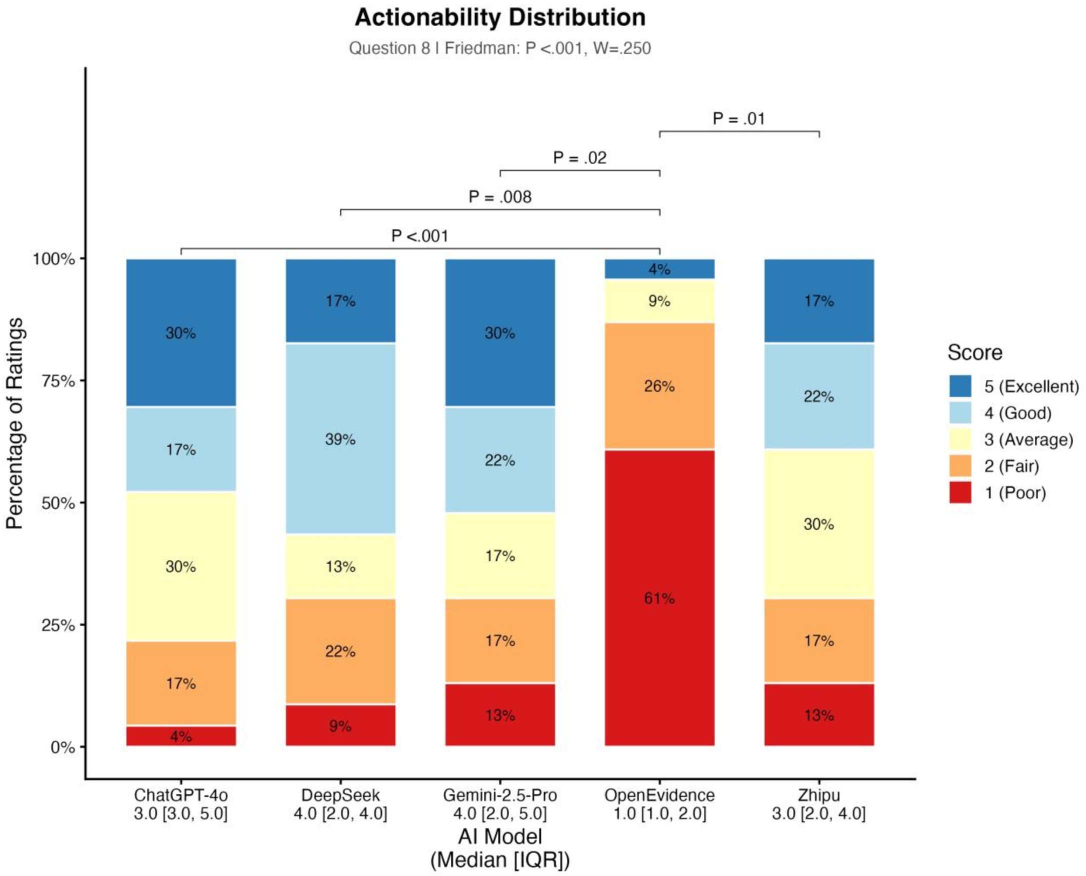
Actionability Distribution
Question 8 | Friedman: P <.001, W=.250
P = .01
P = .02
P = .008
P <.001
Percentage of Ratings
100%
75%
50%
25%
0%
30%
17%
30%
17%
4%
17%
39%
13%
22%
9%
30%
22%
17%
17%
13%
4%
9%
26%
61%
17%
22%
30%
17%
13%
ChatGPT-4o
3.0 [3.0, 5.0]
DeepSeek
4.0 [2.0, 4.0]
Gemini-2.5-Pro
4.0 [2.0, 5.0]
OpenEvidence
1.0 [1.0, 2.0]
Zhipu
3.0 [2.0, 4.0]
AI Model
(Median [IQR])
Score
5 (Excellent)
4 (Good)
3 (Average)
2 (Fair)
1 (Poor)

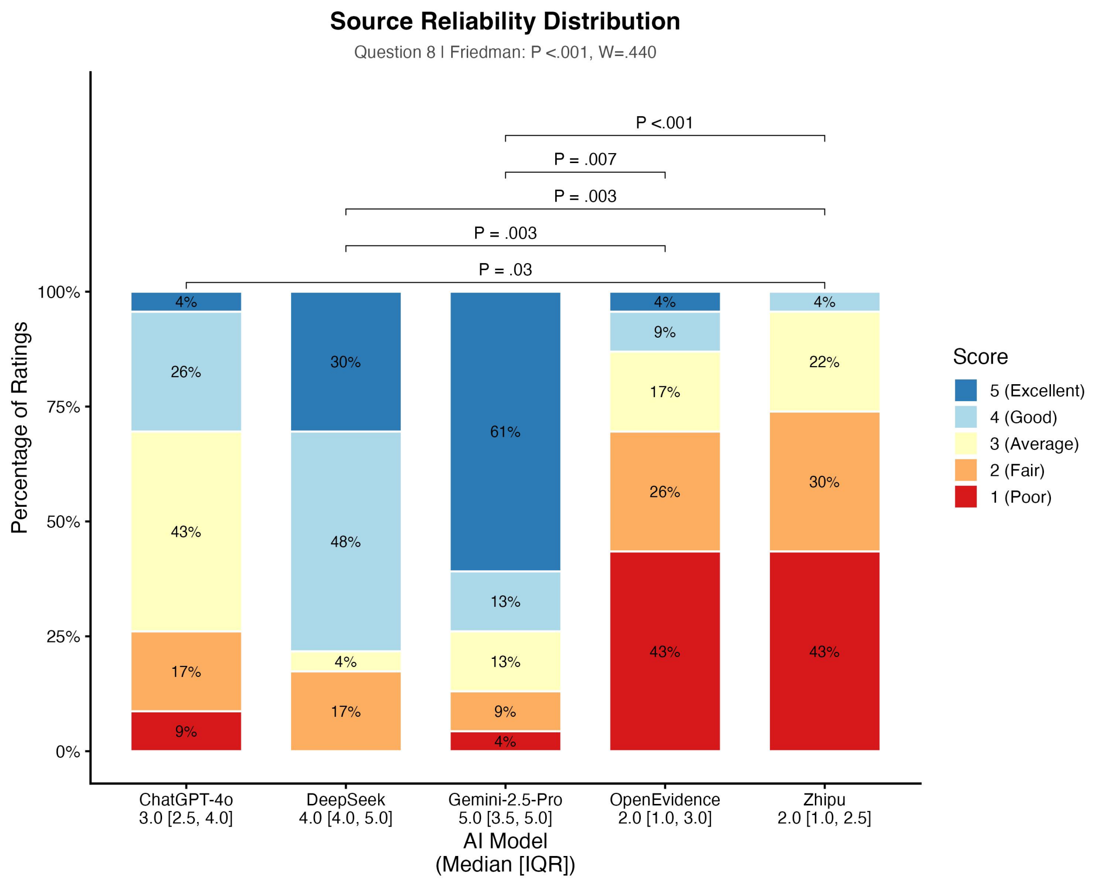
Source Reliability Distribution
Question 8 | Friedman: P <.001, W=.440
P <.001
P = .007
P = .003
P = .003
P = .03
Percentage of Ratings
100%
75%
50%
25%
0%
4%
26%
43%
17%
9%
30%
48%
4%
17%
61%
13%
13%
9%
4%
4%
9%
17%
26%
43%
4%
22%
30%
43%
ChatGPT-4o
3.0 [2.5, 4.0]
DeepSeek
4.0 [4.0, 5.0]
Gemini-2.5-Pro
5.0 [3.5, 5.0]
OpenEvidence
2.0 [1.0, 3.0]
Zhipu
2.0 [1.0, 2.5]
AI Model
(Median [IQR])
Score
5 (Excellent)
4 (Good)
3 (Average)
2 (Fair)
1 (Poor)

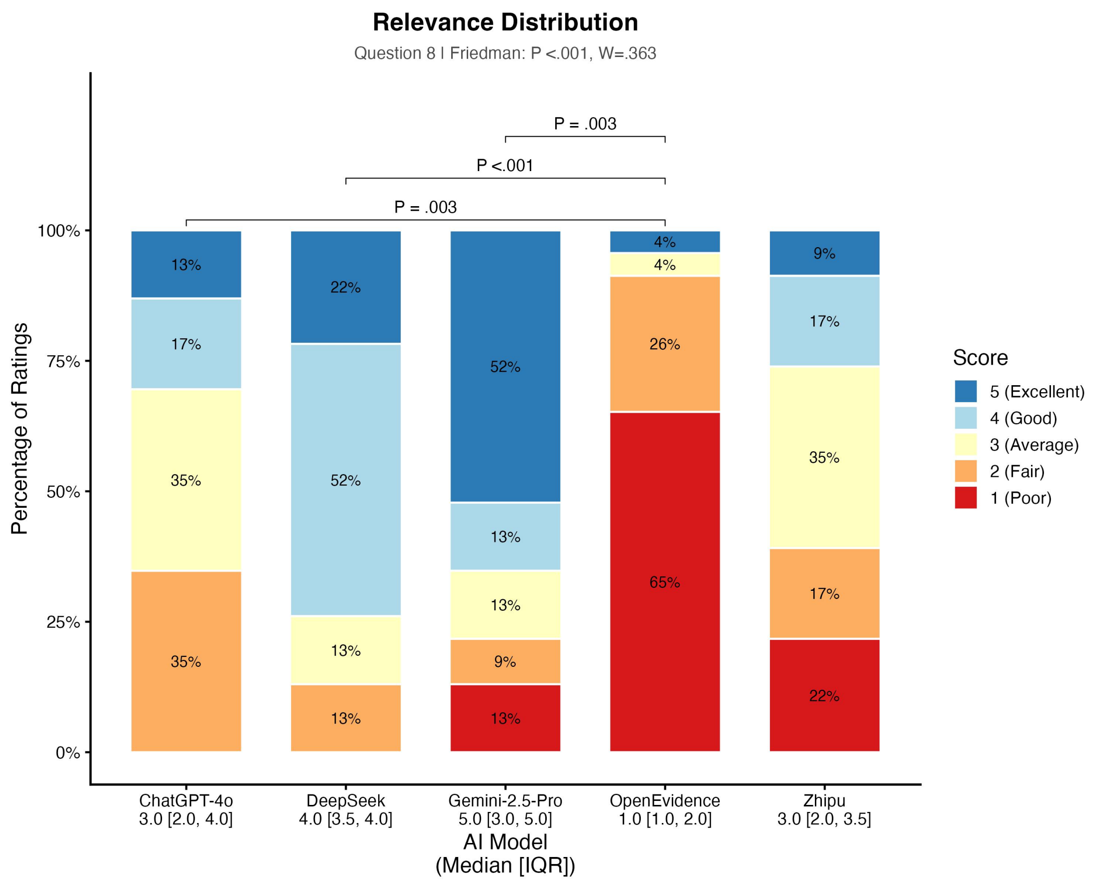
Relevance Distribution
Question 8 | Friedman: P <.001, W=.363
P = .003
P <.001
P = .003
Percentage of Ratings
100%
75%
50%
25%
0%
13%
17%
35%
35%
22%
52%
13%
13%
52%
13%
13%
9%
13%
4%
4%
26%
65%
9%
17%
35%
17%
22%
ChatGPT-4o
3.0 [2.0, 4.0]
DeepSeek
4.0 [3.5, 4.0]
Gemini-2.5-Pro
5.0 [3.0, 5.0]
OpenEvidence
1.0 [1.0, 2.0]
Zhipu
3.0 [2.0, 3.5]
AI Model
(Median [IQR])
Score
5 (Excellent)
4 (Good)
3 (Average)
2 (Fair)
1 (Poor)

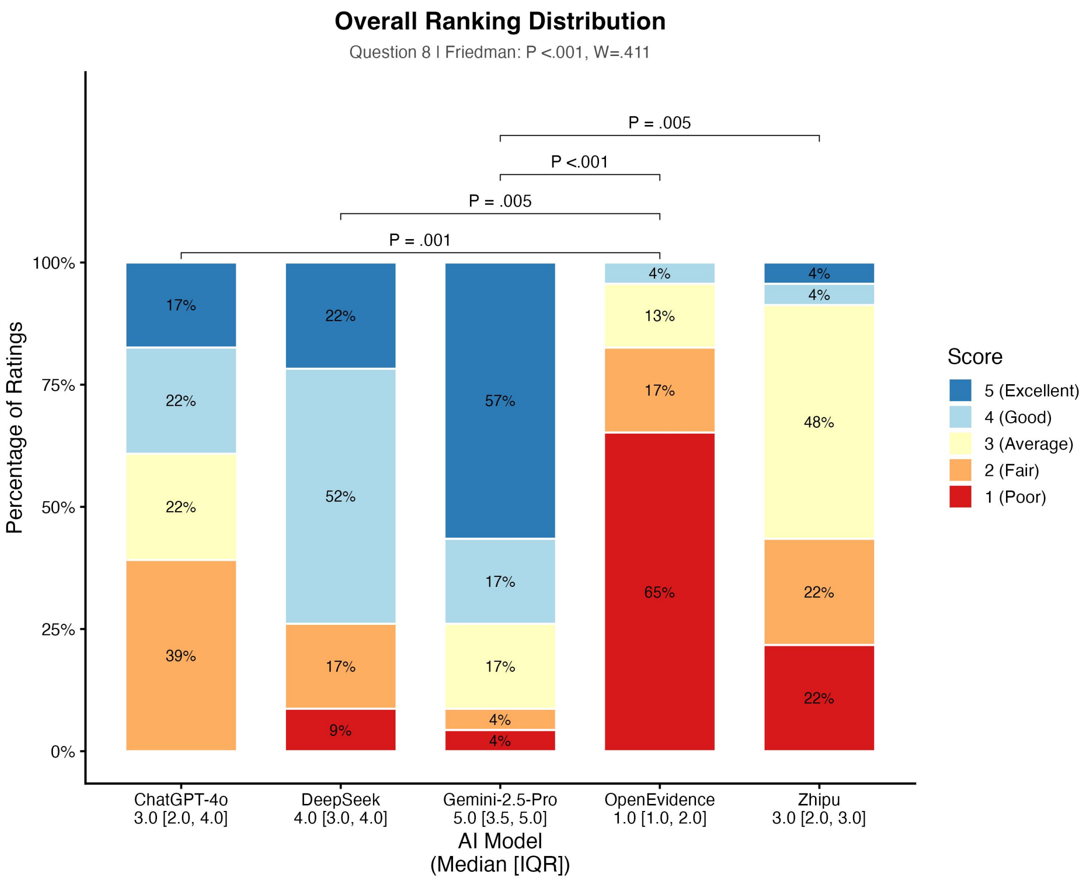
Overall Ranking Distribution
Question 8 I Friedman: P <.001, W=.411
P = .005
P <.001
P = .005
P = .001
Percentage of Ratings
100%
75%
50%
25%
0%
17%
22%
22%
39%
22%
52%
17%
9%
57%
17%
17%
4%
4%
4%
13%
17%
65%
4%
4%
48%
22%
22%
Score
5 (Excellent)
4 (Good)
3 (Average)
2 (Fair)
1 (Poor)
ChatGPT-4o
3.0 [2.0, 4.0]
DeepSeek
4.0 [3.0, 4.0]
Gemini-2.5-Pro
5.0 [3.5, 5.0]
OpenEvidence
1.0 [1.0, 2.0]
Zhipu
3.0 [2.0, 3.0]
AI Model
(Median [IQR])

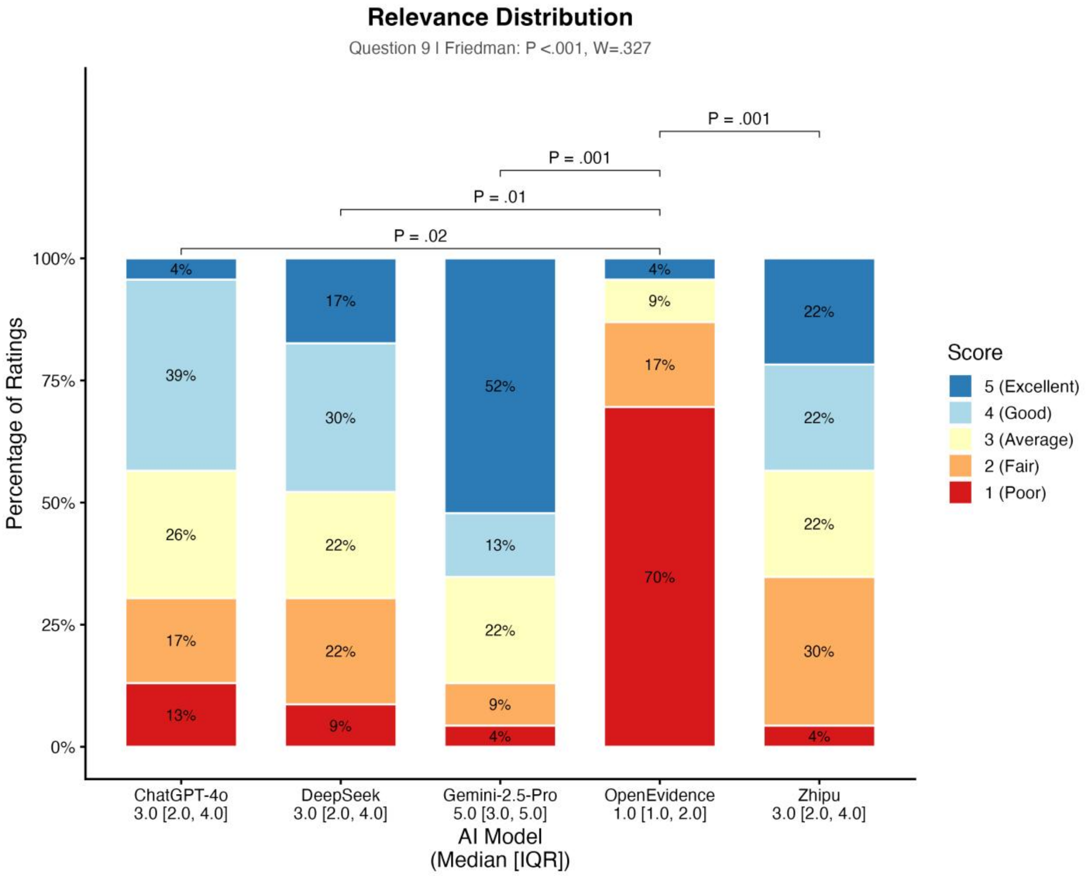
Relevance Distribution
Question 9 I Friedman: P <.001, W=.327
P = .001
P = .001
P = .01
P = .02
Percentage of Ratings
100%
75%
50%
25%
0%
4%
39%
26%
17%
13%
17%
30%
22%
22%
9%
52%
13%
22%
9%
4%
4%
9%
17%
70%
22%
22%
22%
30%
4%
Score
5 (Excellent)
4 (Good)
3 (Average)
2 (Fair)
1 (Poor)
ChatGPT-4o
3.0 [2.0, 4.0]
DeepSeek
3.0 [2.0, 4.0]
Gemini-2.5-Pro
5.0 [3.0, 5.0]
OpenEvidence
1.0 [1.0, 2.0]
Zhipu
3.0 [2.0, 4.0]
AI Model
(Median [IQR])

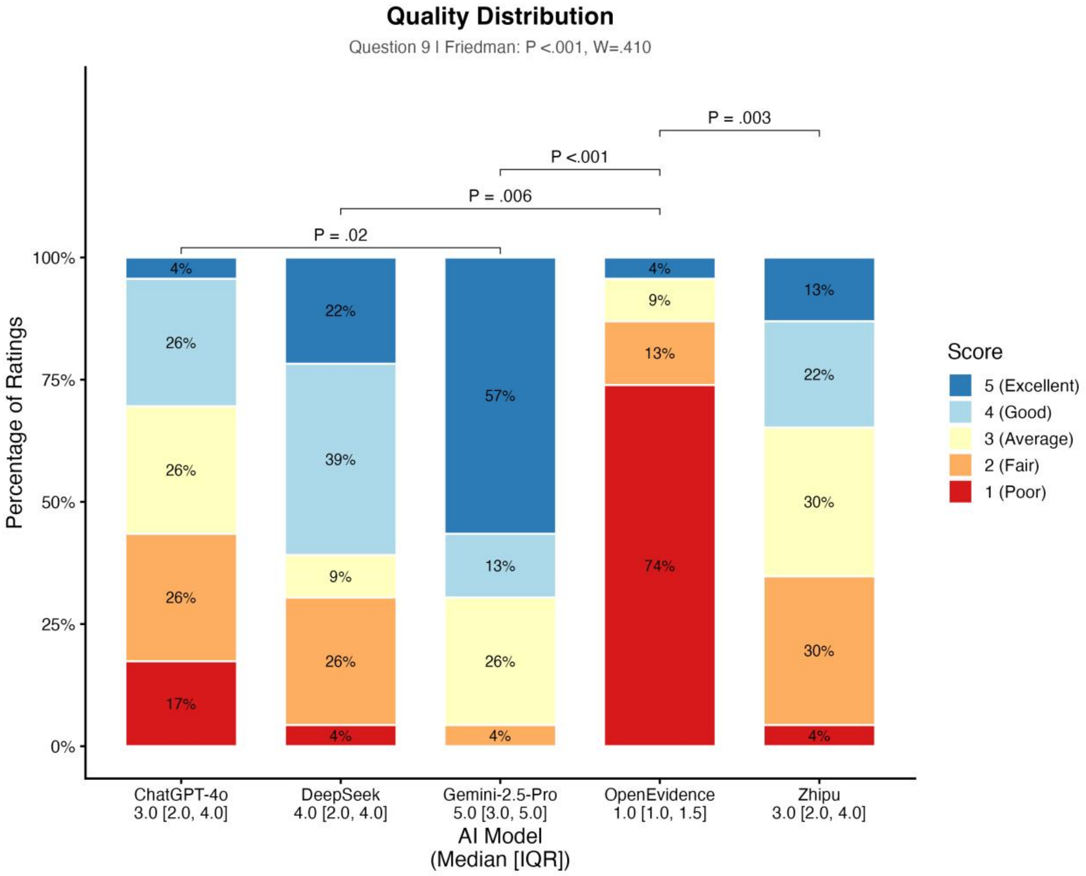
Quality Distribution
Question 9 | Friedman: P <.001, W=.410
P = .003
P <.001
P = .006
P = .02
Percentage of Ratings
100%
75%
50%
25%
0%
4%
26%
26%
26%
17%
22%
39%
9%
26%
4%
57%
13%
26%
4%
4%
9%
13%
74%
13%
22%
30%
30%
4%
ChatGPT-4o
3.0 [2.0, 4.0]
DeepSeek
4.0 [2.0, 4.0]
Gemini-2.5-Pro
5.0 [3.0, 5.0]
OpenEvidence
1.0 [1.0, 1.5]
Zhipu
3.0 [2.0, 4.0]
AI Model
(Median [IQR])
Score
5 (Excellent)
4 (Good)
3 (Average)
2 (Fair)
1 (Poor)

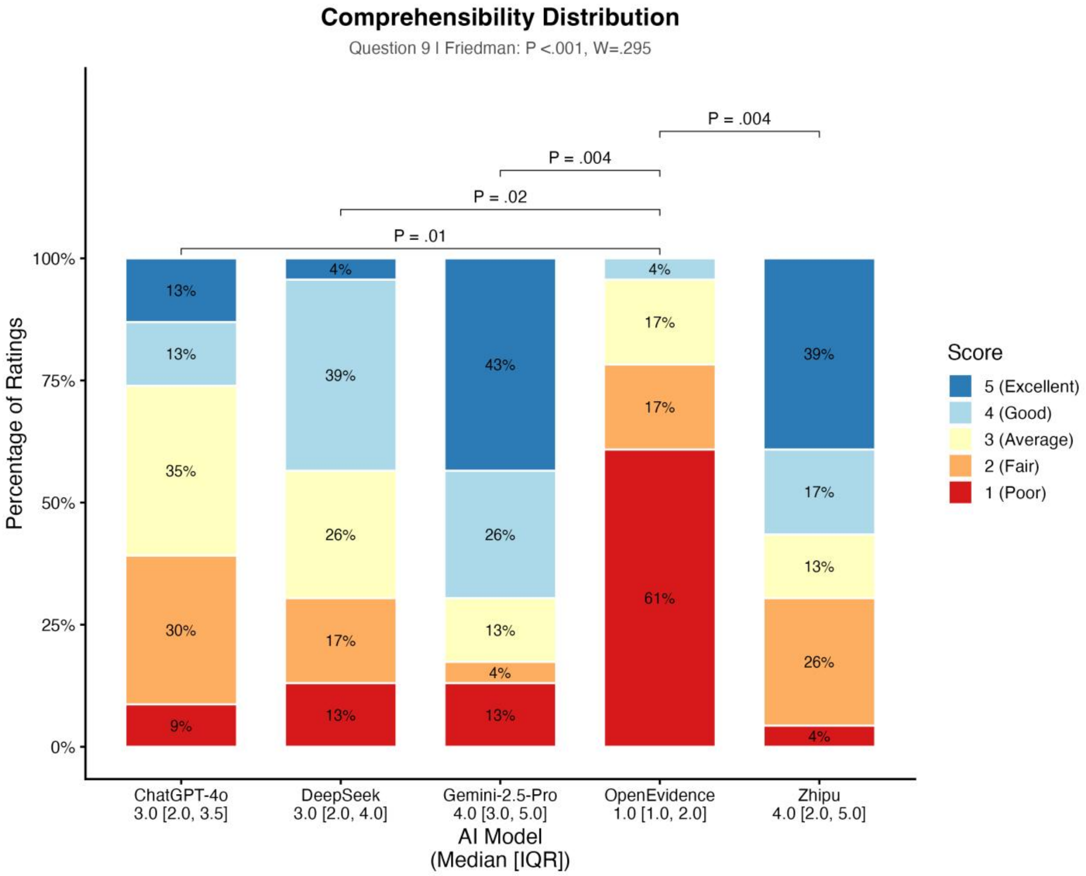
Comprehensibility Distribution
Question 9 | Friedman: P <.001, W=.295
P = .004
P = .004
P = .02
P = .01
Percentage of Ratings
100%
75%
50%
25%
0%
13%
13%
35%
30%
9%
4%
39%
26%
17%
13%
43%
26%
13%
4%
13%
4%
17%
17%
61%
39%
17%
13%
26%
4%
ChatGPT-4o
3.0 [2.0, 3.5]
DeepSeek
3.0 [2.0, 4.0]
Gemini-2.5-Pro
4.0 [3.0, 5.0]
OpenEvidence
1.0 [1.0, 2.0]
Zhipu
4.0 [2.0, 5.0]
AI Model
(Median [IQR])
Score
5 (Excellent)
4 (Good)
3 (Average)
2 (Fair)
1 (Poor)

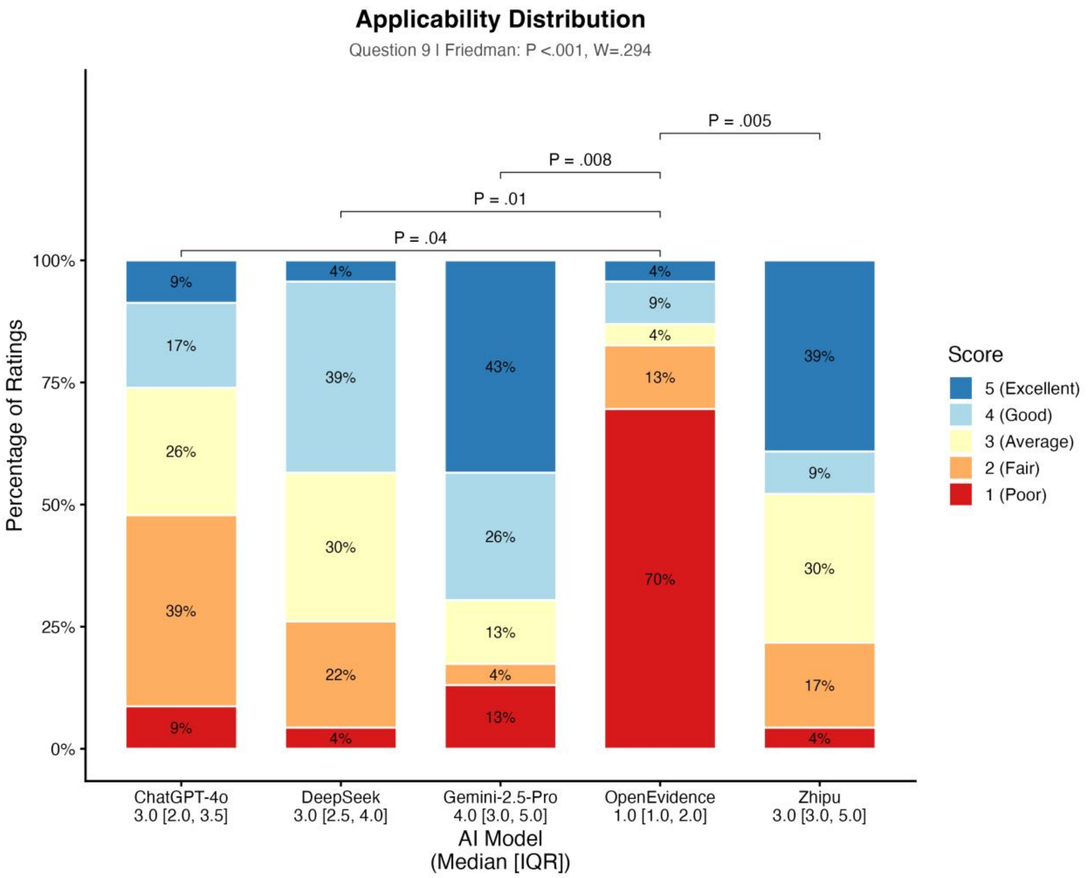
Applicability Distribution
Question 9 | Friedman: P <.001, W=.294
P = .005
P = .008
P = .01
P = .04
Percentage of Ratings
100%
75%
50%
25%
0%
ChatGPT-4o: 9%, 17%, 26%, 39%, 9%
DeepSeek: 4%, 39%, 30%, 22%, 4%
Gemini-2.5-Pro: 43%, 26%, 13%, 4%, 13%
OpenEvidence: 4%, 9%, 4%, 13%, 70%
Zhipu: 39%, 9%, 30%, 17%, 4%
ChatGPT-4o
3.0 [2.0, 3.5]
DeepSeek
3.0 [2.5, 4.0]
Gemini-2.5-Pro
4.0 [3.0, 5.0]
OpenEvidence
1.0 [1.0, 2.0]
Zhipu
3.0 [3.0, 5.0]
AI Model
(Median [IQR])
Score
5 (Excellent)
4 (Good)
3 (Average)
2 (Fair)
1 (Poor)

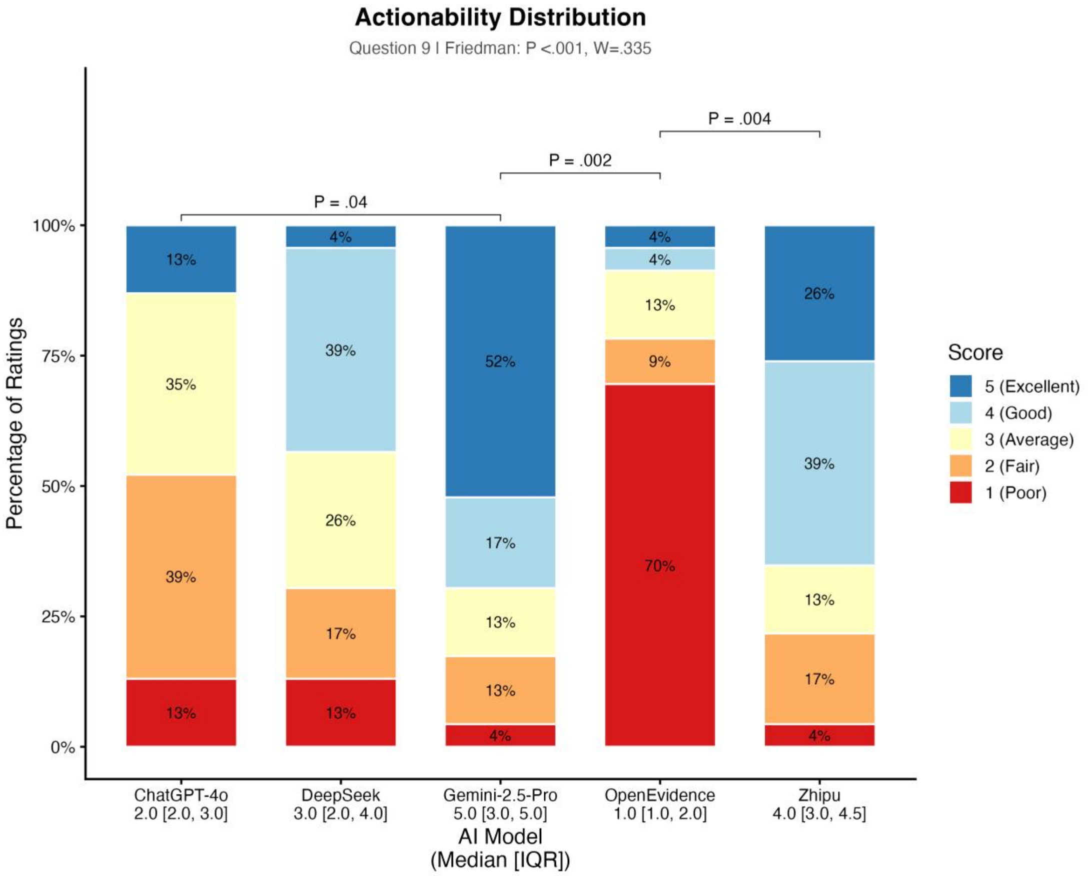
Actionability Distribution
Question 9 | Friedman: P <.001, W=.335
P = .004
P = .002
P = .04
Percentage of Ratings
100%
75%
50%
25%
0%
ChatGPT-4o: 13%, 35%, 39%, 13%
DeepSeek: 4%, 39%, 26%, 17%, 13%
Gemini-2.5-Pro: 52%, 17%, 13%, 13%, 4%
OpenEvidence: 4%, 4%, 13%, 9%, 70%
Zhipu: 26%, 39%, 13%, 17%, 4%
ChatGPT-4o
2.0 [2.0, 3.0]
DeepSeek
3.0 [2.0, 4.0]
Gemini-2.5-Pro
5.0 [3.0, 5.0]
OpenEvidence
1.0 [1.0, 2.0]
Zhipu
4.0 [3.0, 4.5]
AI Model
(Median [IQR])
Score
5 (Excellent)
4 (Good)
3 (Average)
2 (Fair)
1 (Poor)

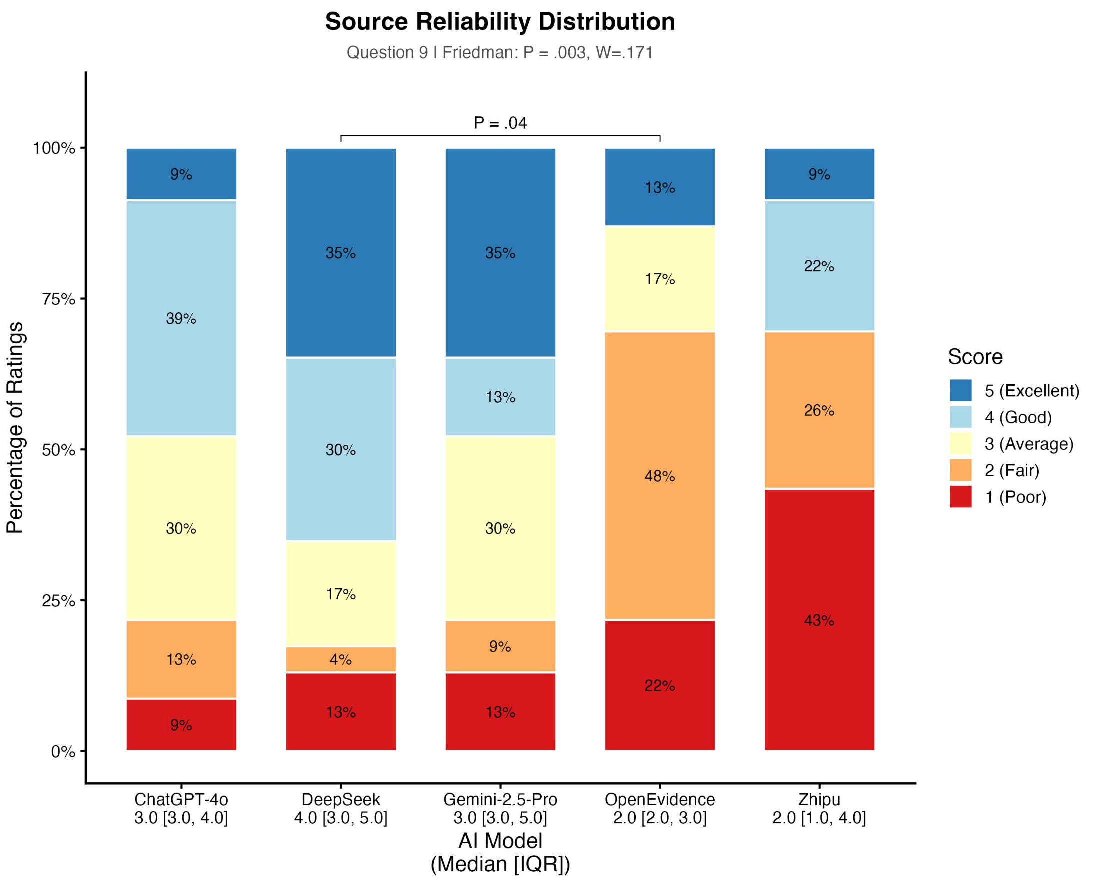
Source Reliability Distribution
Question 9 | Friedman: P = .003, W=.171
P = .04
100%
75%
50%
25%
0%
Percentage of Ratings
9%
39%
30%
13%
9%
35%
30%
17%
4%
13%
35%
13%
30%
9%
13%
13%
17%
48%
22%
9%
22%
26%
43%
ChatGPT-4o
3.0 [3.0, 4.0]
DeepSeek
4.0 [3.0, 5.0]
Gemini-2.5-Pro
3.0 [3.0, 5.0]
OpenEvidence
2.0 [2.0, 3.0]
Zhipu
2.0 [1.0, 4.0]
AI Model
(Median [IQR])
Score
5 (Excellent)
4 (Good)
3 (Average)
2 (Fair)
1 (Poor)

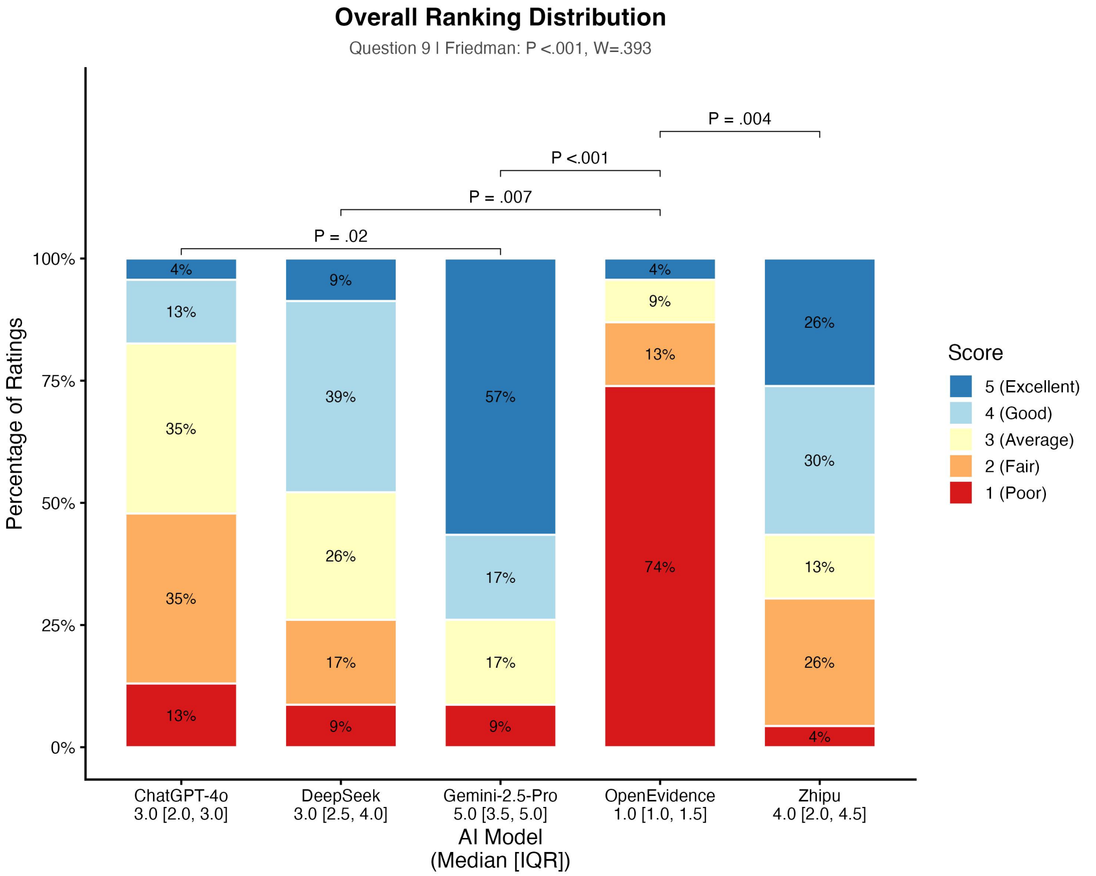
Overall Ranking Distribution
Question 9 | Friedman: P <.001, W=.393
P = .004
P <.001
P = .007
P = .02
100%
75%
50%
25%
0%
Percentage of Ratings
4%
13%
35%
35%
13%
9%
39%
26%
17%
9%
57%
17%
17%
9%
4%
9%
13%
74%
26%
30%
13%
26%
4%
ChatGPT-4o
3.0 [2.0, 3.0]
DeepSeek
3.0 [2.5, 4.0]
Gemini-2.5-Pro
5.0 [3.5, 5.0]
OpenEvidence
1.0 [1.0, 1.5]
Zhipu
4.0 [2.0, 4.5]
AI Model
(Median [IQR])
Score
5 (Excellent)
4 (Good)
3 (Average)
2 (Fair)
1 (Poor)

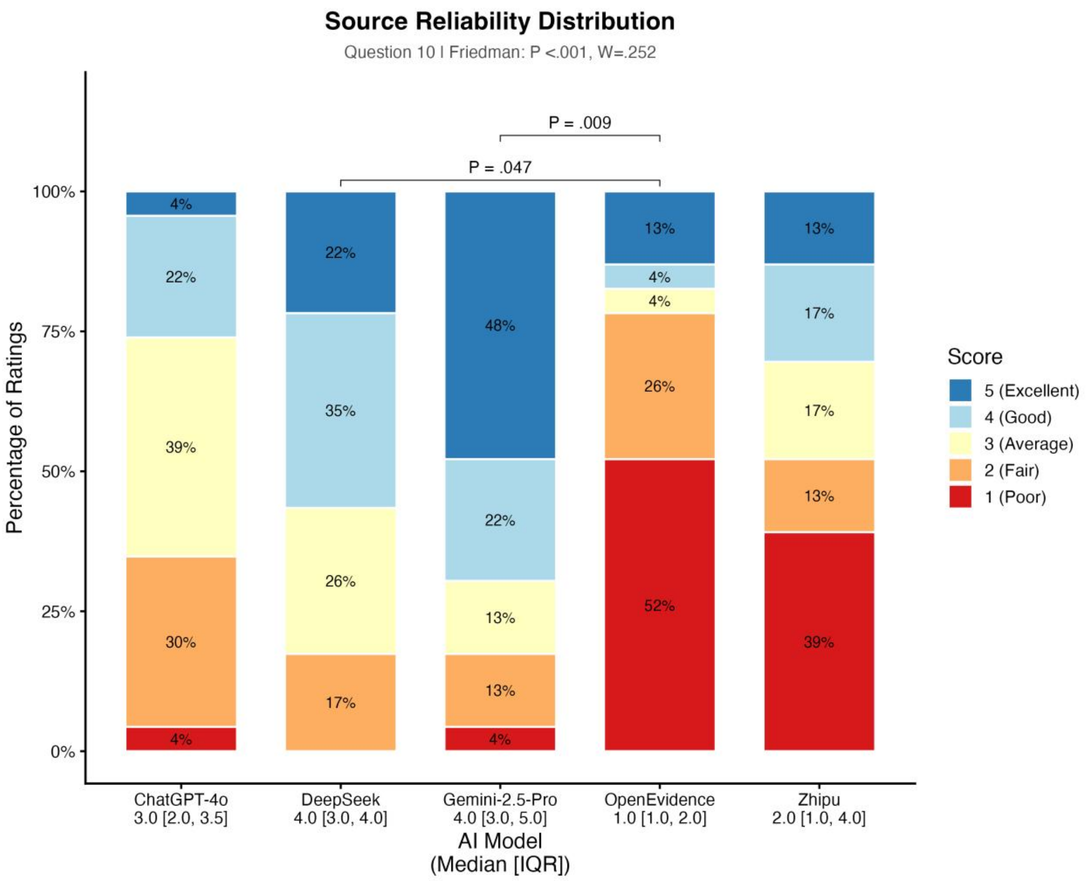

Source Reliability Distribution
Question 10 | Friedman: P <.001, W=.252
P = .009
P = .047
Percentage of Ratings
100%
75%
50%
25%
0%
ChatGPT-4o 3.0 [2.0, 3.5]: 4%, 22%, 39%, 30%, 4%
DeepSeek 4.0 [3.0, 4.0]: 22%, 35%, 26%, 17%
Gemini-2.5-Pro 4.0 [3.0, 5.0]: 48%, 22%, 13%, 13%, 4%
OpenEvidence 1.0 [1.0, 2.0]: 13%, 4%, 4%, 26%, 52%
Zhipu 2.0 [1.0, 4.0]: 13%, 17%, 17%, 13%, 39%
AI Model
(Median [IQR])
Score
5 (Excellent)
4 (Good)
3 (Average)
2 (Fair)
1 (Poor)

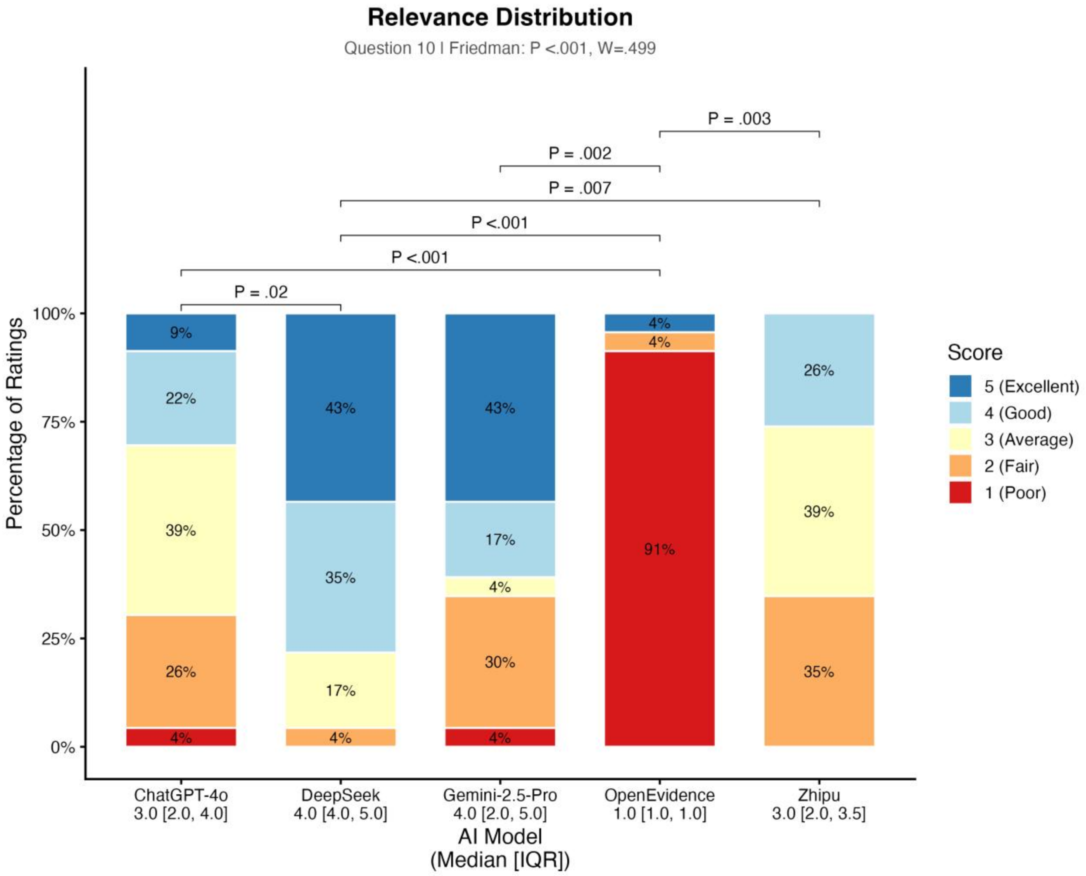

Relevance Distribution
Question 10 | Friedman: P <.001, W=.499
P = .003
P = .002
P = .007
P <.001
P <.001
P = .02
Percentage of Ratings
100%
75%
50%
25%
0%
ChatGPT-4o 3.0 [2.0, 4.0]: 9%, 22%, 39%, 26%, 4%
DeepSeek 4.0 [4.0, 5.0]: 43%, 35%, 17%, 4%
Gemini-2.5-Pro 4.0 [2.0, 5.0]: 43%, 17%, 4%, 30%, 4%
OpenEvidence 1.0 [1.0, 1.0]: 4%, 4%, 91%
Zhipu 3.0 [2.0, 3.5]: 26%, 39%, 35%
AI Model
(Median [IQR])
Score
5 (Excellent)
4 (Good)
3 (Average)
2 (Fair)
1 (Poor)

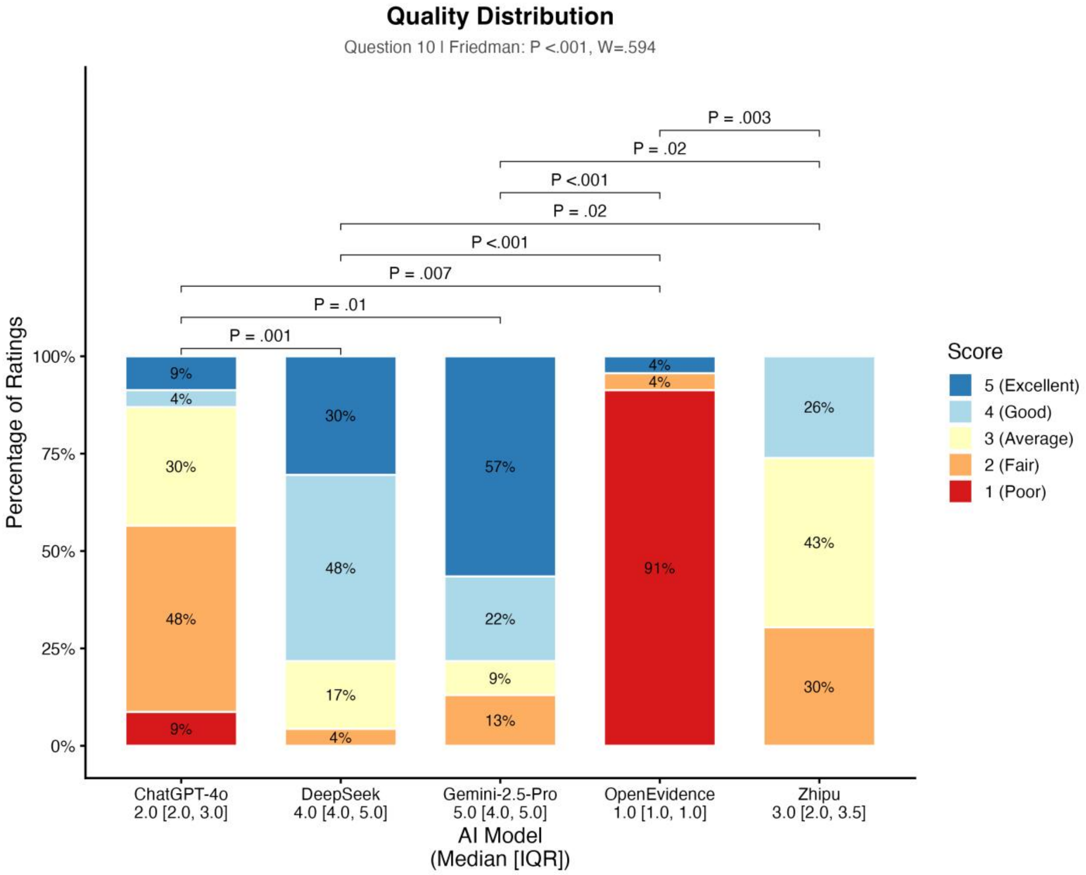
Quality Distribution
Question 10 | Friedman: P <.001, W=.594
P = .003
P = .02
P <.001
P = .02
P <.001
P = .007
P = .01
P = .001
Percentage of Ratings
100%
75%
50%
25%
0%
9%
4%
30%
48%
9%
30%
48%
17%
4%
57%
22%
9%
13%
4%
4%
91%
26%
43%
30%
ChatGPT-4o
2.0 [2.0, 3.0]
DeepSeek
4.0 [4.0, 5.0]
Gemini-2.5-Pro
5.0 [4.0, 5.0]
OpenEvidence
1.0 [1.0, 1.0]
Zhipu
3.0 [2.0, 3.5]
AI Model
(Median [IQR])
Score
5 (Excellent)
4 (Good)
3 (Average)
2 (Fair)
1 (Poor)

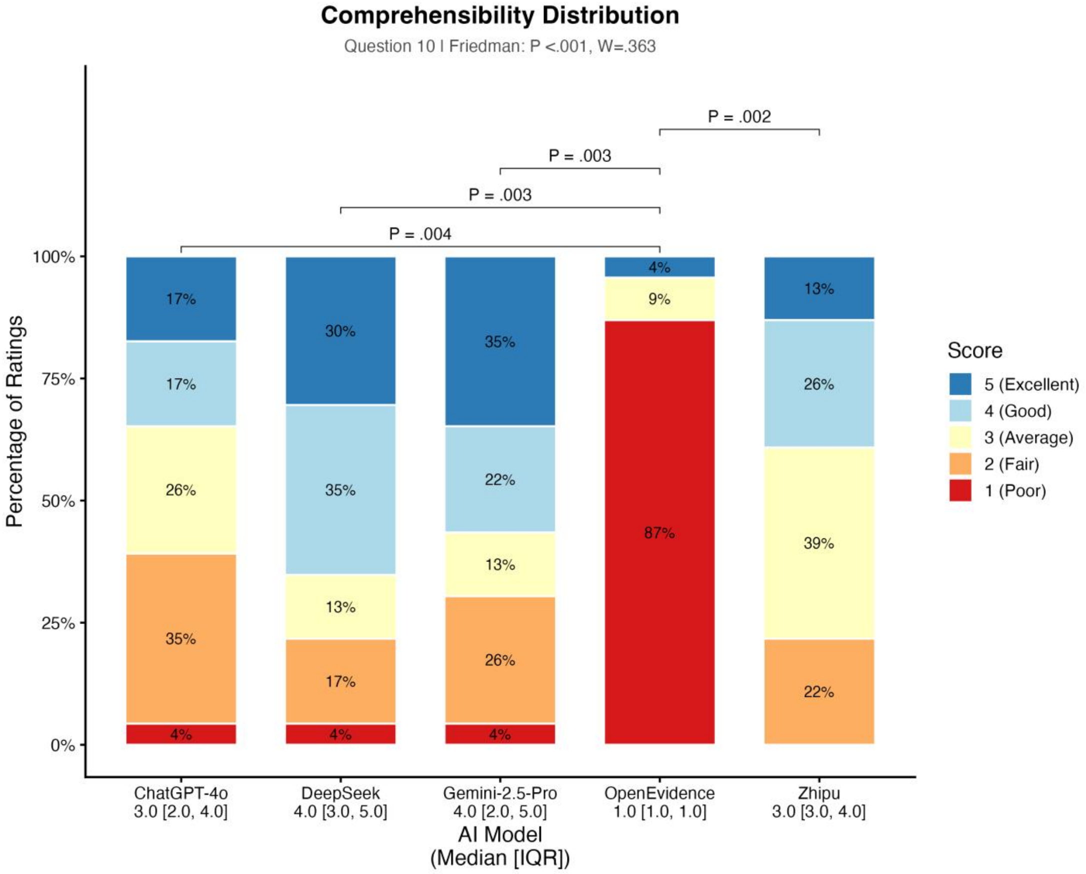
Comprehensibility Distribution
Question 10 | Friedman: P <.001, W=.363
P = .002
P = .003
P = .003
P = .004
Percentage of Ratings
100%
75%
50%
25%
0%
17%
17%
26%
35%
4%
30%
35%
13%
17%
4%
35%
22%
13%
26%
4%
4%
9%
87%
13%
26%
39%
22%
ChatGPT-4o
3.0 [2.0, 4.0]
DeepSeek
4.0 [3.0, 5.0]
Gemini-2.5-Pro
4.0 [2.0, 5.0]
OpenEvidence
1.0 [1.0, 1.0]
Zhipu
3.0 [3.0, 4.0]
AI Model
(Median [IQR])
Score
5 (Excellent)
4 (Good)
3 (Average)
2 (Fair)
1 (Poor)

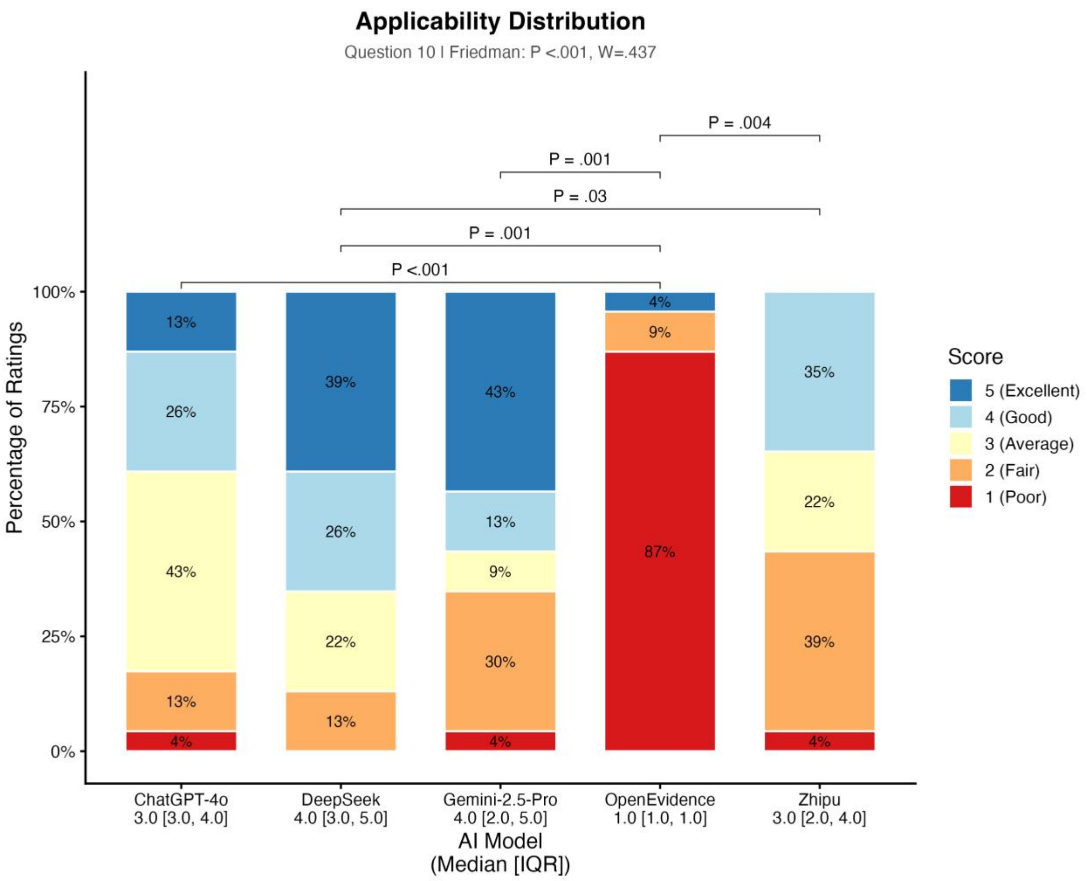
Applicability Distribution
Question 10 | Friedman: P <.001, W=.437
P = .004
P = .001
P = .03
P = .001
P <.001
Percentage of Ratings
100%
75%
50%
25%
0%
13%
26%
43%
13%
4%
39%
26%
22%
13%
43%
13%
9%
30%
4%
4%
9%
87%
35%
22%
39%
4%
Score
5 (Excellent)
4 (Good)
3 (Average)
2 (Fair)
1 (Poor)
ChatGPT-4o
3.0 [3.0, 4.0]
DeepSeek
4.0 [3.0, 5.0]
Gemini-2.5-Pro
4.0 [2.0, 5.0]
OpenEvidence
1.0 [1.0, 1.0]
Zhipu
3.0 [2.0, 4.0]
AI Model
(Median [IQR])

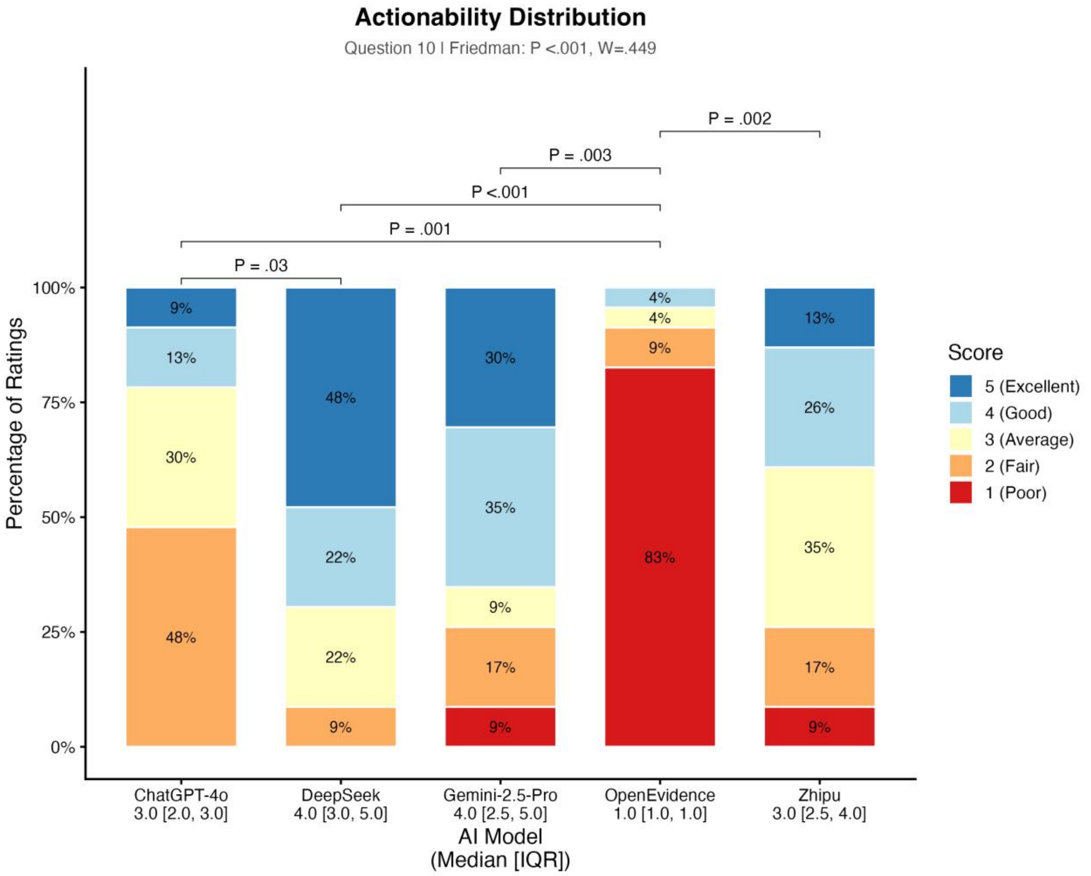
Actionability Distribution
Question 10 | Friedman: P <.001, W=.449
P = .002
P = .003
P <.001
P = .001
P = .03
Percentage of Ratings
100%
75%
50%
25%
0%
9%
13%
30%
48%
48%
22%
22%
9%
30%
35%
9%
17%
9%
4%
4%
9%
83%
13%
26%
35%
17%
9%
Score
5 (Excellent)
4 (Good)
3 (Average)
2 (Fair)
1 (Poor)
ChatGPT-4o
3.0 [2.0, 3.0]
DeepSeek
4.0 [3.0, 5.0]
Gemini-2.5-Pro
4.0 [2.5, 5.0]
OpenEvidence
1.0 [1.0, 1.0]
Zhipu
3.0 [2.5, 4.0]
AI Model
(Median [IQR])

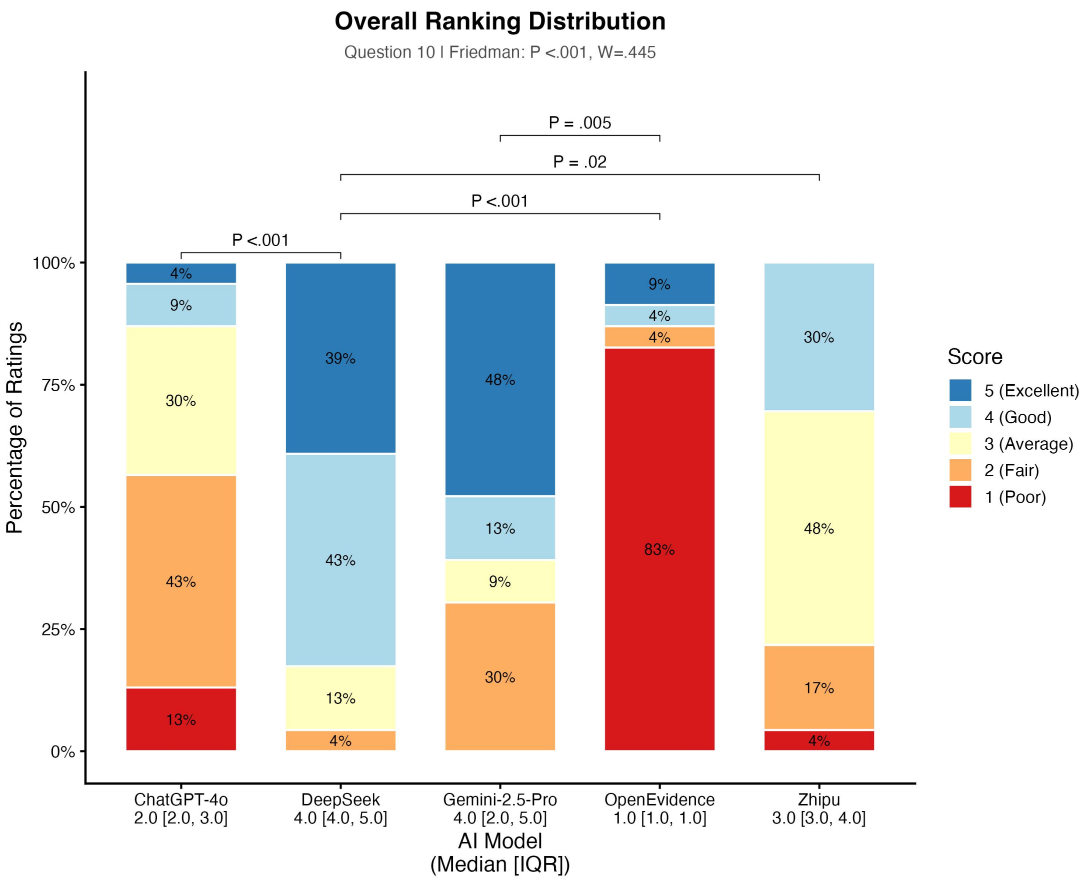
Overall Ranking Distribution
Question 10 | Friedman: P <.001, W=.445
P = .005
P = .02
P <.001
P <.001
Percentage of Ratings
100%
75%
50%
25%
0%
4%
9%
30%
43%
13%
39%
43%
13%
4%
48%
13%
9%
30%
9%
4%
4%
83%
30%
48%
17%
4%
Score
5 (Excellent)
4 (Good)
3 (Average)
2 (Fair)
1 (Poor)
ChatGPT-4o
2.0 [2.0, 3.0]
DeepSeek
4.0 [4.0, 5.0]
Gemini-2.5-Pro
4.0 [2.0, 5.0]
OpenEvidence
1.0 [1.0, 1.0]
Zhipu
3.0 [3.0, 4.0]
AI Model
(Median [IQR])

Violin (Rain-Cloud) Plots: Per-Question Expert Rating Distributions

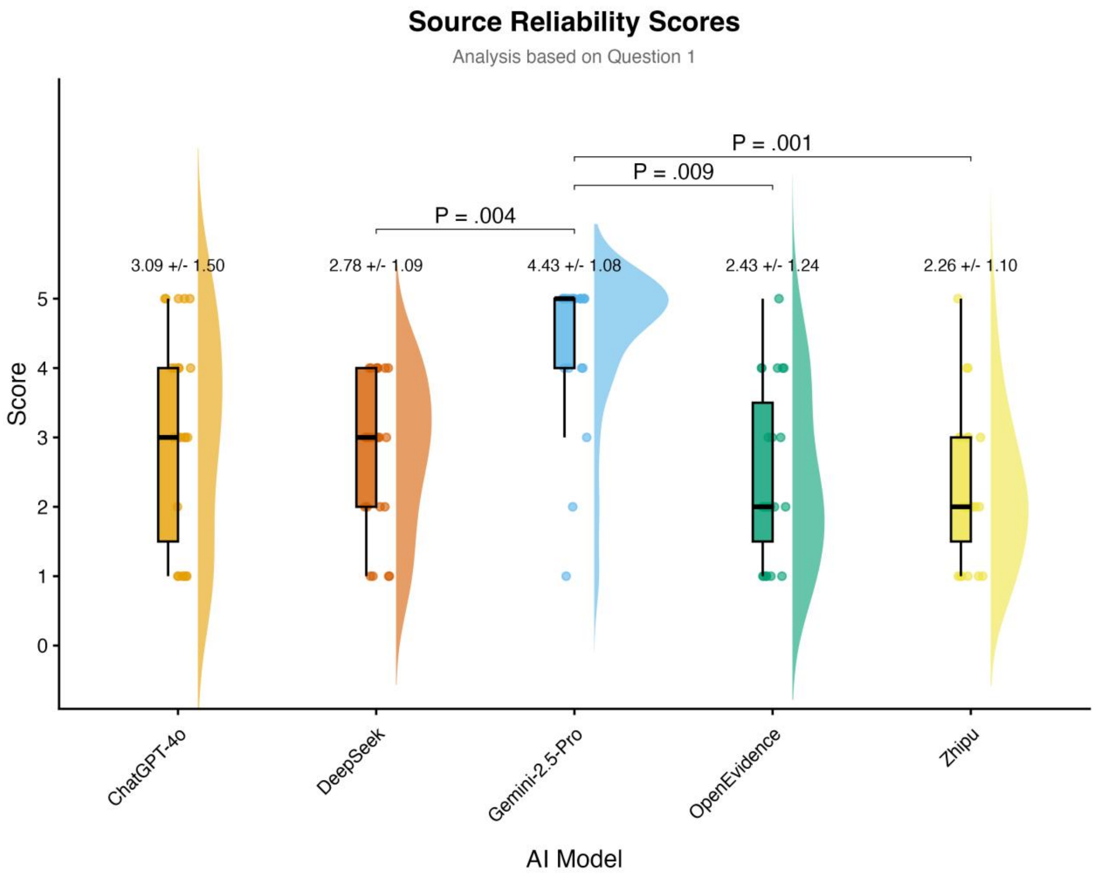

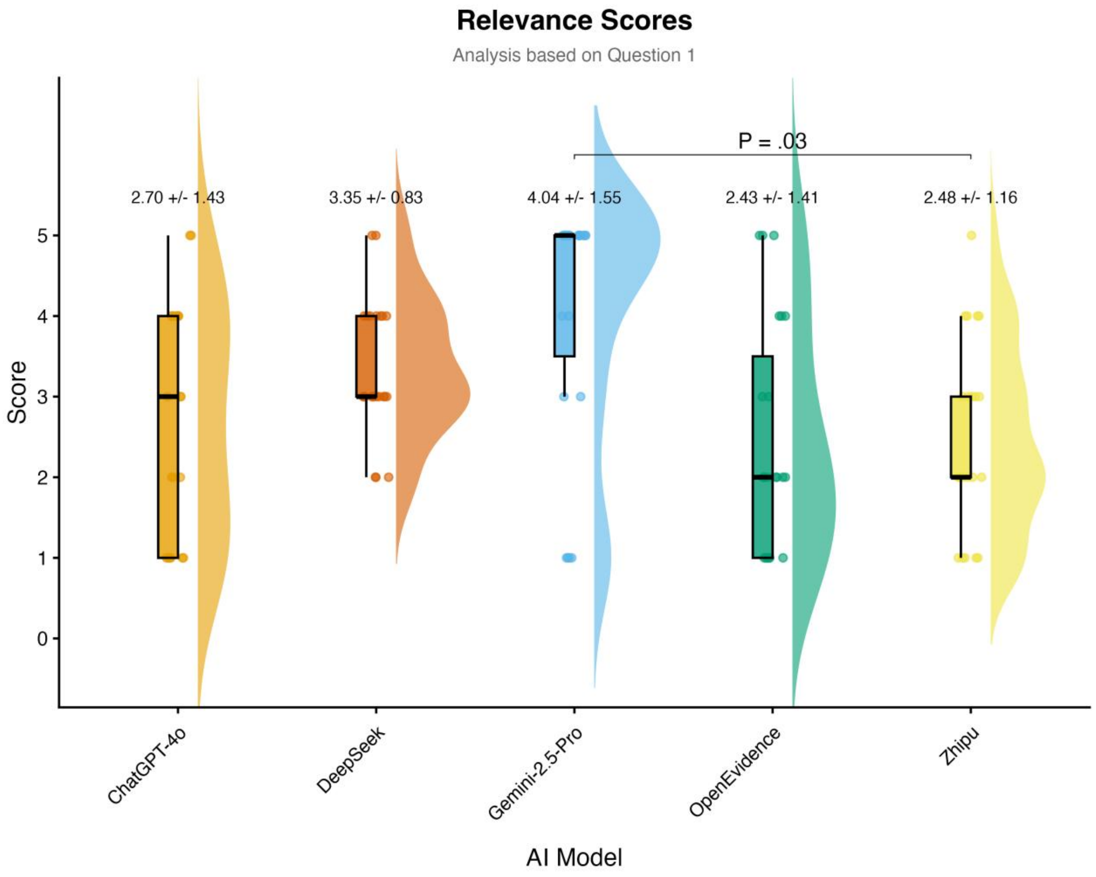

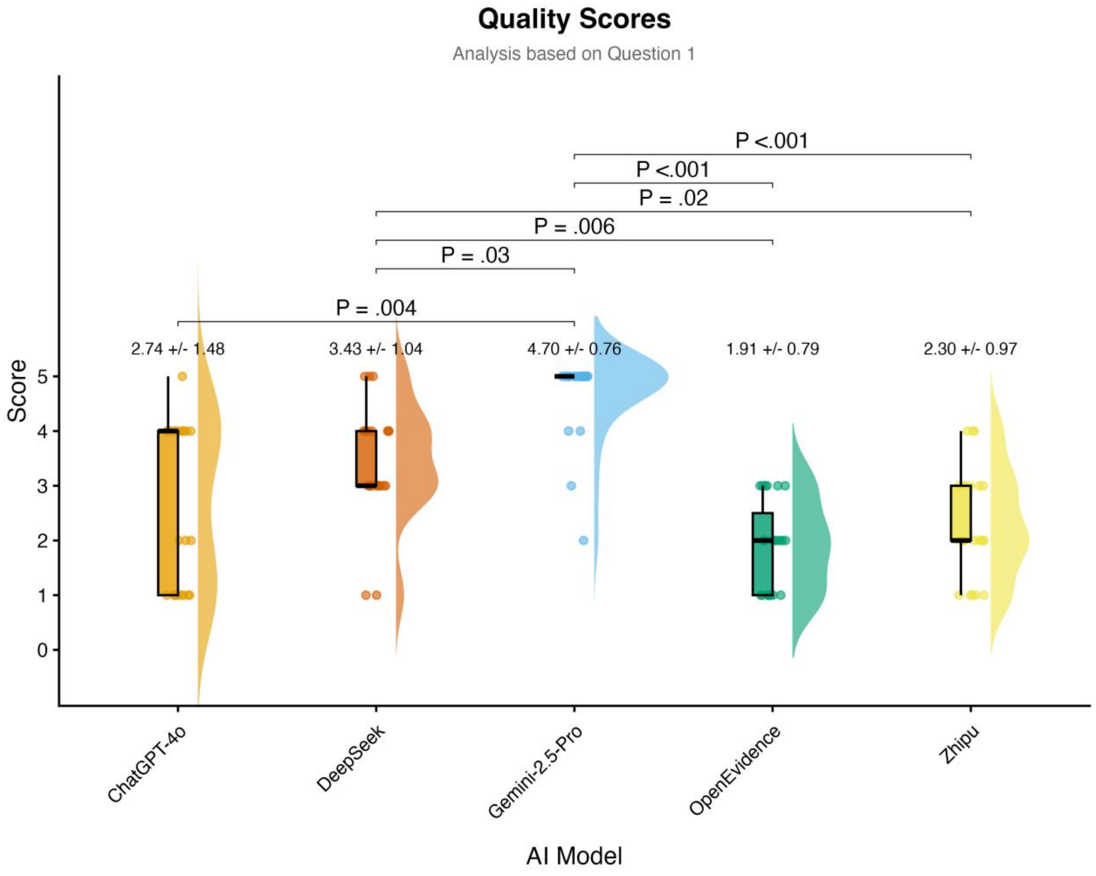
Quality Scores
Analysis based on Question 1
P <.001
P <.001
P = .02
P = .006
P = .03
P = .004
2.74 +/- 1.48
3.43 +/- 1.04
4.70 +/- 0.76
1.91 +/- 0.79
2.30 +/- 0.97
Score
ChatGPT-4o
DeepSeek
Gemini-2.5-Pro
OpenEvidence
Zhipu
AI Model

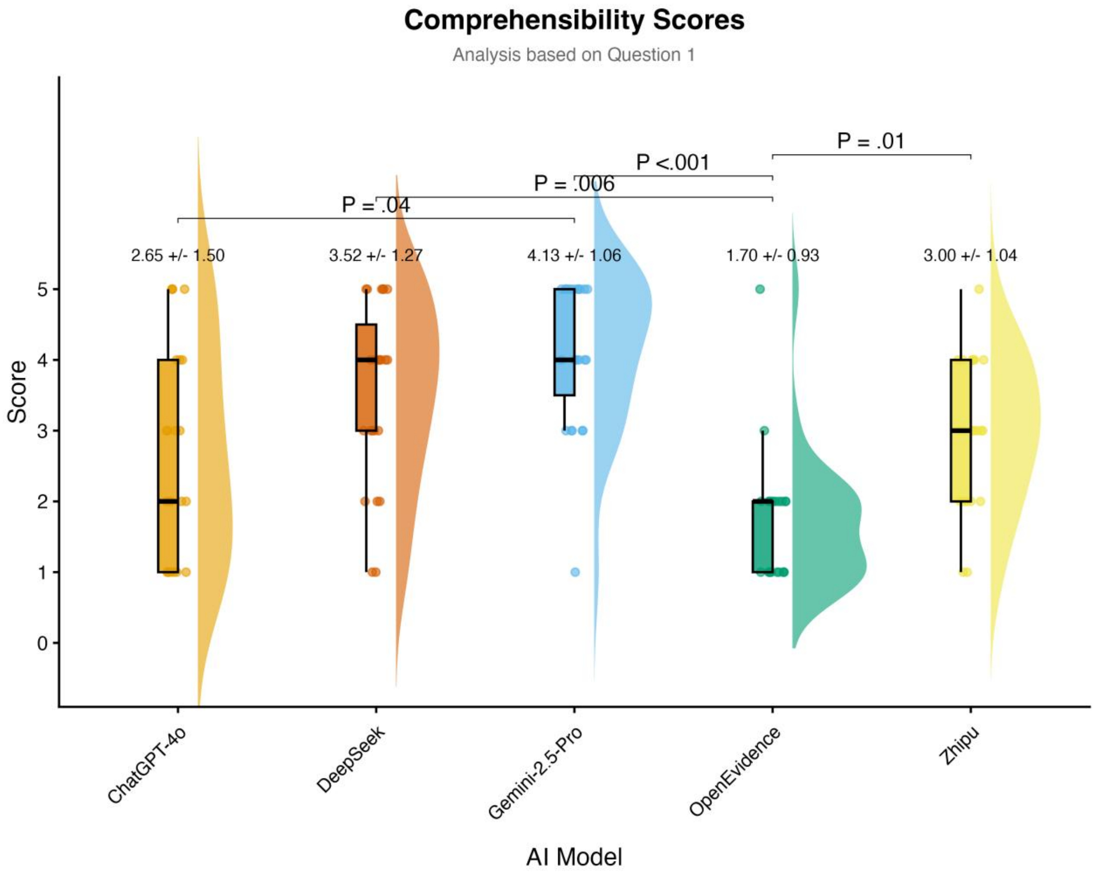
Comprehensibility Scores
Analysis based on Question 1
P = .01
P <.001
P = .006
P = .04
2.65 +/- 1.50
3.52 +/- 1.27
4.13 +/- 1.06
1.70 +/- 0.93
3.00 +/- 1.04
Score
ChatGPT-4o
DeepSeek
Gemini-2.5-Pro
OpenEvidence
Zhipu
AI Model

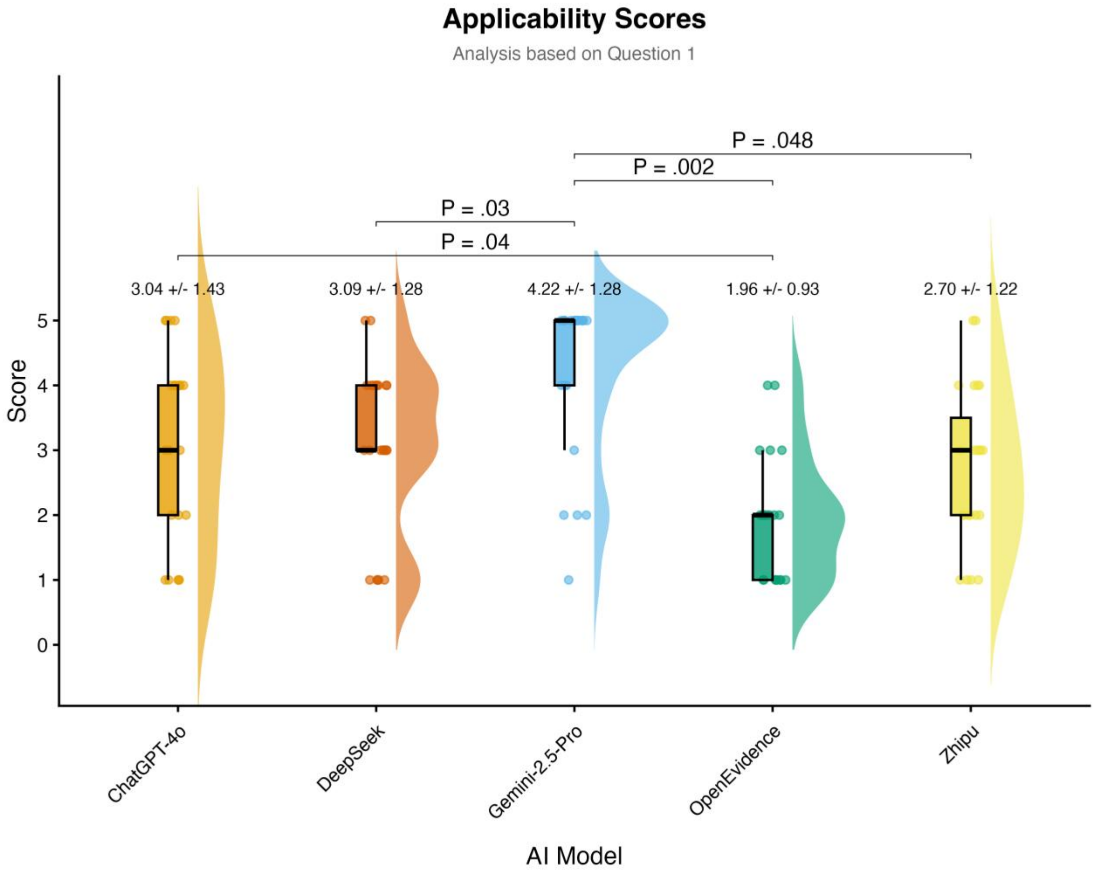
Applicability Scores
Analysis based on Question 1
P = .048
P = .002
P = .03
P = .04
3.04 +/- 1.43
3.09 +/- 1.28
4.22 +/- 1.28
1.96 +/- 0.93
2.70 +/- 1.22
Score
ChatGPT-4o
DeepSeek
Gemini-2.5-Pro
OpenEvidence
Zhipu
AI Model

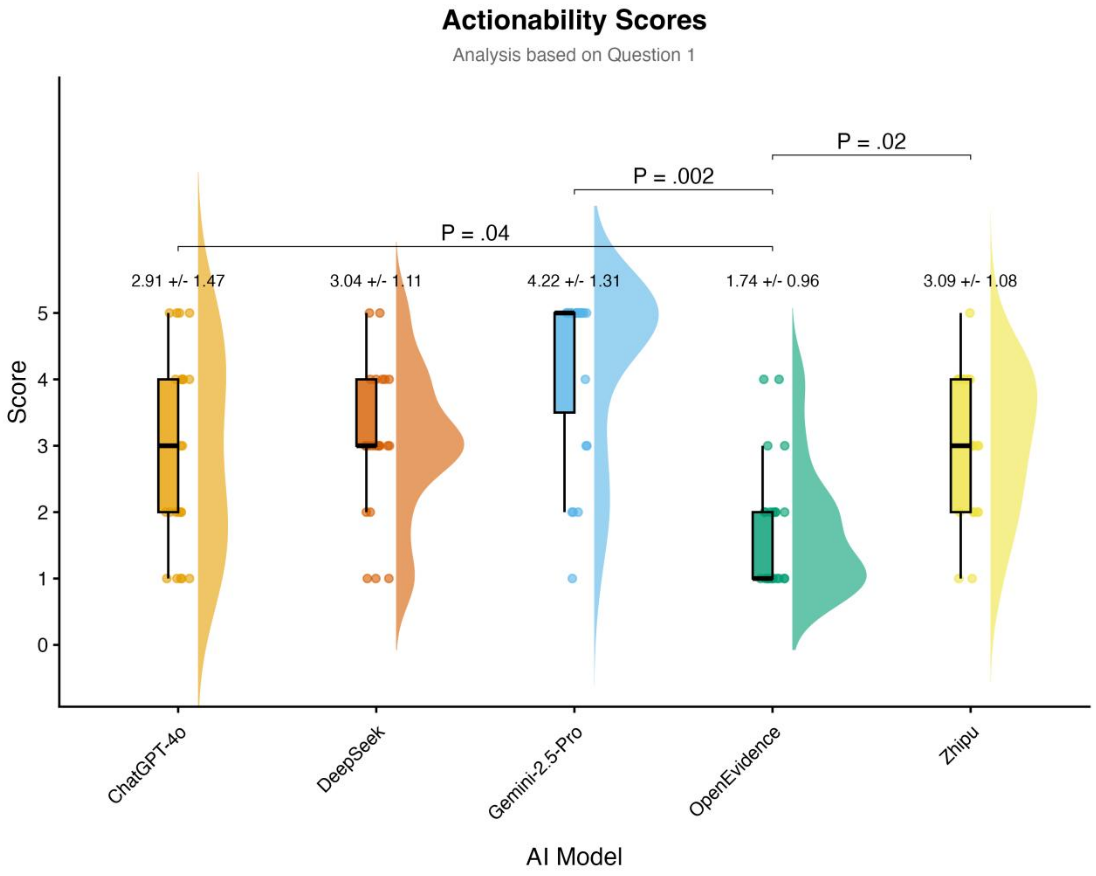
Actionability Scores
Analysis based on Question 1
P = .02
P = .002
P = .04
2.91 +/- 1.47
3.04 +/- 1.11
4.22 +/- 1.31
1.74 +/- 0.96
3.09 +/- 1.08
Score
ChatGPT-4o
DeepSeek
Gemini-2.5-Pro
OpenEvidence
Zhipu
AI Model

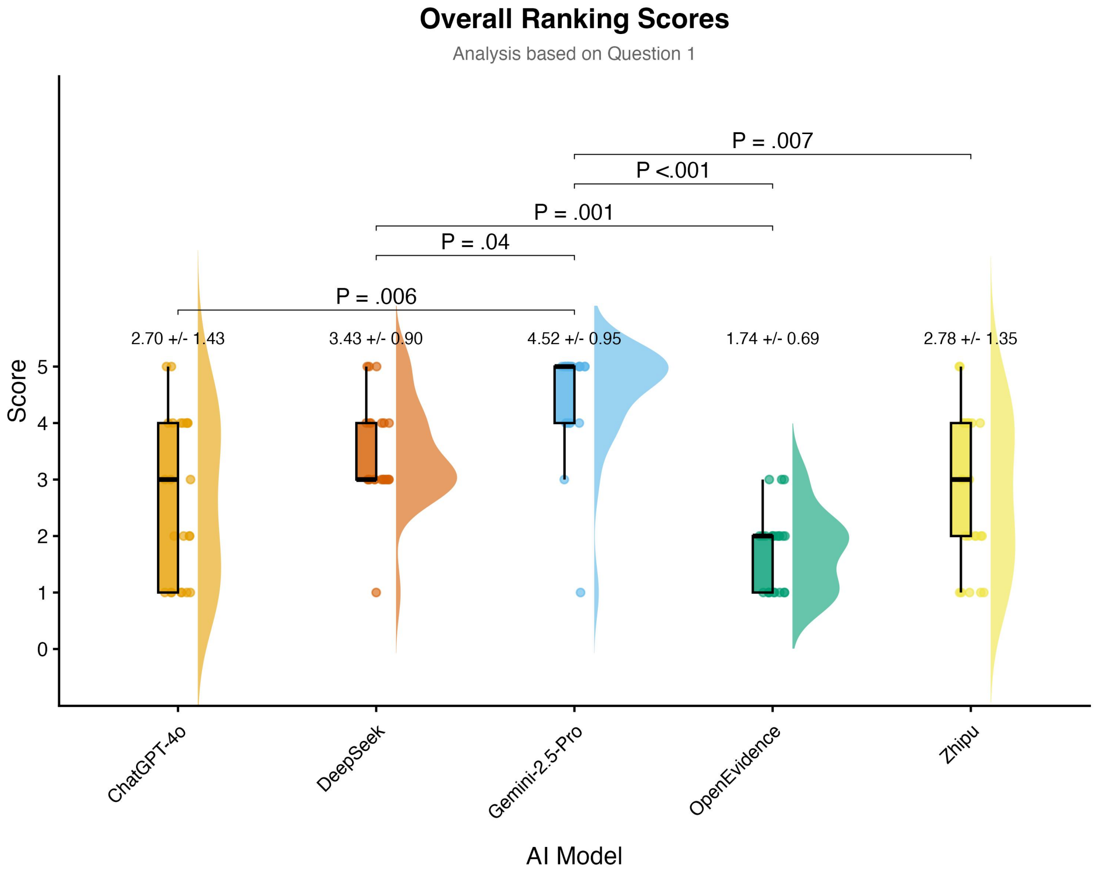

Overall Ranking Scores
Analysis based on Question 1
P = .007
P <.001
P = .001
P = .04
P = .006
2.70 +/- 1.43
3.43 +/- 0.90
4.52 +/- 0.95
1.74 +/- 0.69
2.78 +/- 1.35
Score
5
4
3
2
1
0
ChatGPT-4o
DeepSeek
Gemini-2.5-Pro
OpenEvidence
Zhipu
AI Model

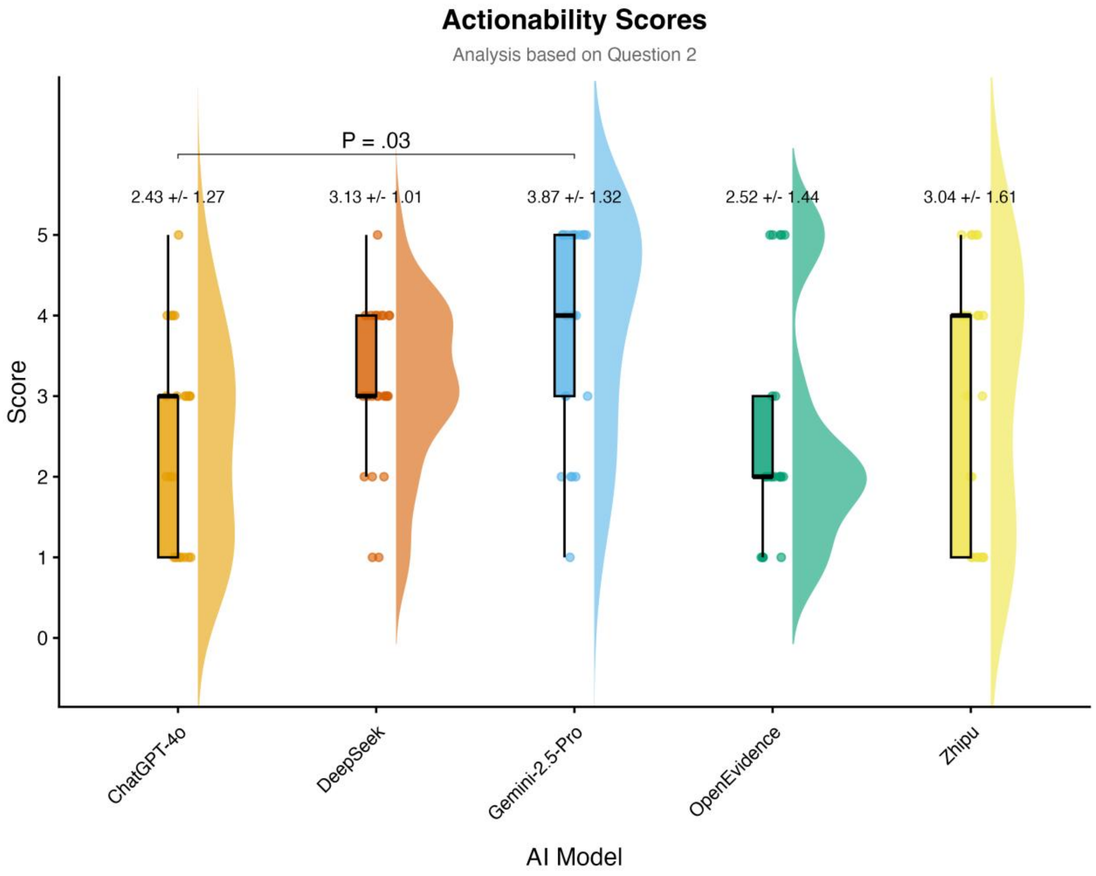

Actionability Scores
Analysis based on Question 2
P = .03
2.43 +/- 1.27
3.13 +/- 1.01
3.87 +/- 1.32
2.52 +/- 1.44
3.04 +/- 1.61
Score
5
4
3
2
1
0
ChatGPT-4o
DeepSeek
Gemini-2.5-Pro
OpenEvidence
Zhipu
AI Model

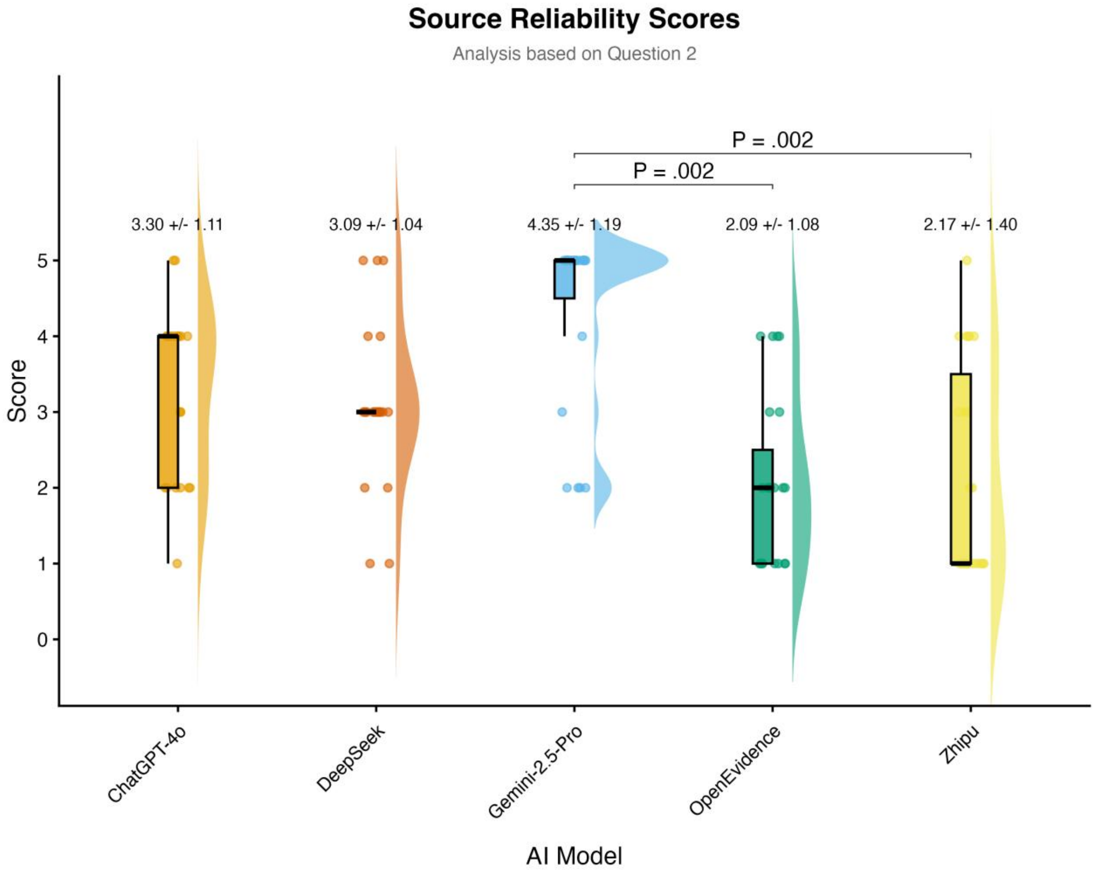
Source Reliability Scores
Analysis based on Question 2
P = .002
P = .002
3.30 +/- 1.11
3.09 +/- 1.04
4.35 +/- 1.19
2.09 +/- 1.08
2.17 +/- 1.40
Score
5
4
3
2
1
0
ChatGPT-4o
DeepSeek
Gemini-2.5-Pro
OpenEvidence
Zhipu
AI Model

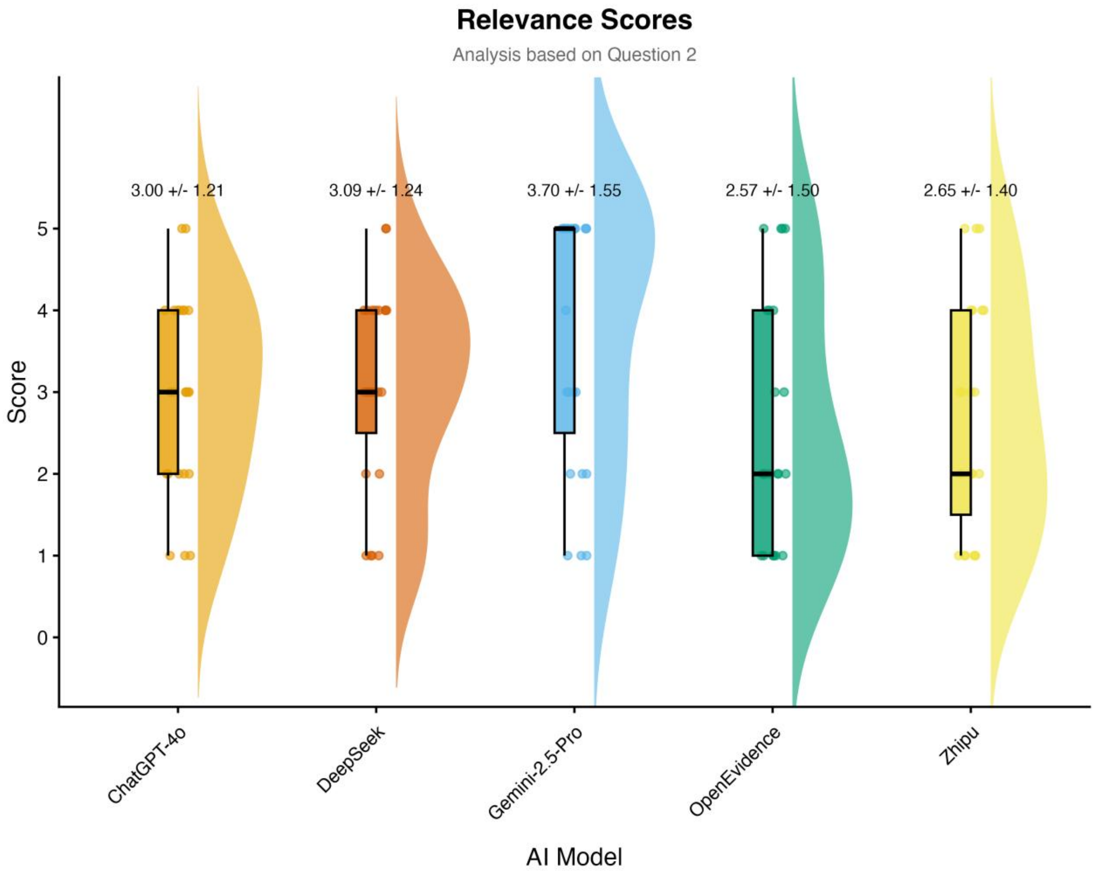
Relevance Scores
Analysis based on Question 2
3.00 +/- 1.21
3.09 +/- 1.24
3.70 +/- 1.55
2.57 +/- 1.50
2.65 +/- 1.40
Score
5
4
3
2
1
0
ChatGPT-4o
DeepSeek
Gemini-2.5-Pro
OpenEvidence
Zhipu
AI Model

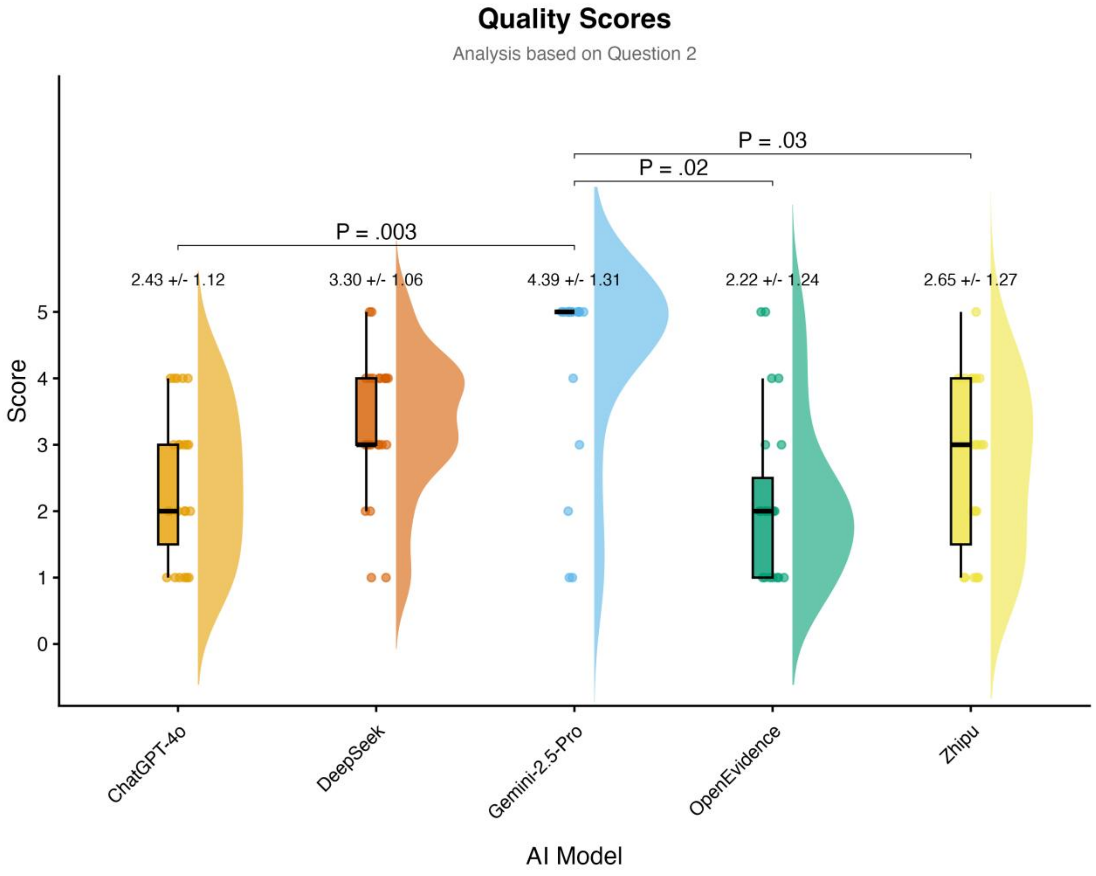
Quality Scores
Analysis based on Question 2
P = .03
P = .02
P = .003
2.43 +/- 1.12
3.30 +/- 1.06
4.39 +/- 1.31
2.22 +/- 1.24
2.65 +/- 1.27
Score
0
1
2
3
4
5
ChatGPT-4o
DeepSeek
Gemini-2.5-Pro
OpenEvidence
Zhipu
AI Model

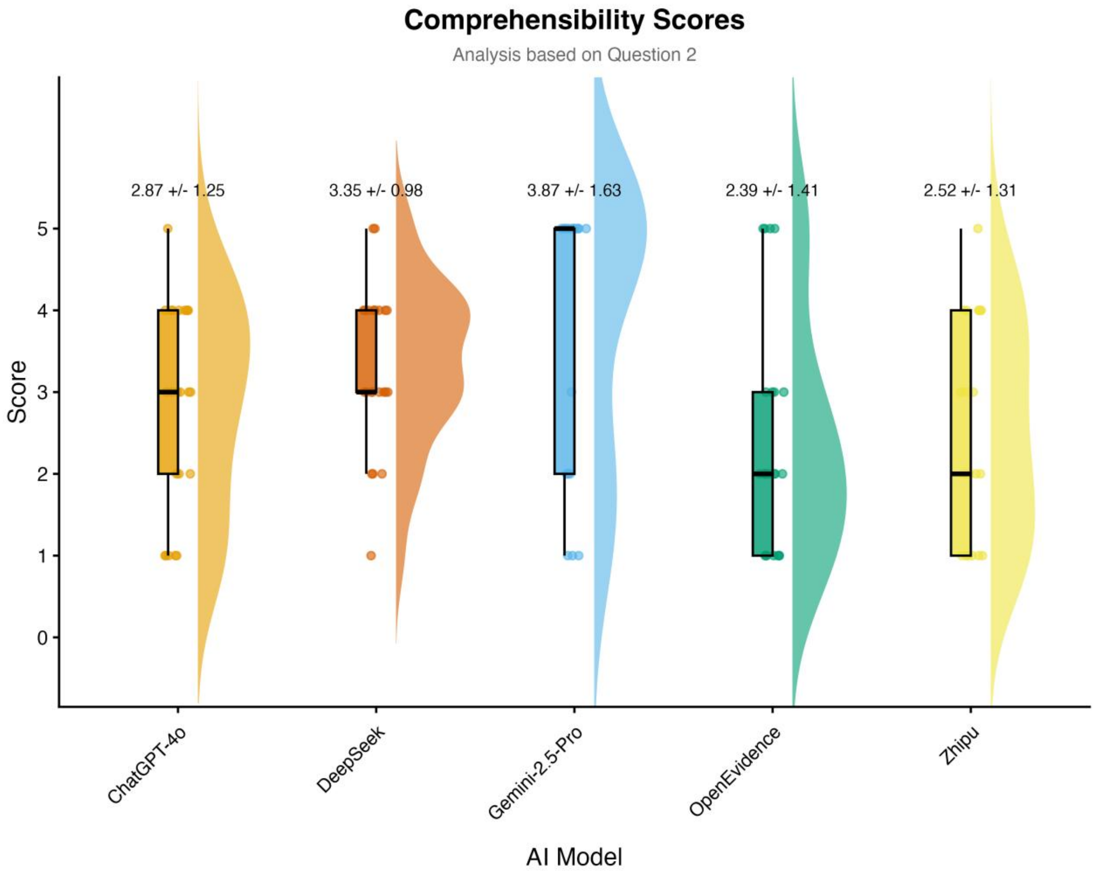
Comprehensibility Scores
Analysis based on Question 2
2.87 +/- 1.25
3.35 +/- 0.98
3.87 +/- 1.63
2.39 +/- 1.41
2.52 +/- 1.31
Score
0
1
2
3
4
5
ChatGPT-4o
DeepSeek
Gemini-2.5-Pro
OpenEvidence
Zhipu
AI Model

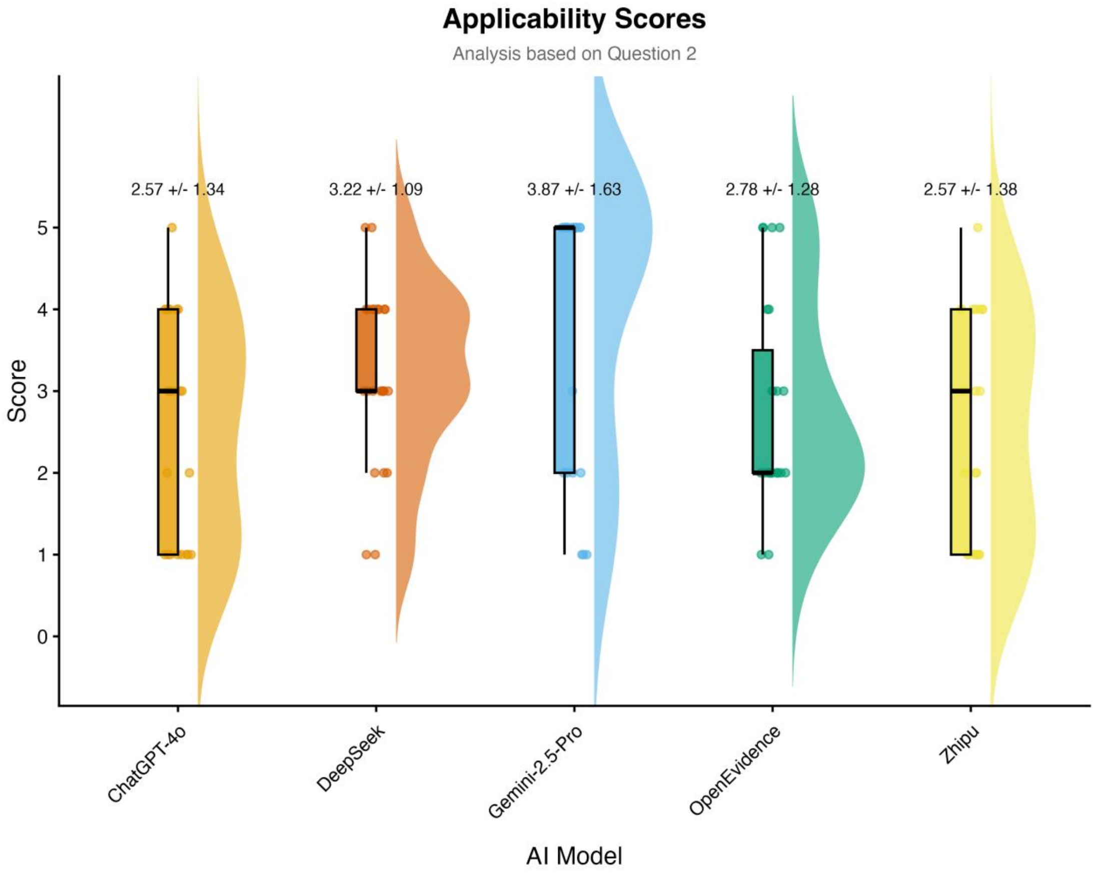
Applicability Scores
Analysis based on Question 2
2.57 +/- 1.34
3.22 +/- 1.09
3.87 +/- 1.63
2.78 +/- 1.28
2.57 +/- 1.38
Score
5
4
3
2
1
0
ChatGPT-4o
DeepSeek
Gemini-2.5-Pro
OpenEvidence
Zhipu
AI Model

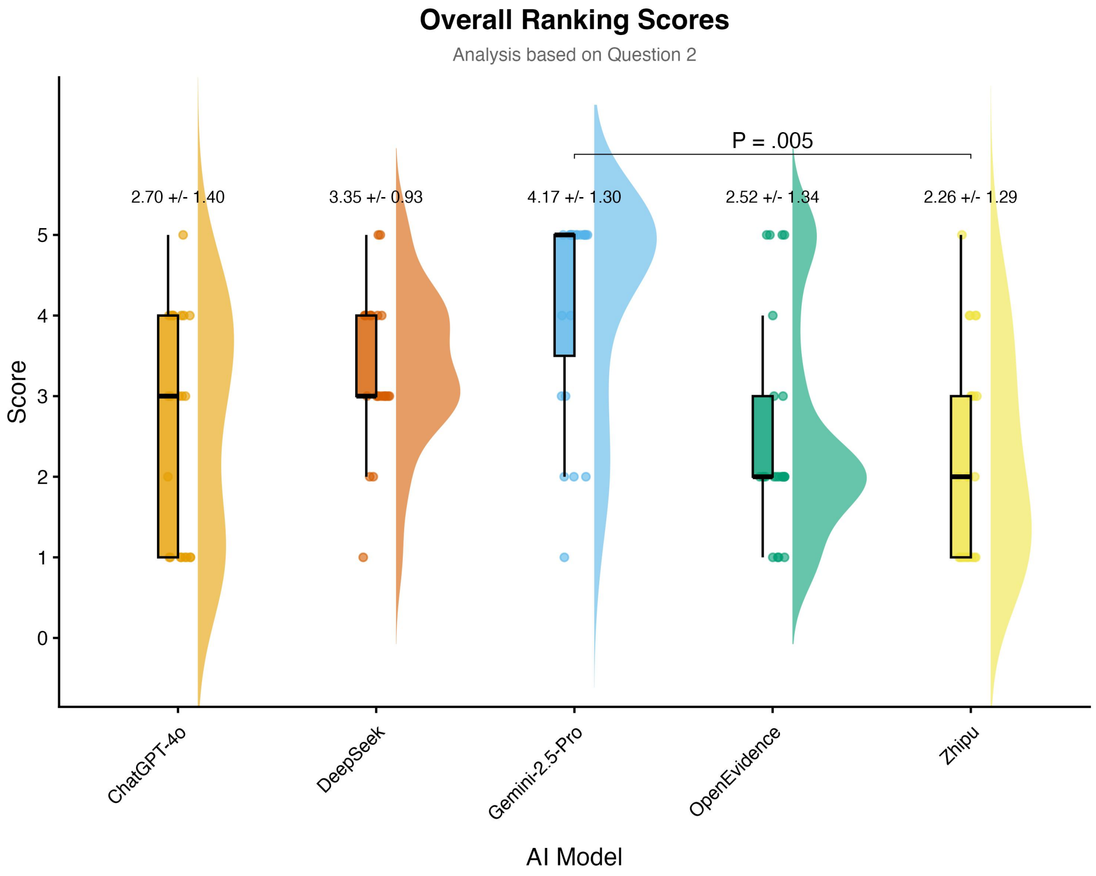
Overall Ranking Scores
Analysis based on Question 2
P = .005
2.70 +/- 1.40
3.35 +/- 0.93
4.17 +/- 1.30
2.52 +/- 1.34
2.26 +/- 1.29
Score
5
4
3
2
1
0
ChatGPT-4o
DeepSeek
Gemini-2.5-Pro
OpenEvidence
Zhipu
AI Model

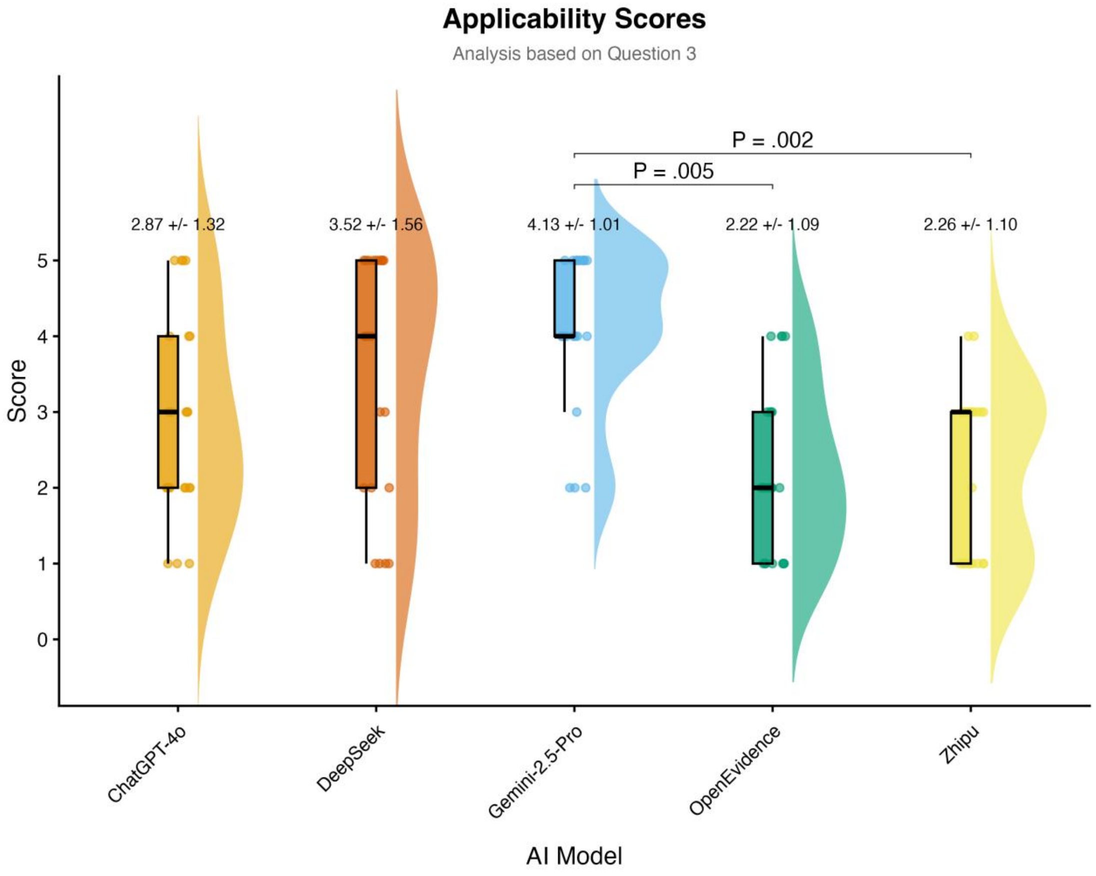
Applicability Scores
Analysis based on Question 3
P = .002
P = .005
2.87 +/- 1.32
3.52 +/- 1.56
4.13 +/- 1.01
2.22 +/- 1.09
2.26 +/- 1.10
Score
5
4
3
2
1
0
ChatGPT-4o
DeepSeek
Gemini-2.5-Pro
OpenEvidence
Zhipu
AI Model

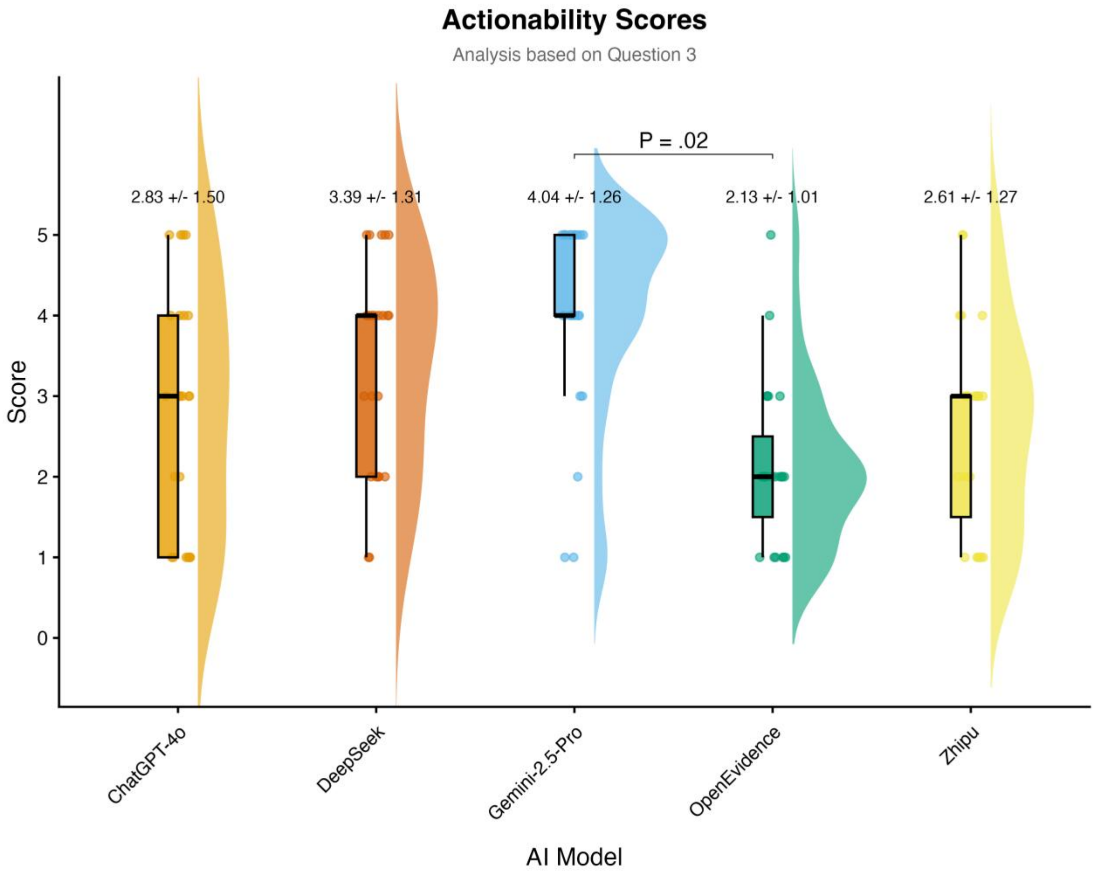
Actionability Scores
Analysis based on Question 3
P = .02
2.83 +/- 1.50
3.39 +/- 1.31
4.04 +/- 1.26
2.13 +/- 1.01
2.61 +/- 1.27
Score
5
4
3
2
1
0
ChatGPT-4o
DeepSeek
Gemini-2.5-Pro
OpenEvidence
Zhipu
AI Model

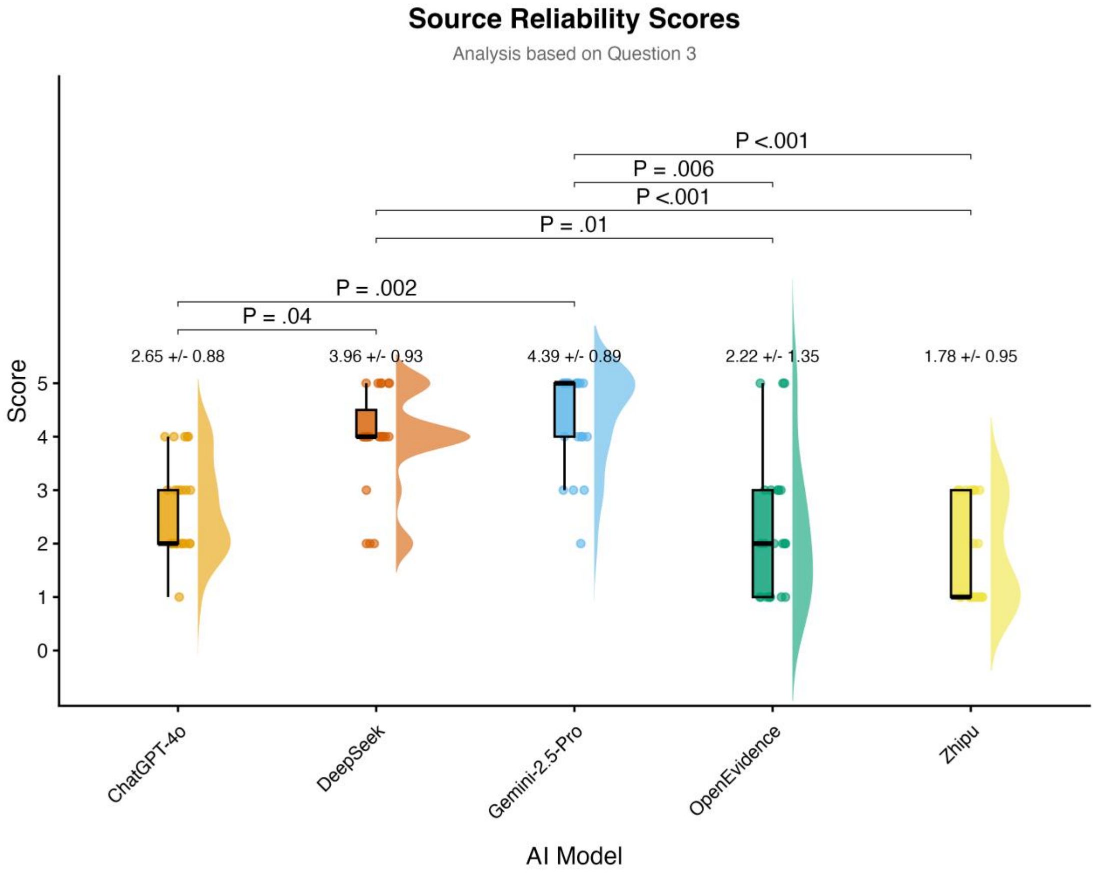
Source Reliability Scores
Analysis based on Question 3
P <.001
P = .006
P <.001
P = .01
P = .002
P = .04
2.65 +/- 0.88
3.96 +/- 0.93
4.39 +/- 0.89
2.22 +/- 1.35
1.78 +/- 0.95
Score
5
4
3
2
1
0
ChatGPT-4o
DeepSeek
Gemini-2.5-Pro
OpenEvidence
Zhipu
AI Model

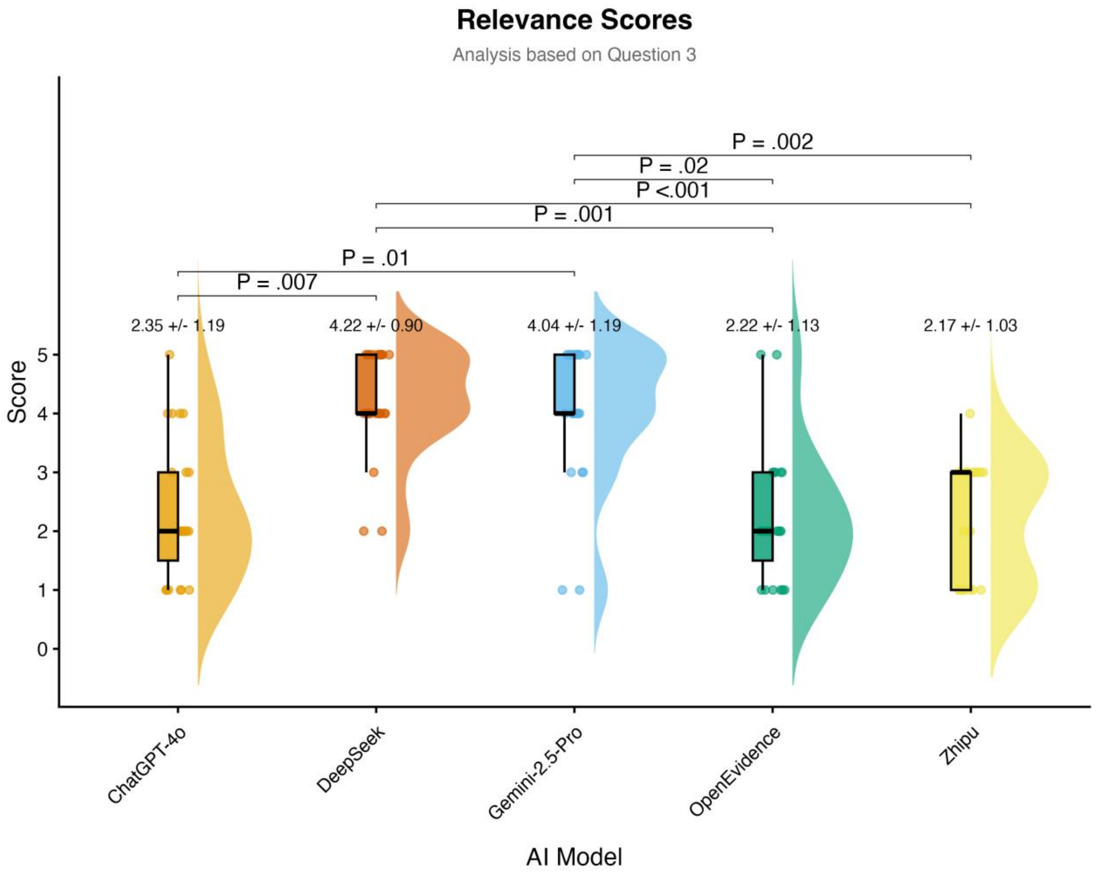
Relevance Scores
Analysis based on Question 3
P = .002
P = .02
P <.001
P = .001
P = .01
P = .007
2.35 +/- 1.19
4.22 +/- 0.90
4.04 +/- 1.19
2.22 +/- 1.13
2.17 +/- 1.03
Score
5
4
3
2
1
0
ChatGPT-4o
DeepSeek
Gemini-2.5-Pro
OpenEvidence
Zhipu
AI Model

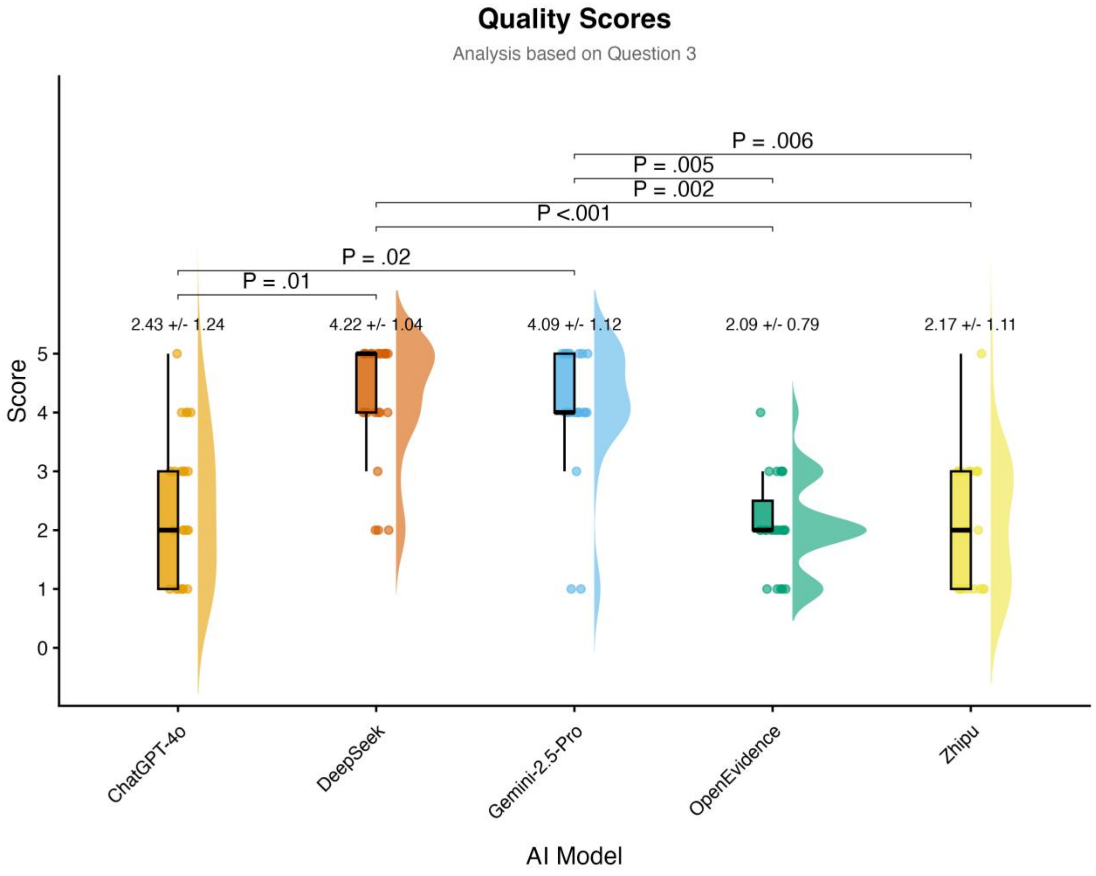
Quality Scores
Analysis based on Question 3
P = .006
P = .005
P = .002
P <.001
P = .02
P = .01
2.43 +/- 1.24
4.22 +/- 1.04
4.09 +/- 1.12
2.09 +/- 0.79
2.17 +/- 1.11
Score
0
1
2
3
4
5
ChatGPT-4o
DeepSeek
Gemini-2.5-Pro
OpenEvidence
Zhipu
AI Model

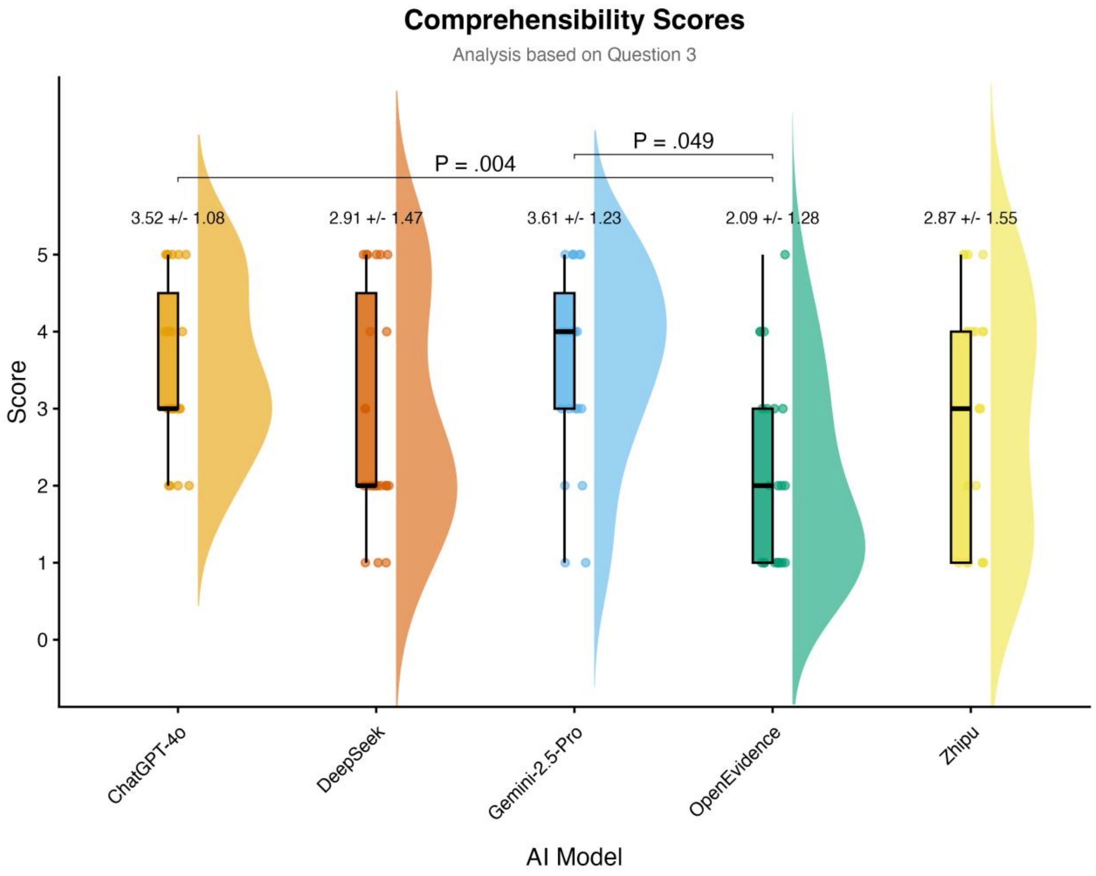
Comprehensibility Scores
Analysis based on Question 3
P = .049
P = .004
3.52 +/- 1.08
2.91 +/- 1.47
3.61 +/- 1.23
2.09 +/- 1.28
2.87 +/- 1.55
Score
0
1
2
3
4
5
ChatGPT-4o
DeepSeek
Gemini-2.5-Pro
OpenEvidence
Zhipu
AI Model

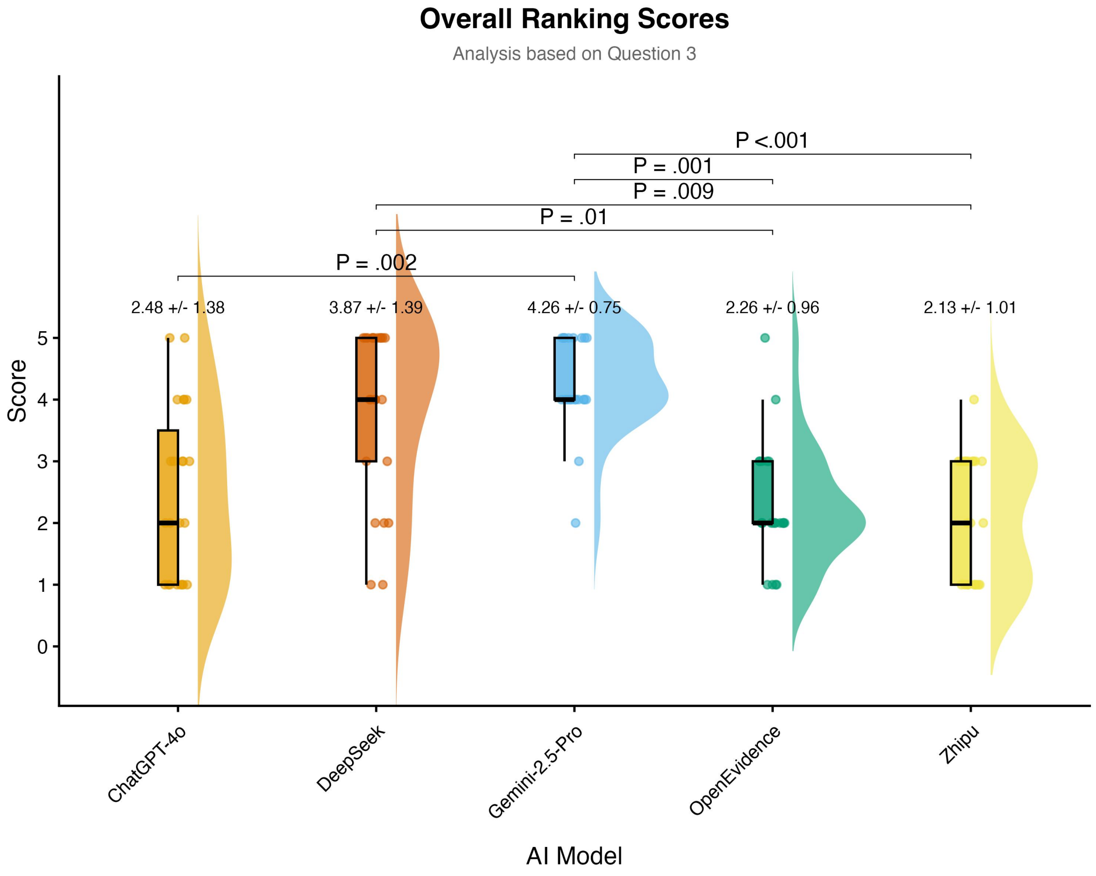
Overall Ranking Scores
Analysis based on Question 3
P <.001
P = .001
P = .009
P = .01
P = .002
2.48 +/- 1.38
3.87 +/- 1.39
4.26 +/- 0.75
2.26 +/- 0.96
2.13 +/- 1.01
Score
0
1
2
3
4
5
ChatGPT-4o
DeepSeek
Gemini-2.5-Pro
OpenEvidence
Zhipu
AI Model

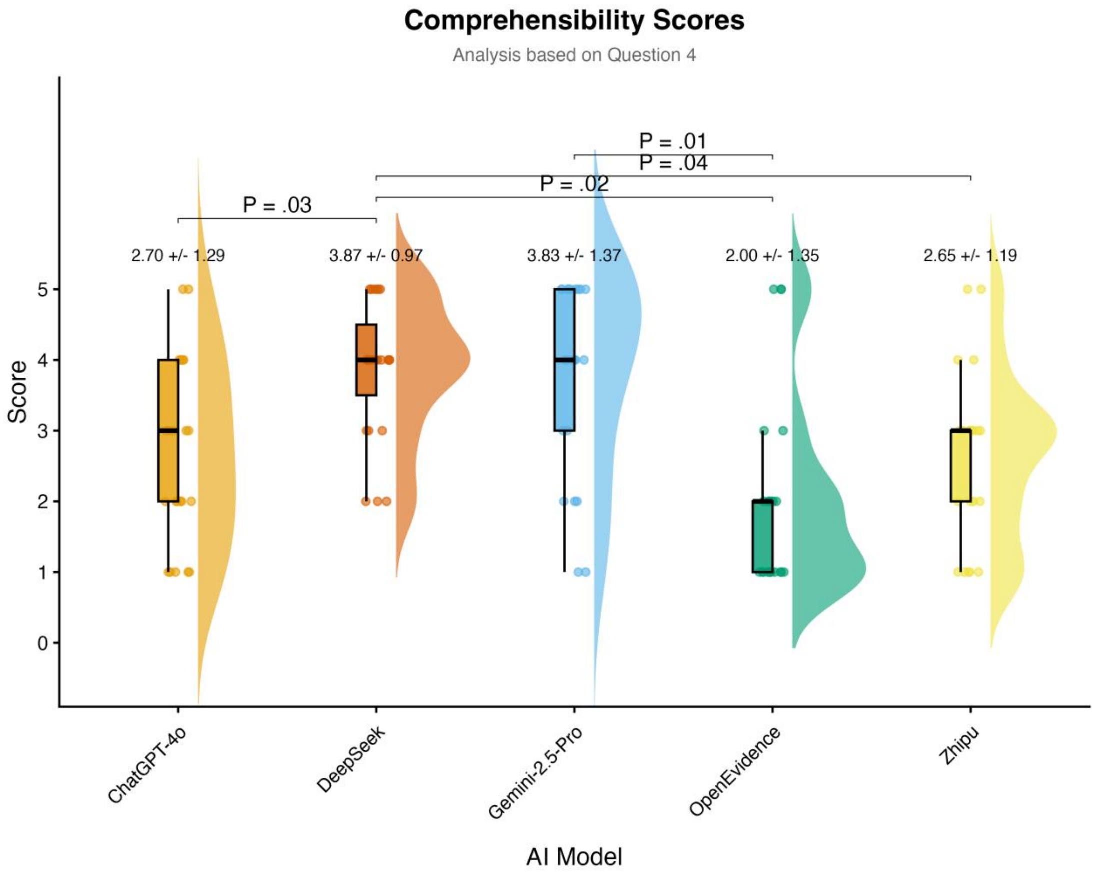
Comprehensibility Scores
Analysis based on Question 4
P = .01
P = .04
P = .02
P = .03
2.70 +/- 1.29
3.87 +/- 0.97
3.83 +/- 1.37
2.00 +/- 1.35
2.65 +/- 1.19
Score
0
1
2
3
4
5
ChatGPT-4o
DeepSeek
Gemini-2.5-Pro
OpenEvidence
Zhipu
AI Model

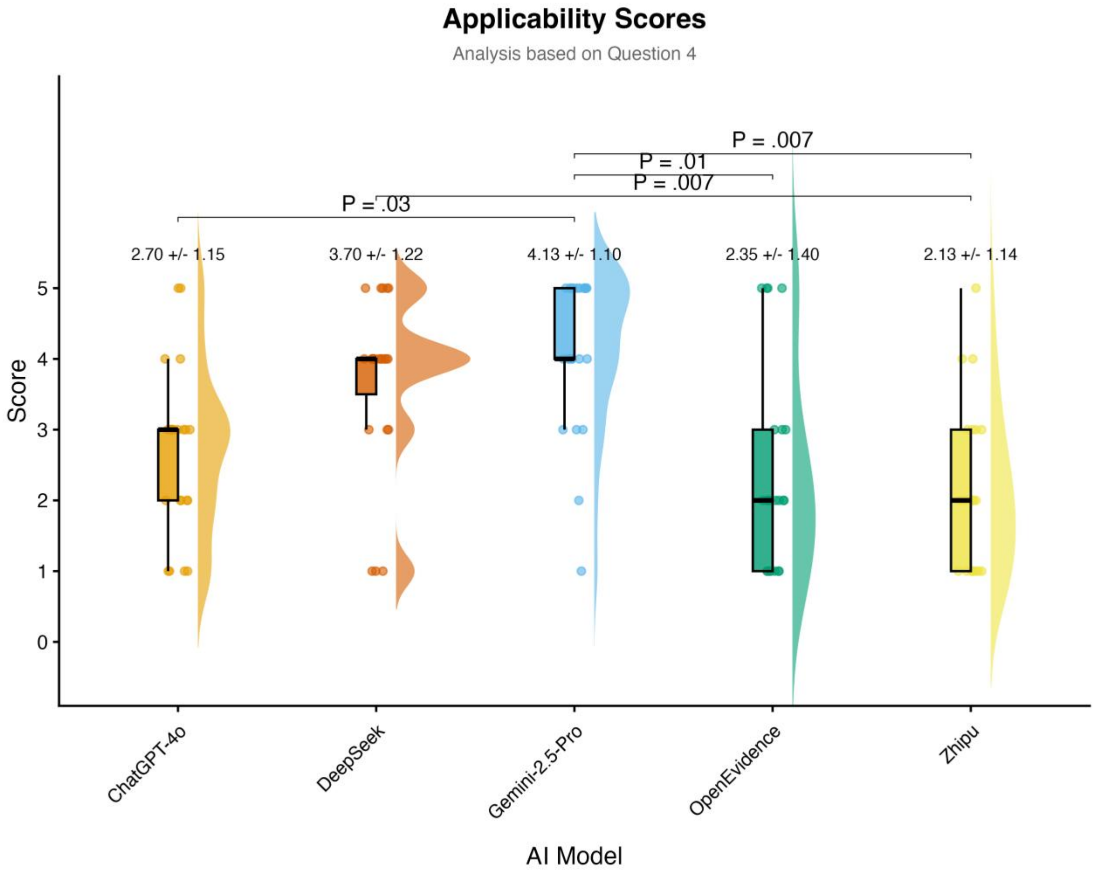
Applicability Scores
Analysis based on Question 4
P = .007
P = .01
P = .007
P = .03
2.70 +/- 1.15
3.70 +/- 1.22
4.13 +/- 1.10
2.35 +/- 1.40
2.13 +/- 1.14
Score
5
4
3
2
1
0
ChatGPT-4o
DeepSeek
Gemini-2.5-Pro
OpenEvidence
Zhipu
AI Model

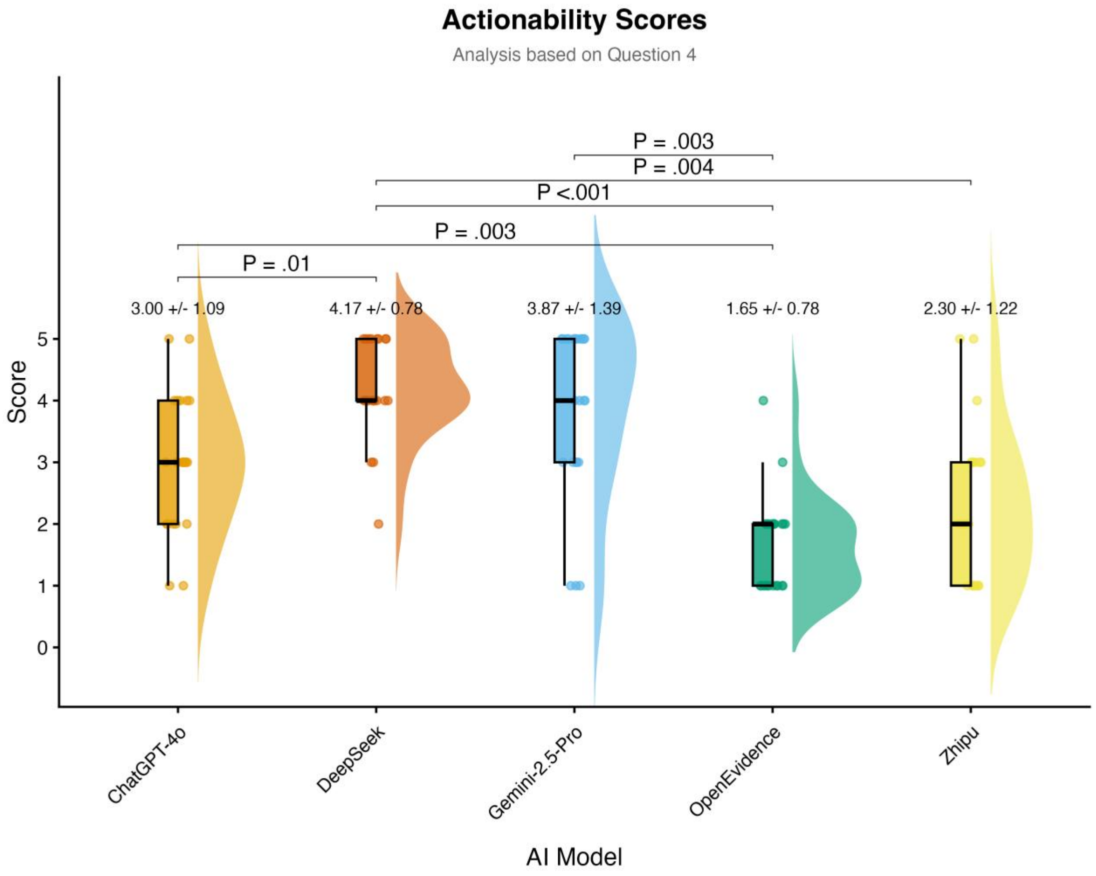
Actionability Scores
Analysis based on Question 4
P = .003
P = .004
P <.001
P = .003
P = .01
3.00 +/- 1.09
4.17 +/- 0.78
3.87 +/- 1.39
1.65 +/- 0.78
2.30 +/- 1.22
Score
5
4
3
2
1
0
ChatGPT-4o
DeepSeek
Gemini-2.5-Pro
OpenEvidence
Zhipu
AI Model

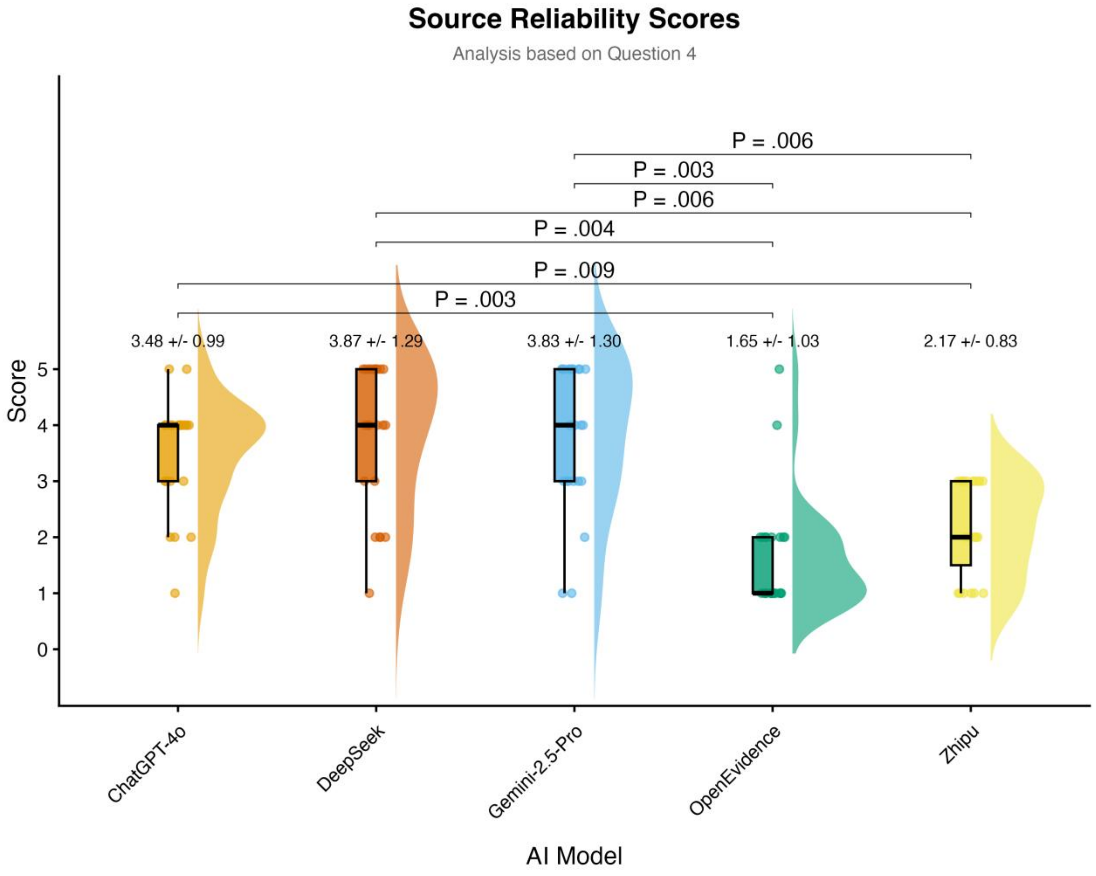
Source Reliability Scores
Analysis based on Question 4
P = .006
P = .003
P = .006
P = .004
P = .009
P = .003
3.48 +/- 0.99
3.87 +/- 1.29
3.83 +/- 1.30
1.65 +/- 1.03
2.17 +/- 0.83
Score
0
1
2
3
4
5
ChatGPT-4o
DeepSeek
Gemini-2.5-Pro
OpenEvidence
Zhipu
AI Model

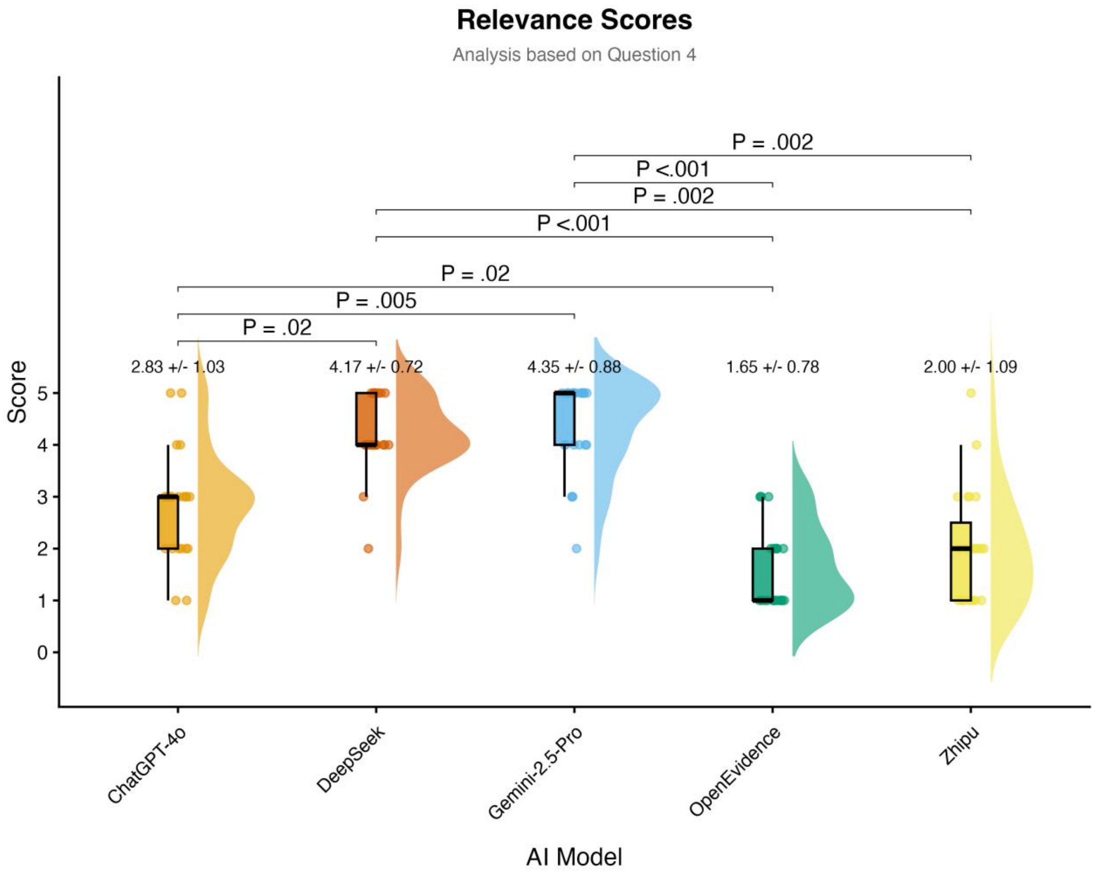
Relevance Scores
Analysis based on Question 4
P = .002
P <.001
P = .002
P <.001
P = .02
P = .005
P = .02
2.83 +/- 1.03
4.17 +/- 0.72
4.35 +/- 0.88
1.65 +/- 0.78
2.00 +/- 1.09
Score
0
1
2
3
4
5
ChatGPT-4o
DeepSeek
Gemini-2.5-Pro
OpenEvidence
Zhipu
AI Model

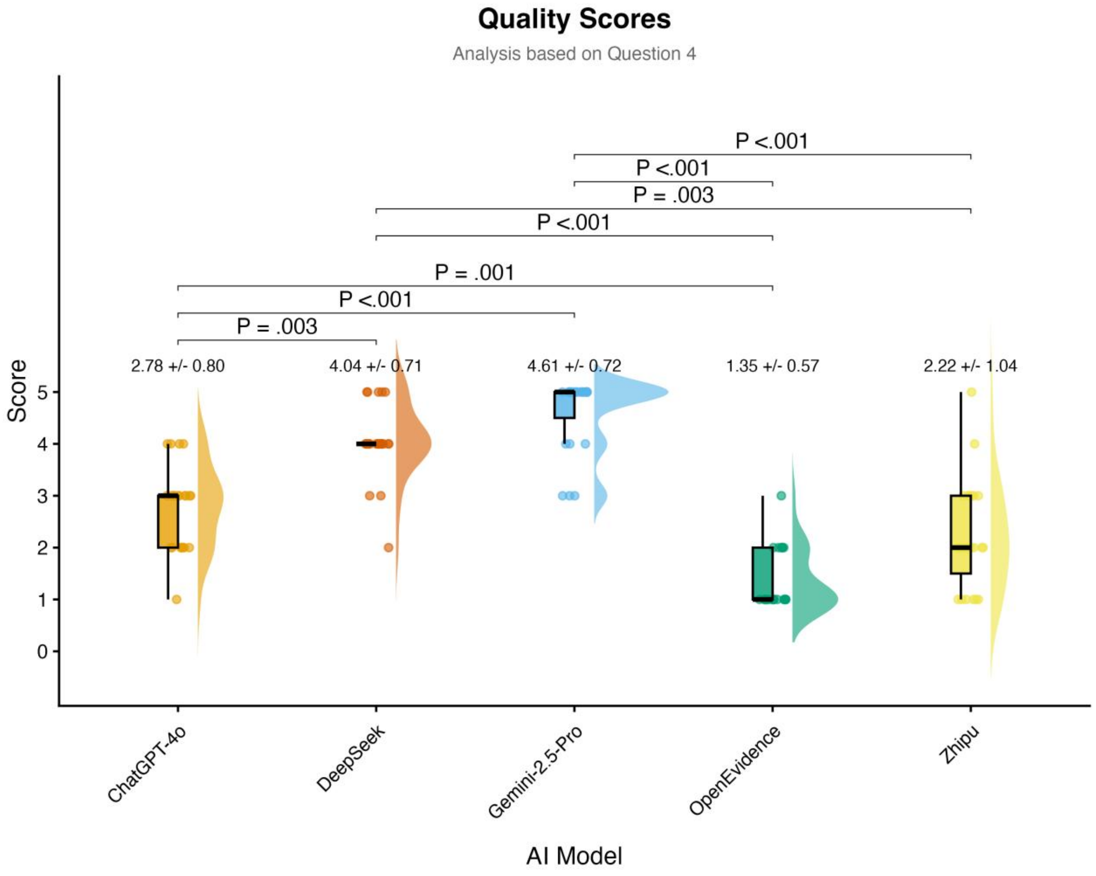

Quality Scores
Analysis based on Question 4
P <.001
P <.001
P = .003
P <.001
P = .001
P <.001
P = .003
2.78 +/- 0.80
4.04 +/- 0.71
4.61 +/- 0.72
1.35 +/- 0.57
2.22 +/- 1.04
Score
5
4
3
2
1
0
ChatGPT-4o
DeepSeek
Gemini-2.5-Pro
OpenEvidence
Zhipu
AI Model

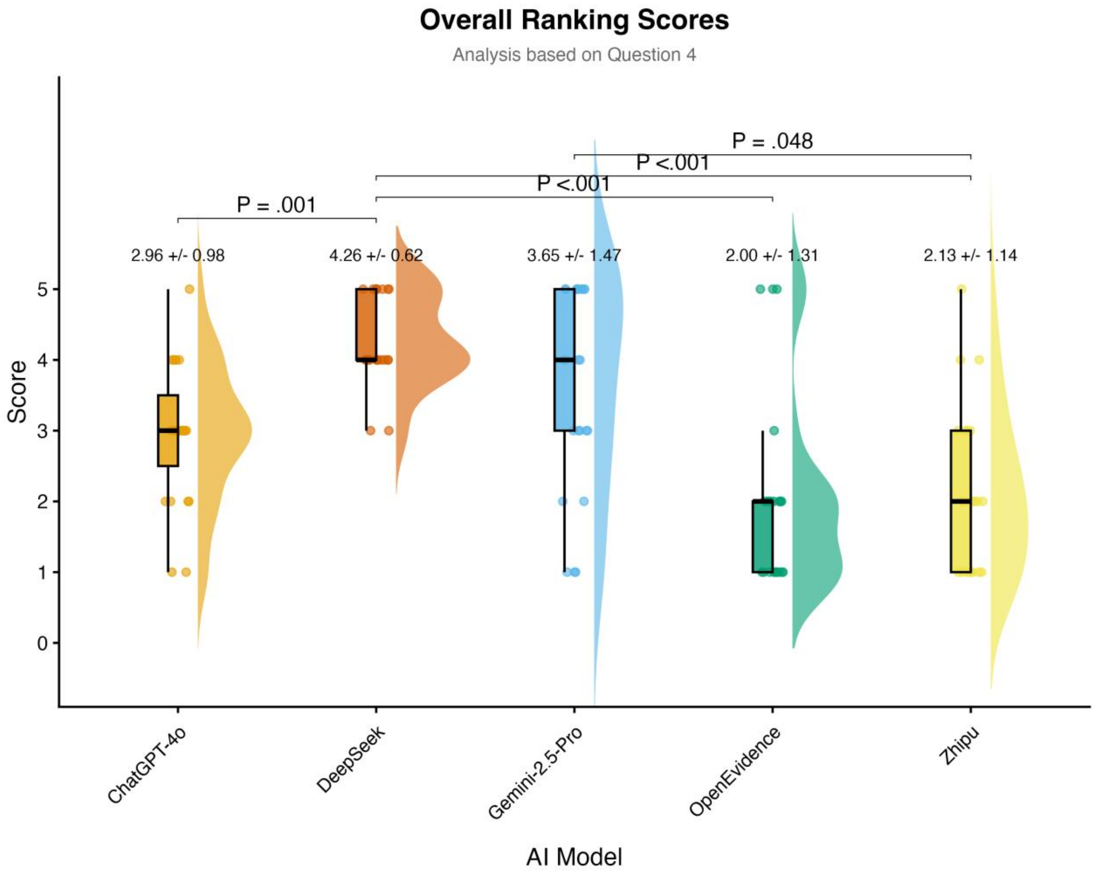

Overall Ranking Scores
Analysis based on Question 4
P = .048
P <.001
P <.001
P = .001
2.96 +/- 0.98
4.26 +/- 0.62
3.65 +/- 1.47
2.00 +/- 1.31
2.13 +/- 1.14
Score
5
4
3
2
1
0
ChatGPT-4o
DeepSeek
Gemini-2.5-Pro
OpenEvidence
Zhipu
AI Model

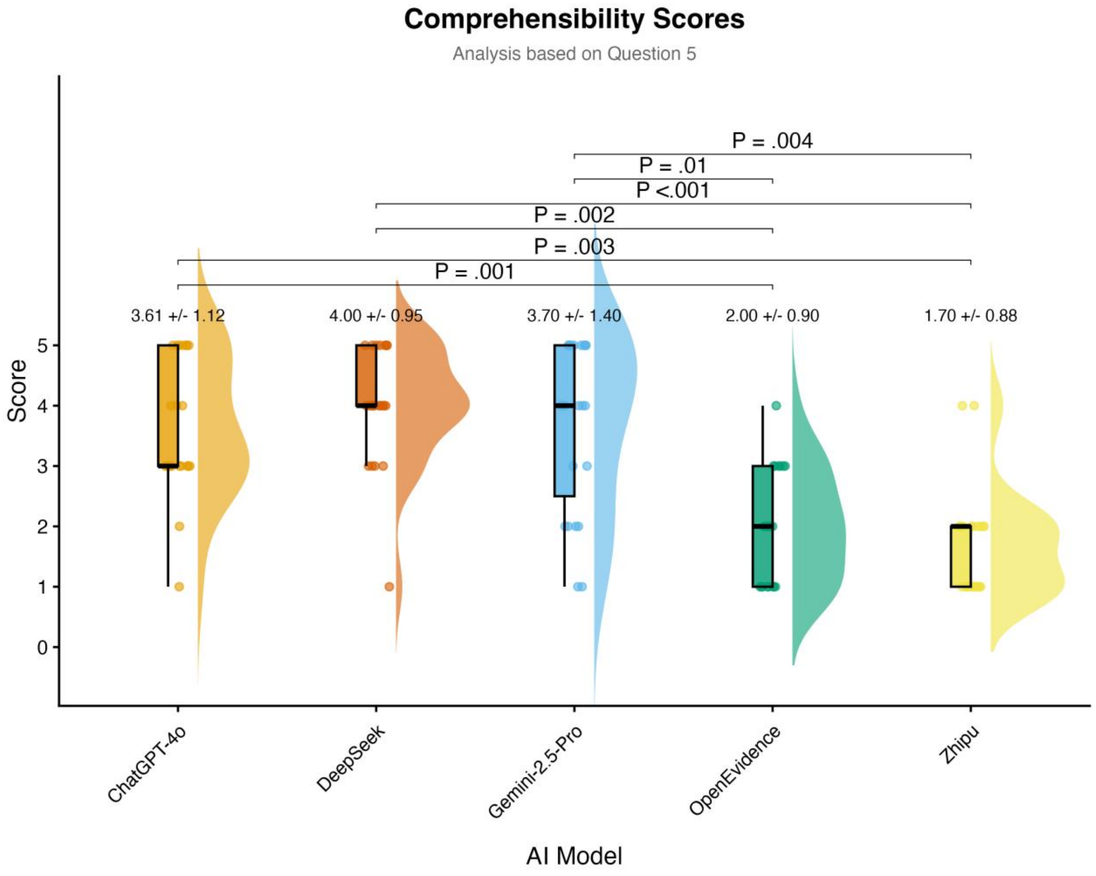
Comprehensibility Scores
Analysis based on Question 5
P = .004
P = .01
P <.001
P = .002
P = .003
P = .001
3.61 +/- 1.12
4.00 +/- 0.95
3.70 +/- 1.40
2.00 +/- 0.90
1.70 +/- 0.88
Score
AI Model
ChatGPT-4o
DeepSeek
Gemini-2.5-Pro
OpenEvidence
Zhipu

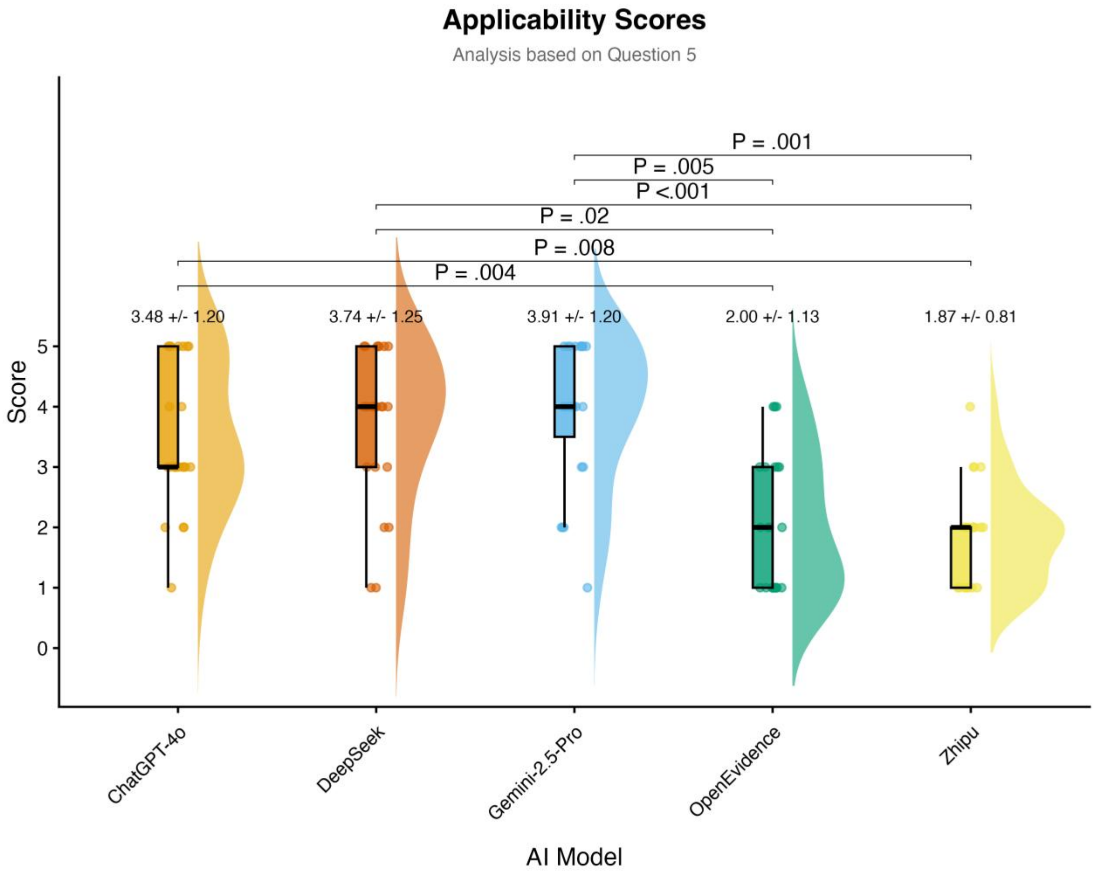
Applicability Scores
Analysis based on Question 5
P = .001
P = .005
P <.001
P = .02
P = .008
P = .004
3.48 +/- 1.20
3.74 +/- 1.25
3.91 +/- 1.20
2.00 +/- 1.13
1.87 +/- 0.81
Score
AI Model
ChatGPT-4o
DeepSeek
Gemini-2.5-Pro
OpenEvidence
Zhipu

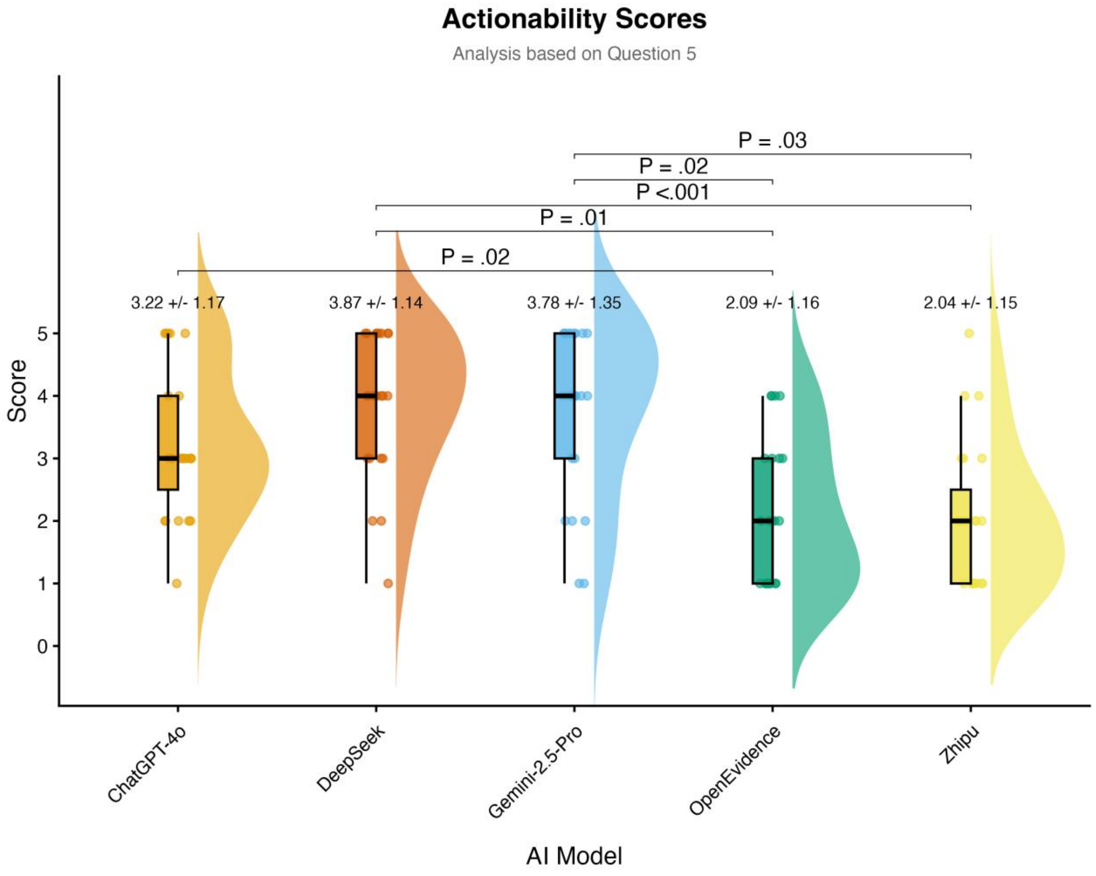
Actionability Scores
Analysis based on Question 5
P = .03
P = .02
P <.001
P = .01
P = .02
3.22 +/- 1.17
3.87 +/- 1.14
3.78 +/- 1.35
2.09 +/- 1.16
2.04 +/- 1.15
Score
0
1
2
3
4
5
ChatGPT-4o
DeepSeek
Gemini-2.5-Pro
OpenEvidence
Zhipu
AI Model

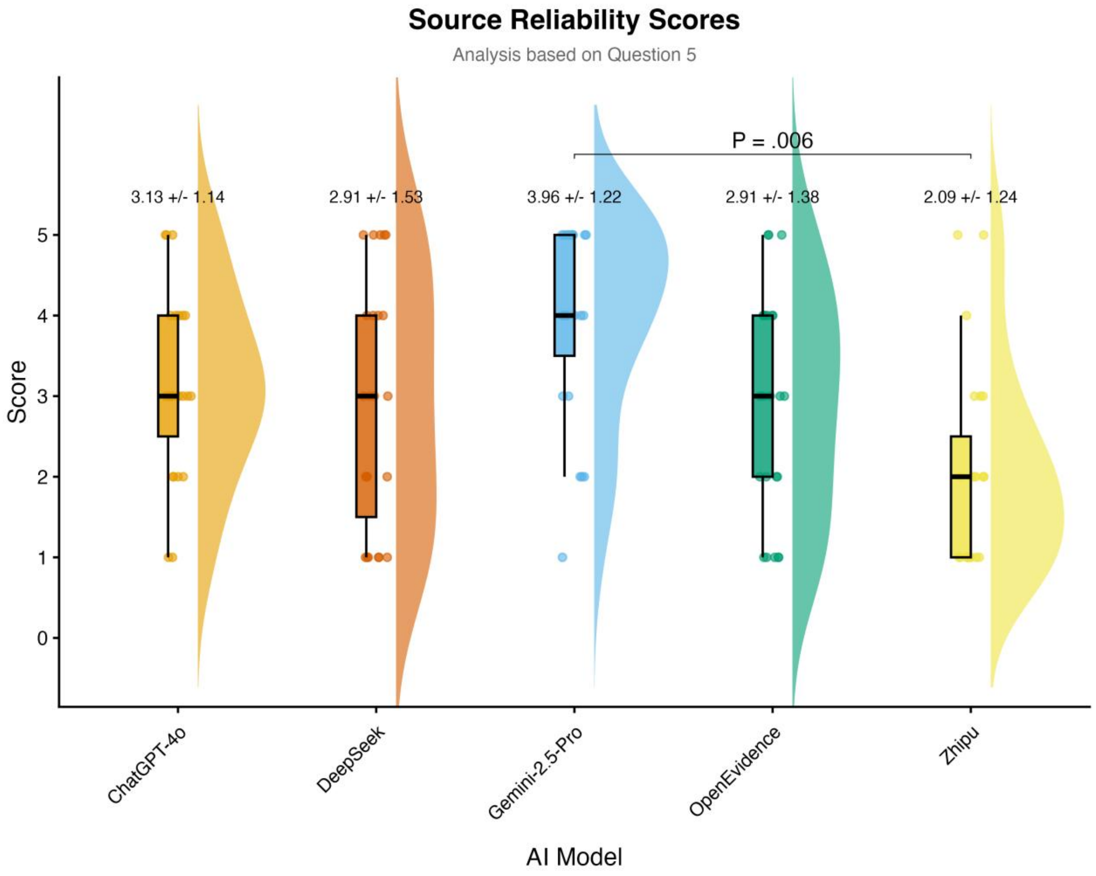
Source Reliability Scores
Analysis based on Question 5
P = .006
3.13 +/- 1.14
2.91 +/- 1.53
3.96 +/- 1.22
2.91 +/- 1.38
2.09 +/- 1.24
Score
0
1
2
3
4
5
ChatGPT-4o
DeepSeek
Gemini-2.5-Pro
OpenEvidence
Zhipu
AI Model

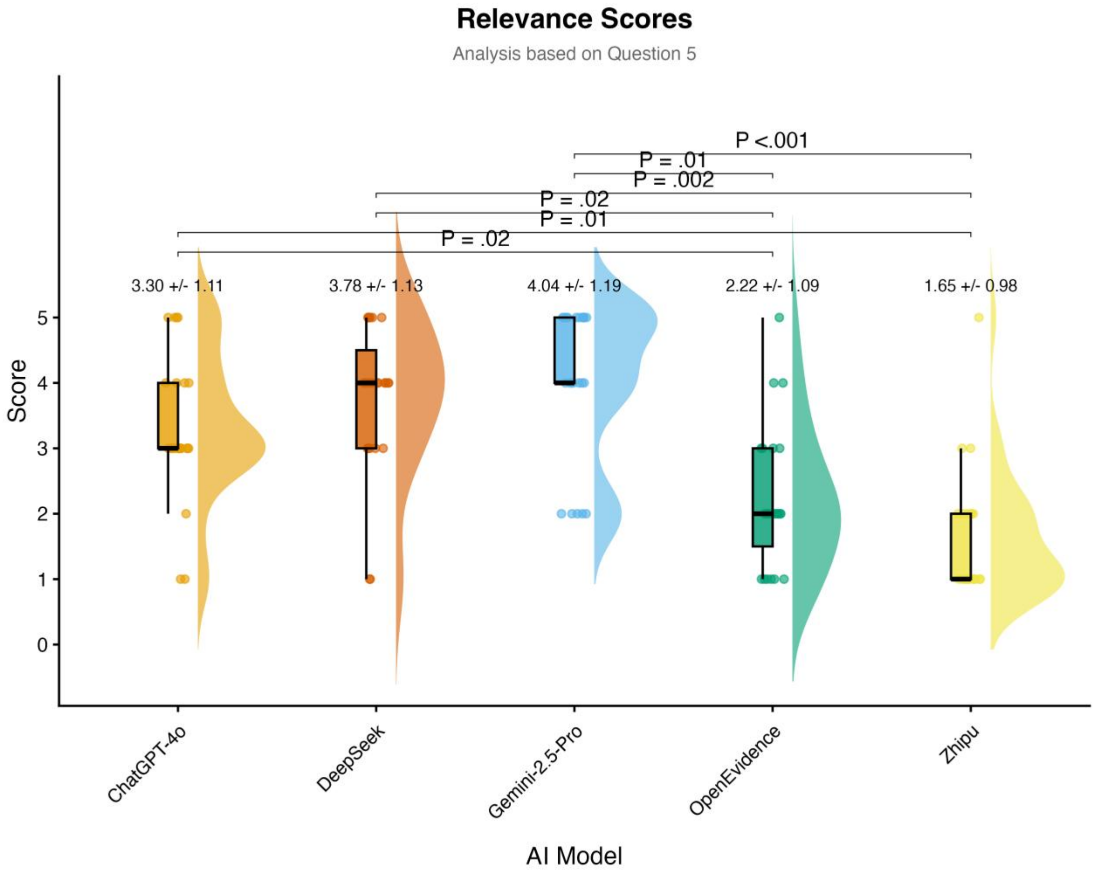

Relevance Scores
Analysis based on Question 5
P <.001
P = .01
P = .002
P = .02
P = .01
P = .02
3.30 +/- 1.11
3.78 +/- 1.13
4.04 +/- 1.19
2.22 +/- 1.09
1.65 +/- 0.98
Score
0
1
2
3
4
5
ChatGPT-4o
DeepSeek
Gemini-2.5-Pro
OpenEvidence
Zhipu
AI Model

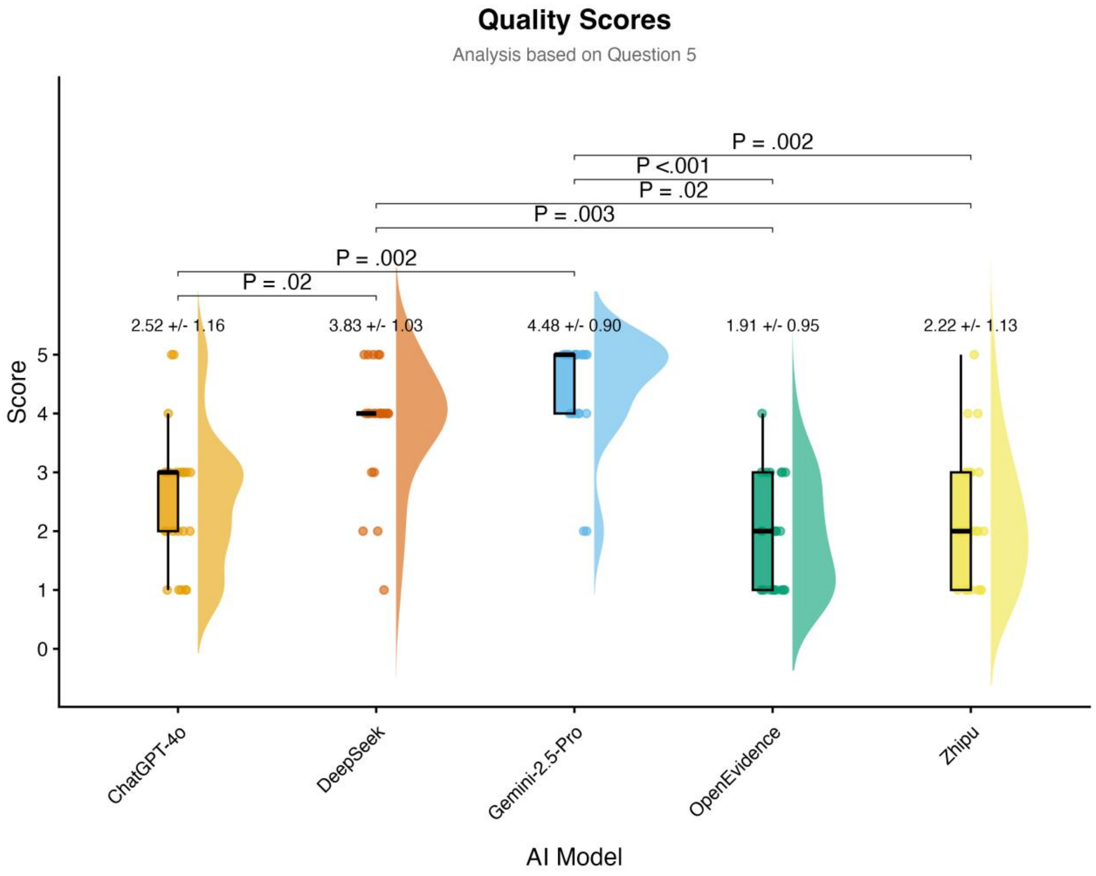

Quality Scores
Analysis based on Question 5
P = .002
P <.001
P = .02
P = .003
P = .002
P = .02
2.52 +/- 1.16
3.83 +/- 1.03
4.48 +/- 0.90
1.91 +/- 0.95
2.22 +/- 1.13
Score
0
1
2
3
4
5
ChatGPT-4o
DeepSeek
Gemini-2.5-Pro
OpenEvidence
Zhipu
AI Model

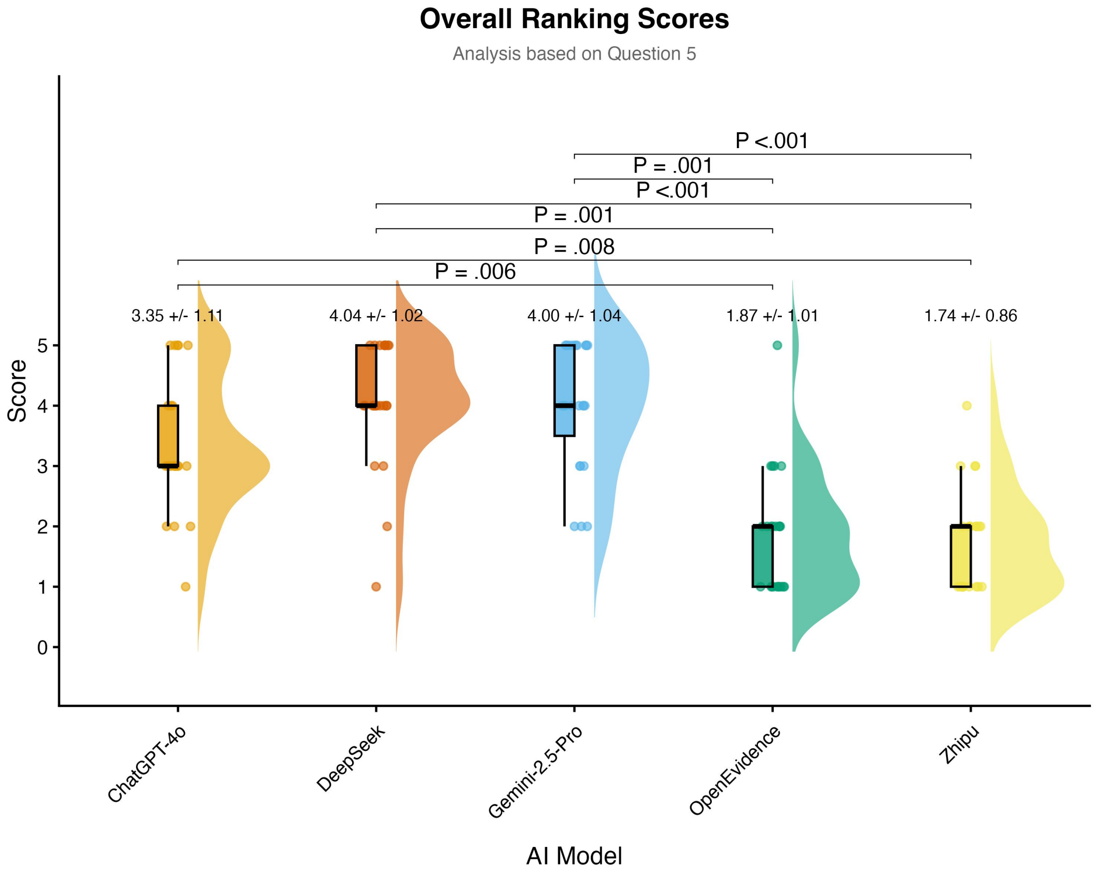

Overall Ranking Scores
Analysis based on Question 5
P <.001
P = .001
P <.001
P = .001
P = .008
P = .006
3.35 +/- 1.11
4.04 +/- 1.02
4.00 +/- 1.04
1.87 +/- 1.01
1.74 +/- 0.86
Score
AI Model
ChatGPT-4o
DeepSeek
Gemini-2.5-Pro
OpenEvidence
Zhipu

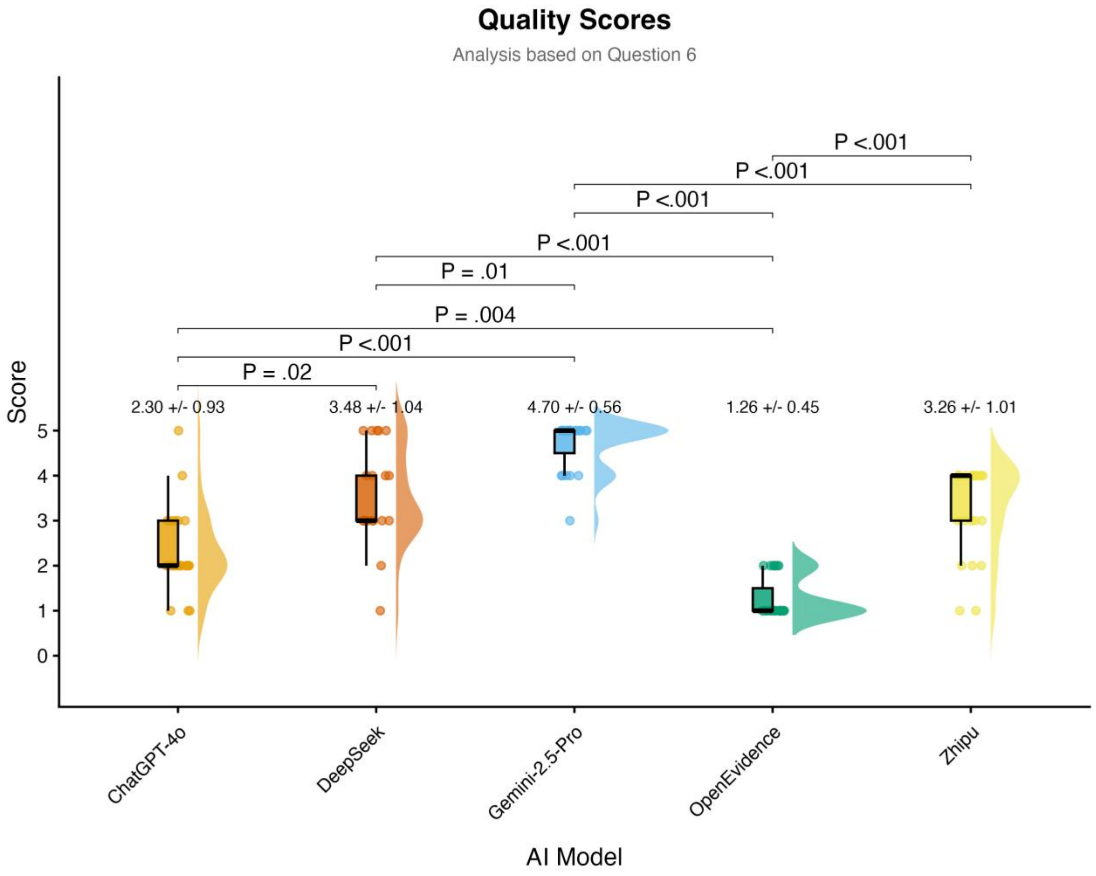

Quality Scores
Analysis based on Question 6
P <.001
P <.001
P <.001
P <.001
P = .01
P = .004
P <.001
P = .02
2.30 +/- 0.93
3.48 +/- 1.04
4.70 +/- 0.56
1.26 +/- 0.45
3.26 +/- 1.01
Score
AI Model
ChatGPT-4o
DeepSeek
Gemini-2.5-Pro
OpenEvidence
Zhipu

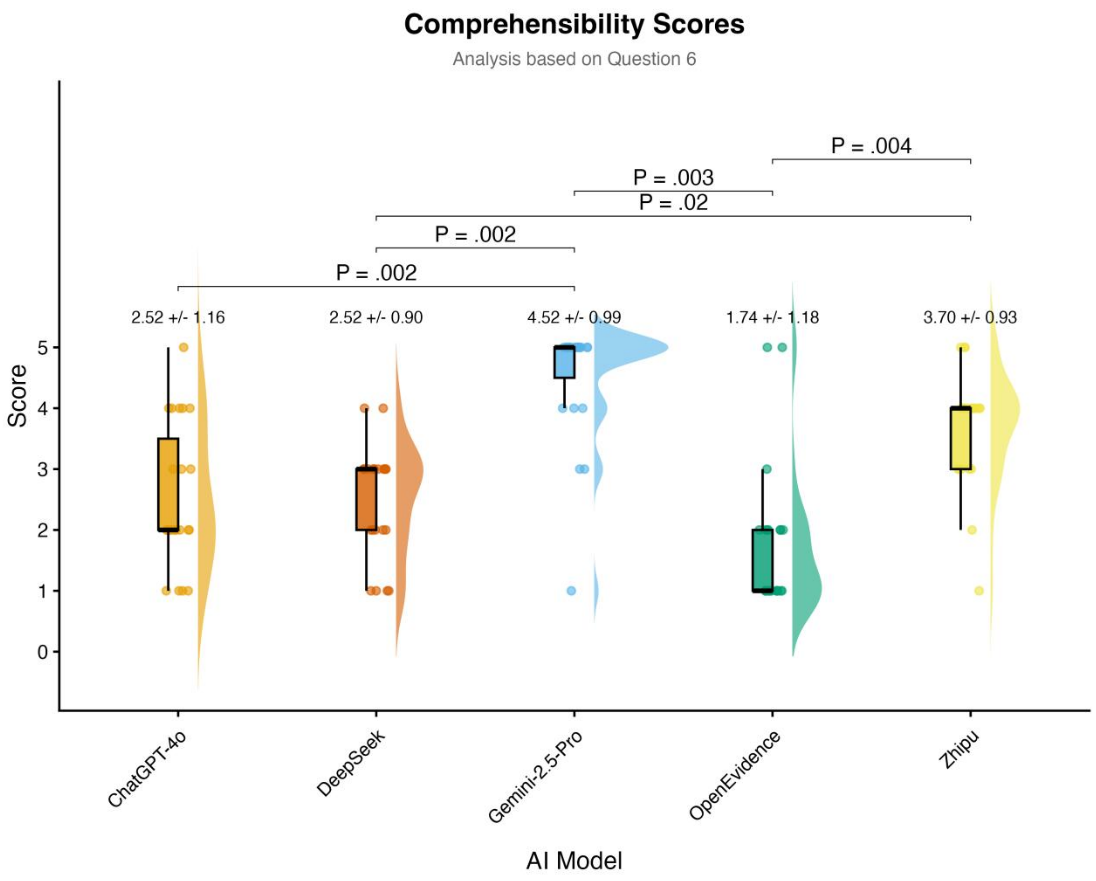
Comprehensibility Scores
Analysis based on Question 6
P = .004
P = .003
P = .02
P = .002
P = .002
2.52 +/- 1.16
2.52 +/- 0.90
4.52 +/- 0.99
1.74 +/- 1.18
3.70 +/- 0.93
Score
0
1
2
3
4
5
ChatGPT-4o
DeepSeek
Gemini-2.5-Pro
OpenEvidence
Zhipu
AI Model

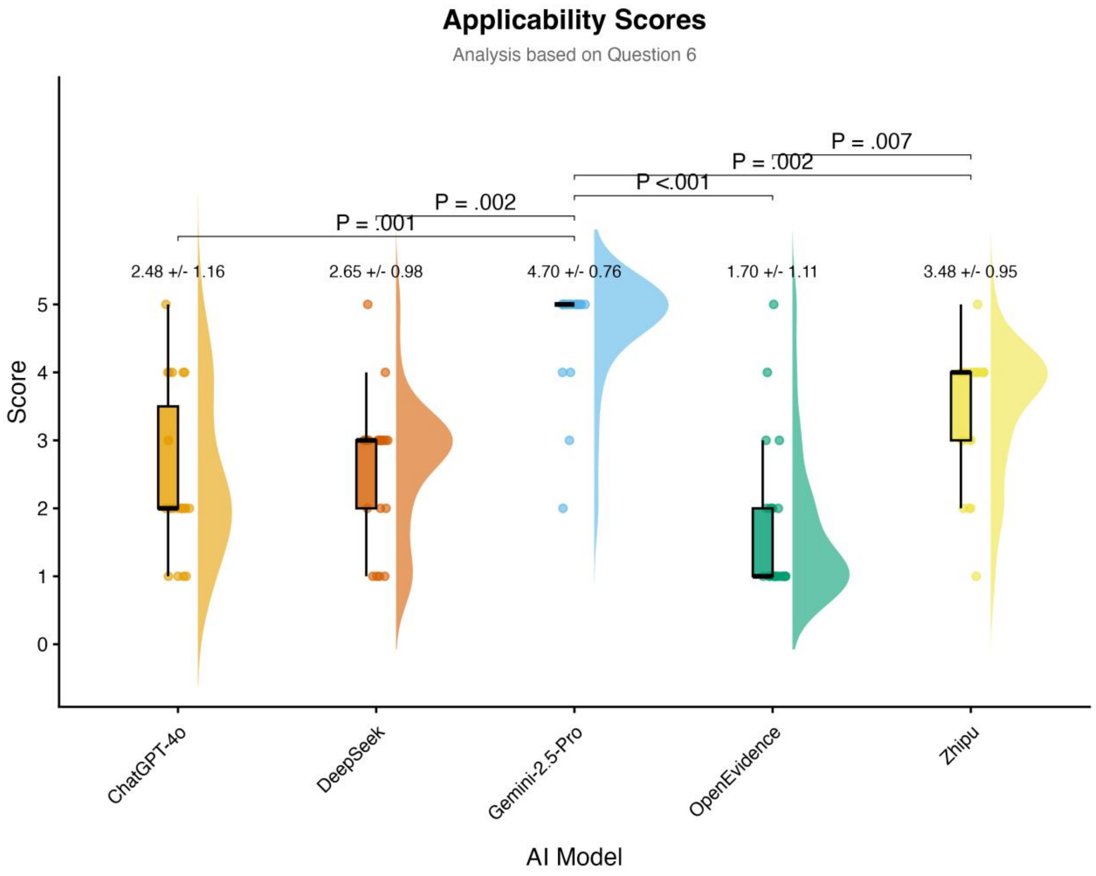
Applicability Scores
Analysis based on Question 6
P = .007
P = .002
P <.001
P = .002
P = .001
2.48 +/- 1.16
2.65 +/- 0.98
4.70 +/- 0.76
1.70 +/- 1.11
3.48 +/- 0.95
Score
0
1
2
3
4
5
ChatGPT-4o
DeepSeek
Gemini-2.5-Pro
OpenEvidence
Zhipu
AI Model

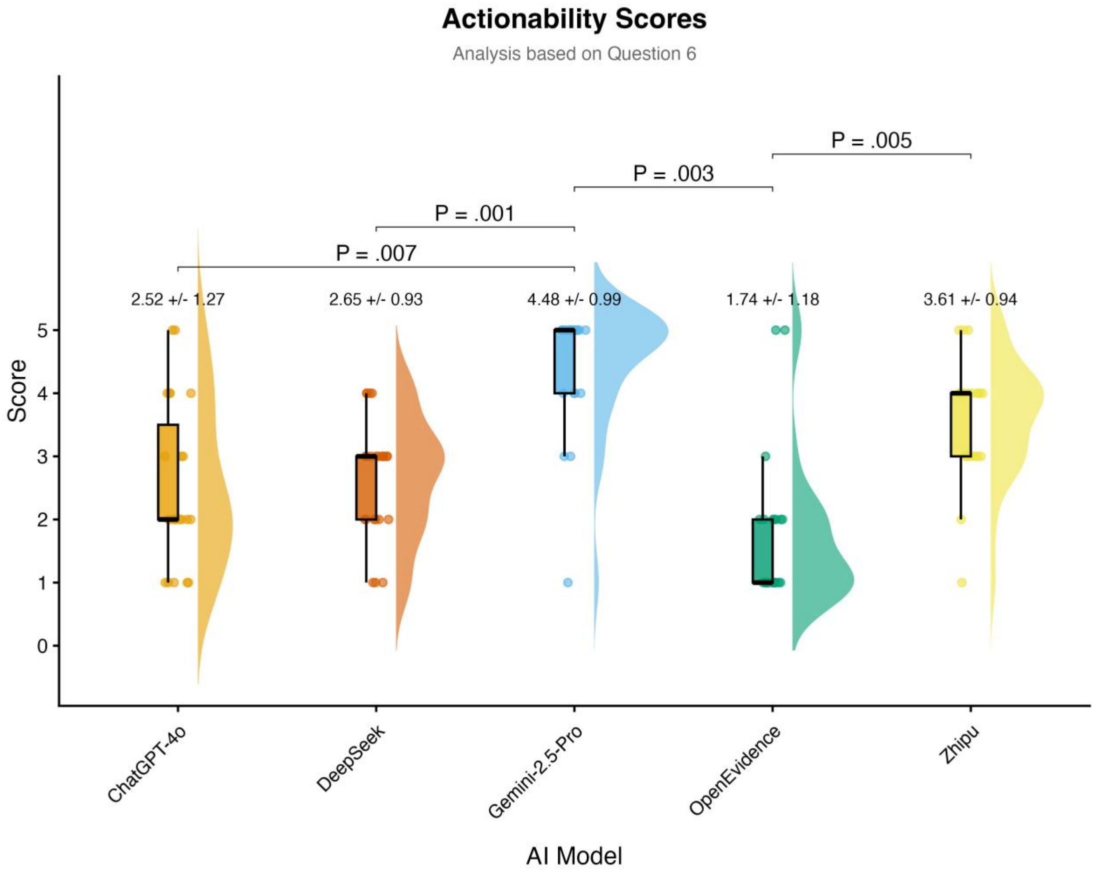

Actionability Scores
Analysis based on Question 6
P = .005
P = .003
P = .001
P = .007
2.52 +/- 1.27
2.65 +/- 0.93
4.48 +/- 0.99
1.74 +/- 1.18
3.61 +/- 0.94
Score
AI Model
ChatGPT-4o
DeepSeek
Gemini-2.5-Pro
OpenEvidence
Zhipu

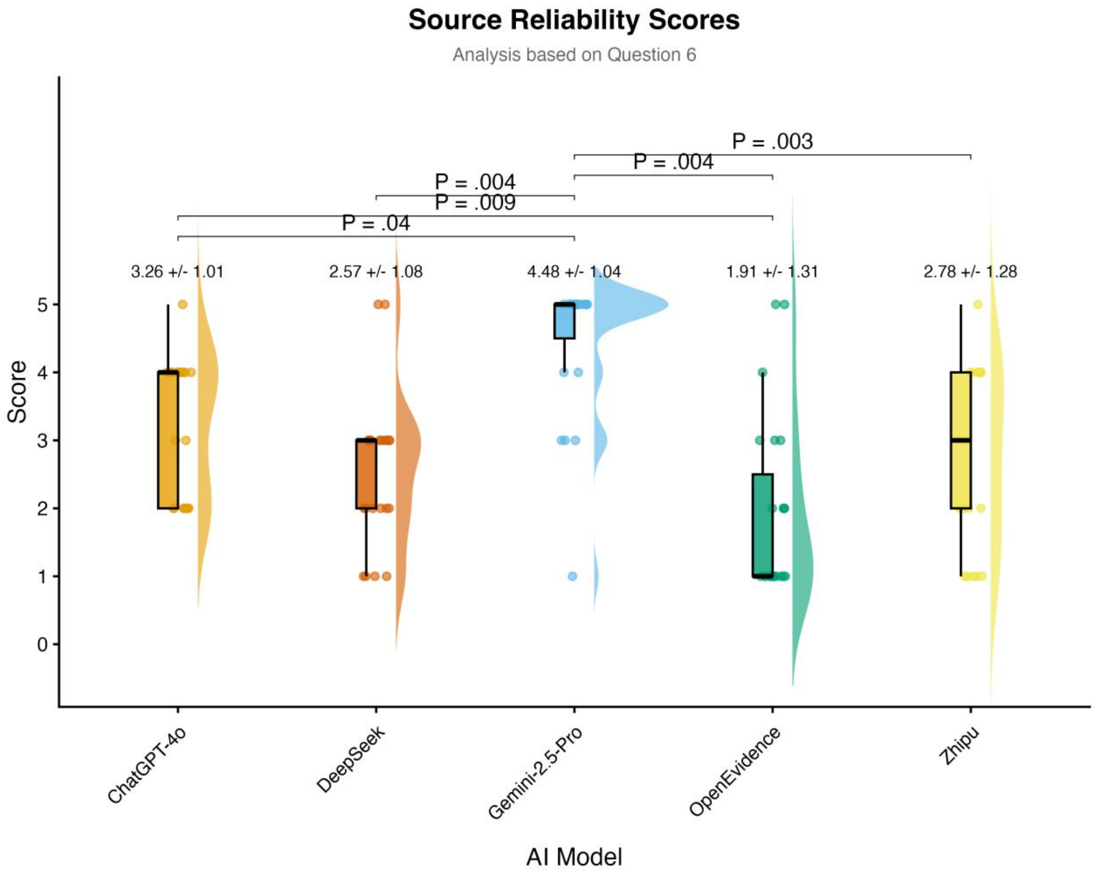

Source Reliability Scores
Analysis based on Question 6
P = .003
P = .004
P = .004
P = .009
P = .04
3.26 +/- 1.01
2.57 +/- 1.08
4.48 +/- 1.04
1.91 +/- 1.31
2.78 +/- 1.28
Score
AI Model
ChatGPT-4o
DeepSeek
Gemini-2.5-Pro
OpenEvidence
Zhipu

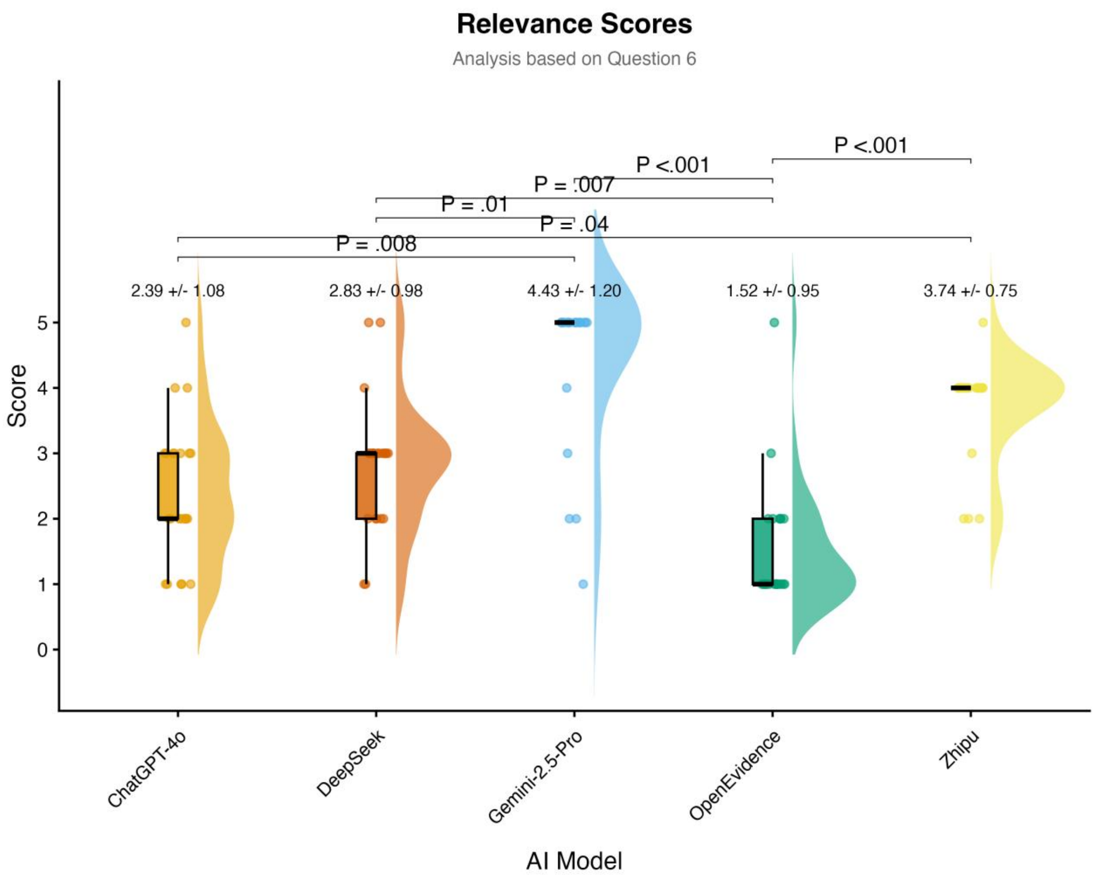

Relevance Scores
Analysis based on Question 6
P <.001
P <.001
P = .007
P = .01
P = .04
P = .008
2.39 +/- 1.08
2.83 +/- 0.98
4.43 +/- 1.20
1.52 +/- 0.95
3.74 +/- 0.75
Score
5
4
3
2
1
0
ChatGPT-4o
DeepSeek
Gemini-2.5-Pro
OpenEvidence
Zhipu
AI Model

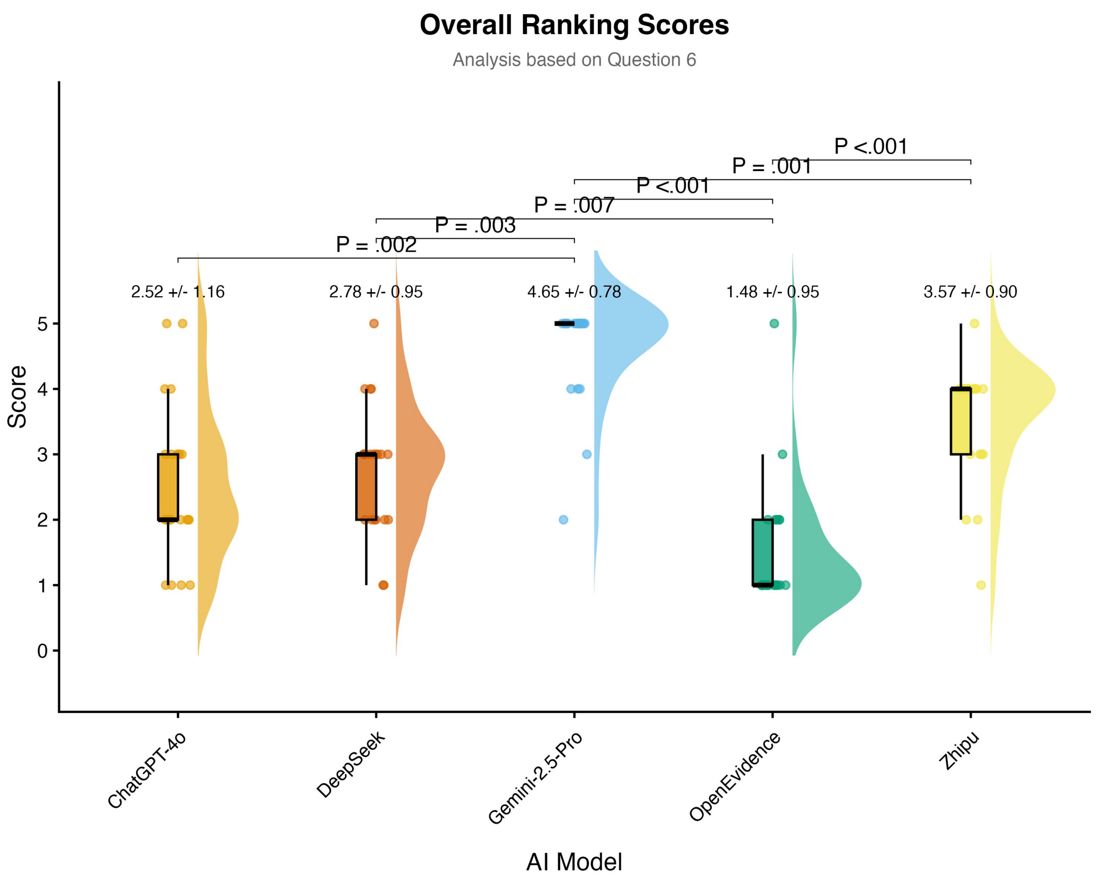

Overall Ranking Scores
Analysis based on Question 6
P <.001
P = .001
P <.001
P = .007
P = .003
P = .002
2.52 +/- 1.16
2.78 +/- 0.95
4.65 +/- 0.78
1.48 +/- 0.95
3.57 +/- 0.90
Score
5
4
3
2
1
0
ChatGPT-4o
DeepSeek
Gemini-2.5-Pro
OpenEvidence
Zhipu
AI Model

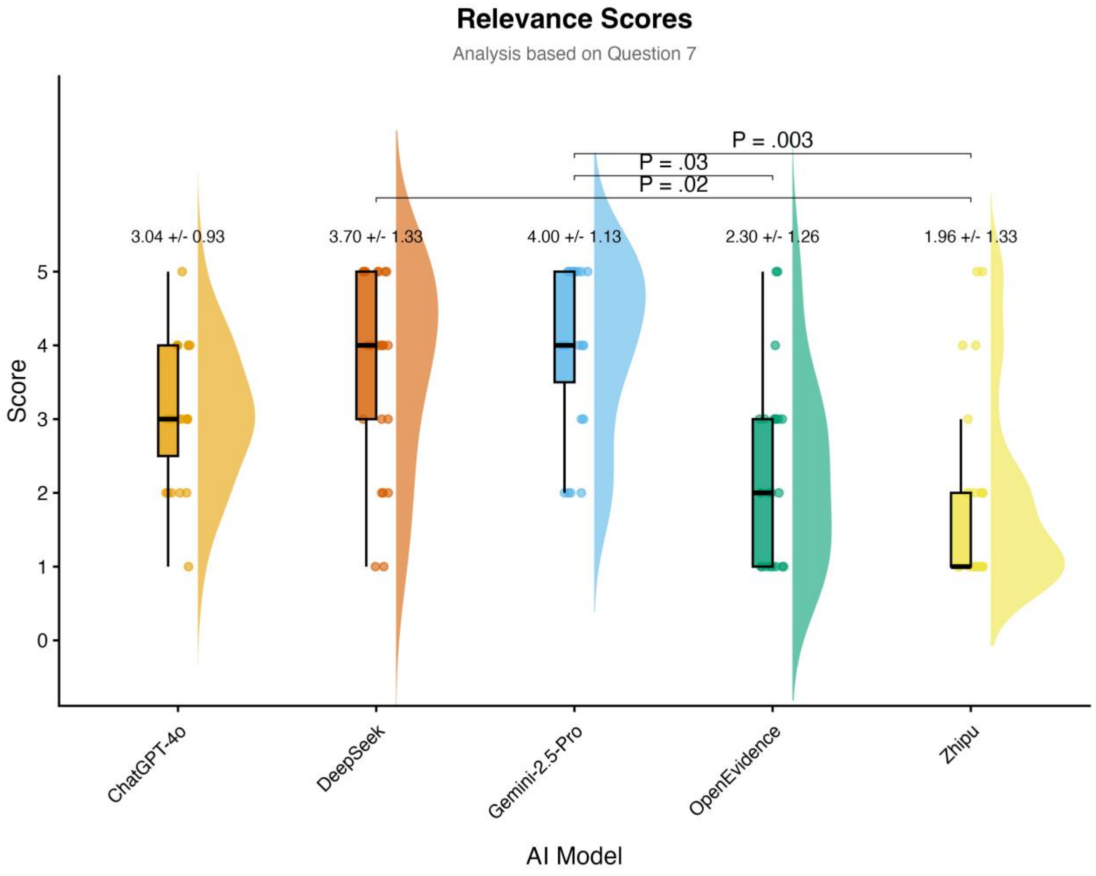
Relevance Scores
Analysis based on Question 7
P = .003
P = .03
P = .02
3.04 +/- 0.93
3.70 +/- 1.33
4.00 +/- 1.13
2.30 +/- 1.26
1.96 +/- 1.33
Score
0
1
2
3
4
5
ChatGPT-4o
DeepSeek
Gemini-2.5-Pro
OpenEvidence
Zhipu
AI Model

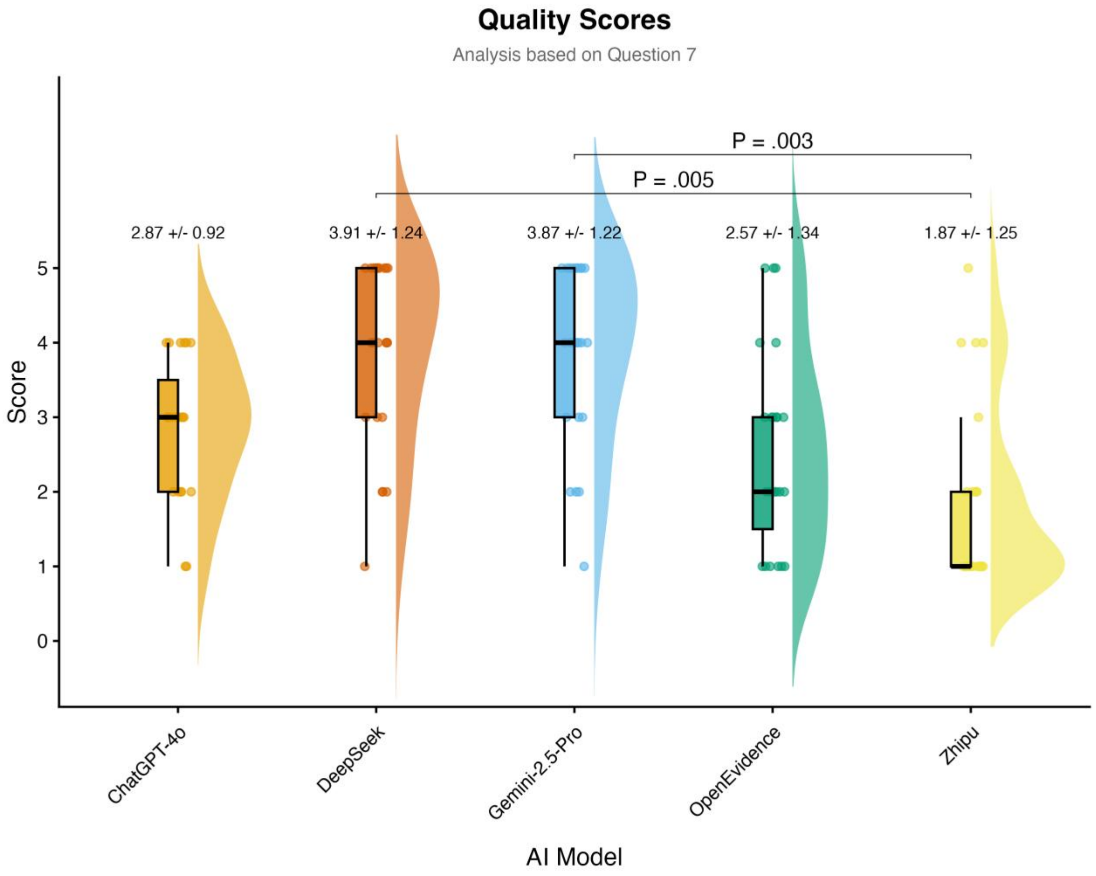
Quality Scores
Analysis based on Question 7
P = .003
P = .005
2.87 +/- 0.92
3.91 +/- 1.24
3.87 +/- 1.22
2.57 +/- 1.34
1.87 +/- 1.25
Score
0
1
2
3
4
5
ChatGPT-4o
DeepSeek
Gemini-2.5-Pro
OpenEvidence
Zhipu
AI Model

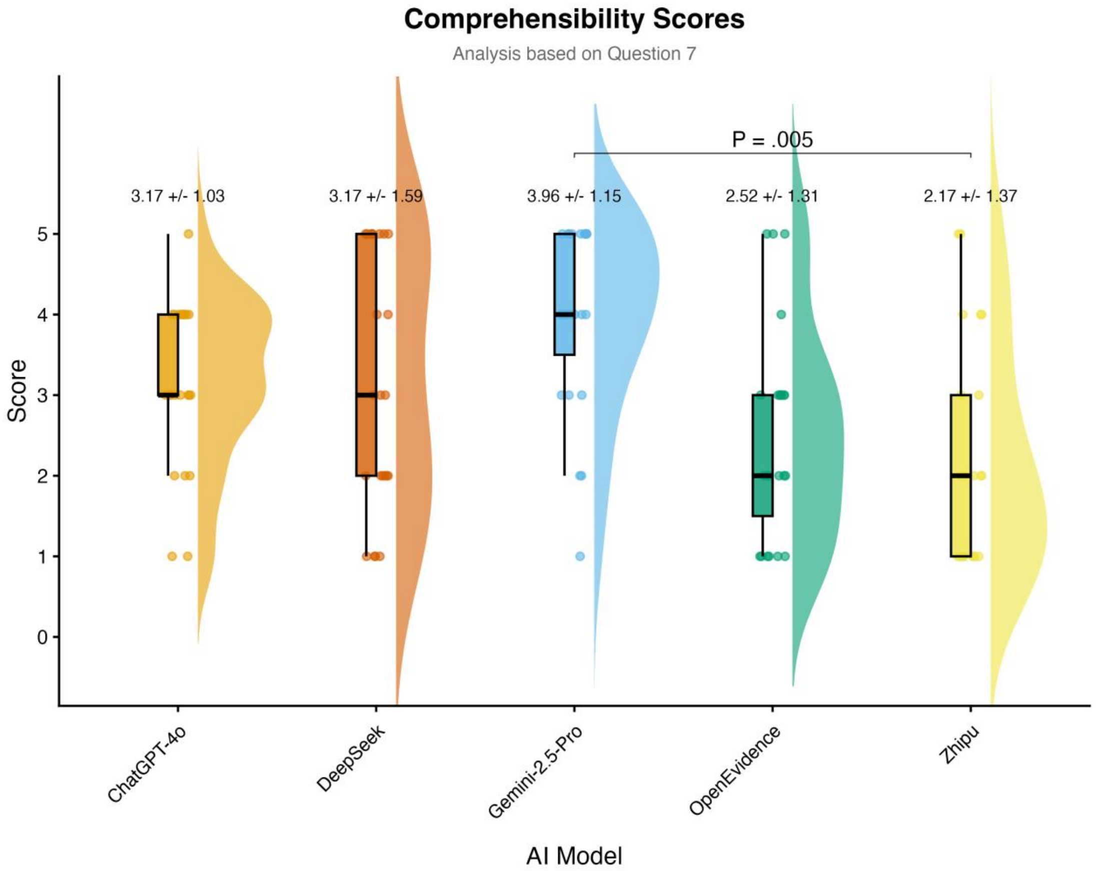
Comprehensibility Scores
Analysis based on Question 7
P = .005
3.17 +/- 1.03
3.17 +/- 1.59
3.96 +/- 1.15
2.52 +/- 1.31
2.17 +/- 1.37
Score
5
4
3
2
1
0
ChatGPT-4o
DeepSeek
Gemini-2.5-Pro
OpenEvidence
Zhipu
AI Model

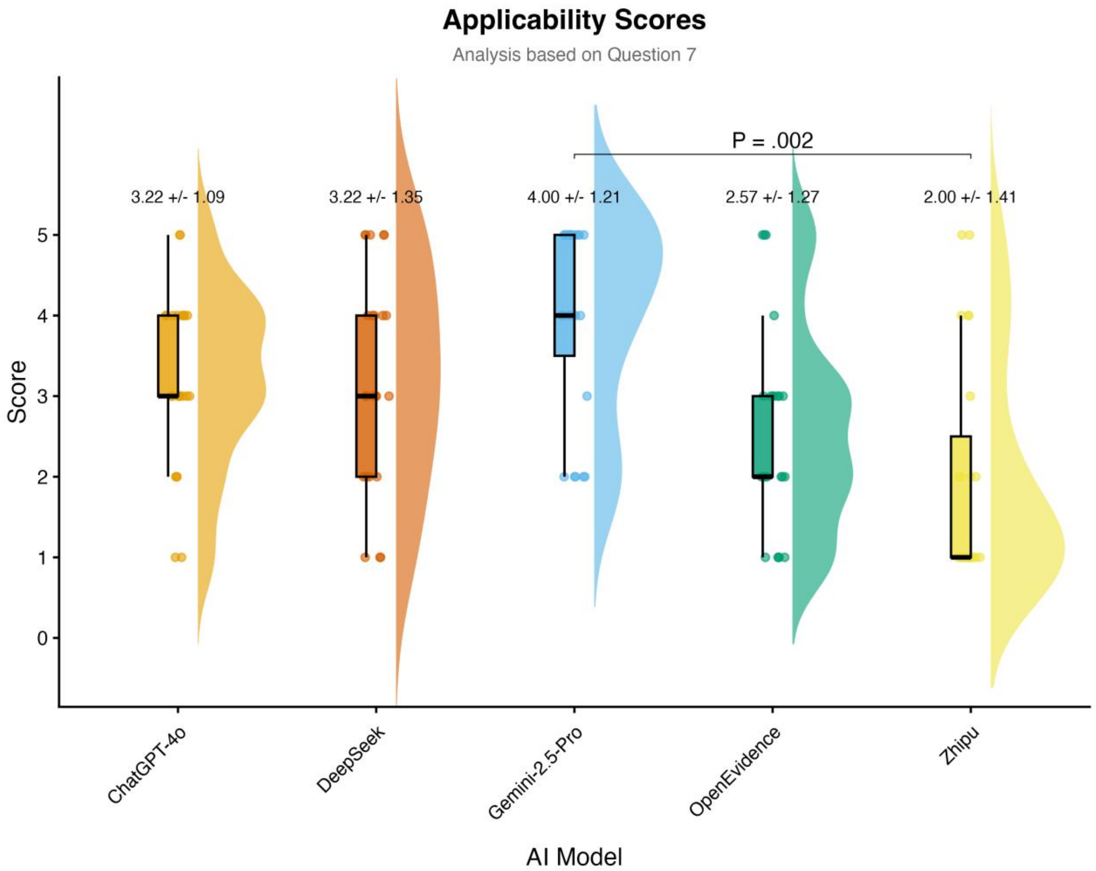
Applicability Scores
Analysis based on Question 7
P = .002
3.22 +/- 1.09
3.22 +/- 1.35
4.00 +/- 1.21
2.57 +/- 1.27
2.00 +/- 1.41
Score
5
4
3
2
1
0
ChatGPT-4o
DeepSeek
Gemini-2.5-Pro
OpenEvidence
Zhipu
AI Model

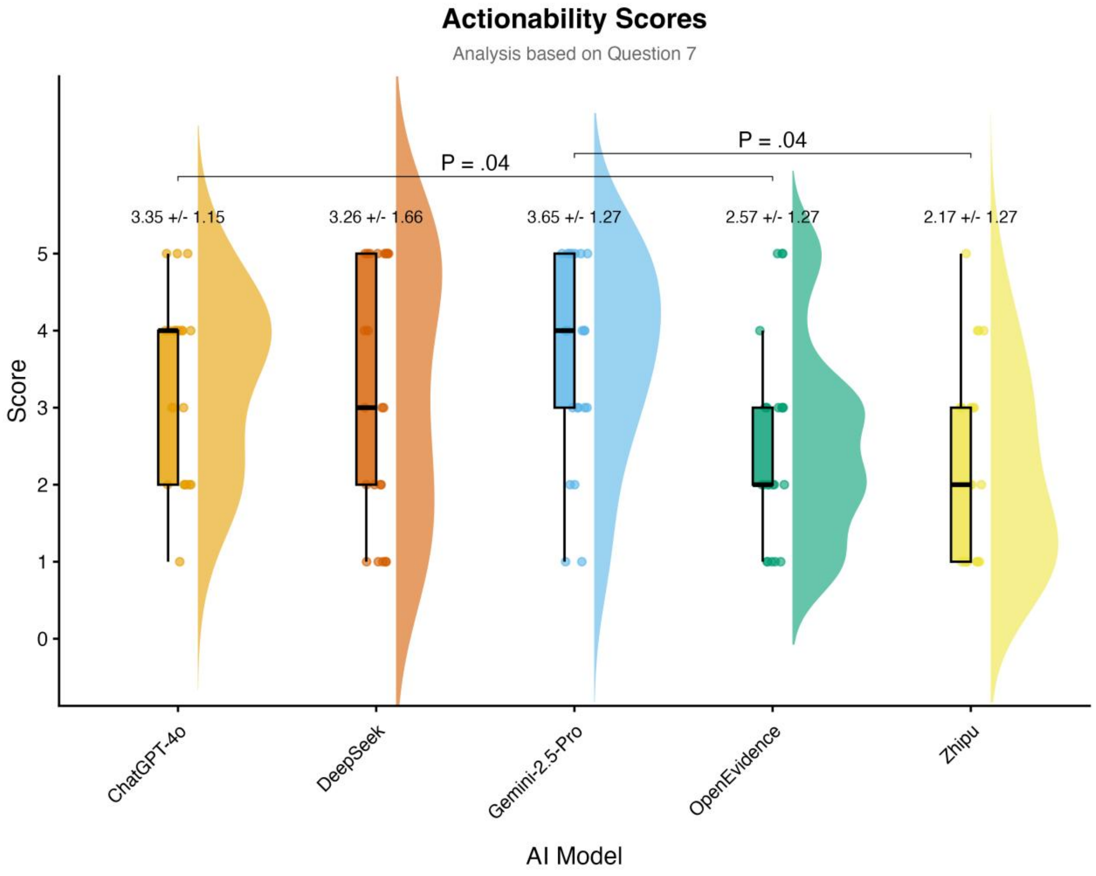
Actionability Scores
Analysis based on Question 7
P = .04
P = .04
3.35 +/- 1.15
3.26 +/- 1.66
3.65 +/- 1.27
2.57 +/- 1.27
2.17 +/- 1.27
Score
5
4
3
2
1
0
ChatGPT-4o
DeepSeek
Gemini-2.5-Pro
OpenEvidence
Zhipu
AI Model

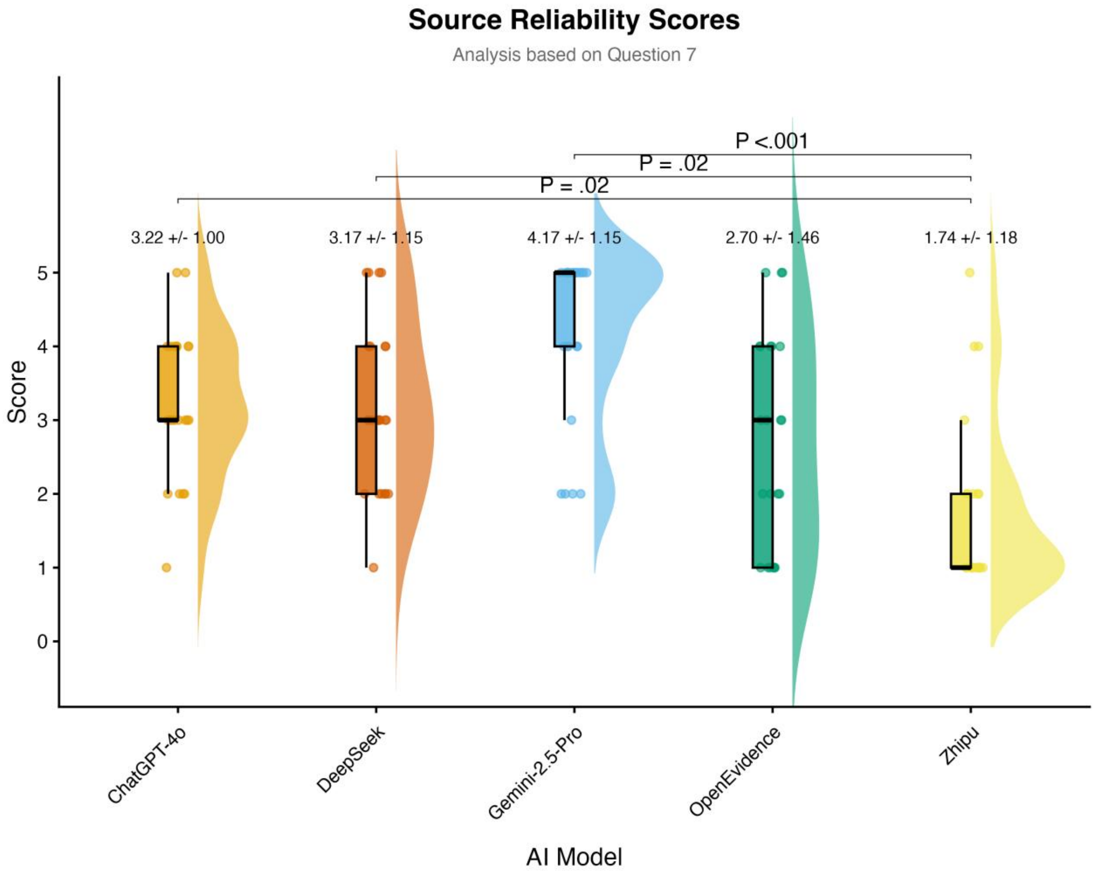
Source Reliability Scores
Analysis based on Question 7
P <.001
P = .02
P = .02
3.22 +/- 1.00
3.17 +/- 1.15
4.17 +/- 1.15
2.70 +/- 1.46
1.74 +/- 1.18
Score
5
4
3
2
1
0
ChatGPT-4o
DeepSeek
Gemini-2.5-Pro
OpenEvidence
Zhipu
AI Model

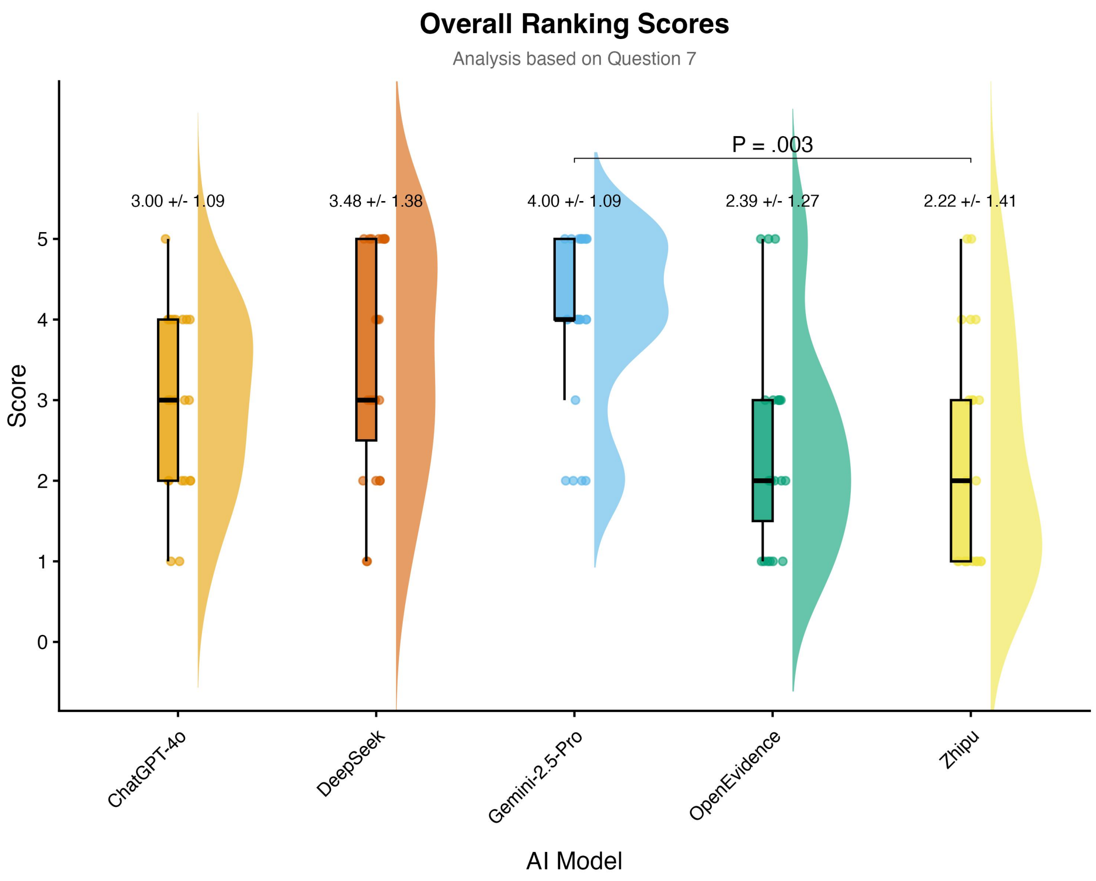
Overall Ranking Scores
Analysis based on Question 7
P = .003
3.00 +/- 1.09
3.48 +/- 1.38
4.00 +/- 1.09
2.39 +/- 1.27
2.22 +/- 1.41
Score
0
1
2
3
4
5
ChatGPT-4o
DeepSeek
Gemini-2.5-Pro
OpenEvidence
Zhipu
AI Model

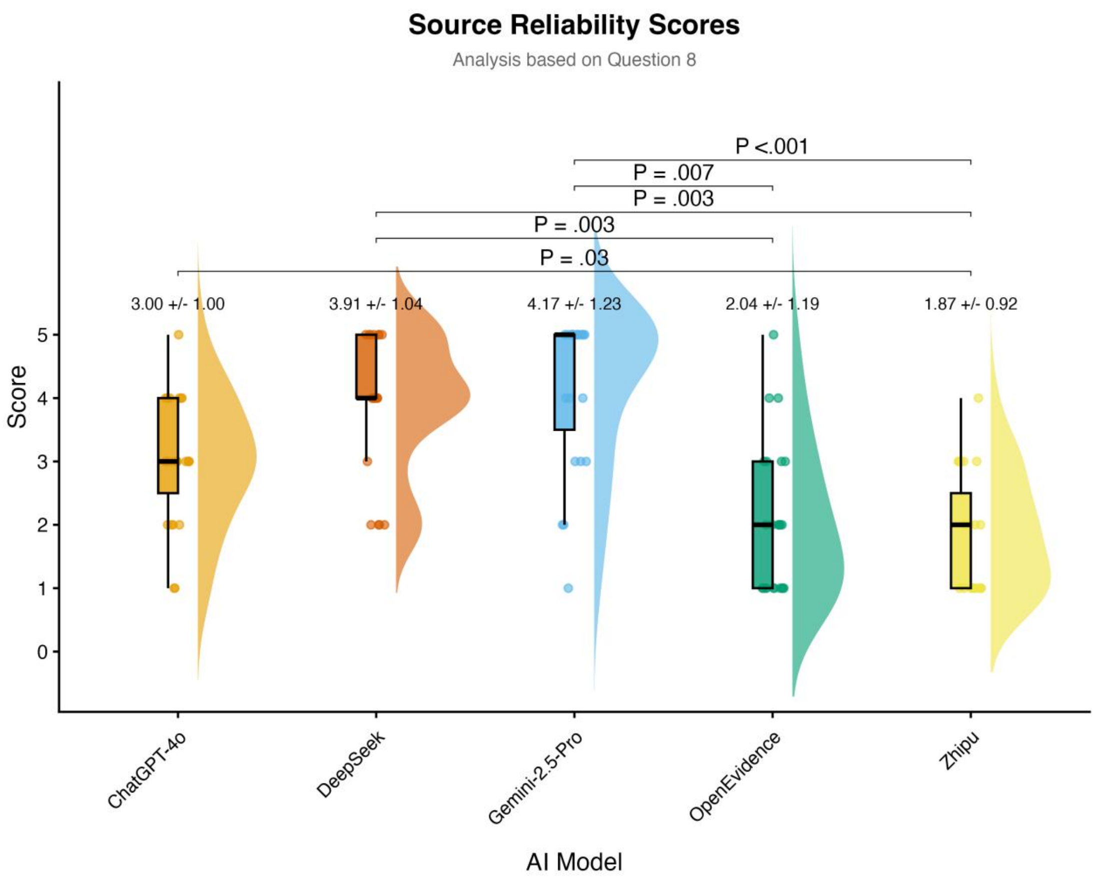
Source Reliability Scores
Analysis based on Question 8
P <.001
P = .007
P = .003
P = .003
P = .03
3.00 +/- 1.00
3.91 +/- 1.04
4.17 +/- 1.23
2.04 +/- 1.19
1.87 +/- 0.92
Score
0
1
2
3
4
5
ChatGPT-4o
DeepSeek
Gemini-2.5-Pro
OpenEvidence
Zhipu
AI Model

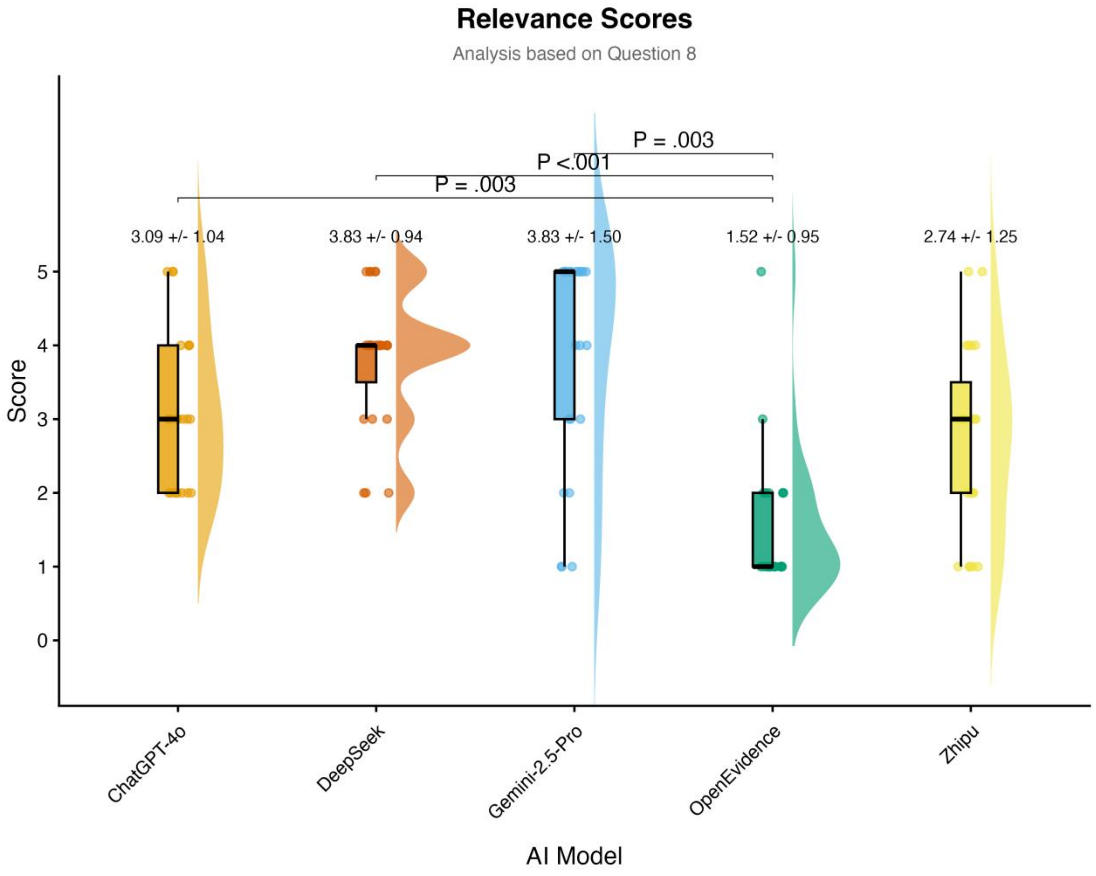

Relevance Scores
Analysis based on Question 8
P = .003
P <.001
P = .003
3.09 +/- 1.04
3.83 +/- 0.94
3.83 +/- 1.50
1.52 +/- 0.95
2.74 +/- 1.25
Score
AI Model
ChatGPT-4o
DeepSeek
Gemini-2.5-Pro
OpenEvidence
Zhipu

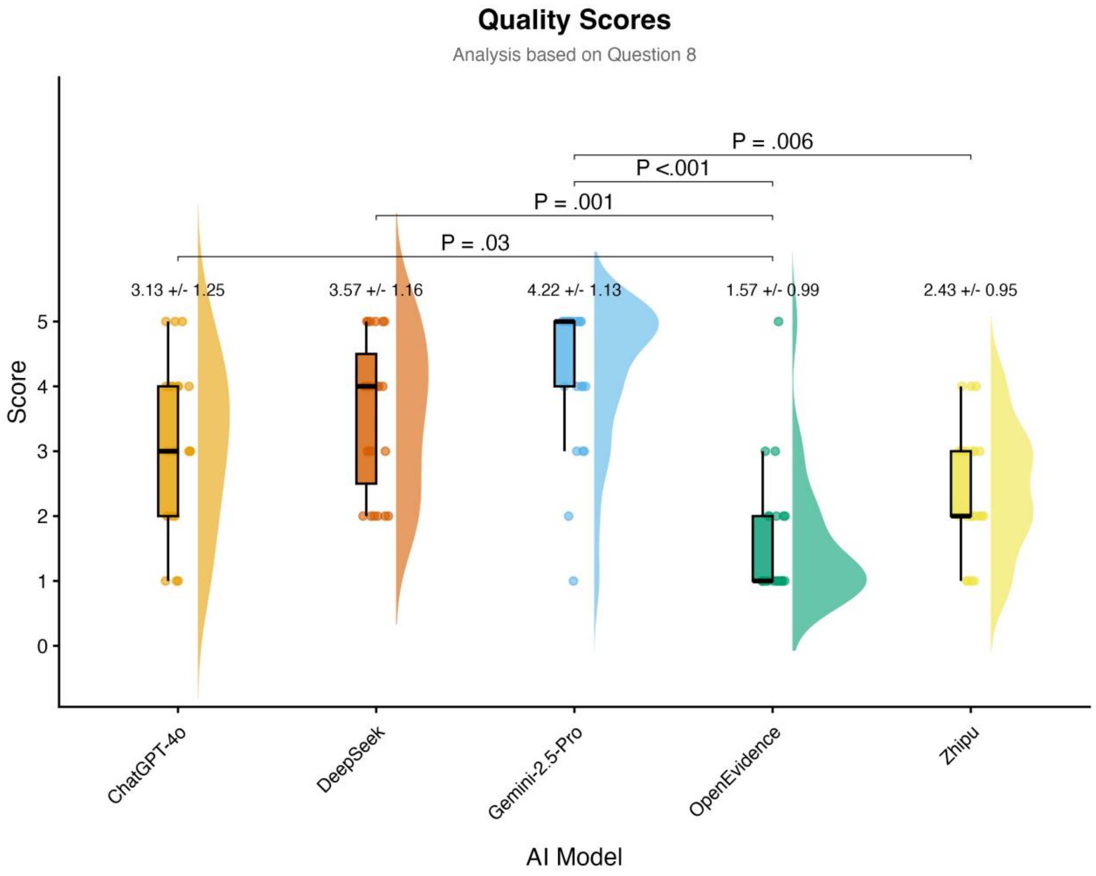

Quality Scores
Analysis based on Question 8
P = .006
P <.001
P = .001
P = .03
3.13 +/- 1.25
3.57 +/- 1.16
4.22 +/- 1.13
1.57 +/- 0.99
2.43 +/- 0.95
Score
AI Model
ChatGPT-4o
DeepSeek
Gemini-2.5-Pro
OpenEvidence
Zhipu

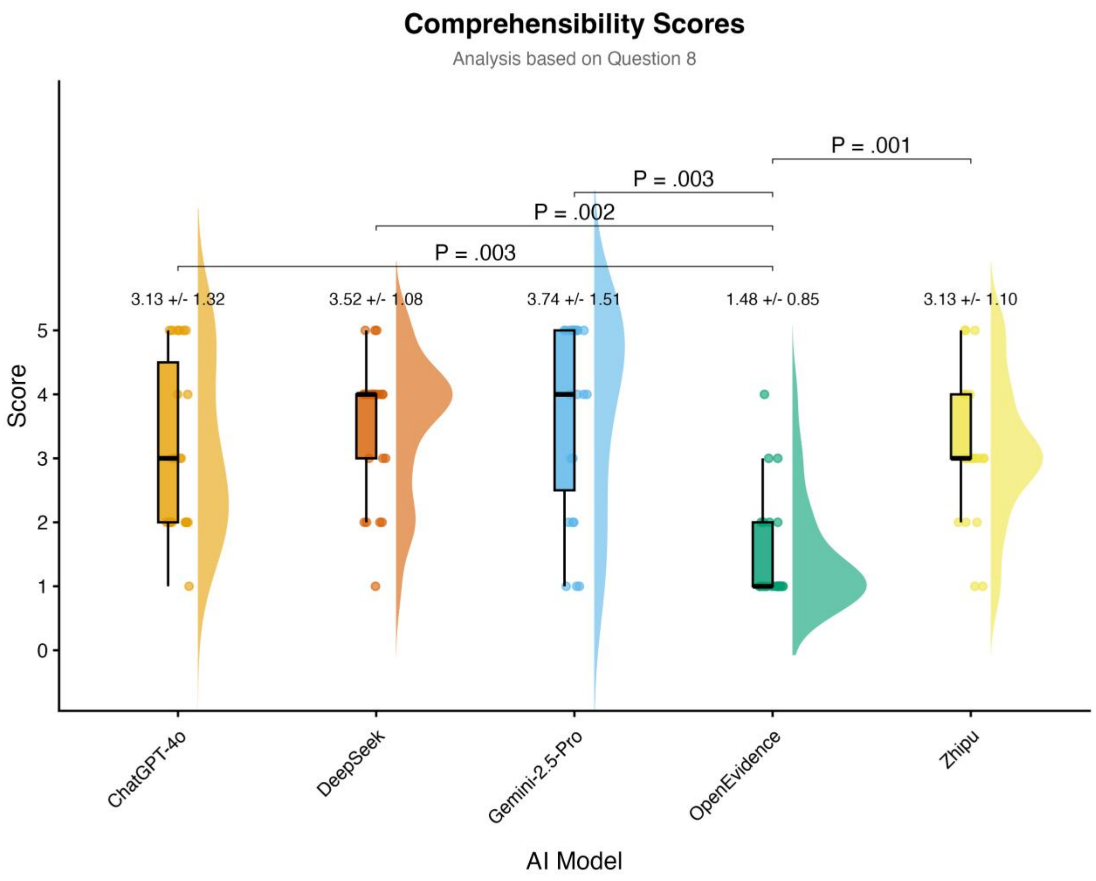
Comprehensibility Scores
Analysis based on Question 8
P = .001
P = .003
P = .002
P = .003
3.13 +/- 1.32
3.52 +/- 1.08
3.74 +/- 1.51
1.48 +/- 0.85
3.13 +/- 1.10
Score
5
4
3
2
1
0
ChatGPT-4o
DeepSeek
Gemini-2.5-Pro
OpenEvidence
Zhipu
AI Model

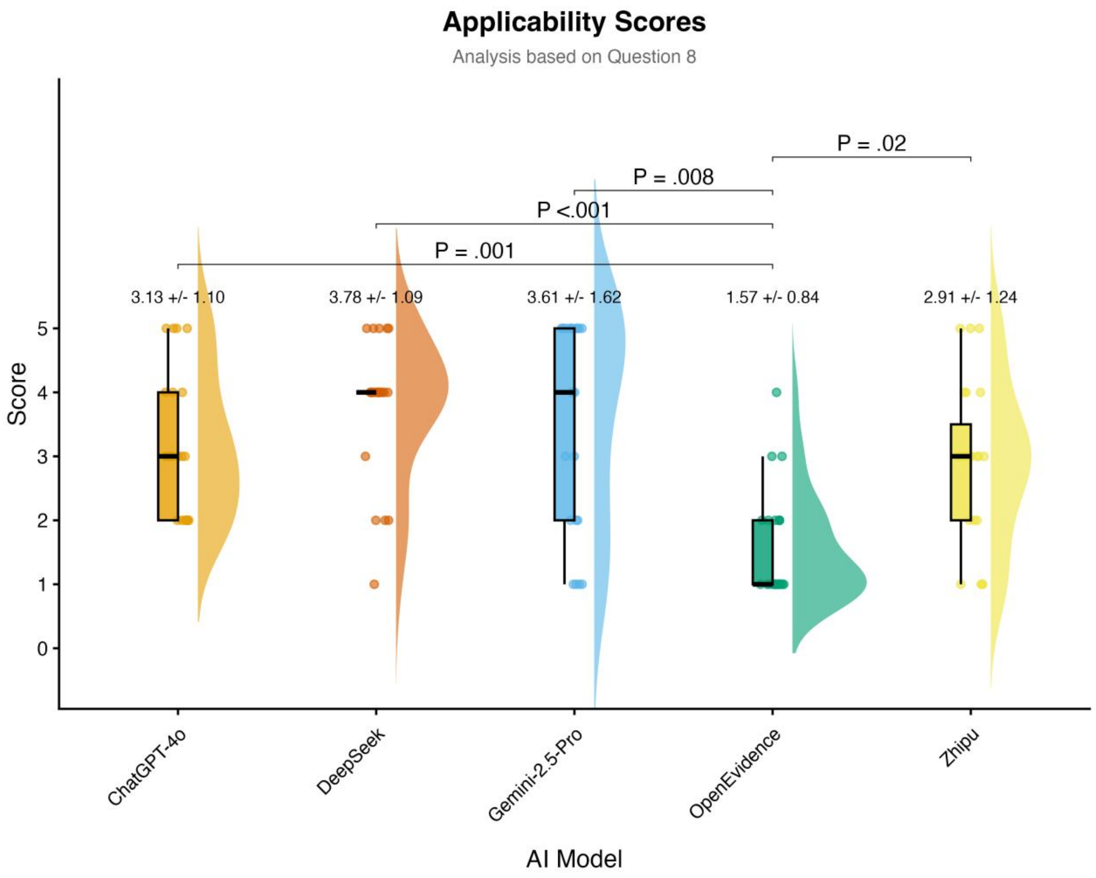
Applicability Scores
Analysis based on Question 8
P = .02
P = .008
P <.001
P = .001
3.13 +/- 1.10
3.78 +/- 1.09
3.61 +/- 1.62
1.57 +/- 0.84
2.91 +/- 1.24
Score
5
4
3
2
1
0
ChatGPT-4o
DeepSeek
Gemini-2.5-Pro
OpenEvidence
Zhipu
AI Model

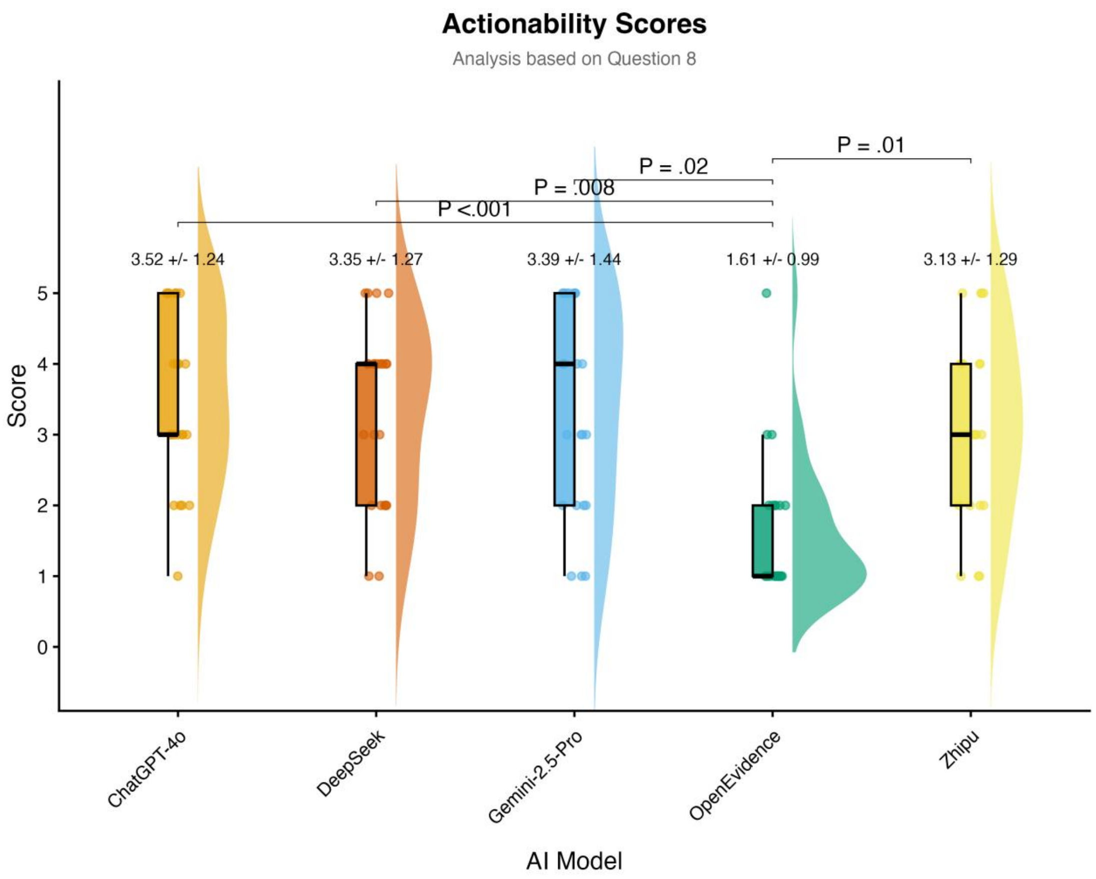
Actionability Scores
Analysis based on Question 8
P = .01
P = .02
P = .008
P <.001
3.52 +/- 1.24
3.35 +/- 1.27
3.39 +/- 1.44
1.61 +/- 0.99
3.13 +/- 1.29
Score
0
1
2
3
4
5
ChatGPT-4o
DeepSeek
Gemini-2.5-Pro
OpenEvidence
Zhipu
AI Model

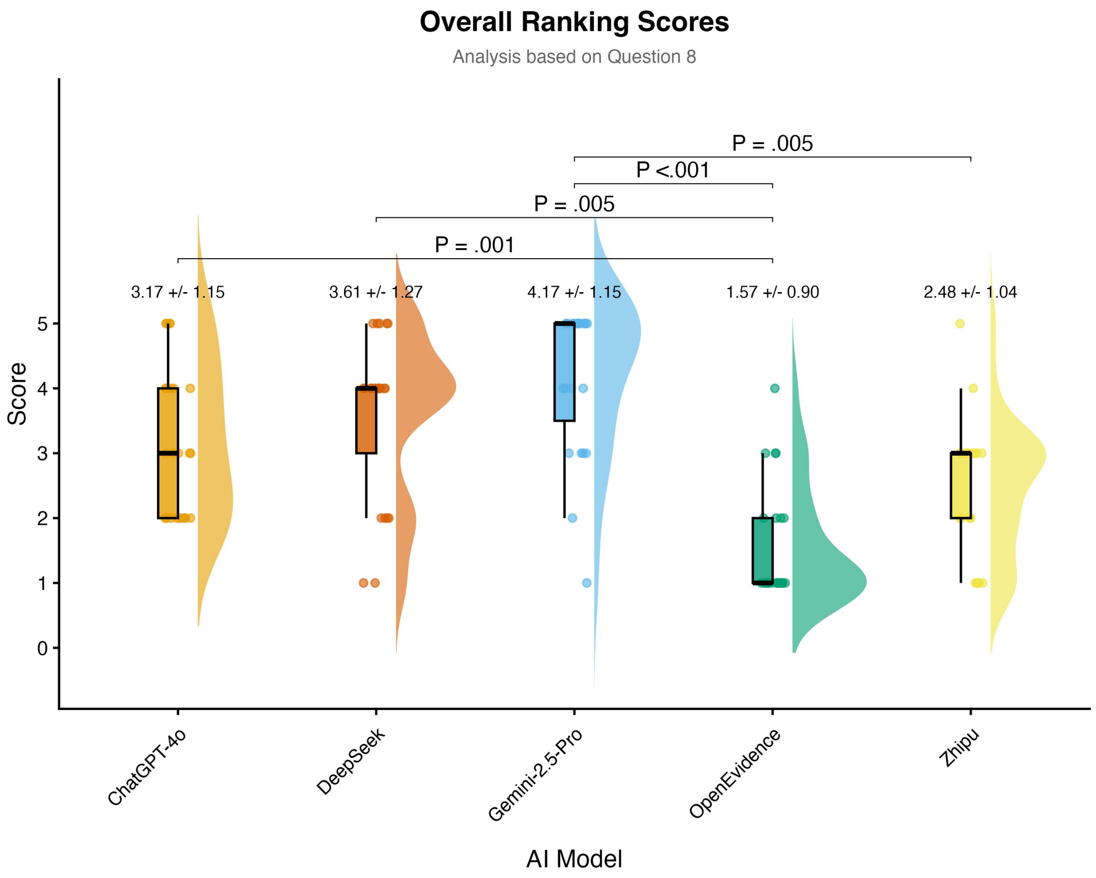
Overall Ranking Scores
Analysis based on Question 8
P = .005
P <.001
P = .005
P = .001
3.17 +/- 1.15
3.61 +/- 1.27
4.17 +/- 1.15
1.57 +/- 0.90
2.48 +/- 1.04
Score
0
1
2
3
4
5
ChatGPT-4o
DeepSeek
Gemini-2.5-Pro
OpenEvidence
Zhipu
AI Model

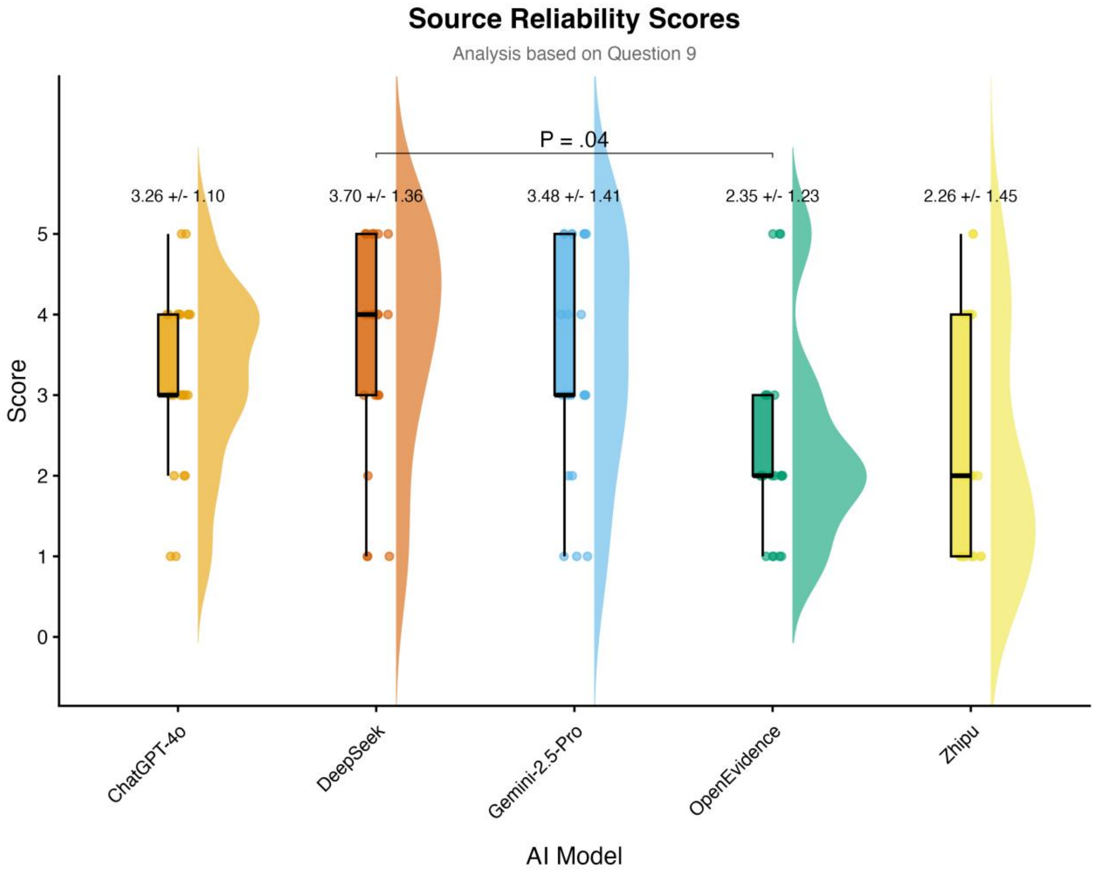
Source Reliability Scores
Analysis based on Question 9
P = .04
3.26 +/- 1.10
3.70 +/- 1.36
3.48 +/- 1.41
2.35 +/- 1.23
2.26 +/- 1.45
Score
AI Model
ChatGPT-4o
DeepSeek
Gemini-2.5-Pro
OpenEvidence
Zhipu

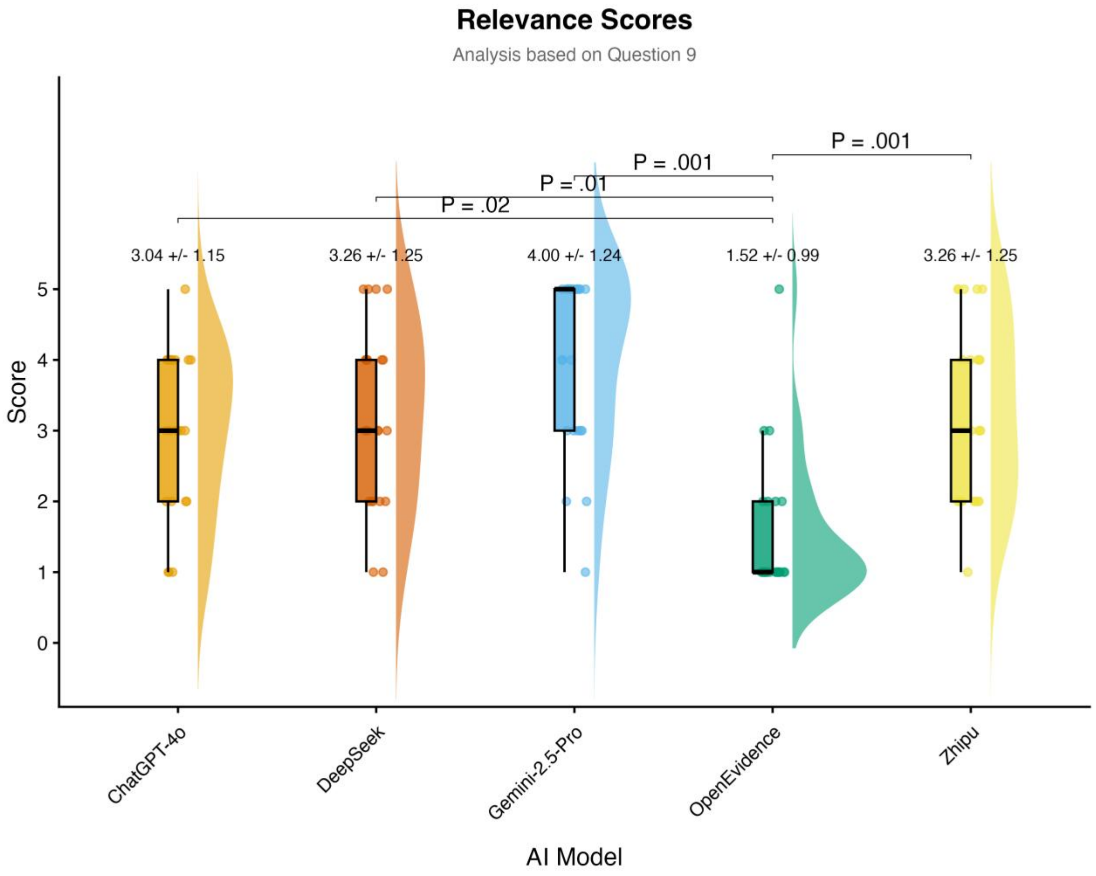
Relevance Scores
Analysis based on Question 9
P = .001
P = .001
P = .01
P = .02
3.04 +/- 1.15
3.26 +/- 1.25
4.00 +/- 1.24
1.52 +/- 0.99
3.26 +/- 1.25
Score
AI Model
ChatGPT-4o
DeepSeek
Gemini-2.5-Pro
OpenEvidence
Zhipu

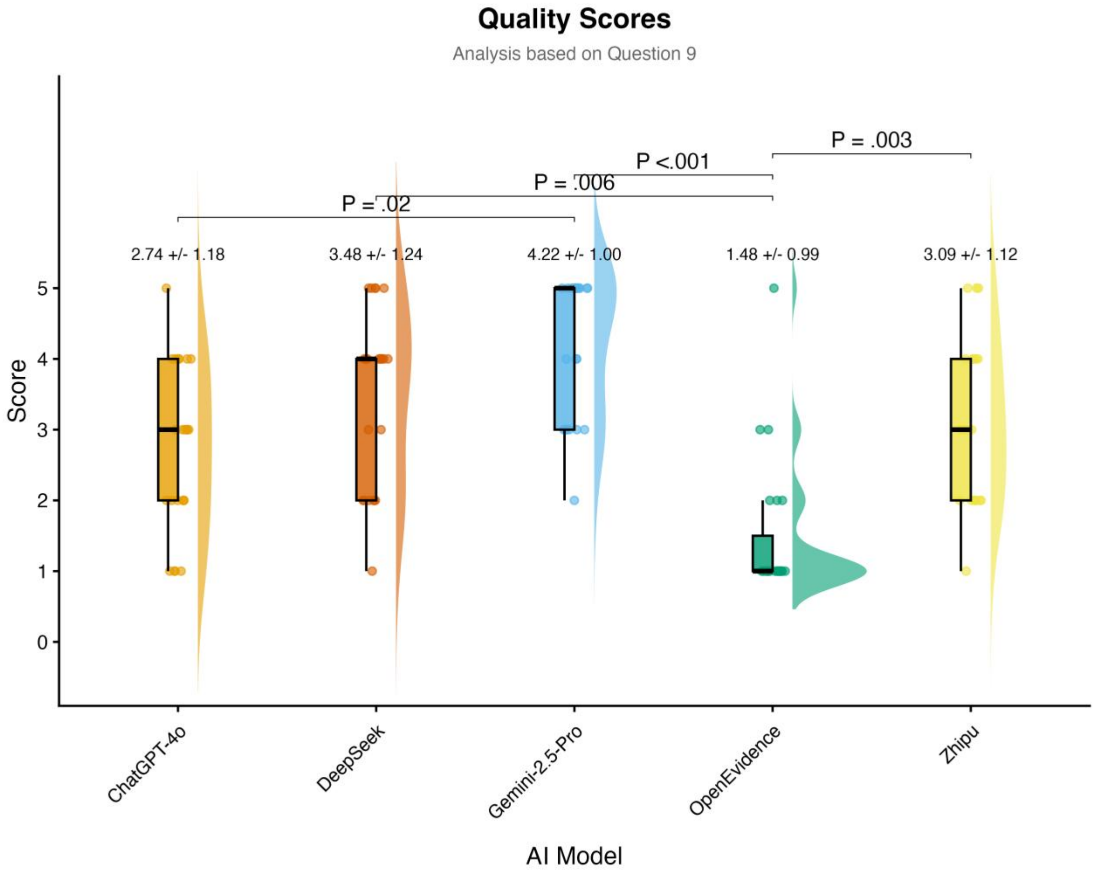
Quality Scores
Analysis based on Question 9
P = .003
P <.001
P = .006
P = .02
2.74 +/- 1.18
3.48 +/- 1.24
4.22 +/- 1.00
1.48 +/- 0.99
3.09 +/- 1.12
Score
5
4
3
2
1
0
ChatGPT-4o
DeepSeek
Gemini-2.5-Pro
OpenEvidence
Zhipu
AI Model

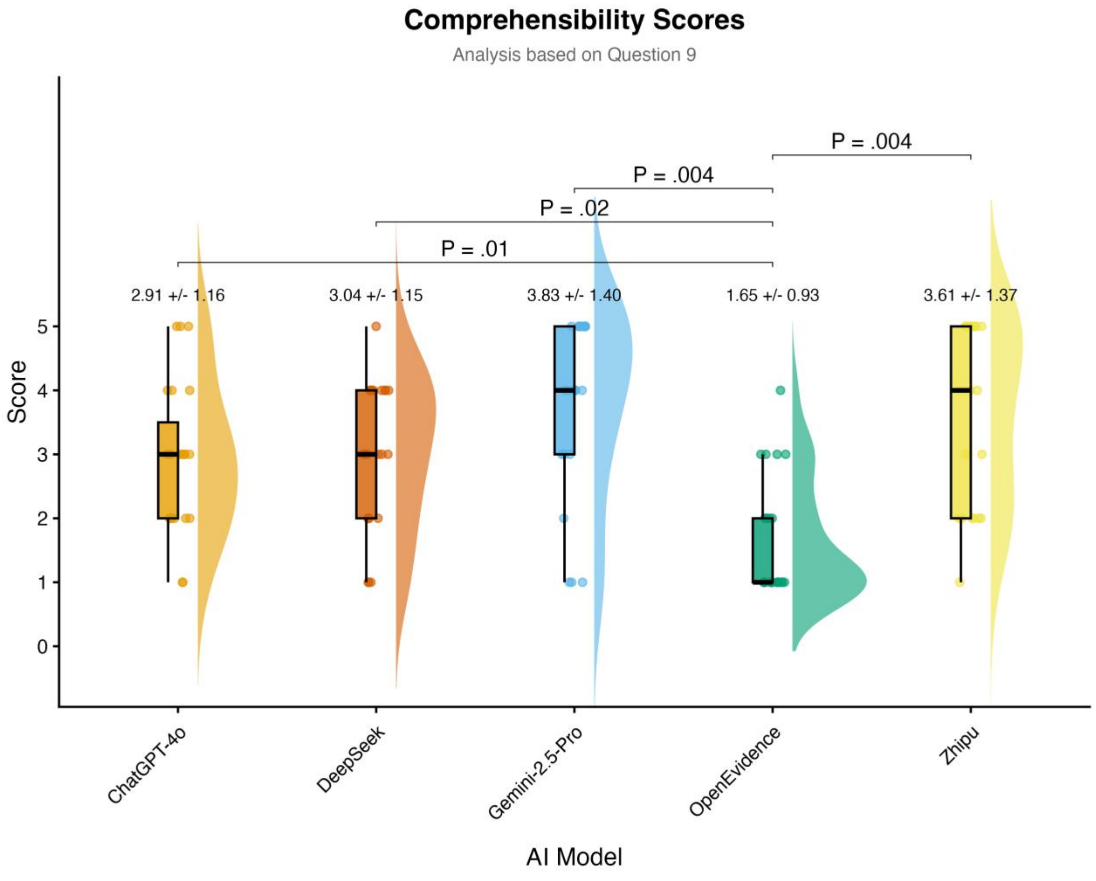
Comprehensibility Scores
Analysis based on Question 9
P = .004
P = .004
P = .02
P = .01
2.91 +/- 1.16
3.04 +/- 1.15
3.83 +/- 1.40
1.65 +/- 0.93
3.61 +/- 1.37
Score
5
4
3
2
1
0
ChatGPT-4o
DeepSeek
Gemini-2.5-Pro
OpenEvidence
Zhipu
AI Model

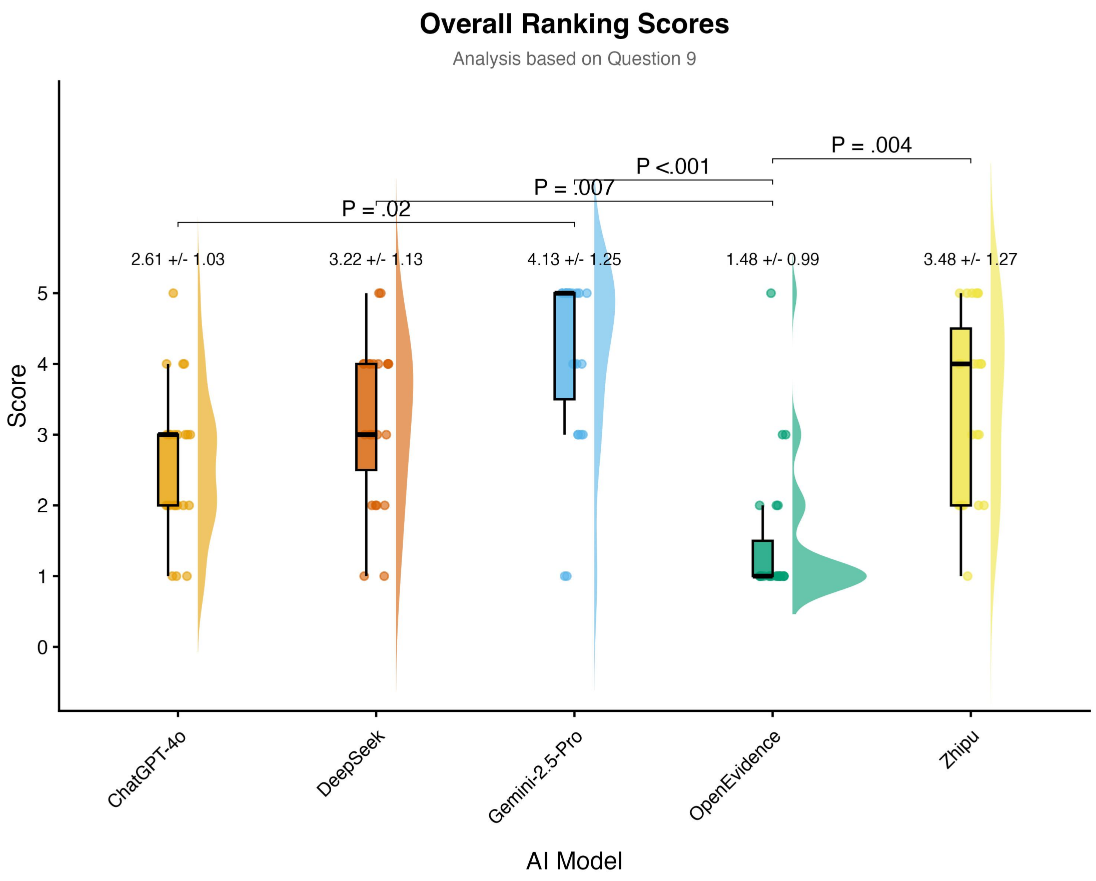

Overall Ranking Scores
Analysis based on Question 9
P = .004
P <.001
P = .007
P = .02
2.61 +/- 1.03
3.22 +/- 1.13
4.13 +/- 1.25
1.48 +/- 0.99
3.48 +/- 1.27
Score
AI Model
ChatGPT-4o
DeepSeek
Gemini-2.5-Pro
OpenEvidence
Zhipu

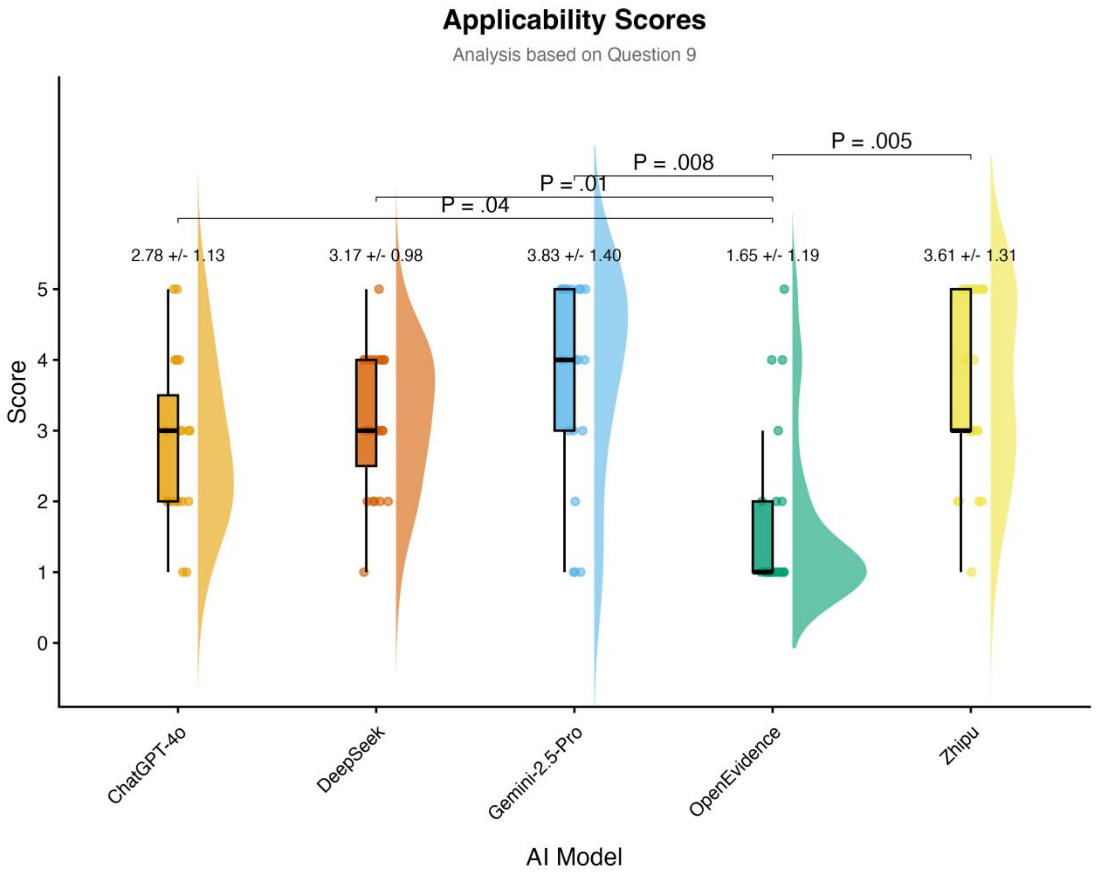

Applicability Scores
Analysis based on Question 9
P = .005
P = .008
P = .01
P = .04
2.78 +/- 1.13
3.17 +/- 0.98
3.83 +/- 1.40
1.65 +/- 1.19
3.61 +/- 1.31
Score
AI Model
ChatGPT-4o
DeepSeek
Gemini-2.5-Pro
OpenEvidence
Zhipu

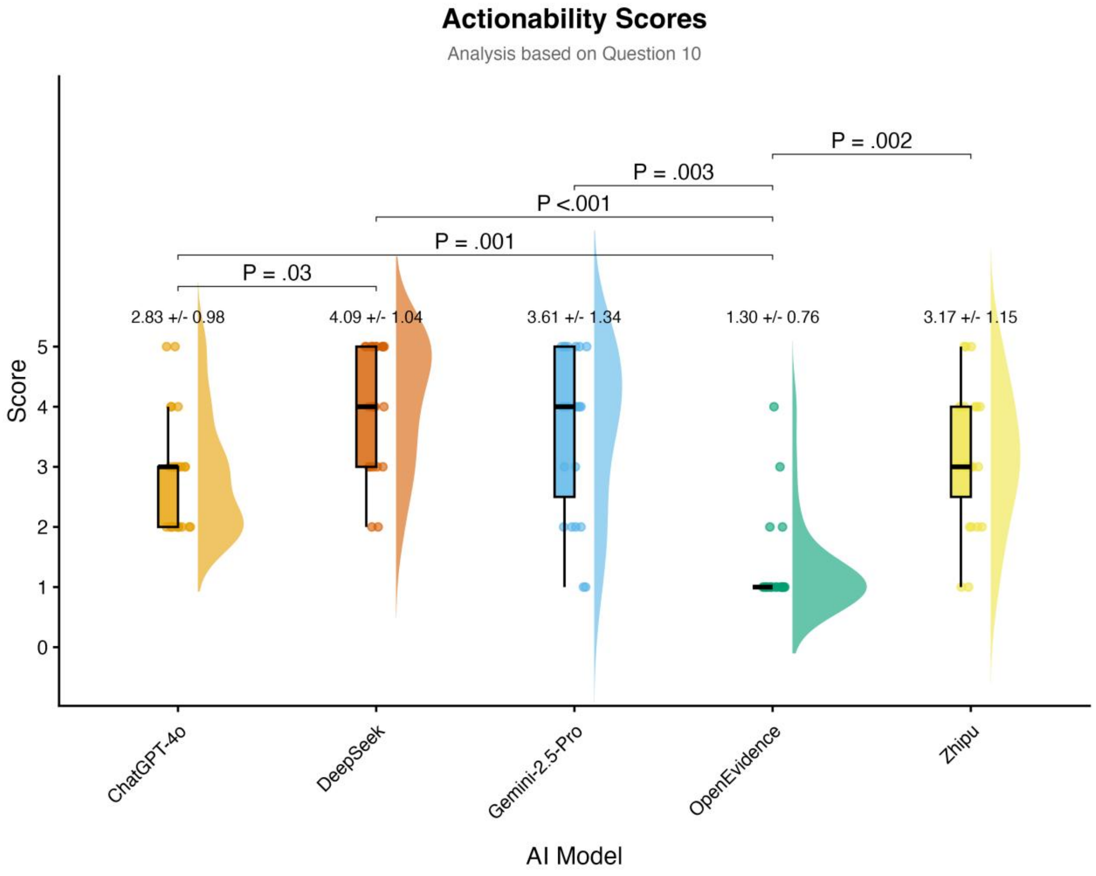
Actionability Scores
Analysis based on Question 10
P = .002
P = .003
P <.001
P = .001
P = .03
2.83 +/- 0.98
4.09 +/- 1.04
3.61 +/- 1.34
1.30 +/- 0.76
3.17 +/- 1.15
Score
5
4
3
2
1
0
ChatGPT-4o
DeepSeek
Gemini-2.5-Pro
OpenEvidence
Zhipu
AI Model

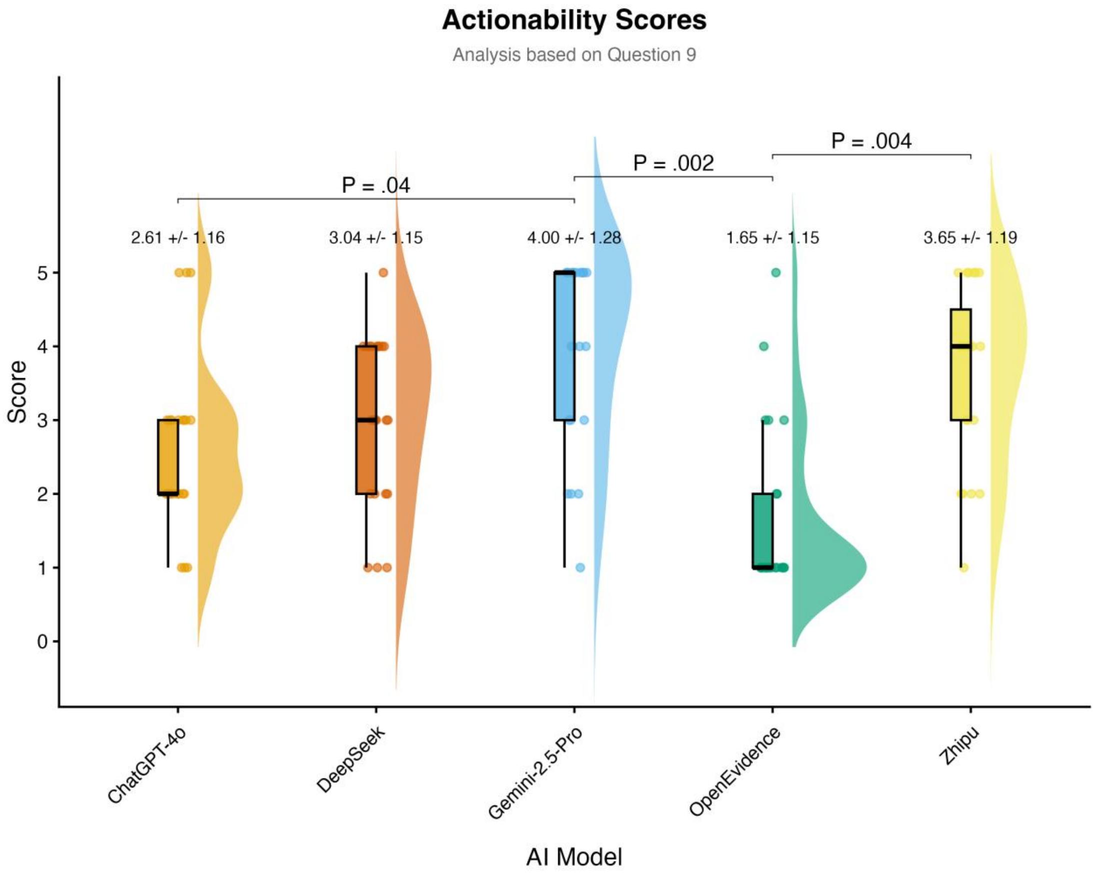
Actionability Scores
Analysis based on Question 9
P = .004
P = .002
P = .04
2.61 +/- 1.16
3.04 +/- 1.15
4.00 +/- 1.28
1.65 +/- 1.15
3.65 +/- 1.19
Score
5
4
3
2
1
0
ChatGPT-4o
DeepSeek
Gemini-2.5-Pro
OpenEvidence
Zhipu
AI Model

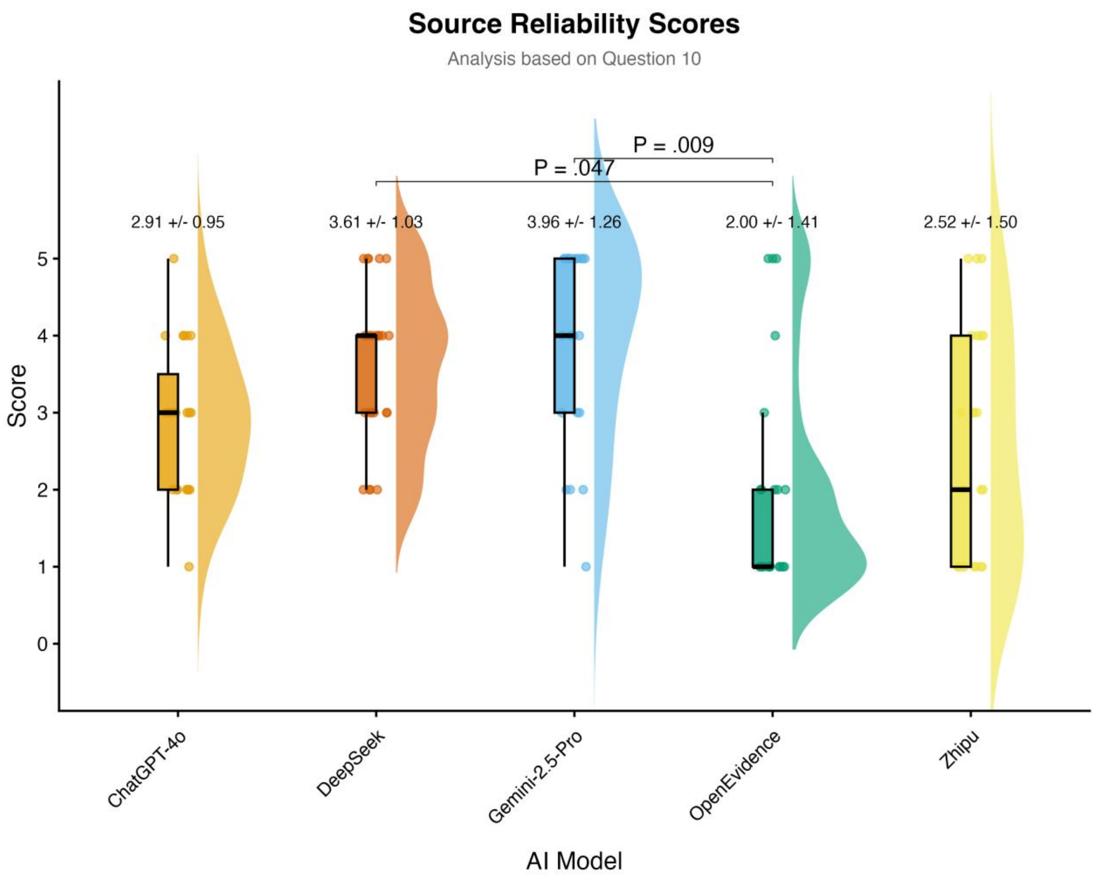
Source Reliability Scores
Analysis based on Question 10
P = .009
P = .047
2.91 +/- 0.95
3.61 +/- 1.03
3.96 +/- 1.26
2.00 +/- 1.41
2.52 +/- 1.50
Score
5
4
3
2
1
0
ChatGPT-4o
DeepSeek
Gemini-2.5-Pro
OpenEvidence
Zhipu
AI Model

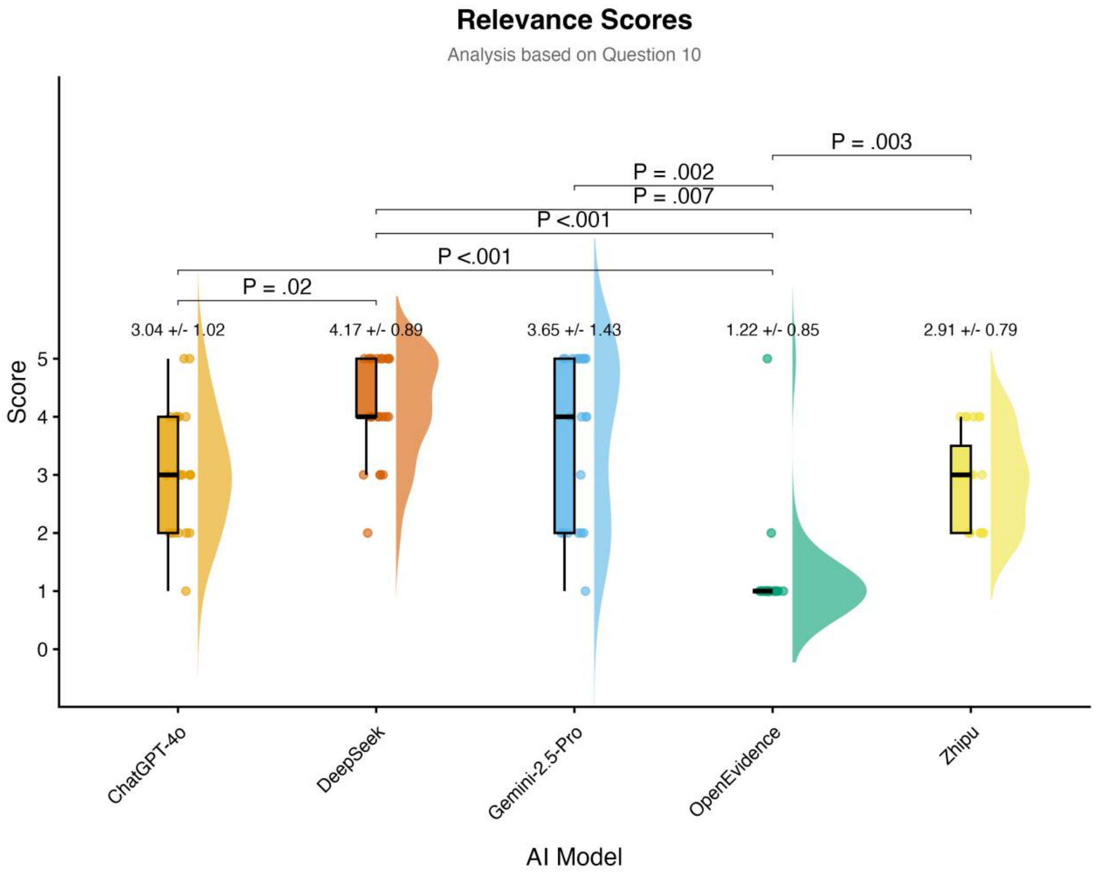
Relevance Scores
Analysis based on Question 10
P = .003
P = .002
P = .007
P <.001
P <.001
P = .02
3.04 +/- 1.02
4.17 +/- 0.89
3.65 +/- 1.43
1.22 +/- 0.85
2.91 +/- 0.79
Score
5
4
3
2
1
0
ChatGPT-4o
DeepSeek
Gemini-2.5-Pro
OpenEvidence
Zhipu
AI Model

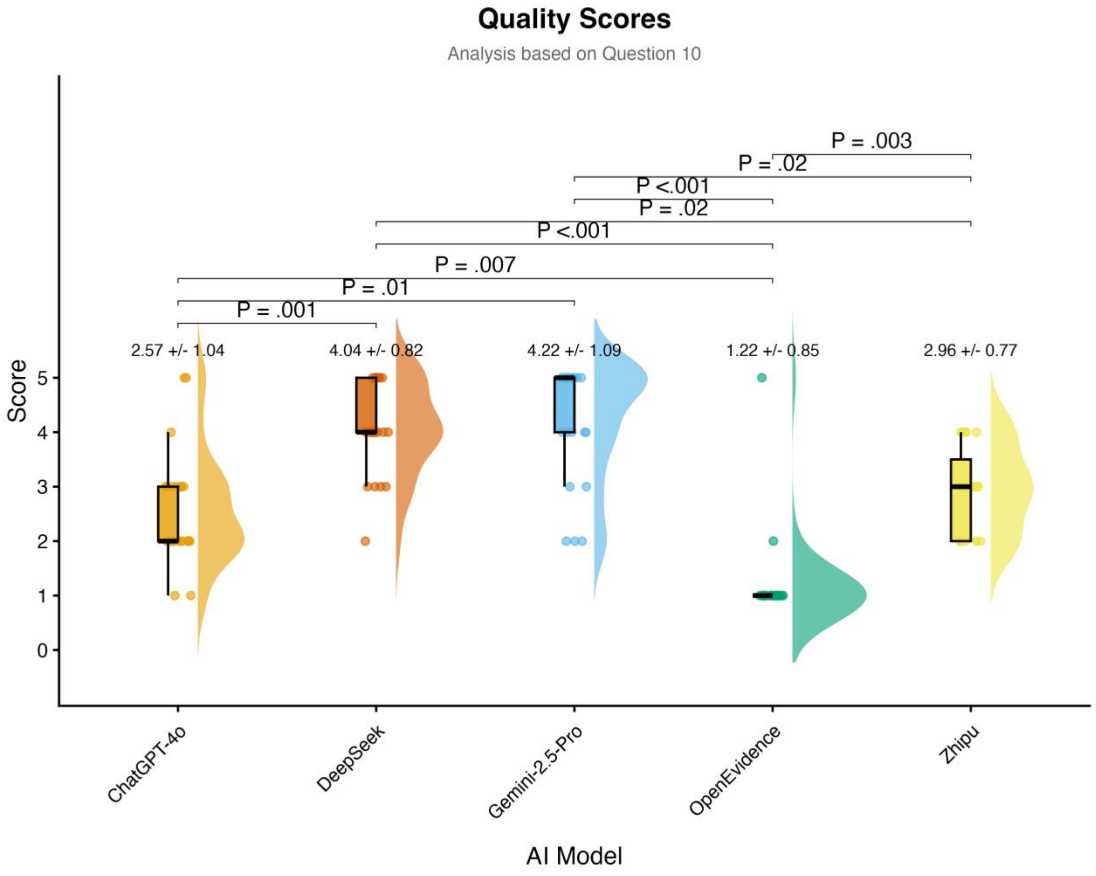
Quality Scores
Analysis based on Question 10
P = .003
P = .02
P <.001
P = .02
P <.001
P = .007
P = .01
P = .001
2.57 +/- 1.04
4.04 +/- 0.82
4.22 +/- 1.09
1.22 +/- 0.85
2.96 +/- 0.77
Score
5
4
3
2
1
0
ChatGPT-4o
DeepSeek
Gemini-2.5-Pro
OpenEvidence
Zhipu
AI Model

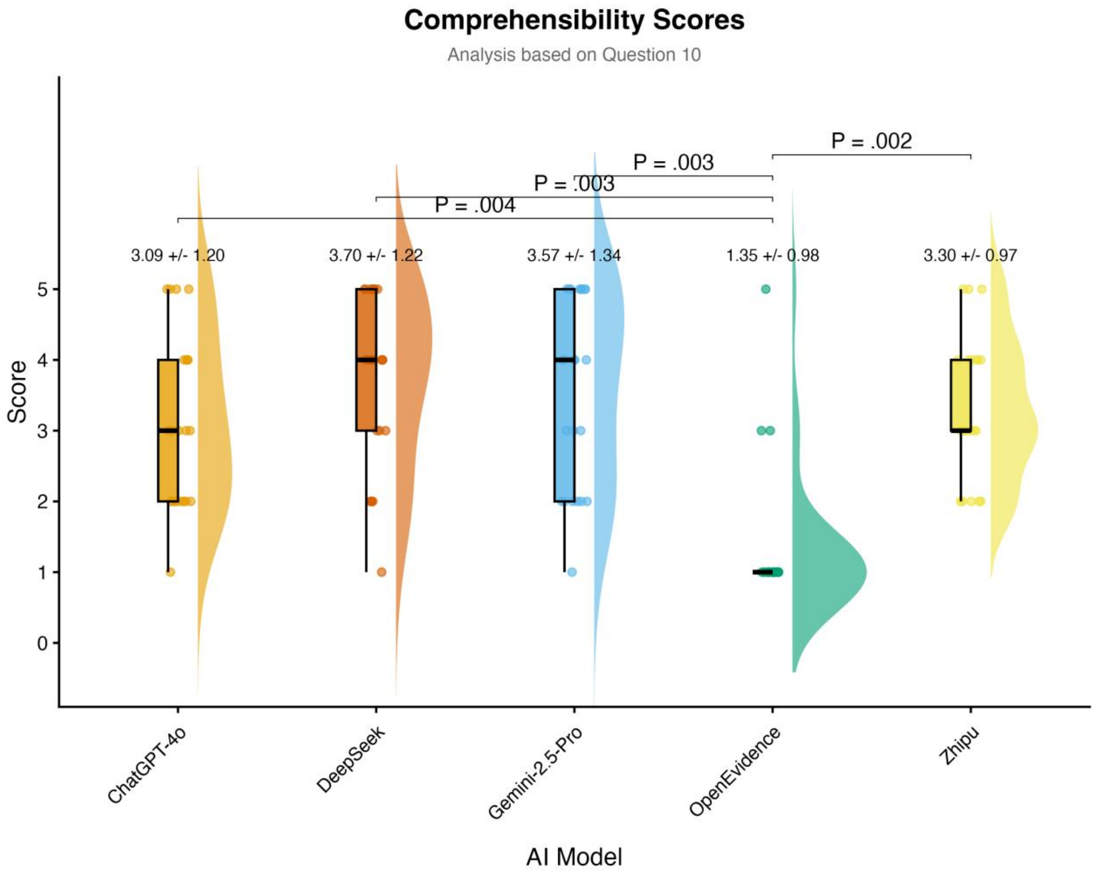
Comprehensibility Scores
Analysis based on Question 10
P = .002
P = .003
P = .003
P = .004
3.09 +/- 1.20
3.70 +/- 1.22
3.57 +/- 1.34
1.35 +/- 0.98
3.30 +/- 0.97
Score
5
4
3
2
1
0
ChatGPT-4o
DeepSeek
Gemini-2.5-Pro
OpenEvidence
Zhipu
AI Model

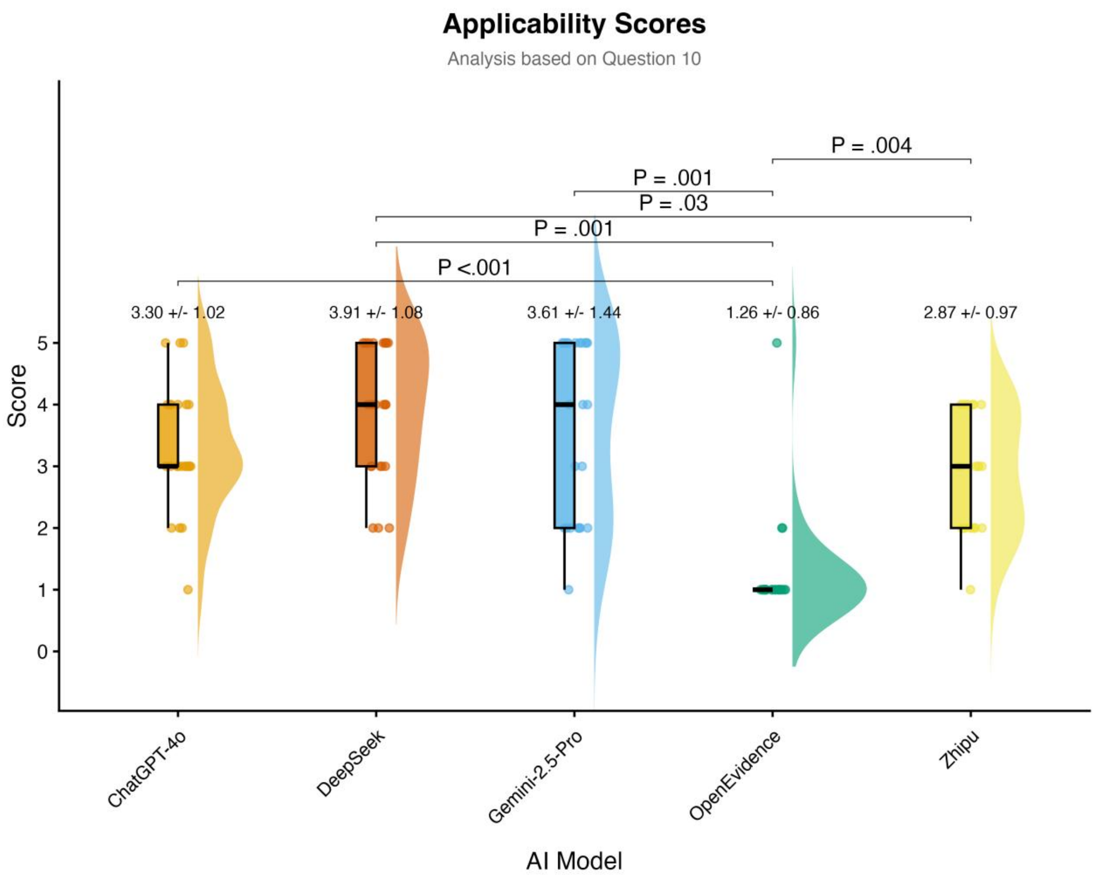
Applicability Scores
Analysis based on Question 10
P = .004
P = .001
P = .03
P = .001
P <.001
3.30 +/- 1.02
3.91 +/- 1.08
3.61 +/- 1.44
1.26 +/- 0.86
2.87 +/- 0.97
Score
5
4
3
2
1
0
ChatGPT-4o
DeepSeek
Gemini-2.5-Pro
OpenEvidence
Zhipu
AI Model

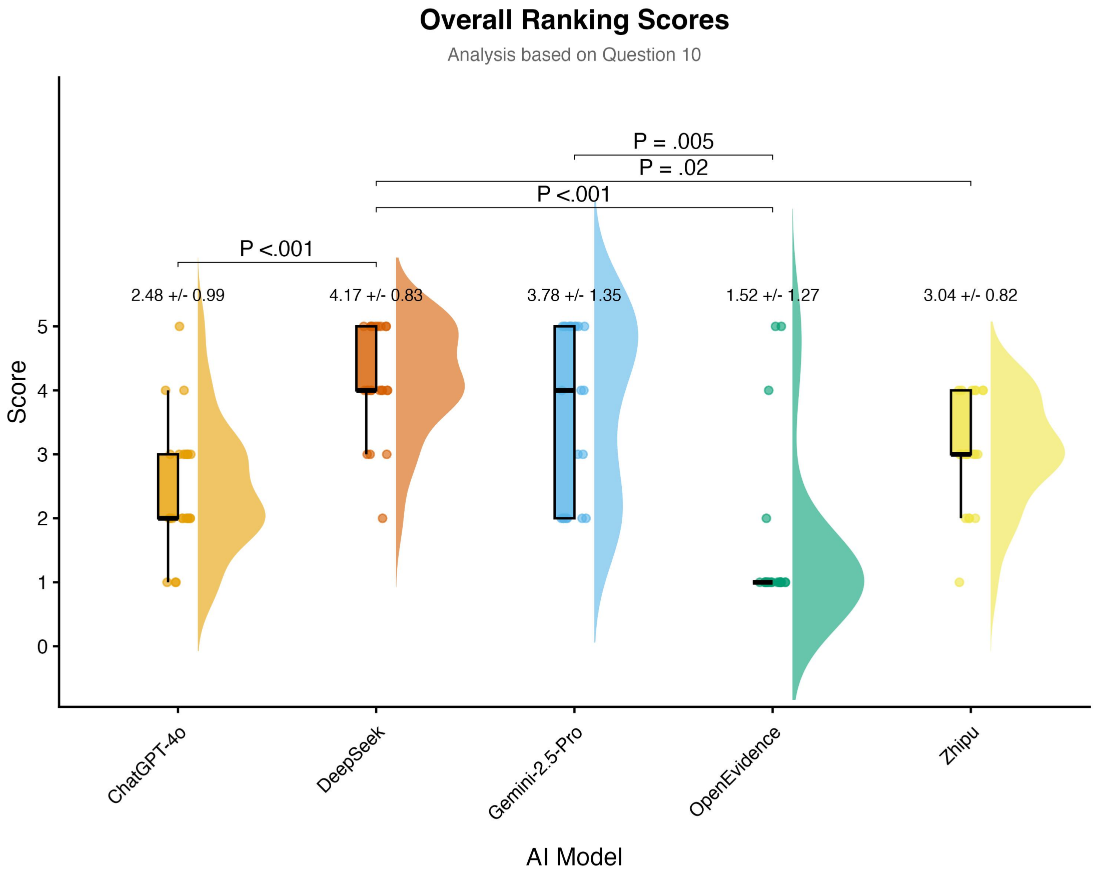
Overall Ranking Scores
Analysis based on Question 10
P = .005
P = .02
P <.001
P <.001
2.48 +/- 0.99
4.17 +/- 0.83
3.78 +/- 1.35
1.52 +/- 1.27
3.04 +/- 0.82
Score
5
4
3
2
1
0
ChatGPT-4o
DeepSeek
Gemini-2.5-Pro
OpenEvidence
Zhipu
AI Model
