# Supplementary material for: Performance of 5 Large Language Models in Perioperative Consultation for Pediatric Hypospadias: Cross-Sectional Comparative Study
Source: J Med Internet Res. 2026 Jul 29;28:e93393. doi: 10.2196/93393 (PMC13419283; doi:10.2196/93393)
Supplement: Multimedia Appendix 12 [file jmir-v28-e93393-s012.pdf]

Per-question caregiver ratings

This appendix consists of twenty figures depicting how the 36 primary caregivers rated each of the five large language models on each of the 10 perioperative questions: ten 100% stacked bar charts and ten violin (rain-cloud) plots. Panel structure and reading conventions are identical to Multimedia Appendix 8; the only differences are the evaluator population (caregivers, n = 36, instead of experts), the number of dimensions ranked (four caregiver dimensions — Empathy, Addressing Concerns, Comprehensibility, Actionability — instead of six expert dimensions), and the total evaluations per model per question (144 = 36 caregivers × 4 dimensions).

How to read the figures (identical to Multimedia Appendix 8).

Visual element	Meaning
Stacked bar chart (each question)	Each bar represents one of the five models; the bar is segmented by score (1–5) using the diverging Likert colour scheme; segment lengths show the proportion of evaluations falling at each score within that model. Total evaluations per model per question = 144 (36 caregivers × 4 dimensions).
Violin (rain-cloud) plot (each question)	The kernel-density cloud on the left, the jittered individual data points on the right, and the inner box (median + IQR) follow the same convention as Multimedia Appendix 8.
Score colour scheme (1–5)	5 = dark blue (Excellent); 4 = light blue (Good); 3 = pale yellow (Average); 2 = orange (Fair); 1 = red (Poor).
Friedman annotation	Top-of-panel "Friedman: χ^2(df) = X, P, W = Y".
Pairwise significance markers	Bonferroni-corrected pairwise Wilcoxon signed-rank comparisons are annotated with exact *P* values are annotated above pairwise comparison bars. *P* values omit leading zeros and are reported to two decimal places for *P* > .01, three decimal places for .001 ≤ *P* ≤ .01, and marked "<.001" for *P* < .001. All significance thresholds are kept to three decimal places to prevent rounding flip errors. Asterisk markers (//) and "ns" labels are removed; non-significant pairs are omitted to reduce visual clutter (corrected *α*' = .005).
Model labels on the x-axis	Unblinded model identities (ChatGPT-4o, DeepSeek, Gemini-2.5-Pro, OpenEvidence, Zhipu Qingyan); during evaluation the caregivers saw only the blinded AI1 – AI5 labels with a per-question randomised mapping.

Stacked Bar Charts: Per-Question caregiver Rating Distributions

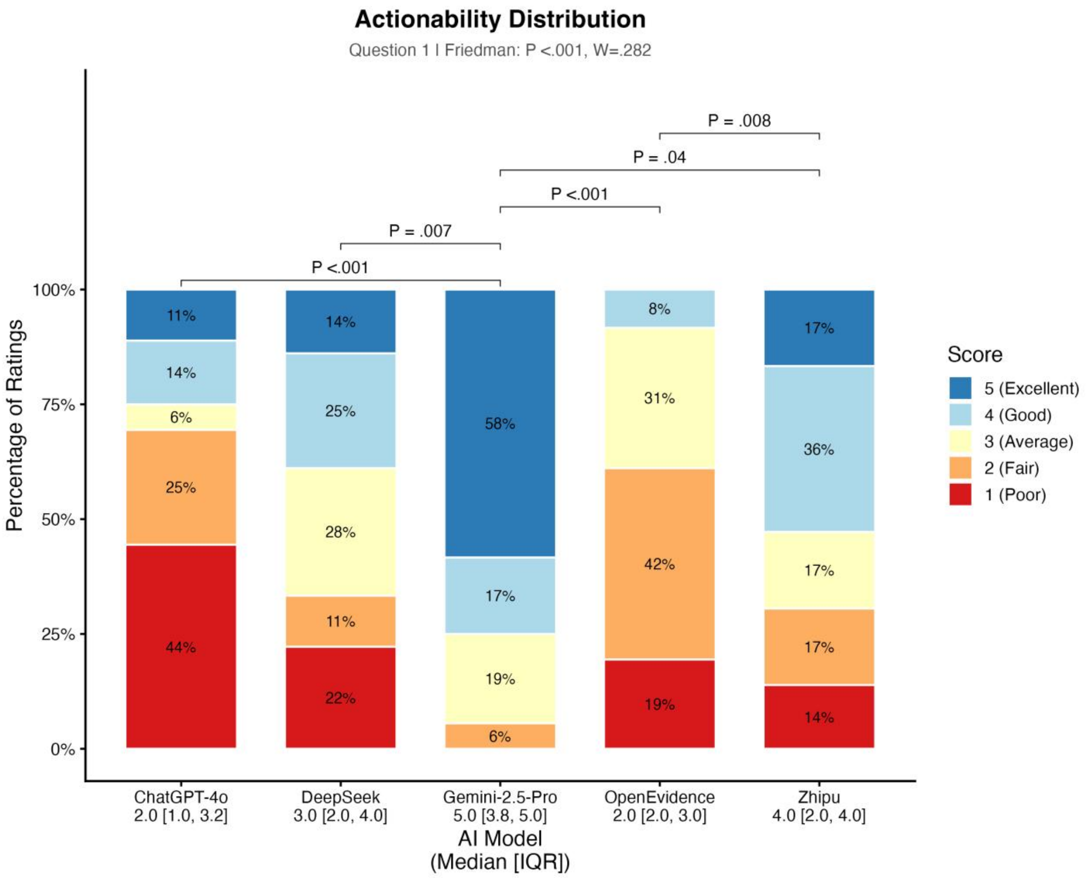

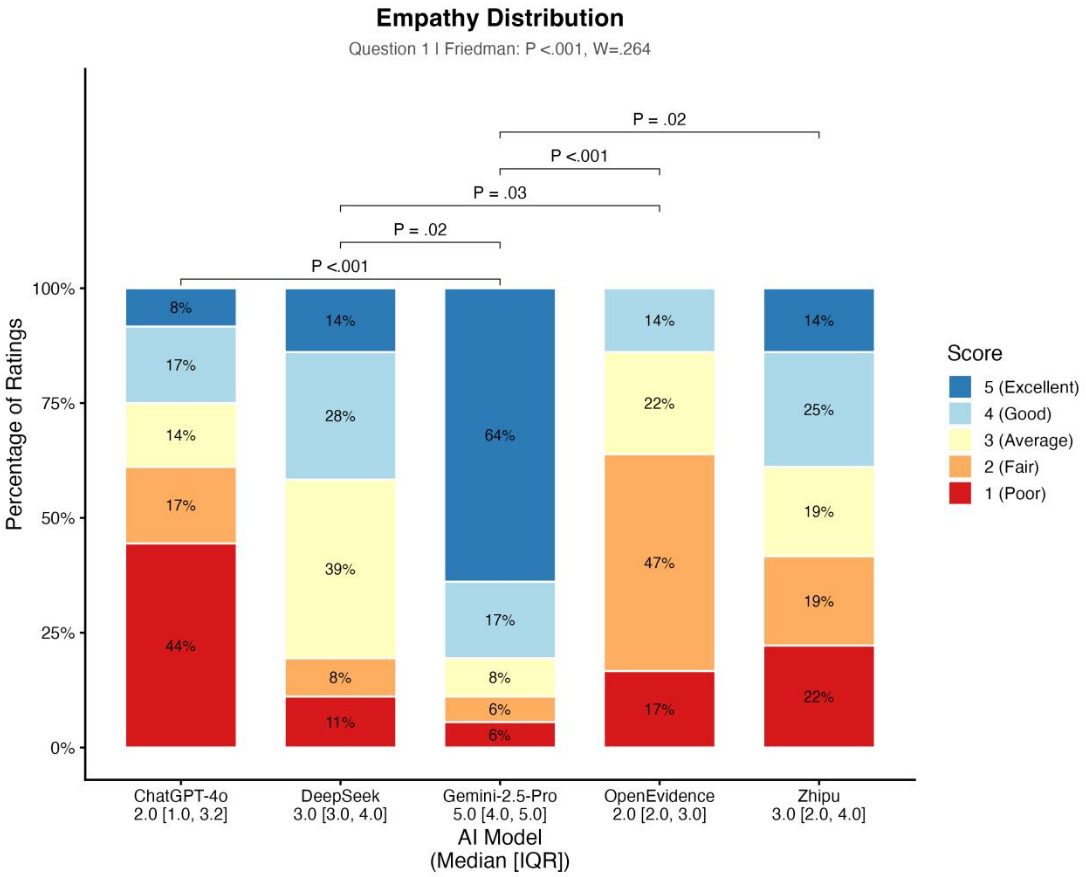

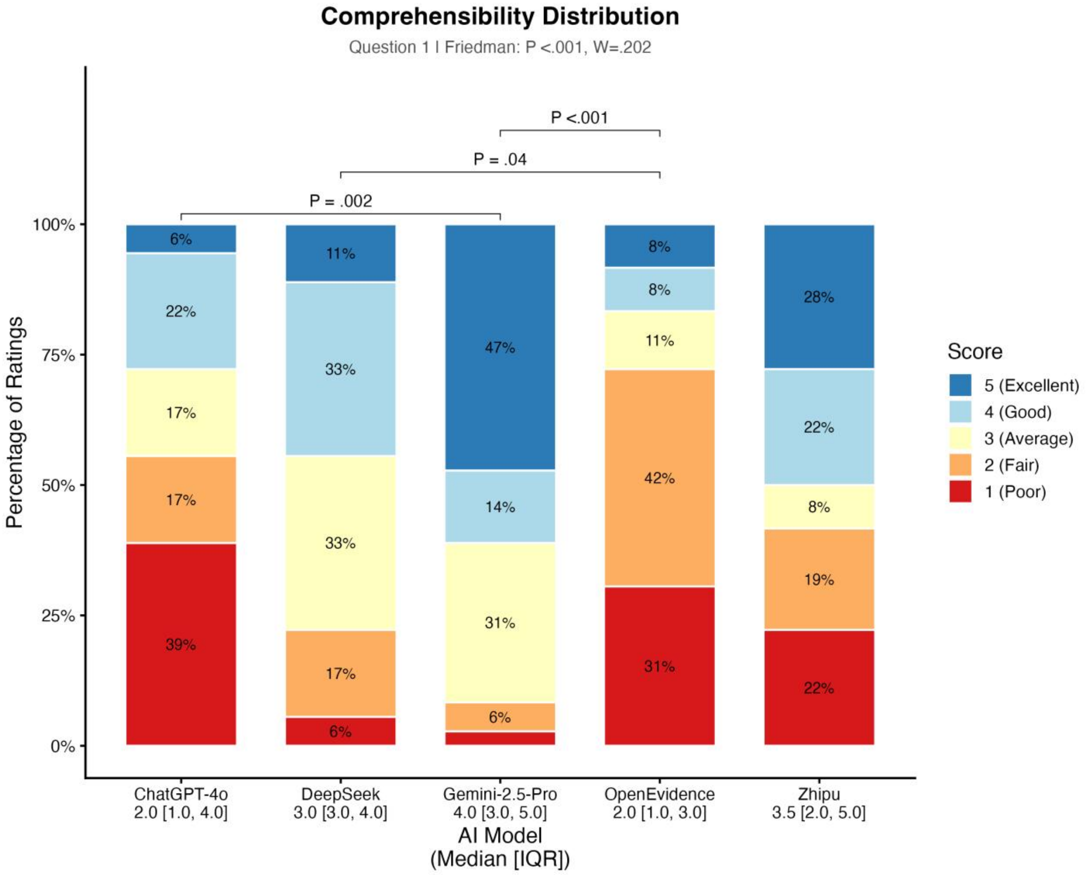
Comprehensibility Distribution
Question 1 | Friedman: P <.001, W=.202
P <.001
P = .04
P = .002
Percentage of Ratings
100%
75%
50%
25%
0%
6%
22%
17%
17%
39%
11%
33%
33%
17%
6%
47%
14%
31%
6%
8%
8%
11%
42%
31%
28%
22%
8%
19%
22%
ChatGPT-4o
2.0 [1.0, 4.0]
DeepSeek
3.0 [3.0, 4.0]
Gemini-2.5-Pro
4.0 [3.0, 5.0]
OpenEvidence
2.0 [1.0, 3.0]
Zhipu
3.5 [2.0, 5.0]
AI Model
(Median [IQR])
Score
5 (Excellent)
4 (Good)
3 (Average)
2 (Fair)
1 (Poor)

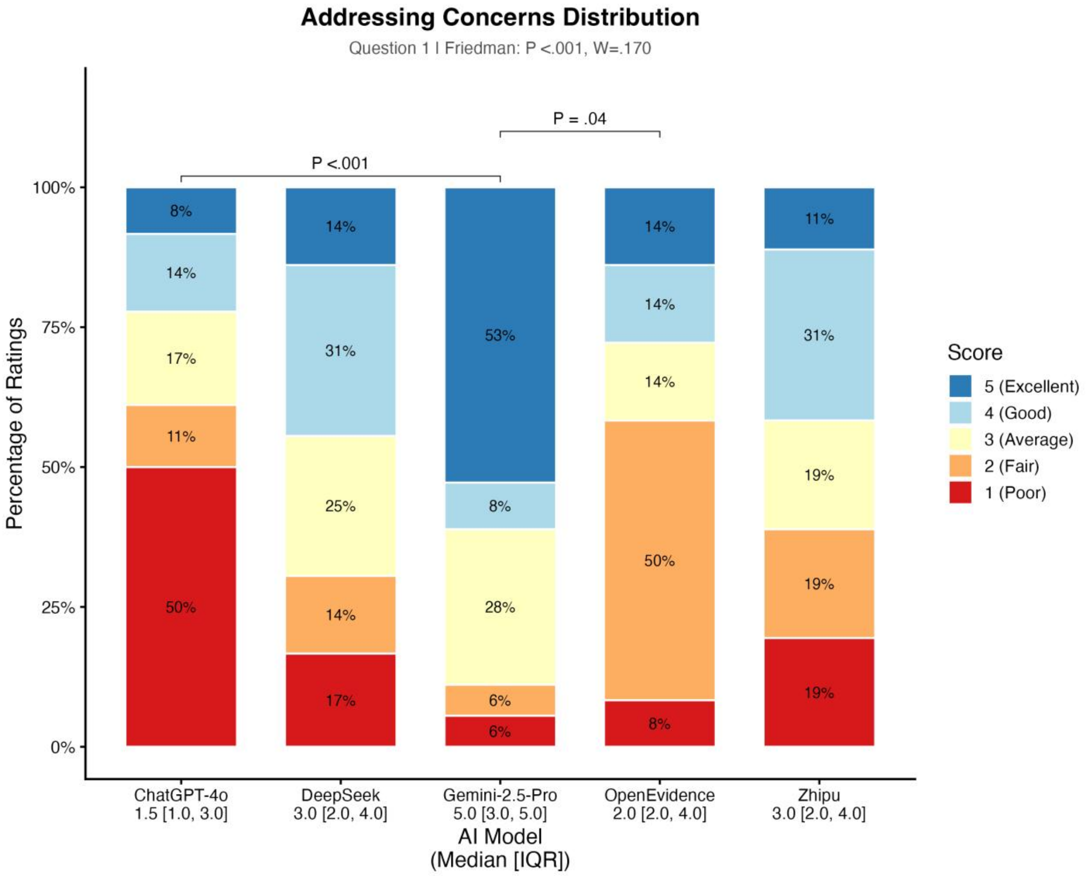
Addressing Concerns Distribution
Question 1 | Friedman: P <.001, W=.170
P = .04
P <.001
Percentage of Ratings
100%
75%
50%
25%
0%
8%
14%
17%
11%
50%
14%
31%
25%
14%
17%
53%
8%
28%
6%
6%
14%
14%
14%
50%
8%
11%
31%
19%
19%
19%
ChatGPT-4o
1.5 [1.0, 3.0]
DeepSeek
3.0 [2.0, 4.0]
Gemini-2.5-Pro
5.0 [3.0, 5.0]
OpenEvidence
2.0 [2.0, 4.0]
Zhipu
3.0 [2.0, 4.0]
AI Model
(Median [IQR])
Score
5 (Excellent)
4 (Good)
3 (Average)
2 (Fair)
1 (Poor)

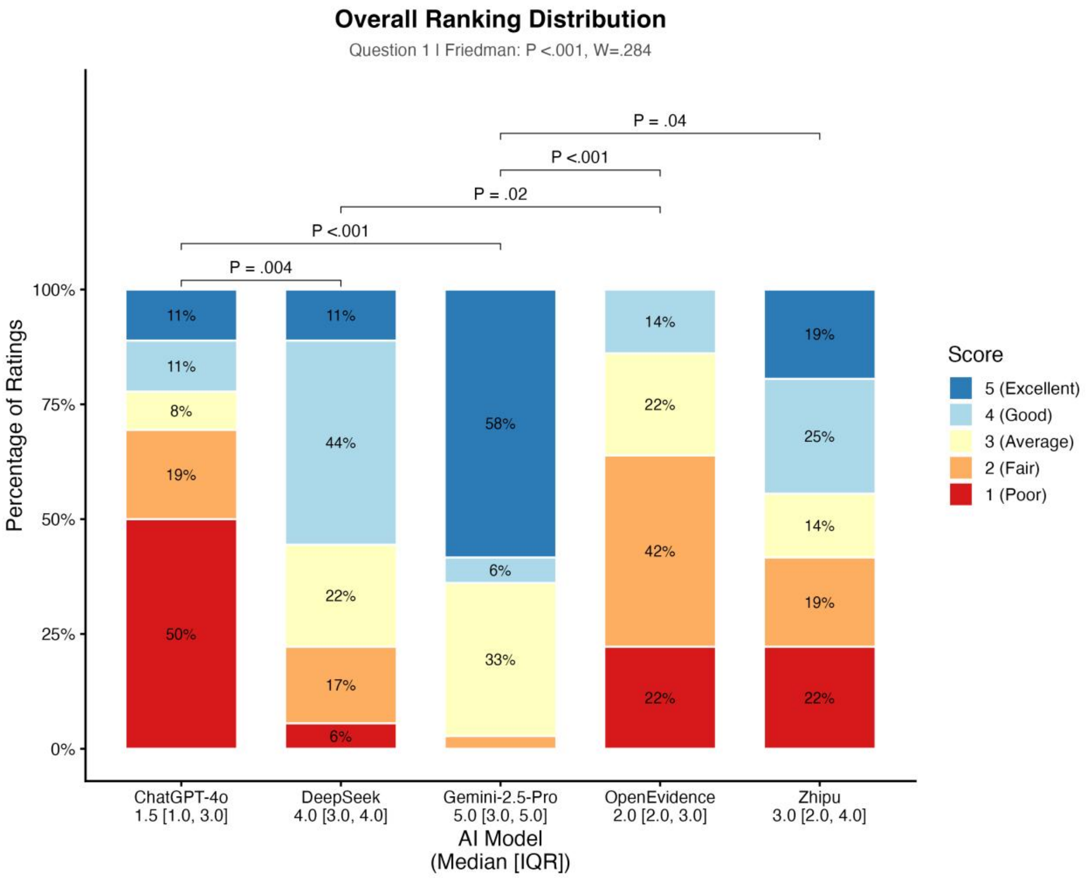
Overall Ranking Distribution
Question 1 | Friedman: P <.001, W=.284
P = .04
P <.001
P = .02
P <.001
P = .004
Percentage of Ratings
100%
75%
50%
25%
0%
11%
11%
8%
19%
50%
11%
44%
22%
17%
6%
58%
6%
33%
14%
22%
42%
22%
19%
25%
14%
19%
22%
ChatGPT-4o
1.5 [1.0, 3.0]
DeepSeek
4.0 [3.0, 4.0]
Gemini-2.5-Pro
5.0 [3.0, 5.0]
OpenEvidence
2.0 [2.0, 3.0]
Zhipu
3.0 [2.0, 4.0]
AI Model
(Median [IQR])
Score
5 (Excellent)
4 (Good)
3 (Average)
2 (Fair)
1 (Poor)

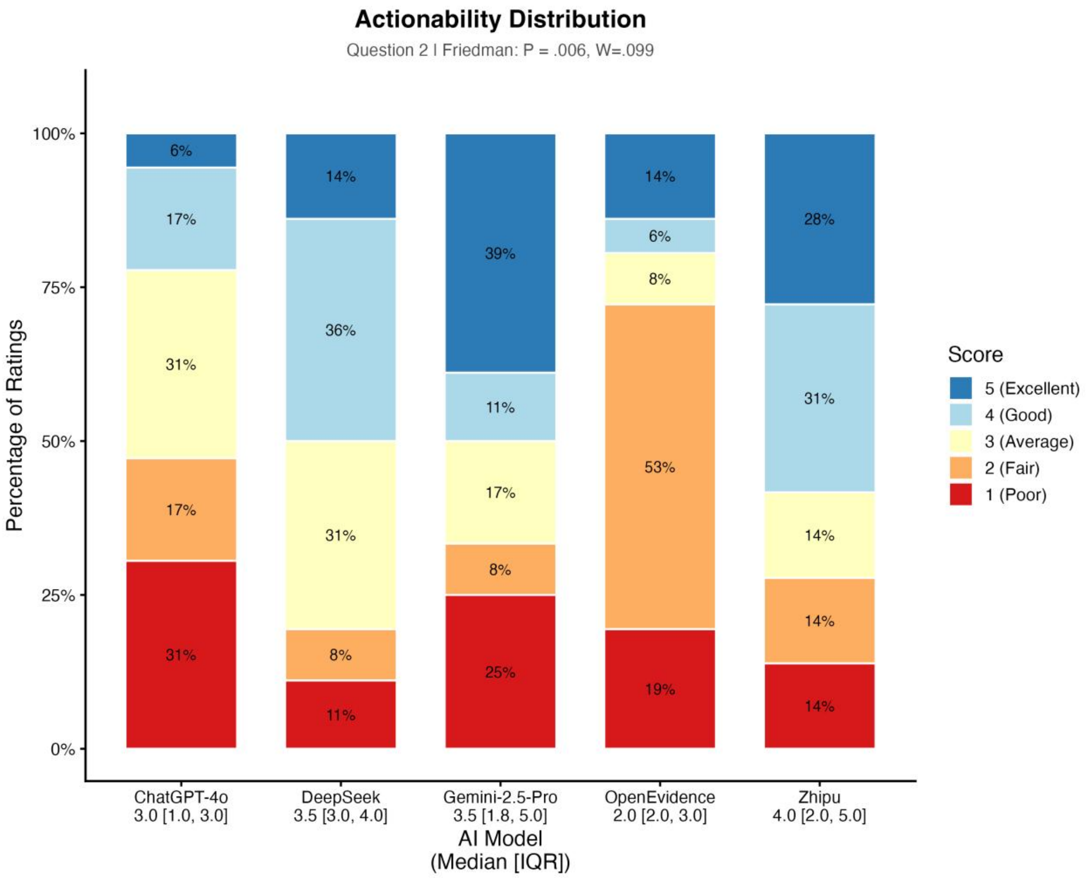
Actionability Distribution
Question 2 | Friedman: P = .006, W=.099
Percentage of Ratings
100%
75%
50%
25%
0%
6%
17%
31%
17%
31%
14%
36%
31%
8%
11%
39%
11%
17%
8%
25%
14%
6%
8%
53%
19%
28%
31%
14%
14%
14%
ChatGPT-4o
3.0 [1.0, 3.0]
DeepSeek
3.5 [3.0, 4.0]
Gemini-2.5-Pro
3.5 [1.8, 5.0]
OpenEvidence
2.0 [2.0, 3.0]
Zhipu
4.0 [2.0, 5.0]
AI Model
(Median [IQR])
Score
5 (Excellent)
4 (Good)
3 (Average)
2 (Fair)
1 (Poor)

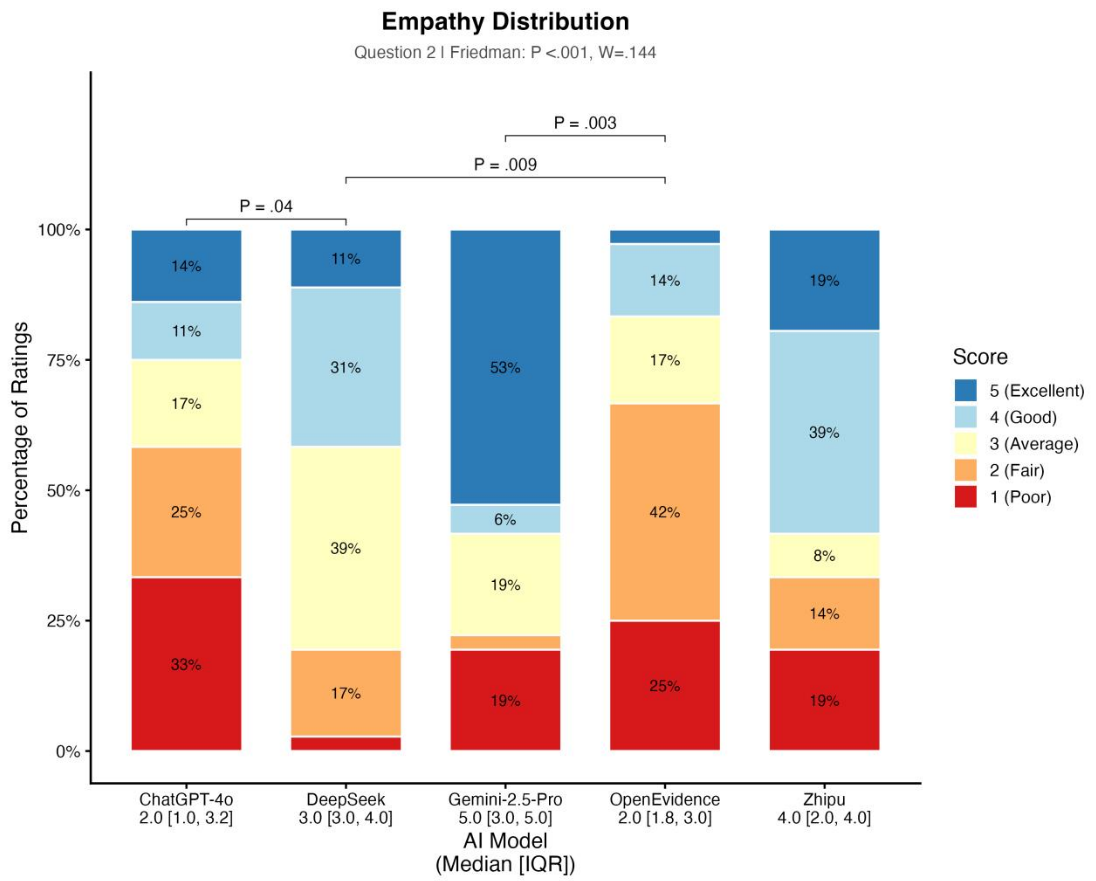

Empathy Distribution
Question 2 | Friedman: P <.001, W=.144
P = .003
P = .009
P = .04
100%
75%
50%
25%
0%
Percentage of Ratings
14%
11%
17%
25%
33%
11%
31%
39%
17%
53%
6%
19%
19%
14%
17%
42%
25%
19%
39%
8%
14%
19%
Score
5 (Excellent)
4 (Good)
3 (Average)
2 (Fair)
1 (Poor)
ChatGPT-4o
2.0 [1.0, 3.2]
DeepSeek
3.0 [3.0, 4.0]
Gemini-2.5-Pro
5.0 [3.0, 5.0]
OpenEvidence
2.0 [1.8, 3.0]
Zhipu
4.0 [2.0, 4.0]
AI Model
(Median [IQR])

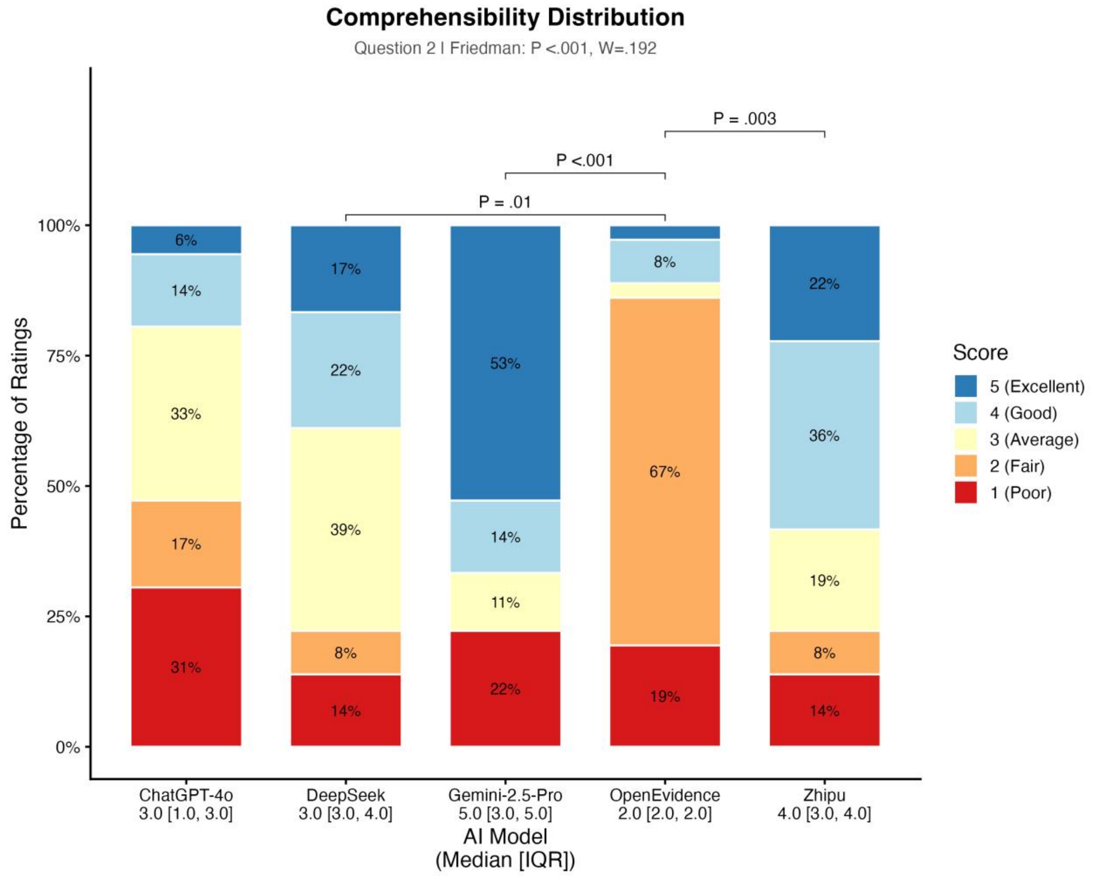

Comprehensibility Distribution
Question 2 | Friedman: P <.001, W=.192
P = .003
P <.001
P = .01
100%
75%
50%
25%
0%
Percentage of Ratings
6%
14%
33%
17%
31%
17%
22%
39%
8%
14%
53%
14%
11%
22%
8%
67%
19%
22%
36%
19%
8%
14%
Score
5 (Excellent)
4 (Good)
3 (Average)
2 (Fair)
1 (Poor)
ChatGPT-4o
3.0 [1.0, 3.0]
DeepSeek
3.0 [3.0, 4.0]
Gemini-2.5-Pro
5.0 [3.0, 5.0]
OpenEvidence
2.0 [2.0, 2.0]
Zhipu
4.0 [3.0, 4.0]
AI Model
(Median [IQR])

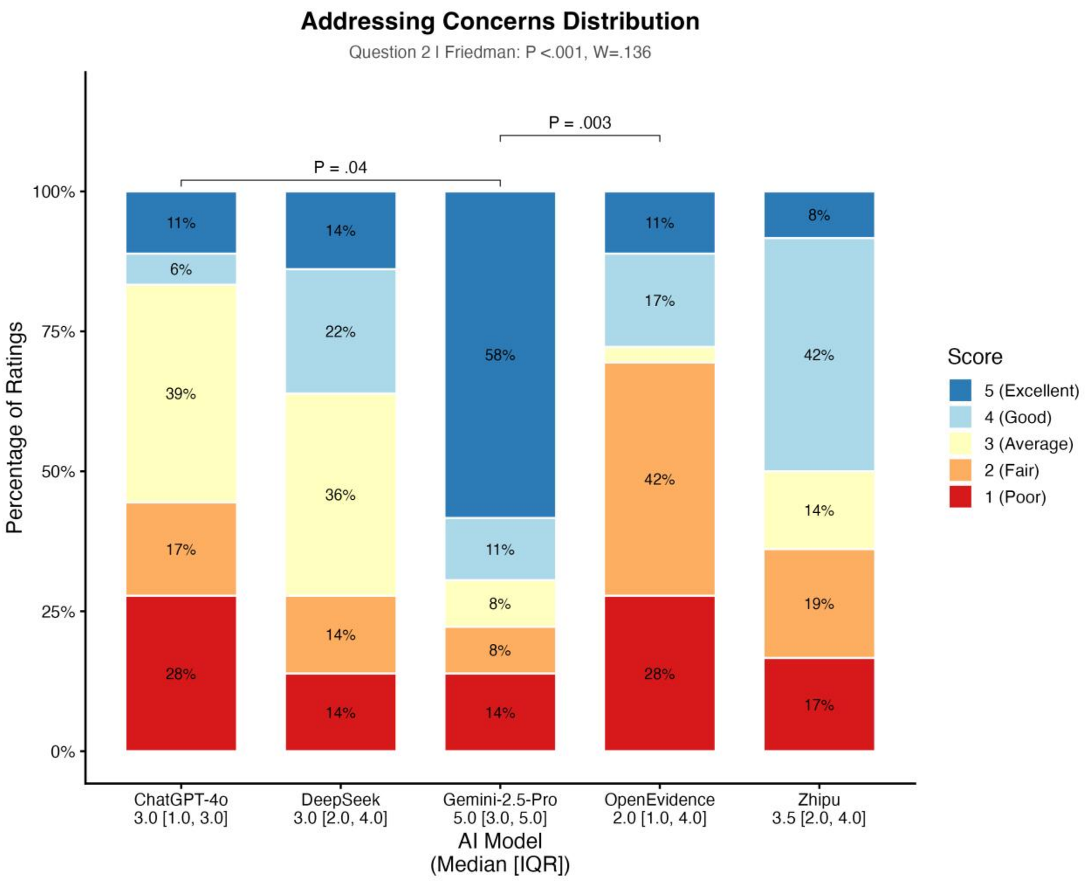
Addressing Concerns Distribution
Question 2 I Friedman: P <.001, W=.136
Percentage of Ratings
100%
75%
50%
25%
0%
P = .04
P = .003
11%
6%
39%
17%
28%
14%
22%
36%
14%
14%
58%
11%
8%
8%
14%
11%
17%
42%
28%
8%
42%
14%
19%
17%
ChatGPT-4o
3.0 [1.0, 3.0]
DeepSeek
3.0 [2.0, 4.0]
Gemini-2.5-Pro
5.0 [3.0, 5.0]
OpenEvidence
2.0 [1.0, 4.0]
Zhipu
3.5 [2.0, 4.0]
AI Model
(Median [IQR])
Score
5 (Excellent)
4 (Good)
3 (Average)
2 (Fair)
1 (Poor)

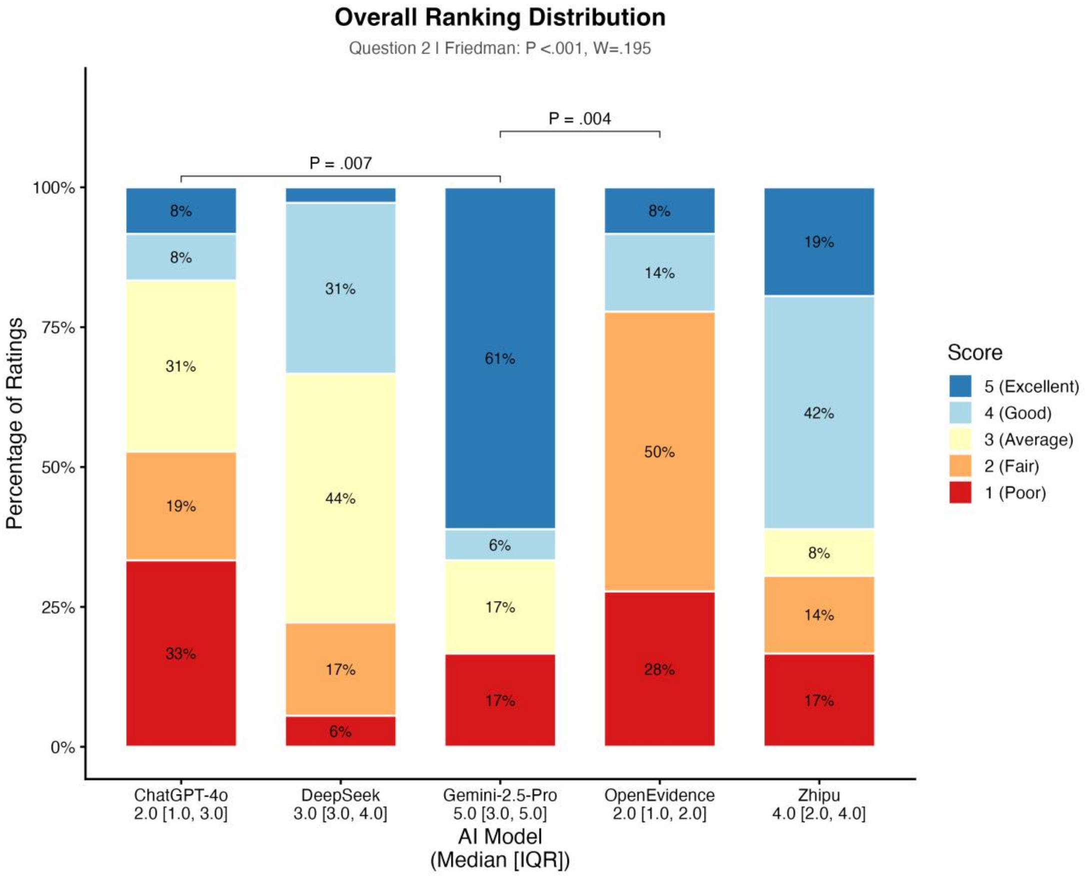
Overall Ranking Distribution
Question 2 I Friedman: P <.001, W=.195
Percentage of Ratings
100%
75%
50%
25%
0%
P = .007
P = .004
8%
8%
31%
19%
33%
31%
44%
17%
6%
61%
6%
17%
17%
8%
14%
50%
28%
19%
42%
8%
14%
17%
ChatGPT-4o
2.0 [1.0, 3.0]
DeepSeek
3.0 [3.0, 4.0]
Gemini-2.5-Pro
5.0 [3.0, 5.0]
OpenEvidence
2.0 [1.0, 2.0]
Zhipu
4.0 [2.0, 4.0]
AI Model
(Median [IQR])
Score
5 (Excellent)
4 (Good)
3 (Average)
2 (Fair)
1 (Poor)

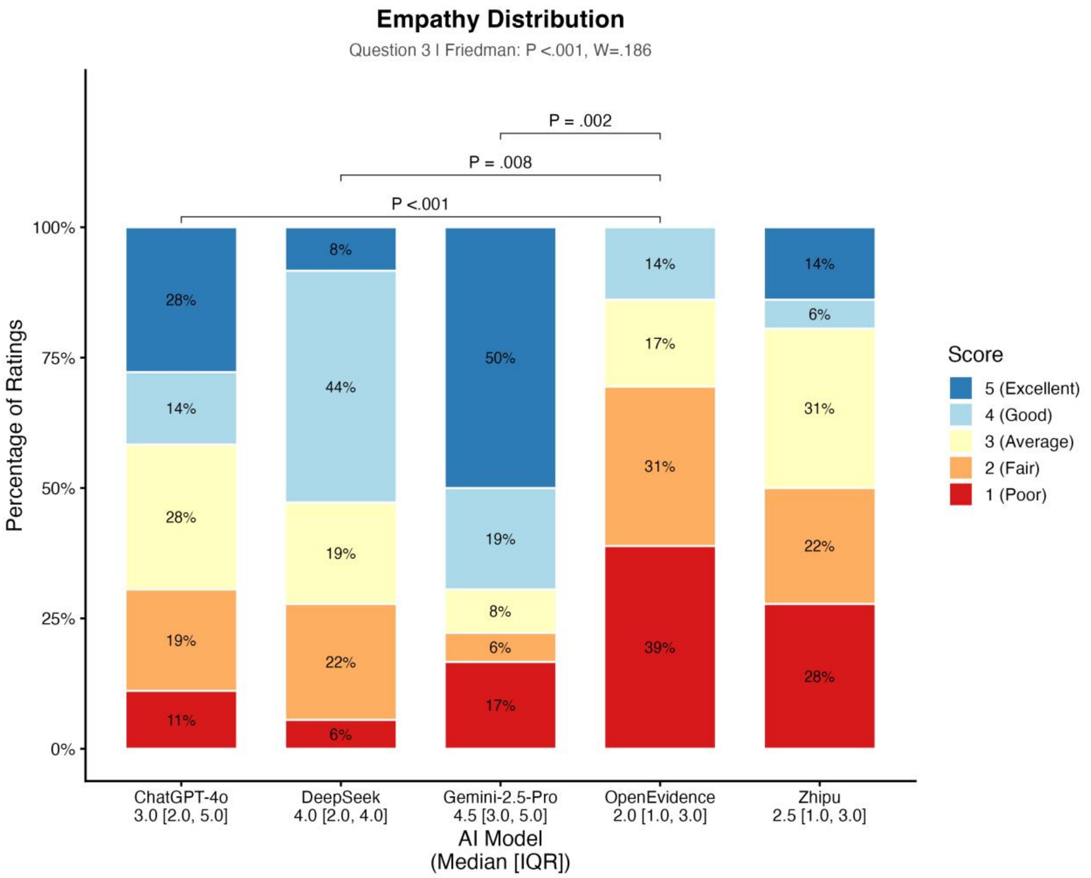
Empathy Distribution
Question 3 | Friedman: P <.001, W=.186
P = .002
P = .008
P <.001
Percentage of Ratings
100%
75%
50%
25%
0%
28%
14%
28%
19%
11%
8%
44%
19%
22%
6%
50%
19%
8%
6%
17%
14%
17%
31%
39%
14%
6%
31%
22%
28%
Score
5 (Excellent)
4 (Good)
3 (Average)
2 (Fair)
1 (Poor)
ChatGPT-4o
3.0 [2.0, 5.0]
DeepSeek
4.0 [2.0, 4.0]
Gemini-2.5-Pro
4.5 [3.0, 5.0]
OpenEvidence
2.0 [1.0, 3.0]
Zhipu
2.5 [1.0, 3.0]
AI Model
(Median [IQR])

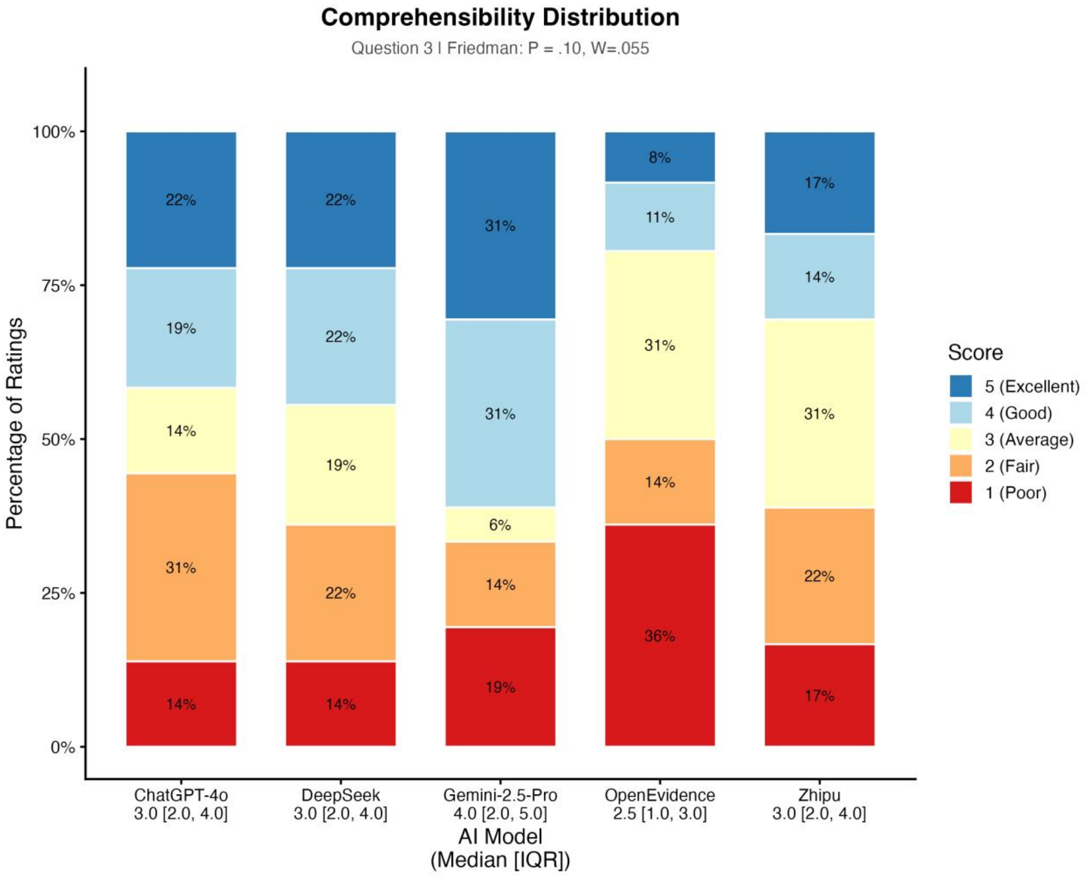
Comprehensibility Distribution
Question 3 | Friedman: P = .10, W=.055
Percentage of Ratings
100%
75%
50%
25%
0%
22%
19%
14%
31%
14%
22%
22%
19%
22%
14%
31%
31%
6%
14%
19%
8%
11%
31%
14%
36%
17%
14%
31%
22%
17%
Score
5 (Excellent)
4 (Good)
3 (Average)
2 (Fair)
1 (Poor)
ChatGPT-4o
3.0 [2.0, 4.0]
DeepSeek
3.0 [2.0, 4.0]
Gemini-2.5-Pro
4.0 [2.0, 5.0]
OpenEvidence
2.5 [1.0, 3.0]
Zhipu
3.0 [2.0, 4.0]
AI Model
(Median [IQR])

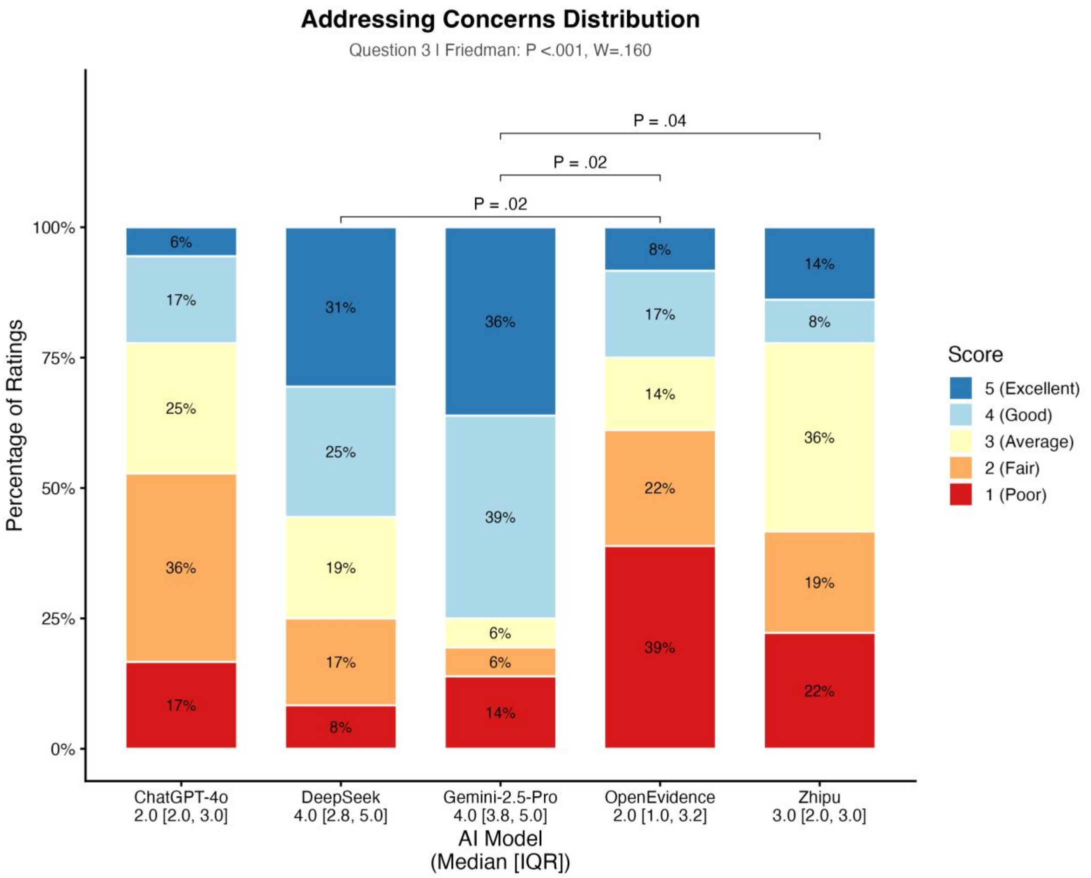
Addressing Concerns Distribution
Question 3 | Friedman: P <.001, W=.160
P = .04
P = .02
P = .02
Percentage of Ratings
100%
75%
50%
25%
0%
6%
17%
25%
36%
17%
31%
25%
19%
17%
8%
36%
39%
6%
6%
14%
8%
17%
14%
22%
39%
14%
8%
36%
19%
22%
Score
5 (Excellent)
4 (Good)
3 (Average)
2 (Fair)
1 (Poor)
ChatGPT-4o
2.0 [2.0, 3.0]
DeepSeek
4.0 [2.8, 5.0]
Gemini-2.5-Pro
4.0 [3.8, 5.0]
OpenEvidence
2.0 [1.0, 3.2]
Zhipu
3.0 [2.0, 3.0]
AI Model
(Median [IQR])

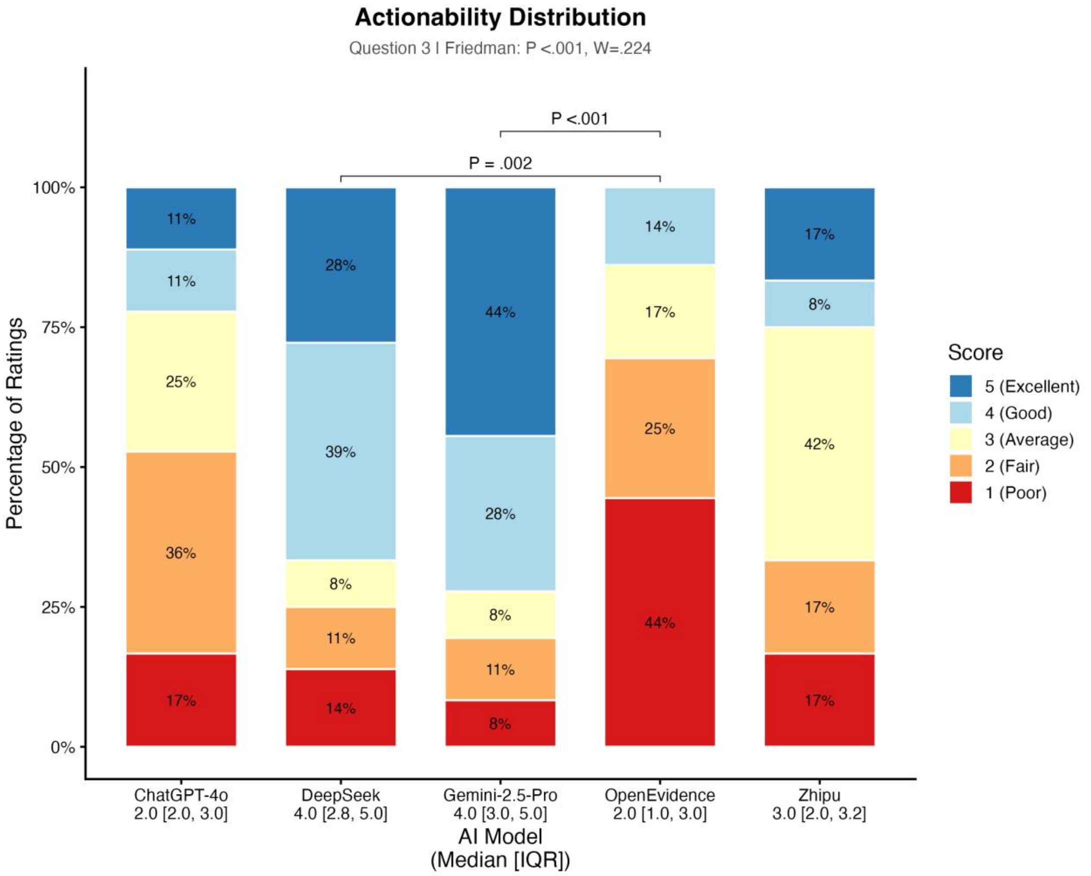
Actionability Distribution
Question 3 | Friedman: P <.001, W=.224
P <.001
P = .002
Percentage of Ratings
100%
75%
50%
25%
0%
11%
11%
25%
36%
17%
28%
39%
8%
11%
14%
44%
28%
8%
11%
8%
14%
17%
25%
44%
17%
8%
42%
17%
17%
Score
5 (Excellent)
4 (Good)
3 (Average)
2 (Fair)
1 (Poor)
ChatGPT-4o
2.0 [2.0, 3.0]
DeepSeek
4.0 [2.8, 5.0]
Gemini-2.5-Pro
4.0 [3.0, 5.0]
OpenEvidence
2.0 [1.0, 3.0]
Zhipu
3.0 [2.0, 3.2]
AI Model
(Median [IQR])

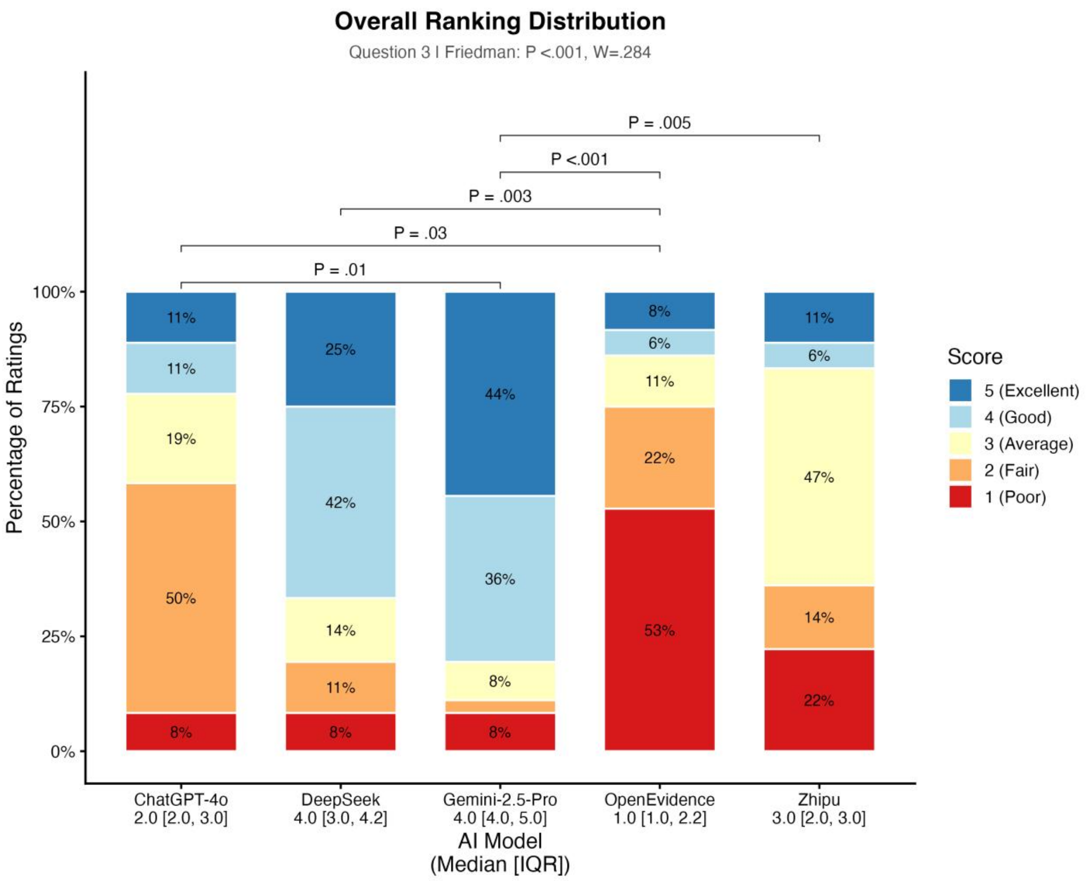

Overall Ranking Distribution
Question 3 I Friedman: P <.001, W=.284
P = .005
P <.001
P = .003
P = .03
P = .01
100%
75%
50%
25%
0%
Percentage of Ratings
11%
11%
19%
50%
8%
25%
42%
14%
11%
8%
44%
36%
8%
8%
8%
6%
11%
22%
53%
11%
6%
47%
14%
22%
ChatGPT-4o
2.0 [2.0, 3.0]
DeepSeek
4.0 [3.0, 4.2]
Gemini-2.5-Pro
4.0 [4.0, 5.0]
OpenEvidence
1.0 [1.0, 2.2]
Zhipu
3.0 [2.0, 3.0]
AI Model
(Median [IQR])
Score
5 (Excellent)
4 (Good)
3 (Average)
2 (Fair)
1 (Poor)

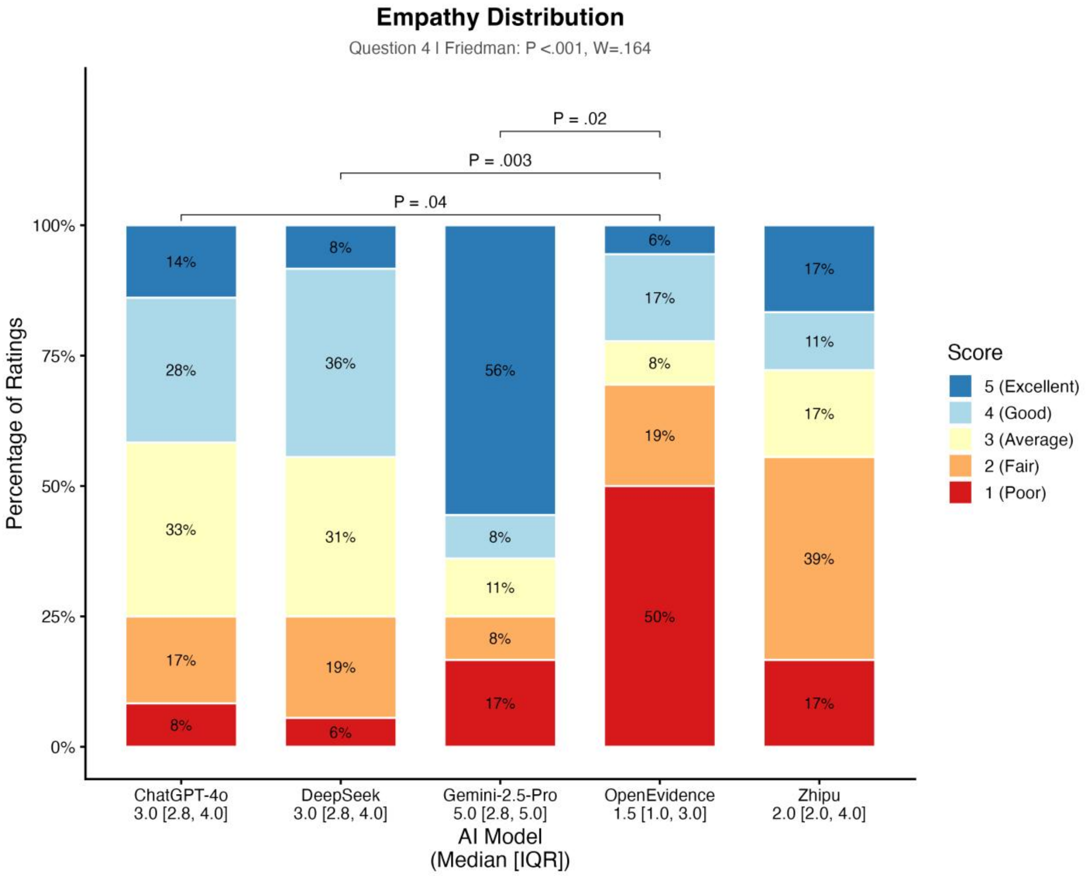

Empathy Distribution
Question 4 I Friedman: P <.001, W=.164
P = .02
P = .003
P = .04
100%
75%
50%
25%
0%
Percentage of Ratings
14%
28%
33%
17%
8%
8%
36%
31%
19%
6%
56%
8%
11%
8%
17%
6%
17%
8%
19%
50%
17%
11%
17%
39%
17%
ChatGPT-4o
3.0 [2.8, 4.0]
DeepSeek
3.0 [2.8, 4.0]
Gemini-2.5-Pro
5.0 [2.8, 5.0]
OpenEvidence
1.5 [1.0, 3.0]
Zhipu
2.0 [2.0, 4.0]
AI Model
(Median [IQR])
Score
5 (Excellent)
4 (Good)
3 (Average)
2 (Fair)
1 (Poor)

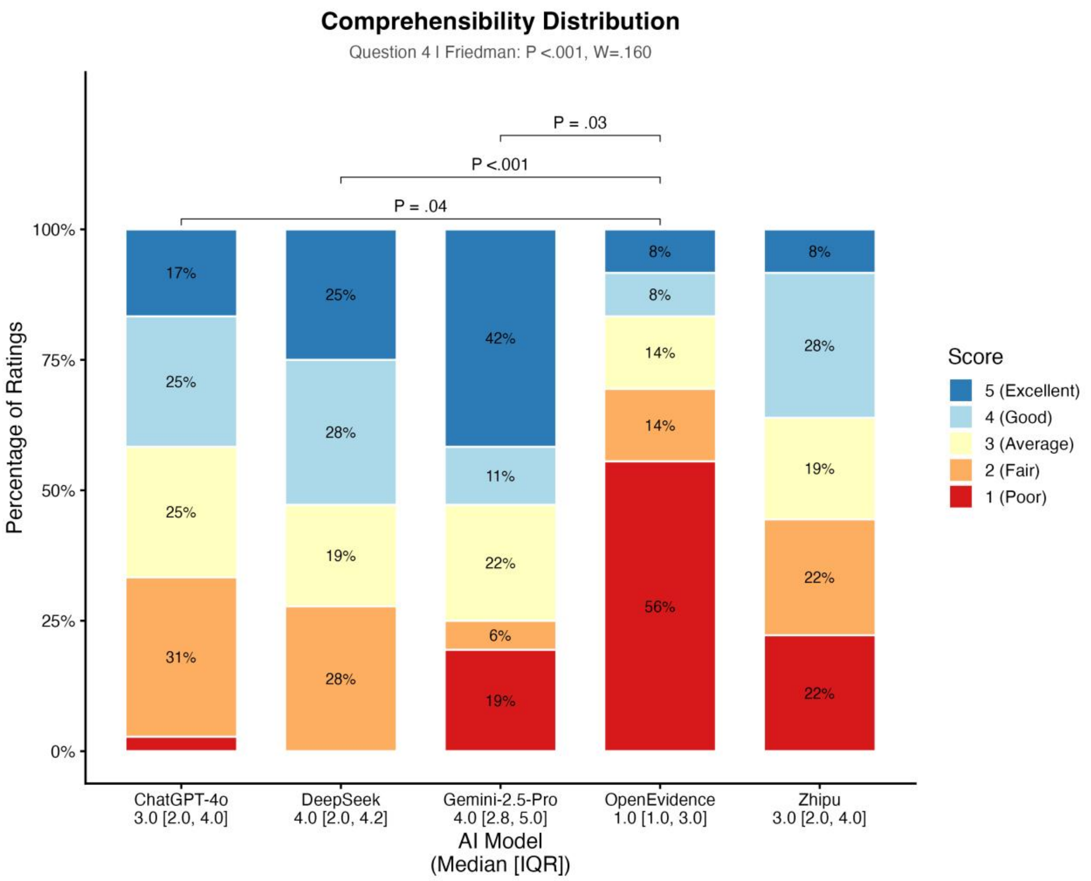
Comprehensibility Distribution
Question 4 | Friedman: P <.001, W=.160
P = .03
P <.001
P = .04
Percentage of Ratings
100%
75%
50%
25%
0%
17%
25%
25%
31%
25%
28%
19%
28%
42%
11%
22%
6%
19%
8%
8%
14%
14%
56%
8%
28%
19%
22%
22%
ChatGPT-4o
3.0 [2.0, 4.0]
DeepSeek
4.0 [2.0, 4.2]
Gemini-2.5-Pro
4.0 [2.8, 5.0]
OpenEvidence
1.0 [1.0, 3.0]
Zhipu
3.0 [2.0, 4.0]
AI Model
(Median [IQR])
Score
5 (Excellent)
4 (Good)
3 (Average)
2 (Fair)
1 (Poor)

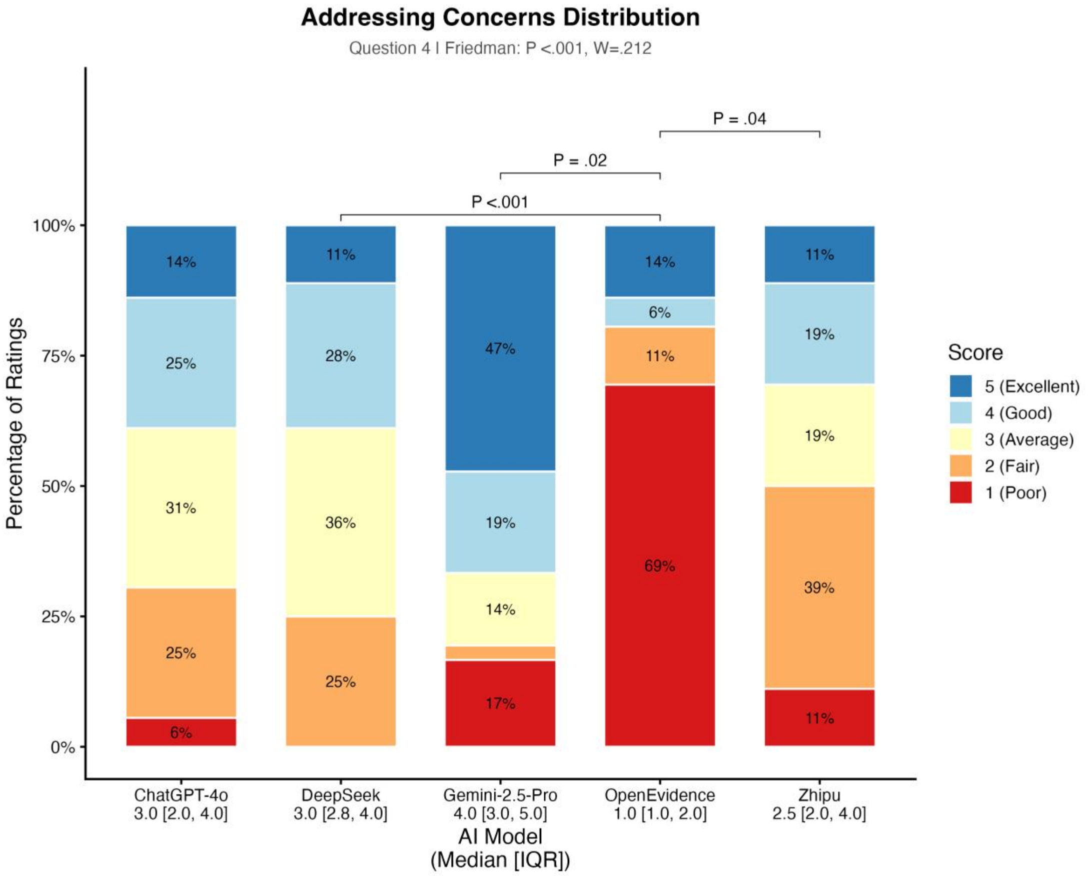
Addressing Concerns Distribution
Question 4 | Friedman: P <.001, W=.212
P = .04
P = .02
P <.001
Percentage of Ratings
100%
75%
50%
25%
0%
14%
25%
31%
25%
6%
11%
28%
36%
25%
47%
19%
14%
17%
14%
6%
11%
69%
11%
19%
19%
39%
11%
ChatGPT-4o
3.0 [2.0, 4.0]
DeepSeek
3.0 [2.8, 4.0]
Gemini-2.5-Pro
4.0 [3.0, 5.0]
OpenEvidence
1.0 [1.0, 2.0]
Zhipu
2.5 [2.0, 4.0]
AI Model
(Median [IQR])
Score
5 (Excellent)
4 (Good)
3 (Average)
2 (Fair)
1 (Poor)

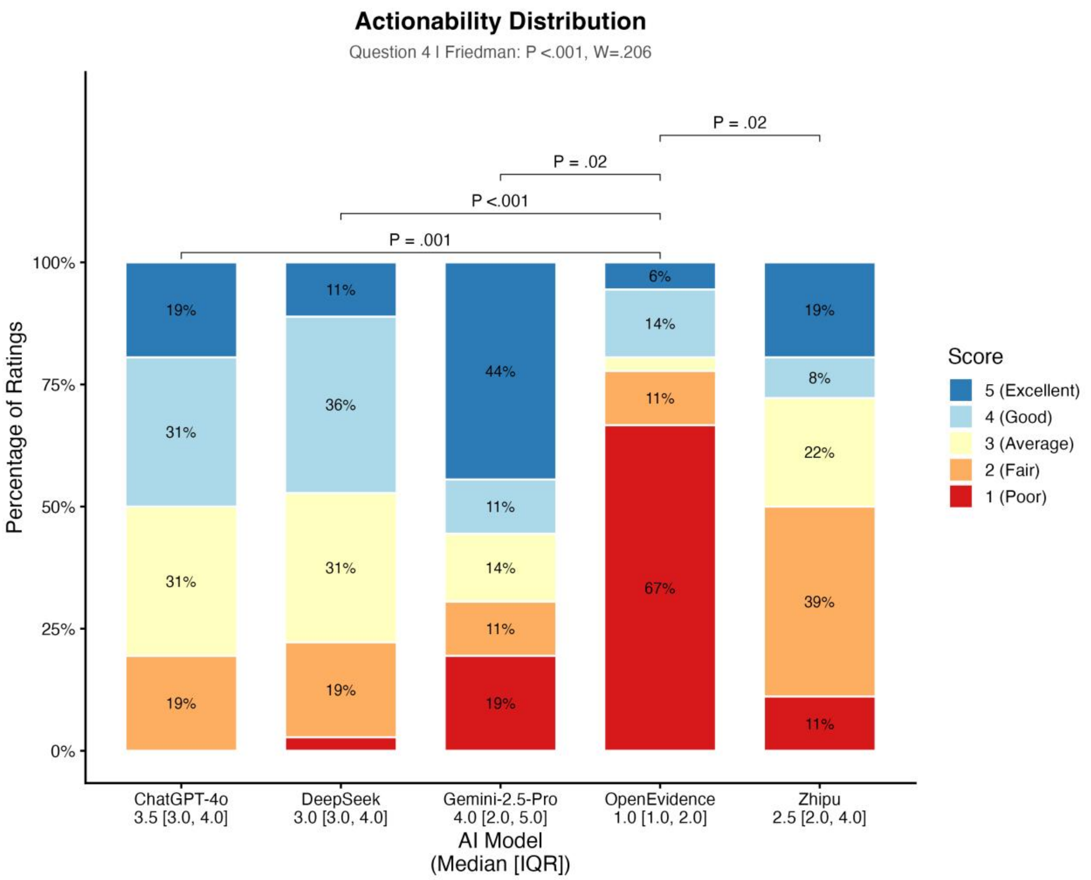
Actionability Distribution
Question 4 | Friedman: P <.001, W=.206
P = .02
P = .02
P <.001
P = .001
Percentage of Ratings
100%
75%
50%
25%
0%
19%
31%
31%
19%
11%
36%
31%
19%
44%
11%
14%
11%
19%
6%
14%
11%
67%
19%
8%
22%
39%
11%
Score
5 (Excellent)
4 (Good)
3 (Average)
2 (Fair)
1 (Poor)
ChatGPT-4o
3.5 [3.0, 4.0]
DeepSeek
3.0 [3.0, 4.0]
Gemini-2.5-Pro
4.0 [2.0, 5.0]
OpenEvidence
1.0 [1.0, 2.0]
Zhipu
2.5 [2.0, 4.0]
AI Model
(Median [IQR])

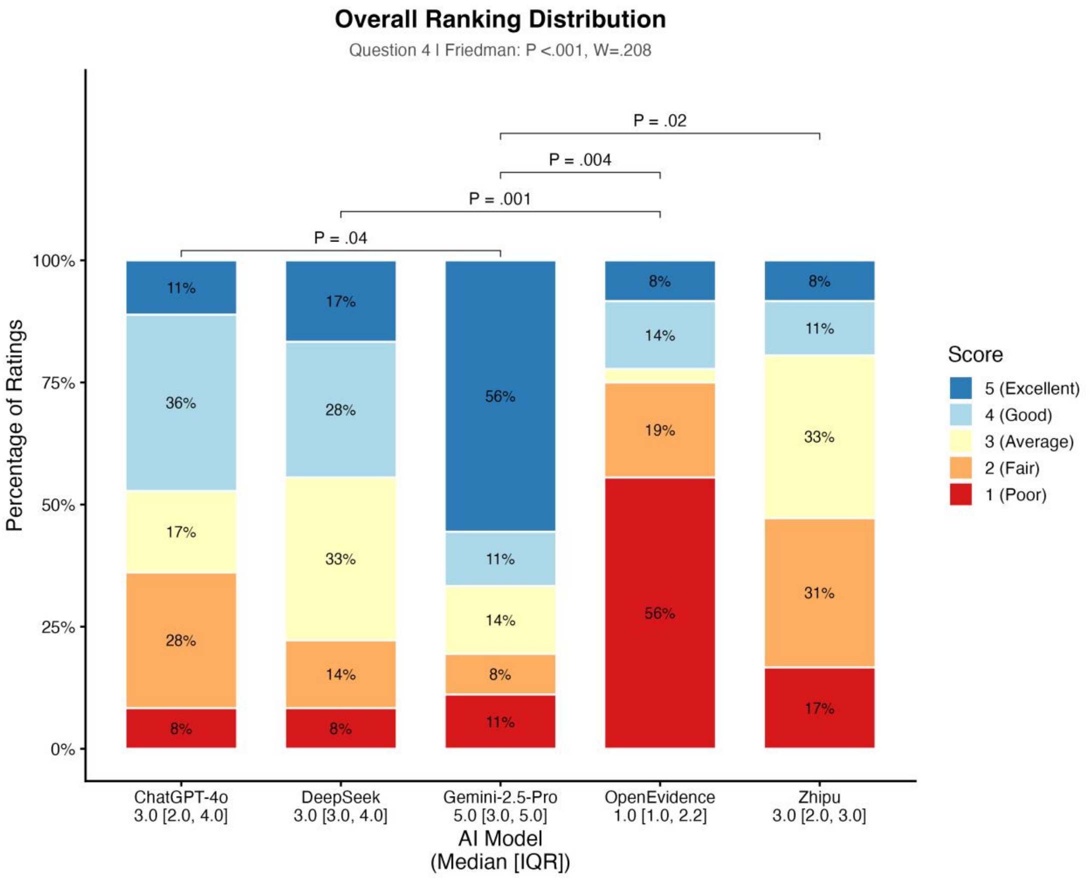
Overall Ranking Distribution
Question 4 | Friedman: P <.001, W=.208
P = .02
P = .004
P = .001
P = .04
Percentage of Ratings
100%
75%
50%
25%
0%
11%
36%
17%
28%
8%
17%
28%
33%
14%
8%
56%
11%
14%
8%
11%
8%
14%
19%
56%
8%
11%
33%
31%
17%
Score
5 (Excellent)
4 (Good)
3 (Average)
2 (Fair)
1 (Poor)
ChatGPT-4o
3.0 [2.0, 4.0]
DeepSeek
3.0 [3.0, 4.0]
Gemini-2.5-Pro
5.0 [3.0, 5.0]
OpenEvidence
1.0 [1.0, 2.2]
Zhipu
3.0 [2.0, 3.0]
AI Model
(Median [IQR])

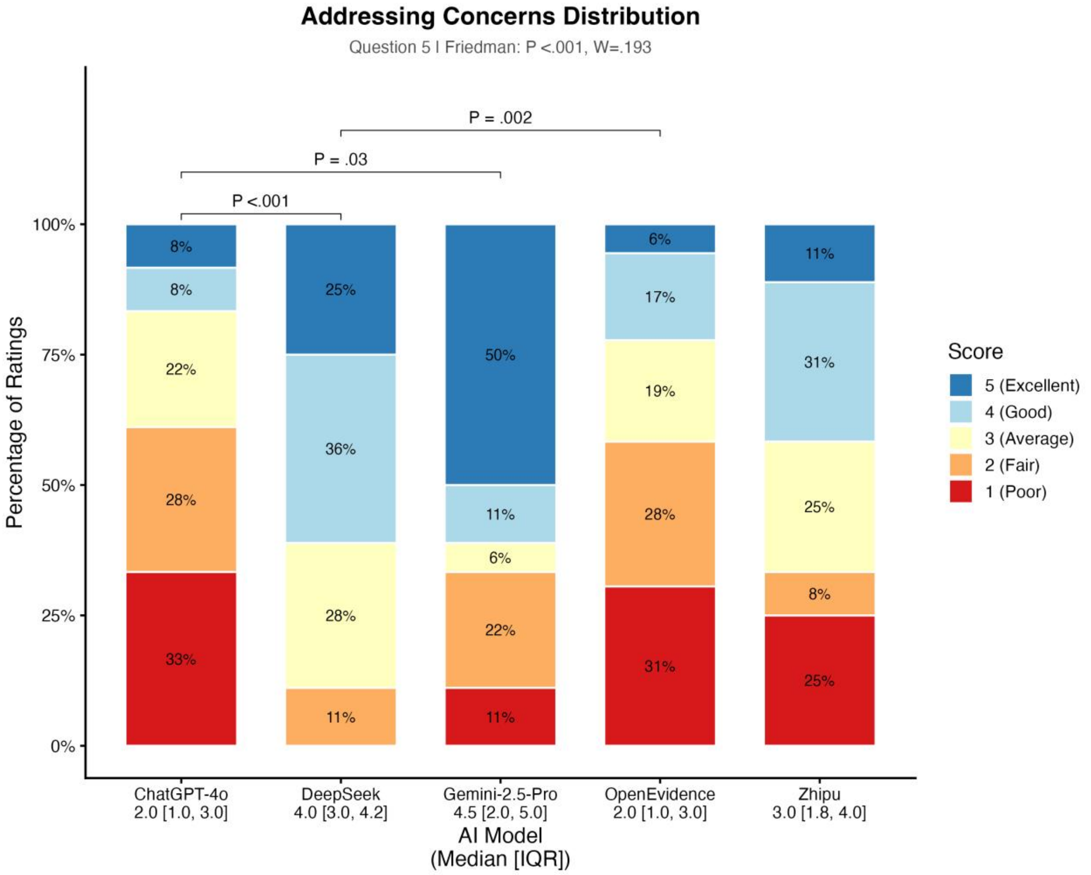
Addressing Concerns Distribution
Question 5 | Friedman: P <.001, W=.193
P = .002
P = .03
P <.001
Percentage of Ratings
100%
75%
50%
25%
0%
8%
8%
22%
28%
33%
25%
36%
28%
11%
50%
11%
6%
22%
11%
6%
17%
19%
28%
31%
11%
31%
25%
8%
25%
ChatGPT-4o
2.0 [1.0, 3.0]
DeepSeek
4.0 [3.0, 4.2]
Gemini-2.5-Pro
4.5 [2.0, 5.0]
OpenEvidence
2.0 [1.0, 3.0]
Zhipu
3.0 [1.8, 4.0]
AI Model
(Median [IQR])
Score
5 (Excellent)
4 (Good)
3 (Average)
2 (Fair)
1 (Poor)

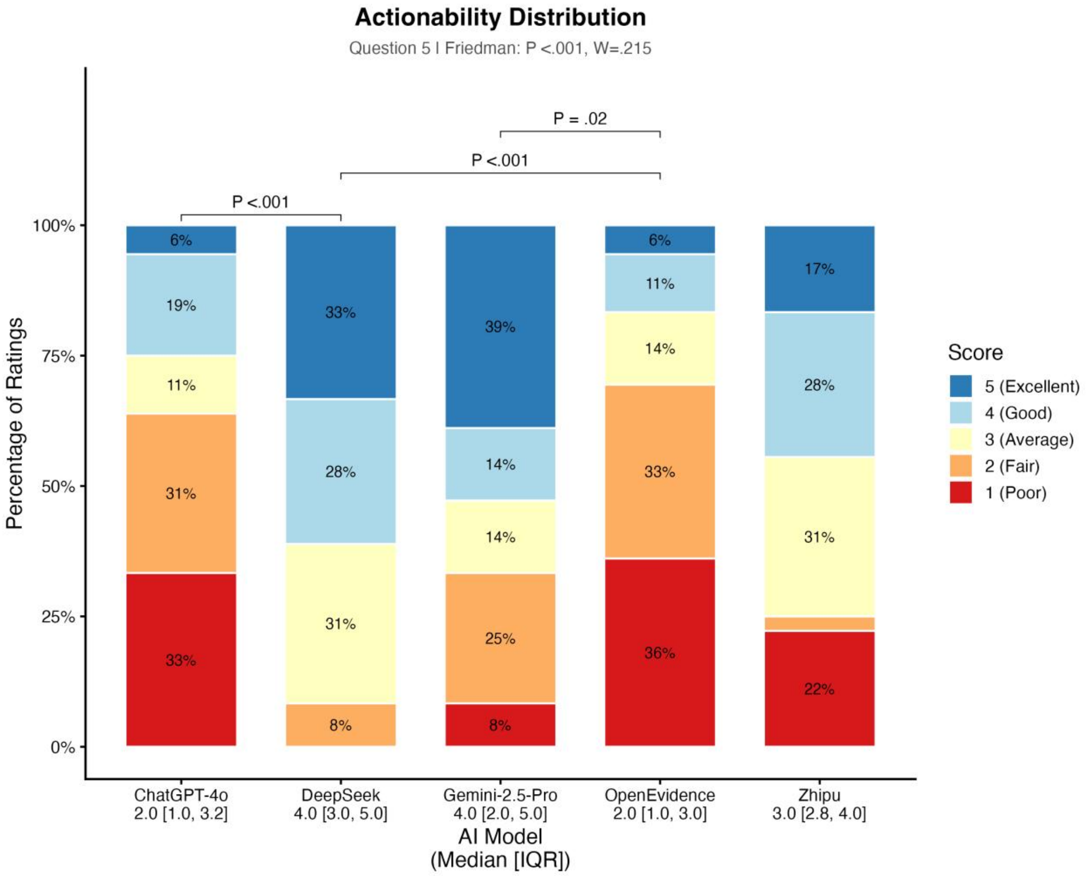
Actionability Distribution
Question 5 | Friedman: P <.001, W=.215
P = .02
P <.001
P <.001
Percentage of Ratings
100%
75%
50%
25%
0%
6%
19%
11%
31%
33%
33%
28%
31%
8%
39%
14%
14%
25%
8%
6%
11%
14%
33%
36%
17%
28%
31%
22%
ChatGPT-4o
2.0 [1.0, 3.2]
DeepSeek
4.0 [3.0, 5.0]
Gemini-2.5-Pro
4.0 [2.0, 5.0]
OpenEvidence
2.0 [1.0, 3.0]
Zhipu
3.0 [2.8, 4.0]
AI Model
(Median [IQR])
Score
5 (Excellent)
4 (Good)
3 (Average)
2 (Fair)
1 (Poor)

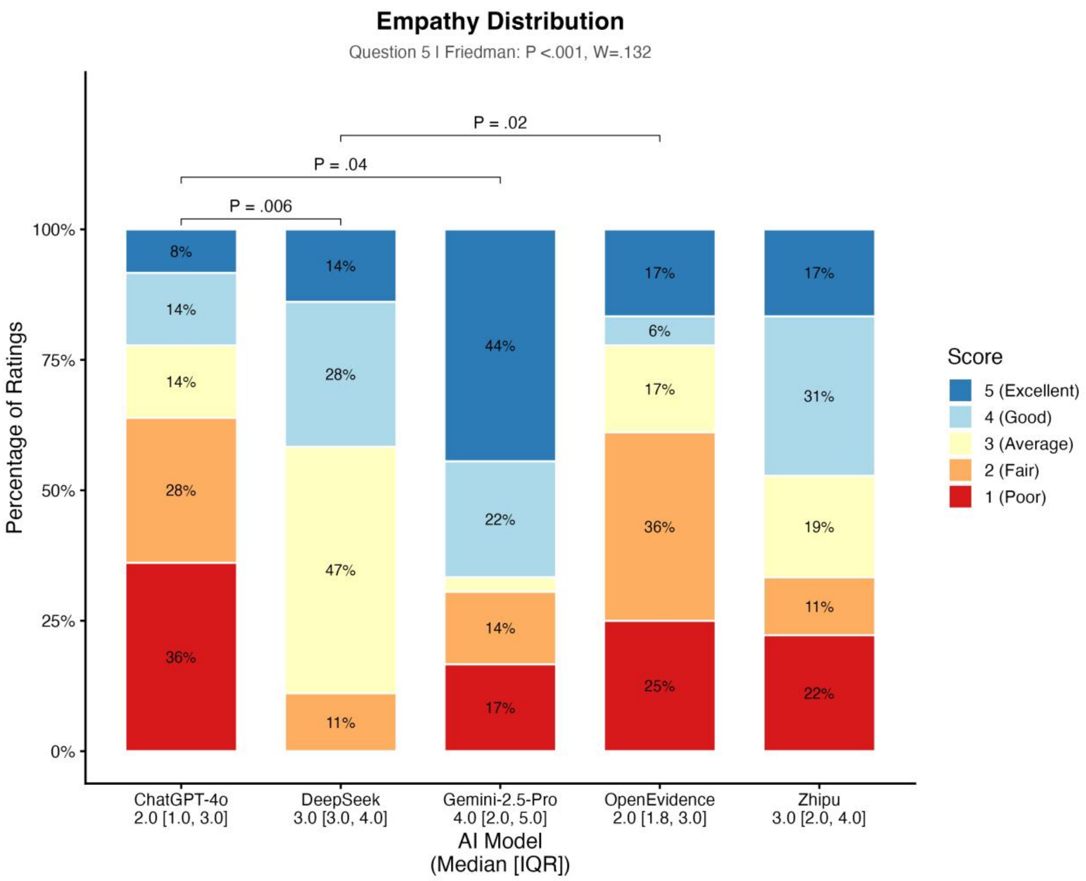
Empathy Distribution
Question 5 I Friedman: P <.001, W=.132
P = .02
P = .04
P = .006
Percentage of Ratings
100%
75%
50%
25%
0%
8%
14%
14%
28%
36%
14%
28%
47%
11%
44%
22%
14%
17%
17%
6%
17%
36%
25%
17%
31%
19%
11%
22%
ChatGPT-4o
2.0 [1.0, 3.0]
DeepSeek
3.0 [3.0, 4.0]
Gemini-2.5-Pro
4.0 [2.0, 5.0]
OpenEvidence
2.0 [1.8, 3.0]
Zhipu
3.0 [2.0, 4.0]
AI Model
(Median [IQR])
Score
5 (Excellent)
4 (Good)
3 (Average)
2 (Fair)
1 (Poor)

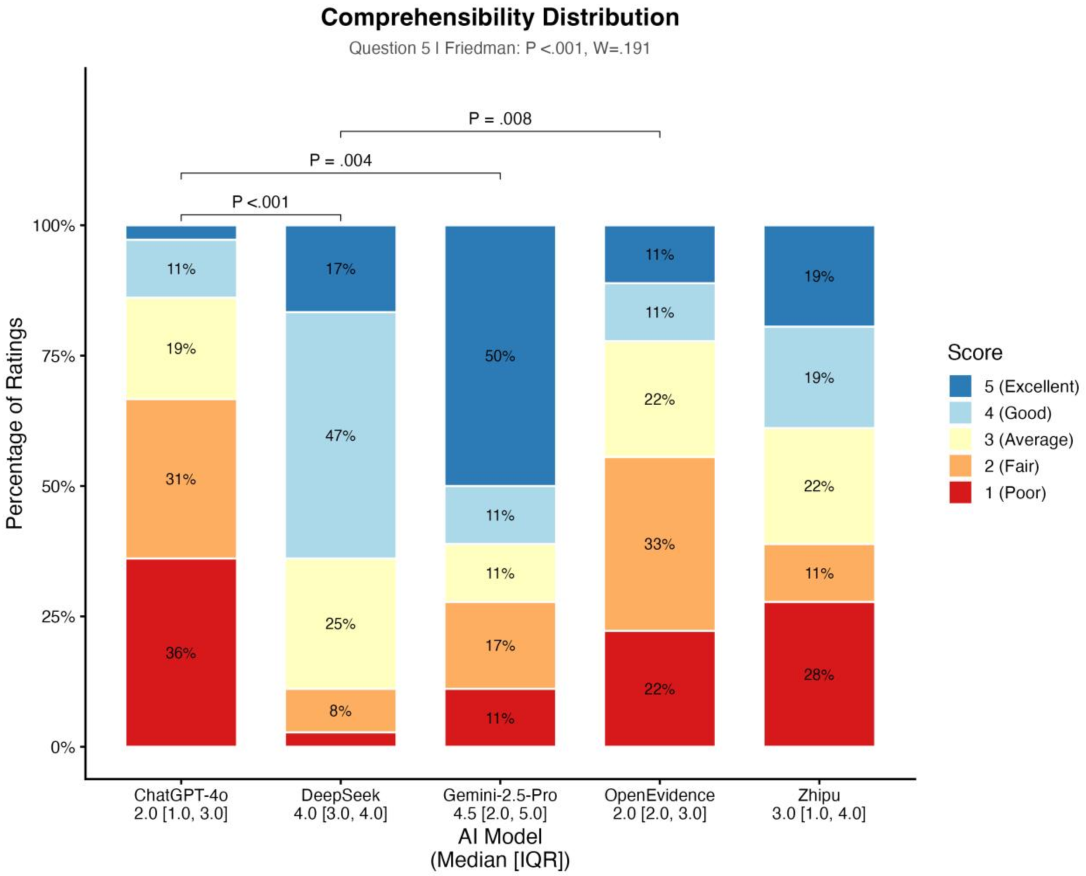
Comprehensibility Distribution
Question 5 I Friedman: P <.001, W=.191
P = .008
P = .004
P <.001
Percentage of Ratings
100%
75%
50%
25%
0%
11%
19%
31%
36%
17%
47%
25%
8%
50%
11%
11%
17%
11%
11%
11%
22%
33%
22%
19%
19%
22%
11%
28%
ChatGPT-4o
2.0 [1.0, 3.0]
DeepSeek
4.0 [3.0, 4.0]
Gemini-2.5-Pro
4.5 [2.0, 5.0]
OpenEvidence
2.0 [2.0, 3.0]
Zhipu
3.0 [1.0, 4.0]
AI Model
(Median [IQR])
Score
5 (Excellent)
4 (Good)
3 (Average)
2 (Fair)
1 (Poor)

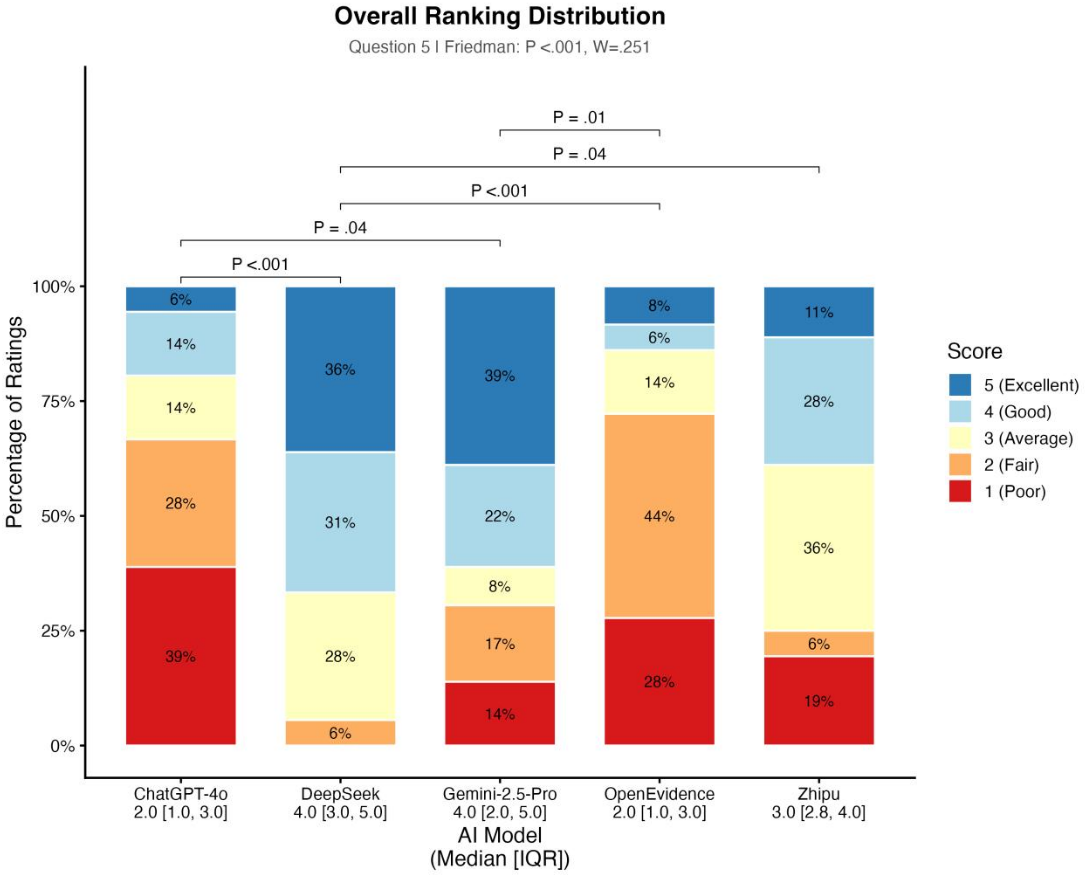
Overall Ranking Distribution
Question 5 | Friedman: P <.001, W=.251
P = .01
P = .04
P <.001
P = .04
P <.001
Percentage of Ratings
100%
75%
50%
25%
0%
6%
14%
14%
28%
39%
36%
31%
28%
6%
39%
22%
8%
17%
14%
8%
6%
14%
44%
28%
11%
28%
36%
6%
19%
Score
5 (Excellent)
4 (Good)
3 (Average)
2 (Fair)
1 (Poor)
ChatGPT-4o
2.0 [1.0, 3.0]
DeepSeek
4.0 [3.0, 5.0]
Gemini-2.5-Pro
4.0 [2.0, 5.0]
OpenEvidence
2.0 [1.0, 3.0]
Zhipu
3.0 [2.8, 4.0]
AI Model
(Median [IQR])

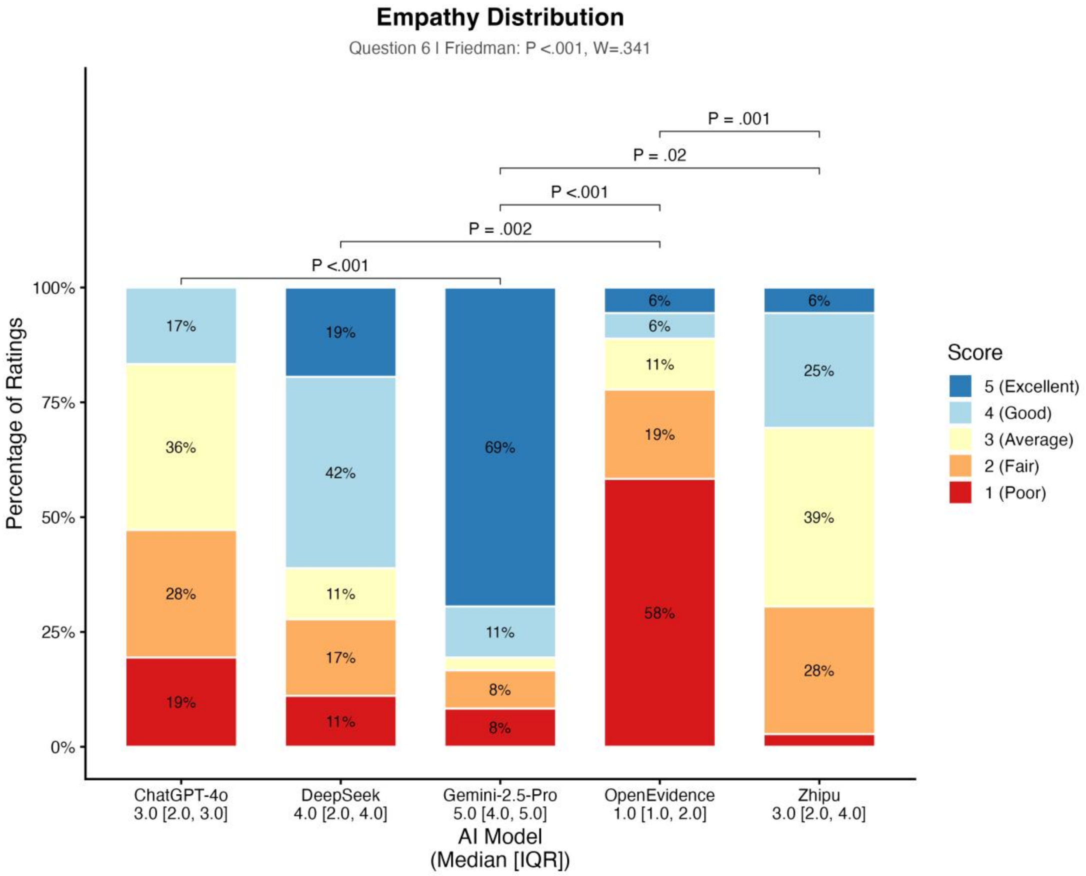
Empathy Distribution
Question 6 | Friedman: P <.001, W=.341
P = .001
P = .02
P <.001
P = .002
P <.001
Percentage of Ratings
100%
75%
50%
25%
0%
17%
36%
28%
19%
19%
42%
11%
17%
11%
69%
11%
8%
8%
6%
6%
11%
19%
58%
6%
25%
39%
28%
Score
5 (Excellent)
4 (Good)
3 (Average)
2 (Fair)
1 (Poor)
ChatGPT-4o
3.0 [2.0, 3.0]
DeepSeek
4.0 [2.0, 4.0]
Gemini-2.5-Pro
5.0 [4.0, 5.0]
OpenEvidence
1.0 [1.0, 2.0]
Zhipu
3.0 [2.0, 4.0]
AI Model
(Median [IQR])

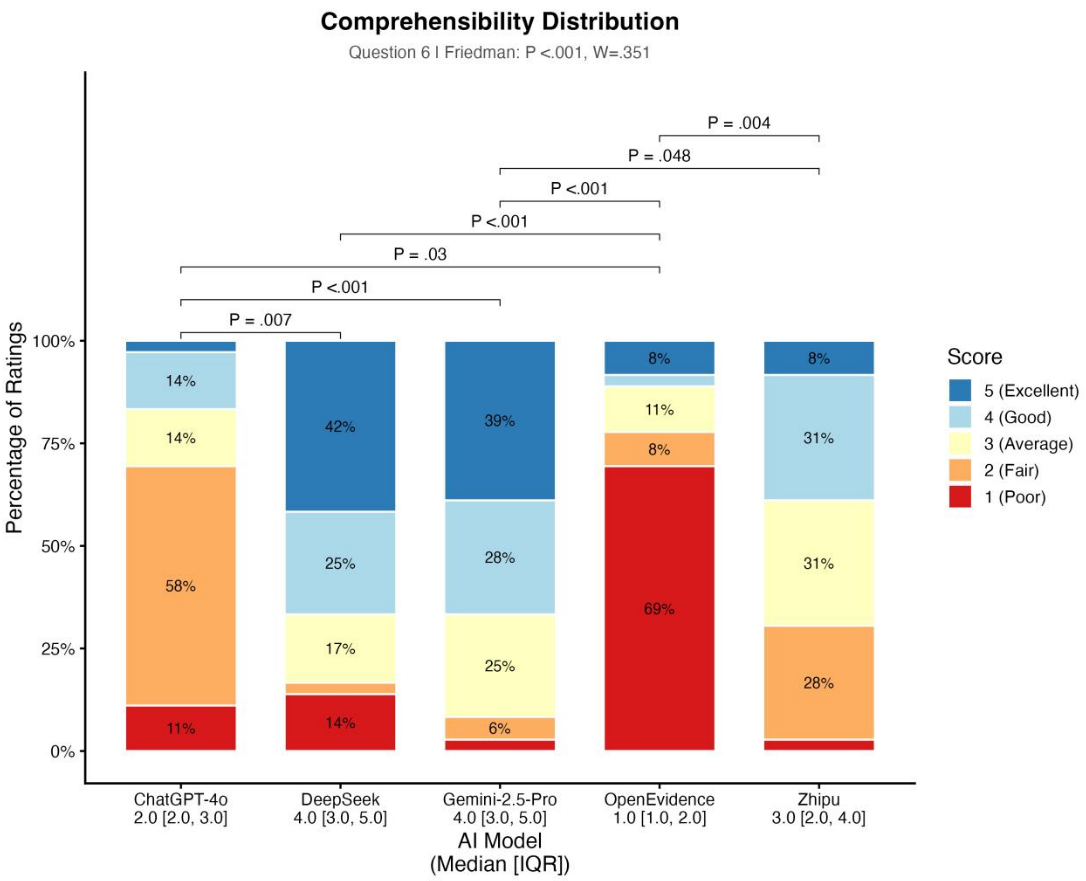
Comprehensibility Distribution
Question 6 I Friedman: P <.001, W=.351
P = .004
P = .048
P <.001
P <.001
P = .03
P <.001
P = .007
Percentage of Ratings
100%
75%
50%
25%
0%
14%
14%
58%
11%
42%
25%
17%
14%
39%
28%
25%
6%
8%
11%
8%
69%
8%
31%
31%
28%
ChatGPT-4o
2.0 [2.0, 3.0]
DeepSeek
4.0 [3.0, 5.0]
Gemini-2.5-Pro
4.0 [3.0, 5.0]
OpenEvidence
1.0 [1.0, 2.0]
Zhipu
3.0 [2.0, 4.0]
AI Model
(Median [IQR])
Score
5 (Excellent)
4 (Good)
3 (Average)
2 (Fair)
1 (Poor)

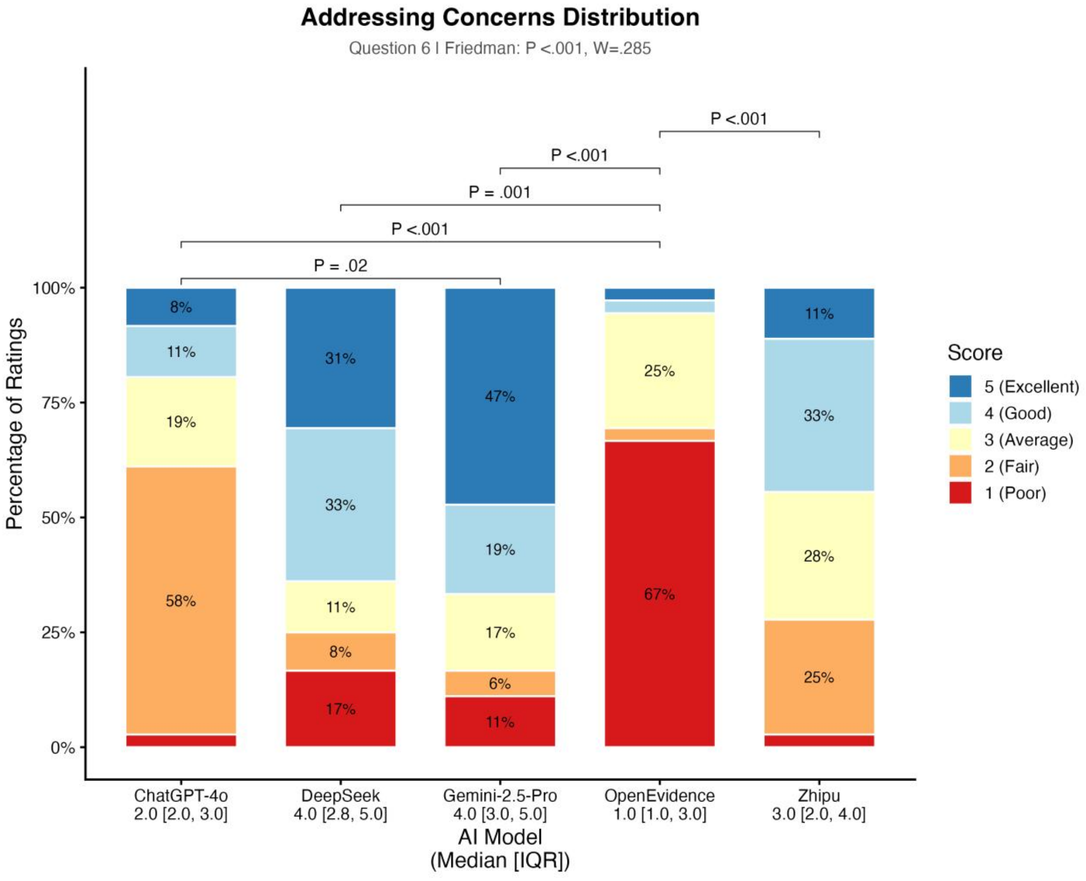
Addressing Concerns Distribution
Question 6 I Friedman: P <.001, W=.285
P <.001
P <.001
P = .001
P <.001
P = .02
Percentage of Ratings
100%
75%
50%
25%
0%
8%
11%
19%
58%
31%
33%
11%
8%
17%
47%
19%
17%
6%
11%
25%
67%
11%
33%
28%
25%
ChatGPT-4o
2.0 [2.0, 3.0]
DeepSeek
4.0 [2.8, 5.0]
Gemini-2.5-Pro
4.0 [3.0, 5.0]
OpenEvidence
1.0 [1.0, 3.0]
Zhipu
3.0 [2.0, 4.0]
AI Model
(Median [IQR])
Score
5 (Excellent)
4 (Good)
3 (Average)
2 (Fair)
1 (Poor)

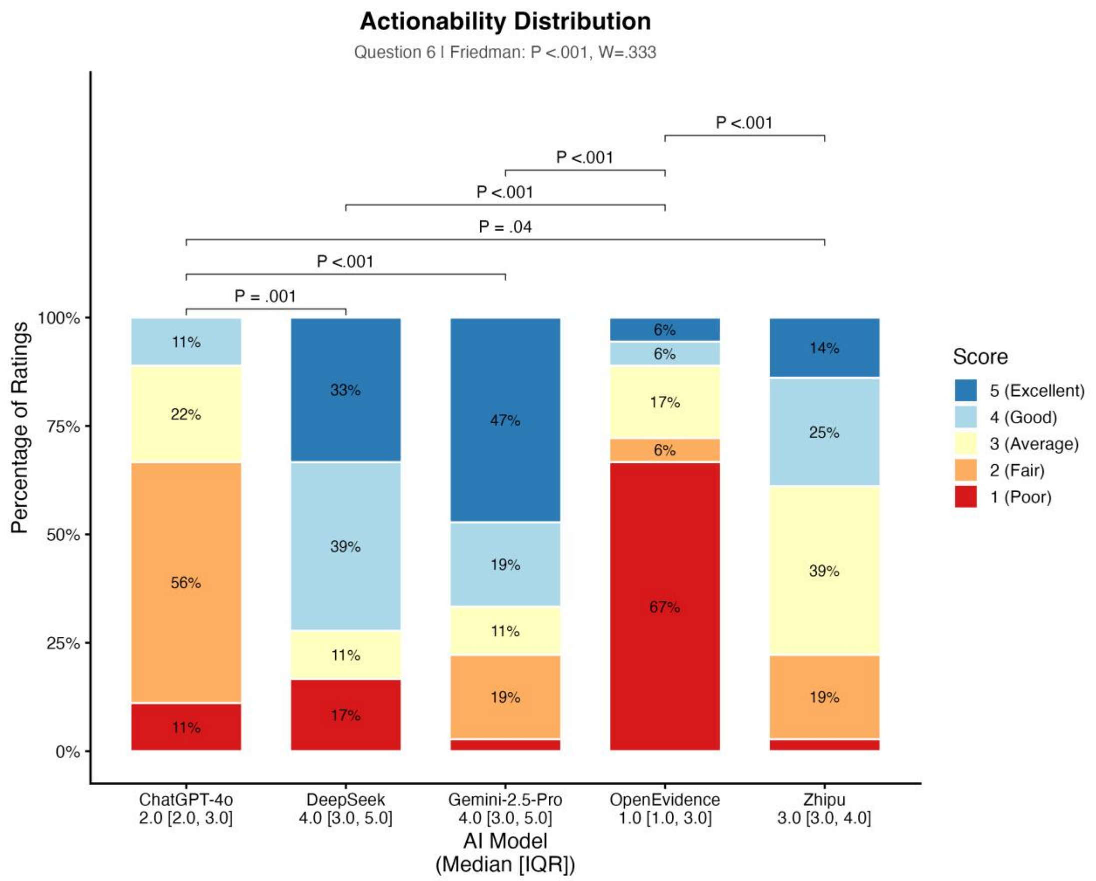
Actionability Distribution
Question 6 | Friedman: P <.001, W=.333
P <.001
P <.001
P <.001
P = .04
P <.001
P = .001
Percentage of Ratings
100%
75%
50%
25%
0%
11%
22%
56%
11%
33%
39%
11%
17%
47%
19%
11%
19%
6%
6%
17%
6%
67%
14%
25%
39%
19%
Score
5 (Excellent)
4 (Good)
3 (Average)
2 (Fair)
1 (Poor)
ChatGPT-4o 2.0 [2.0, 3.0]
DeepSeek 4.0 [3.0, 5.0]
Gemini-2.5-Pro 4.0 [3.0, 5.0]
OpenEvidence 1.0 [1.0, 3.0]
Zhipu 3.0 [3.0, 4.0]
AI Model (Median [IQR])

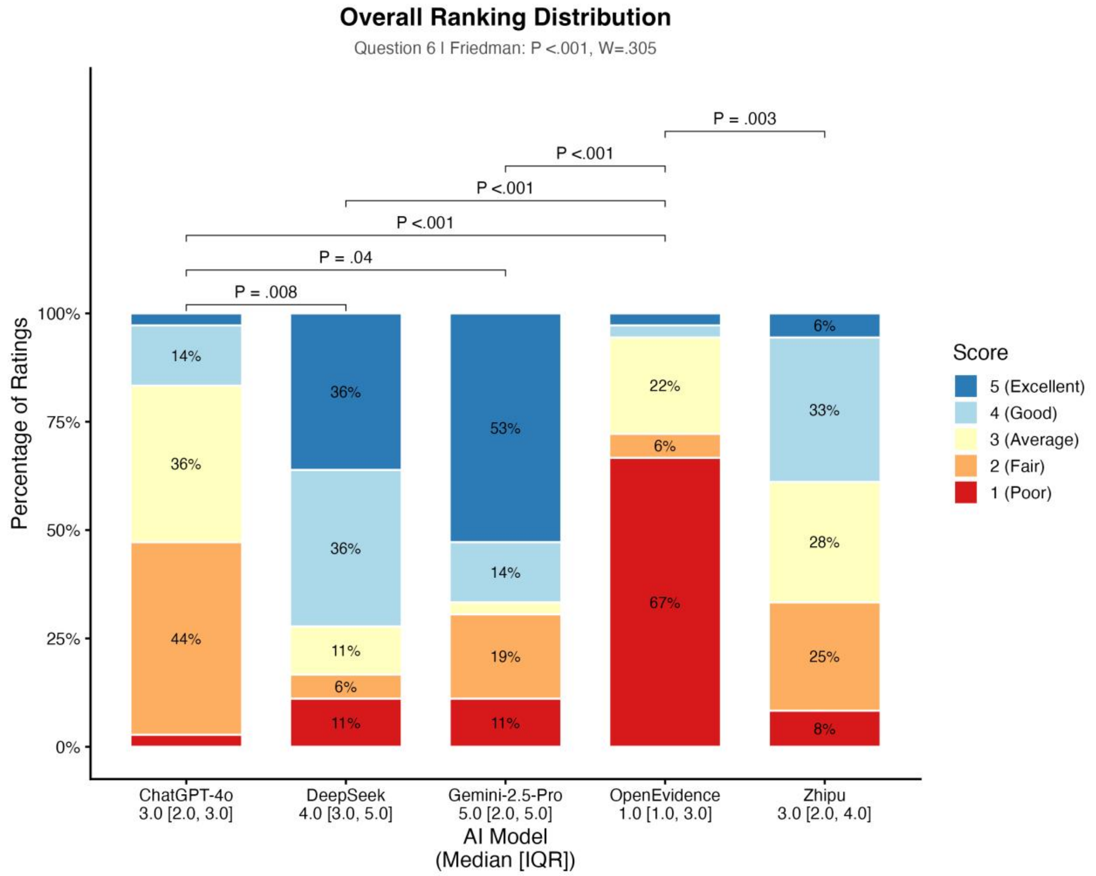
Overall Ranking Distribution
Question 6 | Friedman: P <.001, W=.305
P = .003
P <.001
P <.001
P <.001
P = .04
P = .008
Percentage of Ratings
100%
75%
50%
25%
0%
14%
36%
44%
36%
36%
11%
6%
11%
53%
14%
19%
11%
22%
6%
67%
6%
33%
28%
25%
8%
Score
5 (Excellent)
4 (Good)
3 (Average)
2 (Fair)
1 (Poor)
ChatGPT-4o 3.0 [2.0, 3.0]
DeepSeek 4.0 [3.0, 5.0]
Gemini-2.5-Pro 5.0 [2.0, 5.0]
OpenEvidence 1.0 [1.0, 3.0]
Zhipu 3.0 [2.0, 4.0]
AI Model (Median [IQR])

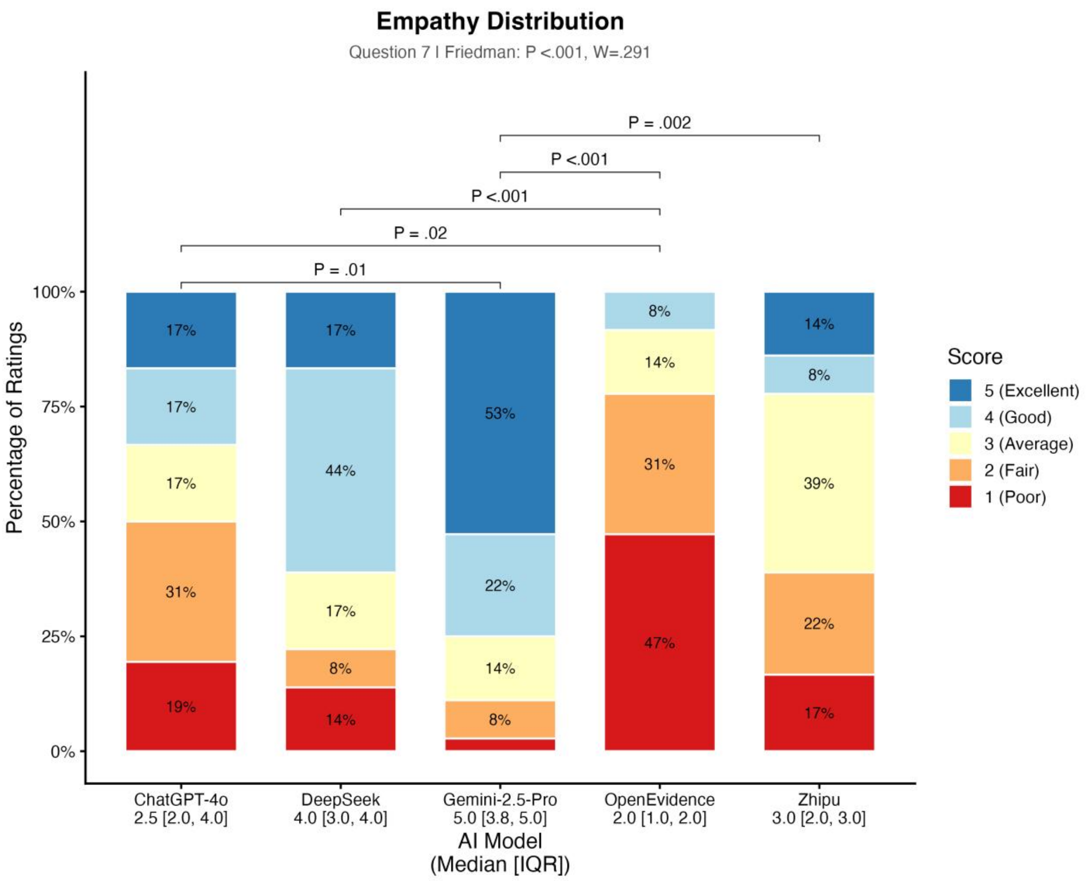
Empathy Distribution
Question 7 | Friedman: P <.001, W=.291
P = .002
P <.001
P <.001
P = .02
P = .01
Percentage of Ratings
100%
75%
50%
25%
0%
ChatGPT-4o
2.5 [2.0, 4.0]
DeepSeek
4.0 [3.0, 4.0]
Gemini-2.5-Pro
5.0 [3.8, 5.0]
OpenEvidence
2.0 [1.0, 2.0]
Zhipu
3.0 [2.0, 3.0]
AI Model
(Median [IQR])
Score
5 (Excellent)
4 (Good)
3 (Average)
2 (Fair)
1 (Poor)

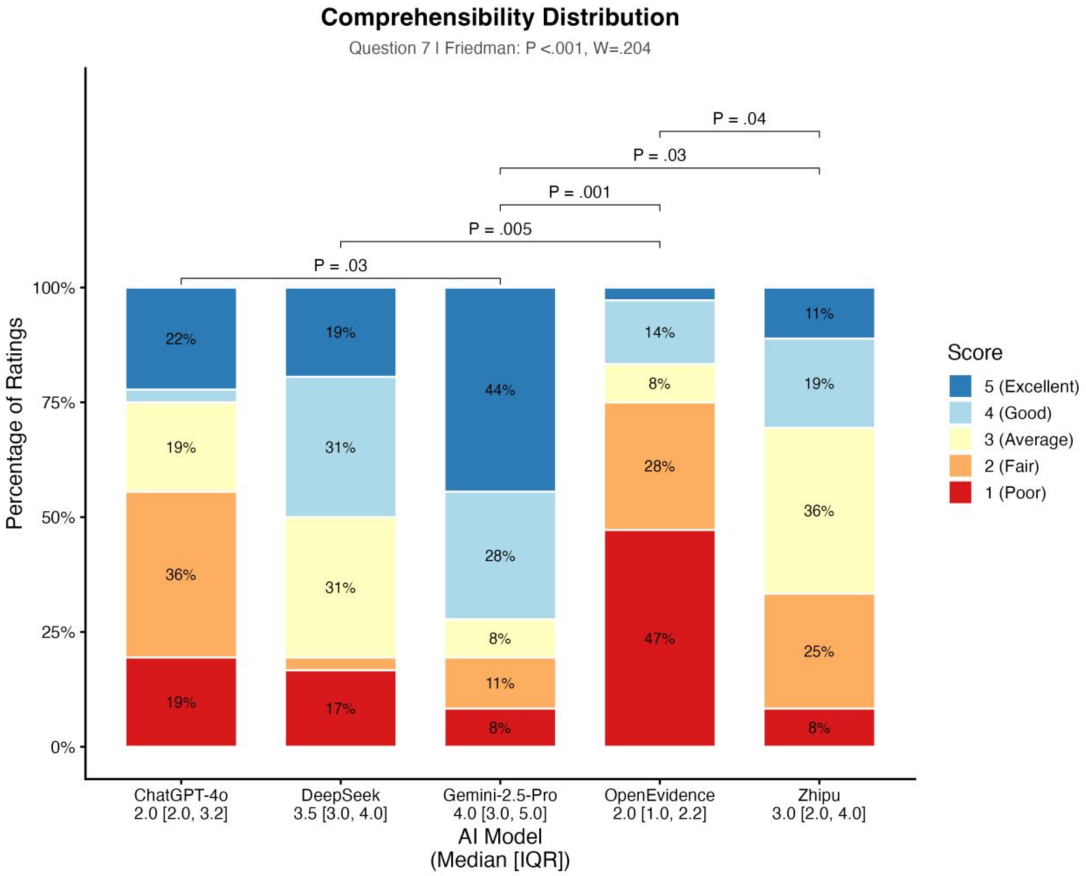
Comprehensibility Distribution
Question 7 | Friedman: P <.001, W=.204
P = .04
P = .03
P = .001
P = .005
P = .03
Percentage of Ratings
100%
75%
50%
25%
0%
ChatGPT-4o
2.0 [2.0, 3.2]
DeepSeek
3.5 [3.0, 4.0]
Gemini-2.5-Pro
4.0 [3.0, 5.0]
OpenEvidence
2.0 [1.0, 2.2]
Zhipu
3.0 [2.0, 4.0]
AI Model
(Median [IQR])
Score
5 (Excellent)
4 (Good)
3 (Average)
2 (Fair)
1 (Poor)

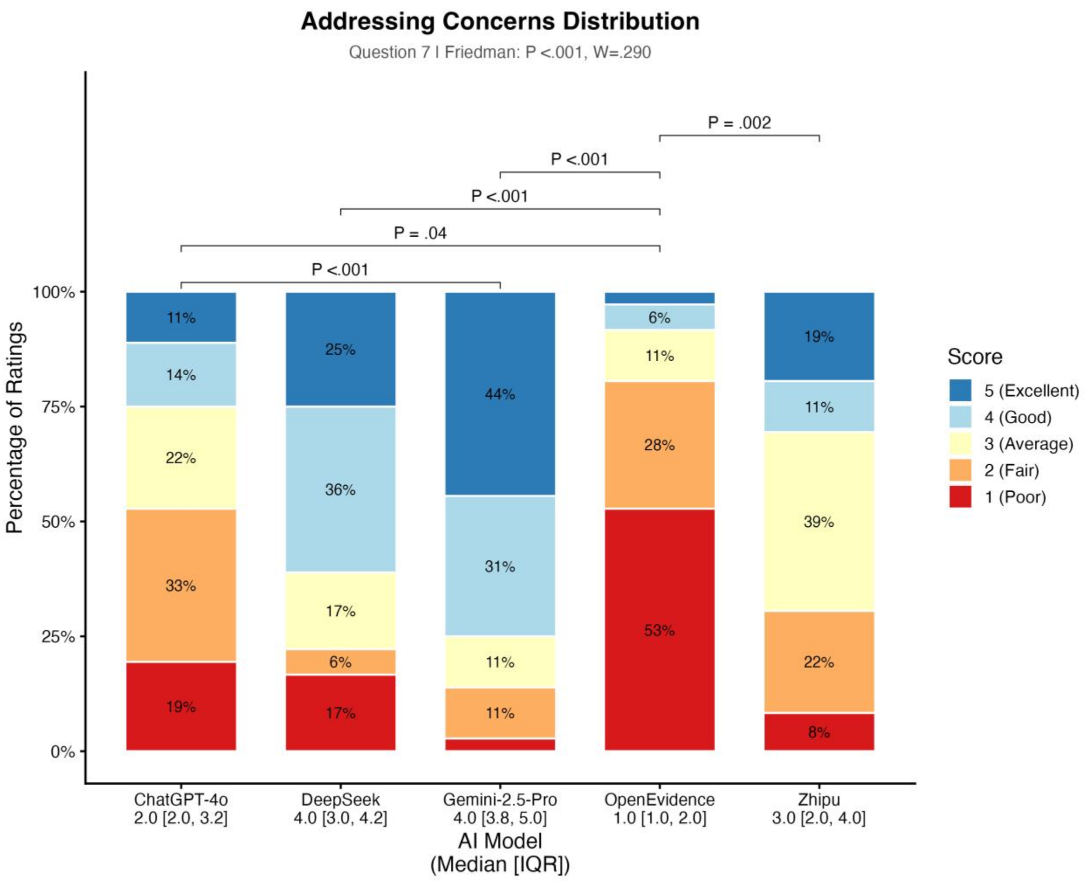
Addressing Concerns Distribution
Question 7 I Friedman: P <.001, W=.290
P = .002
P <.001
P <.001
P = .04
P <.001
100%
75%
50%
25%
0%
Percentage of Ratings
11%
14%
22%
33%
19%
25%
36%
17%
6%
17%
44%
31%
11%
11%
6%
11%
28%
53%
19%
11%
39%
22%
8%
Score
5 (Excellent)
4 (Good)
3 (Average)
2 (Fair)
1 (Poor)
ChatGPT-4o
2.0 [2.0, 3.2]
DeepSeek
4.0 [3.0, 4.2]
Gemini-2.5-Pro
4.0 [3.8, 5.0]
OpenEvidence
1.0 [1.0, 2.0]
Zhipu
3.0 [2.0, 4.0]
AI Model
(Median [IQR])

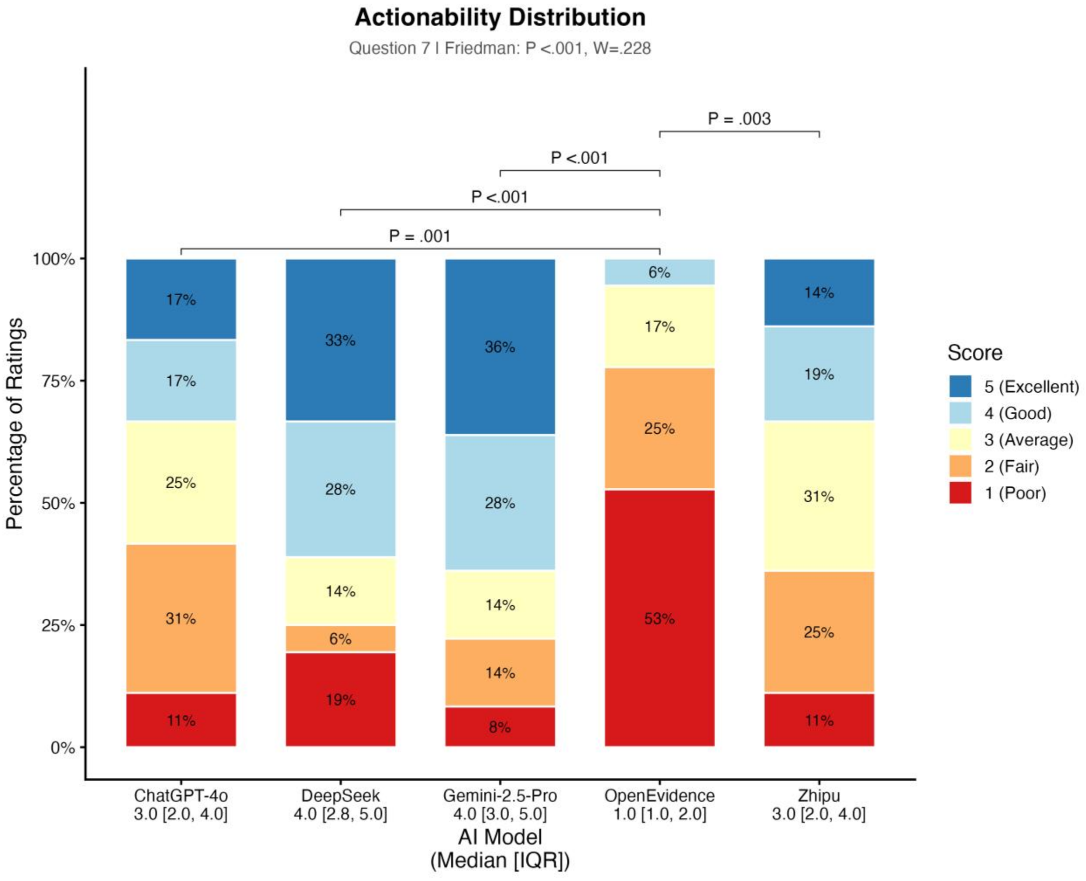
Actionability Distribution
Question 7 I Friedman: P <.001, W=.228
P = .003
P <.001
P <.001
P = .001
100%
75%
50%
25%
0%
Percentage of Ratings
17%
17%
25%
31%
11%
33%
28%
14%
6%
19%
36%
28%
14%
14%
8%
6%
17%
25%
53%
14%
19%
31%
25%
11%
Score
5 (Excellent)
4 (Good)
3 (Average)
2 (Fair)
1 (Poor)
ChatGPT-4o
3.0 [2.0, 4.0]
DeepSeek
4.0 [2.8, 5.0]
Gemini-2.5-Pro
4.0 [3.0, 5.0]
OpenEvidence
1.0 [1.0, 2.0]
Zhipu
3.0 [2.0, 4.0]
AI Model
(Median [IQR])

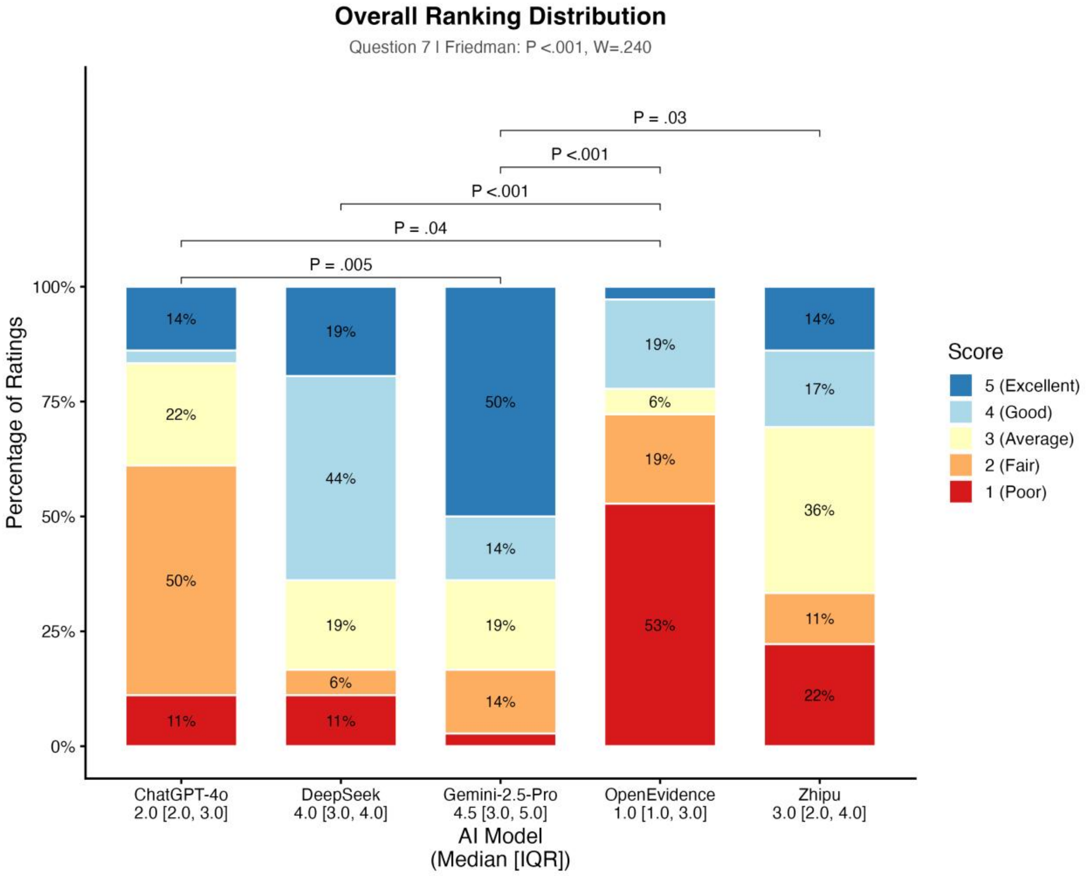
Overall Ranking Distribution
Question 7 l Friedman: P <.001, W=.240
P = .03
P <.001
P <.001
P = .04
P = .005
Percentage of Ratings
100%
75%
50%
25%
0%
14%
22%
50%
11%
19%
44%
19%
6%
11%
50%
14%
19%
14%
19%
6%
19%
53%
14%
17%
36%
11%
22%
Score
5 (Excellent)
4 (Good)
3 (Average)
2 (Fair)
1 (Poor)
ChatGPT-4o
2.0 [2.0, 3.0]
DeepSeek
4.0 [3.0, 4.0]
Gemini-2.5-Pro
4.5 [3.0, 5.0]
OpenEvidence
1.0 [1.0, 3.0]
Zhipu
3.0 [2.0, 4.0]
AI Model
(Median [IQR])

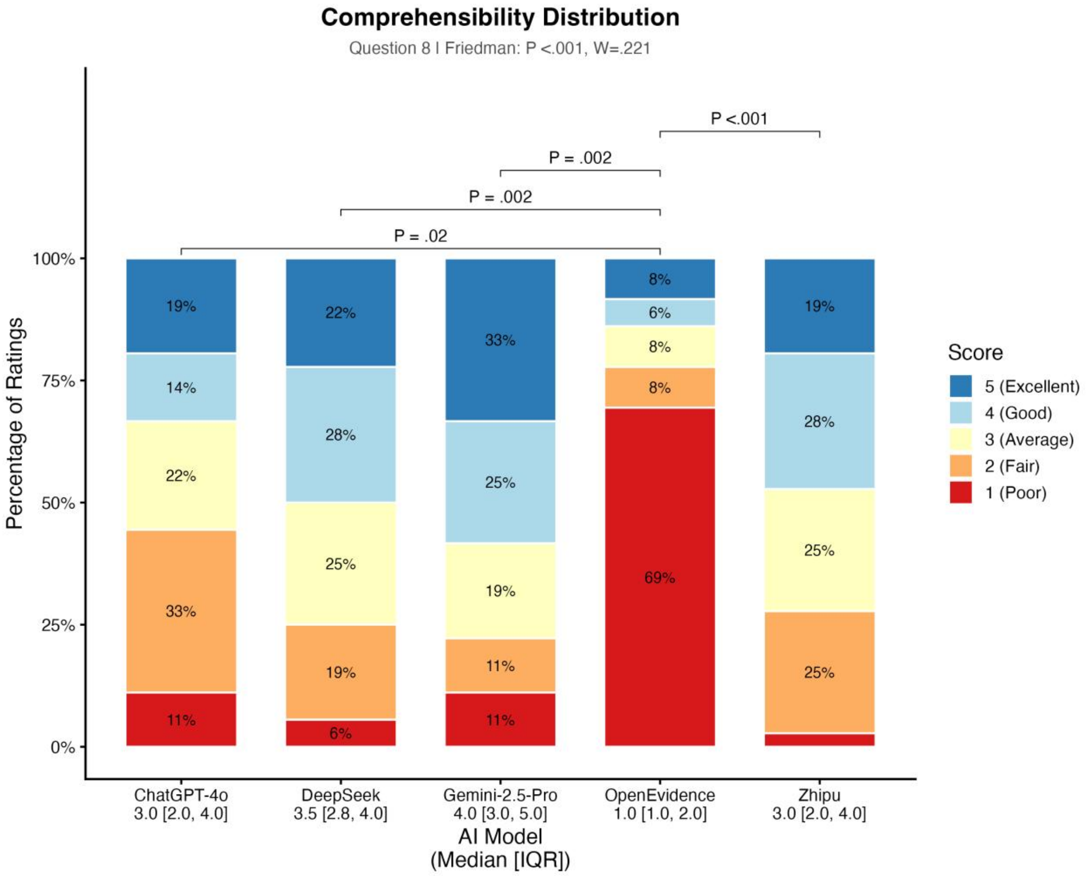
Comprehensibility Distribution
Question 8 l Friedman: P <.001, W=.221
P <.001
P = .002
P = .002
P = .02
Percentage of Ratings
100%
75%
50%
25%
0%
19%
14%
22%
33%
11%
22%
28%
25%
19%
6%
33%
25%
19%
11%
11%
8%
6%
8%
8%
69%
19%
28%
25%
25%
Score
5 (Excellent)
4 (Good)
3 (Average)
2 (Fair)
1 (Poor)
ChatGPT-4o
3.0 [2.0, 4.0]
DeepSeek
3.5 [2.8, 4.0]
Gemini-2.5-Pro
4.0 [3.0, 5.0]
OpenEvidence
1.0 [1.0, 2.0]
Zhipu
3.0 [2.0, 4.0]
AI Model
(Median [IQR])

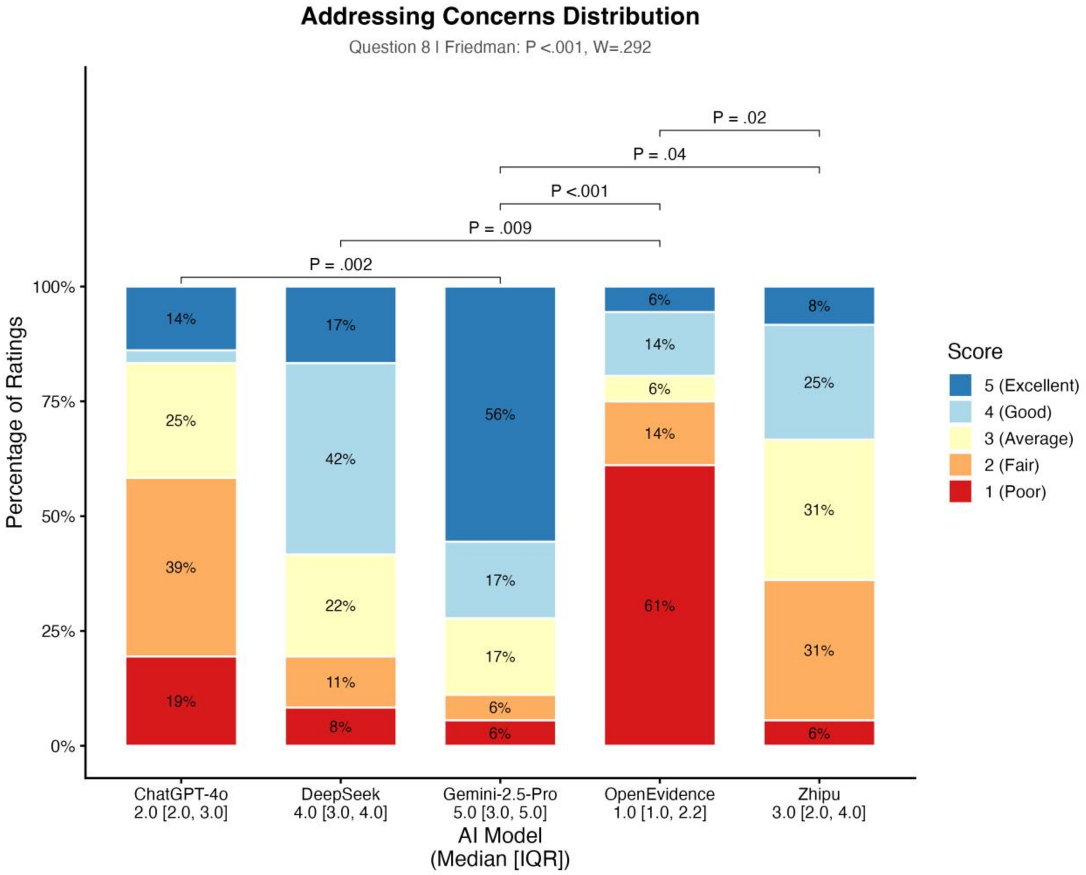

Addressing Concerns Distribution
Question 8 | Friedman: P <.001, W=.292
P = .02
P = .04
P <.001
P = .009
P = .002
100%
75%
50%
25%
0%
Percentage of Ratings
14%
25%
39%
19%
17%
42%
22%
11%
8%
56%
17%
17%
6%
6%
6%
14%
6%
14%
61%
8%
25%
31%
31%
6%
ChatGPT-4o
2.0 [2.0, 3.0]
DeepSeek
4.0 [3.0, 4.0]
Gemini-2.5-Pro
5.0 [3.0, 5.0]
OpenEvidence
1.0 [1.0, 2.2]
Zhipu
3.0 [2.0, 4.0]
AI Model
(Median [IQR])
Score
5 (Excellent)
4 (Good)
3 (Average)
2 (Fair)
1 (Poor)

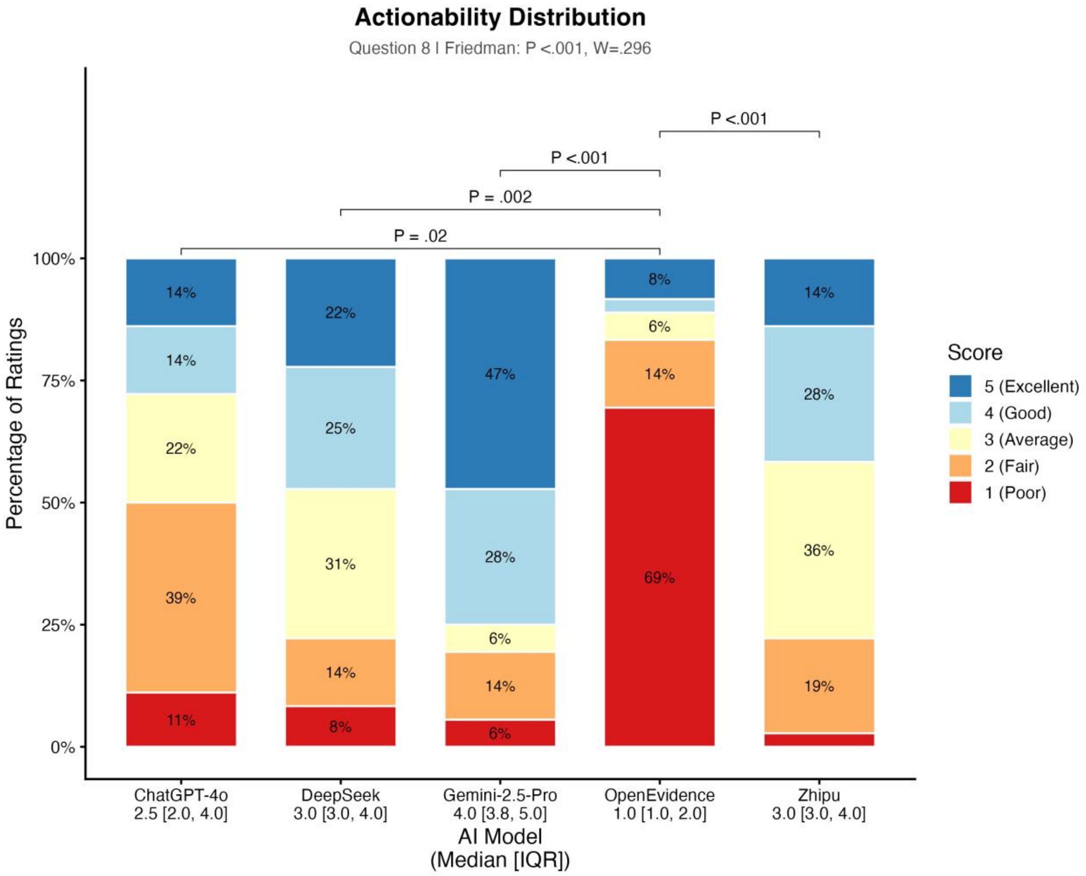

Actionability Distribution
Question 8 | Friedman: P <.001, W=.296
P <.001
P <.001
P = .002
P = .02
100%
75%
50%
25%
0%
Percentage of Ratings
14%
14%
22%
39%
11%
22%
25%
31%
14%
8%
47%
28%
6%
14%
6%
8%
6%
14%
69%
14%
28%
36%
19%
ChatGPT-4o
2.5 [2.0, 4.0]
DeepSeek
3.0 [3.0, 4.0]
Gemini-2.5-Pro
4.0 [3.8, 5.0]
OpenEvidence
1.0 [1.0, 2.0]
Zhipu
3.0 [3.0, 4.0]
AI Model
(Median [IQR])
Score
5 (Excellent)
4 (Good)
3 (Average)
2 (Fair)
1 (Poor)

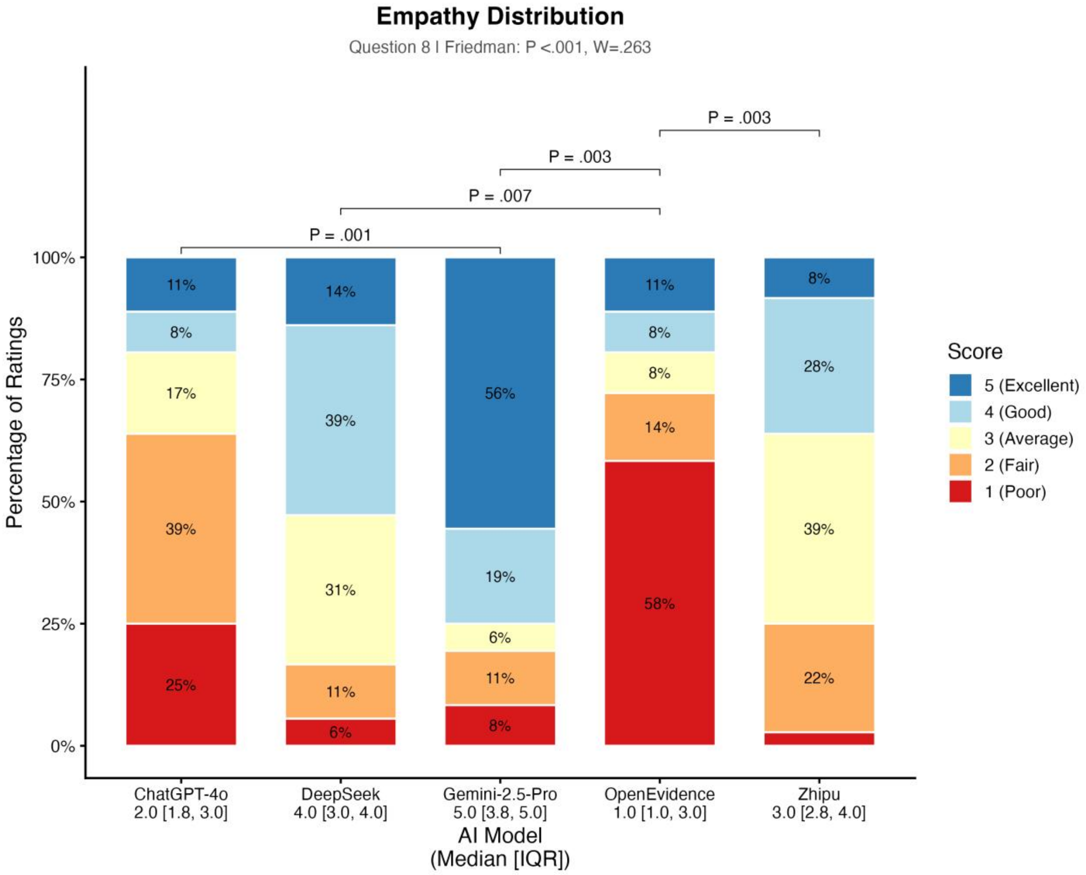
Empathy Distribution
Question 8 | Friedman: P <.001, W=.263
P = .003
P = .003
P = .007
P = .001
100%
75%
50%
25%
0%
Percentage of Ratings
11%
8%
17%
39%
25%
14%
39%
31%
11%
6%
56%
19%
6%
11%
8%
11%
8%
8%
14%
58%
8%
28%
39%
22%
ChatGPT-4o
2.0 [1.8, 3.0]
DeepSeek
4.0 [3.0, 4.0]
Gemini-2.5-Pro
5.0 [3.8, 5.0]
OpenEvidence
1.0 [1.0, 3.0]
Zhipu
3.0 [2.8, 4.0]
AI Model
(Median [IQR])
Score
5 (Excellent)
4 (Good)
3 (Average)
2 (Fair)
1 (Poor)

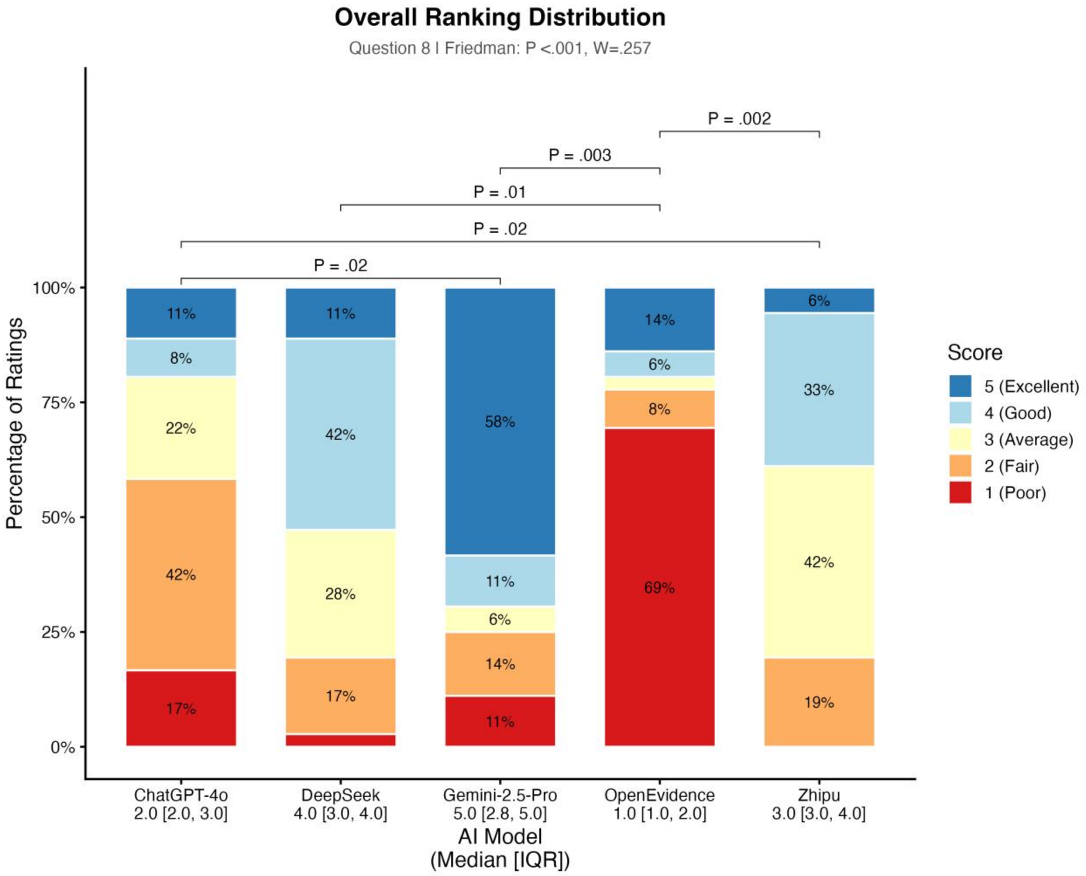
Overall Ranking Distribution
Question 8 | Friedman: P <.001, W=.257
P = .002
P = .003
P = .01
P = .02
P = .02
100%
75%
50%
25%
0%
Percentage of Ratings
11%
8%
22%
42%
17%
11%
42%
28%
17%
58%
11%
6%
14%
11%
14%
6%
8%
69%
6%
33%
42%
19%
ChatGPT-4o
2.0 [2.0, 3.0]
DeepSeek
4.0 [3.0, 4.0]
Gemini-2.5-Pro
5.0 [2.8, 5.0]
OpenEvidence
1.0 [1.0, 2.0]
Zhipu
3.0 [3.0, 4.0]
AI Model
(Median [IQR])
Score
5 (Excellent)
4 (Good)
3 (Average)
2 (Fair)
1 (Poor)

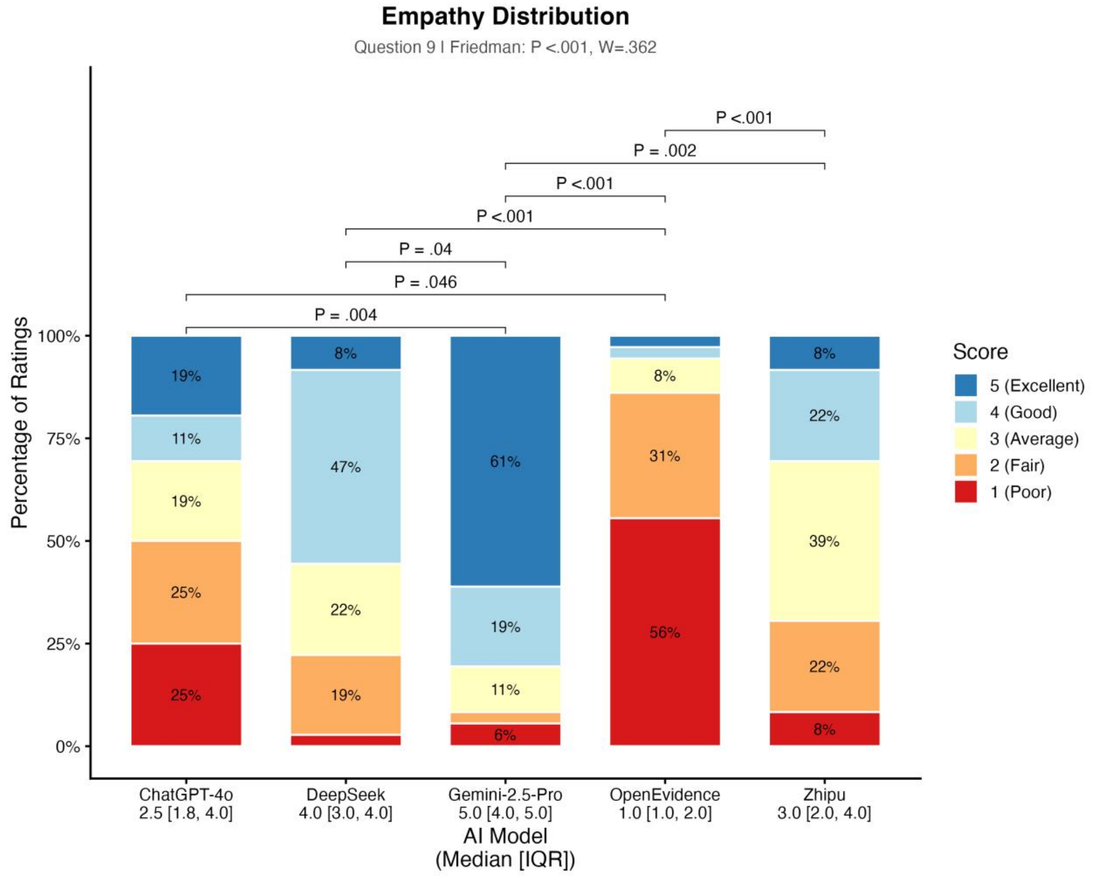

Empathy Distribution
Question 9 | Friedman: P <.001, W=.362
P <.001
P = .002
P <.001
P <.001
P = .04
P = .046
P = .004
Percentage of Ratings
100%
75%
50%
25%
0%
19%
11%
19%
25%
25%
8%
47%
22%
19%
61%
19%
11%
6%
8%
31%
56%
8%
22%
39%
22%
8%
ChatGPT-4o
2.5 [1.8, 4.0]
DeepSeek
4.0 [3.0, 4.0]
Gemini-2.5-Pro
5.0 [4.0, 5.0]
OpenEvidence
1.0 [1.0, 2.0]
Zhipu
3.0 [2.0, 4.0]
AI Model
(Median [IQR])
Score
5 (Excellent)
4 (Good)
3 (Average)
2 (Fair)
1 (Poor)

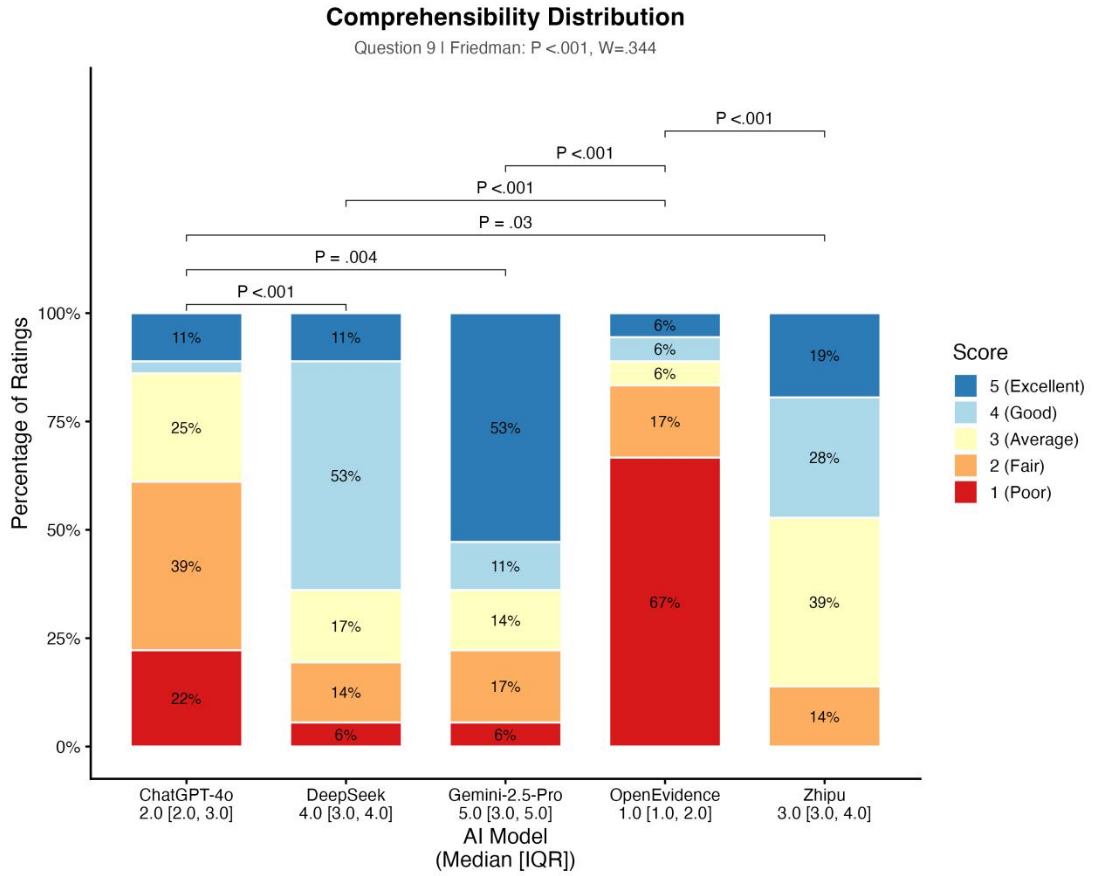

Comprehensibility Distribution
Question 9 | Friedman: P <.001, W=.344
P <.001
P <.001
P <.001
P = .03
P = .004
P <.001
Percentage of Ratings
100%
75%
50%
25%
0%
11%
25%
39%
22%
11%
53%
17%
14%
6%
53%
11%
14%
17%
6%
6%
6%
6%
17%
67%
19%
28%
39%
14%
ChatGPT-4o
2.0 [2.0, 3.0]
DeepSeek
4.0 [3.0, 4.0]
Gemini-2.5-Pro
5.0 [3.0, 5.0]
OpenEvidence
1.0 [1.0, 2.0]
Zhipu
3.0 [3.0, 4.0]
AI Model
(Median [IQR])
Score
5 (Excellent)
4 (Good)
3 (Average)
2 (Fair)
1 (Poor)

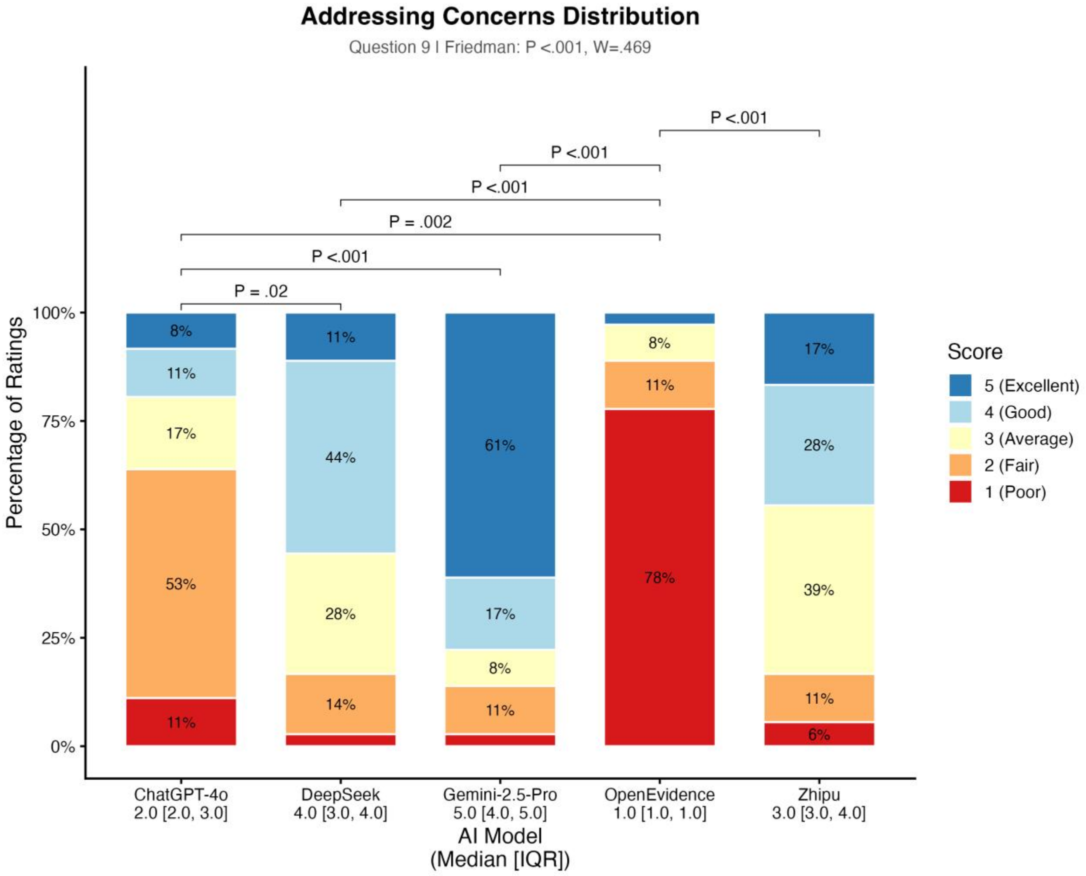
Addressing Concerns Distribution
Question 9 | Friedman: P <.001, W=.469
P <.001
P <.001
P <.001
P = .002
P <.001
P = .02
Percentage of Ratings
100%
75%
50%
25%
0%
8%
11%
17%
53%
11%
11%
44%
28%
14%
61%
17%
8%
11%
8%
11%
78%
17%
28%
39%
11%
6%
ChatGPT-4o
2.0 [2.0, 3.0]
DeepSeek
4.0 [3.0, 4.0]
Gemini-2.5-Pro
5.0 [4.0, 5.0]
OpenEvidence
1.0 [1.0, 1.0]
Zhipu
3.0 [3.0, 4.0]
AI Model
(Median [IQR])
Score
5 (Excellent)
4 (Good)
3 (Average)
2 (Fair)
1 (Poor)

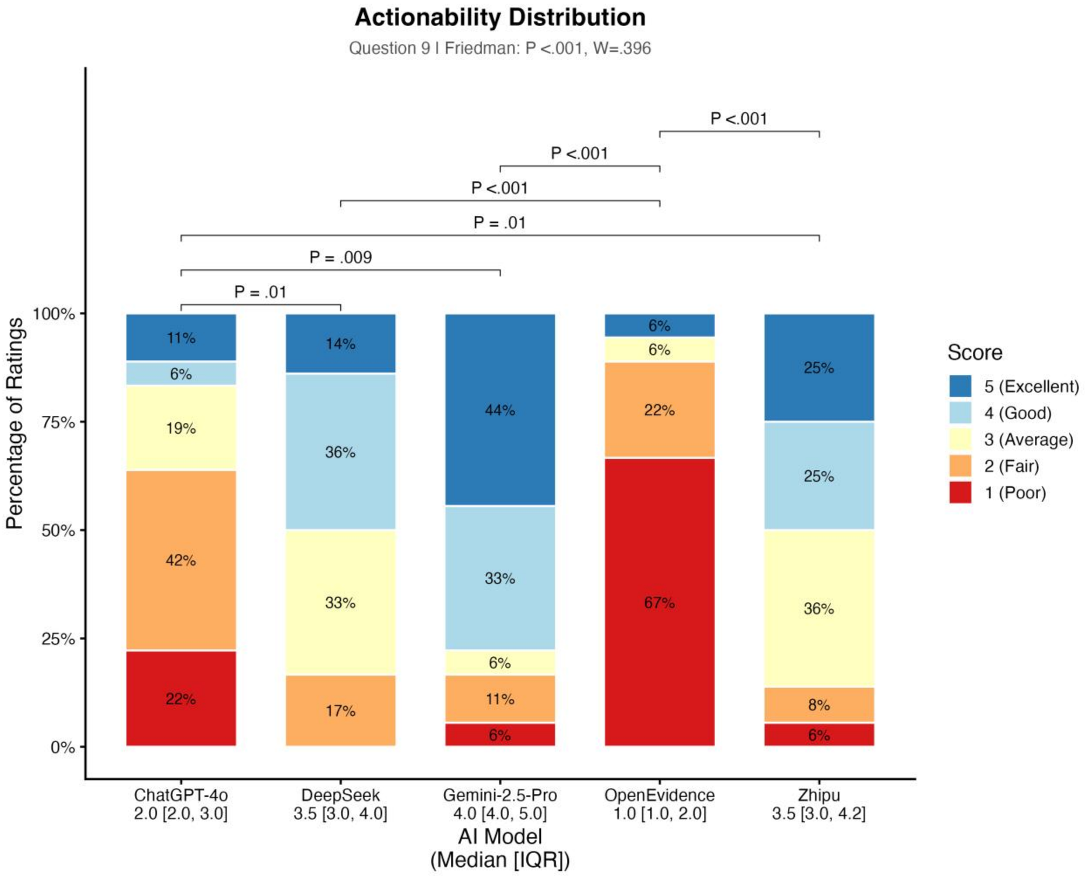
Actionability Distribution
Question 9 | Friedman: P <.001, W=.396
P <.001
P <.001
P <.001
P = .01
P = .009
P = .01
Percentage of Ratings
100%
75%
50%
25%
0%
11%
6%
19%
42%
22%
14%
36%
33%
17%
44%
33%
6%
11%
6%
6%
6%
22%
67%
25%
25%
36%
8%
6%
ChatGPT-4o
2.0 [2.0, 3.0]
DeepSeek
3.5 [3.0, 4.0]
Gemini-2.5-Pro
4.0 [4.0, 5.0]
OpenEvidence
1.0 [1.0, 2.0]
Zhipu
3.5 [3.0, 4.2]
AI Model
(Median [IQR])
Score
5 (Excellent)
4 (Good)
3 (Average)
2 (Fair)
1 (Poor)

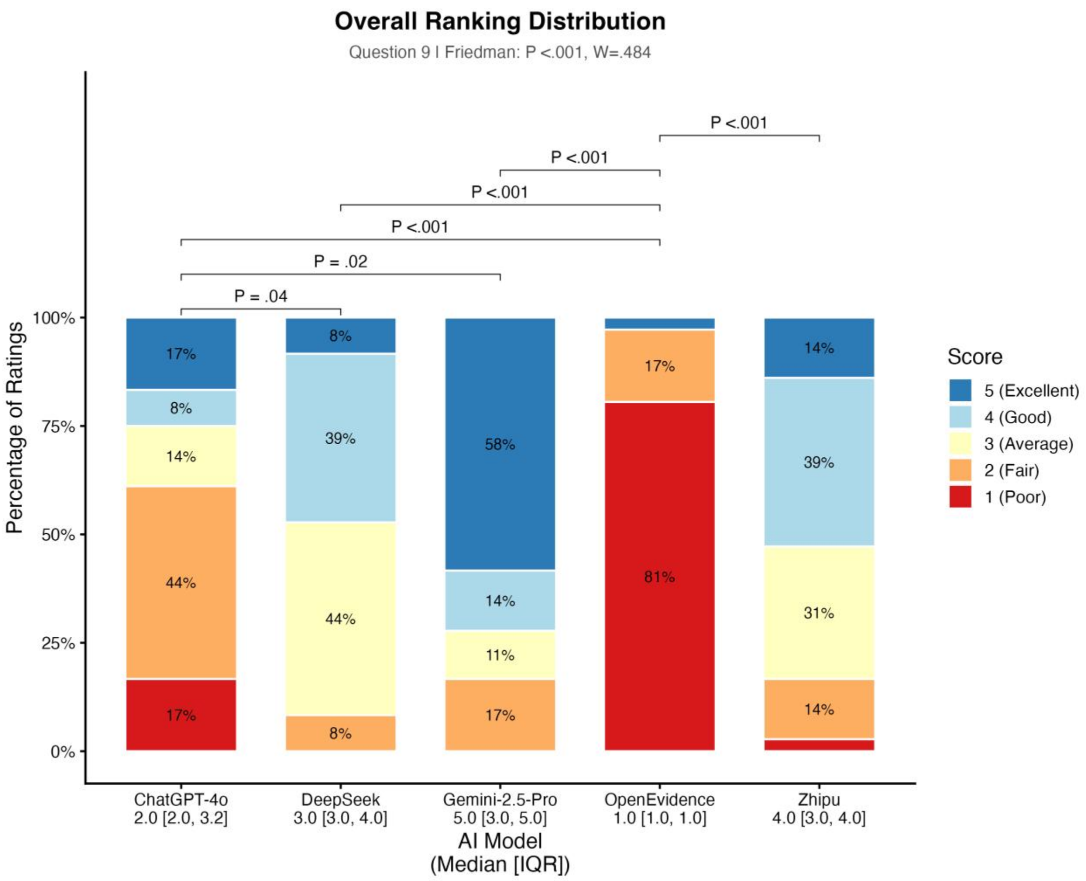

Overall Ranking Distribution
Question 9 I Friedman: P <.001, W=.484
P <.001
P <.001
P <.001
P <.001
P = .02
P = .04
Percentage of Ratings
100%
75%
50%
25%
0%
17%
8%
14%
44%
17%
8%
39%
44%
8%
58%
14%
11%
17%
17%
81%
14%
39%
31%
14%
ChatGPT-4o
2.0 [2.0, 3.2]
DeepSeek
3.0 [3.0, 4.0]
Gemini-2.5-Pro
5.0 [3.0, 5.0]
OpenEvidence
1.0 [1.0, 1.0]
Zhipu
4.0 [3.0, 4.0]
AI Model
(Median [IQR])
Score
5 (Excellent)
4 (Good)
3 (Average)
2 (Fair)
1 (Poor)

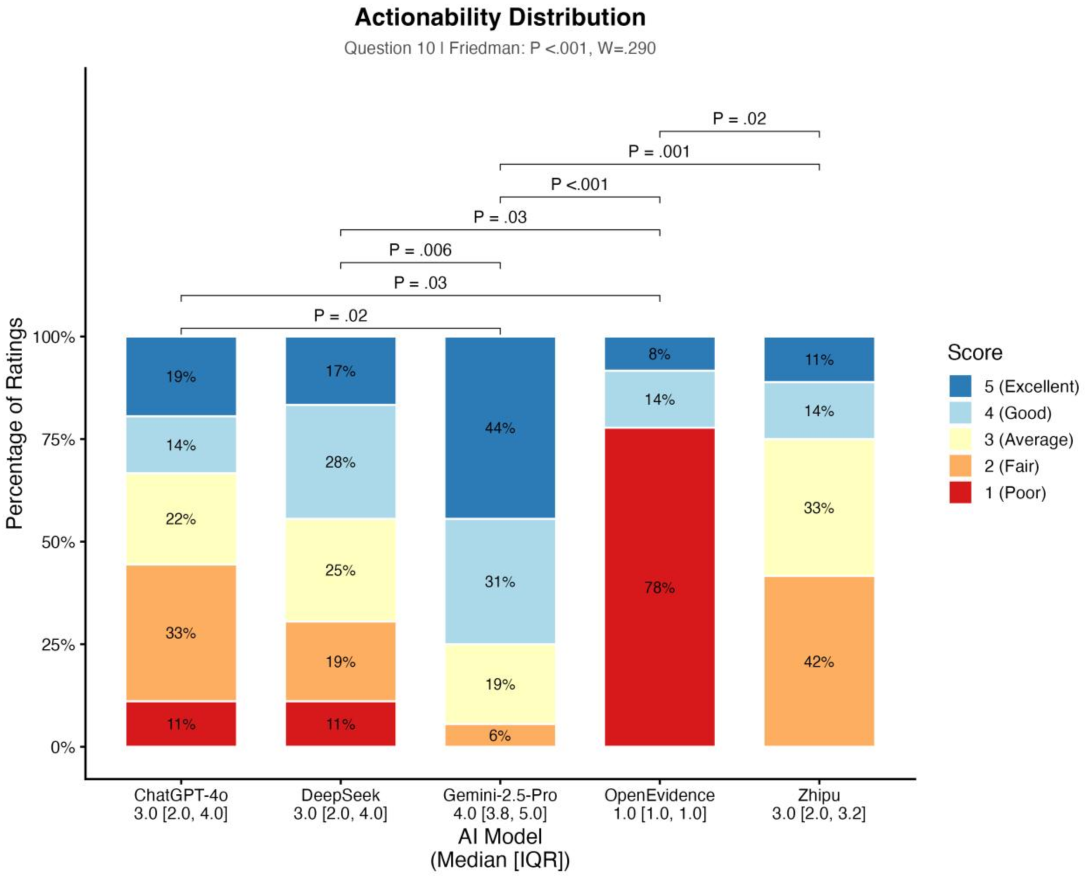

Actionability Distribution
Question 10 I Friedman: P <.001, W=.290
P = .02
P = .001
P <.001
P = .03
P = .006
P = .03
P = .02
Percentage of Ratings
100%
75%
50%
25%
0%
19%
14%
22%
33%
11%
17%
28%
25%
19%
11%
44%
31%
19%
6%
8%
14%
78%
11%
14%
33%
42%
ChatGPT-4o
3.0 [2.0, 4.0]
DeepSeek
3.0 [2.0, 4.0]
Gemini-2.5-Pro
4.0 [3.8, 5.0]
OpenEvidence
1.0 [1.0, 1.0]
Zhipu
3.0 [2.0, 3.2]
AI Model
(Median [IQR])
Score
5 (Excellent)
4 (Good)
3 (Average)
2 (Fair)
1 (Poor)

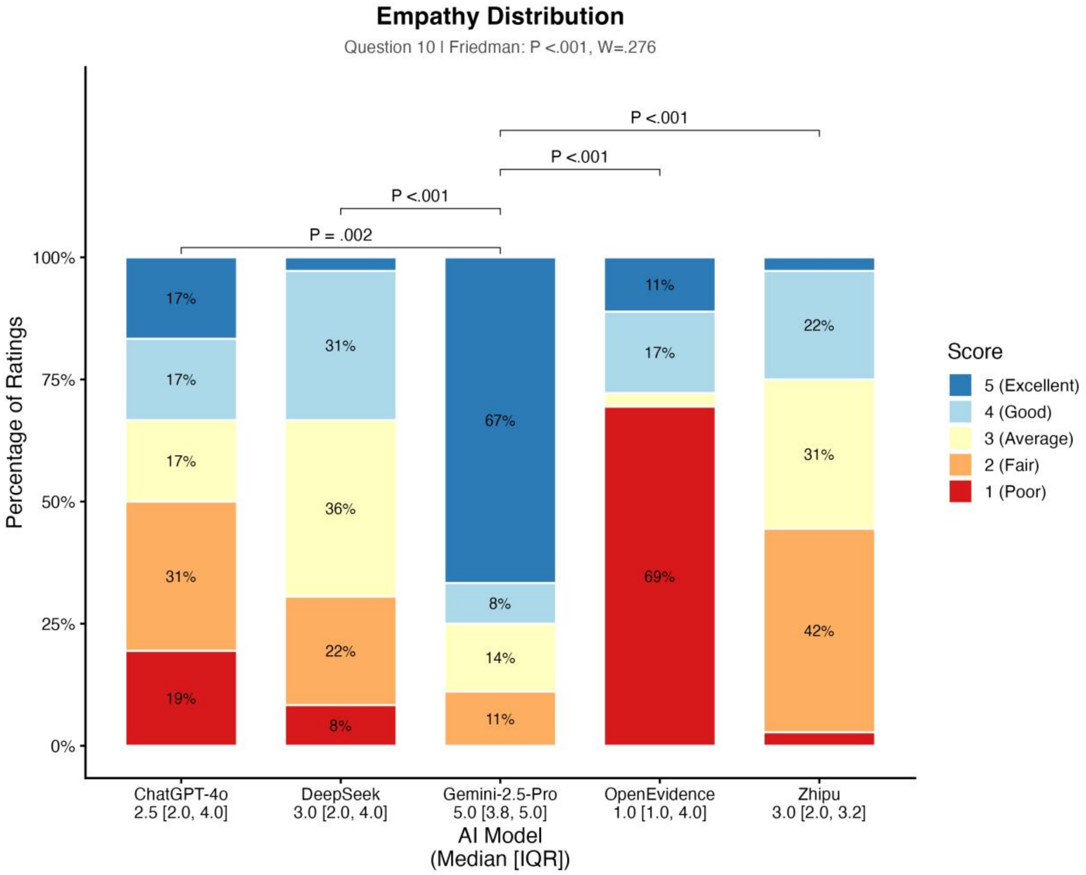
Empathy Distribution
Question 10 | Friedman: P <.001, W=.276
P <.001
P <.001
P <.001
P = .002
Percentage of Ratings
100%
75%
50%
25%
0%
17%
17%
17%
31%
19%
31%
36%
22%
8%
67%
8%
14%
11%
11%
17%
69%
22%
31%
42%
ChatGPT-4o
2.5 [2.0, 4.0]
DeepSeek
3.0 [2.0, 4.0]
Gemini-2.5-Pro
5.0 [3.8, 5.0]
OpenEvidence
1.0 [1.0, 4.0]
Zhipu
3.0 [2.0, 3.2]
AI Model
(Median [IQR])
Score
5 (Excellent)
4 (Good)
3 (Average)
2 (Fair)
1 (Poor)

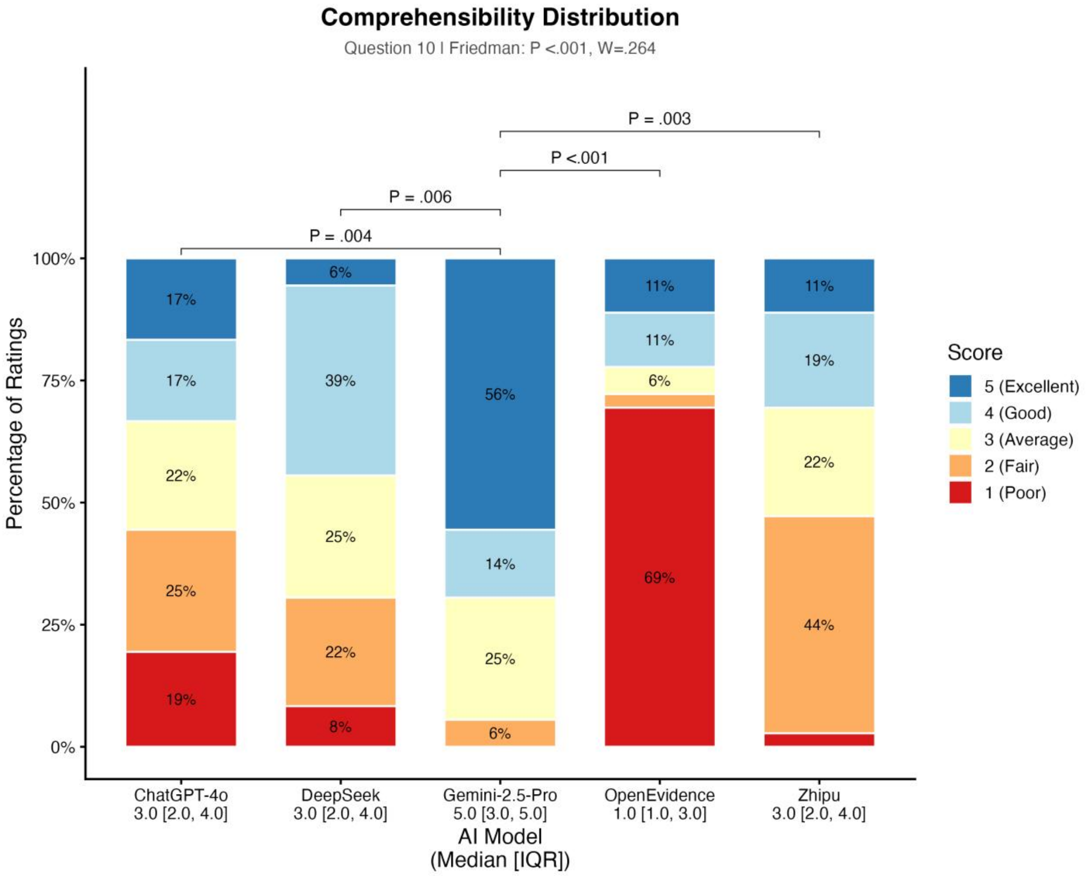
Comprehensibility Distribution
Question 10 | Friedman: P <.001, W=.264
P = .003
P <.001
P = .006
P = .004
Percentage of Ratings
100%
75%
50%
25%
0%
17%
17%
22%
25%
19%
6%
39%
25%
22%
8%
56%
14%
25%
6%
11%
11%
6%
69%
11%
19%
22%
44%
ChatGPT-4o
3.0 [2.0, 4.0]
DeepSeek
3.0 [2.0, 4.0]
Gemini-2.5-Pro
5.0 [3.0, 5.0]
OpenEvidence
1.0 [1.0, 3.0]
Zhipu
3.0 [2.0, 4.0]
AI Model
(Median [IQR])
Score
5 (Excellent)
4 (Good)
3 (Average)
2 (Fair)
1 (Poor)

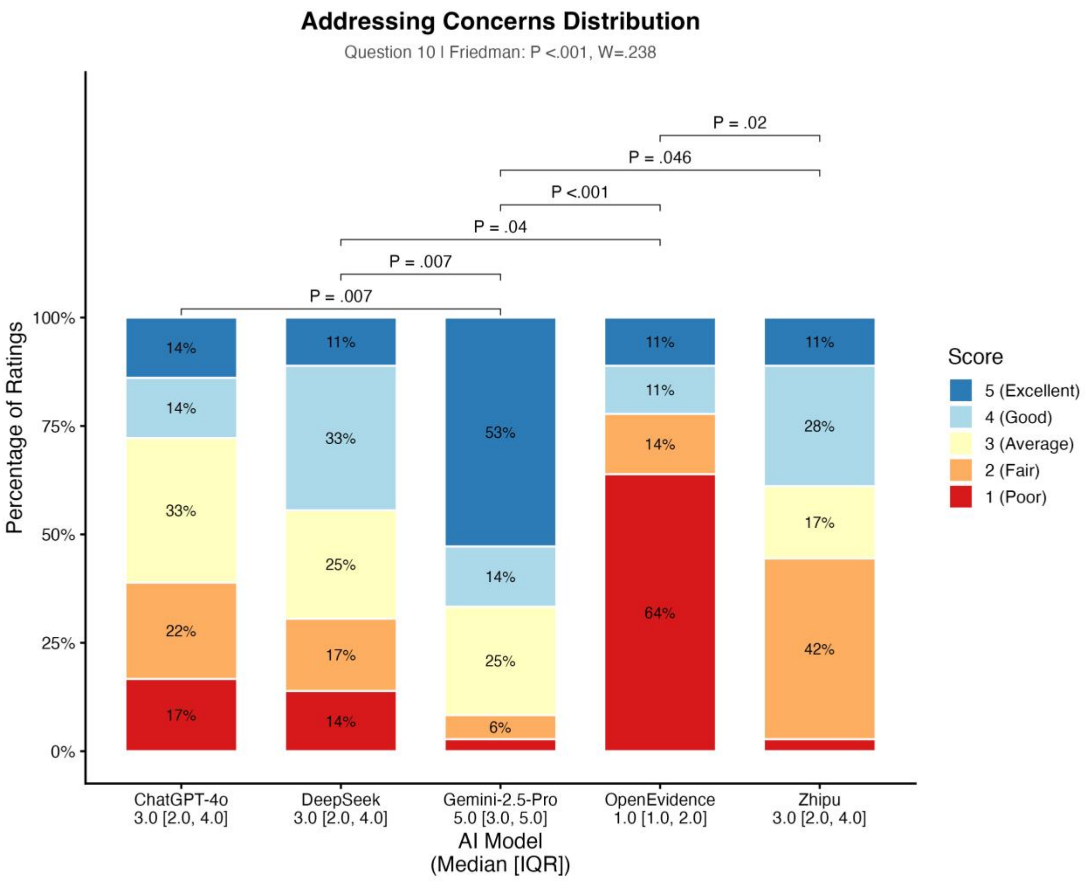
Addressing Concerns Distribution
Question 10 | Friedman: P <.001, W=.238
P = .02
P = .046
P <.001
P = .04
P = .007
P = .007
Percentage of Ratings
100%
75%
50%
25%
0%
14%
14%
33%
22%
17%
11%
33%
25%
17%
14%
53%
14%
25%
6%
11%
11%
14%
64%
11%
28%
17%
42%
Score
5 (Excellent)
4 (Good)
3 (Average)
2 (Fair)
1 (Poor)
ChatGPT-4o
3.0 [2.0, 4.0]
DeepSeek
3.0 [2.0, 4.0]
Gemini-2.5-Pro
5.0 [3.0, 5.0]
OpenEvidence
1.0 [1.0, 2.0]
Zhipu
3.0 [2.0, 4.0]
AI Model
(Median [IQR])

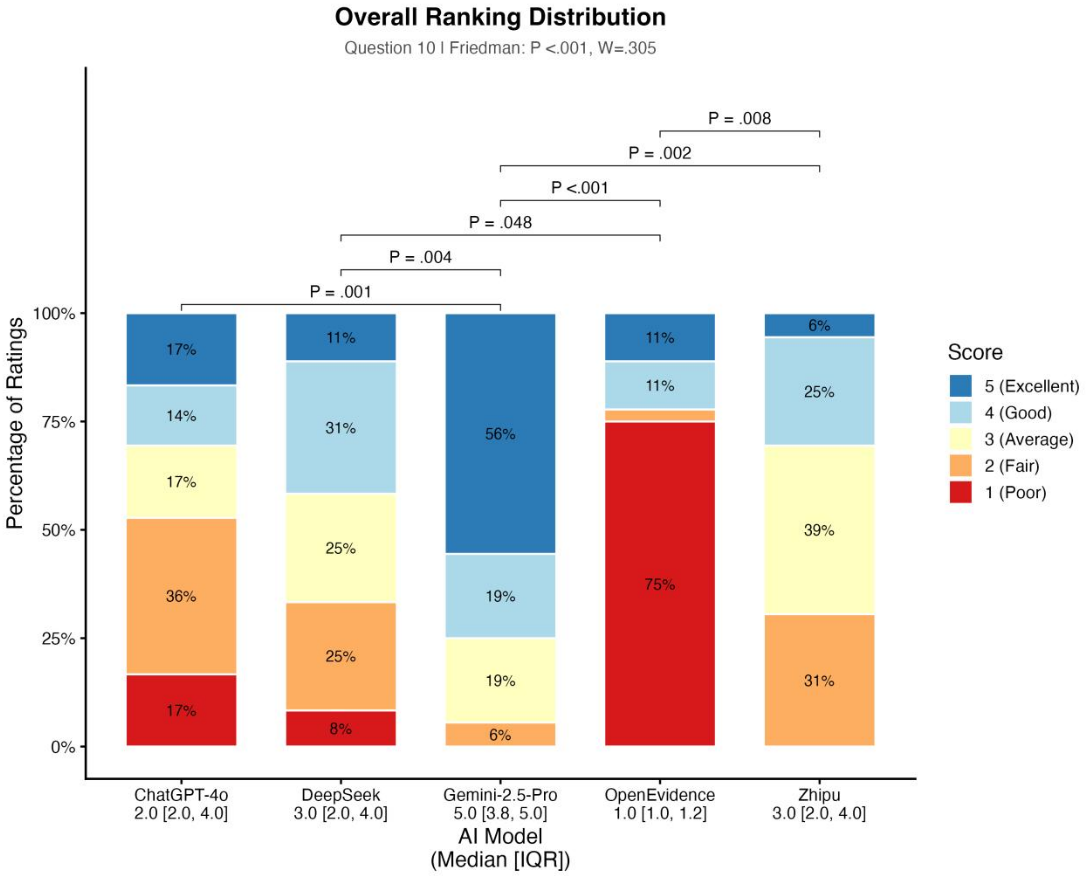
Overall Ranking Distribution
Question 10 | Friedman: P <.001, W=.305
P = .008
P = .002
P <.001
P = .048
P = .004
P = .001
Percentage of Ratings
100%
75%
50%
25%
0%
17%
14%
17%
36%
17%
11%
31%
25%
25%
8%
56%
19%
19%
6%
11%
11%
75%
6%
25%
39%
31%
Score
5 (Excellent)
4 (Good)
3 (Average)
2 (Fair)
1 (Poor)
ChatGPT-4o
2.0 [2.0, 4.0]
DeepSeek
3.0 [2.0, 4.0]
Gemini-2.5-Pro
5.0 [3.8, 5.0]
OpenEvidence
1.0 [1.0, 1.2]
Zhipu
3.0 [2.0, 4.0]
AI Model
(Median [IQR])

Violin (Rain-Cloud) Plots: Per-Question caregiver Rating Distributions

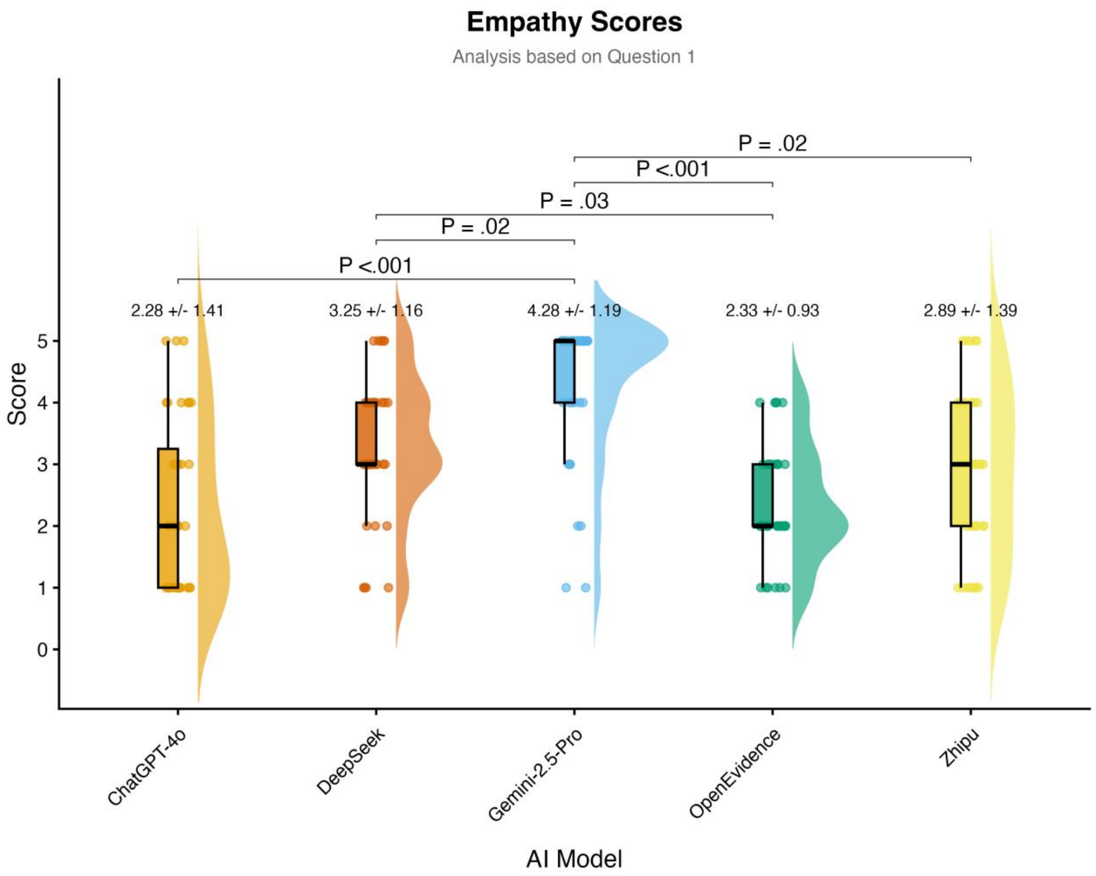

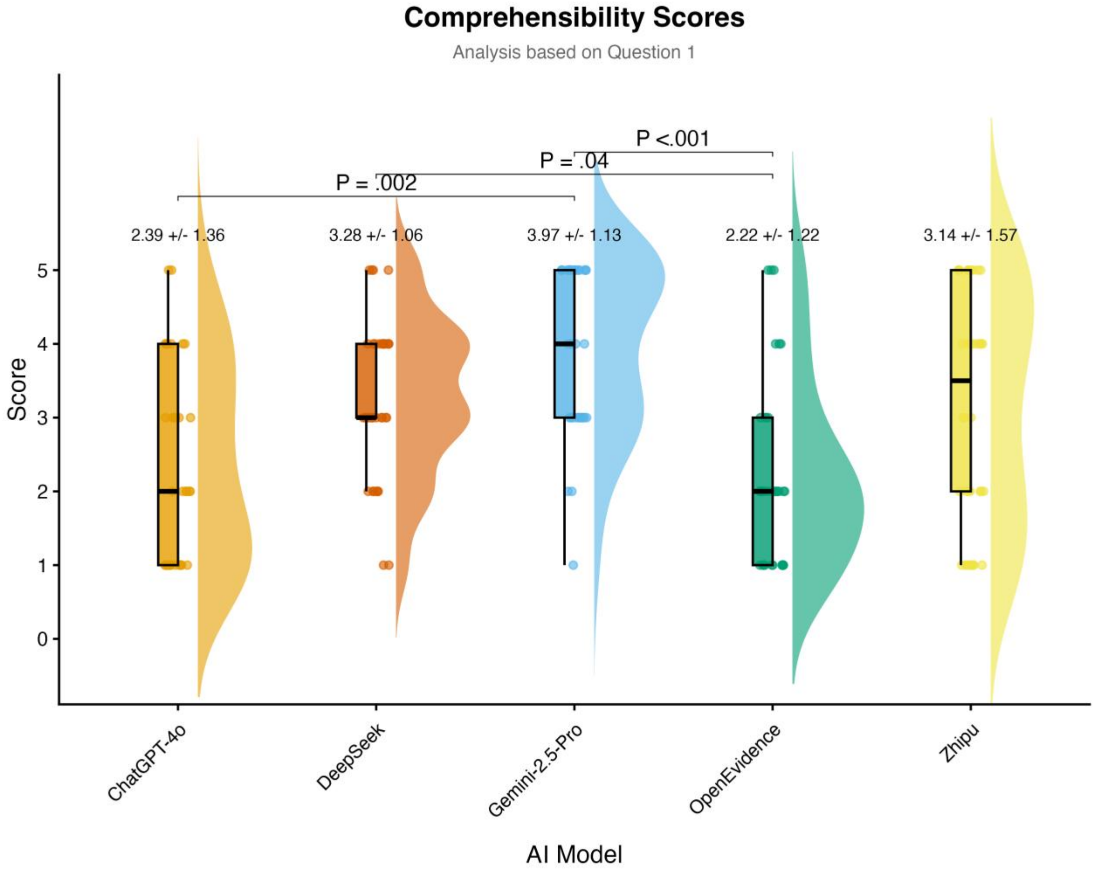

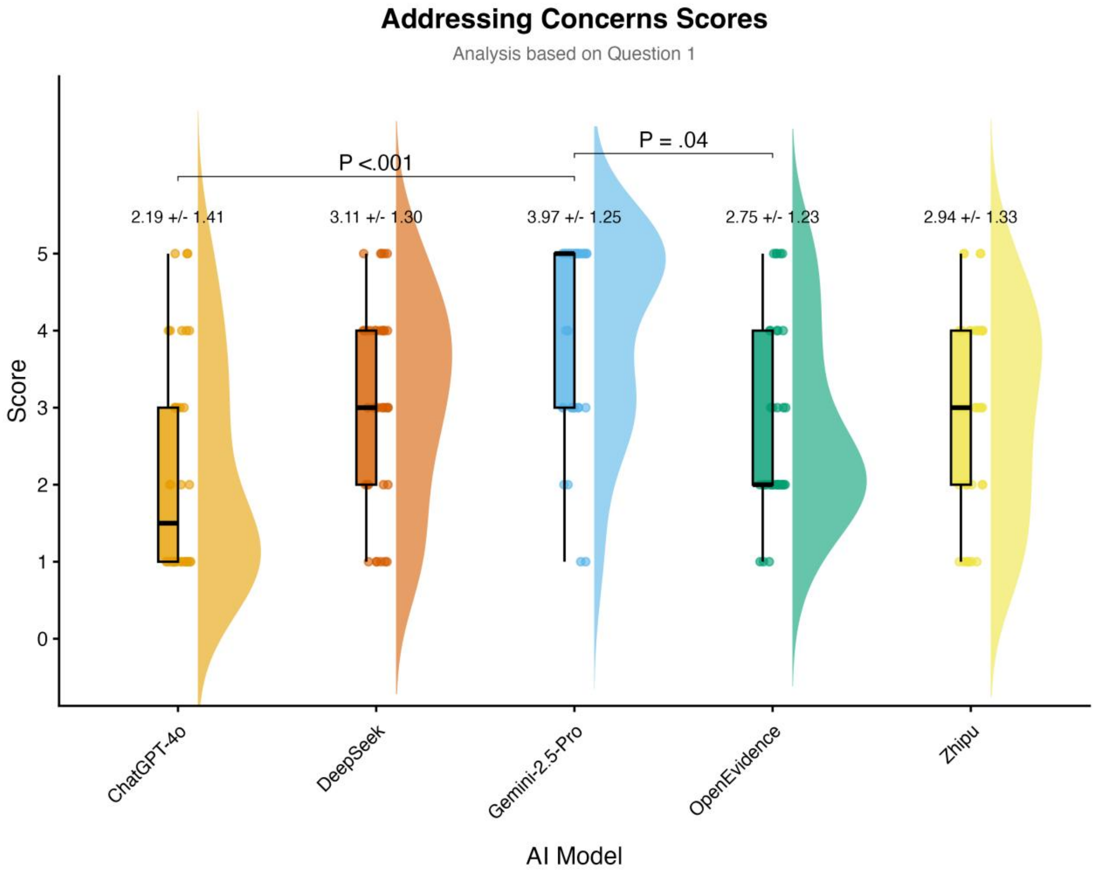
Addressing Concerns Scores
Analysis based on Question 1
P <.001
P = .04
2.19 +/- 1.41
3.11 +/- 1.30
3.97 +/- 1.25
2.75 +/- 1.23
2.94 +/- 1.33
Score
0
1
2
3
4
5
ChatGPT-4o
DeepSeek
Gemini-2.5-Pro
OpenEvidence
Zhipu
AI Model

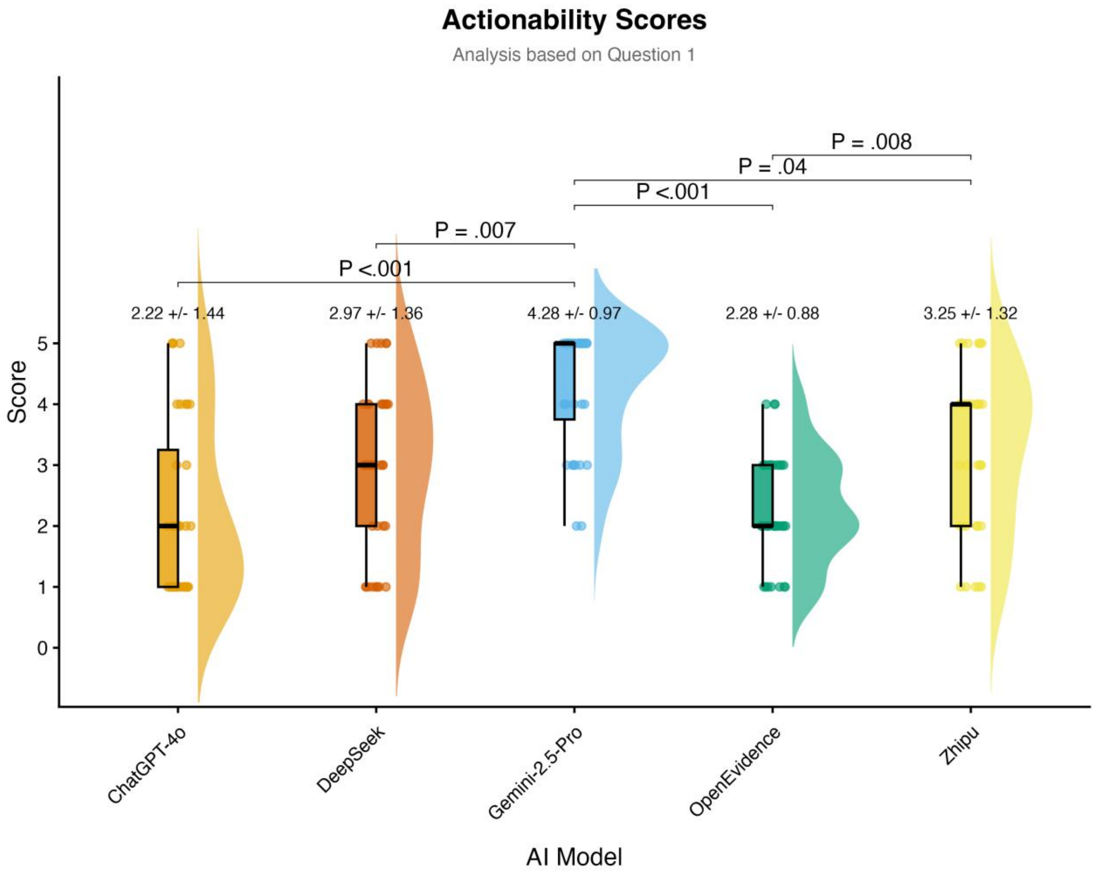
Actionability Scores
Analysis based on Question 1
P = .008
P = .04
P <.001
P = .007
P <.001
2.22 +/- 1.44
2.97 +/- 1.36
4.28 +/- 0.97
2.28 +/- 0.88
3.25 +/- 1.32
Score
0
1
2
3
4
5
ChatGPT-4o
DeepSeek
Gemini-2.5-Pro
OpenEvidence
Zhipu
AI Model

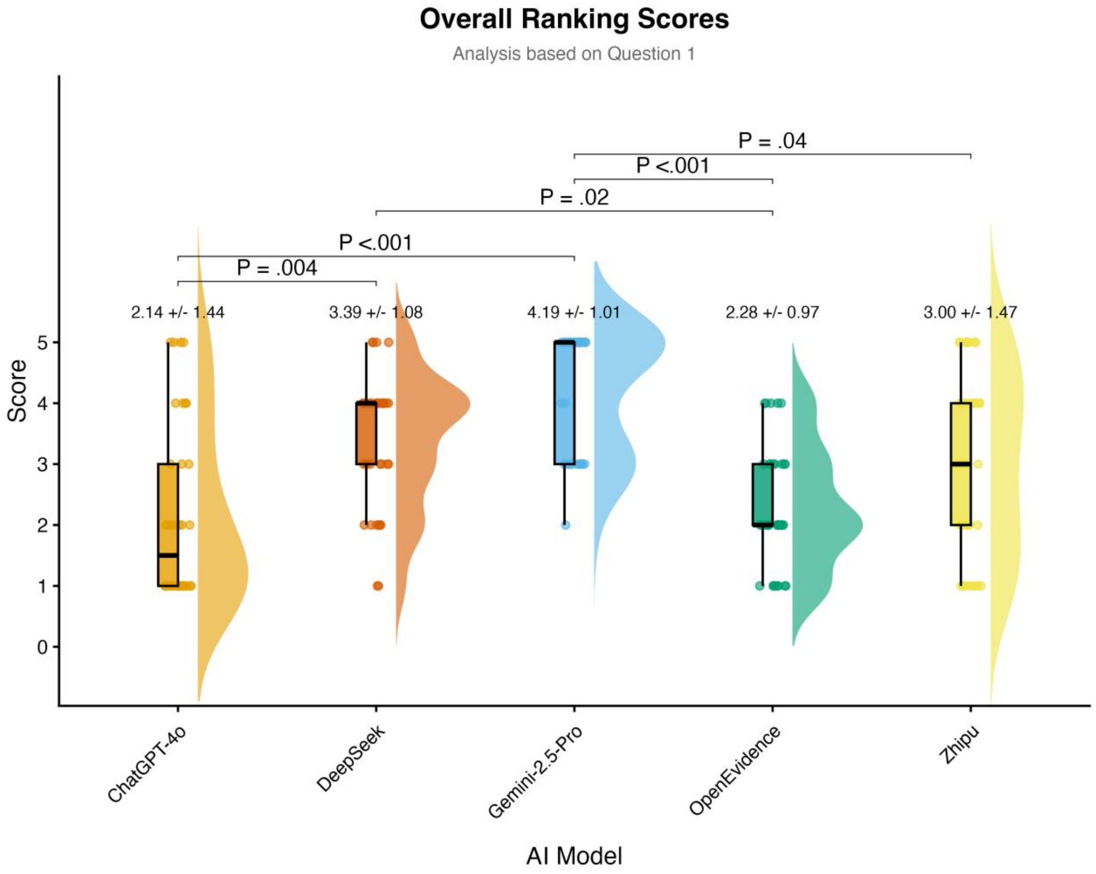
Overall Ranking Scores
Analysis based on Question 1
P = .04
P <.001
P = .02
P <.001
P = .004
2.14 +/- 1.44
3.39 +/- 1.08
4.19 +/- 1.01
2.28 +/- 0.97
3.00 +/- 1.47
Score
0
1
2
3
4
5
ChatGPT-4o
DeepSeek
Gemini-2.5-Pro
OpenEvidence
Zhipu
AI Model

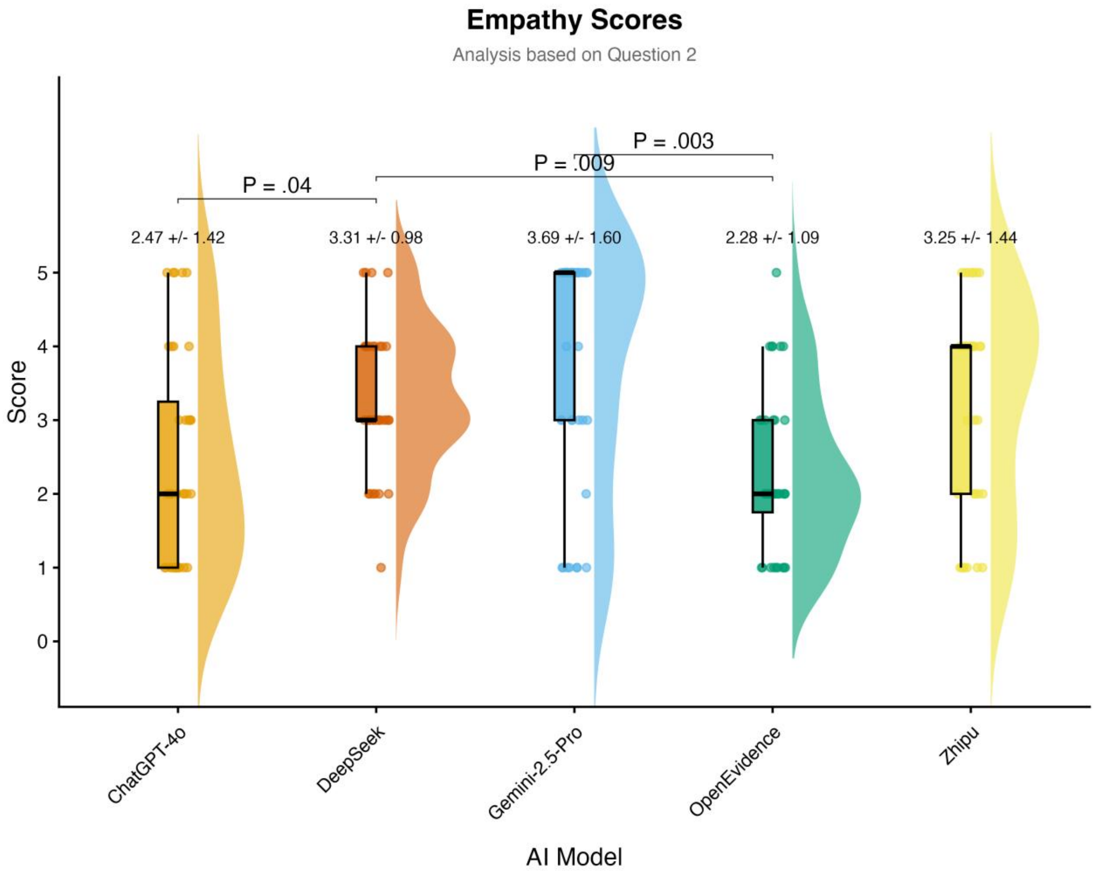
Empathy Scores
Analysis based on Question 2
P = .003
P = .009
P = .04
2.47 +/- 1.42
3.31 +/- 0.98
3.69 +/- 1.60
2.28 +/- 1.09
3.25 +/- 1.44
Score
0
1
2
3
4
5
ChatGPT-4o
DeepSeek
Gemini-2.5-Pro
OpenEvidence
Zhipu
AI Model

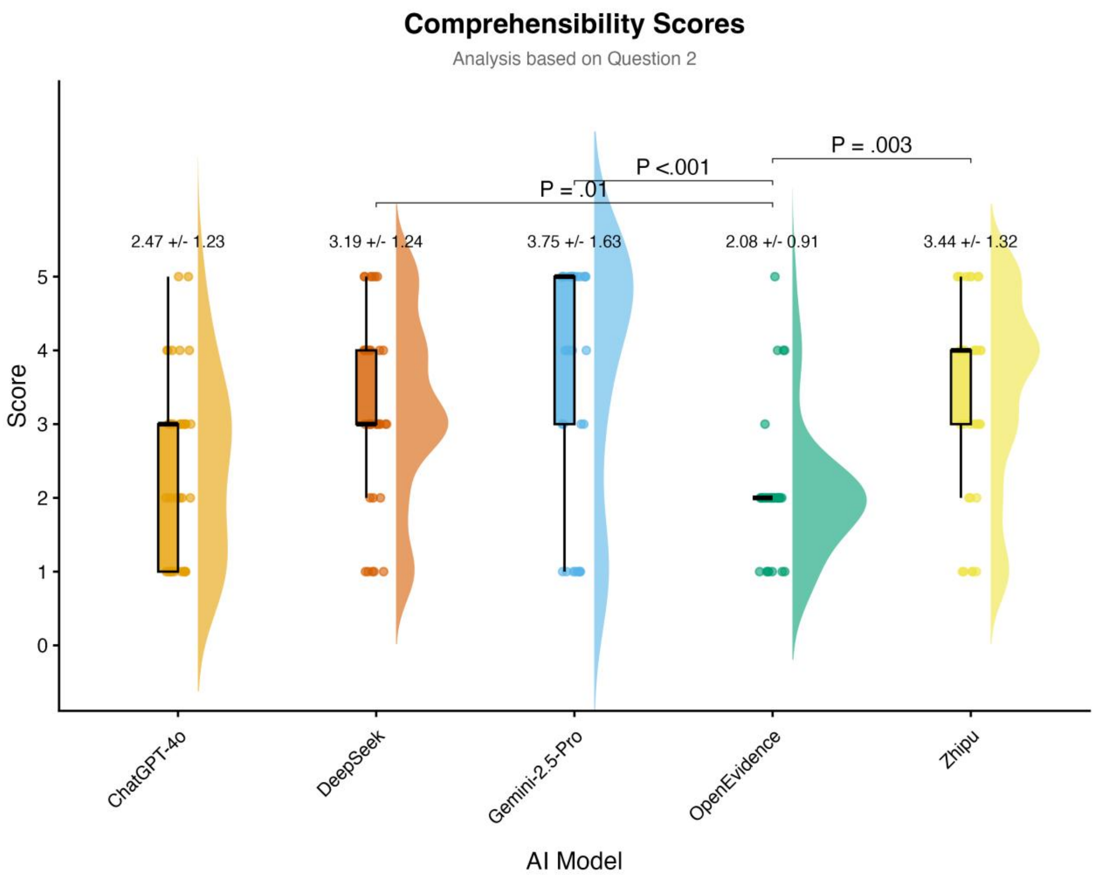
Comprehensibility Scores
Analysis based on Question 2
P = .003
P <.001
P = .01
2.47 +/- 1.23
3.19 +/- 1.24
3.75 +/- 1.63
2.08 +/- 0.91
3.44 +/- 1.32
Score
AI Model
ChatGPT-4o
DeepSeek
Gemini-2.5-Pro
OpenEvidence
Zhipu

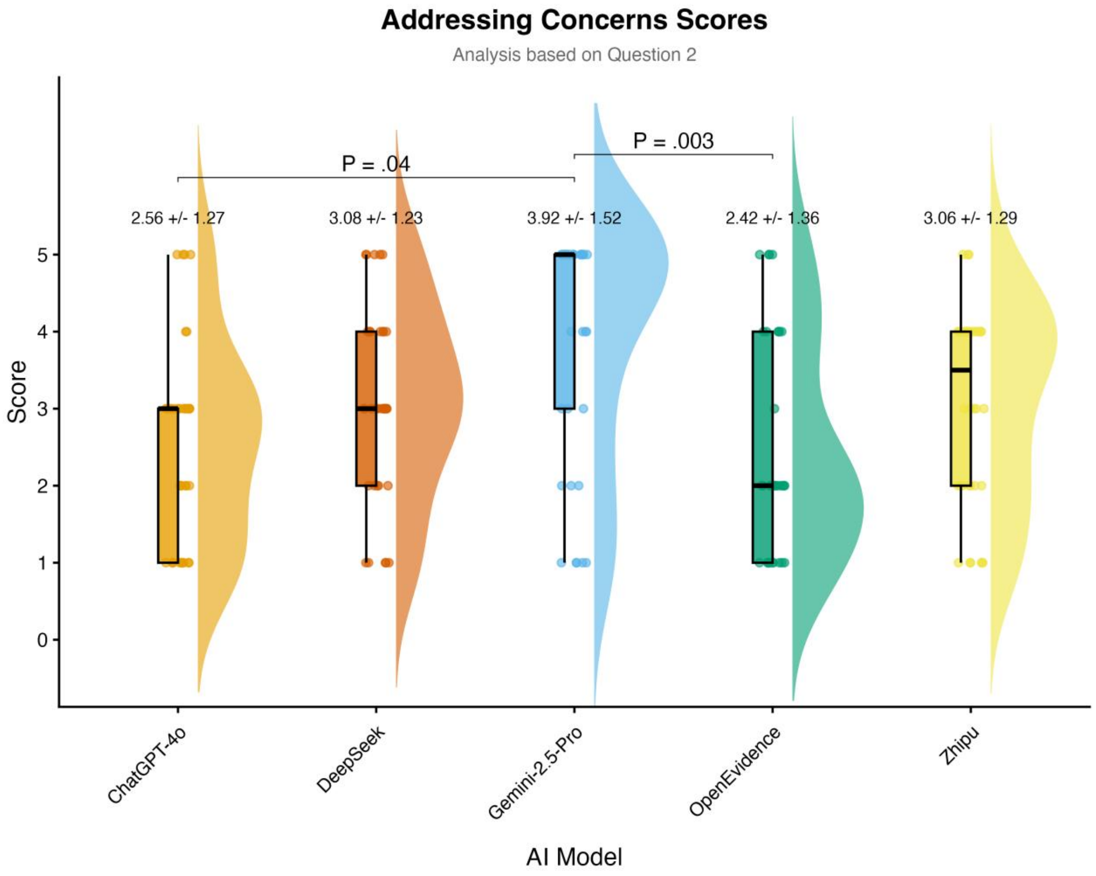
Addressing Concerns Scores
Analysis based on Question 2
P = .003
P = .04
2.56 +/- 1.27
3.08 +/- 1.23
3.92 +/- 1.52
2.42 +/- 1.36
3.06 +/- 1.29
Score
AI Model
ChatGPT-4o
DeepSeek
Gemini-2.5-Pro
OpenEvidence
Zhipu

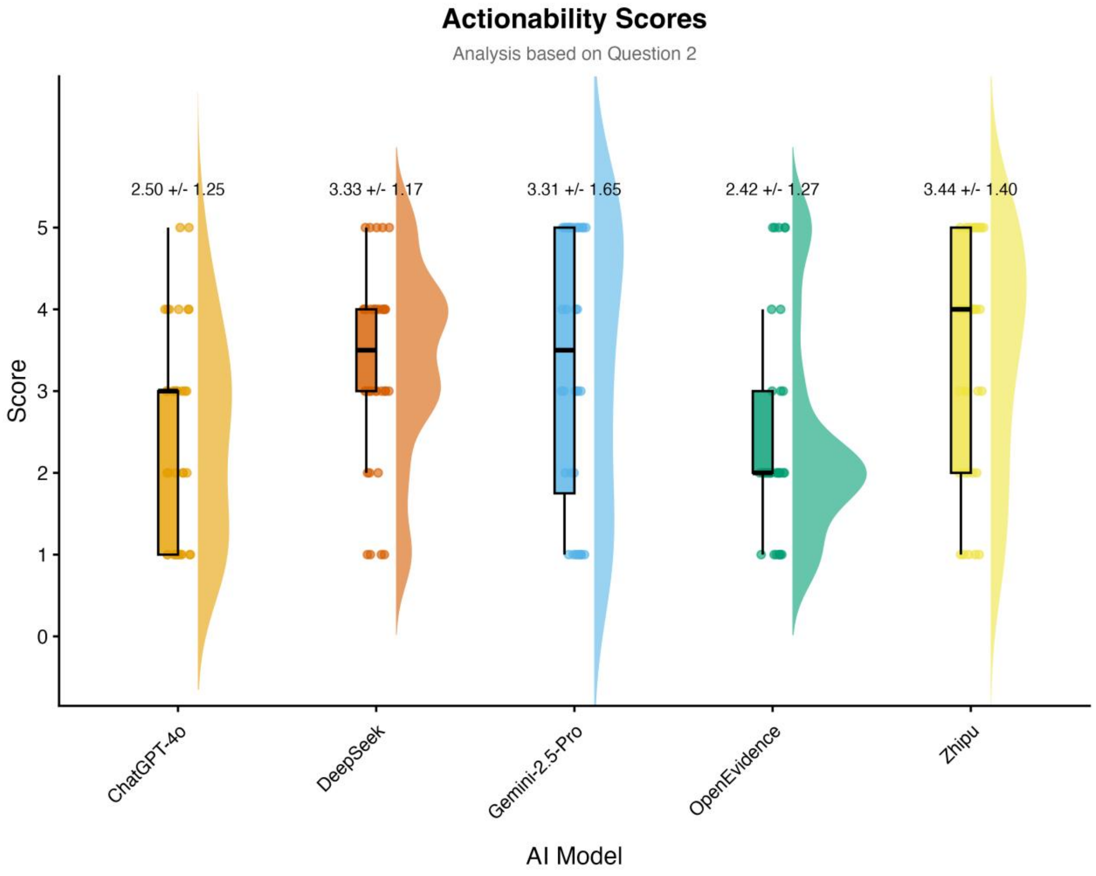
Actionability Scores
Analysis based on Question 2
Score
2.50 +/- 1.25
3.33 +/- 1.17
3.31 +/- 1.65
2.42 +/- 1.27
3.44 +/- 1.40
ChatGPT-4o
DeepSeek
Gemini-2.5-Pro
OpenEvidence
Zhipu
AI Model

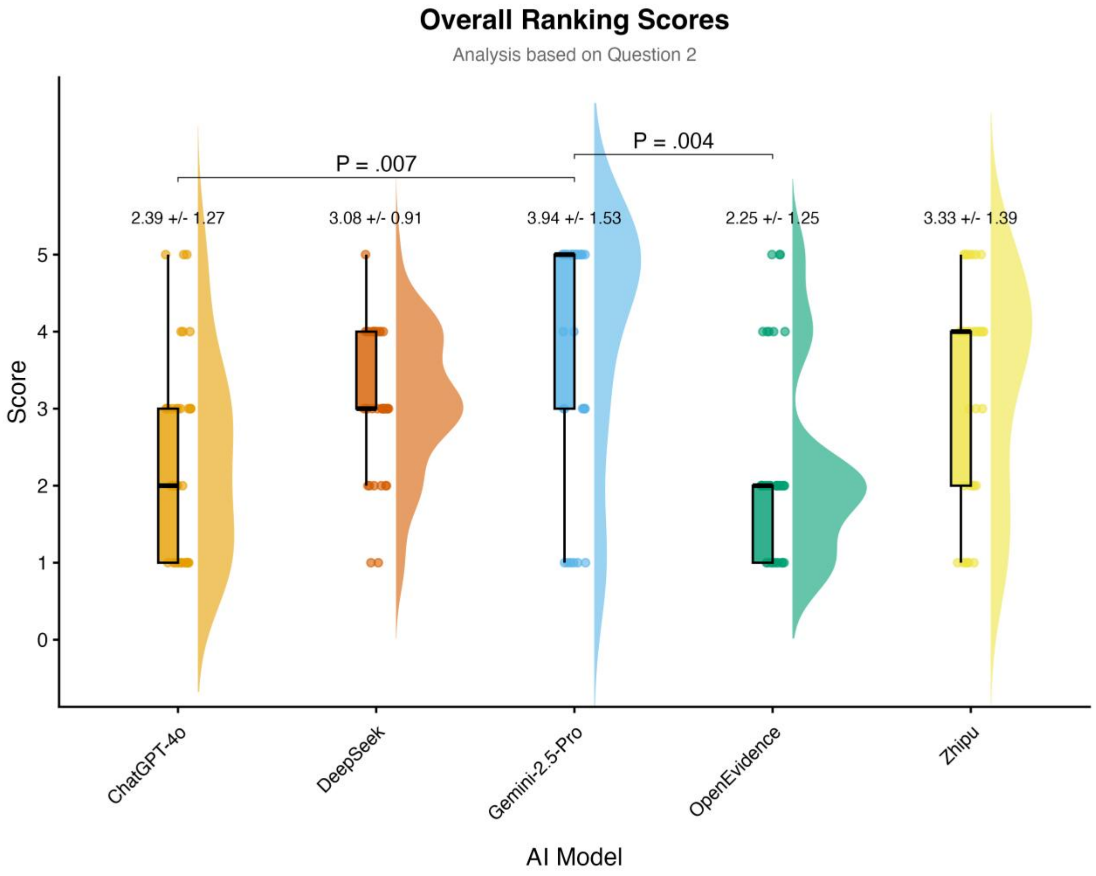
Overall Ranking Scores
Analysis based on Question 2
Score
P = .007
P = .004
2.39 +/- 1.27
3.08 +/- 0.91
3.94 +/- 1.53
2.25 +/- 1.25
3.33 +/- 1.39
ChatGPT-4o
DeepSeek
Gemini-2.5-Pro
OpenEvidence
Zhipu
AI Model

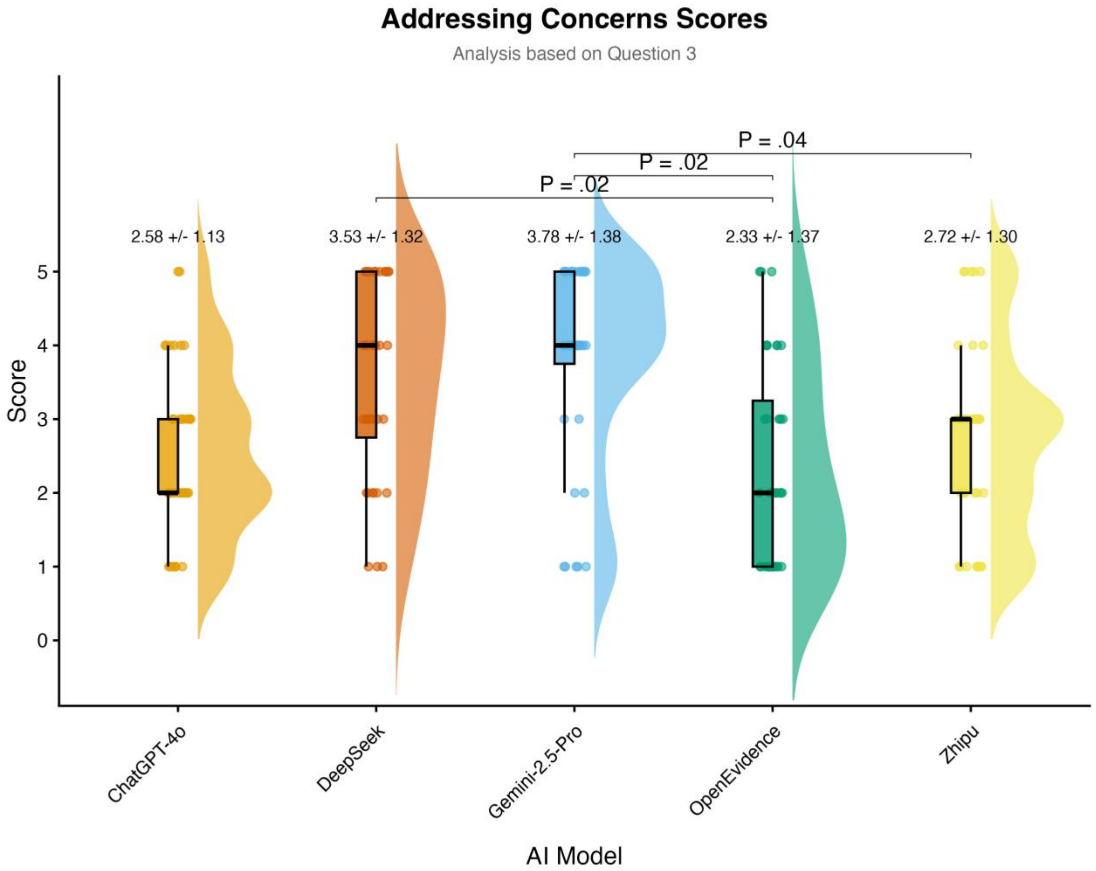
Addressing Concerns Scores
Analysis based on Question 3
P = .04
P = .02
P = .02
2.58 +/- 1.13
3.53 +/- 1.32
3.78 +/- 1.38
2.33 +/- 1.37
2.72 +/- 1.30
Score
0
1
2
3
4
5
ChatGPT-4o
DeepSeek
Gemini-2.5-Pro
OpenEvidence
Zhipu
AI Model

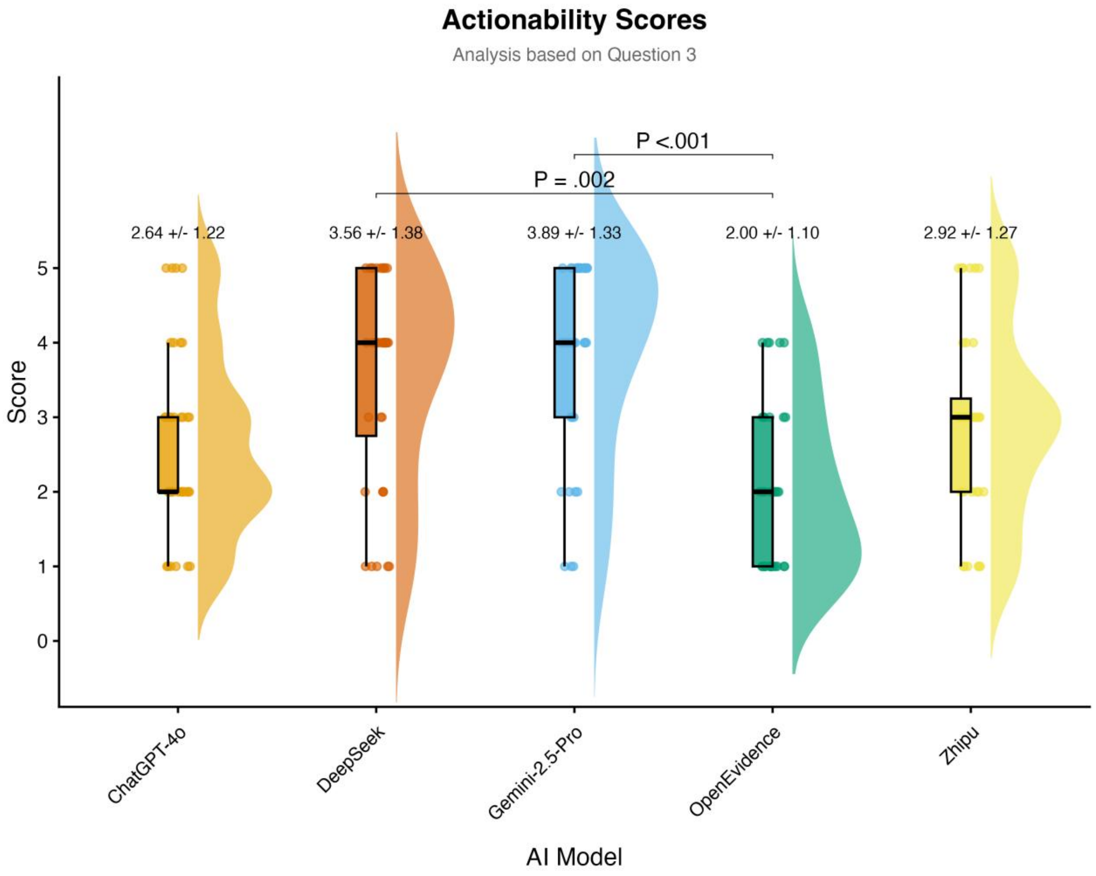
Actionability Scores
Analysis based on Question 3
P <.001
P = .002
2.64 +/- 1.22
3.56 +/- 1.38
3.89 +/- 1.33
2.00 +/- 1.10
2.92 +/- 1.27
Score
0
1
2
3
4
5
ChatGPT-4o
DeepSeek
Gemini-2.5-Pro
OpenEvidence
Zhipu
AI Model

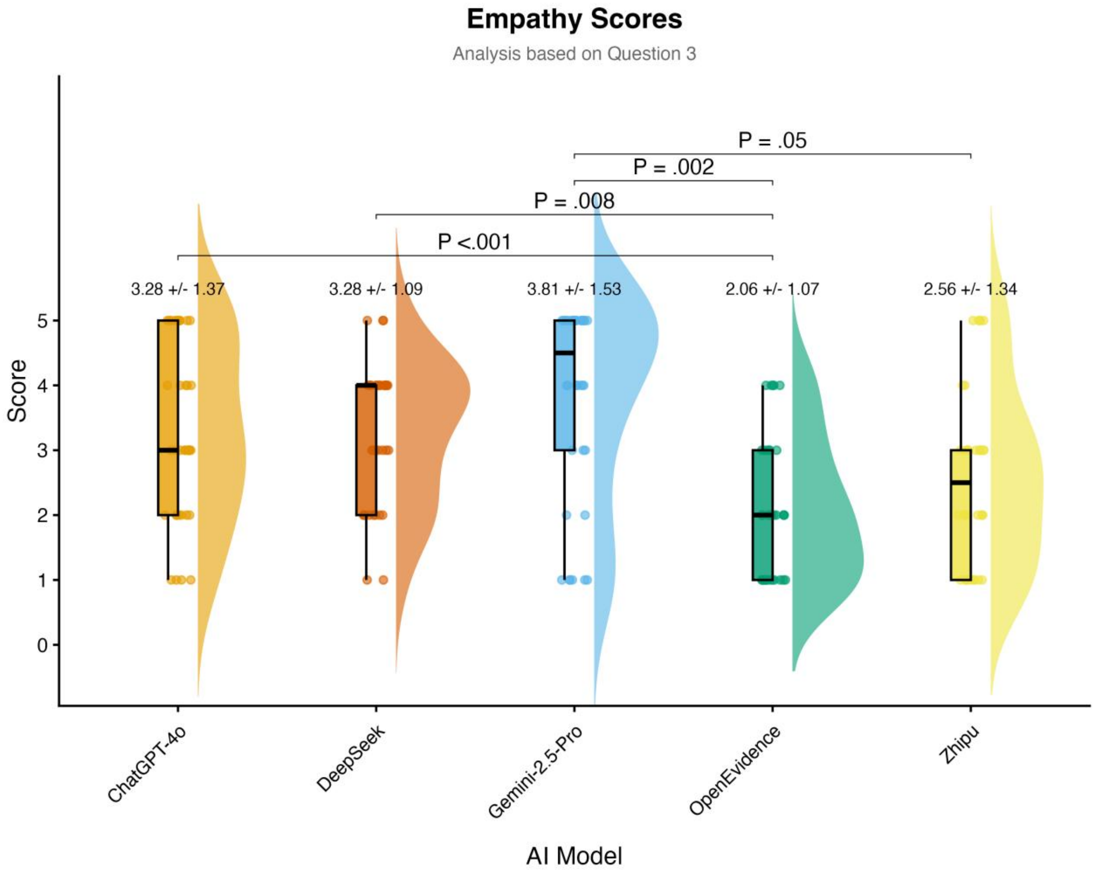

Empathy Scores
Analysis based on Question 3
P = .05
P = .002
P = .008
P <.001
3.28 +/- 1.37
3.28 +/- 1.09
3.81 +/- 1.53
2.06 +/- 1.07
2.56 +/- 1.34
Score
0
1
2
3
4
5
ChatGPT-4o
DeepSeek
Gemini-2.5-Pro
OpenEvidence
Zhipu
AI Model

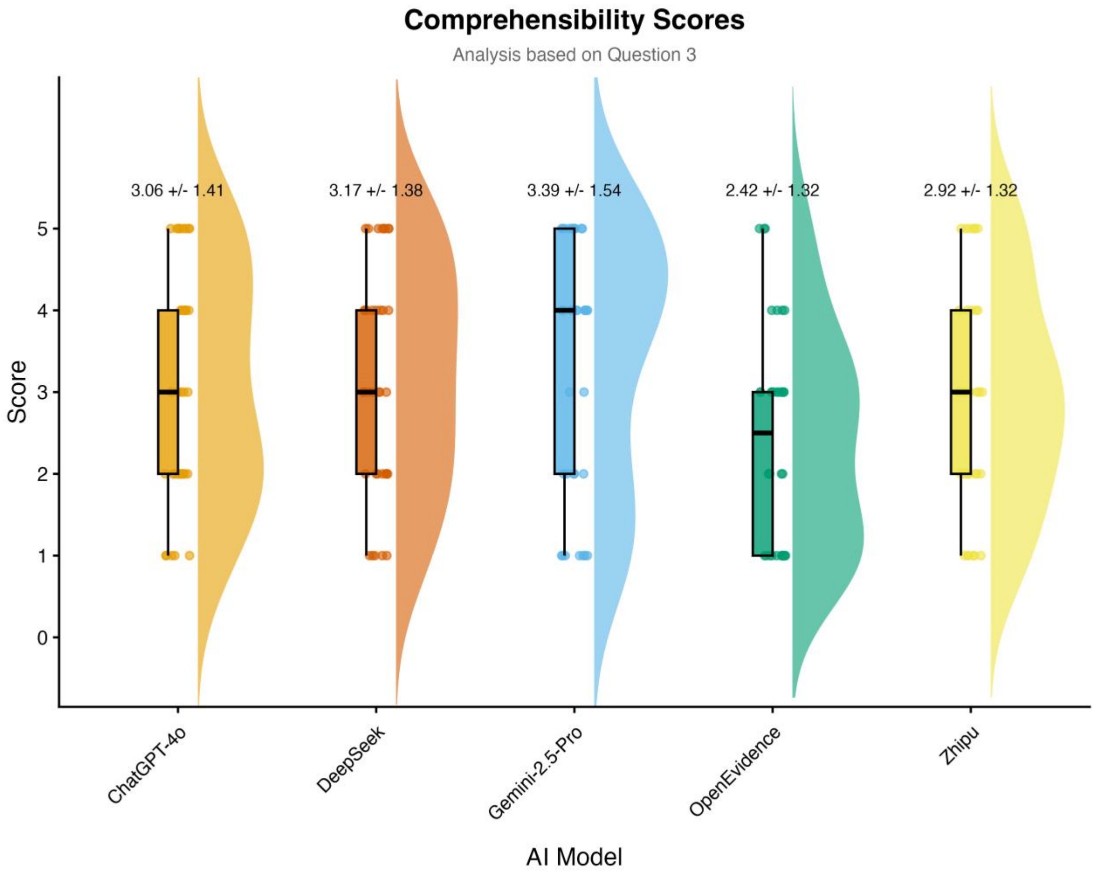

Comprehensibility Scores
Analysis based on Question 3
3.06 +/- 1.41
3.17 +/- 1.38
3.39 +/- 1.54
2.42 +/- 1.32
2.92 +/- 1.32
Score
0
1
2
3
4
5
ChatGPT-4o
DeepSeek
Gemini-2.5-Pro
OpenEvidence
Zhipu
AI Model

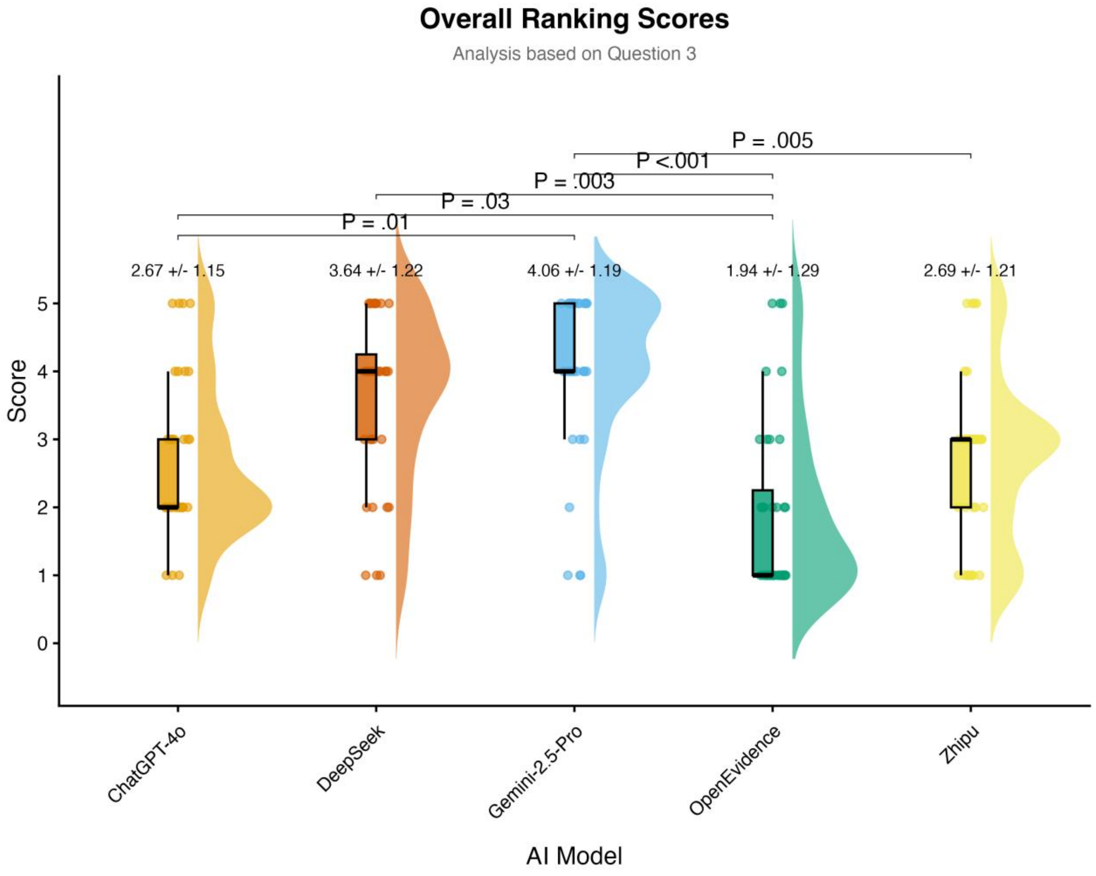
Overall Ranking Scores
Analysis based on Question 3
P = .005
P <.001
P = .003
P = .03
P = .01
2.67 +/- 1.15
3.64 +/- 1.22
4.06 +/- 1.19
1.94 +/- 1.29
2.69 +/- 1.21
Score
5
4
3
2
1
0
ChatGPT-4o
DeepSeek
Gemini-2.5-Pro
OpenEvidence
Zhipu
AI Model

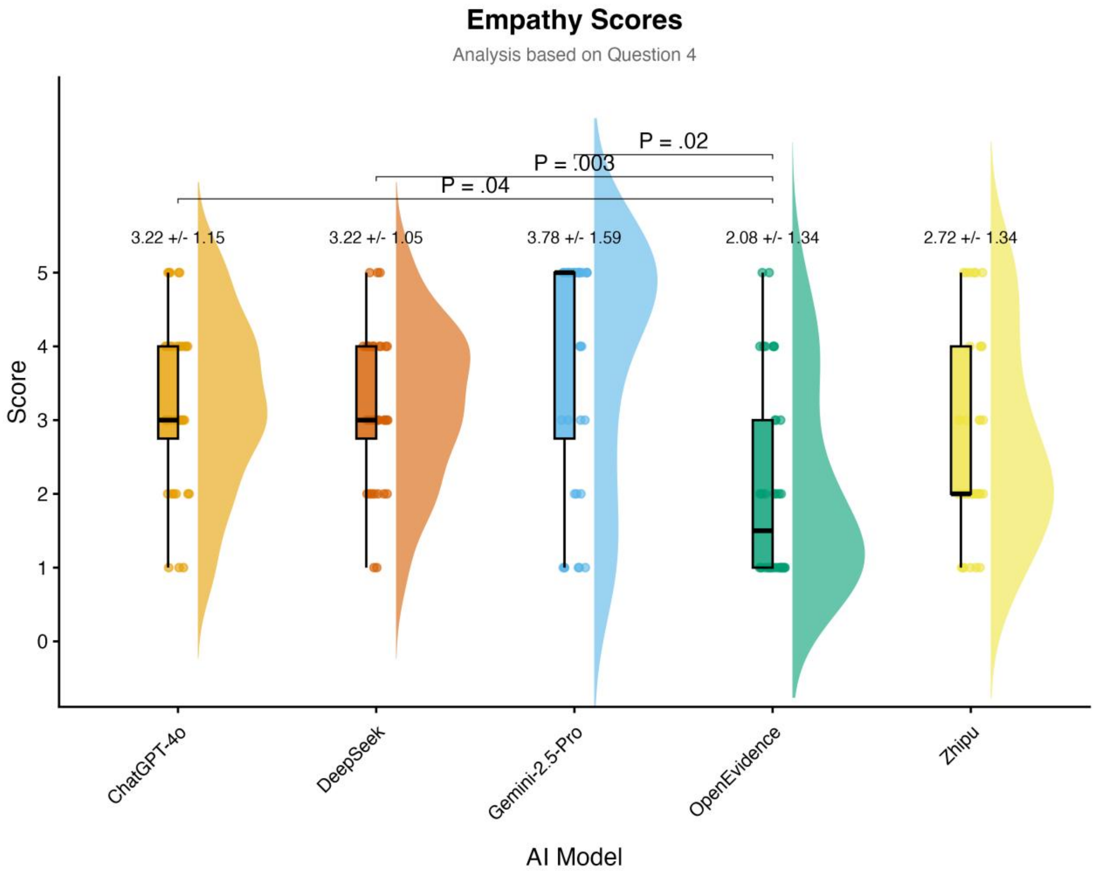
Empathy Scores
Analysis based on Question 4
P = .02
P = .003
P = .04
3.22 +/- 1.15
3.22 +/- 1.05
3.78 +/- 1.59
2.08 +/- 1.34
2.72 +/- 1.34
Score
5
4
3
2
1
0
ChatGPT-4o
DeepSeek
Gemini-2.5-Pro
OpenEvidence
Zhipu
AI Model

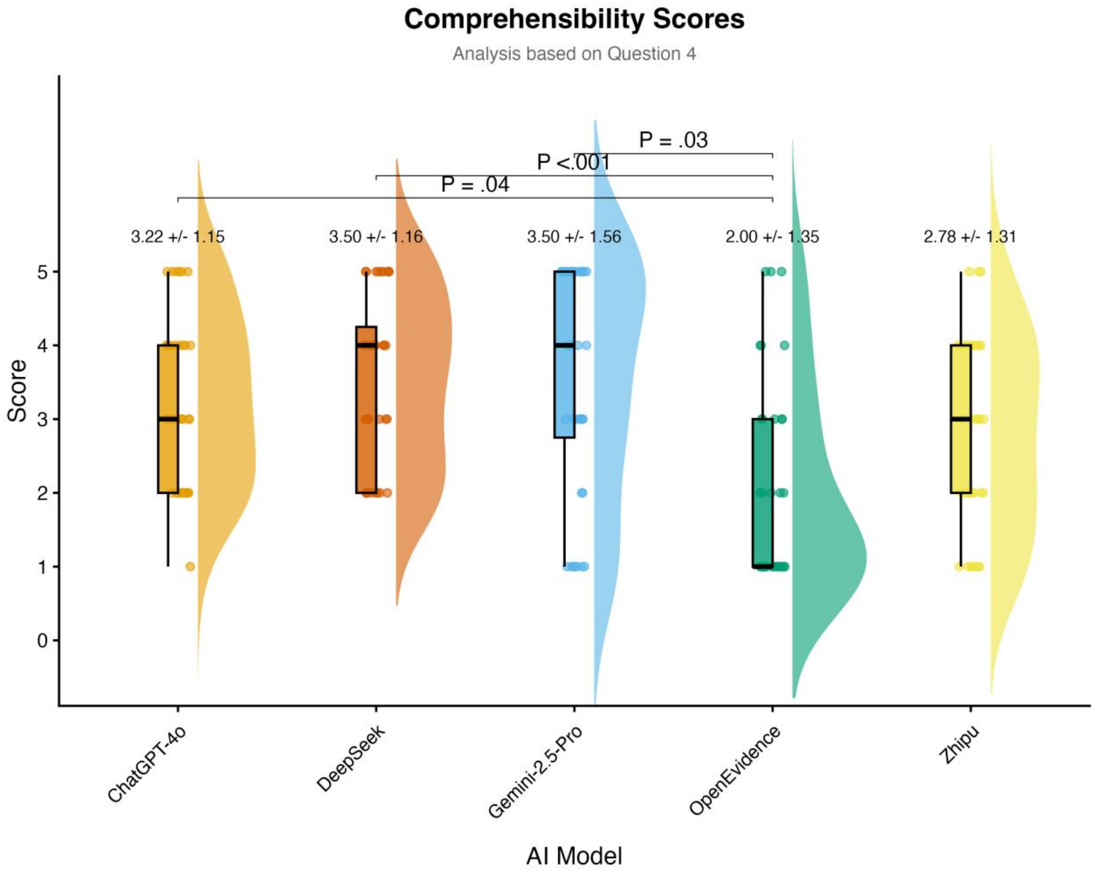
Comprehensibility Scores
Analysis based on Question 4
P = .03
P <.001
P = .04
3.22 +/- 1.15
3.50 +/- 1.16
3.50 +/- 1.56
2.00 +/- 1.35
2.78 +/- 1.31
Score
AI Model
ChatGPT-4o
DeepSeek
Gemini-2.5-Pro
OpenEvidence
Zhipu

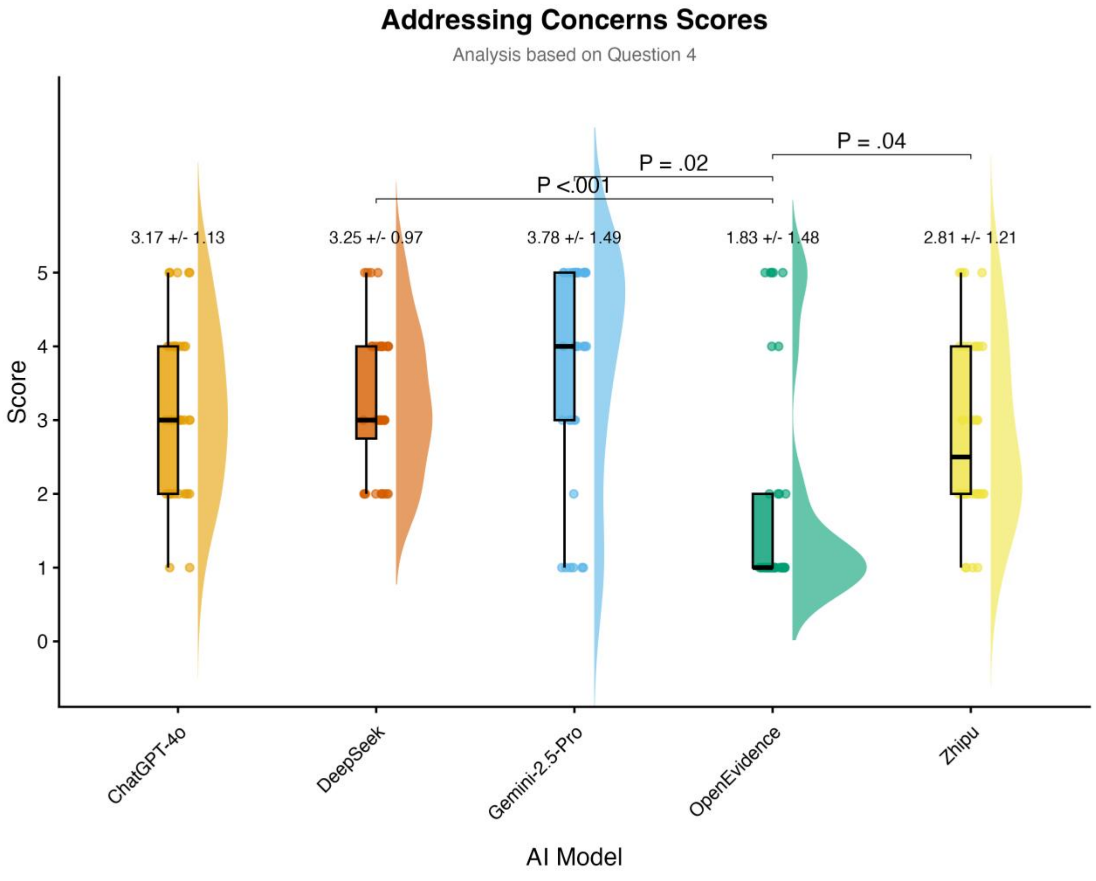
Addressing Concerns Scores
Analysis based on Question 4
P = .04
P = .02
P <.001
3.17 +/- 1.13
3.25 +/- 0.97
3.78 +/- 1.49
1.83 +/- 1.48
2.81 +/- 1.21
Score
AI Model
ChatGPT-4o
DeepSeek
Gemini-2.5-Pro
OpenEvidence
Zhipu

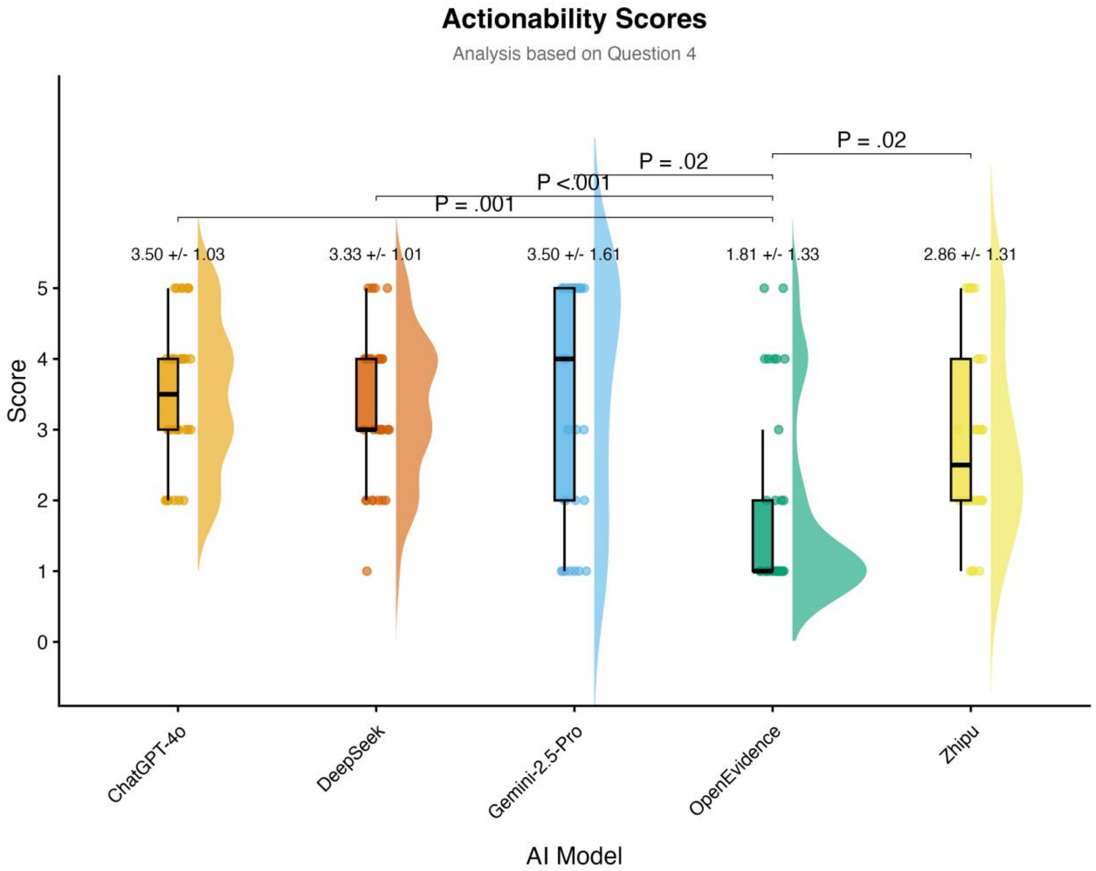
Actionability Scores
Analysis based on Question 4
P = .02
P = .02
P < .001
P = .001
3.50 +/- 1.03
3.33 +/- 1.01
3.50 +/- 1.61
1.81 +/- 1.33
2.86 +/- 1.31
Score
5
4
3
2
1
0
ChatGPT-4o
DeepSeek
Gemini-2.5-Pro
OpenEvidence
Zhipu
AI Model

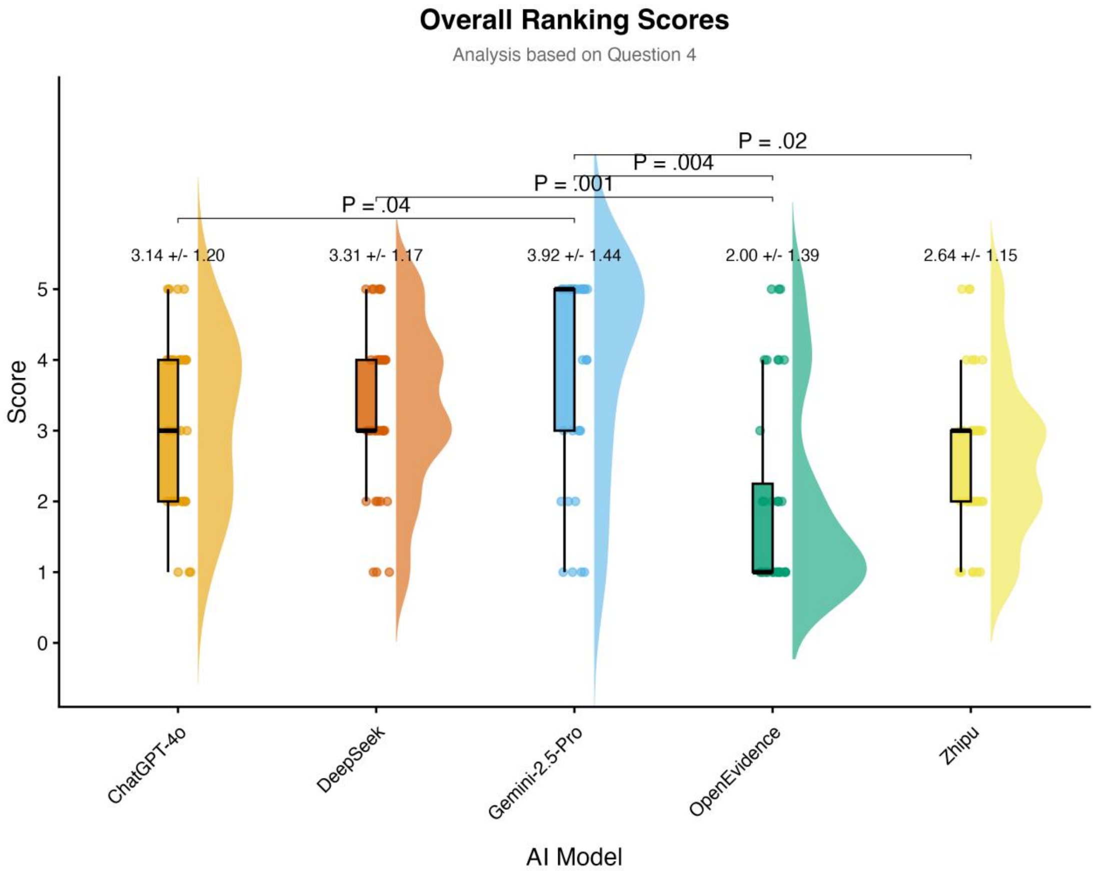
Overall Ranking Scores
Analysis based on Question 4
P = .02
P = .004
P = .001
P = .04
3.14 +/- 1.20
3.31 +/- 1.17
3.92 +/- 1.44
2.00 +/- 1.39
2.64 +/- 1.15
Score
5
4
3
2
1
0
ChatGPT-4o
DeepSeek
Gemini-2.5-Pro
OpenEvidence
Zhipu
AI Model

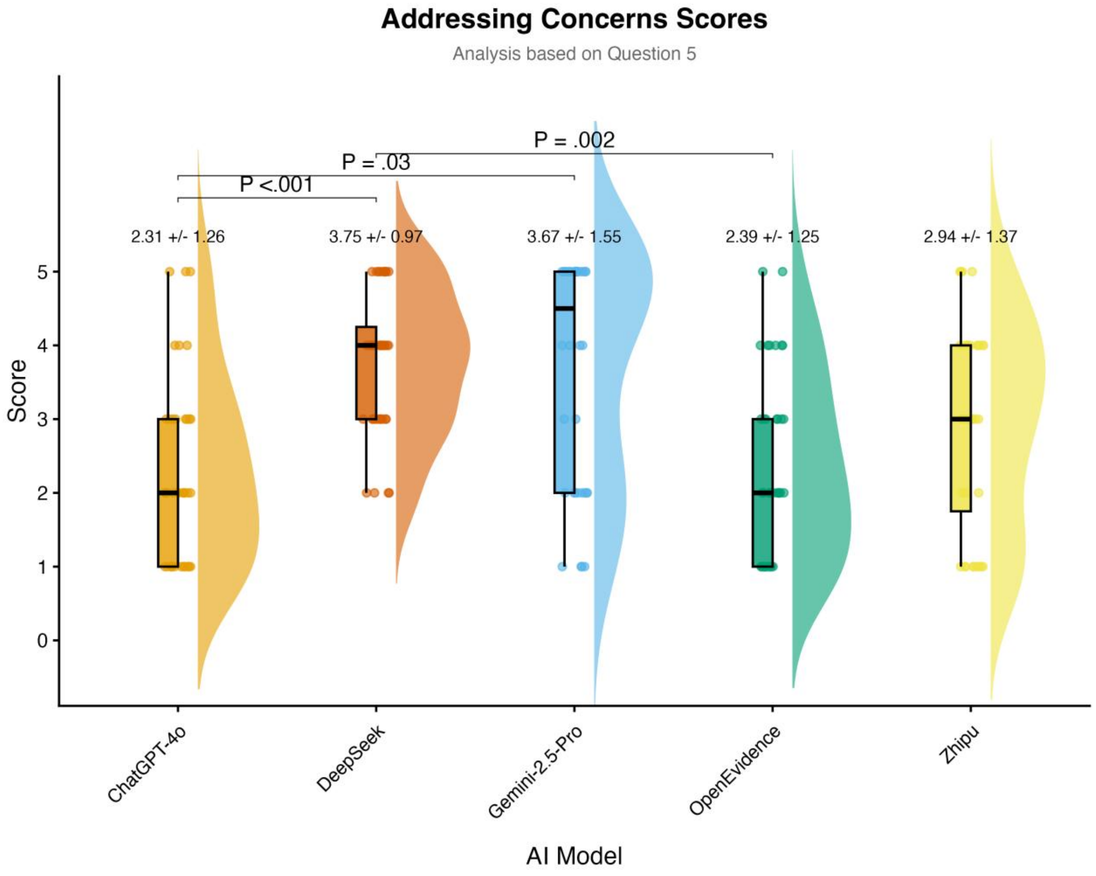

Addressing Concerns Scores
Analysis based on Question 5
P = .002
P = .03
P <.001
2.31 +/- 1.26
3.75 +/- 0.97
3.67 +/- 1.55
2.39 +/- 1.25
2.94 +/- 1.37
Score
ChatGPT-4o
DeepSeek
Gemini-2.5-Pro
OpenEvidence
Zhipu
AI Model

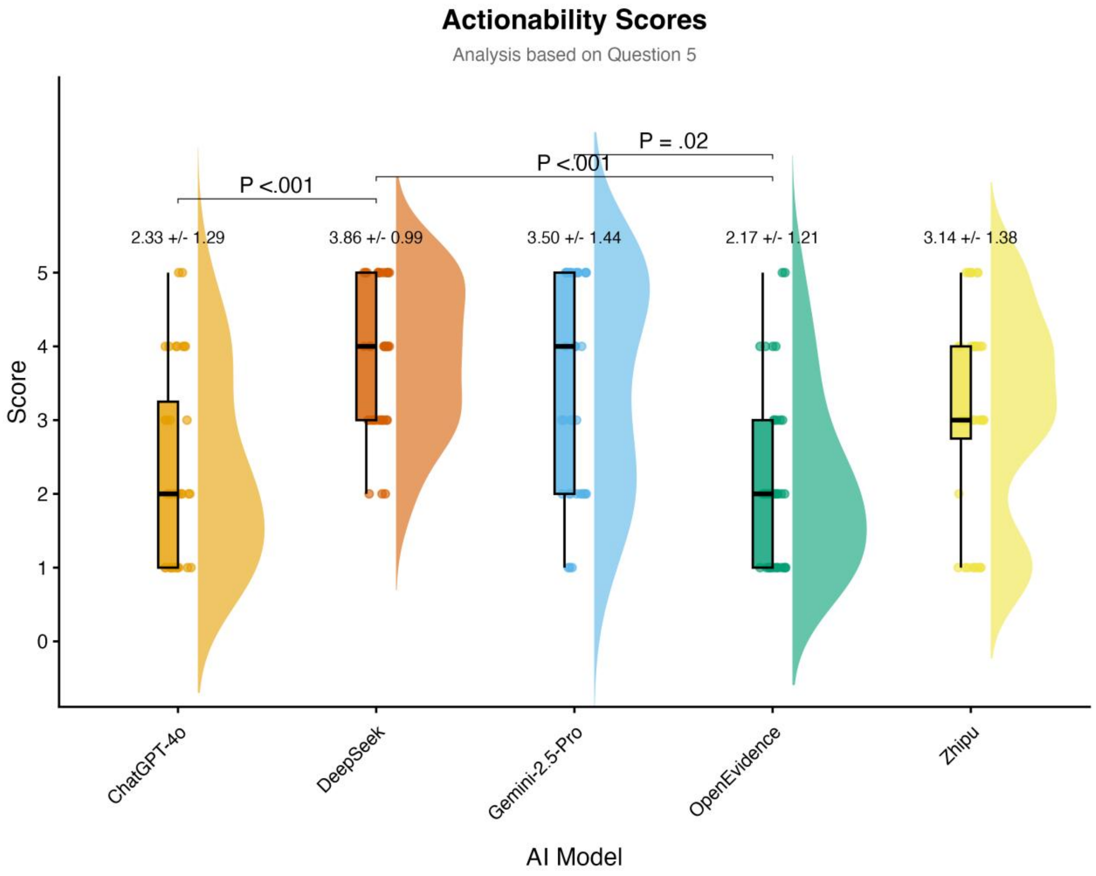

Actionability Scores
Analysis based on Question 5
P = .02
P <.001
P <.001
2.33 +/- 1.29
3.86 +/- 0.99
3.50 +/- 1.44
2.17 +/- 1.21
3.14 +/- 1.38
Score
ChatGPT-4o
DeepSeek
Gemini-2.5-Pro
OpenEvidence
Zhipu
AI Model

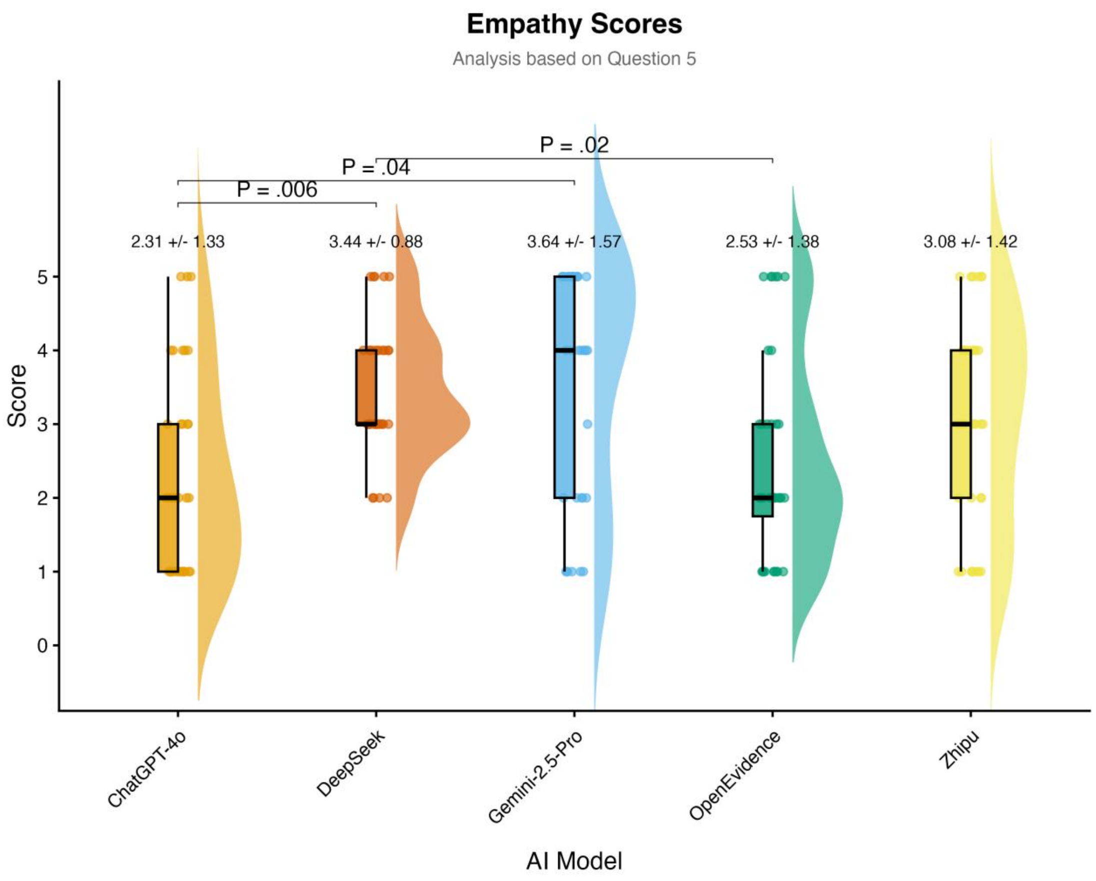
Empathy Scores
Analysis based on Question 5
P = .02
P = .04
P = .006
2.31 +/- 1.33
3.44 +/- 0.88
3.64 +/- 1.57
2.53 +/- 1.38
3.08 +/- 1.42
Score
5
4
3
2
1
0
ChatGPT-4o
DeepSeek
Gemini-2.5-Pro
OpenEvidence
Zhipu
AI Model

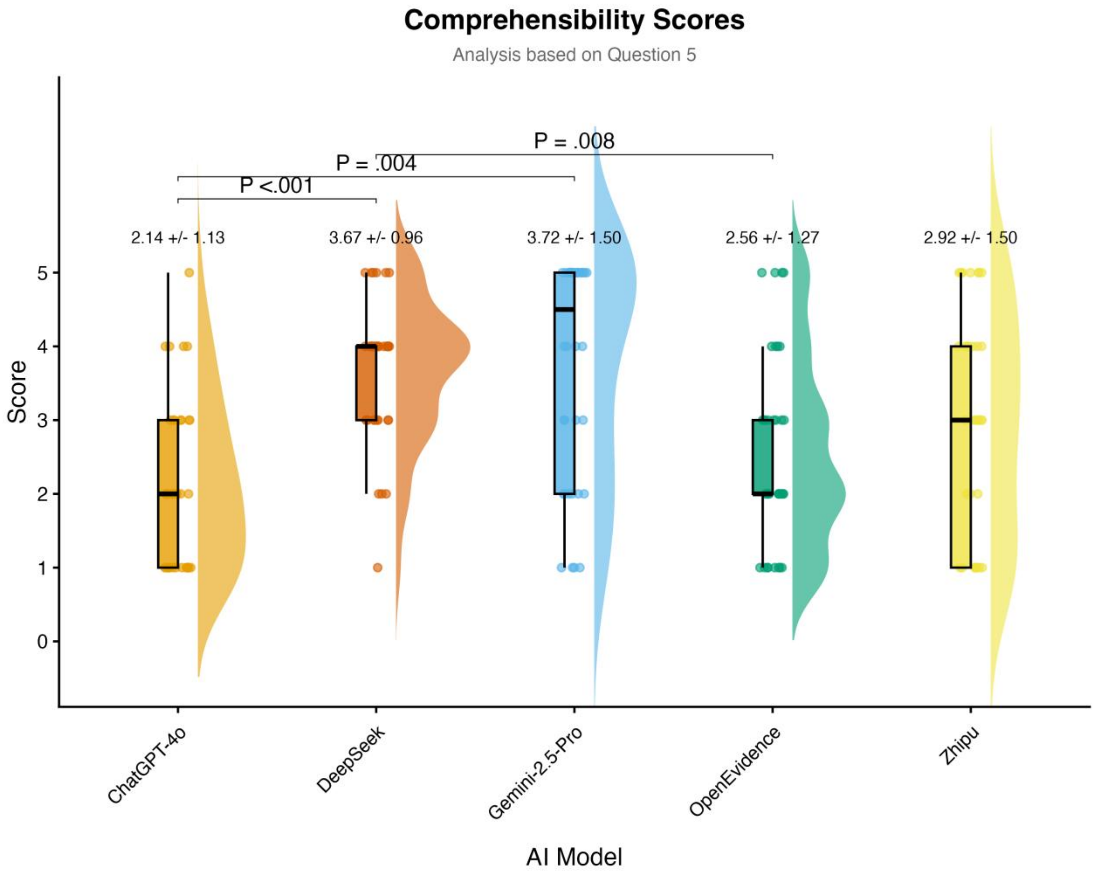
Comprehensibility Scores
Analysis based on Question 5
P = .008
P = .004
P <.001
2.14 +/- 1.13
3.67 +/- 0.96
3.72 +/- 1.50
2.56 +/- 1.27
2.92 +/- 1.50
Score
5
4
3
2
1
0
ChatGPT-4o
DeepSeek
Gemini-2.5-Pro
OpenEvidence
Zhipu
AI Model

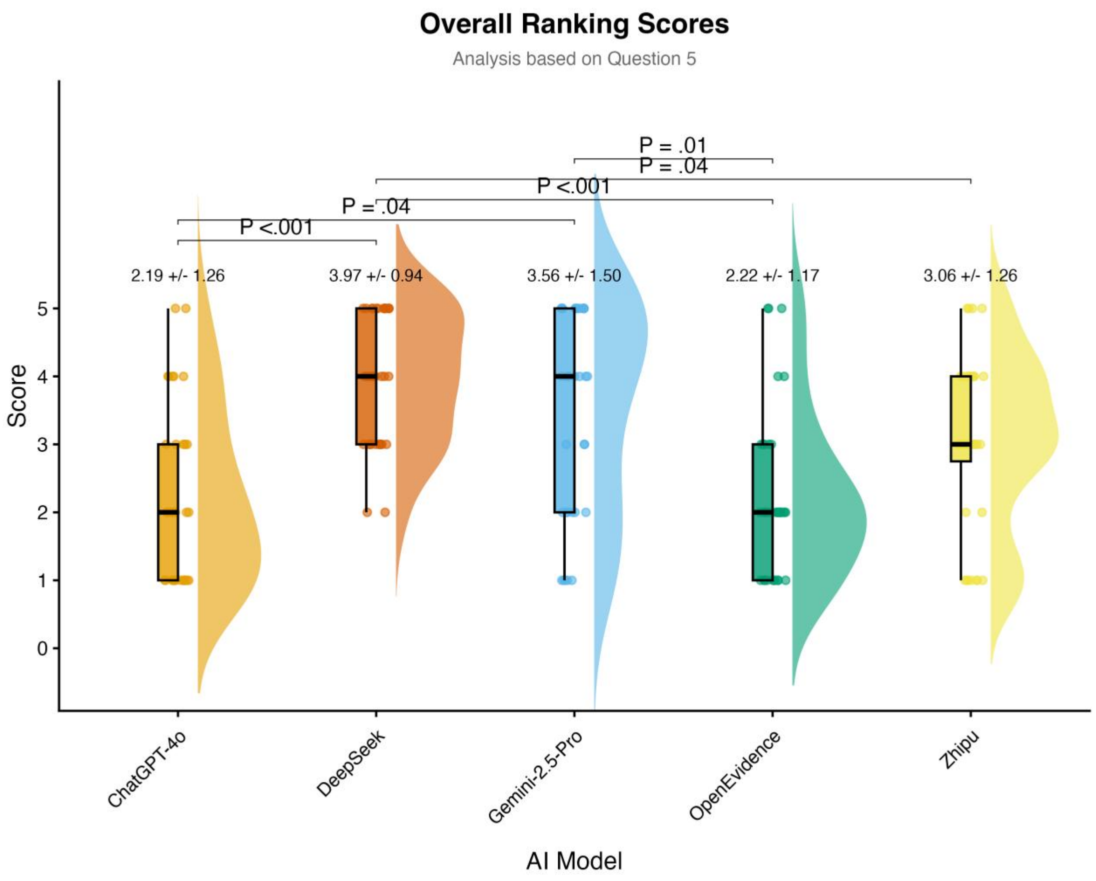
Overall Ranking Scores
Analysis based on Question 5
P = .01
P = .04
P <.001
P = .04
P <.001
2.19 +/- 1.26
3.97 +/- 0.94
3.56 +/- 1.50
2.22 +/- 1.17
3.06 +/- 1.26
Score
ChatGPT-4o
DeepSeek
Gemini-2.5-Pro
OpenEvidence
Zhipu
AI Model

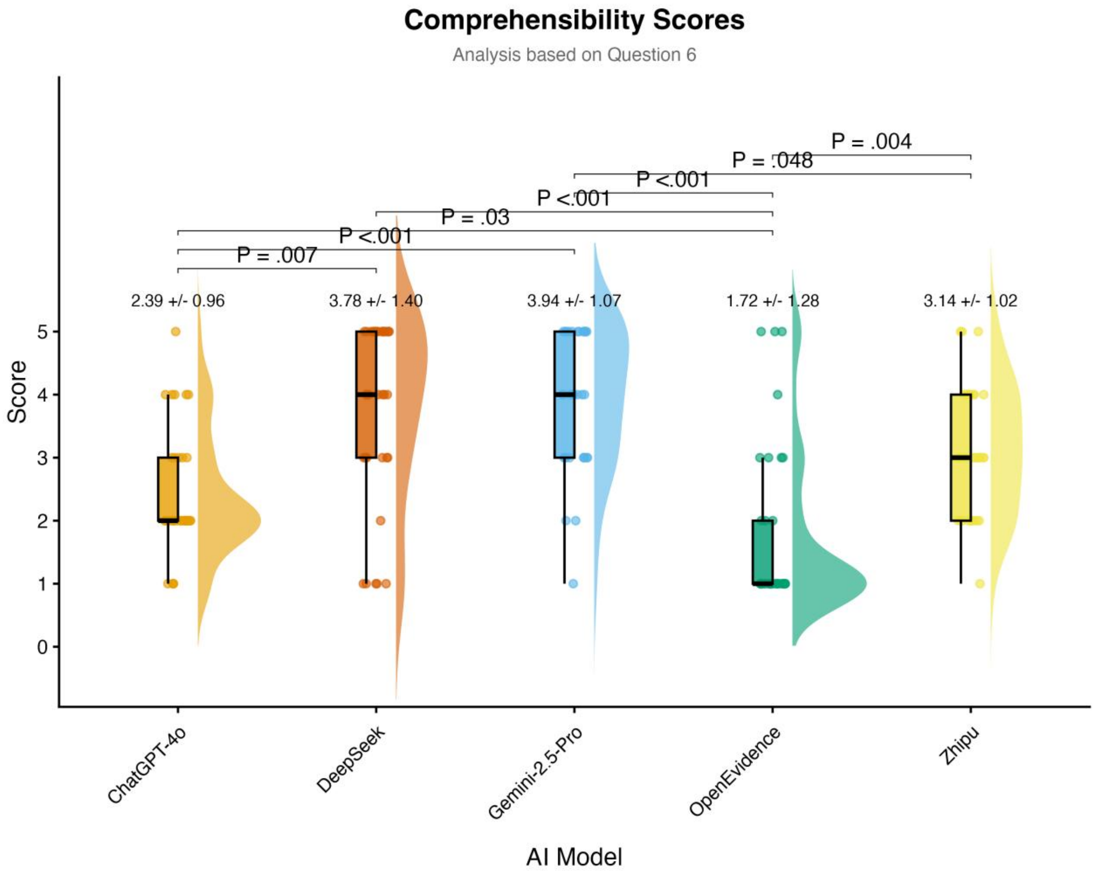
Comprehensibility Scores
Analysis based on Question 6
P = .004
P = .048
P <.001
P <.001
P = .03
P <.001
P = .007
2.39 +/- 0.96
3.78 +/- 1.40
3.94 +/- 1.07
1.72 +/- 1.28
3.14 +/- 1.02
Score
ChatGPT-4o
DeepSeek
Gemini-2.5-Pro
OpenEvidence
Zhipu
AI Model

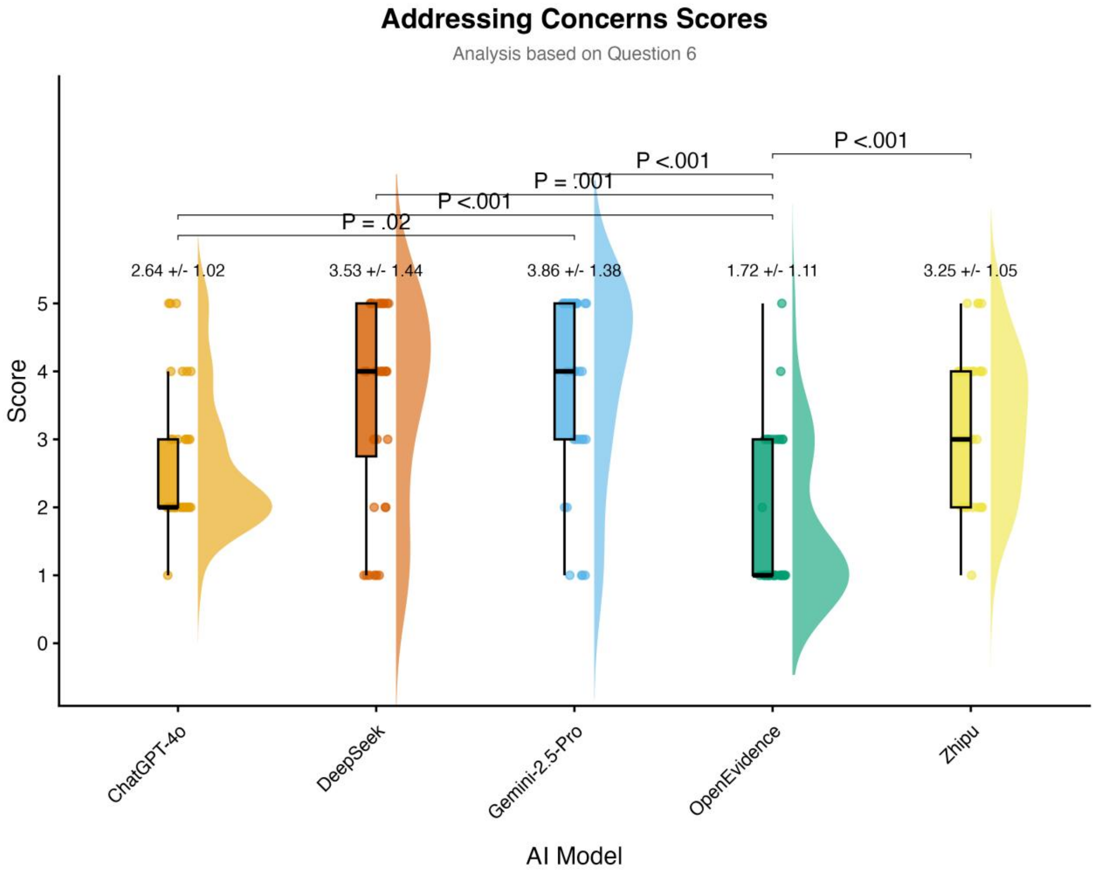
Addressing Concerns Scores
Analysis based on Question 6
P <.001
P <.001
P = .001
P <.001
P = .02
2.64 +/- 1.02
3.53 +/- 1.44
3.86 +/- 1.38
1.72 +/- 1.11
3.25 +/- 1.05
Score
AI Model
ChatGPT-4o
DeepSeek
Gemini-2.5-Pro
OpenEvidence
Zhipu

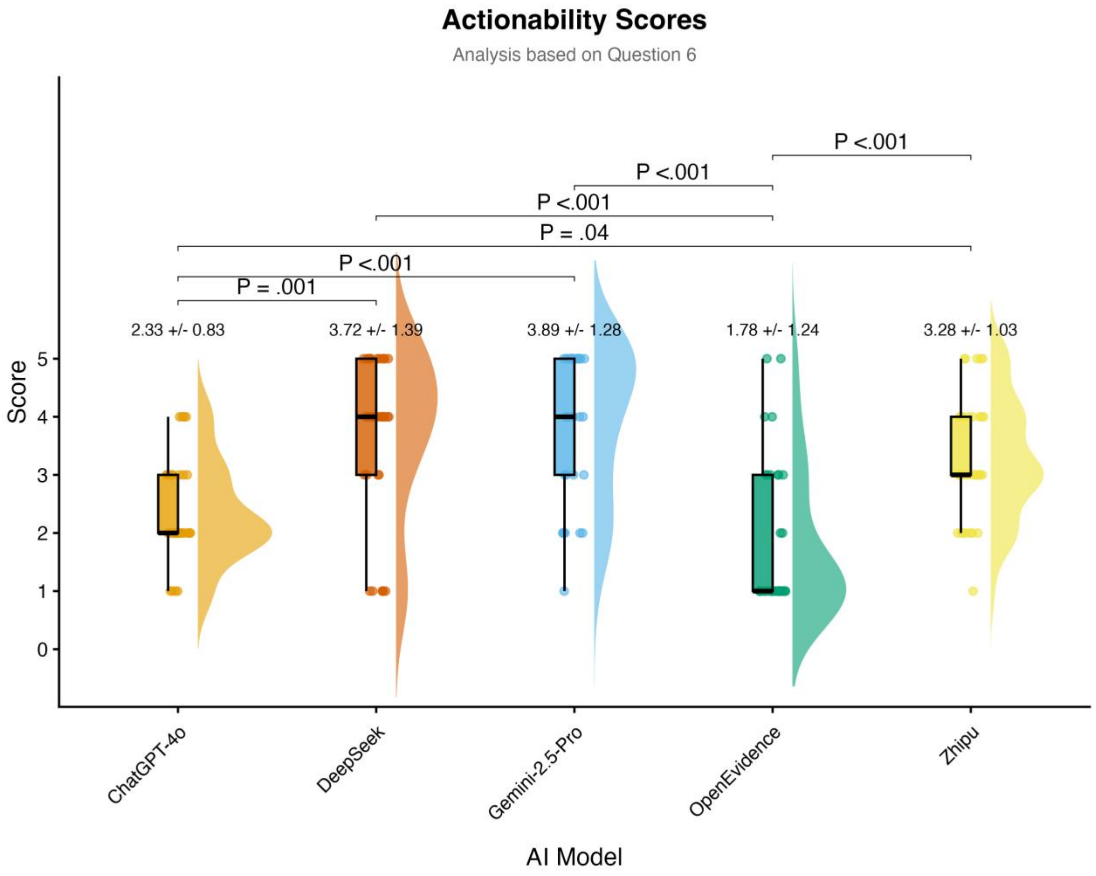
Actionability Scores
Analysis based on Question 6
P <.001
P <.001
P <.001
P = .04
P <.001
P = .001
2.33 +/- 0.83
3.72 +/- 1.39
3.89 +/- 1.28
1.78 +/- 1.24
3.28 +/- 1.03
Score
AI Model
ChatGPT-4o
DeepSeek
Gemini-2.5-Pro
OpenEvidence
Zhipu

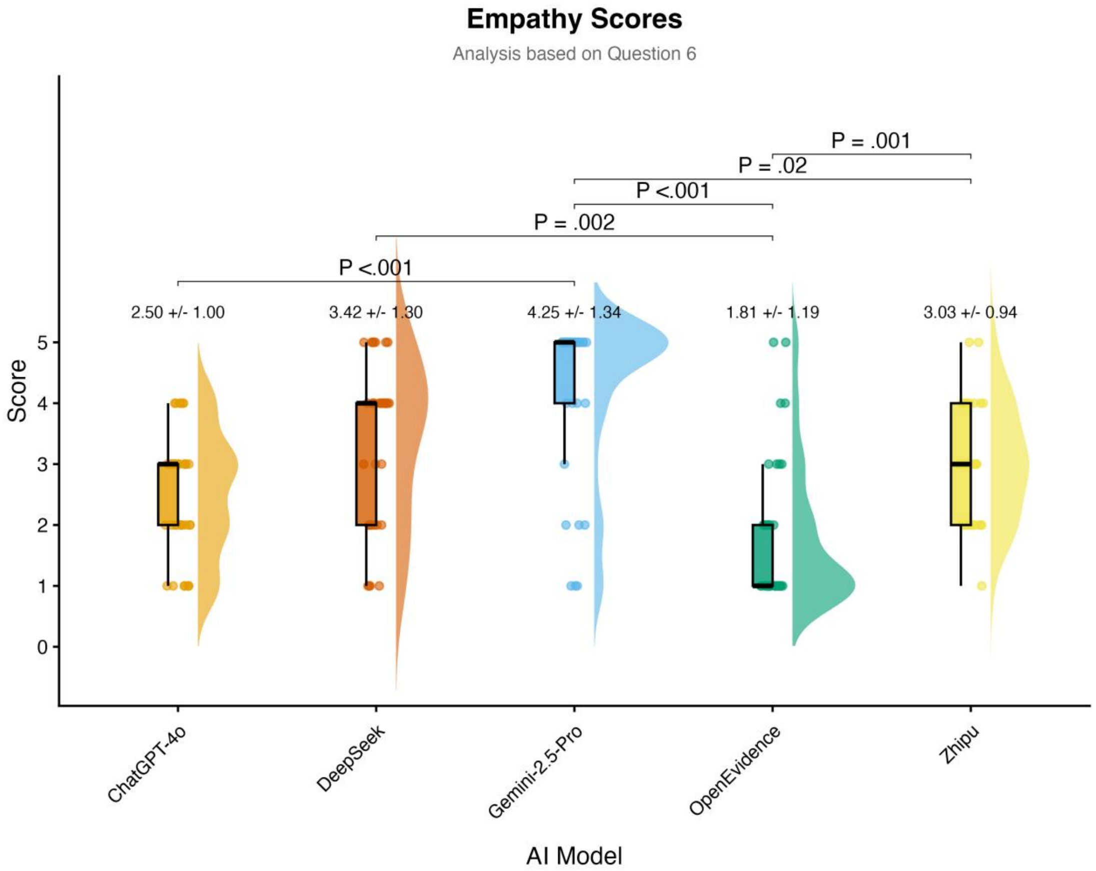

Empathy Scores
Analysis based on Question 6
P = .001
P = .02
P <.001
P = .002
P <.001
2.50 +/- 1.00
3.42 +/- 1.30
4.25 +/- 1.34
1.81 +/- 1.19
3.03 +/- 0.94
Score
0
1
2
3
4
5
ChatGPT-4o
DeepSeek
Gemini-2.5-Pro
OpenEvidence
Zhipu
AI Model

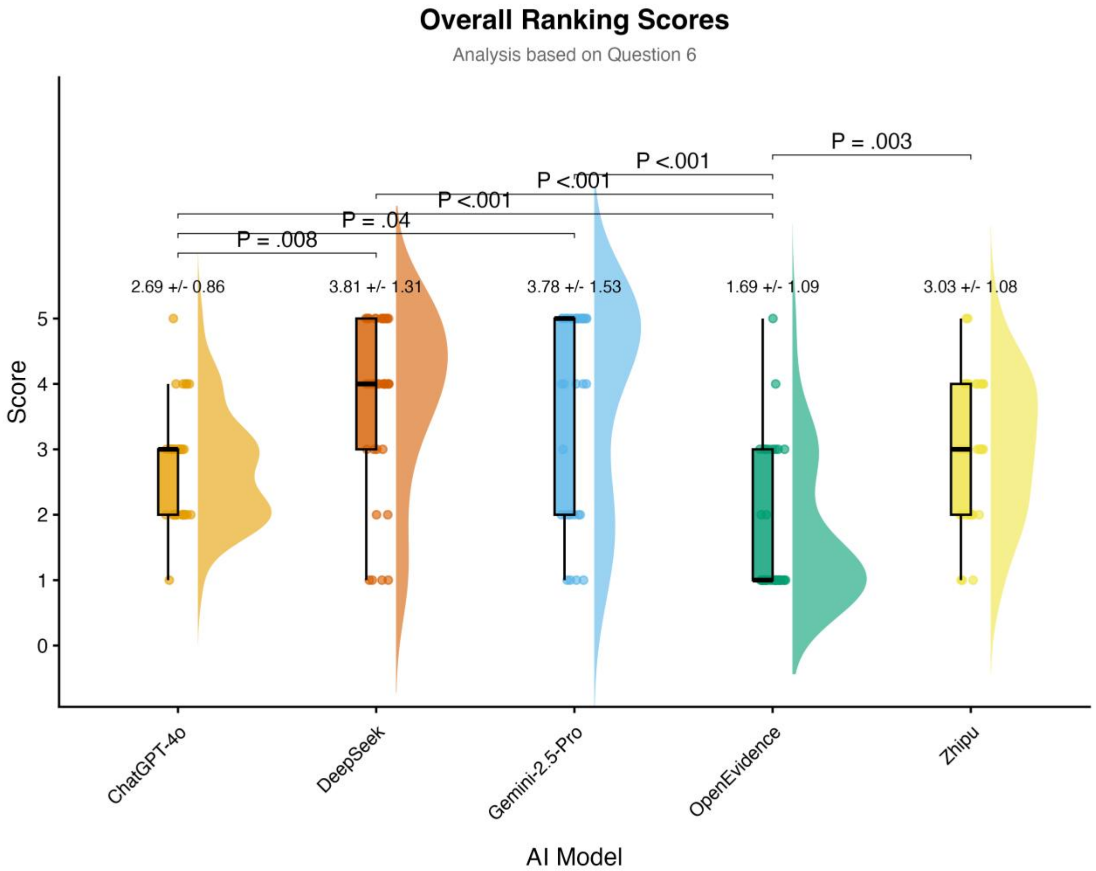

Overall Ranking Scores
Analysis based on Question 6
P = .003
P <.001
P <.001
P <.001
P = .04
P = .008
2.69 +/- 0.86
3.81 +/- 1.31
3.78 +/- 1.53
1.69 +/- 1.09
3.03 +/- 1.08
Score
0
1
2
3
4
5
ChatGPT-4o
DeepSeek
Gemini-2.5-Pro
OpenEvidence
Zhipu
AI Model

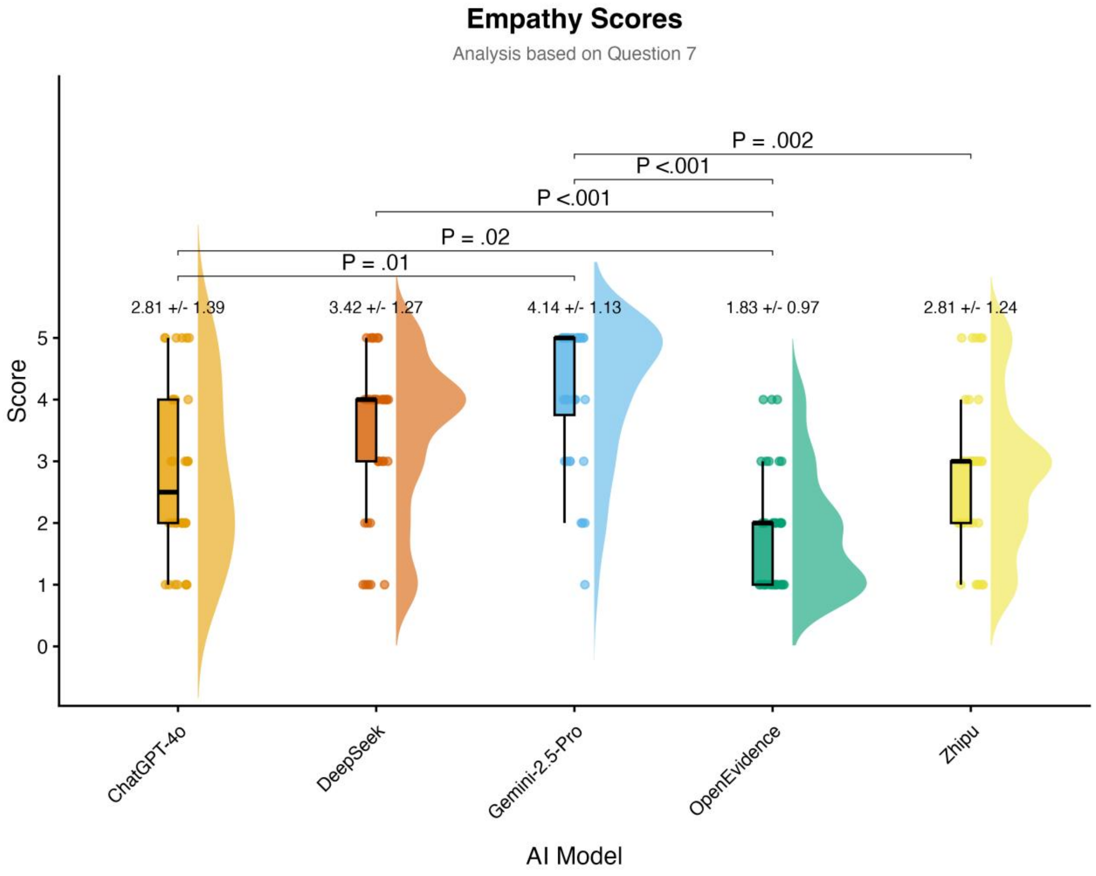
Empathy Scores
Analysis based on Question 7
P = .002
P <.001
P <.001
P = .02
P = .01
2.81 +/- 1.39
3.42 +/- 1.27
4.14 +/- 1.13
1.83 +/- 0.97
2.81 +/- 1.24
Score
0
1
2
3
4
5
ChatGPT-4o
DeepSeek
Gemini-2.5-Pro
OpenEvidence
Zhipu
AI Model

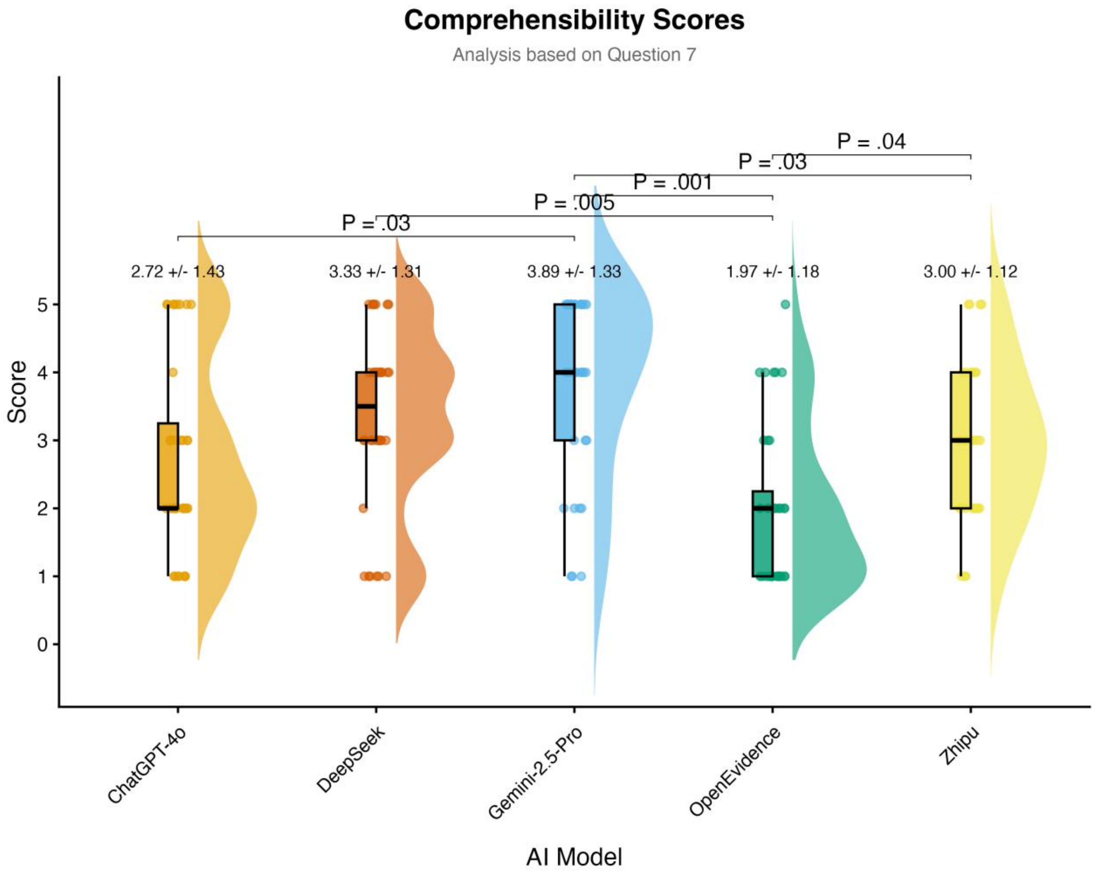
Comprehensibility Scores
Analysis based on Question 7
P = .04
P = .03
P = .001
P = .005
P = .03
2.72 +/- 1.43
3.33 +/- 1.31
3.89 +/- 1.33
1.97 +/- 1.18
3.00 +/- 1.12
Score
0
1
2
3
4
5
ChatGPT-4o
DeepSeek
Gemini-2.5-Pro
OpenEvidence
Zhipu
AI Model

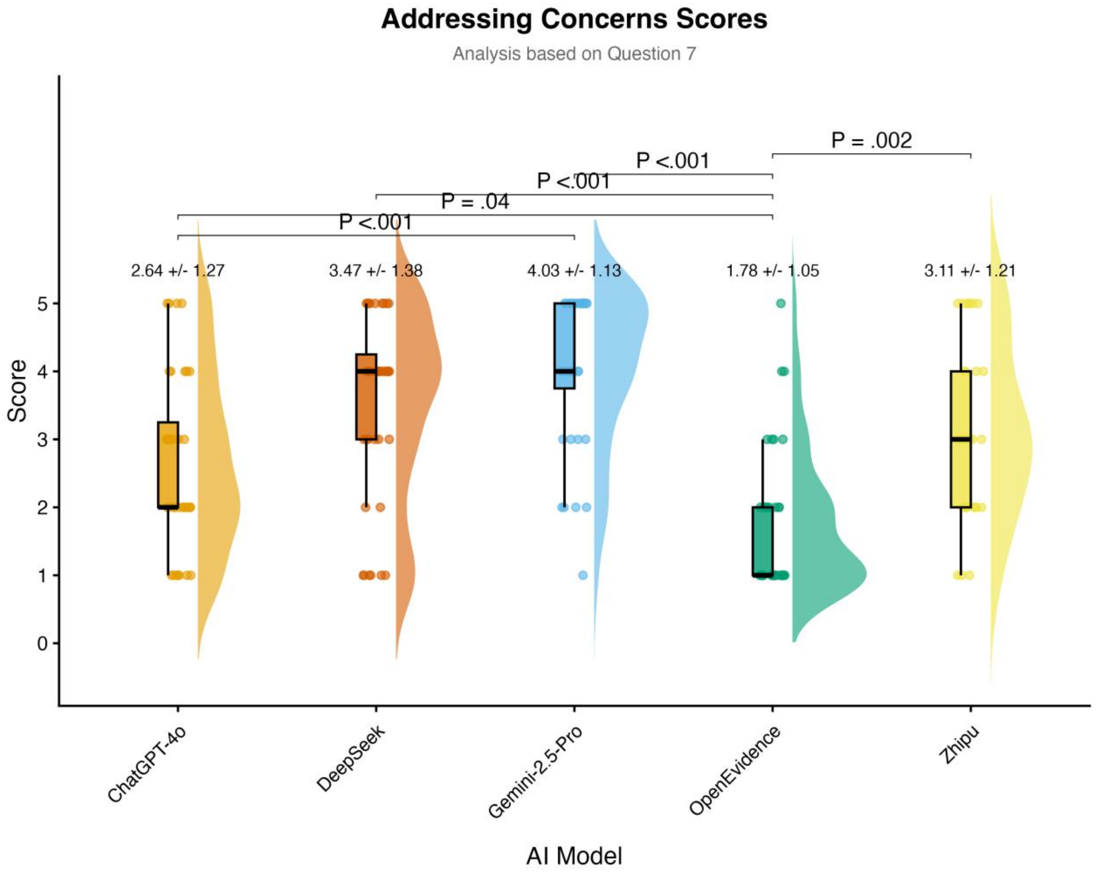

Addressing Concerns Scores
Analysis based on Question 7
P = .002
P <.001
P <.001
P = .04
P <.001
2.64 +/- 1.27
3.47 +/- 1.38
4.03 +/- 1.13
1.78 +/- 1.05
3.11 +/- 1.21
Score
5
4
3
2
1
0
ChatGPT-4o
DeepSeek
Gemini-2.5-Pro
OpenEvidence
Zhipu
AI Model

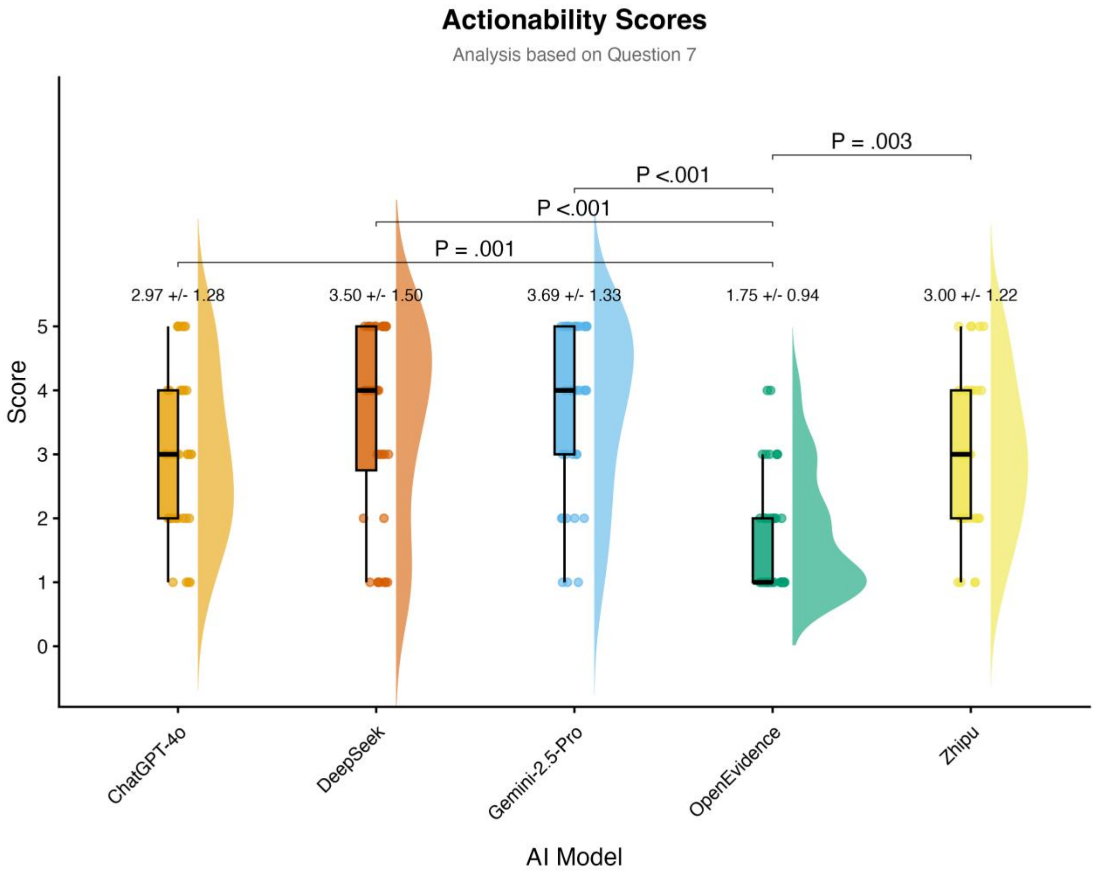

Actionability Scores
Analysis based on Question 7
P = .003
P <.001
P <.001
P = .001
2.97 +/- 1.28
3.50 +/- 1.50
3.69 +/- 1.33
1.75 +/- 0.94
3.00 +/- 1.22
Score
5
4
3
2
1
0
ChatGPT-4o
DeepSeek
Gemini-2.5-Pro
OpenEvidence
Zhipu
AI Model

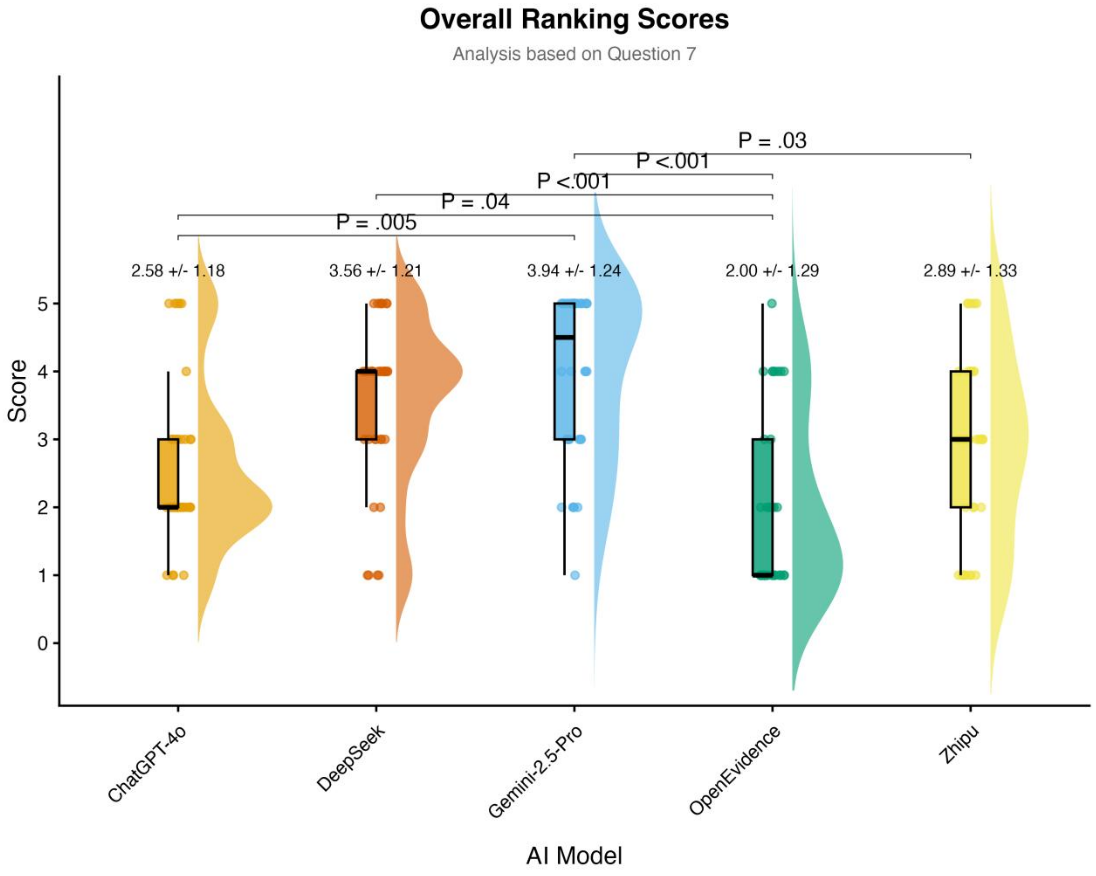
Overall Ranking Scores
Analysis based on Question 7
P = .03
P <.001
P <.001
P = .04
P = .005
2.58 +/- 1.18
3.56 +/- 1.21
3.94 +/- 1.24
2.00 +/- 1.29
2.89 +/- 1.33
Score
ChatGPT-4o
DeepSeek
Gemini-2.5-Pro
OpenEvidence
Zhipu
AI Model

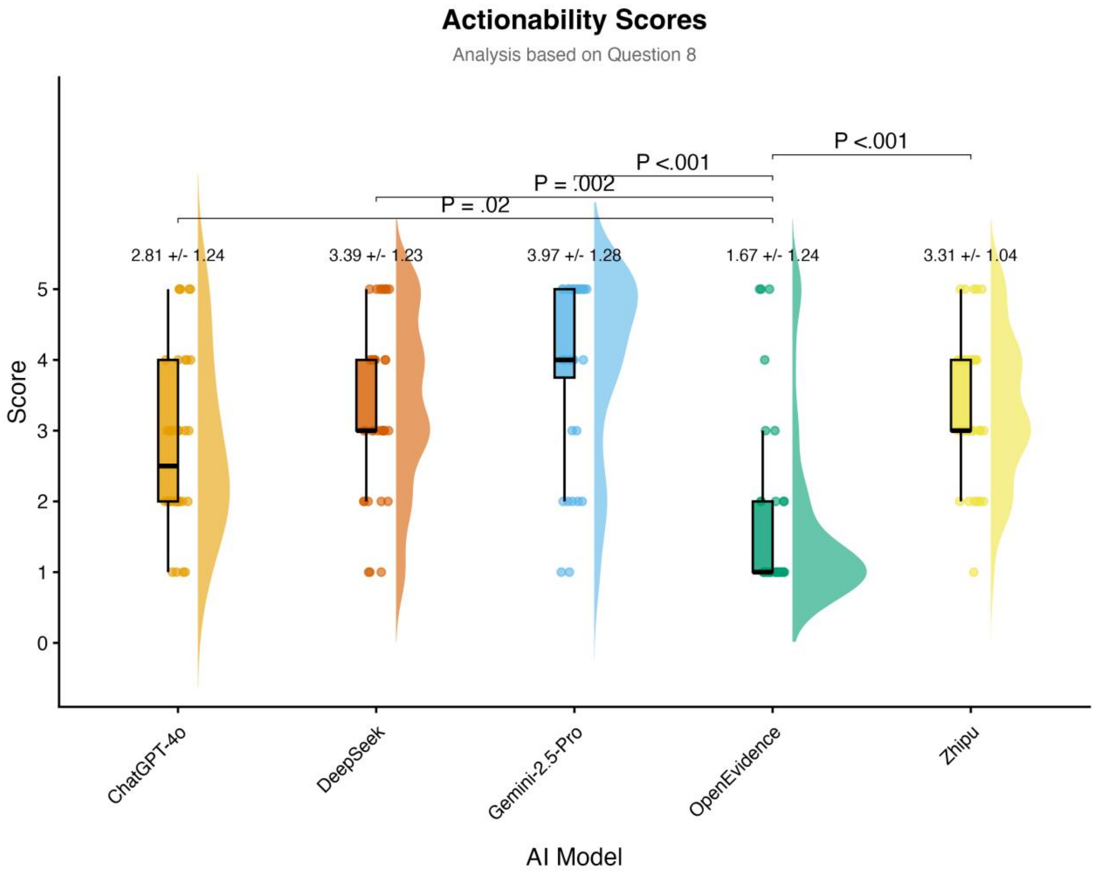
Actionability Scores
Analysis based on Question 8
P <.001
P <.001
P = .002
P = .02
2.81 +/- 1.24
3.39 +/- 1.23
3.97 +/- 1.28
1.67 +/- 1.24
3.31 +/- 1.04
Score
ChatGPT-4o
DeepSeek
Gemini-2.5-Pro
OpenEvidence
Zhipu
AI Model

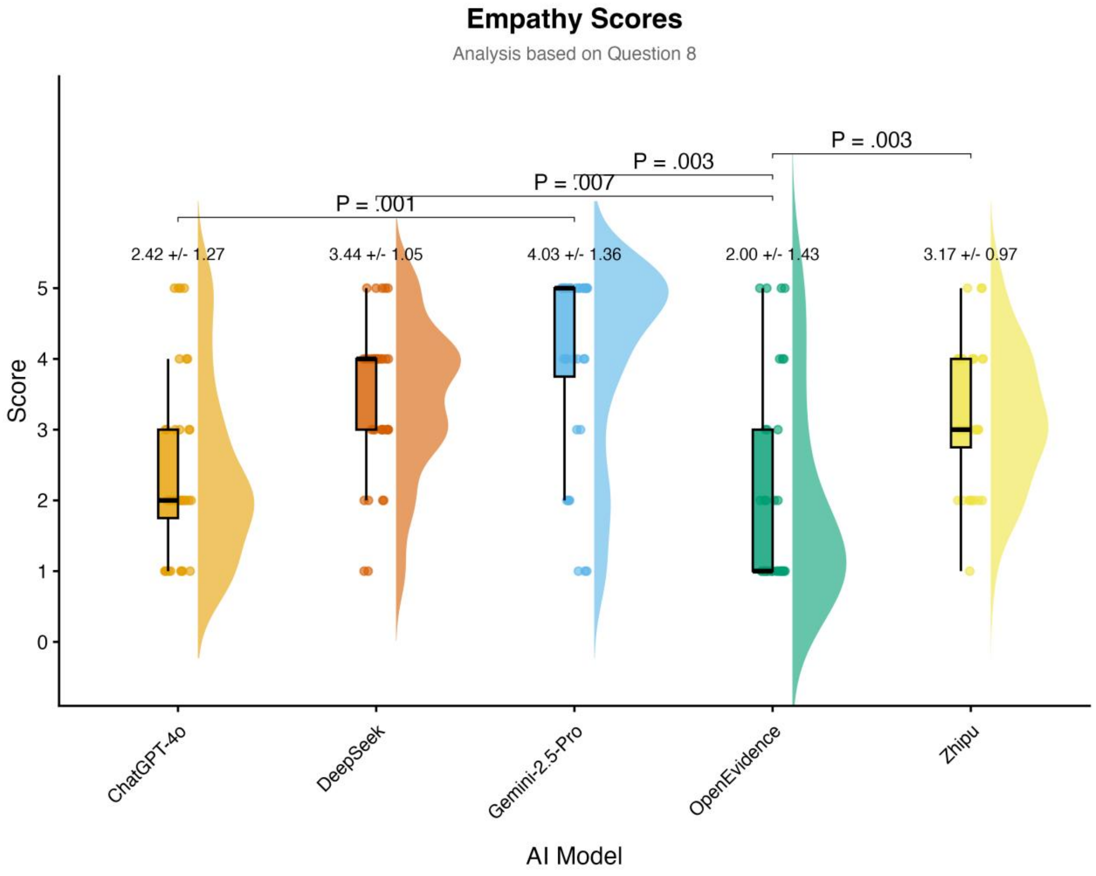

Empathy Scores
Analysis based on Question 8
P = .003
P = .003
P = .007
P = .001
2.42 +/- 1.27
3.44 +/- 1.05
4.03 +/- 1.36
2.00 +/- 1.43
3.17 +/- 0.97
Score
0
1
2
3
4
5
ChatGPT-4o
DeepSeek
Gemini-2.5-Pro
OpenEvidence
Zhipu
AI Model

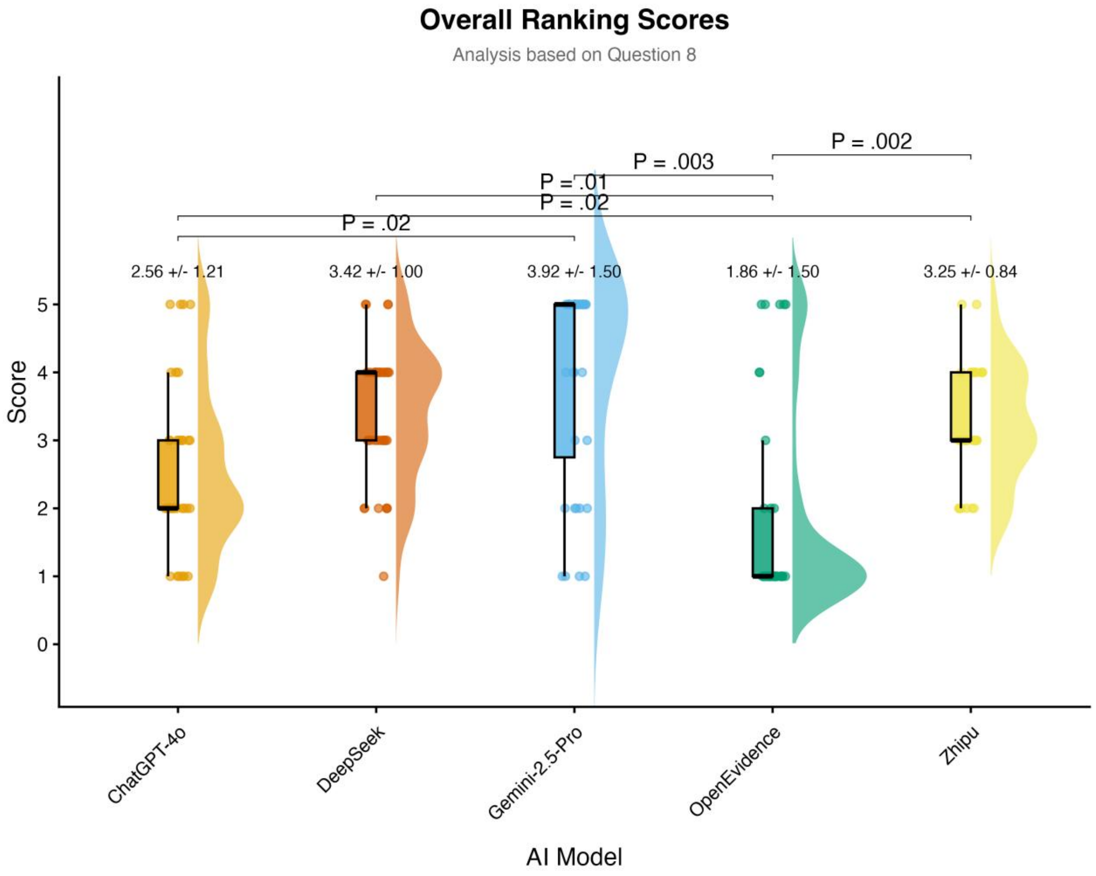

Overall Ranking Scores
Analysis based on Question 8
P = .002
P = .003
P = .01
P = .02
P = .02
2.56 +/- 1.21
3.42 +/- 1.00
3.92 +/- 1.50
1.86 +/- 1.50
3.25 +/- 0.84
Score
0
1
2
3
4
5
ChatGPT-4o
DeepSeek
Gemini-2.5-Pro
OpenEvidence
Zhipu
AI Model

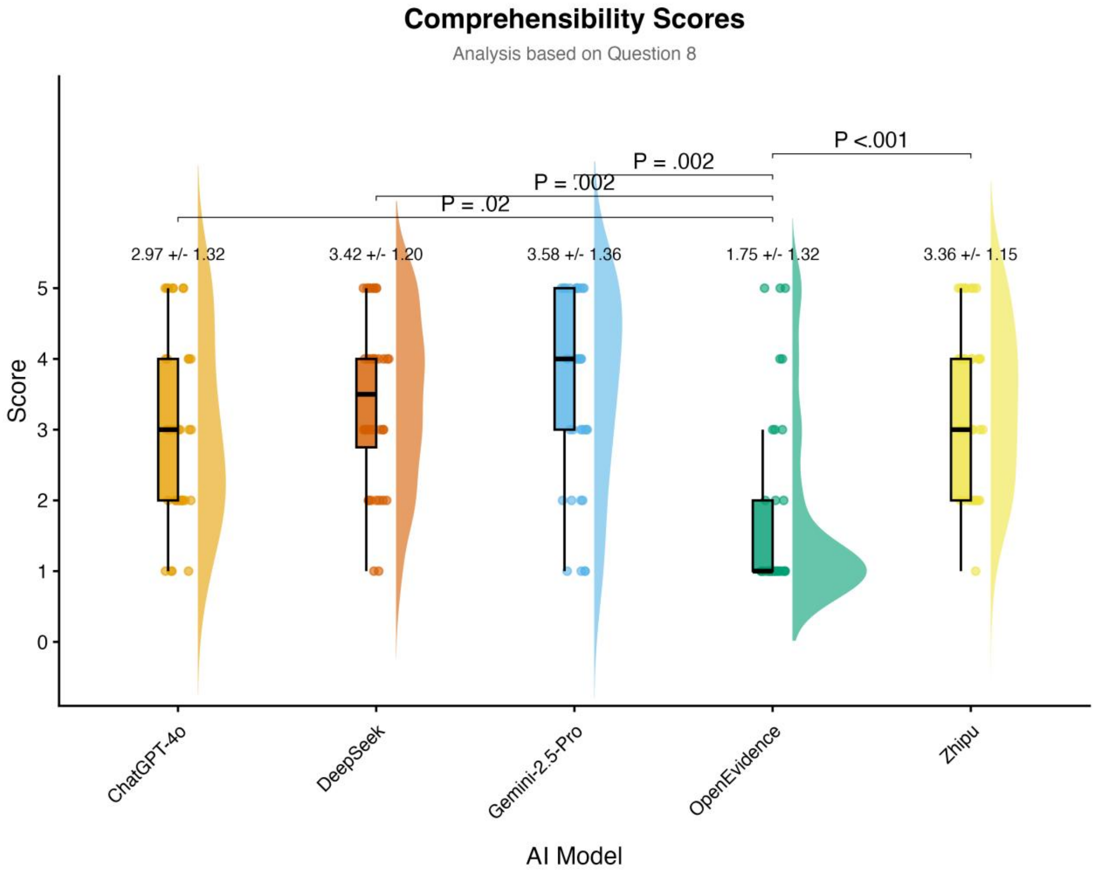
Comprehensibility Scores
Analysis based on Question 8
P <.001
P = .002
P = .002
P = .02
2.97 +/- 1.32
3.42 +/- 1.20
3.58 +/- 1.36
1.75 +/- 1.32
3.36 +/- 1.15
Score
0
1
2
3
4
5
ChatGPT-4o
DeepSeek
Gemini-2.5-Pro
OpenEvidence
Zhipu
AI Model

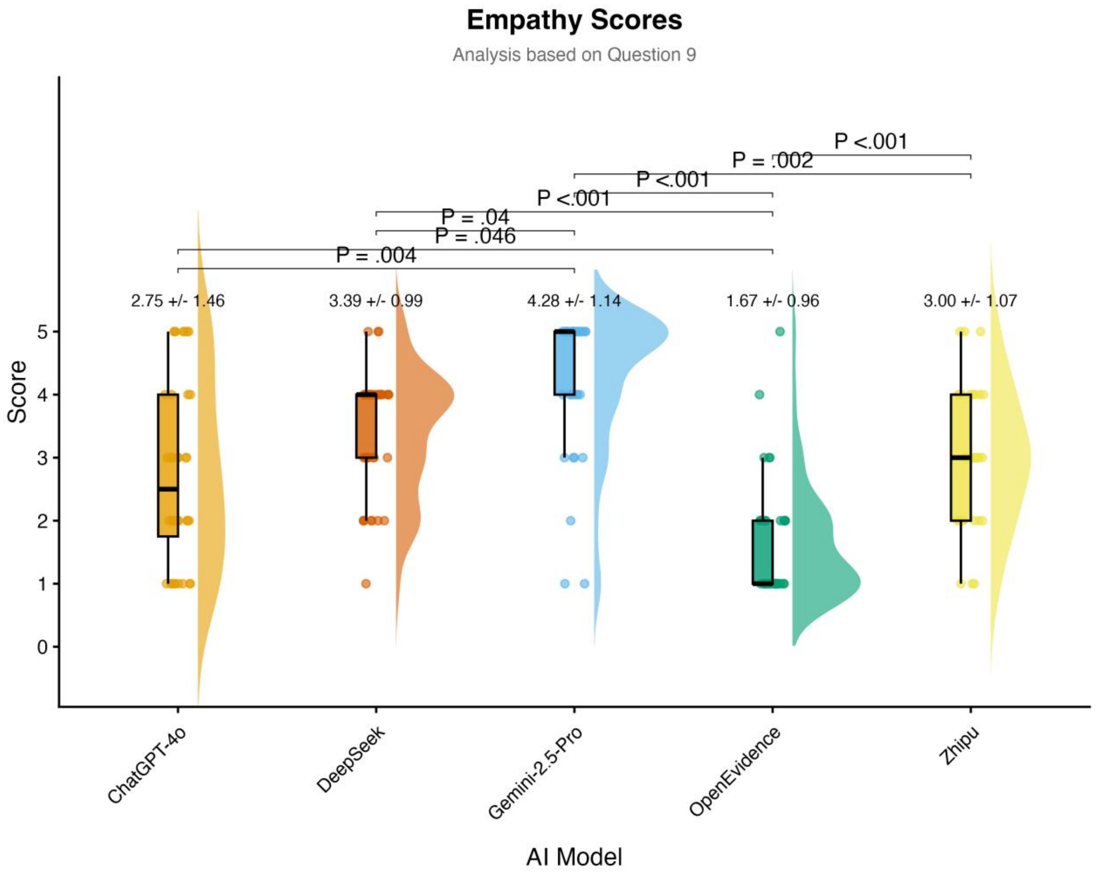
Empathy Scores
Analysis based on Question 9
P <.001
P = .002
P <.001
P <.001
P = .04
P = .046
P = .004
2.75 +/- 1.46
3.39 +/- 0.99
4.28 +/- 1.14
1.67 +/- 0.96
3.00 +/- 1.07
Score
0
1
2
3
4
5
ChatGPT-4o
DeepSeek
Gemini-2.5-Pro
OpenEvidence
Zhipu
AI Model

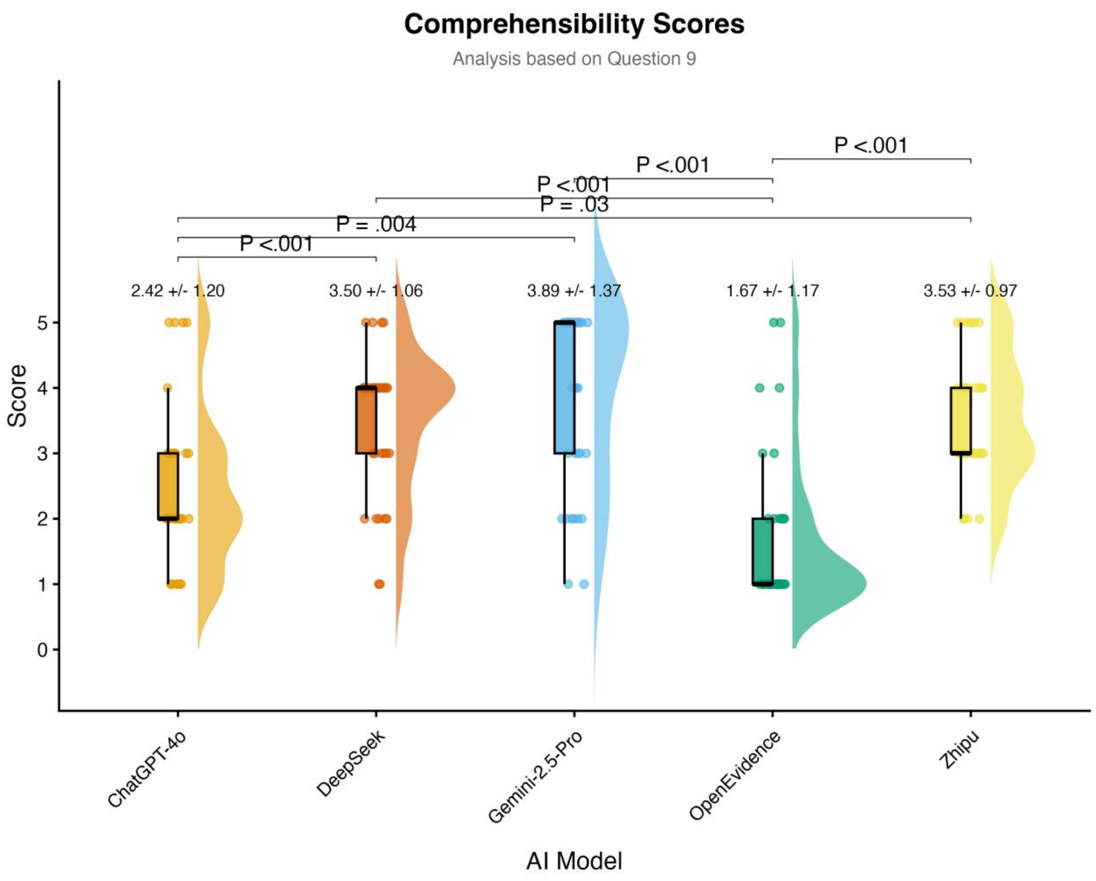
Comprehensibility Scores
Analysis based on Question 9
P <.001
P <.001
P <.001
P = .03
P = .004
P <.001
2.42 +/- 1.20
3.50 +/- 1.06
3.89 +/- 1.37
1.67 +/- 1.17
3.53 +/- 0.97
Score
ChatGPT-4o
DeepSeek
Gemini-2.5-Pro
OpenEvidence
Zhipu
AI Model

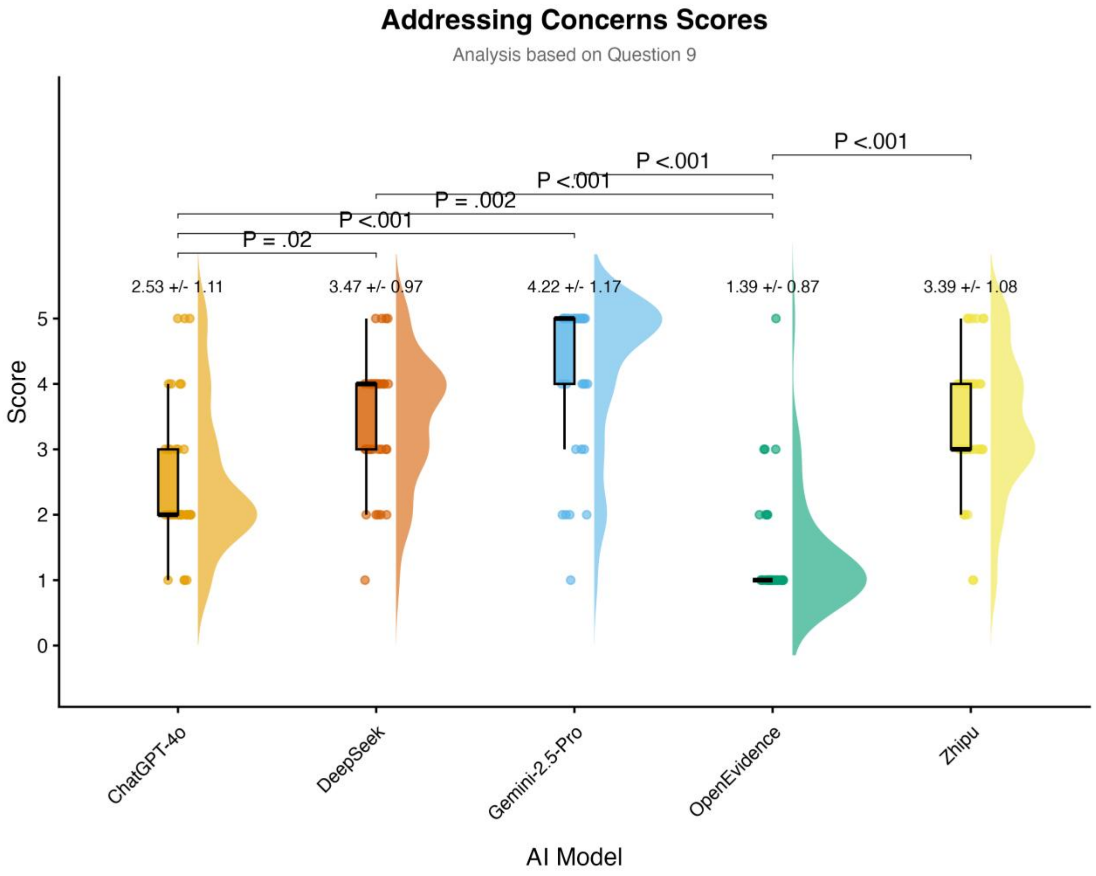
Addressing Concerns Scores
Analysis based on Question 9
P <.001
P <.001
P <.001
P = .002
P <.001
P = .02
2.53 +/- 1.11
3.47 +/- 0.97
4.22 +/- 1.17
1.39 +/- 0.87
3.39 +/- 1.08
Score
ChatGPT-4o
DeepSeek
Gemini-2.5-Pro
OpenEvidence
Zhipu
AI Model

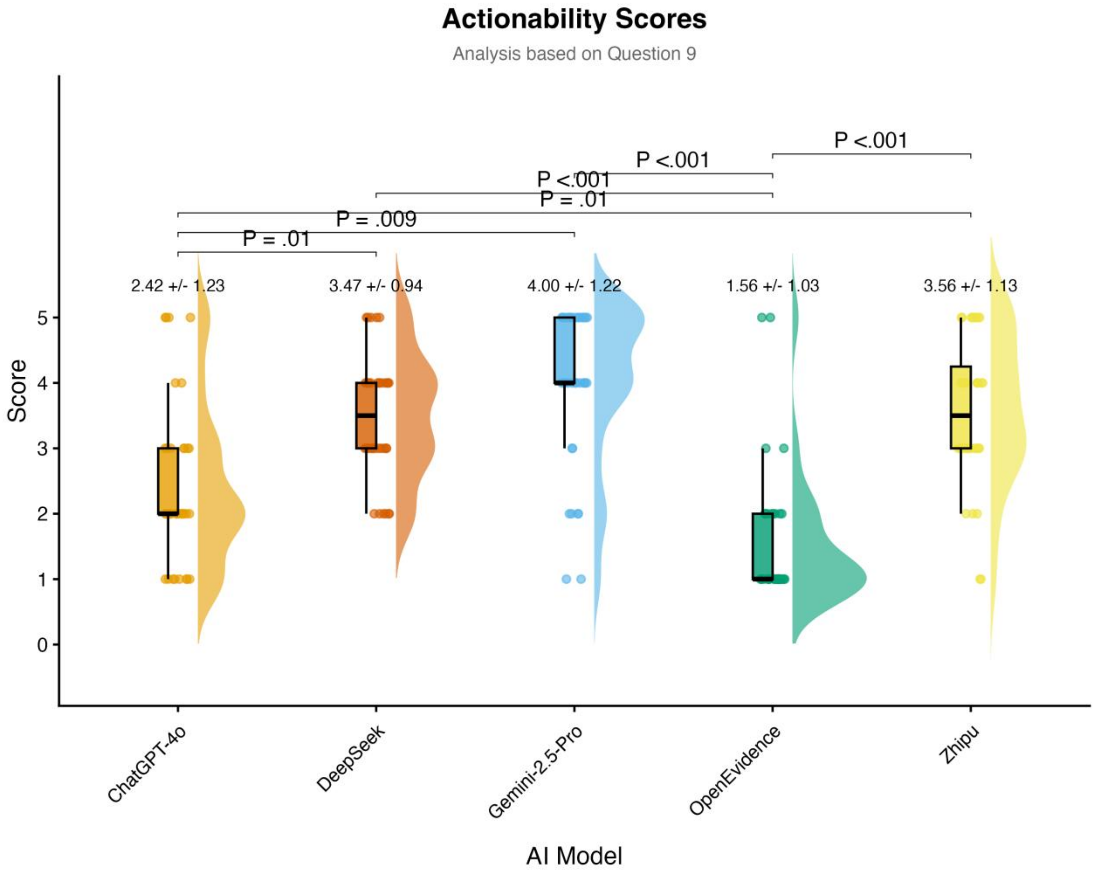
Actionability Scores
Analysis based on Question 9
P <.001
P <.001
P <.001
P = .01
P = .009
P = .01
2.42 +/- 1.23
3.47 +/- 0.94
4.00 +/- 1.22
1.56 +/- 1.03
3.56 +/- 1.13
Score
0
1
2
3
4
5
ChatGPT-4o
DeepSeek
Gemini-2.5-Pro
OpenEvidence
Zhipu
AI Model

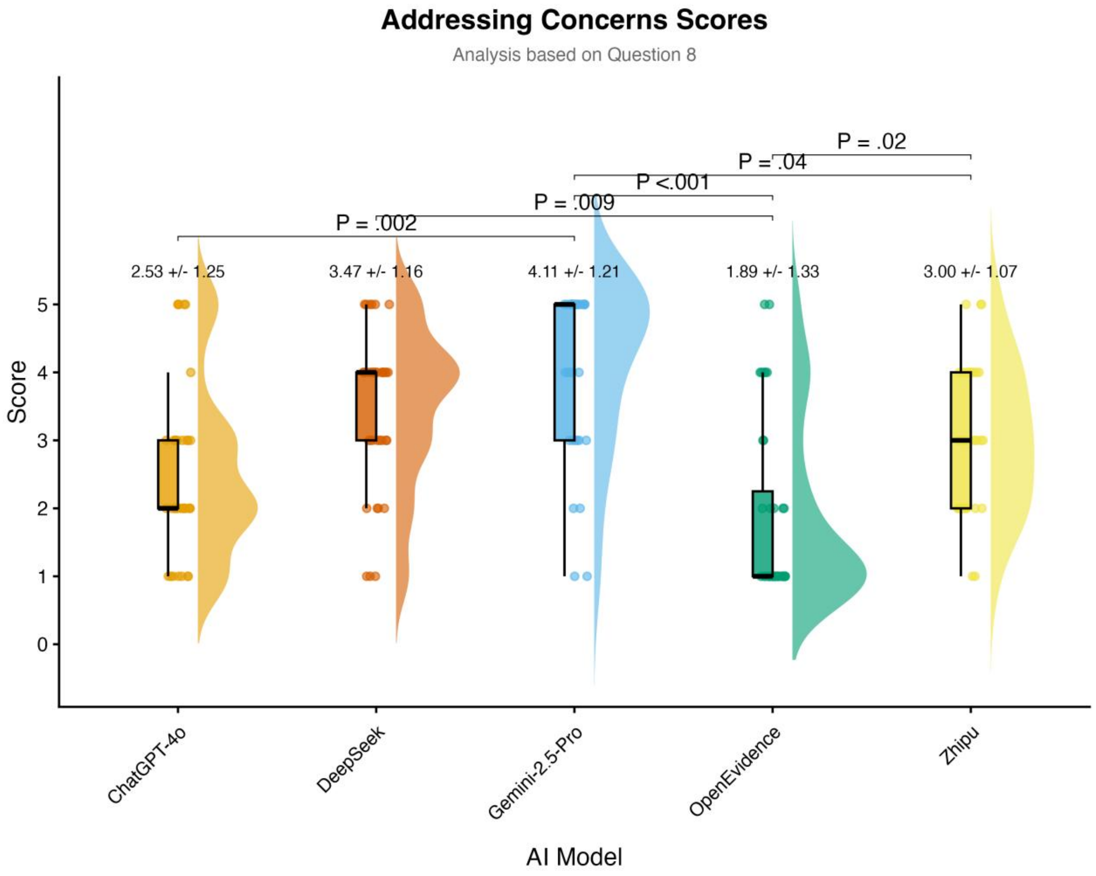
Addressing Concerns Scores
Analysis based on Question 8
P = .02
P = .04
P <.001
P = .009
P = .002
2.53 +/- 1.25
3.47 +/- 1.16
4.11 +/- 1.21
1.89 +/- 1.33
3.00 +/- 1.07
Score
0
1
2
3
4
5
ChatGPT-4o
DeepSeek
Gemini-2.5-Pro
OpenEvidence
Zhipu
AI Model

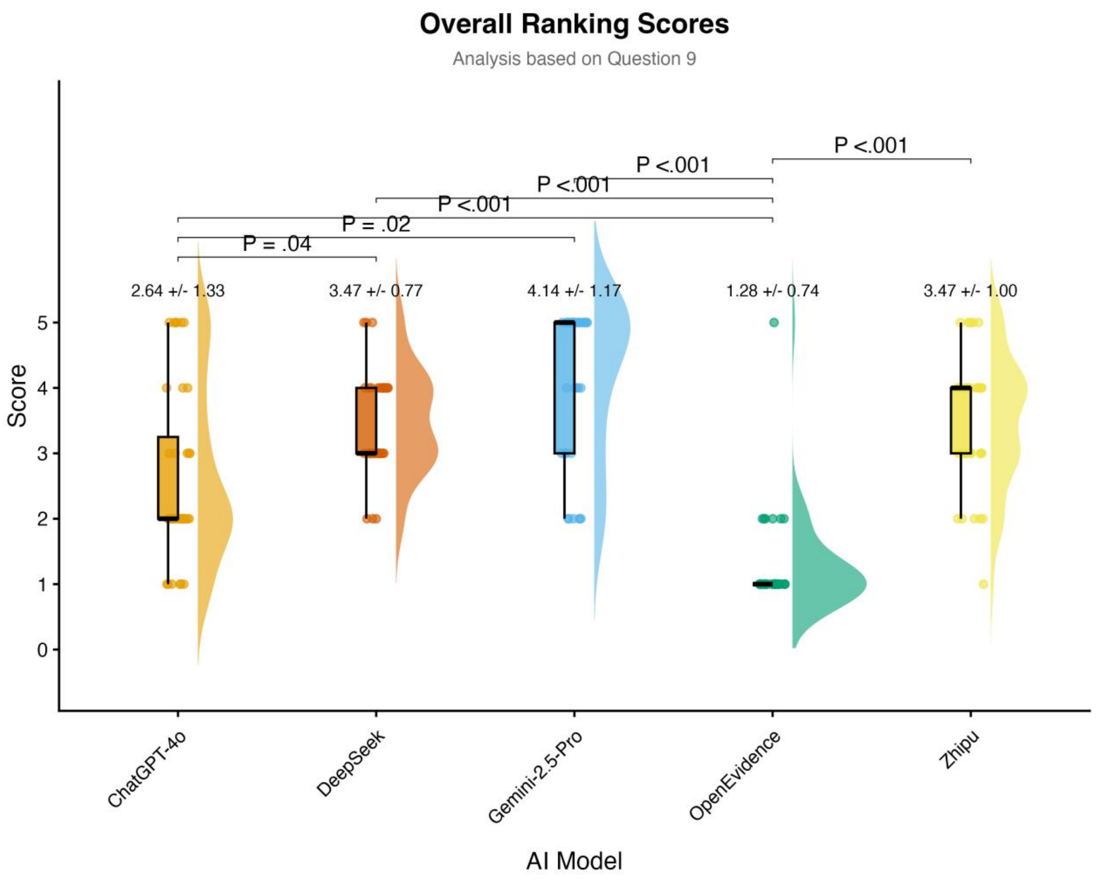

Overall Ranking Scores
Analysis based on Question 9
P <.001
P <.001
P <.001
P <.001
P = .02
P = .04
2.64 +/- 1.33
3.47 +/- 0.77
4.14 +/- 1.17
1.28 +/- 0.74
3.47 +/- 1.00
Score
5
4
3
2
1
0
ChatGPT-4o
DeepSeek
Gemini-2.5-Pro
OpenEvidence
Zhipu
AI Model

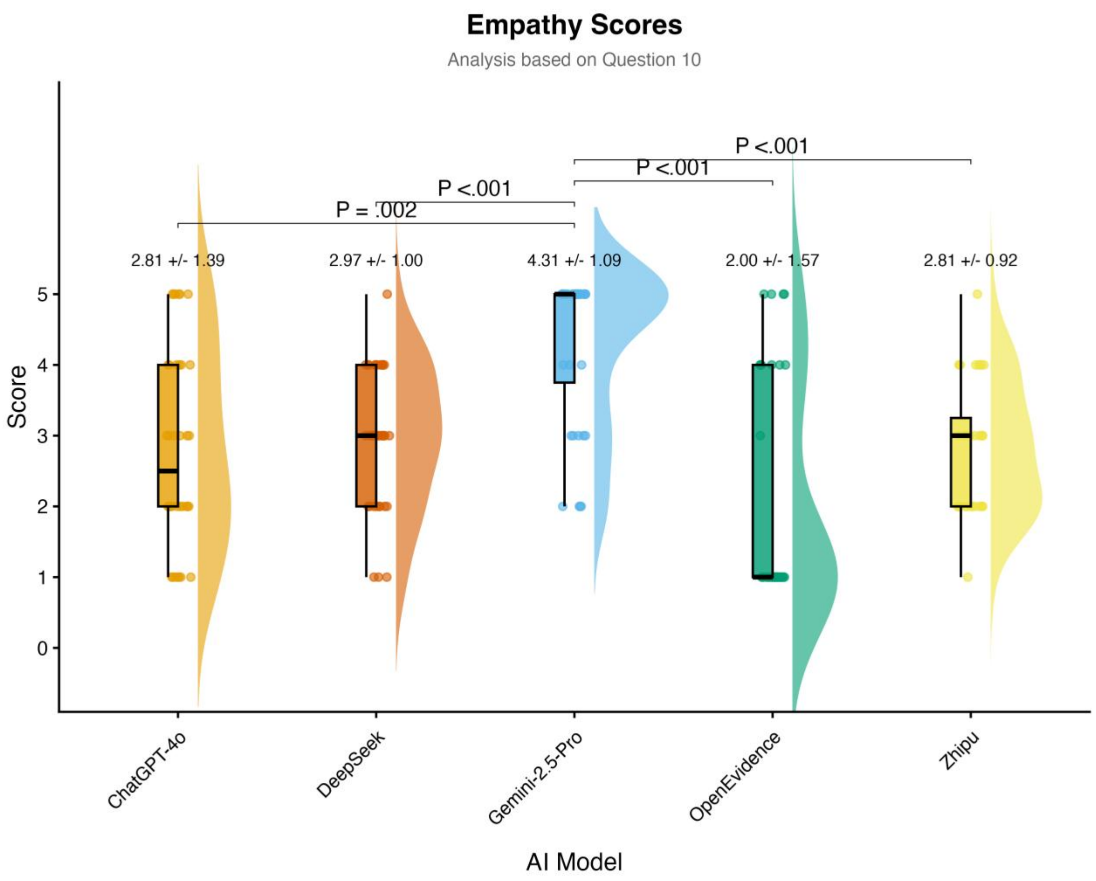

Empathy Scores
Analysis based on Question 10
P <.001
P <.001
P <.001
P = .002
2.81 +/- 1.39
2.97 +/- 1.00
4.31 +/- 1.09
2.00 +/- 1.57
2.81 +/- 0.92
Score
5
4
3
2
1
0
ChatGPT-4o
DeepSeek
Gemini-2.5-Pro
OpenEvidence
Zhipu
AI Model

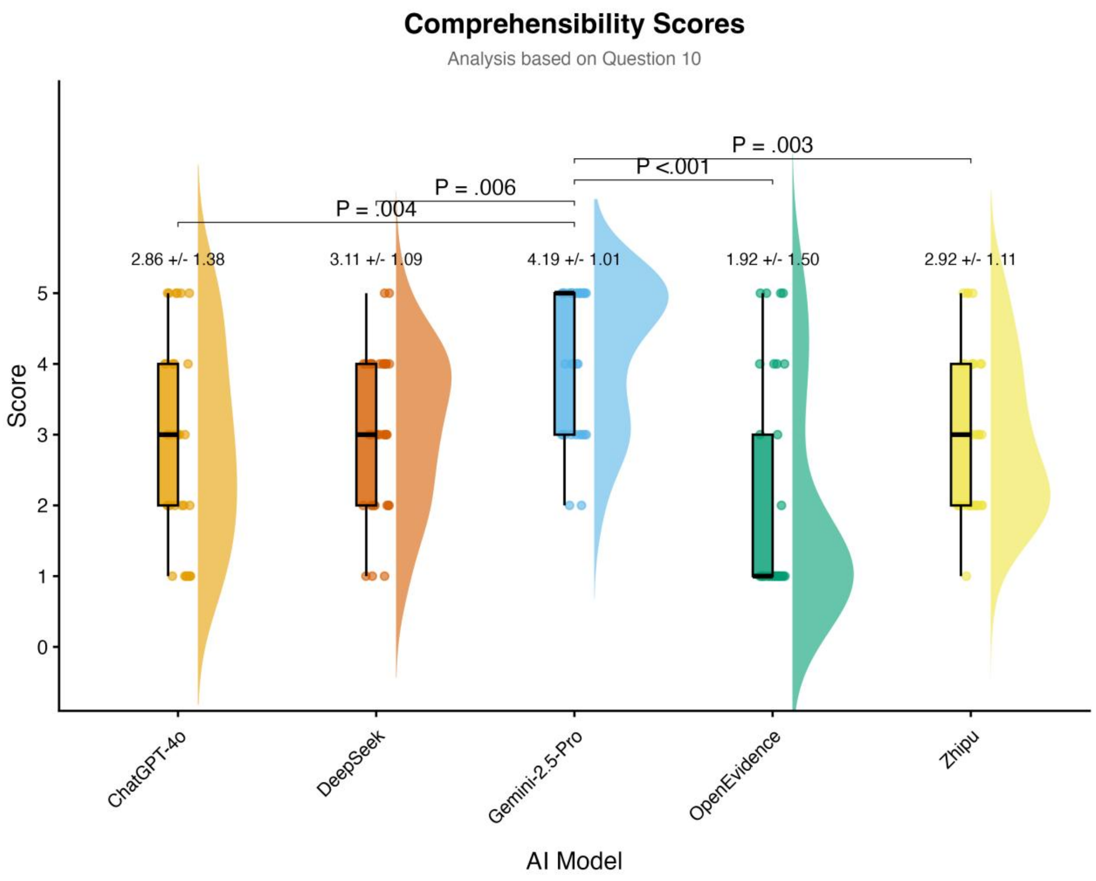
Comprehensibility Scores
Analysis based on Question 10
P = .003
P <.001
P = .006
P = .004
2.86 +/- 1.38
3.11 +/- 1.09
4.19 +/- 1.01
1.92 +/- 1.50
2.92 +/- 1.11
Score
5
4
3
2
1
0
ChatGPT-4o
DeepSeek
Gemini-2.5-Pro
OpenEvidence
Zhipu
AI Model

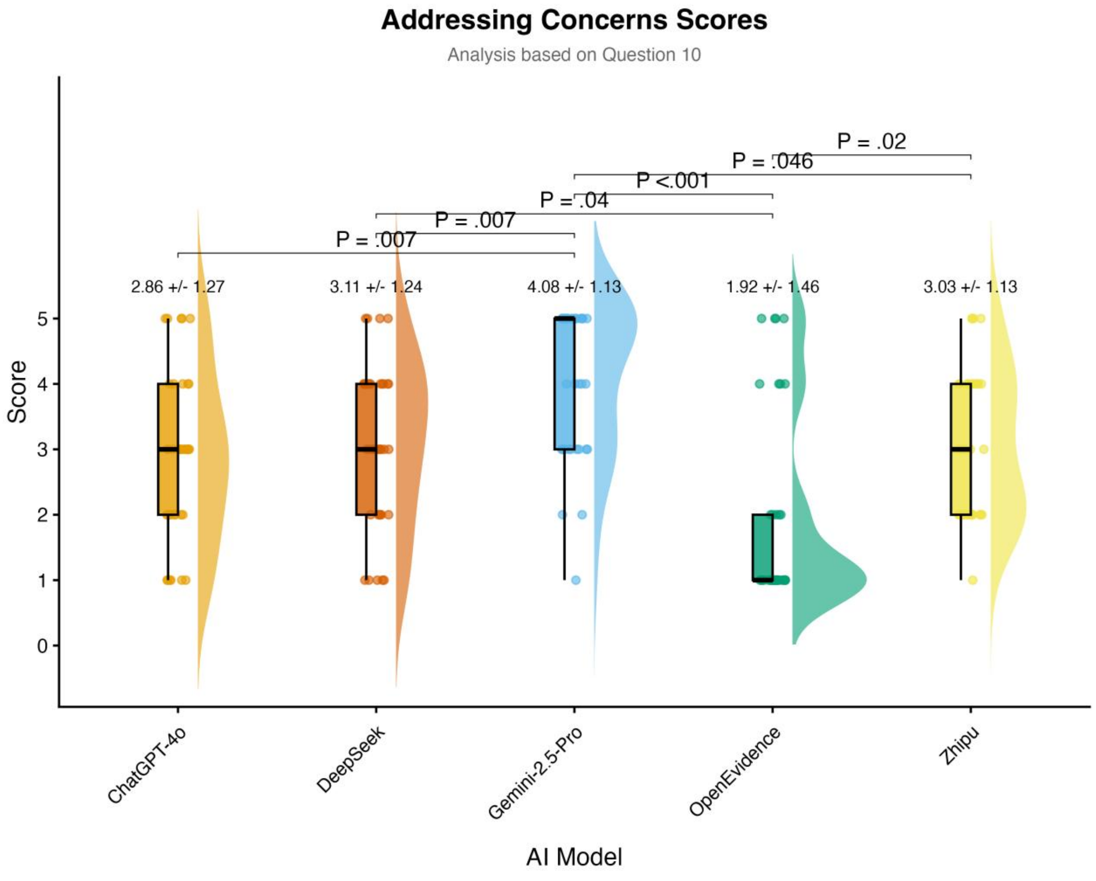
Addressing Concerns Scores
Analysis based on Question 10
P = .02
P = .046
P <.001
P = .04
P = .007
P = .007
2.86 +/- 1.27
3.11 +/- 1.24
4.08 +/- 1.13
1.92 +/- 1.46
3.03 +/- 1.13
Score
5
4
3
2
1
0
ChatGPT-4o
DeepSeek
Gemini-2.5-Pro
OpenEvidence
Zhipu
AI Model

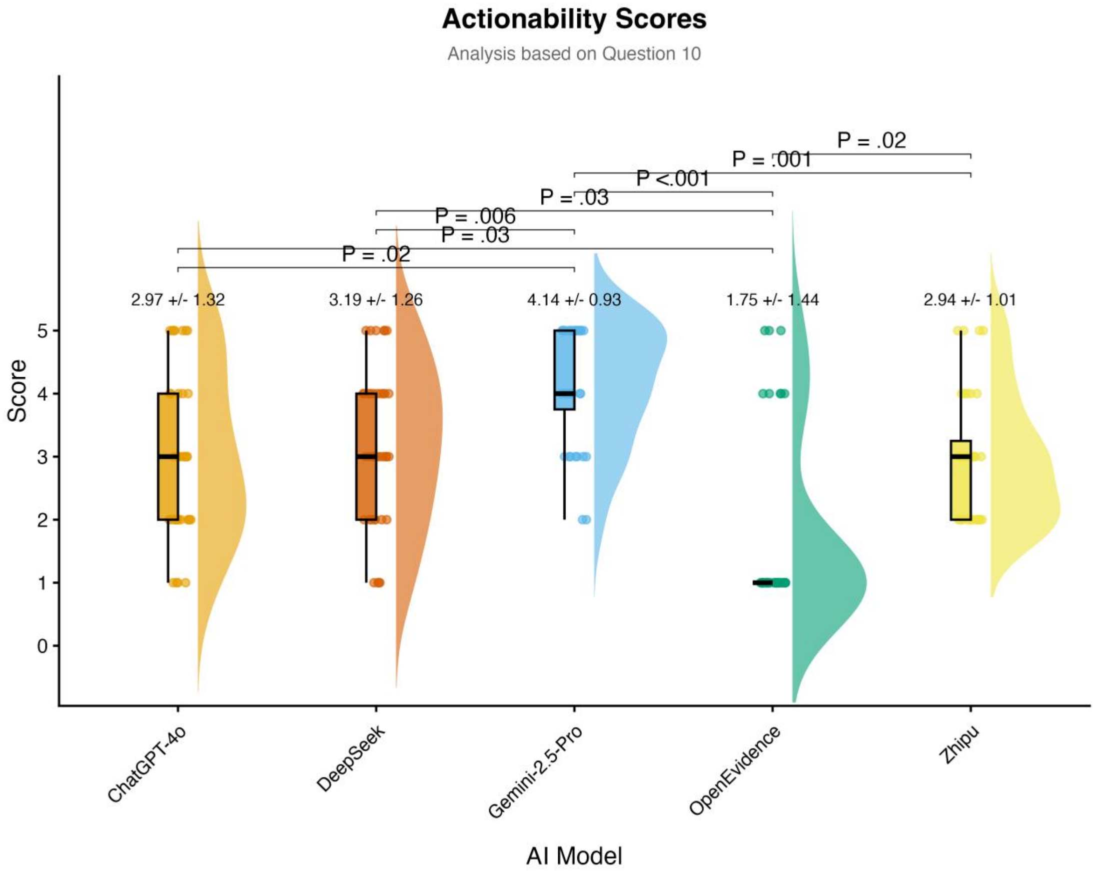
Actionability Scores
Analysis based on Question 10
P = .02
P = .001
P <.001
P = .03
P = .006
P = .03
P = .02
2.97 +/- 1.32
3.19 +/- 1.26
4.14 +/- 0.93
1.75 +/- 1.44
2.94 +/- 1.01
Score
ChatGPT-4o
DeepSeek
Gemini-2.5-Pro
OpenEvidence
Zhipu
AI Model

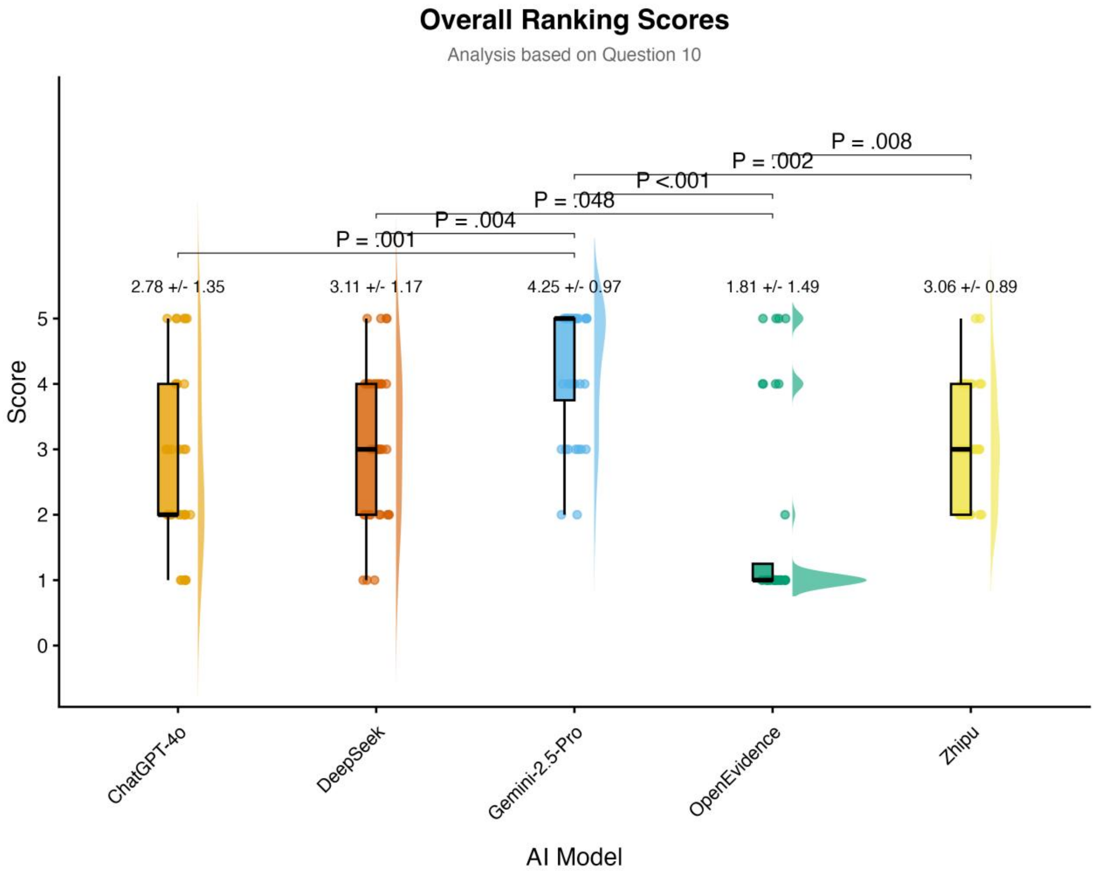
Overall Ranking Scores
Analysis based on Question 10
P = .008
P = .002
P <.001
P = .048
P = .004
P = .001
2.78 +/- 1.35
3.11 +/- 1.17
4.25 +/- 0.97
1.81 +/- 1.49
3.06 +/- 0.89
Score
ChatGPT-4o
DeepSeek
Gemini-2.5-Pro
OpenEvidence
Zhipu
AI Model
