# Supplementary material for: Performance of 5 Large Language Models in Perioperative Consultation for Pediatric Hypospadias: Cross-Sectional Comparative Study
Source: J Med Internet Res. 2026 Jul 29;28:e93393. doi: 10.2196/93393 (PMC13419283; doi:10.2196/93393)
Supplement: Multimedia Appendix 13 [file jmir-v28-e93393-s013.pdf]

European Association of Urology 2025 cross-check

This appendix presents the cross-verification of the eight-reviewer safety findings against specific EAU 2025 chapter sections.

Key safety findings with explicitEAU 2025anchors.

Finding	Model and question	EAU 2025 section	EAU verdict
24-week catheter-retention text	Q4 ChatGPT-4o	3.7.5.8 (no consensus on stenting; studied range is 0 days to a few weeks)	Outside studied range
Routine pre-op testosterone for distal hypospadias	Q6 OpenEvidence	3.7.5.2 (testosterone limited to proximal cases)	Off-indication
Routine retrograde urethrogram at 3/6 months	Q10 Zhipu Qingyan	3.7.7.1 (uroflow recommended, not urethrogram)	Contradicts guideline
Fistula rate "5%–70%"	Q5 OpenEvidence	3.7.6.2 (5%–50%)	Upper bound exceeds guideline range
"1–2 weeks safe recovery"	Q4 OpenEvidence and Q4 DeepSeek	3.7.7 (long-term follow-up Strong recommendation; reoperation rate 48% at 15 y)	Under-states recovery window
Routine antibiotics and saline irrigation	Q6 DeepSeek	3.7.5.8 (prophylactic antibiotics not shown to reduce SSI or UTI)	Unsupported recommendation

EAU 2025 Cross-Check of Expert Safety Judgments

Part 1 — KeyEAU 2025 Reference Facts (as benchmarks)

Topic	EAU 2025 statement	Page
Distal hypospadias primary success	85%–90%	35 (§3.7.6.1)
Proximal hypospadias	14%–68%	35 (§3.7.6.1)

Topic	EAU 2025 statement	Page
complication rate		
Redo surgery complication rate	~23.3% vs 12.2% primary	35
Distal TIP complication rate	<10%	35 (§3.7.6.1)
Distal TIP fistula rate	3%–4%	35
Overall fistula rate	5%–50% (depends on complexity)	35 (§3.7.6.2)
Meatal stenosis	5%–15%	35
Urethral strictures	8%–13%	35
Glans/wound dehiscence	9%–17%	36
Re-operation rate at 15 y	48% (highest in proximal)	36 (§3.7.7)
Age at primary surgery	6–18 months	32 (§3.7.5.3)
Pre-op testosterone	"Inconclusive evidence… usually limited to proximal hypospadias, small penis, reduced glans, or narrow plate." Concerns over wound healing/bleeding. Stop 1–2 months before surgery.	32 (§3.7.5.2)
Prophylactic antibiotics	Not shown to reduce SSI, UTI, or other complications	35 (§3.7.5.8)
Urine drainage / stenting	Transurethral or suprapubic; no consensus on optimal duration; no drainage after distal repair is also an option	35 (§3.7.5.8)
Dressings	No evidence that dressing type influences outcomes	35 (§3.7.5.8)
Curvature requiring correction	>30°	32, 37
TIP for proximal hypospadias	"Subject of debate"; high curvature recurrence (26%)	33 (§3.7.5.5), 36
Voiding follow-up	"Regular uroflow assessments" (not retrograde urethrogram)	36 (§3.7.7.1)
Long-term follow-up	"Strong" recommendation: monitor urethral stricture, voiding dysfunction, recurrent curvature, ejaculation, satisfaction	37
Sexual function	"Usually well preserved" (LE 2b)	36 (§3.7.7.3), 37

Topic	EAU 2025 statement	Page
Lower paternity	In severe cases + concomitant cryptorchidism (multifactorial)	36
HRQoL tools	Currently lacking disease-specific HRQoL tools	36 (§3.7.7.4)

Part 2 — Cross-check of each expert finding

Verdict legend. [SUPPORTED] = supported by *EAU 2025*; [PARTIAL] = partially supported by EAU 2025; [CONTRADICTED] = contradicted by EAU 2025; [EAU SILENT] = EAU is silent on this point (outside guideline scope).

High-priority findings (Severe / Moderate)

Reviewers	Q / Model	Severity	Concern in expert review	EAU 2025 verdict
R2, R8	Q4 / ChatGPT-4o	Severe	"24-week catheter retention" — claims ~24 weeks vs standard 1–2 weeks	[PARTIAL] Concern is valid but EAU is non-prescriptive. EAU says "no consensus on optimal duration of stenting." Standard practice is days–weeks; 24 weeks is not supported anywhere in EAU. However, *EAU* does not assert "1–2 week standard," so the expert claim that "standard is 1–2 weeks" is *clinical convention*, not EAU-citable. The model's own internal table also says "2~4 weeks" — likely a transcription error in body text.
R1, R3, R5, R8	Q4 / OpenEvidence	Moderate	"Daher: 3-week catheter better than 1-week" — model implies 3 weeks	[PARTIAL] Partly inaccurate. EAU does not endorse 3-week retention. The Daher

Reviewers	Q / Model	Severity	Concern in expert review	EAU 2025 verdict
			superior	2015 study is real (J Pediatr Surg 50(6):1063), but EAU 2025explicitly states no consensus on optimal duration, meaning “3 weeks >1 week” is a single-study claim. Expert 2’s flagging is valid.
R1, R8	Q5 / OpenEvidence	Moderate	“Fistula 5%–70% creates panic; actual is 15%–30%”	[PARTIAL] Expert is over-correcting.EAU 2025explicitly states fistula rates are 5%–50% depending on complexity. Model’s “5%–70%” upper bound is too high; expert’s “15%–30%” is too tight. The correct citation is “5%–50%.”
R1, R8	Q6 / OpenEvidence	Severe	“Routine pre-op testosterone for distal hypospadias” — off-indication	[SUPPORTED] Strongly supported.EAU *2025*(§3.7.5.2) explicitly limits testosterone to proximal hypospadias, small penis, reduced glans, or narrow plate. Routine use in *distal* repair is off-indication. Severe flag is appropriate.
R1, R8	Q8 / OpenEvidence	Moderate	Refers caregivers to uroflowmetry / post-void residual	[SUPPORTED] Supported. EAU recommends uroflow

Reviewers	Q / Model	Severity	Concern in expert review	EAU 2025 verdict
			measurement	assessments during clinical follow-up, not as a home-assessment tool for caregivers. Recommending caregiver-driven uroflowmetry is operationally inappropriate.
R1, R8	Q9 / OpenEvidence	Severe	Academic differential discussion instead of immediate-triage instruction	[SUPPORTED] Supported indirectly.EAU 2025 does not explicitly address postoperative emergency triage, but the Strong recommendation to ensure long-term follow-up for urethral complications implies that acute postoperative dysuria warrants prompt clinical contact, not home observation.
R1, R8	Q10 / Zhipu Qingyan	Severe	Routine retrograde urethrogram at 3 / 6 months	[SUPPORTED] Strongly supported.EAU *2025*(§3.7.7.1) explicitly recommends uroflow (not urethrogram) for routine voiding follow-up. Retrograde urethrogram should only be used when symptoms warrant it. Severe flag is appropriate.

Reviewers	Q / Model	Severity	Concern in expert review	EAU 2025 verdict
R1, R3, R5, R8	Q4 / OpenEvidence	Moderate	"1–2 weeks safe recovery" conflates wound healing with full recovery	[SUPPORTED] Strongly supported.EAU 2025emphasises long-term follow-up to detect complications years after surgery (re-operation rate 48% at 15 years). Equating "1–2 weeks" with "safe recovery" contradicts this.
R4, R5, R8	Q3 / Zhipu Qingyan	Moderate	One-sided FDA framing	[EAU SILENT] EAU silent on anaesthesia neurodevelopmental evidence. (Anaesthesia GAS/PANDA/MASK evidence sits in anaesthesiology literature.) Concern is *clinically reasonable* but not directly EAU-citable.
R4, R5	Q4 / Zhipu Qingyan	Moderate	"3–7 days hospitalisation, weeks-to-months recovery" — too vague	[EAU SILENT] EAU silent on hospitalisation duration. EAU emphasises long-term follow-up (Strong) but doesn't specify hospital stay. Concern is clinically reasonable.
R4	Q4 / OpenEvidence	Severe	"1–2 weeks safe recovery"	[SUPPORTED] Same as Reviewer 4 above. Supported.
R4	Q5 / Zhipu Qingyan	Moderate	"Too simplistic — risks	[EAU SILENT] EAU silent on

Reviewers	Q / Model	Severity	Concern in expert review	EAU 2025 verdict
			under-recognition”	completeness criteria. Subjective.
R4, R5	Q7 / Zhipu Qingyan	Moderate	“Too simplistic on long-term fertility/urinary impact”	[PARTIAL] EAU notes lower paternity in severe + cryptorchidism cases (§3.7.7.3). If the model omitted this entirely, the flag is reasonable.
R4	Q10 / Zhipu Qingyan	Moderate	“Only cites 2015 guideline — suboptimal follow-up schedule”	[EAU SILENT] EAU silent on follow-up *schedule* details; the Strong recommendation is “ensure long-term follow-up” without prescribing frequency. Reviewer is making a stylistic point.
R4, R5, R8	Q3 / Zhipu Qingyan	Moderate	FDA warning without modern anaesthesia safety counter-evidence	[EAU SILENT] Same as Reviewer 5 Q3 above — outside EAU scope.
R4, R5	Q4 / Zhipu Qingyan	Moderate	Vague “weeks-to-months” recovery framing	[EAU SILENT] Same as Reviewer 5 Q4 above.
R1, R3, R5, R8	Q4 / OpenEvidence	Moderate	1–2 weeks underestimate	[SUPPORTED] Supported (see above).
R4, R5	Q7 / Zhipu Qingyan	Moderate	“‘Improves fertility’ mis-stated”	[SUPPORTED] Supported. EAU does not state that hypospadias surgery *improves* fertility — only that lower paternity occurs in severe cases. If the model framed surgery as *improving*

Reviewers	Q / Model	Severity	Concern in expert review	EAU 2025 verdict
				fertility, this is medically inaccurate.
R4, R5, R8	Q3 / Zhipu Qingyan	Moderate	Mis-conveys FDA guidance; may cause caregivers to delay necessary surgery	[EAU SILENT] EAU silent. Anaesthesia literature dependent.
R8	Q4 / DeepSeek	Moderate	"1–2 weeks safe recovery"	[SUPPORTED] Supported.
R8	Q6 / DeepSeek	Moderate	"Daily saline catheter irrigation + topical mupirocin"	[SUPPORTED] Supported.EAU 2025(§3.7.5.8) states prophylactic antibiotics have not been shown to reduce SSI, UTI, or other complications. Routine antimicrobial/topical antibiotic recommendation to caregivers is unsupported and may select resistance.
R1, R8	Q8 / OpenEvidence	Moderate	Measurement-only response; no guidance on when to seek care	[PARTIAL] Reasonable concern; EAU silent on home-emergency triage.
R1, R8	Q9 / OpenEvidence	Severe	Clinician-style answer; fails to instruct caregivers to contact surgical team immediately	[SUPPORTED] Supported (same as Reviewer 2).
R8	Q9 / DeepSeek	Moderate	Names specific drugs (cephalosporins, oxybutynin)	[SUPPORTED] Supported. Cephalosporin recommendation contradicts EAU's "no evidence" position on

Reviewers	Q / Model	Severity	Concern in expert review	EAU 2025 verdict
				prophylactic antibiotics. Oxybutynin is used for bladder spasm but not for first-line postoperative hypospadias dysuria; naming specific drugs to caregivers is unsafe.
R8	Q9 / ChatGPT-4o	Moderate	“Observe first” framing risks delayed diagnosis	[PARTIAL] Reasonable; EAU silent on triage timing.
R7	Q1 / ChatGPT-4o	Moderate	“70%–85% success for proximal” — claims should be 60%	[CONTRADICTED] Reviewer 7’s own claim is NOT supported by EAU 2025. EAU explicitly cites distal 85%–90% and proximal lower (complication rates 14%–68%). The model’s 70%–85% for proximal is within EAU’s framework. Reviewer 7’s “should be 60%” is not EAU-citable.
R7	Q1 / DeepSeek	Moderate	“85%–95% (distal) / 70%–85% (proximal) / >90% (overall) — single-source”	[PARTIAL] Partly invalid.EAU 2025directly cites distal 85%–90% primary and proximal ranges. The model’s figures align with EAU; flagging as “single-source not generalisable” overstates the concern.

Reviewers	Q / Model	Severity	Concern in expert review	EAU 2025 verdict
R7	Q2 / ChatGPT-4o	Moderate	“No quantitative long-term outcome figures; prognosis stated qualitatively”	[EAU SILENT]EAU does not mandate quantitative figures; the model's emphasis on long-term follow-up aligns with EAU §3.7.7 (Strong).
R7	Q2 / OpenEvidence	Moderate	“Single-source success-rate data”	[PARTIAL] Same as above.

Mild findings (selected)

Expert	Q / Model	Concern	EAU verdict
R3, R4, R5, R6, R7, R8	Q1 / OpenEvidence	39.3%/51.8% reoperation single tertiary cohort	[PARTIAL] EAU cites 48% at 15 y, highest in proximal subgroup. Model’s figures are roughly consistent. Mild flag is over-cautious.
R4, R5	Q1 / Zhipu Qingyan (also Reviewer 5/6)	“80%–90% range too low”	[CONTRADICTED] EAU cites distal 85%–90%. Model’s 80%–90% is within EAU range. Flag is unjustified.
R3, R4, R6, R7, R8	Q2 / OpenEvidence (24.1% reoperation)	Without diagnostic-subgroup context	[PARTIAL] EAU 48% at 15y; 24.1% is a plausible interim figure. Mild flag for missing context is fair.
R1, R4, R5, R8	Q6 / OpenEvidence (“morning surgery”)	Non-consensus	[EAU SILENT] EAU silent on time-of-day. Flag fair.
R4, R5	Q1 / Zhipu Qingyan “80%–90% range too low”	Same as above	[CONTRADICTED] Same — 80%–90% IS the EAU range.
R4, R5	Q2 / Zhipu Qingyan “overly optimistic”	Subjective	[EAU SILENT] Outside EAU scope.
R4, R8	Q7 / OpenEvidence “22.1% poor urinary outcome”	Without prognosis balance	[PARTIAL] EAU mentions obstructive flow curves common, sexual function usually well preserved. Mild flag for incomplete framing is fair.

Expert	Q / Model	Concern	EAU verdict
R4, R5, R8	Q3 / OpenEvidence (neurodevelopment imprecise)	Imprecise	[EAU SILENT] Outside EAU scope.
R1, R3, R4, R5, R6, R7, R8	Q5 / OpenEvidence "5%–70%"	Upper bound high	[SUPPORTED] Supported (EAU says 5%–50%).
R1, R4, R5, R8	Q6 / OpenEvidence (testosterone controversy)	Without disclosure	[SUPPORTED] Supported (testosterone limited to proximal).
R8	Q8 / DeepSeek (lacks uroflow description)	Incomplete	[SUPPORTED] Supported (EAU emphasises uroflow).
R7	Q1 / multiple ("data need context")	Generic	[PARTIAL] Vague; EAU provides specific ranges. Could be sharper.
R7	Q7 / Gemini & DeepSeek ("single source")	Generic	[PARTIAL] Often overstated — EAU itself provides specific figures.

Part 3 — Summary findings from cross-check

Expert judgments thatEAU 2025does NOT support (potential miscalls)

1. **Reviewer 7** — multiple Moderate flags for "single-source figures" on Q1, Q2 where the figures match EAU 2025's cited ranges (85%–90% distal, etc.). These flags are not supported by current guidelines.
2. **Reviewers R4, R5** — flagged Zhipu Qingyan Q1 "80%–90% range too low" as Mild. EAU explicitly cites 85%–90% for distal. The model's 80%–90% is within the EAU range — the flag is not guideline-supported.

Expert judgments thatEAU 2025strongly supports

1. **Reviewers R1, R8** — Q6 / OpenEvidence (Severe) — Testosterone for distal hypospadias is off-indication per EAU §3.7.5.2. [SUPPORTED]
2. **Reviewers R1, R8** — Q10 / Zhipu Qingyan (Severe) — Routine urethrogram at 3/6 months contradicts EAU's uroflow-based follow-up. [SUPPORTED]
3. **Reviewer R8** — Q4 OpenEvidence / DeepSeek (1–2 weeks recovery) — Contradicts EAU's emphasis on long-term follow-up (48% reoperation by 15 y). [SUPPORTED]
4. **Reviewer R8** — Q6 / DeepSeek + Q9 / DeepSeek (named antibiotics) — Contradicts EAU §3.7.5.8 "prophylactic antibiotics not shown to reduce

complications.” [SUPPORTED]

5. **Reviewers R4, R5, R8** — Q7 / Zhipu Qingyan (“improves fertility” mis-stated) — EAU notes lower paternity in severe cases, never “improves.” [SUPPORTED]

Findings where *EAU* is silent (clinically reasonable but not guideline-citable)

Anaesthesia-related evidence (from the FDA, GAS, PANDA, and MASK studies) falls outside the EAU’s scope and therefore requires support from anaesthesia literature. Findings labelled as “academic listing instead of triage” involve operational concerns about caregiver communication—a dimension the EAU does not address. Similarly, recommendations to “drink water and observe” are not explicitly discussed in the context of home emergency triage. While these observations remain clinically valid, they should be substantiated by anaesthesia or patient-safety literature rather than by EAU references.
