# Supplementary material for: Performance of 5 Large Language Models in Perioperative Consultation for Pediatric Hypospadias: Cross-Sectional Comparative Study
Source: J Med Internet Res. 2026 Jul 29;28:e93393. doi: 10.2196/93393 (PMC13419283; doi:10.2196/93393)
Supplement: Checklist 1 [file jmir-v28-e93393-s014.docx]

**CHART reporting checklist**

The CHART (Chatbot Assessment Reporting Tool) checklist was developed to standardise reporting of studies evaluating health-related chatbots and large language models. The mapping below identifies the section, appendix, or other location in the manuscript that addresses each CHART item.

| Item | Topic | Reported in |
| --- | --- | --- |
| 1 | Title and abstract — chatbot evaluation framing, study design, key findings | Title; Abstract (Background/Objective/Methods/Results/Conclusion) |
| 2 | Background — rationale, prior chatbot evaluations, study question | Introduction (Problem; Review of Relevant Scholarship) |
| 3 | Specific objectives or hypotheses | Hypothesis, Aims, and Objectives; Abstract Objective |
| 4 | Study design | Methods, Conditions and Design (prospective non-interventional cross-sectional) |
| 5 | Setting — location, timeframe | Methods, Sampling Procedures (West China Hospital of Sichuan University; April 2025) |
| 6 | Eligibility criteria for participants and evaluators | Methods, Inclusion and Exclusion |
| 7 | Sources and methods of participant selection | Methods, Sampling Procedures (convenience sampling) |
| 8 | Sample-size determination and power | Methods, Sample Size, Power, and Precision (no a-priori calculation; post-hoc CIs) |
| 9 | Chatbot/LLM identifier, version, configuration | Multimedia Appendix 1 |
| 10 | Access route (URL, app, API) | Multimedia Appendix 1 |
| 11 | Browsing/grounding/retrieval-augmentation status | Multimedia Appendix 1 |
| 12 | System prompts, temperature, other parameters | Multimedia Appendix 1 |
| 13 | Prompt set (full set of input prompts) | Multimedia Appendix 1 |
| 14 | Date of model interaction | Multimedia Appendix 1; Methods, Selection of Large Language Models and Access Protocol (April 6, 2025) |
| 15 | Output handling and pre-processing | Methods, Question Bank and Risk Stratification; LLM Response Generation |
| 16 | Evaluation instrument and dimensions | Multimedia Appendix 6 |
| 17 | Rater identity, training, and calibration | Methods, Evaluation Implementation; Quality of Measurements |
| 18 | Blinding | Methods, Masking; Evaluation Implementation (per-question randomised AI labels) |
| 19 | Outcome measures (primary and secondary) | Methods, Primary Outcome Measures; Secondary Outcome Measures |
| 20 | Reference-authenticity verification protocol | Methods, Secondary Outcome Measures; Multimedia Appendix 2 |
| 21 | Clinical-safety assessment protocol | Methods, Secondary Outcome Measures; Multimedia Appendices 3 and 13 |
| 22 | Statistical methods | Methods, Analytic Strategy |
| 23 | Effect sizes and 95% CIs | Methods, Analytic Strategy; Results tables; Multimedia Appendices7,9,10,11,12 |
| 24 | Multiplicity and multiple-testing correction | Methods, Analytic Strategy (Bonferroni alpha' = .005) |
| 25 | Missing data handling | Methods, Analytic Strategy; Data Diagnostics (no missing data) |
| 26 | Sensitivity analyses | Results, Post-Stratification Weighted Sensitivity Analysis |
| 27 | Subgroup analyses (with multiplicity) | Results, Heterogeneity Effects of Population Characteristics; Multimedia Appendices 9 and 10 |
| 28 | Participant flow | Figure 1 (JARS Participant Flowchart); Results, Participant Flow |
| 29 | Baseline characteristics | Table 1; Results, Baseline Participant Characteristics |
| 30 | Outcome data (omnibus, pairwise, and dimensional) | Figures 2-4; Multimedia Appendices 7-12 |
| 31 | Citation and reference-accuracy results | Table 2; Multimedia Appendix 2 |
| 32 | Clinical-safety results | Table 3; Multimedia Appendices 3 and 13 |
| 33 | Reproducibility statement (time-stamping, configuration specificity) | Methods, Selection of Large Language Models and Access Protocol (final paragraph); Discussion, Generalizability |
| 34 | Interpretation linking findings to objectives | Discussion, Support of Original Hypotheses (opening) |
| 35 | Comparison with prior literature | Discussion, Similarity of Results |
| 36 | Limitations | Discussion, Interpretation |
| 37 | Generalisability and external validity | Discussion, Generalizability |
| 38 | Implications, future research, RAG, and prompt engineering | Discussion, Implications; Disclosure of Delegation to Generative AI |
| 39 | Funding | Funding |
| 40 | Generative-AI use disclosure | Disclosure of Delegation to Generative AI |
| 41 | Ethics approval, consent, and data protection | Methods, Ethical Considerations |
| 42 | Conflicts of interest | Conflicts of Interest |
| 43 | Registration | Methods, Conditions and Design; Informed Consent (registration exemption justified) |

Note: All 43 CHART items are addressed. Items where the standard recommendation could not be fully met are explicitly disclosed: item 8 (no a-priori sample-size calculation), item 33 (no temporal-reproducibility re-query), and item 43 (retrospective trial registration and justification for exemption).
